# Lysophosphatidic Acid Receptor 3 (LPA3): Signaling and Phosphorylation Sites

**DOI:** 10.3390/ijms25126491

**Published:** 2024-06-12

**Authors:** K. Helivier Solís, M. Teresa Romero-Ávila, Ruth Rincón-Heredia, J. Adolfo García-Sáinz

**Affiliations:** 1 Departamento de Biología Celular y Desarrollo, Instituto de Fisiología Celular, Universidad Nacional Autónoma de México, Ciudad Universitaria, Ap. Postal 70-600, Ciudad de México 04510, Mexico; samsonyte09@gmail.com (K.H.S.); tromero@ifc.unam.mx (M.T.R.-Á.); 2 Unidad de Imagenología, Instituto de Fisiología Celular, Universidad Nacional Autónoma de México, Ciudad Universitaria, Ap. Postal 70-600, Ciudad de México 04510, Mexico; rrincon@ifc.unam.mx

**Keywords:** lysophosphatidic acid, LPA, lysophosphatidic acid receptor 3, LPA_3_, phosphorylation sites, signaling

## Abstract

LPA_3_ receptors were expressed in TREx HEK 293 cells, and their signaling and phosphorylation were studied. The agonist, lysophosphatidic acid (LPA), increased intracellular calcium and ERK phosphorylation through pertussis toxin-insensitive processes. Phorbol myristate acetate, but not LPA, desensitizes LPA_3_-mediated calcium signaling, the agonists, and the phorbol ester-induced LPA_3_ internalization. Pitstop 2 (clathrin heavy chain inhibitor) markedly reduced LPA-induced receptor internalization; in contrast, phorbol ester-induced internalization was only delayed. LPA induced rapid β-arrestin–LPA_3_ receptor association. The agonist and the phorbol ester-induced marked LPA_3_ receptor phosphorylation, and phosphorylation sites were detected using mass spectrometry. Phosphorylated residues were detected in the intracellular loop 3 (S221, T224, S225, and S229) and in the carboxyl terminus (S321, S325, S331, T333, S335, Y337, and S343). Interestingly, phosphorylation sites are within sequences predicted to constitute β-arrestin binding sites. These data provide insight into LPA_3_ receptor signaling and regulation.

## 1. Introduction

Lysophosphatidic acid (LPA) is a simple lipid containing a glycerol moiety esterified to a phosphate group at position 3 and a fatty acid at position 2. It is considered a “bioactive lipid”, implying that, in addition to its metabolic roles, it modulates a wide range of cellular, organ, and whole animal responses through a series of six G protein-coupled receptors, i.e., the LPA receptor family [1,2,3]. They have the characteristic seven transmembrane hydrophobic domains connected by three intracellular loops and three extracellular loops, with an extracellular amino-terminal group and an intracellular carboxyl terminus. These receptors are subdivided into those belonging to the lysophospholipid receptors (LPA_1–3_) and those related to the purinergic family (LPA_4–6_) [1,2,4].

The present work is focused on the LPA_3_ receptor (previously named EDG 7 [5]), which is known to interact mainly with two different G protein types, G_αi/o_ and G_αq/11_, likely involved in adenylyl cyclase inhibition, and calcium mobilization, triggering different signaling processes via their α and βγ subunits (reviewed in [1,2,3,4,5,6]); other G proteins might also be involved in LPA_3_ actions [7,8]. It is generally accepted that in addition to the immediate membrane signaling, many G protein-coupled receptors exert an endosomal second wave of signaling (reviewed in [9,10,11,12,13]); to our knowledge, information on the molecular events in LPA_3_ endosomal signaling has not been documented. In contrast, there is essential and abundant information on the participation of LPA_3_ receptors in many physiological functions and pathological processes. These include chemotaxis [14], migration [15], proliferation [16], differentiation [17], embryo implantations [18], determination of vertebrate left–right patterning during embryogenesis [19], and cardiac dysfunction [20,21], among many others (see also [6,22]). The relevant role of LPA and its receptors in cancer has been recently reviewed (see [23] and references therein). It is worth mentioning that an increase in the LPA_3_ receptor action has been observed in some cancer cell lines, such as rat neuroblastoma cells [24] or rat hepatoma [25]. In addition, LPA_3_ receptor expression seems to be directly related to resistance to chemotherapy, metastases, and, therefore, aggressiveness in some cancers, such as lung cancer [26], breast cancer [27], or pancreatic cancer [28]. In ovarian cancer, LPA_3_ expression is considered a therapeutic target and a poor prognosis marker [16,29]. 

Despite its physiological and pathophysiological relevance, many aspects of the signaling and regulation of the function of this receptor remain poorly explored or unknown. Receptor phosphorylation seems to be an early event participating in the desensitization of G protein-mediated signaling, endocytosis, endosomal traffic, and triggering of the second wave of signaling [9,10,11,12,13,30]. Phosphorylation of LPA_3_ receptors in response to the agonist, LPA, and to activation of protein kinase C (PKC) by phorbol esters has already been reported [31]. However, the key elements determining the consequences of receptor phosphorylation seem to be the individual sites or clusters phosphorylated; the process is commonly called the “phosphorylation barcode hypothesis” [13,30,32,33,34]. In silico analysis has allowed the suggestion of putative phosphorylation sites [6,35], but no experimental evidence is available. In the present work, we provide evidence to fill the mentioned knowledge gaps. We expressed the human LPA_3_ receptor employing a cellular model system that allows abundant receptor expression in an inducible fashion [36]. We characterized various signaling events using these cells, immunopurified the receptors, and used mass spectrometry to detect LPA_3_ phosphorylation sites experimentally.

## 2. Results

**Figure 1:**
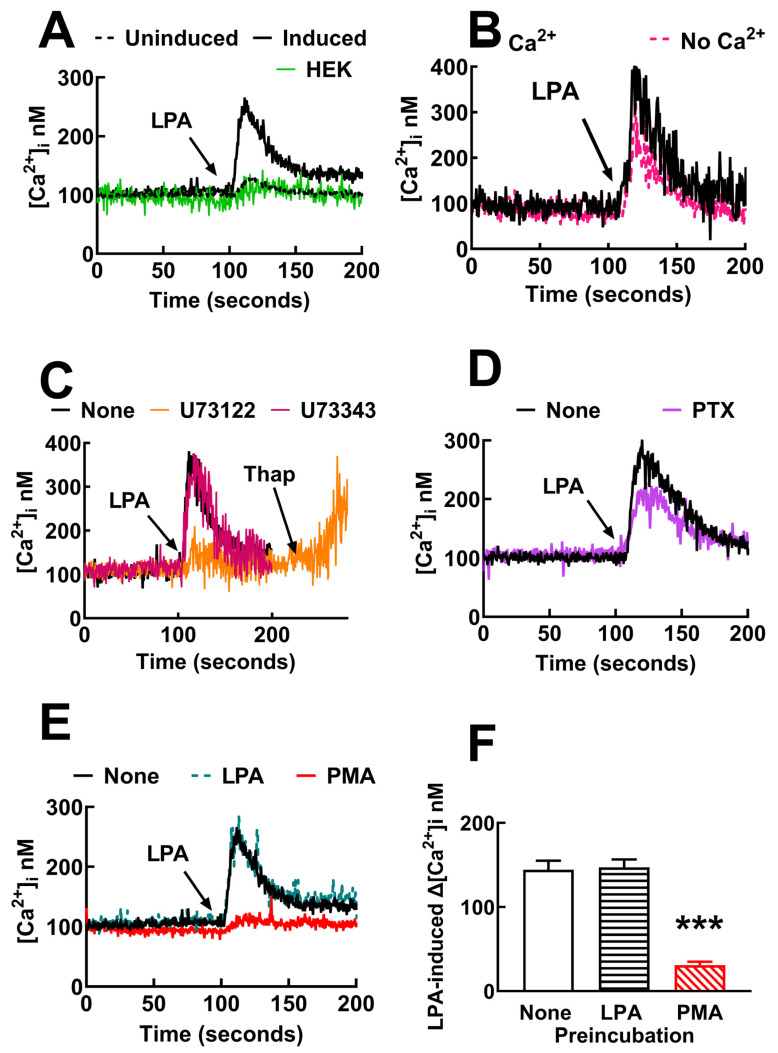
LPA_3_ receptors expressed are functional and markedly sensitive to heterologous but not to homologous desensitization. Panel (**A**), Representative calcium tracings in response to 1 µM LPA (arrow) in HEK 293 cells (green line) and HEK293 TREx TM transfected to express LPA_3_ receptors (induced, black continuous line, and not induced, black dotted line). Panel (**B**), calcium response to 1 µM LPA (arrow) of LPA_3_-expressing cells incubated in buffer with (black line) or without (red dotted line) calcium. Panel (**C**), intracellular calcium tracings of LPA_3_-expressing cells preincubated for 30 min in the absence of any agent (black line) or presence of 10 µM U73122 (phospholipase C inhibitor, orange line) or 10 µM U73343 (inactive analog, red line) and challenged with 1 µM LPA (arrow). Thapsigargin 1 µM (Thap, second arrow). Panel (**D**), calcium tracings of induced cells preincubated overnight without (black line) and with pertussis toxin (purple line); arrow indicates the addition of 1 µM LPA. Panel (**E**), calcium tracings in response to 1 µM LPA of cells preincubated without any agent (black line) or with 1 µM LPA for 5 min and washed before re-challenging (black dotted line) with 1 µM PMA (PMA, red line). Panel (**F**), calcium tracings in response to LPA of cells preincubated without any agent (None, open bar), with 1 µM LPA for 5 min and washed before re-challenging (LPA, dashed bar) or with 1 µM PMA (PMA, red dashed bar). The means are plotted, and vertical lines indicate the SEM of 4–5 experiments performed on different days and distinct cell cultures. *** *p* < 0.001 vs. baseline.

The effect of LPA on intracellular calcium was tested in HEK 293 TREx cells; a concentration of 1 µM LPA was selected based on concentration–response curves performed using this cellular model [37]. As shown in Figure 1A, the lysophospholipid induced a very small (≈20 nM) increase in calcium concentration in non-transfected cells. A similar effect was observed in transfected cells in which receptor expression was not induced (Figure 1A). In contrast, a robust increase in the intracellular calcium concentration (≈200–300 nM) was induced by 1 µM LPA in transfected cells in which LPA_3_ receptor expression was induced (Figure 1A); these data indicate that the LPA_3_ receptors were functional. 

The role of calcium entry in the action of LPA was tested. When cells were incubated in buffer without calcium, the LPA-induced increase in the calcium response was only marginally decreased (352 ± 61 nM and 328 ± 92 nM in cells incubated in buffer with and without calcium, respectively; *n* = 4, using cells from different cell cultures). The data indicate a predominant role of intracellular calcium mobilization in the effect of the lysophospholipid (Figure 1B). Preincubation for 30 min with the phospholipase C inhibitor, U73122 (10 µM) [38,39], essentially abolished the action of LPA (the increases in calcium concentration were: 306 ± 46 nM without inhibitor and 23 ± 13 with U73122; n = 3, *p* < 0.001), but thapsigargin was still able to increase intracellular calcium (Figure 1C). The inactive analog, U73343 [38,39], did not alter the LPA action under the same conditions (increase 363 nM).

Afterward, we examined the effect of pertussis toxin, a well-known blocker of G_i/o_. Surprisingly, the LPA-induced increase in intracellular calcium concentration was not diminished even using preincubation for 24 h with 300 ng/mL (usually we employ 100 ng/mL) (Figure 1D). As a control, we examined pertussis toxin action in cells expressing the LPA_1_ receptor. Supplementary Figure S1A shows that LPA_1_-expressing cells have a robust calcium response to the lysophospholipid and that such effect was completely blocked by preincubation with 300 ng/mL pertussis toxin. These data suggest that these two LPA receptor subtypes exhibit distinct preferences in their coupling to the various types of G proteins. To further characterize the G protein(s) involved, we employed YM-254890, a cyclic depsipeptide Gαq inhibitor [40,41]. Surprisingly, cell preincubation for 1 min with 1 µM YM-254890 [41] essentially eliminated the calcium response to LPA in cells expressing either LPA_1_ or LPA_3_ receptors; in both cases, thapsigargin was able to increase intracellular calcium (Supplementary Figure S2). Lower YM-254890 concentrations (100–300 nM) only marginally diminished LPA-induced calcium response in cells expressing LPA_1_ or LPA_3_ receptors.

It is known that either receptor activation by agonists or stimulation of unrelated receptors or signaling proteins, such as some protein kinases, can induce receptor desensitization. They are usually named homologous and heterologous desensitization, respectively. To explore this, cells were incubated with 1 µM LPA for 5 min and washed three times with the Krebs-Ringer solution (Section 4.3) to remove the LPA present. The cells were resuspended in the same buffer and stimulated with 1 µM LPA. As shown in Figure 1E, preincubation with the agonist did not induce any detectable homologous desensitization. On the contrary, pretreatment with the PKC activator, PMA (1 µM), for 5 min markedly diminished the effect of 1 µM LPA (≈80% reduction) (Figure 1E,F); i.e., the data evidenced heterologous desensitization. Data in Supplementary Figure S1B,C showed that cells expressing the LPA_1_ receptors are subjected to marked homologous and heterologous desensitization. 

We considered the possibility that low expression of G protein-coupled receptor kinases (GRKs) could be responsible for the essentially absent, homologous desensitization of the LPA_3_ receptors. However, in experiments in which GRK2 or GRK5 were overexpressed, agonist activation did not induce any significant desensitization of calcium signaling (Supplementary Figure S3). Similarly, in cells where LPA_3_ expression was decreased by induction with only 10 ng/mL doxycycline hyclate, absence of agonist-induced LPA_3_ desensitization was observed.

**Figure 2:**
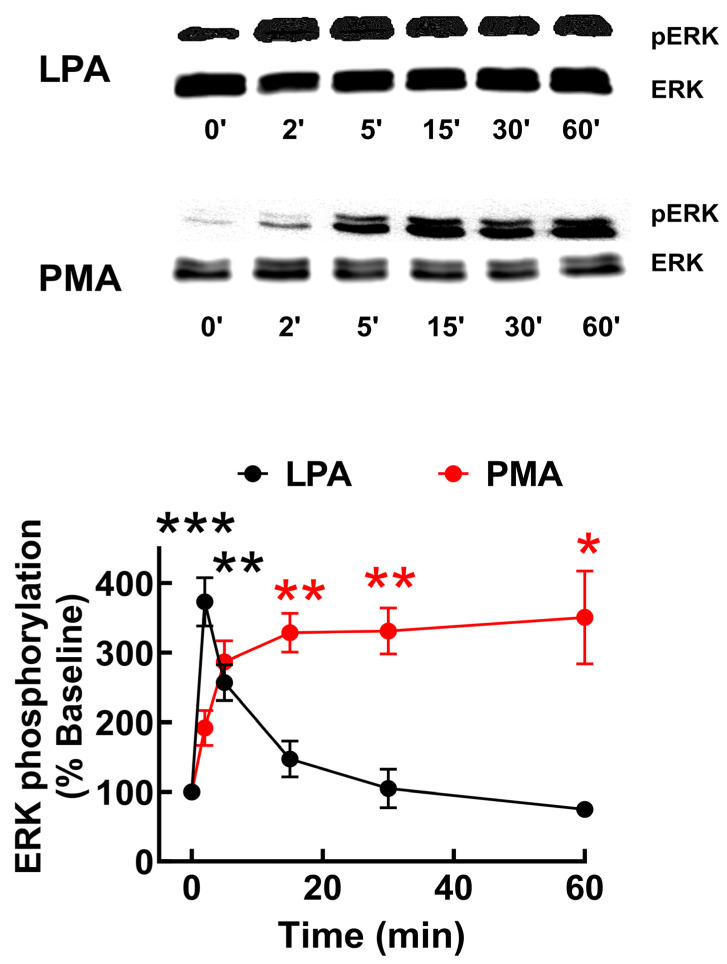
Time-course of LPA- and PMA-induced ERK 1/2 phosphorylation in LPA_3_ receptor-expressing cells. Cells were stimulated with 1 µM LPA (black symbols and line) or 1 µM PMA (red symbols and line). The mean is plotted, and vertical lines indicate the SEM of 7–8 experiments performed on different days and distinct cell cultures. *** *p* < 0.0001, ** *p* < 0.001, and * *p* < 0.01 vs. baseline value. Representative Western blots are presented above the figure.

The increase in intracellular calcium is a transient but immediate response to LPA_3_ receptor stimulation. ERK 1/2 phosphorylation, a lengthier LPA action, was also studied to explore signaling further. LPA_3_ activation with 1 µM LPA resulted in a rapid increment in ERK 1/2 phosphorylation, which was maximal at 2 min and progressively decreased to near-baseline values (Figure 2). In contrast, 1 µM PMA increased ERK1/2 phosphorylation more slowly, reaching a near maximum level at 5 min, and remaining at this phosphorylation state during the incubation (60 min) (Figure 2); PMA is known to activate the mitogen-activated protein kinase pathway through PKC [42].

Intrigued by the insensitivity to pertussis toxin of the calcium response to LPA, we assessed the effect of this toxin in the ERK1/2 phosphorylation response. In parallel experiments, LPA_3_-expressing cells were preincubated with or without 300 ng/mL pertussis toxin and then challenged with 1 µM LPA for the times indicated; samples were also run in the same gel and Western blot analysis performed in the same membranes (Supplementary Figure S4). As observed, treatment with pertussis toxin decreased baseline (time 0 min) ERK 1/2 phosphorylation, but the time courses of LPA action were very similar (i.e., essentially identical if the respective baselines were normalized to 100%). Therefore, the data indicate that G_αi_ blockade with pertussis toxin barely affects the agonist LPA_3_ receptor-mediated ERK phosphorylation response. 

The possibility that internalization of the LPA_3_ receptor could participate in ERK phosphorylation was explored employing Pitstop 2, an inhibitor of the terminal domain of clathrin heavy chains [43,44]. Pretreatment for 15 min with 10 µM Pitstop 2 markedly decreased (almost eliminated) baseline ERK 1/2 phosphorylation and the effect of LPA at times studied (Supplementary Figure S5). In contrast, the effect of Pitstop 2 on PMA-mediated action showed a different pattern. The marked decrease in baseline ERK phosphorylation was confirmed in these experiments, but PMA markedly and consistently increased ERK phosphorylation in cells pretreated with Pitstop 2, although the effect was noticeably delayed (Supplementary Figure S6). The effect of EGF was also tested; it was observed in parallel experiments that the action of the peptide growth factor was only marginally affected by Pitstop 2 at the time studied (15 min) (Supplementary Figure S7). 

The effect of the Gαq inhibitor, YM-254890, on LPA-induced ERK phosphorylation was explored in cells expressing LPA_1_ or LPA_3_ receptors. As shown in Supplementary Figure S8, this agent decreased the action of LPA on ERK phosphorylation in cells expressing LPA_1_ or LPA_3_ receptors; however, the inhibition was more noticeable in the LPA_3_-expressing cells.

**Figure 3:**
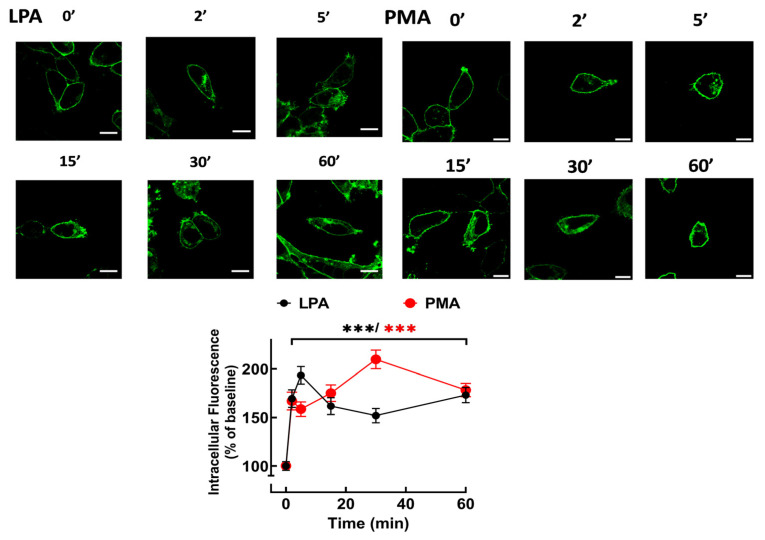
Time-course of LPA- and PMA-mediated LPA_3_ internalization. Cells were incubated for the times indicated with 1 µM LPA (black symbols and line) or 1 µM PMA (red symbols and line). Internalization is presented as the percentage of baseline intracellular fluorescence. The mean is plotted, and vertical lines indicate the SEM of 14 images obtained from 5 experiments performed on different days and cell cultures. *** *p* < 0.001 vs. baseline value. Representative images are presented above the graph. Bars 10 µm.

Accumulation of fluorescence in intracellular vesicles in response to the agonist, LPA, or the PKC activator, PMA, was observed, indicating receptor internalization (Figure 3). These actions took place rapidly (detected as early as 2 min) and were sustained during the 60 min incubations (Figure 3). Supplementary videos show the effects of LPA (Video S1) and PMA (Video S2) on receptor internalization, see Supplementary Legends for the Videos file. These videos allowed observation of cell displacement (LPA) and marked changes in form, including cell contraction (roundness). They also allow the detection of clusters of fluorescence forming “pearl-necklace”-like structures, formation of lamellipodia and blebs, and other changes in membrane appearance. Experiments were also performed, inducing lower LPA_3_ expression (1 ng/mL doxycycline), and under these conditions, both LPA and PMA induced also marked receptor internalization. The cell nucleus was stained with DAPI in these cells, and fluorescent vesicles occupied most of the cytoplasm in LPA- or PMA-stimulated cells (Supplementary Figure S9).

Cells expressing the LPA_3_ receptors preincubated with Pitstop 2 notedly exhibit decreased intracellular fluorescence before stimulation (Supplementary Figures S10 and S11). When cells not preincubated with Pitstop 2 were stimulated with LPA, internalization was observed as early as 2 min and maintained throughout the experiment (Supplementary Figure S10). As indicated, Pitstop 2 decreased baseline fluorescence; LPA increased such baseline value, but the effect was smaller than that observed in the absence of the inhibitor (Supplementary Figure S10). PMA-induced LPA_3_ receptor internalization was quick and sustained in cells preincubated without any agent. In cells preincubated with Pitstop 2, internalization was similar to that observed without the inhibitor but departing from a different baseline (Supplementary Figure S11). These data suggest that a clathrin-independent process might also be involved in receptor internalization, pointing to differences between LPA-mediated and PMA-mediated internalization.

**Figure 4:**
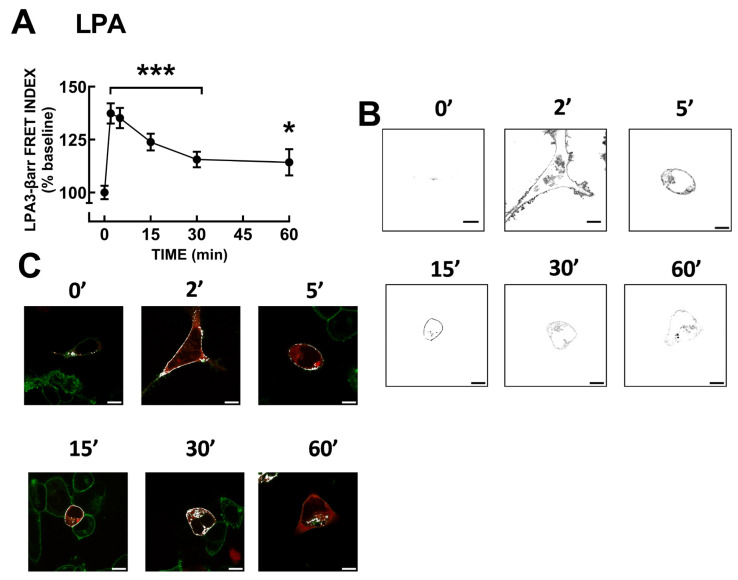
LPA-induced LPA_3_ receptor–β-arrestin interaction. Panel (**A**), Time-course of LPA_3_ receptor–β-arrestin interaction as reflected by FRET. Cells were challenged with 1 µM LPA at time 0. The means are plotted, and vertical lines indicate the SEM of 5–6 experiments performed on different days and distinct cell cultures. *** *p* < 0.0001 and * *p* < 0.05 vs. baseline value. Panel (**B**), Representative FRET images. Panel (**C**), Representative images showing colocalization (white) of the LPA_3_–eGFP receptors (green) and β-arrestin–mCherry (red). Bars 10 µm.

It is well-known that β-arrestins play a role in G protein-coupled receptor desensitization, internalization, and signaling [9,10,11,12,13]. Therefore, LPA_3_–β-arrestin 2 interaction was studied utilizing FRET. It was observed (Figure 4A,B) that LPA induced a rapid receptor–arrestin interaction (maximal at 2 min), which decreased progressively but maintained the signal clearly above the baseline during the experiment (60 min). Supplementary Figure S12 shows the images obtained exciting the LPA_3_–eGFP(enhanced Green Fluorescent Protein) construct (green channel), the β-arrestin-mCherry construct (red channel), raw FRET, and the FRET channel images (these are images of the same cells presented in Figure 4). Images of LPA_3_–β-arrestin colocalization are also presented (Figure 4C); Pearson coefficient indicates that in the baseline condition, there was no clear colocalization (<0.5), whereas after stimulation with LPA colocalization, it was evident (Pearson coefficients > 0.9). Videos showing raw LPA-induced FRET (not FRET channel) (Video S3) and colocalization (Video S4) are presented as Supplementary Materials.

**Figure 5:**
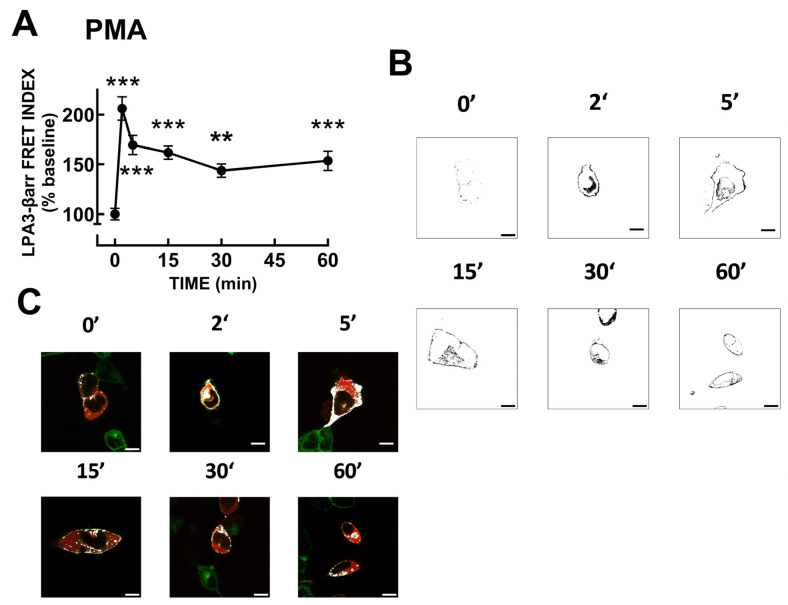
PMA-induced LPA_3_ receptor–β-arrestin interaction. Panel (**A**), Time-course of LPA_3_ receptor–β-arrestin interaction as reflected by FRET. Cells were challenged with 1 µM PMA at time 0. The means are plotted, and vertical lines indicate the SEM of 5–6 experiments performed on different days and distinct cell cultures. *** *p* < 0.001 and ** *p* < 0.01 vs. baseline value. Panel (**B**), Representative FRET images. Panel (**C**), Representative images showing colocalization (white) of LPA_3_–eGFP receptors (green) and β-arrestin–mCherry (red). Bars 10 µm.

LPA_3_–β-arrestin FRET was also studied using 1 µM PMA. The protein kinase C activator also induced a rapid increase in signal followed by a slow decrease (Figure 5A,B). Supplementary Figure S13 shows the images obtained exciting the LPA_3_–eGFP construct (green channel), the β-arrestin–mCherry construct (red channel), raw FRET, and the FRET channel images (these are images of the same cells presented in Figure 5). In Figure 5C, LPA_3_–β-arrestin colocalization images are presented; it can be observed that PMA also induced colocalization. We explored, afterward, LPA_3_ receptor phosphorylation in response to 1 µM LPA or 1 µM PMA. A representative whole membrane autoradiograph is presented in Supplementary Figure S14. 

**Figure 6:**
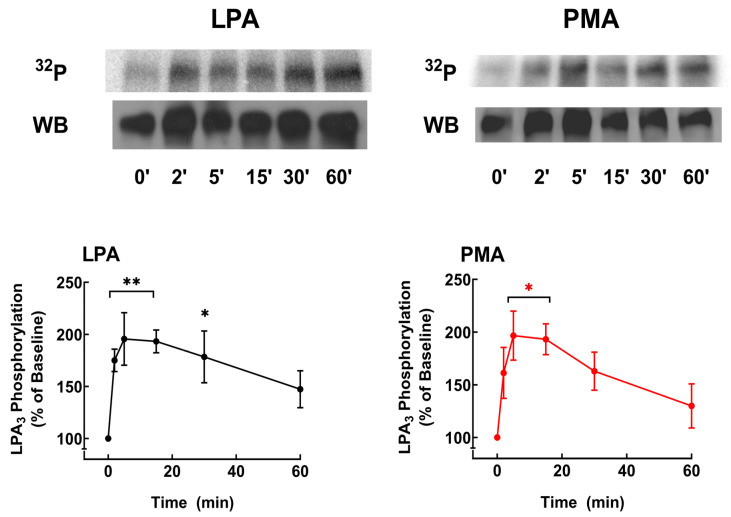
LPA- and PMA-induced LPA_3_ receptor phosphorylation. Cells were challenged with 1 µM LPA or 1 µM PMA at time 0. Receptor phosphorylation is expressed as the percentage of the baseline value. The means are plotted, and vertical lines indicate the SEM of 9 experiments performed on different days using different cell cultures. ** *p* < 0.01, * *p* < 0.05 vs. baseline value. Representative autoradiographs (^32^P) and Western blots (WB) are presented above the graph.

The time courses of the action of these agents are presented in Figure 6. It can be observed that both LPA and PMA induced a rapid increase in LPA_3_ phosphorylation; the effects were of similar magnitude and reached their maximum at 5–10 min of stimulation, decreasing progressively after that and maintaining phosphorylation states above baseline up to the end of the incubation (60 min). These data are consistent with previous observations [31,45]. In silico analyses of putative phosphorylation sites in LPA receptors have already been reported [6,35]. Nevertheless, we repeated such analysis using updated versions of available software systems: GPS, PhosphoSVM, NetPhos, and MusiteDeep (see Section 4.9), and the results are presented in Table 1. Putative phosphorylation sites with high scores include one site in the intracellular loop 2, five sites in the intracellular loop 3, and ten in the carboxyl terminus. The table indicates putative protein kinases potentially participating in the phosphorylation of these residues. Not surprisingly, isoforms of G protein-coupled receptor kinases (GRKs) and PKC, as well as other kinases, might play a role. 

**Figure 7:**
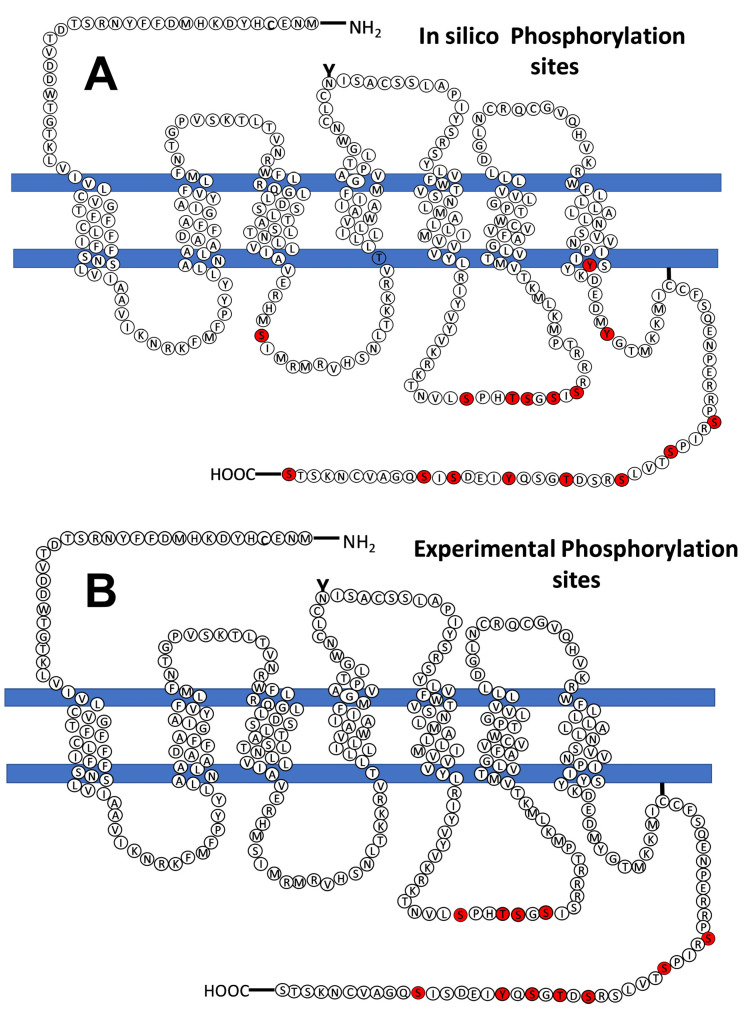
Models showing the LPA_3_ receptor phosphorylation sites predicted in silico (panel (**A**)) and obtained experimentally (mass spectrometry, panel (**B**)). Phosphorylation sites are indicated in red.

LPA_3_ receptors from cells not treated with any agent, 1 µM LPA or 1 µM PMA for 15 min, were immunopurified, and samples were sent to the Taplin Mass Spectrometry Facility (Harvard Medical School). The representative gel and blot are presented in Supplementary Figure S15. Table 2 summarizes data from distinct experiments and conditions. It should be noted that the mass spectrometry analyses were not quantitative and only indicated sites that were detected phosphorylated but not their abundance in the samples. Mass spectrometry spectra are available in the Supplementary Mass Spectrometry Spectra file. In our studies, four sites were detected in the intracellular loop 3 (S221, T224, S225, and S229), and seven in the carboxyl terminus (S321, S325, S331, T333, S335, Y337, and S343). Many, but not all, the experimentally detected phosphorylation sites were predicted by the in silico analyses (Table 2), and similarly, not all predicted sites were experimentally detected. The prediction programs identified, with high score, the following number of sites: GPS, ten of the eleven experimentally detected sites (90%, S331 not predicted); PhosphoSVM also ten (90%, S331 not predicted); NetPhos, nine sites (81%, S331 and Y337 not predicted); and MusiteDeep, only six of them (S229, S331, T333, S335, and S343 not predicted). A cartoon showing the putative, in silico, and experimentally found sites in the LPA_3_ receptor is presented in Figure 7. It is worth mentioning that the coverage of peptide detection was 100% for the intracellular loop 3 in all the experiments, and that of the carboxyl terminus ≈70–75% in four, and only 40% in one of the five separate experiments performed. The carboxyl terminus tripeptide (STS) was not detected in any experiment.

**Figure 8:**
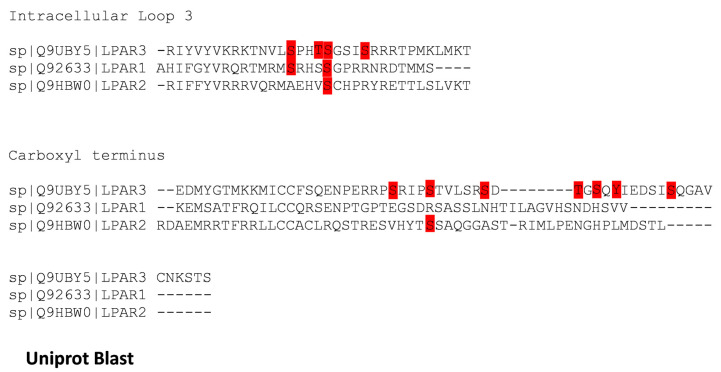
Sequence alignment of the intracellular loop 3 and the carboxyl terminus of the three human LPA receptors belonging to the lysophosphatidic acid family. Amino acids detected phosphorylated in mass spectrometry in the LPA_3_ receptors or conserved in the other subtypes are marked in red.

**Figure 9:**
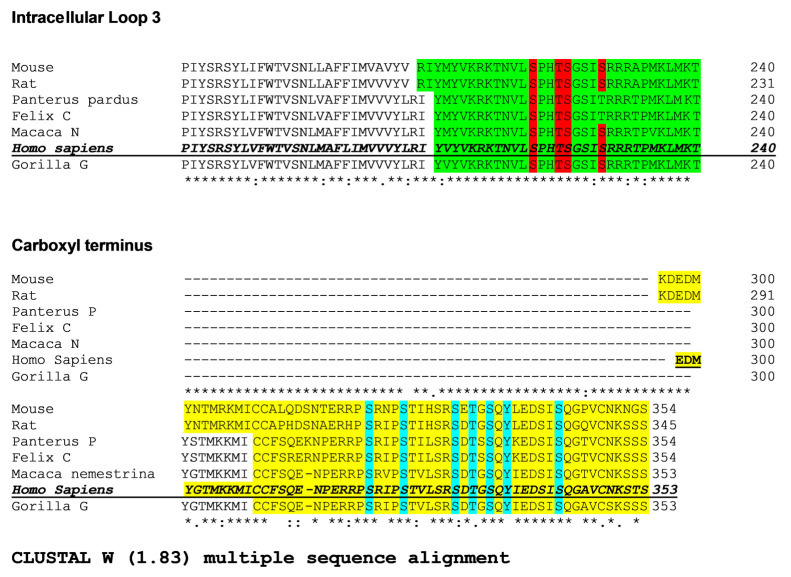
Sequence alignment of the intracellular loop 3 and the carboxyl terminus of the three human LPA receptors belonging to the lysophosphatidic acid family. Conserved amino acids detected phosphorylated (human) among LPA_3_ receptors orthologs. The intracellular loop 3 of the distinct orthologs is indicated in green, and the conserved (compared to the human ortholog) phosphorylated amino acids are marked in red. The carboxyl terminus is indicated in yellow, and the conserved phosphorylated amino acids are marked in blue.

Sequence alignment of the intracellular loop 3 and carboxyl termini of LPA_1–3_ receptors is presented in Figure 8. It can be observed that only S225 (intracellular loop 3) of the human LPA_3_ is conserved in all three of the human lysophospholipid LPA receptor subtypes. Two residues are conserved in two of these receptors: (a) S221 (intracellular loop 3, LPA_3_, and LPA_1_) and (b) S325 (carboxyl terminus, LPA_3_, and LPA_2_) (Figure 8). In contrast, the alignment of the available LPA_3_ receptor ortholog sequences revealed that in the intracellular loop 3, (Figure 9) three phosphorylated residues are conserved in all species studied, and S229, which is not conserved in the feline orthologs, was substituted by T (a phosphorylatable amino acid, conserved substitution). All seven phosphorylated residues in the carboxyl terminus are conserved among the species studied (Figure 9).

To validate that the phosphorylation sites detected in the mass spectroscopy studies were those phosphorylated in cellulo, we employed cells expressing a mutant receptor in which the amino acids found phosphorylated were substituted by non-phosphorylatable residues (i.e., serines were substituted with alanines and threonines with valines). The mutant receptor construct was fluorescent and functional, as evidenced by the ability of LPA to increase intracellular calcium. As anticipated, baseline receptor phosphorylation was markedly diminished (≈70%) in cells expressing the mutant receptor compared to those expressing the wild-type, and no significant effect of LPA or PMA was detected (Supplementary Figure S16). It should be mentioned that the mutant receptor migrated more in the electrophoresis experiments, which could be due to proteolysis (that we cannot discard) or to the substitutions made to the sequence. Supplementary Figure S17 shows representative images of cells expressing the wild-type (panel A) and the mutant receptor (panel B). It can be observed that fluorescence was very similar in these cells, although more LPA3–GFP receptors were detected intracellularly in cells expressing the mutant (panel C). Immunoprecipitated receptors detected by Western blotting also indicated that the receptor abundance was similar in cells expressing these receptors (panel D). It seems unlikely that differences in receptor expression might explain the marked changes in receptor phosphorylation observed. Evidence on the functional effect of substitution of the sites found phosphorylated for non-phosphorylatable amino acids in the LPA3 receptor has recently been published [37]. In the case of the mutant receptor studied here, it was observed that LPA could increase intracellular calcium with similar efficacy but less apparent potency, and both agonist-induced LPA3 receptor–β-arrestin interaction and agonist-induced receptor internalization were markedly decreased [37].

## 3. Discussion

**Figure 10:**
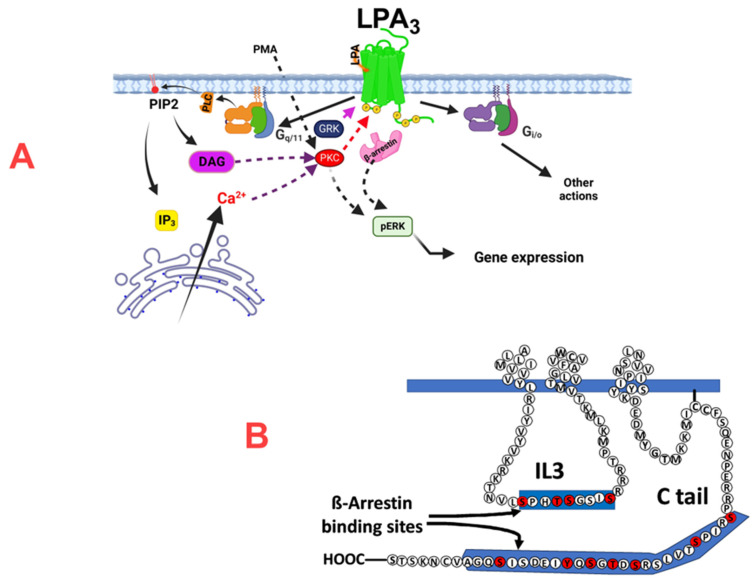
Cartoon depicting the possible mechanism of action of LPA_3_ receptors and the possible role of phosphorylation sites in β-arrestin binding. Panel (**A**) shows that LPA_3_ receptors couple to G_q/11_ and G_i/o_ and the finding that it is through G_q/11_ that agonist activation of this receptor subtype mainly triggers calcium signaling and ERK phosphorylation. In Panel (**B**), the possible role of the phosphorylation sites detected (marked in red) in the IL3 and Ctail domains in β-arrestin binding is suggested. IL3, intracellular loop 3; Ctail, carboxyl terminus; PLC, phospholipase C; PIP2, phosphatidylinositol 4,5-bisphosphate, DAG, diacylglycerol, GRK, G protein-coupled receptor kinase; PKC, protein kinase C.

LPA receptors can be coupled to several G proteins; in particular, LPA_1_ and LPA_3_ receptors can stimulate the G_αq_ and G_αi/o_ proteins [6,22]. Activation of LPA receptors, including LPA_3_, increase intracellular calcium in cells endogenously expressing these receptors and in transfected cells (see, for example, [2,4,5,22,31,45]). LPA_1_ receptor stimulation elevates intracellular calcium concentration mainly through the stimulation of G_i/o_ proteins, as reflected by the marked sensitivity to pertussis toxin [45,46], a control employed in the present work. Interestingly, we observed that the LPA_3_ receptor-mediated increase in intracellular calcium was poorly sensitive to pertussis toxin, indicating that G_i/o_ proteins are not the primary mediators of this response. These data are consistent with the early observation of Bandoh et al. [5], who reported that the LPA_3_-mediated calcium response in insect Sf9 cells was insensitive to pertussis toxin but blocked by the phospholipase C blocker U73122. We attempted to define the role of G_q_ in this action by using the inhibitor YM-254890 [41,47], which prevents G_q_ protein-coupled receptor signaling by blocking GTP/GDP exchange [41,47]. However, this agent blocked the LPA calcium response in cells expressing either LPA_1_ or LPA_3_ receptors, which precluded a more precise definition of the G protein involved. Consistent with our previous work using transfected C9 cells [31], we observed that LPA-activated LPA_3_ receptors induced marked but transient ERK phosphorylation, and we show here that this response was also insensitive to pertussis toxin, which indicates that it is not mainly mediated through G_i_. The action of YM-254890 was also assessed on ERK phosphorylation using LPA_1_- and LPA_3_-expressing cells. The inhibitor decreased the LPA-induced ERK response in both cells, but the effect was more robust in cells expressing the LPA_3_ receptors. The selectivity of the G_q_ blocker has been questioned [48], and we cannot discard the possibility that YM-254890 might have actions at levels other than G proteins. Therefore, our present data suggest a role of G_q_ in these LPA_3_ effects (intracellular calcium and ERK phosphorylation) but did not permit a clear experimental demonstration. Obviously, our data do not exclude roles of G_i_ in other LPA_3_ receptor-mediated actions. Figure 10 (panel A) depicts some of these data interpretations. 

As anticipated by previous work [31], PKC activation with PMA markedly blocked the calcium response observed in cells expressing LPA_1_ or LPA_3_ receptors. The possibility that protein kinase activation might alter calcium signaling at other steps in the process cannot be completely ruled out. Our data also confirmed that LPA_3_ receptors were less sensitive to agonist-induced (homologous) desensitization. In our previous work [31], we observed reduced desensitization, whereas no homologous desensitization was detected in the present work. This difference is likely due to the cellular models used (C9 cells [31] and Flp-In TREx HEK 293 cells in this work) that express different repertoires of signaling elements and the fact that the inducible model expresses a high density of receptors (required for the receptor purification studies). This difference in desensitization is probably relevant to the roles of LPA_3_ receptors in cancer, particularly in those involving interaction with high concentrations of this lysophospholipid, such as ovarian cancer [6,16,22,29]. It is worth mentioning that the LPA_3_ receptor has been suggested as a therapeutic target and biomarker for ovarian cancer [29].

Arrestins are a family of proteins that bind to phosphorylated receptors and participate in desensitization, signaling, internalization, and many other functions [9,10,12]. The LPA_3_ receptors–β-arrestin 2 interactions observed in the FRET and colocalization experiments suggest that in the baseline state, β-arrestin is distributed in the cytoplasm; upon stimulation with LPA, β-arrestin interacts with plasma membrane-located LPA_3_ receptors, and after a short time, the FRET signal diminished (although remained above baseline during the 60 min of incubation), and the colocalization images suggest that some receptors internalize colocalizing with β-arrestin. G protein-coupled receptors have been divided into two classes based on the stability of the interaction with arrestins [49,50]. Class A receptors have a transient interaction with β-arrestin and translocate with this protein to clathrin-coated pits; once internalized, the receptor and β-arrestin dissociate. Class B receptors’ interaction with β-arrestin is more stable, and the receptors internalize accompanied by β-arrestin [49,50]. Our data indicate that the LPA_3_ receptors have many characteristics of Class A receptors but also have a feature of Class B receptors (i.e., apparently, some receptors maintain interaction with β-arrestin after internalization and FRET and colocalization signals decrease but do not vanish). The possibility that classification might not be binary but that receptors with intermediate characteristics might exist is suggested. It is worth reminding that colocalization and FRET are different parameters reflecting distinct aspects. Colocalization has a resolution of ≈ 200 nm, and objects could be at distinct optical planes, whereas to obtain a FRET signal, a distance of <10 nm should exist between fluorophores. 

We confirmed [31] the ability of LPA and PMA to induce LPA_3_ receptor internalization. Pitstop 2 inhibits the terminal domain of clathrin-heavy chains [43,44] and markedly decreases LPA-induced LPA_3_ internalization. The action of PMA on receptor internalization was delayed but essentially followed the same pattern of cells incubated without the inhibitor. Similarly, Pitstop 2 markedly decreased LPA-induced ERK phosphorylation but only delayed the action of PMA. The parallelism of these processes is consistent with the idea that complexes involving receptors, β-arrestin, clathrin, the endocytic machinery, and MAP kinases are involved in both events [51]. 

LPA_3_ receptor phosphorylation was confirmed in the present study. This work’s central objective was to characterize the phosphorylated LPA_3_ residues. We detected sites in the intracellular loop 3 and carboxyl terminus with reproducibility and a high A score [52]. Determination of these sites opens the possibility to study their functional impact. Nevertheless, further work will be required to define this in the two domains. This final goal will help better understand this receptor’s function and regulation, which is relevant considering its roles in health and disease. It is worth mentioning that phosphorylation codes for β-arrestin recruitment by G protein-coupled receptors have been discovered [53] (see also http://tools.vai.org/phoscofinder/downloads.php, accessed on 10 March 2019), which allows finding putative β-arrestin binding sites in receptors. Several of these sites were reported for the LPA_3_ receptors [53] located in the carboxyl terminus within the region S321–A346. Other sites were detected using the “phoscofinder” webpage in the intracellular loop 3 within the sequence S221–S229. All phosphorylated residues in the mass spectrometry studies were within these regions. These aspects are depicted in Figure 10 (panel B).

It is worth noticing that the lack of conservation of the phosphorylation sites was far from surprising. Previous work has shown that phosphorylation sites are not conserved among the α-adrenergic subtypes [54,55,56]. In contrast, the phosphorylation sites were conserved among receptor orthologs, which is also similar to what has been observed for α_1_-adrenoceptor subtypes [54,55,56]. 

The present experiments provide insight into the actions and regulation of LPA_3_ receptors. Limitations of this work include using a single cellular model, i.e., Flp-In TREx HEK293 cells. We previously studied LPA_3_ receptor phosphorylation, internalization, and desensitization in C9 cells [31]; some differences in these studies were observed, which is not unexpected [33,34] since receptor action and regulation depend on the proteins expressed, which is cell-specific. However, in both cellular systems, the LPA_3_ receptor was overexpressed. Unfortunately, to our knowledge, there is no suitable cellular model to study LPA_3_ action and regulation since most cells express various receptor subtypes for lysophosphatidic acid. In the present work, LPA3 receptors were tagged at the carboxyl terminus with the eGFP, a commonly used tag to study G protein-coupled receptors [57,58,59]. However, evidence indicates that tags might alter receptor function [60]. Although our data (intracellular calcium) were comparable to those obtained in RH7777 cells transfected with untagged LPA3 receptors [61], and we did not detect any anomalous receptor function, we cannot exclude the possibility that the tag might have altered some of the responses.

Another limitation is the use of PMA to study heterologous desensitization and internalization. One advantage of PMA is that most of its actions are mediated through the activation of PKC isoforms; however, there are other targets for active phorbol esters and diacylglycerol [62,63]. Nevertheless, we have previously shown that PKC inhibitors and down-regulation of some isoforms block the ability of PMA to induce LPA_3_ phosphorylation and block LPA_3_-mediated increases in intracellular calcium [31]. These data strongly indicate that these actions are mediated through PKC but do not allow us to exclude the roles of other phorbol ester-targets completely; similarly, actions secondary to PKC cascade activation might alter signaling processes (such as ERK phosphorylation studied in this work) [42]. In addition, sustained activation of PKC is induced by phorbol esters, whereas diacylglycerol action seems to be transient under physiological conditions. Despite these limitations, unavoidable due to pharmacological and molecular biological strategies, the present finding provides new insights into LPA_3_-triggered signaling pathways, actions, and regulation.

In our opinion, the phosphorylation experiments with the mutant receptor validate that the sites detected were those mainly phosphorylated in cellulo. However, defining the functional roles of the phosphorylation sites detected in the intracellular loop 3 and carboxyl terminus is an experimental challenge. Understanding the roles of different phosphorylation domains, clusters of phosphorylation sites, and even individual phosphor amino acids seems relevant, considering the importance of this receptor in physiology and pathophysiology. Experiments on this aspect are currently being performed in our laboratory.

## 4. Materials and Methods

### 4.1. Reagents

1-Oleyl lysophosphatidic acid (LPA) was from Cayman Chemical Co. (Ann Arbor, MI, USA). Phorbol 12-myristate-13-acetate (PMA), cyanogen bromide-activated agarose, thapsigargin, the phospholipase C inhibitor U73122 and its inactive analog U73343, as well as Pitstop 2 (N-[5-[(4-Bromophenyl) methylene]-4,5-dihydro-4-oxo-2-thiazolyl]-1-naphthalene-sulfonamide) and free fatty acid-free bovine serum albumin (fraction V), were obtained from Sigma-Aldrich (St. Louis, MO, USA). YM 254890 was obtained from Wako Chemicals (Richmond, VA, USA) (lot: WDK3168). Dulbecco’s modified Eagle’s medium, trypsin, streptomycin, penicillin, amphotericin B, blasticidin, hygromycin B, doxycycline hyclate, and Fura-2 AM were purchased from Invitrogen-Life Technologies (Carlsbad, CA, USA). Fetal bovine serum was obtained from BioWest (Nuaillé, France). Pertussis toxin was purified from vaccine concentrates [64]. [^32^P]P_i_ (8500–9120 Ci/mmol) was obtained from Perkin-Elmer Life Sciences-New England Nuclear (Boston, MA, USA). Polyvinylidene difluoride membranes were purchased from BioRad (Hercules, CA, USA), Lipofectamine 2000 (catalog number 11668-019, lot 1854318), and SuperSignal West Pico Chemiluminescence kits were purchased from Thermo Fisher Scientific (Waltham, MA, USA). DAPI (4′,6-diamidino-2-fenilindol), anti-GRK2 (G protein-coupled receptor kinase 2) rabbit polyclonal antibody (catalog number SC 562, Lot F248), and anti-GRK5 (G protein-coupled receptor kinase 2) rabbit polyclonal antibodies (catalog number SC 565, lot D1912) were obtained from Santa Cruz Biotechnology (Santa Cruz, CA, USA) and employed as indicated by the supplier. Agarose-coupled protein A was obtained from Merck-Millipore (Burlington, MA, USA). Anti-phospho-ERK 1/2 (Thr202/Tyr204) (catalog number 9101S, Lot: 30) and anti-total ERK (p42/44) antibodies (catalog number 4695S, Lot: 21) were from Cell Signaling Technology (Danvers, MA, USA). Monoclonal anti-GFP (green fluorescent protein) antibodies were from Clontech (JL-8; catalog number 632381, Lot: A8034133) (Mountain View, CA, USA), and polyclonal anti-GFP antibody was generated in our laboratory [31,54,55]. Antibodies were obtained from a polyclonal anti-GFP antisera by ammonium sulfate salting-out. Following the supplier indications, the dialyzed IgGs were coupled to cyanogen bromide-activated agarose beds. This anti-GFP coupled agarose was used for the immunopurification procedure. Primary antibodies were used at a dilution of 1:2000, whereas secondary antibody dilution was 1:10,000. The peroxidase affiniPure Goat anti-mouse IgG light-high chain antibody and other secondary antibodies were purchased from Zymed (Thermo Fisher Scientific; (Waltham, MA, USA) or Jackson ImmunoResearch (West Grove, PA, USA. Human embryonic kidney (HEK) 293 cells were obtained from the American Type Culture Collection (HEK293; ATCC CRL-1573) (Manassas, VA, USA). Parental Flp-In T-Rex HEK293 cells and the plasmid, pOG44, were obtained from Invitrogen (Carlsbad, CA, USA). The plasmid for the expression of the LPA_1_ receptor fused to the mCherry red fluorescent protein (plasmid ID EX-Z7377-M56) was obtained from Genecopoeia (Rockville, MD, USA). The plasmid for the expression of β-arrestin 2 mCherry-tagged was generously provided by Dr. Adrian J. Butcher (University of Leicester, Leicester, UK) [59]. Plasmids for the expression of GRK2 and GRK5 were generously provided by Dr. Jeffrey Benovic (Thomas Jefferson University [65], Philadelphia, PA, USA).

LPA (10 mM) was dissolved in culture medium supplemented with 0.1% fatty acid-free bovine serum albumin, and subsequent dilutions were made in water. PMA (10 mM) was dissolved in dimethyl sulfoxide (DMSO), and subsequent dilutions were also made in water (concentration of DMSO in contact with the cells 0.01%). The absence of an effect of the vehicle was checked in all experiments. In the FRET experiments, we notice that DMSO, per se, induced some transient changes in the signal. Therefore, for these experiments, PMA was dissolved in absolute ethanol (PMA 10 mM), and subsequent dilutions were made in water containing 10% ethanol (use of water or culture medium resulted in turbid suspensions). Ethanol in contact with the cells was 0.11% and was devoid of any effect on FRET; PMA dissolved this way was fully effective, as evidenced by its effect on LPA-induced increases in intracellular calcium.

### 4.2. Cell Lines and LPA_1_ and LPA_3_ Receptor Expression

The LPA_3_ receptor sequence was fused at the carboxyl terminus (Ctail) with the GFP and cloned into the pCDNA5/FRT/TO plasmid (Bioinnovatise, Inc., Rockville, MD, USA) to employ the inducible Flp-In TREx expression system [36]. A plasmid to express a mutant LPA_3_ receptor, in which the residues found phosphorylated in the mass spectrometry analysis were substituted by non-phosphorylatable residues, was obtained commercially, and proper substitution was confirmed by sequencing (Bioinnovatise, Inc., Rockville, MD, USA). The plasmids were transfected into parental Flp-In TREx HEK293 cells with the pOG44 plasmid using lipofectamine 2000. These cells were subjected to selection for one month with 10 µg/mL blasticidin and 100 µg/mL hygromycin B and grown in Dulbecco’s modified Eagle’s medium supplemented with 10% fetal bovine serum, 100 μg/mL streptomycin, 100 U/mL penicillin, and 0.25 μg/mL amphotericin B. Unless otherwise indicated, LPA_3_ receptor expression was induced with 10 µg/mL doxycycline hyclate for 12 h, and expression was confirmed using fluorescence microscopy and calcium signaling

In some experiments, cells expressing the LPA_1_ receptor were used for comparison. HEK 293 cells were cultured as previously described [66] and transfected with the plasmid to express the LPA_1_ receptor fused to the mCherry red fluorescent protein indicated above (Section 4.1). Cells were subjected to selection with G418 (initially, 900 µg/mL was used and subsequently decreased, for maintenance, to 300 µg/mL). A cell line was selected based on expression (fluorescence microscopy) and robust functional response (rise in intracellular calcium concentration).

### 4.3. Intracellular Calcium Concentration

Determinations were performed as previously described ([31,66]; see [67] for a detailed description). In brief, the cells (80–90% confluence) were serum-starved and treated for 12 h with 10 µg/mL doxycycline hyclate to induce LPA_3_ expression. After confirming the expression of this receptor construct (epifluorescence microscopy), the cells were loaded with 2.5 µM Fura-2 AM in Krebs-Ringer–Hepes containing 0.05% bovine serum albumin (pH 7.4) for 1 h. Cells were carefully detached from the Petri dish, washed to eliminate unincorporated dye, and maintained in suspension [67]. In the experiments to determine if extracellular calcium entry was involved in LPA action, the cell suspension was divided into two halves; one half was washed in the regular buffer and the other half in the same buffer but without calcium. Determinations were carried out in an AMINCO-Bowman Series 2 luminescence spectrometer (Thermo Electron Scientific Instruments, Madison, WI, USA) equipped to maintain the temperature at 37 °C and gentle, constant stirring of the cell suspension. Two excitation wavelengths (340 and 380 nm) and an emission wavelength of 510 nm were employed with a chopper interval of 0.5 s. Intracellular calcium levels were calculated as described by Grynkiewicz et al. [68]; maximal fluorescence by determined by lysing the cells with Triton X-100 (0.1%, final), and the minimal fluorescence signal by adding EGTA (5 mM, final) [67]. Only the intracellular calcium determinations were made in cells in suspension, and all other studies were performed in cells attached to the Petri dishes. 

### 4.4. ERK 1/2 Phosphorylation

The cells were serum-starved for 4 h and then stimulated with 1 µM LPA or 1 µM PMA (these concentrations were determined in preliminary experiments) for the times indicated; after this incubation, cells were washed twice with ice-cold phosphate-buffered saline and lysed [66] for 1 h on an ice bath; the lysates were centrifuged at 12,700× *g* for 15 min, and proteins contained in supernatants were denatured with Laemmli sample buffer [69] and separated by SDS-polyacrylamide gel electrophoresis. Proteins were electrotransferred onto polyvinylidene difluoride membranes, and immunoblotting was performed. Total- and phospho-ERK 1/2 levels were determined in the same membranes for each experiment; the baseline value was considered 100% for normalization.

### 4.5. LPA_3_ Receptor–β-Arrestin 2 Interaction

Cells expressing the LPA_3_–GFP construct were transfected with the β-arrestin 2–mCherry plasmid described above (Section 4.1) (500 ng/6 cm Petri dish). After 24 h, the cells were collected and seeded on glass-bottomed Petri dishes, and after an additional 48 h in culture, protein–protein interactions were studied in these cells. The LPA_3_–β-arrestin interaction was analyzed using Föster Resonance Energy Transfer (FRET), employing an FV10i Olympus microscope with an automated laser spectral scan (Olympus, Tokyo, Japan). The GFP was excited at 488 nm, and the emitted fluorescence was detected at 510 nm, whereas mCherry was excited at 580 nm and emitted fluorescence was detected at 610 nm; this was routinely performed, and images were obtained to check the expression of these proteins. For FRET channel analysis, GFP (but not mCherry) was excited, and fluorescence was detected at 610 nm; such fluorescence indicated that the proximity among the fluorescent proteins was enough to allow energy transfer (i.e., 1–10 nm) [70]. The FRET index was quantified using ImageJ software (version 1.49v) [71], which removes bleed-through and false FRET. The images were analyzed using 8 bits that permit pixel-by-pixel supervised computational FRET index analysis. The average FRET index obtained with the vehicle (time 0 min) was normalized as 100%. Individual cells (not clusters) expressing both fluorescent proteins were randomly selected; 10–14 cells were analyzed for each experimental condition in all the experiments.

### 4.6. Video Experiments

The video experiments employed a confocal Zeiss LSM800 microscope with a temperature- and atmosphere (CO_2_ and humidity)-controlled chamber (Zeiss, Jena, Germany). The expressions of eGFP- and mCherry-tagged proteins were confirmed for each experiment. The eGFP was excited at 488 nm, and the emitted fluorescence was detected at 490–570 nm, whereas mCherry was excited at 564 nm, and the fluorescence emitted was detected at 580–700 nm. In the receptor internalization experiments, only eGFP was excited, and emission was detected. In these experiments, recording (1.27 frames per second) was for 5 min (176 frames, no interval) during the baseline conditions, and then cells were stimulated with 1 μM LPA or 1 μM PMA. Recording under stimulated conditions was for 60 min (2182 frames, no interval). In the colocalization experiments, eGFP and mCherry were excited, and their fluorescence was recorded. Image merge was performed using the software included in the microscope. In these experiments, recording “Frame mode” (2.53 frames per second) was for 5 min (134 frames, no interval) during the baseline, and then cells were stimulated for 15 min (599 frames, no interval). In the FRET experiments, eGFP (but not mCherry) was excited, and fluorescence was detected in the red channel (580–700 nm); fluorescence at this range was considered raw FRET. Conditions (gain, contrast, brightness) were determined by allowing a minimum “background noise” that permits cell and nucleus delimitation. Such background was maintained constant in all and during all the experiments. In these studies, recording “Line mode” (1.27 frames per second) was for 5 min (185 frames, no interval) during the baseline, and under-stimulated conditions were for 15 min (556 frames, no interval). In all the video experiments, processing was performed with the FIJI program (a distribution of ImageJ) (version 2.14.0/1.54F; JAVA 1.8.0_322 (64 bits)) [72]. The video was saved in the “mp4” format, and the letterings “baseline”, “LPA”, or “PMA” were included to specify the condition being studied. The fact that cells and organelles move (i.e., migrate and change their form) during the experiments and can leave the plane of observation should be reminded. In addition, LPA is known to induce cell contraction and migration (see, for example, [73] and references therein). 

### 4.7. Receptor Internalization

Cells seeded at a low density were cultured on glass-bottomed Petri dishes for 12 h. After this, LPA_3_ receptor expression was induced by incubation with doxycycline (10 µg/mL) for an additional 12 h. Before the experiment, the cells were serum-fasted for 1 h (prolonged serum fasting (i.e., 4 h or more) resulted in diminished internalization). After this incubation, cells were stimulated with LPA or PMA for the times indicated. Cells were washed with phosphate-buffered saline and immediately fixed with 4% paraformaldehyde for 10 min. The images were obtained using a FluoView Confocal model FV10i microscope (Olympus); GFP was excited at 488 nm and emitted fluorescence registered at 515–540 nm. The plasma membrane was delineated using differential interference contrast images to determine receptor internalization. Each cell’s intracellular fluorescence (i.e., excluding the plasma membrane) was quantified as “integrated density”, employing the ImageJ software [71]. The procedure is described in detail for “Corrected total cell fluorescence” (in [66] and in The Open Lab Book (https://theolb.readthedocs.io/en/latest/imaging/measuring-cell-fluorescence-using-imagej.html, accessed on 10 January 2023). Usually, 10–14 images were taken from 3 or 4 cultures obtained on different days for each condition. In some experiments, doxycyclin was employed at a concentration of 10 ng/mL to reduce the amount of receptor expressed in the cells and allow a more evident detection of internalization.

### 4.8. Receptor Phosphorylation

The cells were incubated for 1 h in phosphate-free Dulbecco’s Modified Eagle’s media and 3 h in phosphate-free media supplemented with 50 μCi/mL [^32^P]P_i_. Labeled cells were treated with vehicle, LPA, or PMA, washed with ice-cold phosphate-buffered saline, and solubilized for 1 h in the lysis buffer [54,56]. The extracts were centrifuged, and the supernatants were incubated overnight with protein A agarose and the anti-GFP antiserum generated in our laboratory. Samples were washed five times, and the pellets were denaturalized with sample buffer [69]. Proteins were separated using SDS-polyacrylamide gel electrophoresis, electrotransferred onto nitrocellulose membranes, and exposed for 24 h. The amount of phosphorylated receptor was assessed by PhosphorImager analysis using the ImageQuant program version 5.0. Western blotting for loading controls was performed utilizing a commercial monoclonal anti-GFP antibody.

### 4.9. In Silico Analysis

In silico analyses were performed to determine putative phosphorylation sites in the sequence of the LPA_3_ receptor. These were carried out using the software Group-based Prediction System, GPS [74], versions 6.0 (http://gps.biocuckoo.cn; accessed on 23 March 2022), PhosphoSVM [75,76]: (http://sysbio.unl.edu/PhosphoSVM/; accessed on 10 May 2023) and NetPhos 3.1 (http://www.cbs.dtu.dk; accessed on 9 April 2023) and MusiteDeep (https://www.musite.net/; accessed on 10 May 2023). We considered only the sites with the highest phosphorylation score.

### 4.10. Immunopurification and Mass Spectrometric Analysis

Immunopurification was performed as previously employed for other receptors [54,55,56]. In brief, fifteen Petri dishes (10 cm) containing LPA_3_ receptor-expressing cells (with 95–100% confluence) were divided for the three experimental conditions studied (five Petri dishes each): baseline, LPA, or PMA. After the stimulation, cultures were washed twice with ice-cold phosphate-buffered saline and solubilized in a lysis buffer containing detergents, proteases, and phosphatase inhibitors [54,55,56] for 1 h in an ice bath. Cell lysates were centrifuged for 15 min at 4 °C, and the supernatant was added to a slurry of anti-GFP agarose beads and incubated with constant movement for 4 h at 4 °C. The pellets were washed five times with lysis buffer and solubilized with Laemmli sample buffer [69], and the samples were resolved using SDS-polyacrylamide gel electrophoresis. Finally, the bands corresponding to the LPA_3_–GFP construct (≈70 kDa, identified by Western blotting) were excised and sent to the Taplin Mass Spectrometry Facility (Harvard Medical School, Cambridge, MA, USA), where the analysis was performed. Five independent experiments, including all conditions, were performed and analyzed. Mass spectrometry spectra are available in Supplementary Mass Spectrometry Spectra file.

### 4.11. Statistical Analyses

The data are presented as the means ± standard errors of the means. Statistical analysis between comparable groups was performed using Student’s *t*-test when two groups were compared (Supplementary Figure S17C) or ordinary one-way ANOVA with the Bonferroni post-test when three or more groups were compared, employing the included software in the GraphPad Prism program (version 10.1.2). A *p*-value < 0.05 was considered statistically significant.

## Figures and Tables

**Table 1:** Receptor phosphorylation sites detected in silico. Protein kinases are abbreviated as suggested by Uniprot (https://www.uniprot.org/uniprotkb/P80885/entry, accessed on 9 April 2023). IL3, intracellular loop 3; IL2, intracellular loop 2; Ctail, carboxyl terminus.

Kinases	Site	Amino Acid	Sequence	Domain
CAMK, CAMKL CK1/VRK Pyk 2, JAK GRK 4 CAMK, MAPK PKC α, β, γ, δ TK TK PKA, PKC α, ζ, GRK 3 PKA, PKC α, ε, GRK 3, 5 Akt PKA GRK 2 AGC/GRK AGC/GRK PKC γ, δ, ζ, GRK 1, 2, 4	130 221 224 225 227 229 293 301 321 325 329 333 337 341 343 353	S S T S S S Y Y S S S T Y S S S	IAVERHMSIMRMRVH KRKTNVLSPHTSGS TNVLSPHTSGSISRR NVLSPHTSGSISRRR LSPHTSGSISRRRTP PHTSGSISRRRTPMK SVVNPIIYSYKDEDM SYKDEDMYGTMKKMI ENPERRPSRIPSTVL RRPSRIPSTVLSRSD RIPSTVLSRSDTGSQ TVLSRSDTGSQYIED RSDTGSQYIEDSISQ GSQYIEDSISQGAVC QYIEDSISQGAVCNK AVCNKSTS	IL2 IL3 IL3 IL3 IL3 IL3 Ctail Ctail Ctail Ctail Ctail Ctail Ctail Ctail Ctail Ctail

**Table 2:** Receptor sites detected phosphorylated by mass spectrometry. Conditions: baseline (B, no color background), and stimulations by 1 µM LPA (grey) or 1 µM PMA (red); the numbers inside the boxes indicate the times peptides were detected phosphorylated in the experiments.

Location	Amino Acid	B	LPA	PMA
221 * 224 * 225 * 229 * 321 * 325 * 331 333 * 335 337 * 343 *	S T S S S S S T S Y S	33 13 59 31 27 23 21 20	26 18 12 18 40 11 15 19	49 18 14 90 31 22 20 13 20

* Suggested in the in silico analyses.

## Data Availability

The data presented in this study are available upon request from the corresponding author.

## Supplementary Materials

The following supporting information can be downloaded at: https://www.mdpi.com/article/10.3390/ijms25126491/s1.
