# Supplementary material for: Lysophosphatidic Acid Receptor 3 (LPA3): Signaling and Phosphorylation Sites
Source: Int J Mol Sci. 2024 Jun 12;25(12):6491. doi: 10.3390/ijms25126491 (PMC11203643; doi:10.3390/ijms25126491)
Supplement: Supplementary file 1 [file ijms-25-06491-s001.zip › Legends for the Videos.pdf]

Legends for the Videos

Video S1. LPA-induced LPA_3 internalization. The large dark zone inside the cell corresponds to the nucleous. After adding 1 μM LPA, fluorescence accumulates intracellularly (see the accumulation in the insert), forming "pearl necklace-like" structures (an asterisk indicates a large one; please see also the insert that allows observing vesicle interactions). The cell moves toward the right. The plasma membrane shows marked activity with changes in the form of the cell, lamellipodia movements, and bleb formation.

Video S2. PMA-induced LPA_3 internalization. An arrow indicates the accumulation of fluorescence in areas of the plasma membrane. After adding 1 μM PMA, marked changes in the cell shape occur, and fluorescence accumulates intracellularly, forming "pearl necklace-like" structures, which can be easily observed in the insert. The cells markedly contract, form membrane blebs, and move from the observation plane, disappearing and returning afterward. After returning to the observation plane, the intracellular fluorescence accumulation in vesicles can be observed (see insert).

Video S3. LPA_3-β-arrestin raw FRET in response to LPA. After adding 1 μM LPA, the red channel signal increases near the plasma membrane and in the cytoplasm. The arrows and asterisks indicate the accumulation and transit of FRET signals. The cells contract in response to LPA.

Video S4. LPA_3-β-arrestin colocalization in response to LPA. After adding 1 μM LPA, colocalization (white signal; please see the arrows and the insert) in cells expressing both proteins increases near the plasma membrane and within the cytoplasm. The cells contract.
