# Supplementary material for: Lysophosphatidic Acid Receptor 3 (LPA3): Signaling and Phosphorylation Sites
Source: Int J Mol Sci. 2024 Jun 12;25(12):6491. doi: 10.3390/ijms25126491 (PMC11203643; doi:10.3390/ijms25126491)

SUPPLEMENTARY MATERIALS

Supplementary Fig. S1. The LPA_1 receptor is functional and promotes heterologous and homologous desensitization. Panel A, calcium tracings of cells expressing LPA_1 receptors pre-incubated overnight without (black line) or with pertussis toxin (purple line); arrow indicates the addition of 1 μM LPA**. Panel B**, calcium tracings in response to 1 μM LPA of cells pre-incubated without any agent (black line) or with 1 μM LPA for 5 min and washed before re-challenging (LPA, black dotted line) or with 1 μM PMA (PMA, red line). **Panel C**, calcium increases in response to LPA of cells pre-incubated without any agent (None, open bar), with 1 μM LPA for 5 min and washed before re-challenging (LPA, dashed bar) or with 1 μM PMA (PMA, red dashed bar). The means are plotted, and vertical lines indicate the SEM of 4-5 experiments performed on different days and cell cultures. ***$p < 0.001$ vs. baseline.

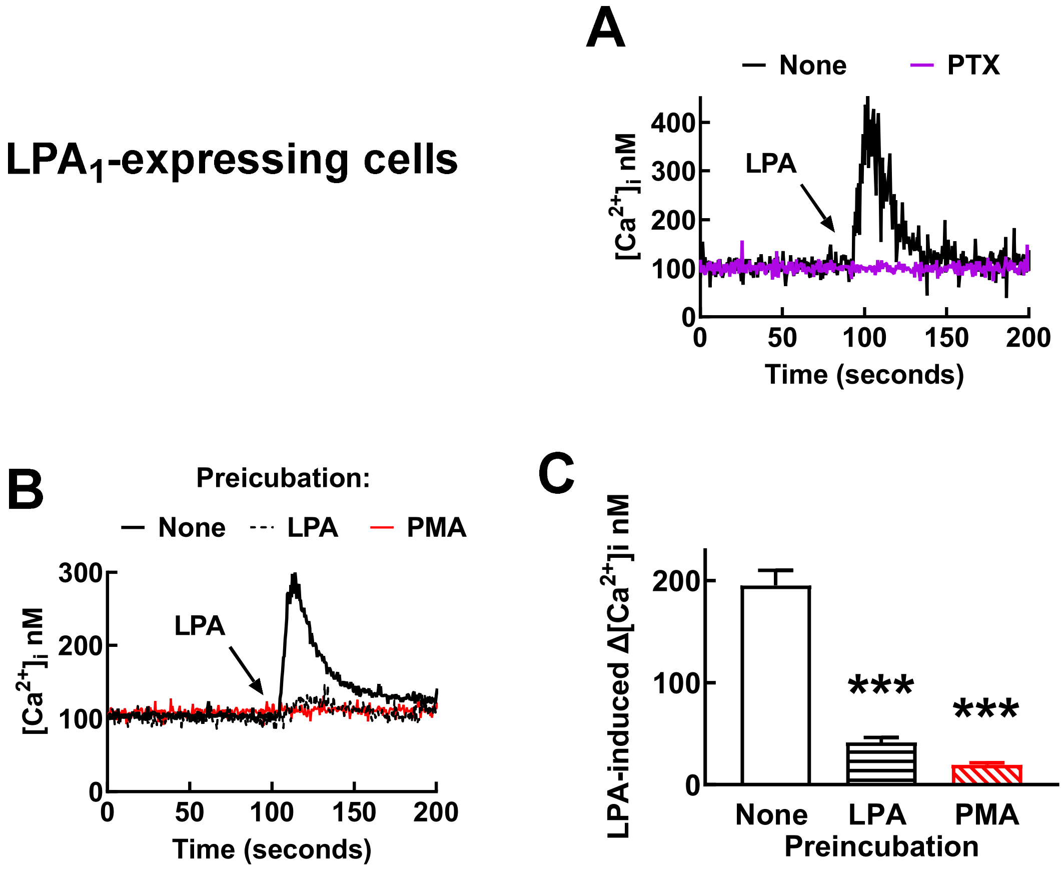

Supplementary Fig. S2. Effect of the Gα_q inhibitor, YM-254890, on LPA-induced increase in intracellular calcium concentrations in cells expressing LPA_1 or LPA_3 receptors. Representative calcium tracings of cells expressing LPA_1 (**panel A**) or LPA_3 (**panel B**) receptors were pretreated for 1 min without any agent (black lines) or with 1 μM YM-254890 (red lines) and challenged with 1 μM LPA (arrow). When no response was observed, thapsigargin (1 μM) was added to define the ability of the cells to increase the intracellular calcium concentration.

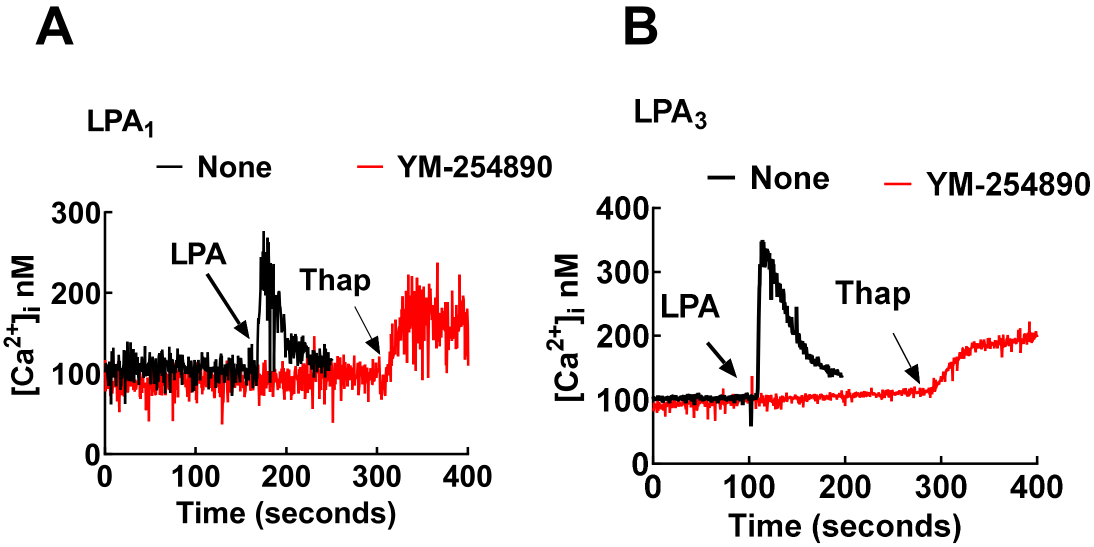

Supplementary Fig. S3. Absence of effect of overexpression of GRK2 and GRK5 on homologous LPA_3 desensitization. Cells expressing the LPA_3 receptor were not transfected (none) or transfected with plasmids for expression of GRK2 (**panels A-D**) or GRK 5 (**panels E-H**). Cells were challenged with 1 µM LPA (1st stimulus, representative tracings in **panels A and E**), washed, and challenged again with 1 µM LPA (2nd stimulus, representative tracings in **panels B and F**). LPA-induced increases in intracellular calcium are presented in **panels C and G** (the means are plotted, and vertical lines indicate the SEM of 5-7 experiments performed using different cell cultures). In **panels D** (GRK2, Mr ≈ 80 kDa) **and H** (GRK5, Mr ≈ 65 kDa) representative Western blots, using GRK2- or GRK5-speficific primary antibodies, of cell lysates are presented.

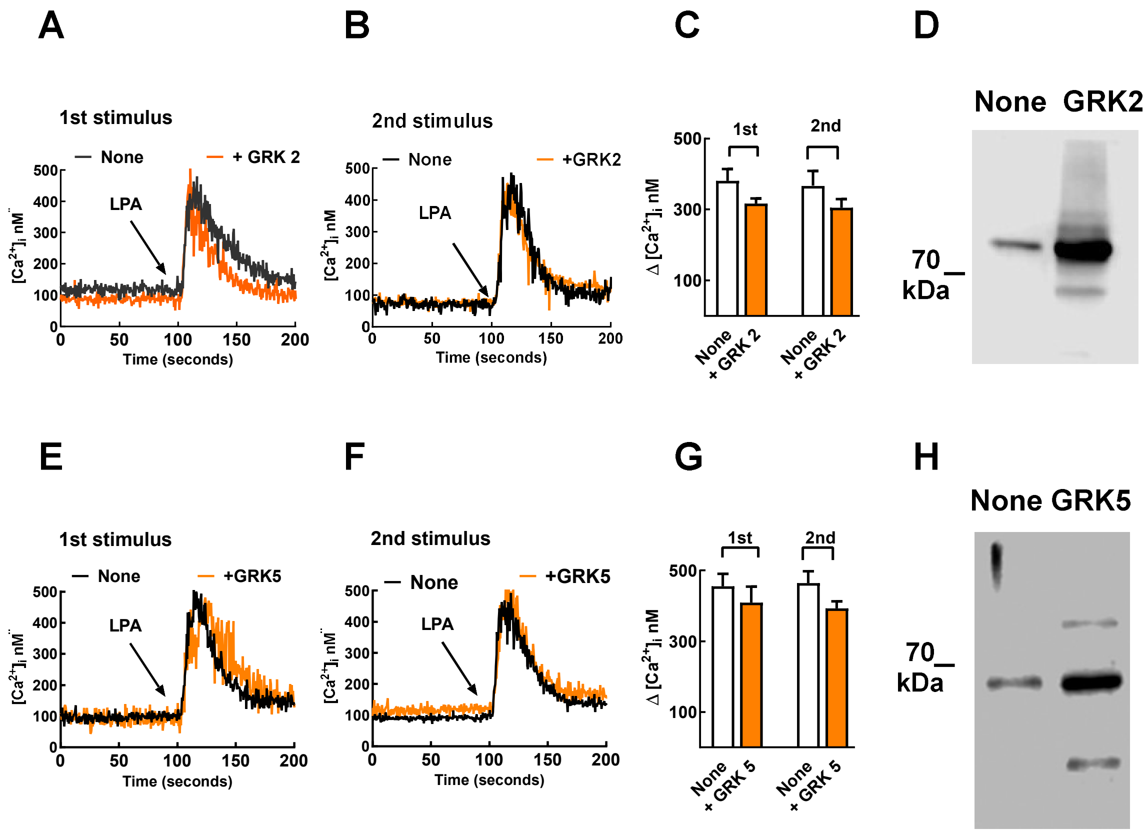

Supplementary Fig. S4. Effect of pertussis toxin on LPA_1-induced ERK 1/2 phosphorylation. Cells were incubated overnight with (PTX, purple line) or without (None, black line) pertussis toxin, and cells were challenged with 1 μM LPA for the times indicated. Phospho-ERK (pERK) and total ERK (ERK) were determined by Western blotting. The means are plotted, and vertical lines indicate the SEM of 4-5 experiments performed on different days using different cell cultures. ***$p < 0.001$, and **$p < 0.005$ vs. baseline value. Baseline ERK was significantly diminished by pertussis toxin treatment, $p < 0.001$. Representative Western blots are presented above the figure.

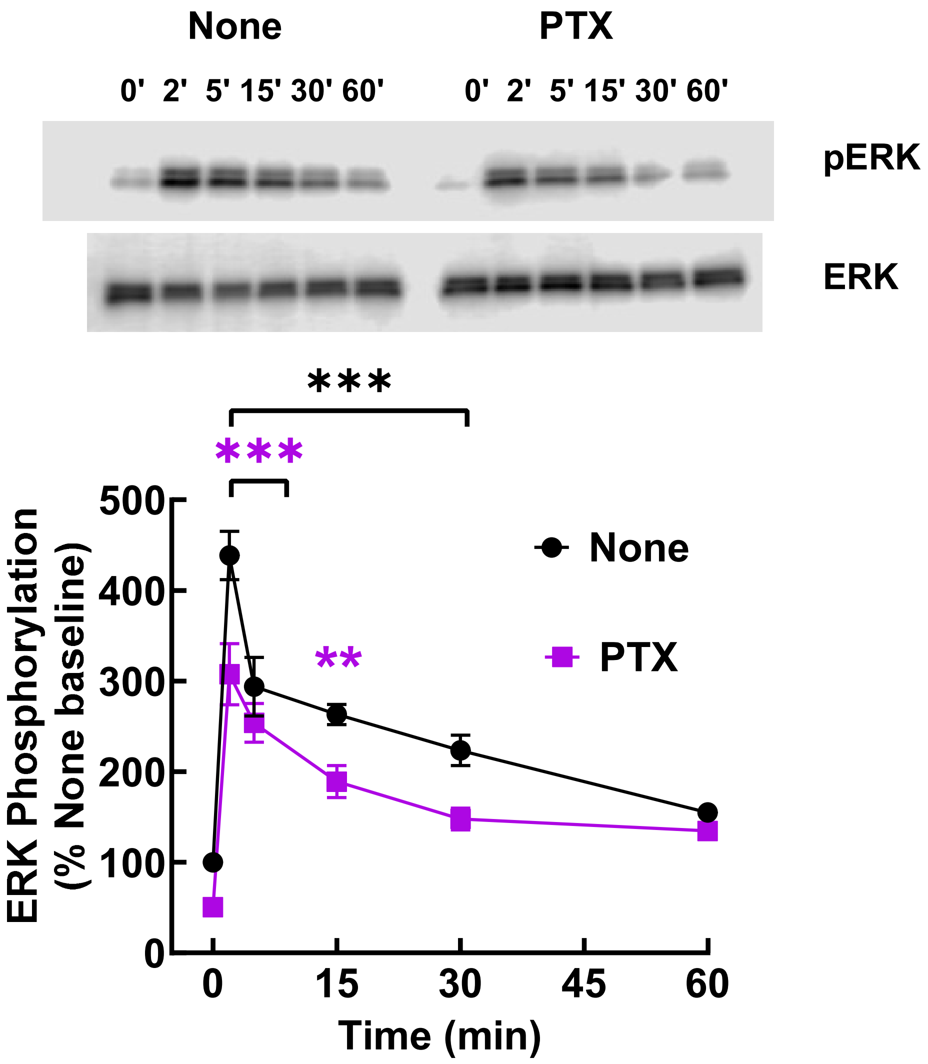

Supplementary Fig. S5. Effect of Pitstop 2 on LPA-induced ERK phosphorylation. LPA_3-expressing cells were pre-incubated without (None) or with 10 µM Pitstop 2 for 15 min and then treated with 1 µM LPA for the times indicated. The means are plotted, and vertical lines indicate the SEM of 4-5 experiments performed on different days using distinct cell cultures. ***$p < 0.001$, **$p < 0.005$ vs. baseline value. The clathrin inhibitor, Pitstop 2, decreased baseline ERK 1/2 phosphorylation, $p < 0.001$. Representative Western blots are presented above the figure.

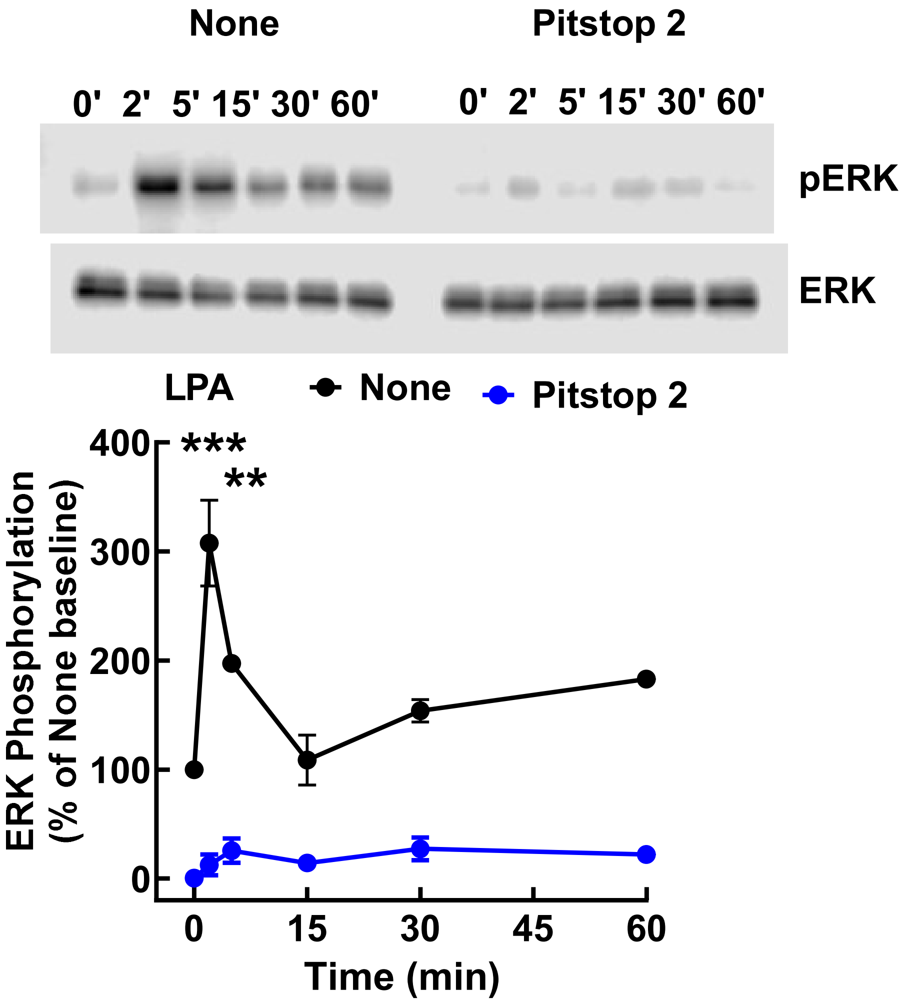

Supplementary Fig. S6. Effect of Pitstop 2 on PMA-induced ERK phosphorylation. LPA_3-expressing cells were pre-incubated without (None) or with 10 μM Pitstop 2 for 15 min and then treated with 1 μM PMA for the times indicated. The means are plotted, and vertical lines indicate the SEM of 4-5 experiments performed on different days using distinct cell cultures. ***p < 0.001 vs. baseline value. The clathrin inhibitor, Pitstop 2, decreased baseline ERK 1/2 phosphorylation, p < 0.001. Representative Western blots are presented above the figure.

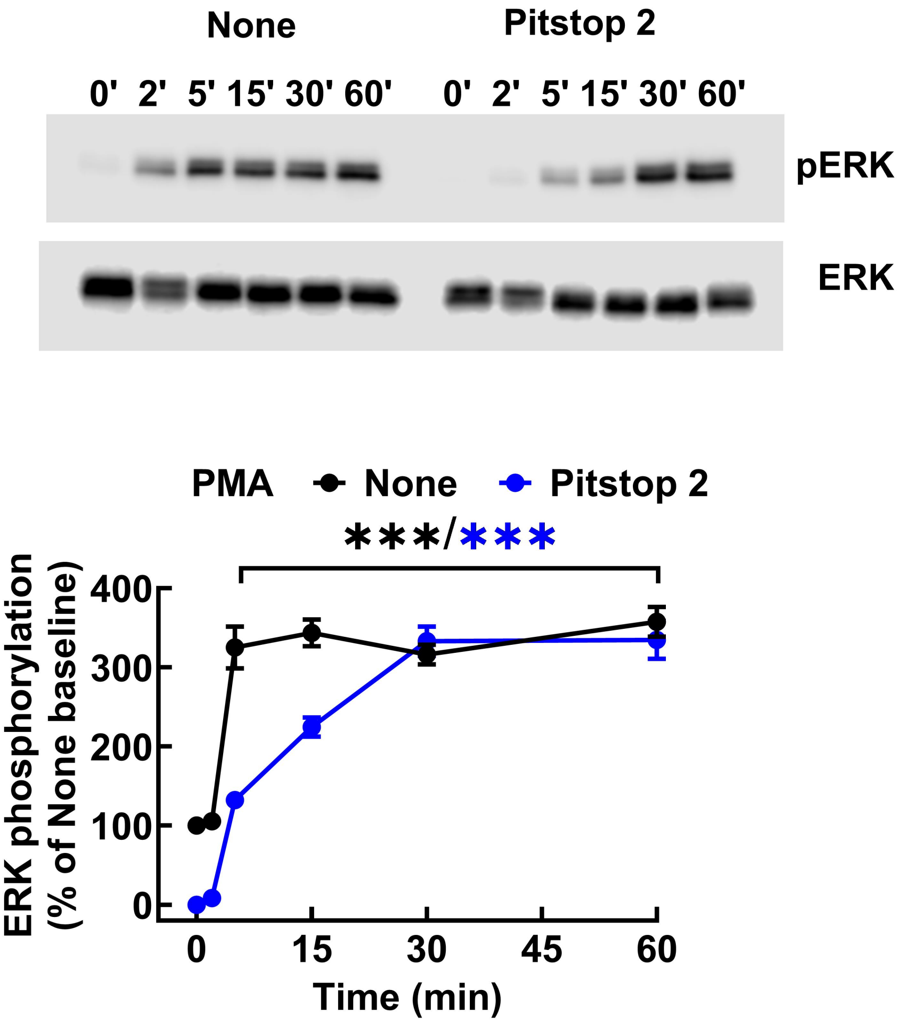

Supplementary Fig. S7. Effect of Pitstop 2 LPA-, PMA- and EGF-mediated ERK phosphorylation. LPA_3-expressing cells were pre-incubated without (None) or with 10 μM Pitstop 2 for 15 min and then treated with 1 μM LPA, 1 μM PMA, or 100 ng/ml EGF for 15 min. *** $p < 0.001$. Representative Western blots are presented above the figure.

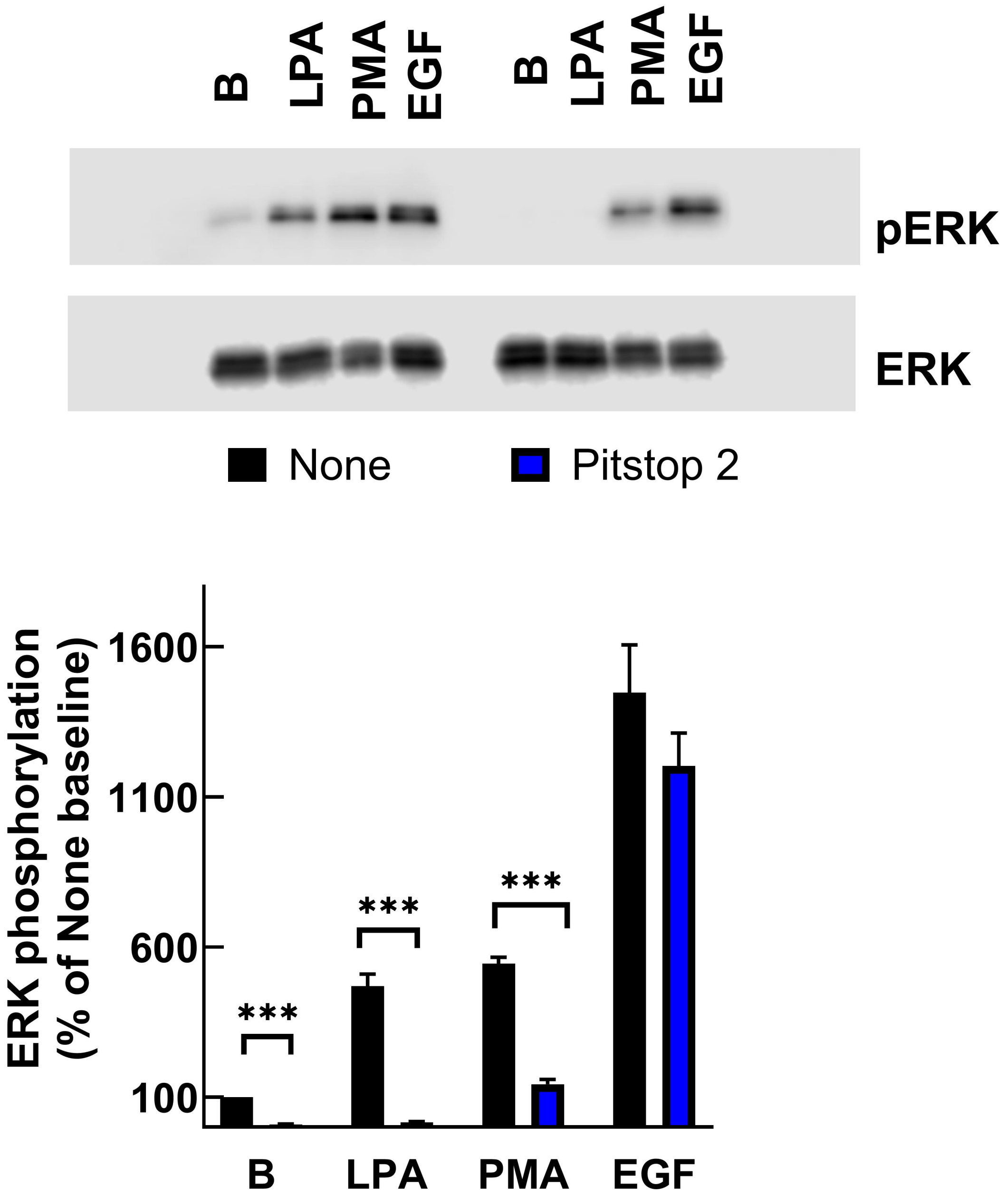

Supplementary Fig. S8. Effect of the Gα_q inhibitor, YM-254890, on LPA-induced ERK phosphorylation in LPA_1 and LPA_3-expressing cells. LPA_3- (**panel A**) or LPA_1- (**panel B**) expressing cells were pre-incubated for 1 min without (None, black lines and symbols) or 1 μM YM-254890 (red lines and symbols) and then incubated with 1 μM LPA for the times indicated. ***p < 0.001, and *p≤0.05 vs. their respective baseline values. YM-254890, decreased baseline ERK 1/2 phosphorylation , p < 0.001. Representative Western blots are presented above the figures.

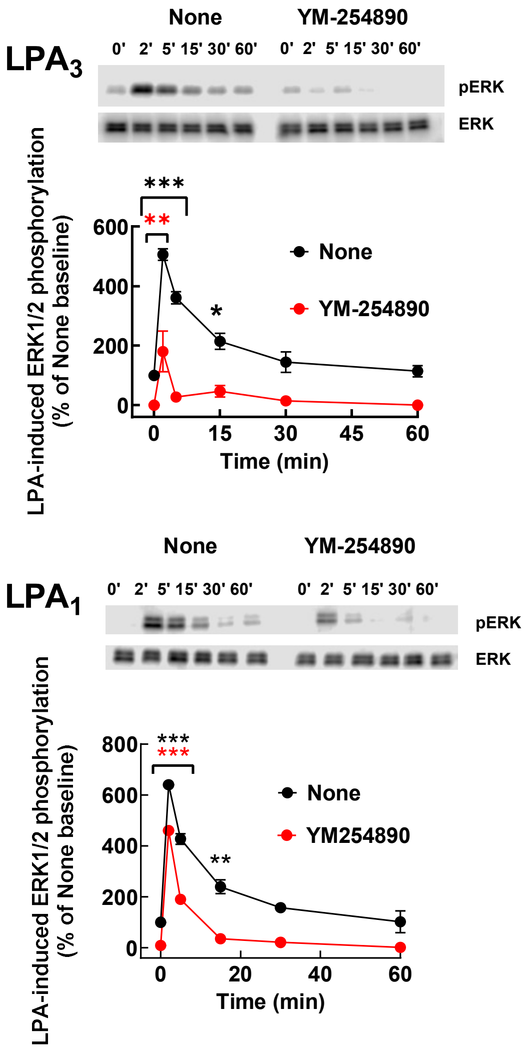

Supplementary Fig. S9. Receptor internalization in cells in which receptor expression was induced by a reduced (1 ng/ml) concentration of doxycycline. Cells were incubated for the times indicated in the presence of 1 μM LPA or 1 μM PMA. Internalization is presented as the percentage of baseline intracellular fluorescence. The mean is plotted, and vertical lines indicate the SEM of 14 images obtained from each of 5 experiments performed on different days. At the right side of the graph, representative images without or with the nuclei satained with DAPI are presented. Scale bars 10 μm. ***p < 0.001 vs baseline (0').

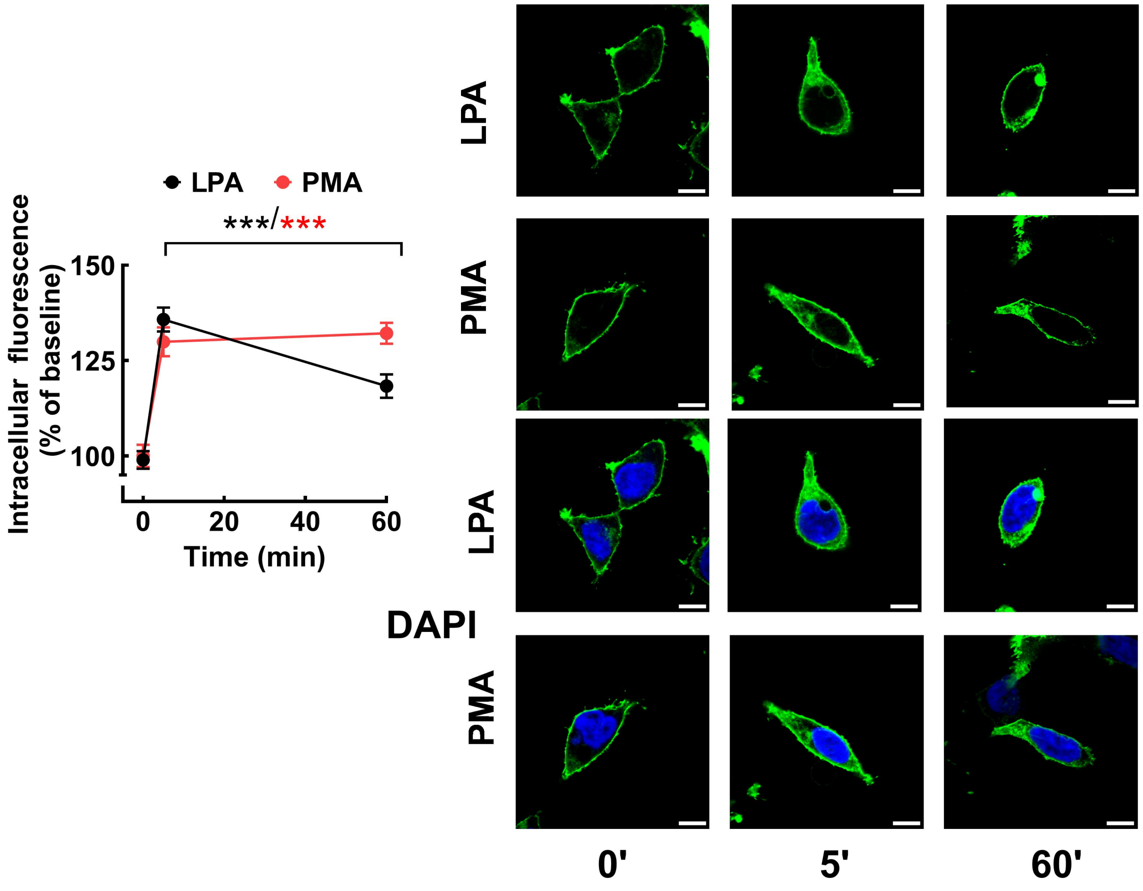

Supplementary Fig. S10. Effect of Pitstop 2 on LPA-induced LPA_3 internalization. Cells expressing the LPA_3 receptor were pre-incubated without any agent (None, black line and symbols) or with Pitstop 2 (red line and symbols) for 15 min and then stimulated with 1 μM LPA. Pitstop 2 decreased baseline internal fluorescence, ***$p < 0.001$. Baseline internal fluoresce was decreased by pre-incubation with Pitstop 2 ($p < 0.001$). When compared to their baseline, LPA increased internalization in the presence of Pitstop 2 (blue asterisks) ***$p < 0.001$, **$p < 0.005$, and *$p < 0.05$ vs. baseline value. Representative images are presented above the figure. Scale bars 10 μm.

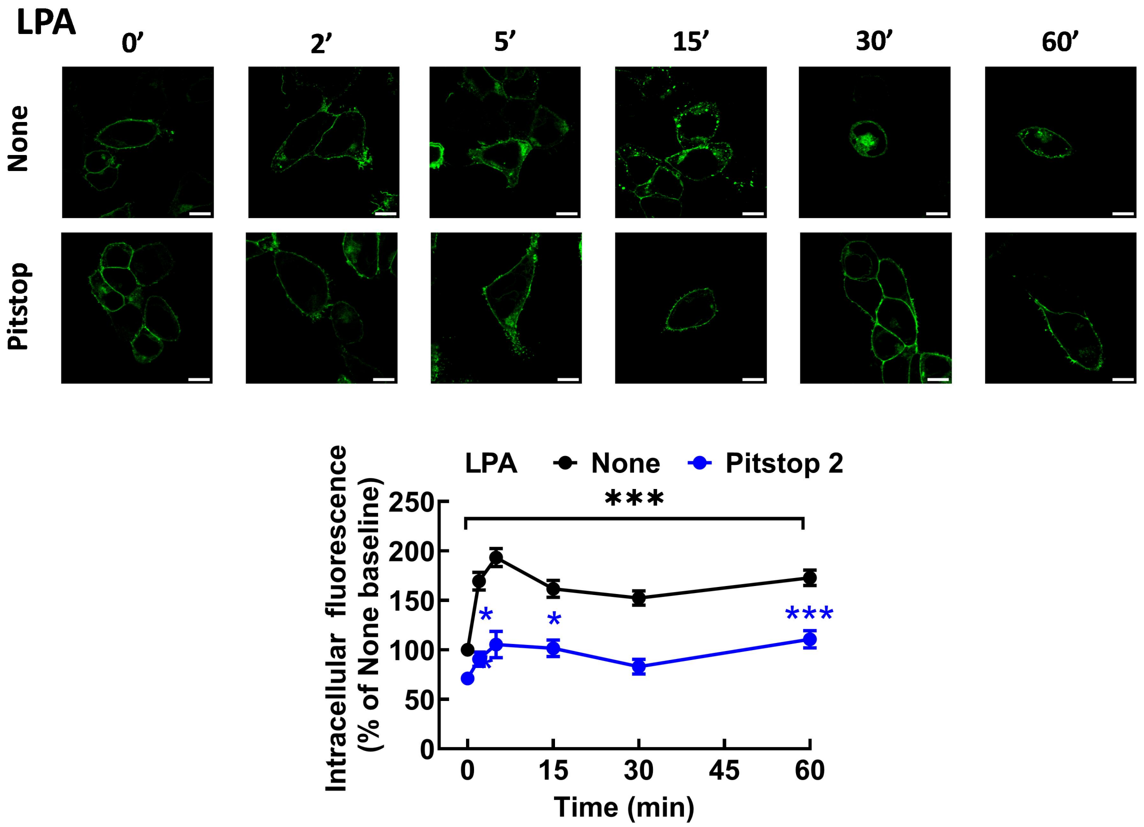

Supplementary Fig. S11. Effect of Pitstop 2 on PMA-induced LPA_3 internalization. Cells expressing the LPA_3 receptor were pre-incubated without any agent (None, black line and symbols) or with Pitstop 2 (red line and symbols) for 15 min and then stimulated with 1 µM PMA. ***p < 0.001 vs. none baseline, (blue asterisks) ***p < 0.001 vs. baseline (Pitstop 2). Baseline internal fluoresce was decreased by pre-incubation with Pitstop 2 (p < 0.001). Representative images are presented above the figure. Scale bars 10 µm.

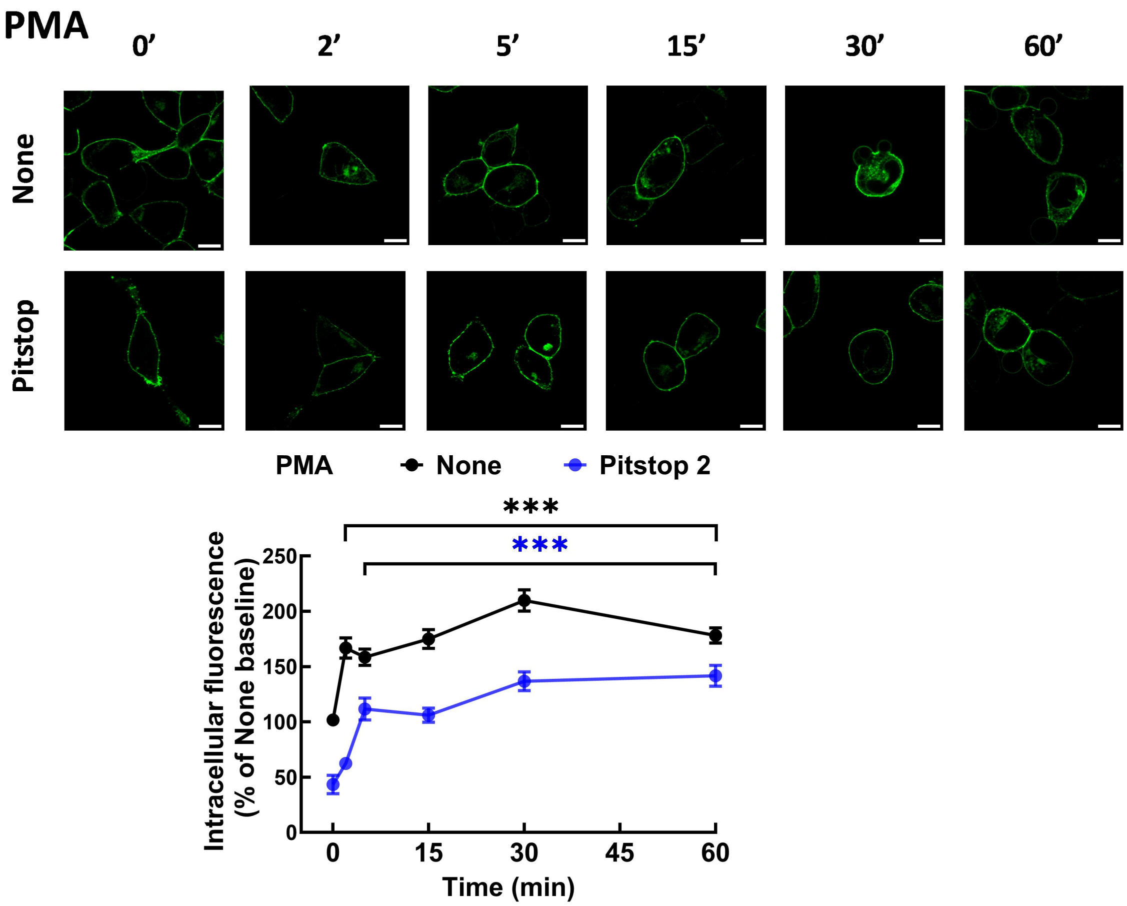

Supplementary Fig. S12. Representative images of the time-course of the effect of LPA on eGFP-tagged LPA_3 receptor-mCherry-tagged β-arrestin 2 interaction (FRET). Cells were incubated for the times indicated in the presence of 1 μM LPA. The following images are presented: LPA_3-eGFP fluorescence (eGFP was excited and its fluorescence was recorded; first column), β-arrestin-mCherry fluorescence (mCherry was excited and its fluorescence was recorded; second column), FRET (raw FRET) (eGFP was excited, the laser to excite mCherry remained off, and mCherry fluorescence was recorded; third column) and "FRET index" image (images processed with the ImageJ "FRET and Colocalization Analyzer", fourth column). Scale bars: 10 μm.

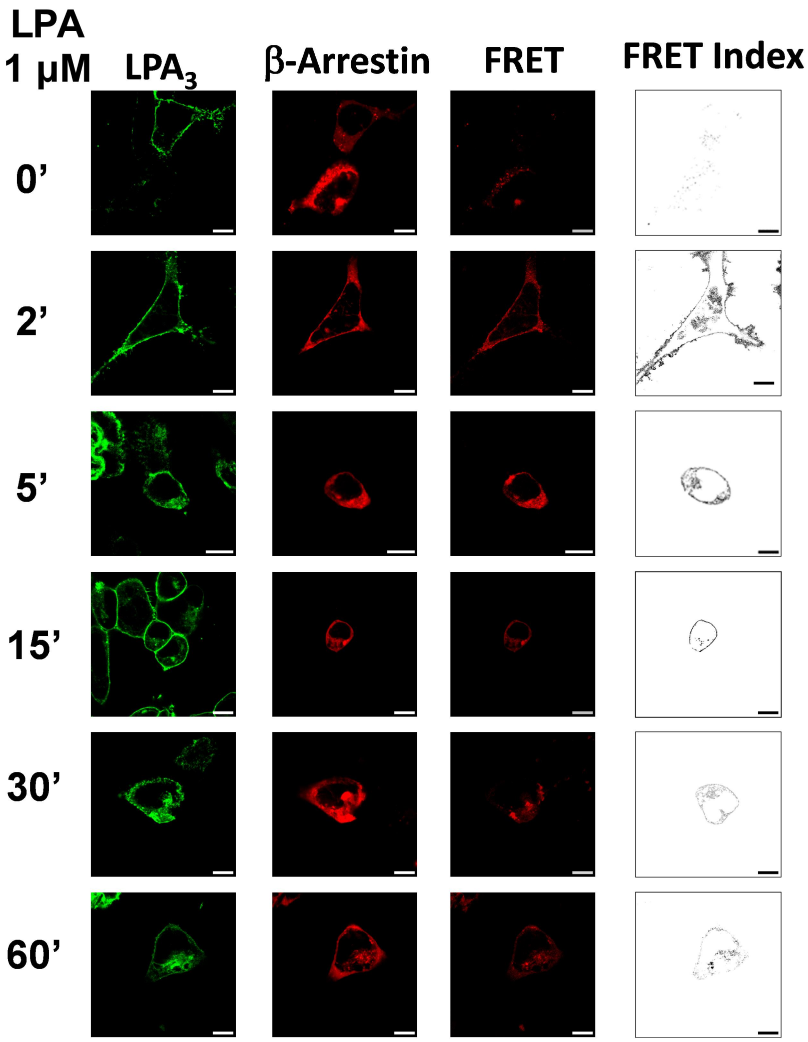

Supplementary Fig. S13. Representative images of the time-course of the effect of PMA on eGFP-tagged LPA_3 receptor-mCherry-tagged β-arrestin 2 interaction (FRET). Cells were incubated for the times indicated in the presence of 1 µM PMA. The following images are presented: LPA_3-eGFP fluorescence (eGFP was excited and its fluorescence was recorded; first column), β-arrestin-mCherry fluorescence (mCherry was excited and its fluorescence was recorded; second column), FRET (raw FRET) (eGFP was excited, the laser to excite mCherry remained off, and mCherry fluorescence was recorded; third column) and "FRET index" image (images processed with the ImageJ "FRET and Colocalization Analyzer", fourth column). Scale bars: 10 µm.

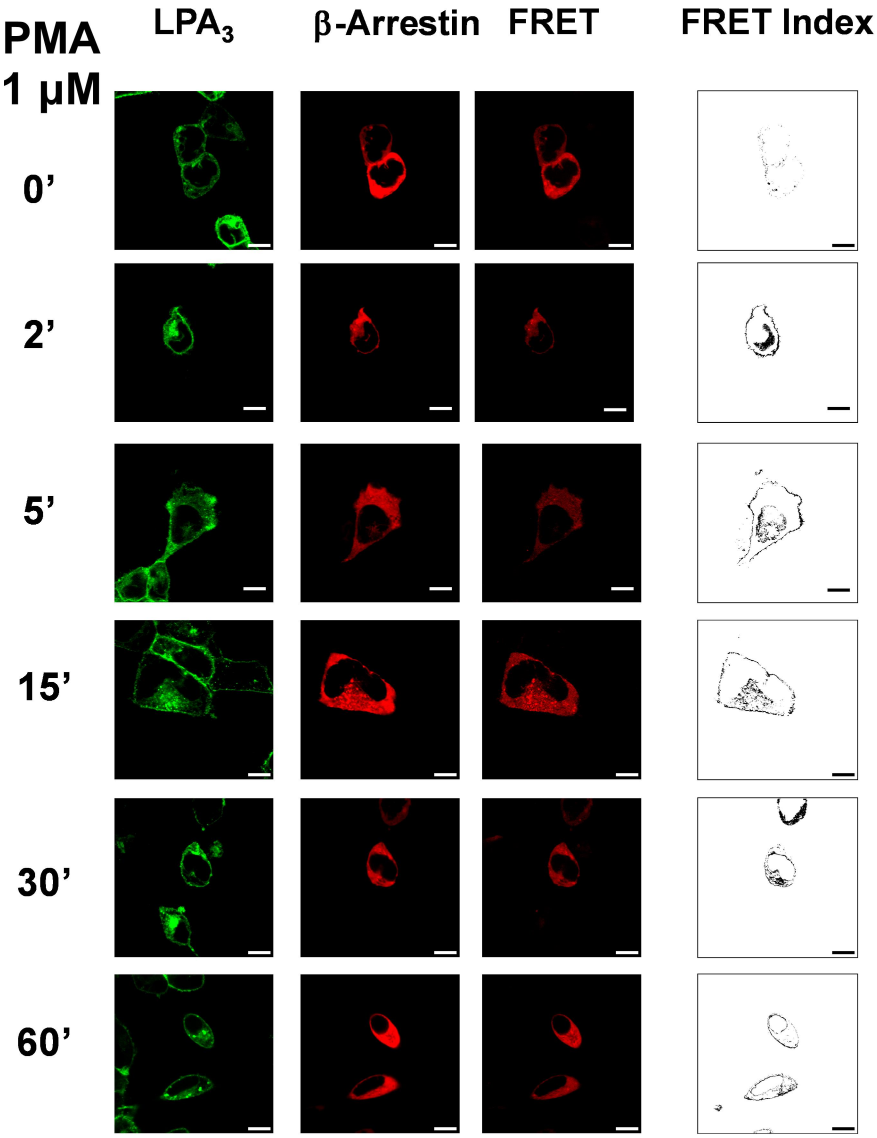

Supplementary Fig. S14. Representative image of the time course of LPA-induced LPA_3 receptor phosphorylation. The image shows a whole membrane autoradiograph of the phosphorylation pattern of the LPA_3 receptor when activated with LPA. The arrow indicates the LPA_3 receptor construct.

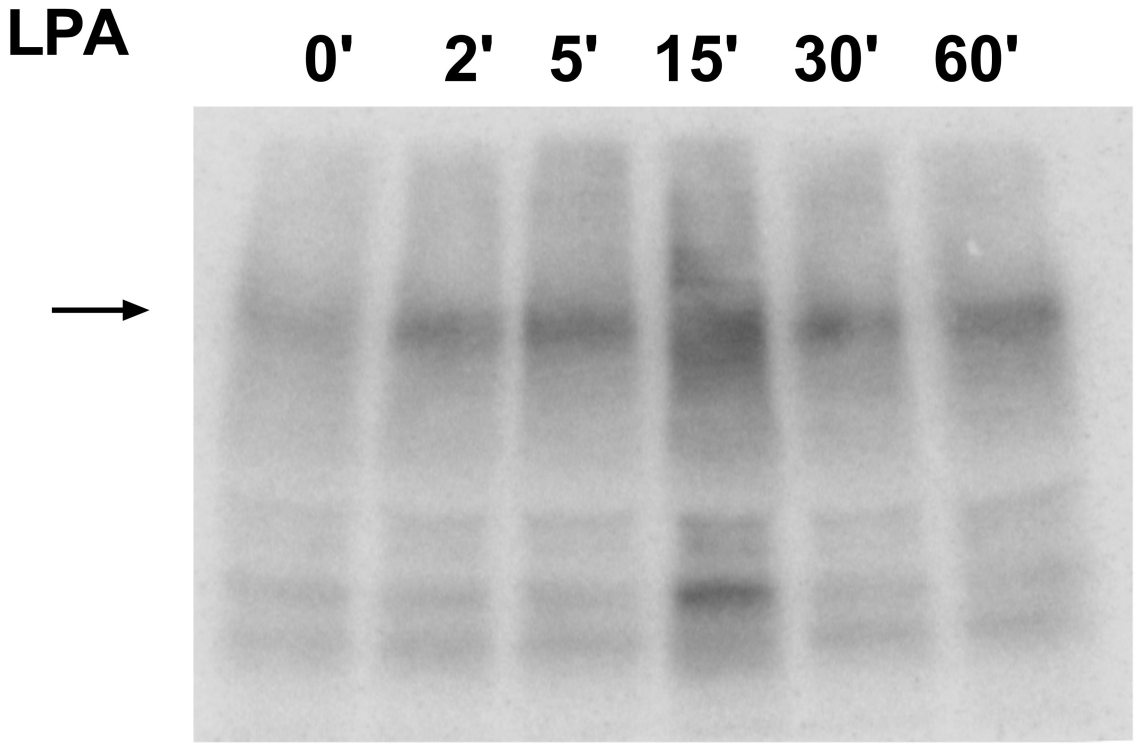

Supplementary Fig. S15. Representative LPA_3 immunopurification samples used for mass spectrometry analysis. The image on the left shows an SDS-polyacrylamide gel electrophoresis stained with Coomassie blue; the bands detected at Mr $\approx$ 70 kD, corresponding to the LPA_3 construct (arrow), were cut and sent to mass spectrometry analysis. Identification of the LPA_3 receptor-eGFP (arrow) was performed by Western blot (**right panel**). Identification of the IgG heavy (IgG HC) and light (IgG LC) chains are indicated.

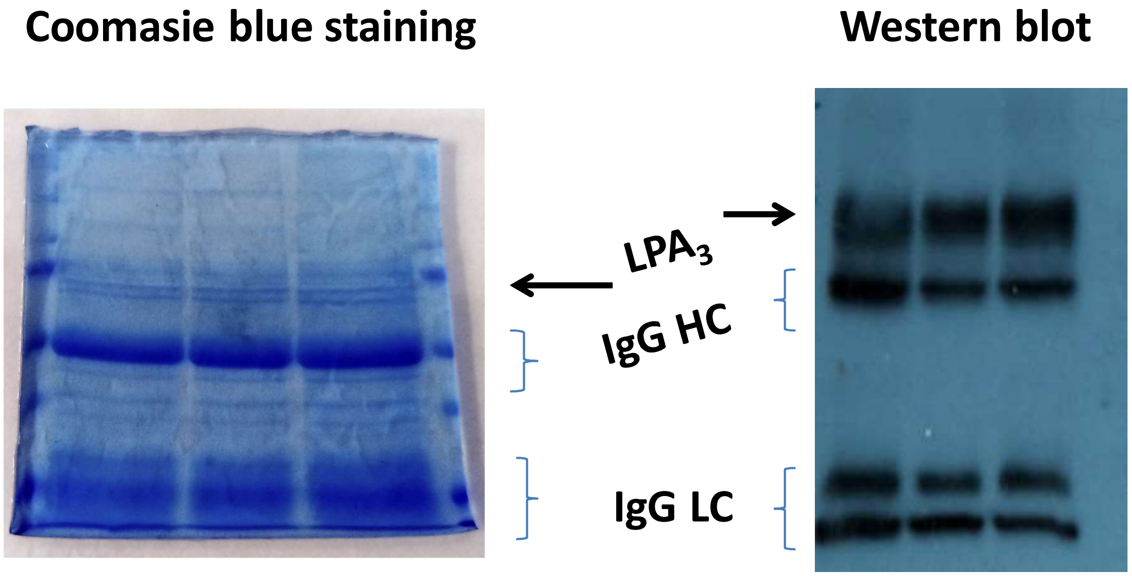

Supplementary Fig. S16. LPA- and PMA-induced wild type and Mutant LPA_3 receptor phosphorylation. Cells expressing the wild-type or mutant receptors were challenged with 1 µM LPA or 1 µM PMA for 10 min.. Receptor phosphorylation was determined as in Fig. 5 and it is expressed as the percentage of the wild-type baseline value. The means are plotted, and vertical lines indicate the SEM of 4 experiments performed. ***p < 0.001, **p < 0.01, ΔΔ p < 0.01 vs. wild-type baseline value. Representative autoradiographs (^{32}P) and Western blots (WB) are presented above the graph.

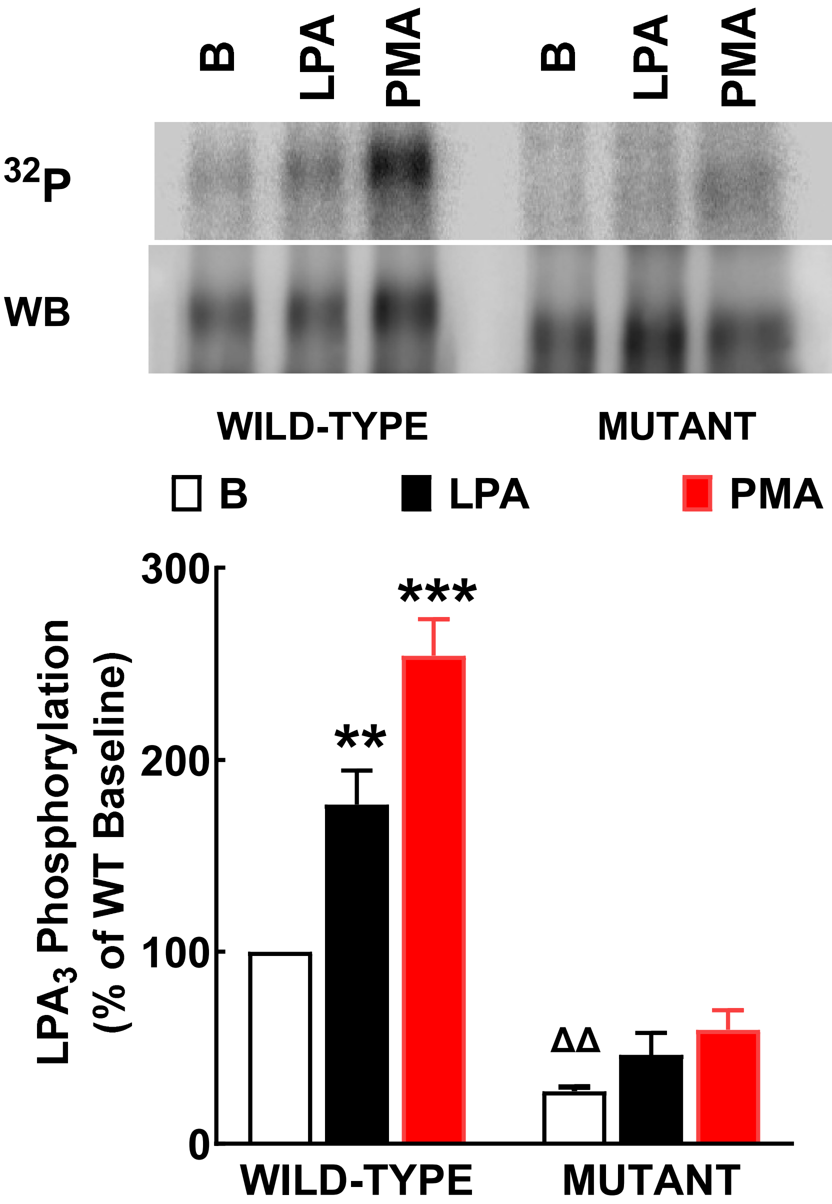

Supplementary Fig. S17. Expression of wild-type and mutant LPA_3 receptors. Representative images of cells expressing the wild-type (panel A) and the mutant receptors (panel B). Bars, 10 µm. Panel C shows the fluorescence intracellular/total ratio detected in cells expressing wild-type and mutant receptors. The means are plotted, and vertical lines indicate the SEM of 5 experiments in which 10 cells were analyzed. ***$p < 0.001$, Student t-test. Western blot detection of immunopurified wild-type and mutant LPA_3 receptors. Data were normalized to the abundance detected in the wild-type samples. The means are plotted, and vertical lines indicate the SEM of 4 with samples in triplicate, including variation in the control samples. A representative Western blot is shown above the figure.

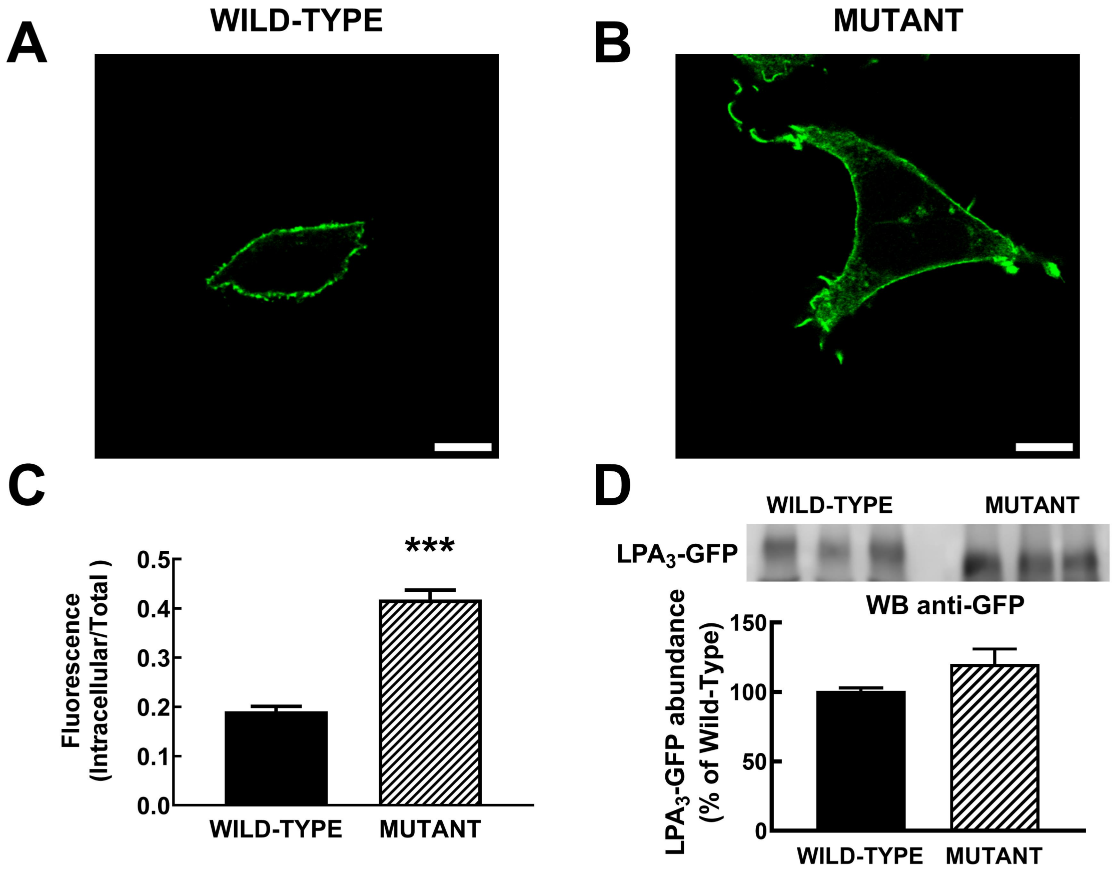

Supplement: Supplementary file 1 [file ijms-25-06491-s001.zip › Supplementary Materials Figures S1-S17.pdf]
