# Supplementary material for: Lysophosphatidic Acid Receptor 3 (LPA3): Signaling and Phosphorylation Sites
Source: Int J Mol Sci. 2024 Jun 12;25(12):6491. doi: 10.3390/ijms25126491 (PMC11203643; doi:10.3390/ijms25126491)

SUPPLEMENTARY MASS SPECTROMETRY SPECTRA.

Mass spectrometry analysis was performed at the Taplin Mass Spectrometry Facility (Harvard Medical School. Cambridge, MA, USA). Five independent experiments, including all conditions, were performed and analyzed. The authors thank Ross Tomaino, Associate Director for his generous help with the mass spectrometry analysis and obtention of the spectra required.

LPA3-Basal

25 jun 2020

Predicted Fragmentation Pattern

+1

Seq	#	b: Δ Error	b	y	y: Δ Error	+1
S	1	---	88.039	---	---	17
Q	2	---	216.098	1958.981	---	16
E	3	-141.766	**345.140**	1830.922	---	15
N	4	238.289	**459.183**	1701.880	---	14
P	5	---	556.236	1587.837	---	13
E	6	---	685.279	1490.784	---	12
R	7	---	841.380	1361.741	---	11
R	8	156.534	**997.481**	1205.640	---	10
P	9	---	1094.534	1049.539	---	9
S#	10	---	1261.532	952.486	---	8
R	11	---	1417.633	**785.488**	226.857	7
I	12	---	1530.717	629.387	---	6
P	13	---	1627.770	**516.303**	109.584	5
S	14	---	1714.802	419.250	---	4
T	15	---	1815.850	332.218	---	3
V	16	---	1914.918	231.170	---	2
L	17	---	---	132.102	---	1

+2

Seq	#	b: Δ Error	b	y	y: Δ Error	+1
S	1	---	44.523	---	---	17
Q	2	---	108.553	979.994	---	16
E	3	---	173.074	915.965	---	15
N	4	---	230.095	**851.443**	215.133	14
P	5	---	278.622	**794.422**	226.552	13
E	6	---	343.143	**745.896**	103.392	12
R	7	---	421.194	681.374	---	11
R	8	-156.365	**499.244**	603.324	---	10
P	9	-679.489	**547.771**	525.273	---	9
S#	10	-192.308	**631.270**	476.747	---	8
R	11	---	709.320	393.248	---	7
I	12	---	765.862	**315.197**	-229.656	6
P	13	---	814.389	258.655	---	5
S	14	---	857.905	210.129	---	4
T	15	14.327	**908.429**	166.613	---	3
V	16	---	957.963	116.089	---	2
L	17	---	---	66.555	---	1

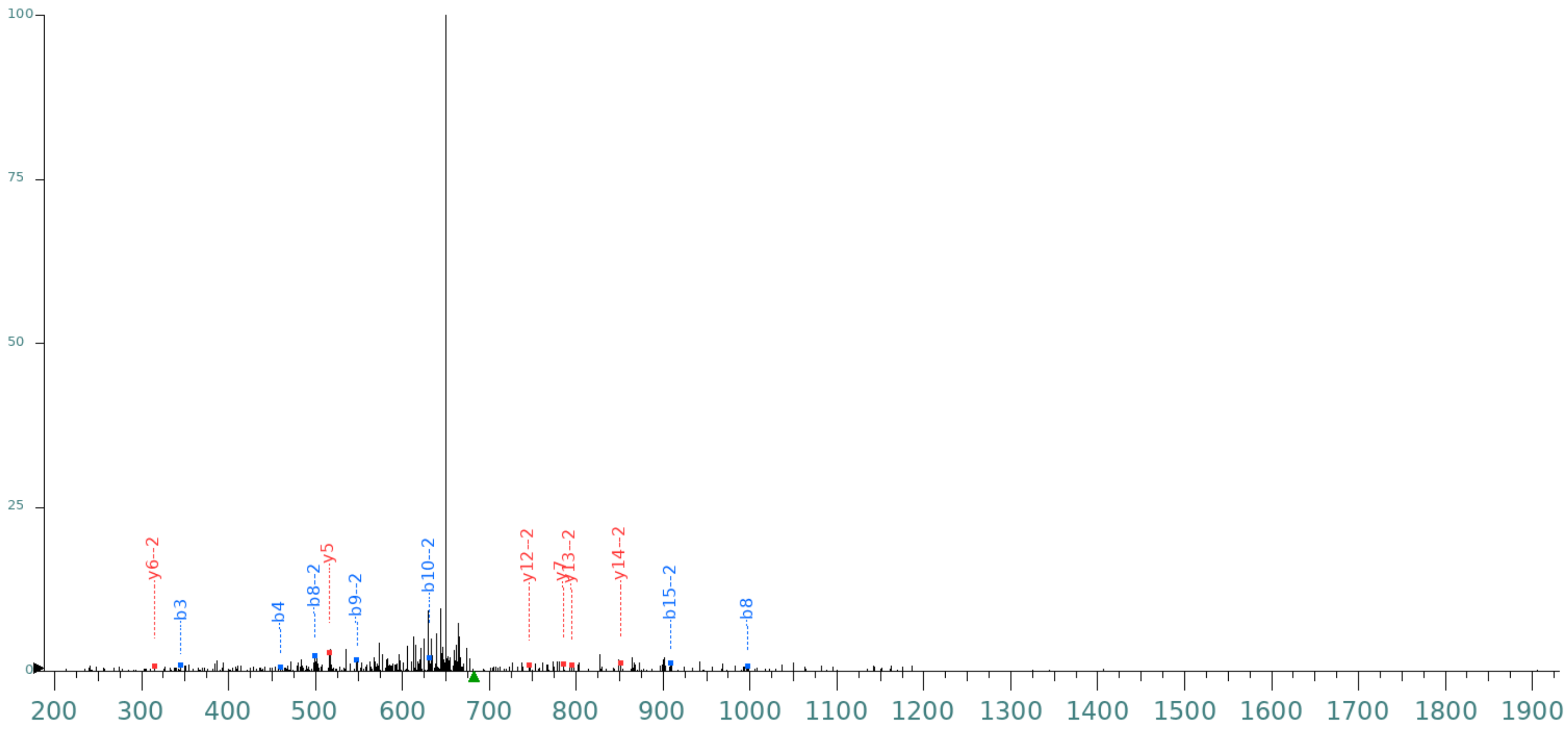

Predicted Fragmentation Pattern

Seq	#	b: Δ Error	b	y	y: Δ Error	+
V	1	---	100.076	---	---	1
L	2	---	213.160	1221.562	---	1
S#	3	671.456	**380.158**	1108.478	---	1
P	4	-962.476	**477.211**	**941.480**	-9.725	
H	5	-98.698	**614.270**	**844.427**	253.312	
T	6	---	715.317	**707.368**	275.146	
S	7	---	802.349	**606.321**	236.794	
G	8	---	859.371	**519.289**	380.156	
S	9	---	946.403	**462.267**	540.952	
I	10	---	1059.487	375.235	---	
S	11	---	1146.519	**262.151**	128.346	
R	12	---	---	175.119	---	

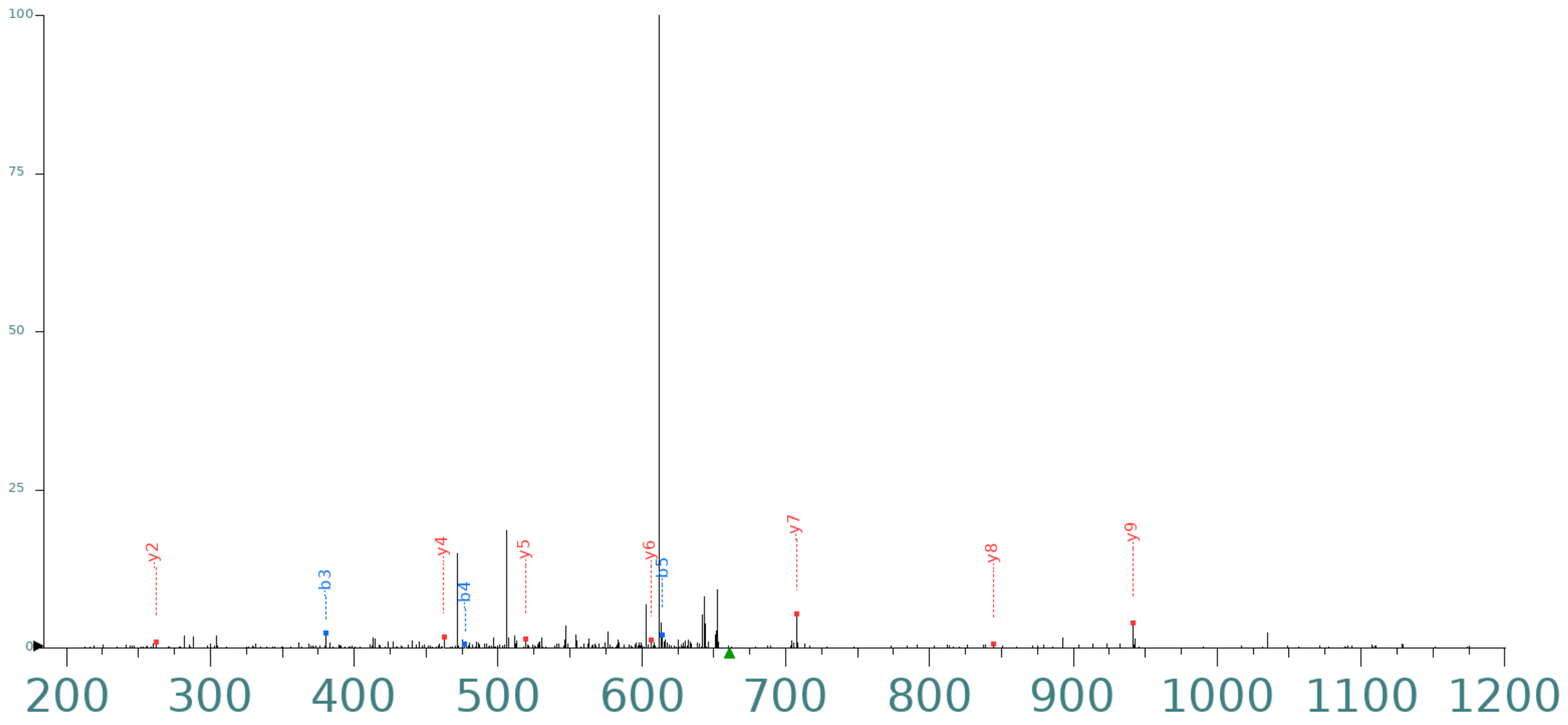

Predicted Fragmentation Pattern

Seq	#	b: Δ Error	b	y	y: Δ Error
R	1	---	157.108	---	---
I	2	607.601	270.192	709.353	---
P	3	1089.548	367.245	596.269	-68.615
S#	4	-723.781	534.244	499.216	---
T	5	370.817	635.291	332.218	---
V	6	234.926	734.360	231.170	443.101
L	7	---	---	132.102	80.909

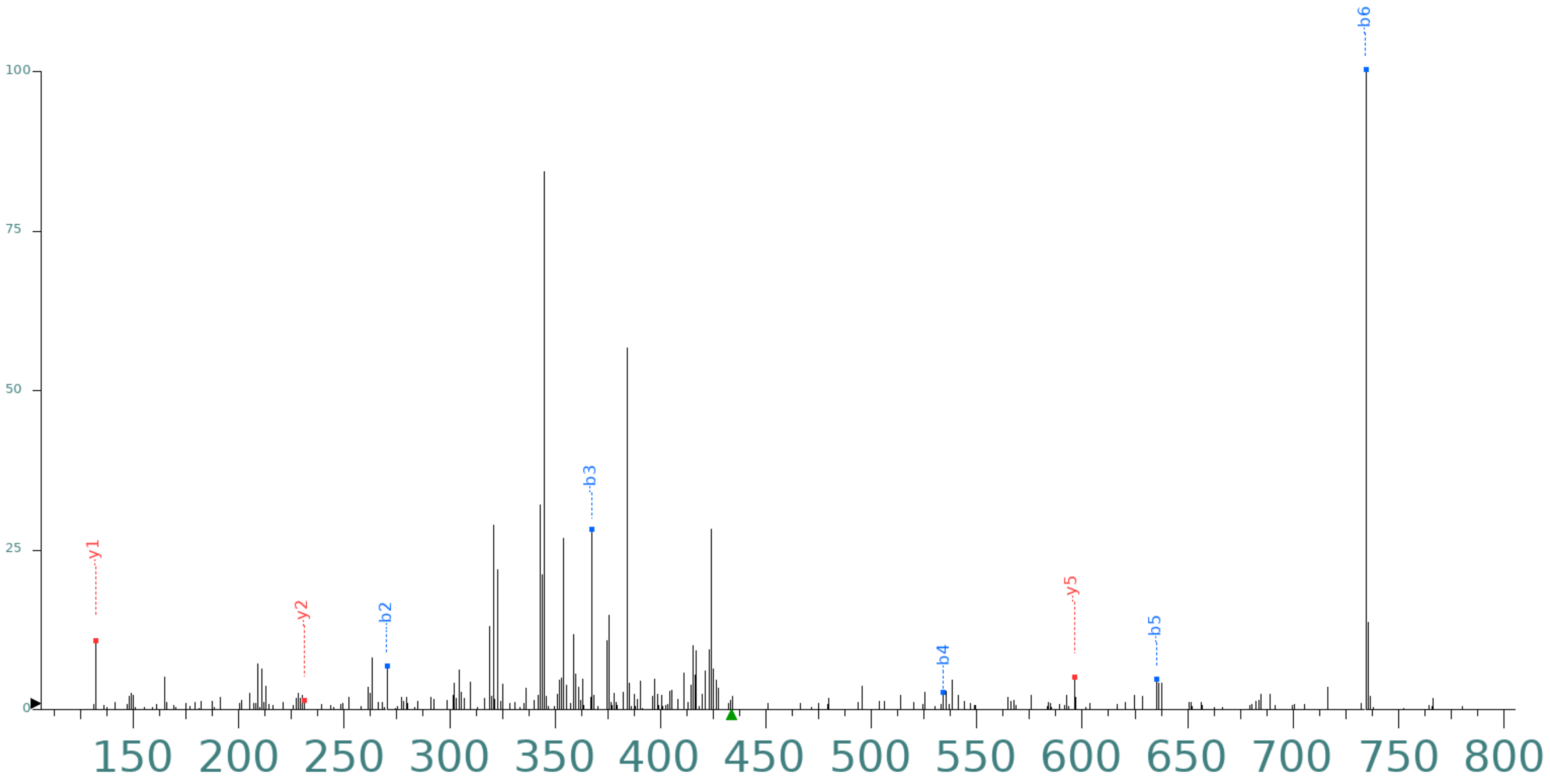

LPA3-LPA

25 jun 2020

Predicted Fragmentation Pattern

Seq	#	b: Δ Error	b	y	y: Δ Error	+1
R	1	---	157.108	---	---	7
I	2	356.760	270.192	709.353	---	6
P	3	807.050	367.245	596.269	166.589	5
S#	4	787.253	534.244	499.216	331.368	4
T	5	112.694	635.291	332.218	---	3
V	6	-65.478	734.360	231.170	1141.137	2
L	7	---	---	132.102	557.075	1

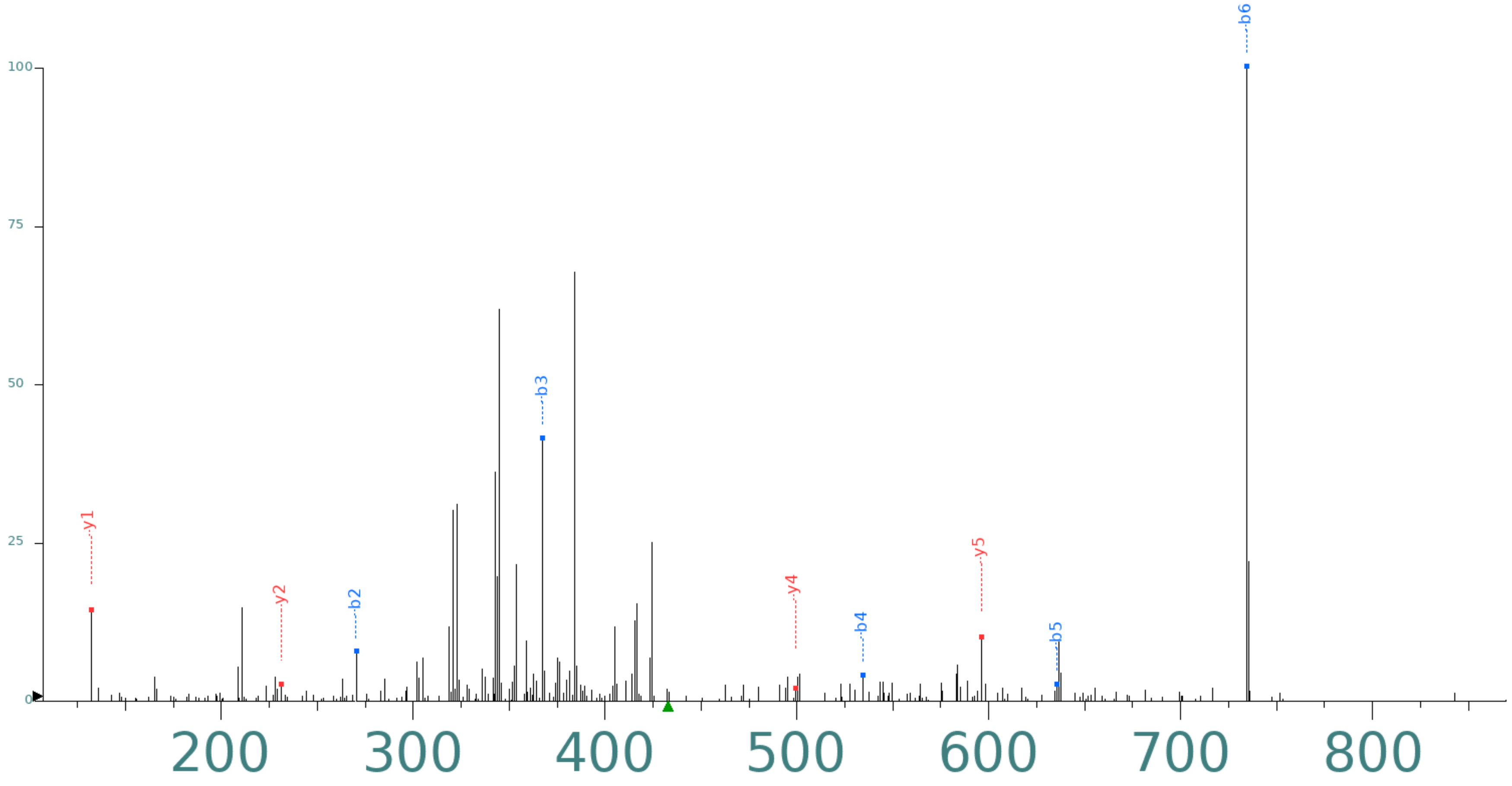

Predicted Fragmentation Pattern

Seq	#	b: Δ Error	b	y	y: Δ Error	+1
R	1	---	157.108	---	---	10
P	2	58.040	**254.161**	**1049.539**	66.151	9
S	3	134.228	**341.193**	**952.486**	-11.594	8
R	4	---	497.294	865.454	---	7
I	5	317.889	**610.378**	709.353	---	6
P	6	---	707.431	**596.269**	318.523	5
S	7	---	794.463	**499.216**	370.831	4
T#	8	---	975.477	**412.184**	-487.417	3
V	9	---	1074.546	231.170	---	2
L	10	---	---	132.102	---	1

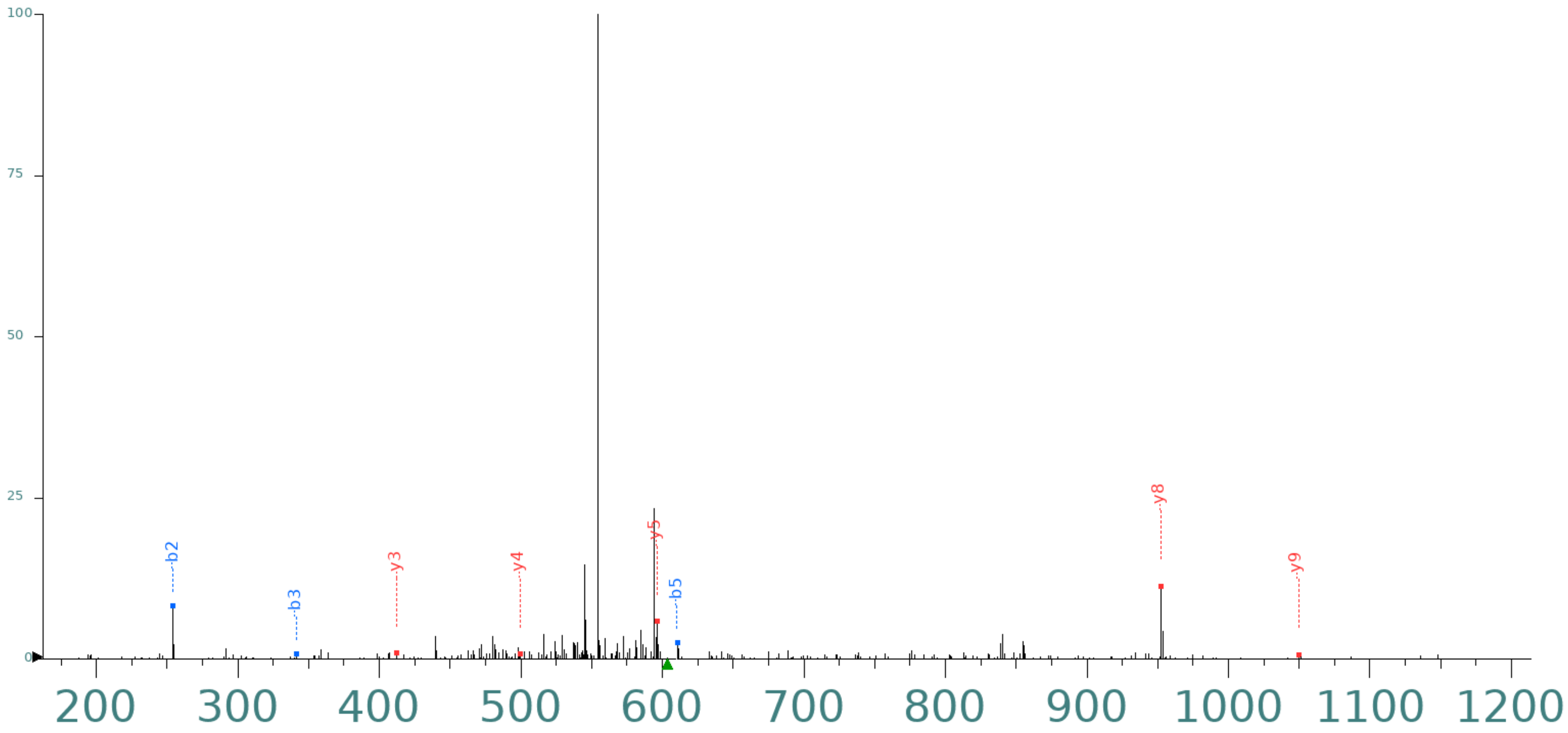

Predicted Fragmentation Pattern

Seq	#	b: Δ Error	b	y	y: Δ Error	+1
V	1	---	100.076	---	---	12
L	2	---	213.160	1221.562	---	11
S#	3	390.628	**380.158**	1108.478	---	10
P	4	225.564	**477.211**	**941.480**	15.299	9
H	5	---	614.270	844.427	---	8
T	6	349.852	**715.317**	**707.368**	211.327	7
S	7	---	802.349	**606.321**	-75.922	6
G	8	---	859.371	519.289	---	5
S	9	---	946.403	**462.267**	678.100	4
I	10	---	1059.487	375.235	---	3
S	11	---	1146.519	262.151	---	2
R	12	---	---	175.119	---	1

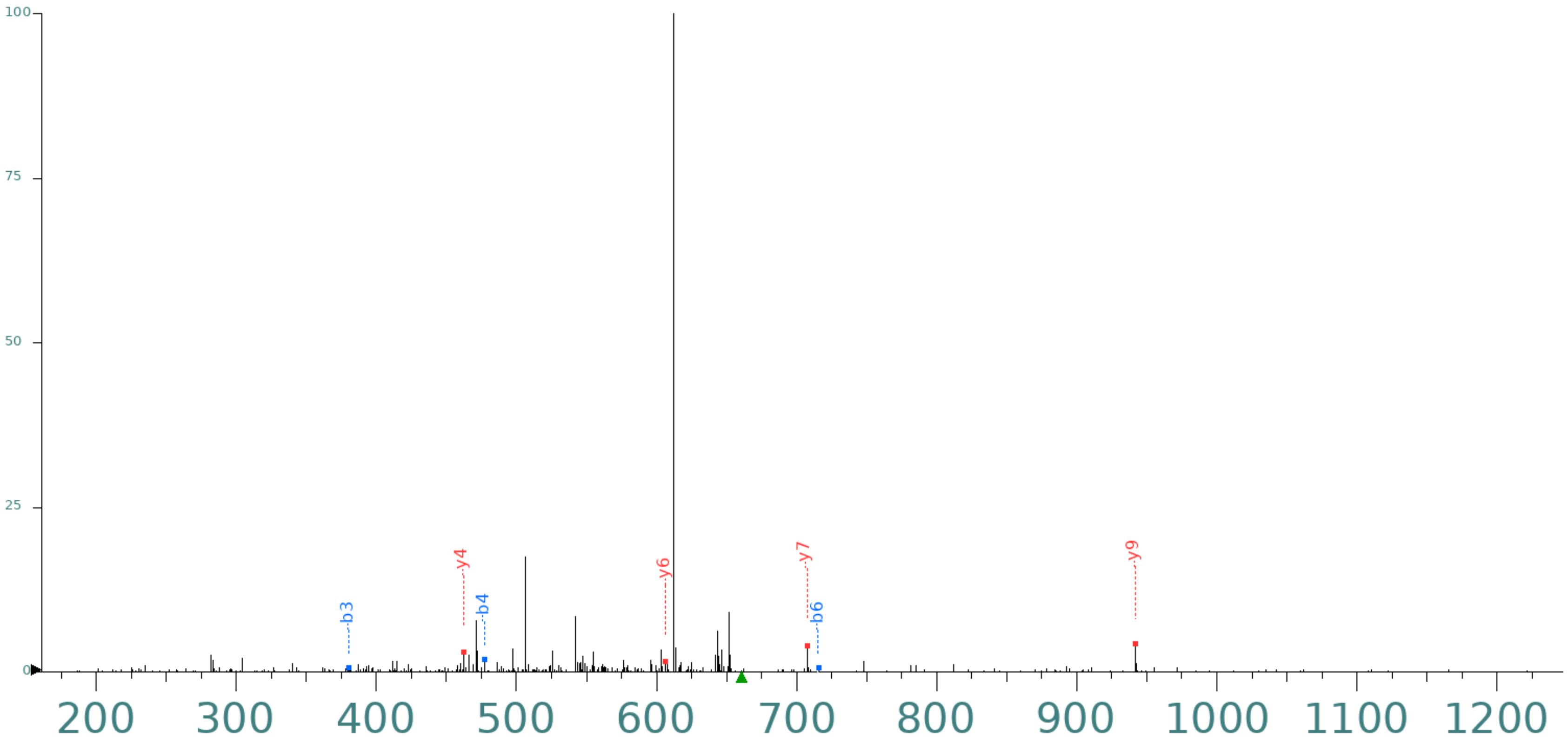

LPA3-PMA

25 jun 2020

Predicted Fragmentation Pattern

Seq	#	b: Δ Error	b	y	y: Δ Error	+1
V	1	---	100.076	---	---	12
L	2	---	213.160	1221.562	---	11
S#	3	-56.119	**380.158**	1108.478	---	10
P	4	---	477.211	**941.480**	123.873	9
H	5	537.140	**614.270**	844.427	---	8
T	6	309.861	**715.317**	**707.368**	152.070	7
S	7	-520.984	**802.349**	**606.321**	188.797	6
G	8	---	859.371	**519.289**	624.973	5
S	9	-280.782	**946.403**	**462.267**	105.388	4
I	10	---	1059.487	375.235	---	3
S	11	---	1146.519	262.151	---	2
R	12	---	---	175.119	---	1

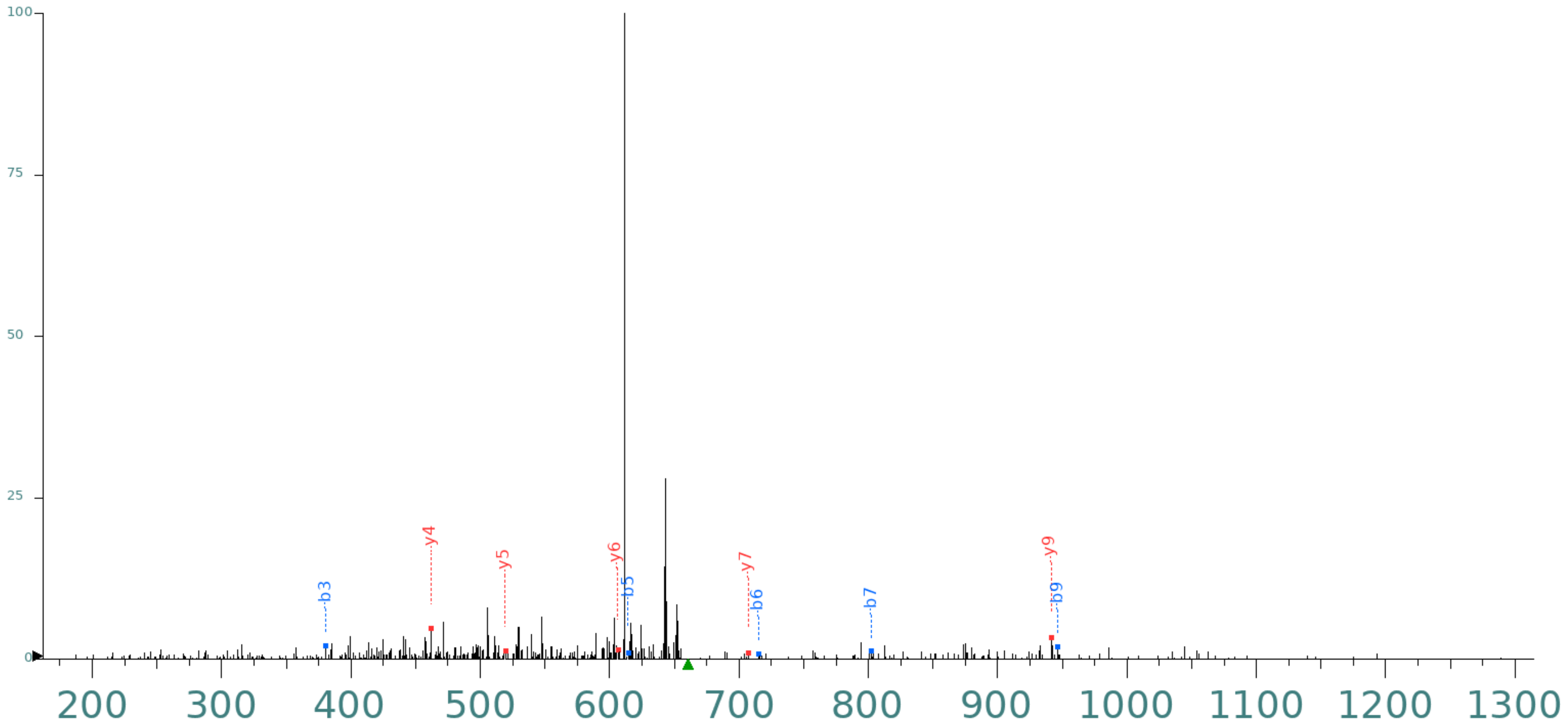

Jan 24 2020 LPA3-Basal

Predicted Fragmentation Pattern

Seq	#	b: Δ Error	b	y	y: Δ Error	+1
S	1	---	88.039	---	---	20
D	2	---	203.066	2151.890	---	19
T	3	---	304.114	2036.863	---	18
G	4	-170.259	**361.135**	1935.815	---	17
S#	5	---	528.134	1878.794	---	16
Q	6	192.031	**656.192**	**1711.796**	-117.560	15
Y	7	-30.594	**819.256**	**1583.737**	-117.980	14
I	8	123.192	**932.340**	**1420.674**	-17.675	13
E	9	229.406	**1061.382**	**1307.590**	-143.046	12
D	10	-79.139	**1176.409**	**1178.547**	-59.470	11
S	11	-67.153	**1263.441**	**1063.520**	-190.733	10
I	12	55.542	**1376.525**	**976.488**	-133.032	9
S	13	-66.731	**1463.557**	**863.404**	-96.204	8
Q	14	4.534	**1591.616**	**776.372**	-17.140	7
G	15	-99.429	**1648.637**	**648.313**	-105.479	6
A	16	-8.359	**1719.675**	**591.292**	279.181	5
V	17	42.806	**1818.743**	**520.255**	-221.609	4
C	18	-9.147	**1978.774**	**421.186**	-227.659	3
N	19	---	2092.817	261.156	---	2
K	20	---	---	147.113	---	1

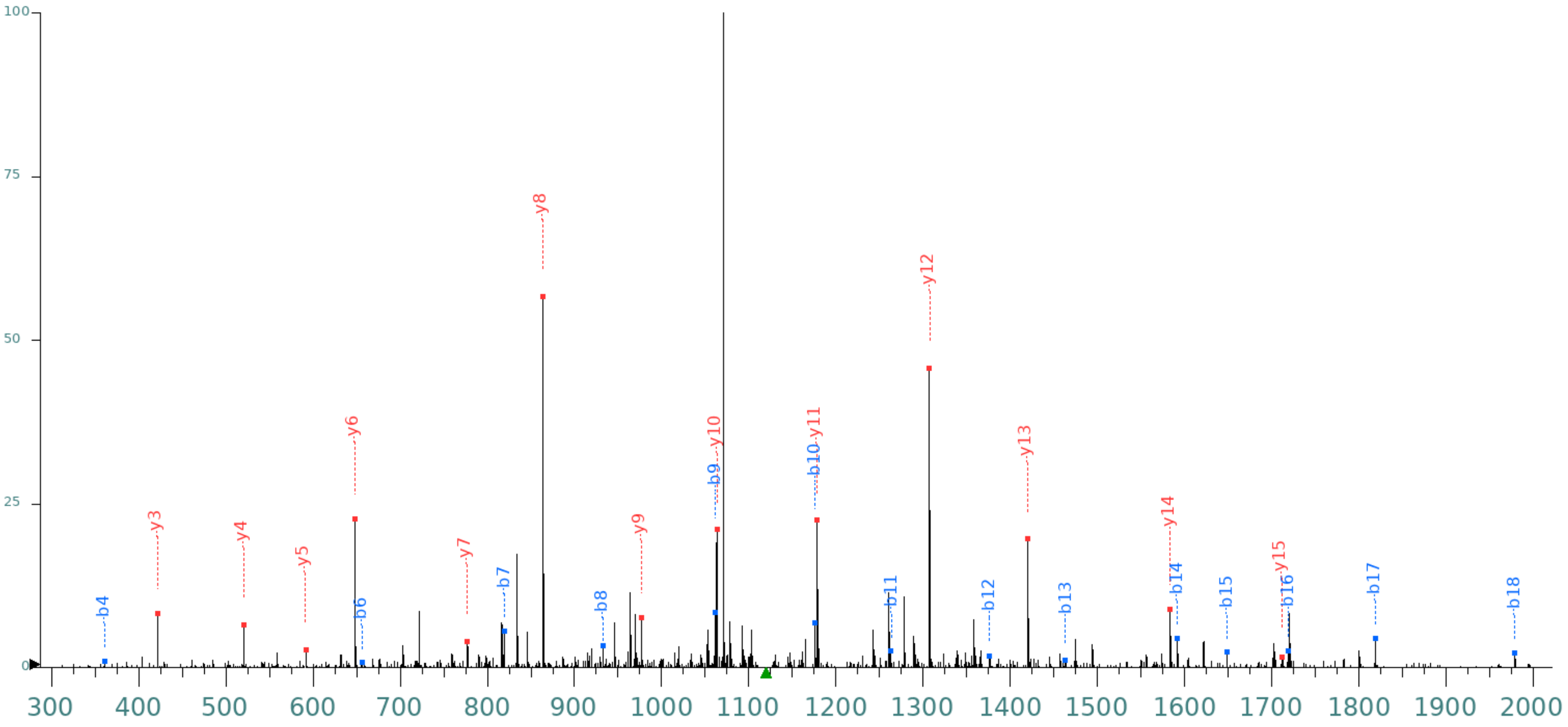

Jan 24 2020 LPA3-Basal

Predicted Fragmentation Pattern

Seq	#	b: Δ Error	b	y	y: Δ Error	+1
T	1	---	102.055	---	---	14
N	2	---	216.098	1514.640	---	13
V	3	-6.699	315.166	1400.597	---	12
L	4	-208.325	428.250	1301.529	-8.900	11
S#	5	---	595.249	1188.445	-21.903	10
P	6	---	692.301	1021.446	7.268	9
H	7	-32.360	829.360	924.393	250.585	8
T	8	---	930.408	787.335	120.092	7
S	9	---	1017.440	686.287	-127.416	6
G	10	---	1074.462	599.255	230.972	5
S#	11	---	1241.460	542.233	---	4
I	12	---	1354.544	375.235	-69.944	3
S	13	223.694	1441.576	262.151	443.992	2
R	14	---	---	175.119	---	1

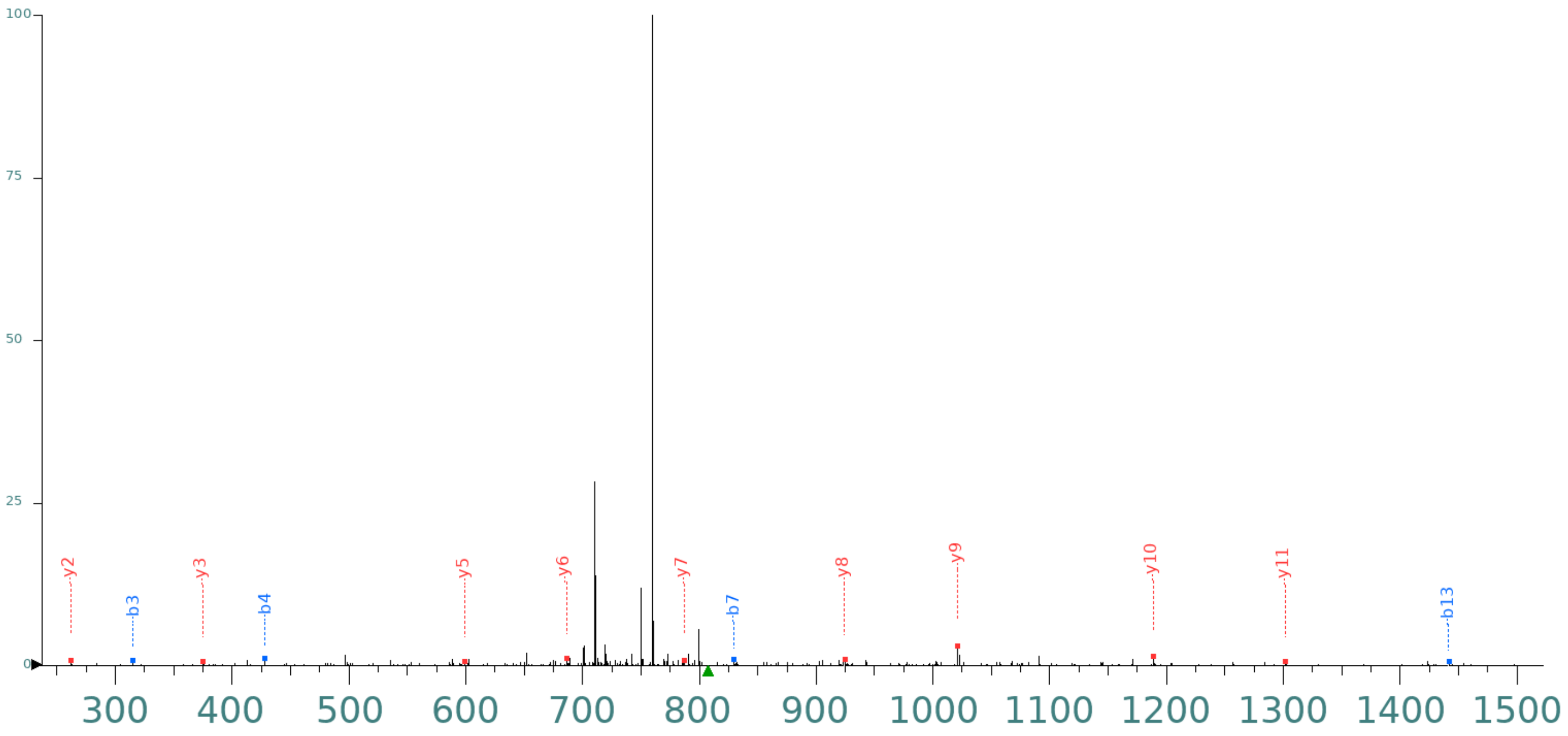

Predicted Fragmentation Pattern

Seq	#	b: Δ Error	b	y	y: Δ Error	+1
I	1	---	114.091	---	---	8
P	2	-345.122	211.144	839.402	-127.382	7
S#	3	-453.052	378.142	742.349	-29.365	6
T	4	---	479.190	575.351	143.613	5
V	5	-143.692	578.259	474.303	113.567	4
L	6	-46.570	691.343	375.235	-246.973	3
S	7	---	778.375	262.151	135.678	2
R	8	---	---	175.119	---	1

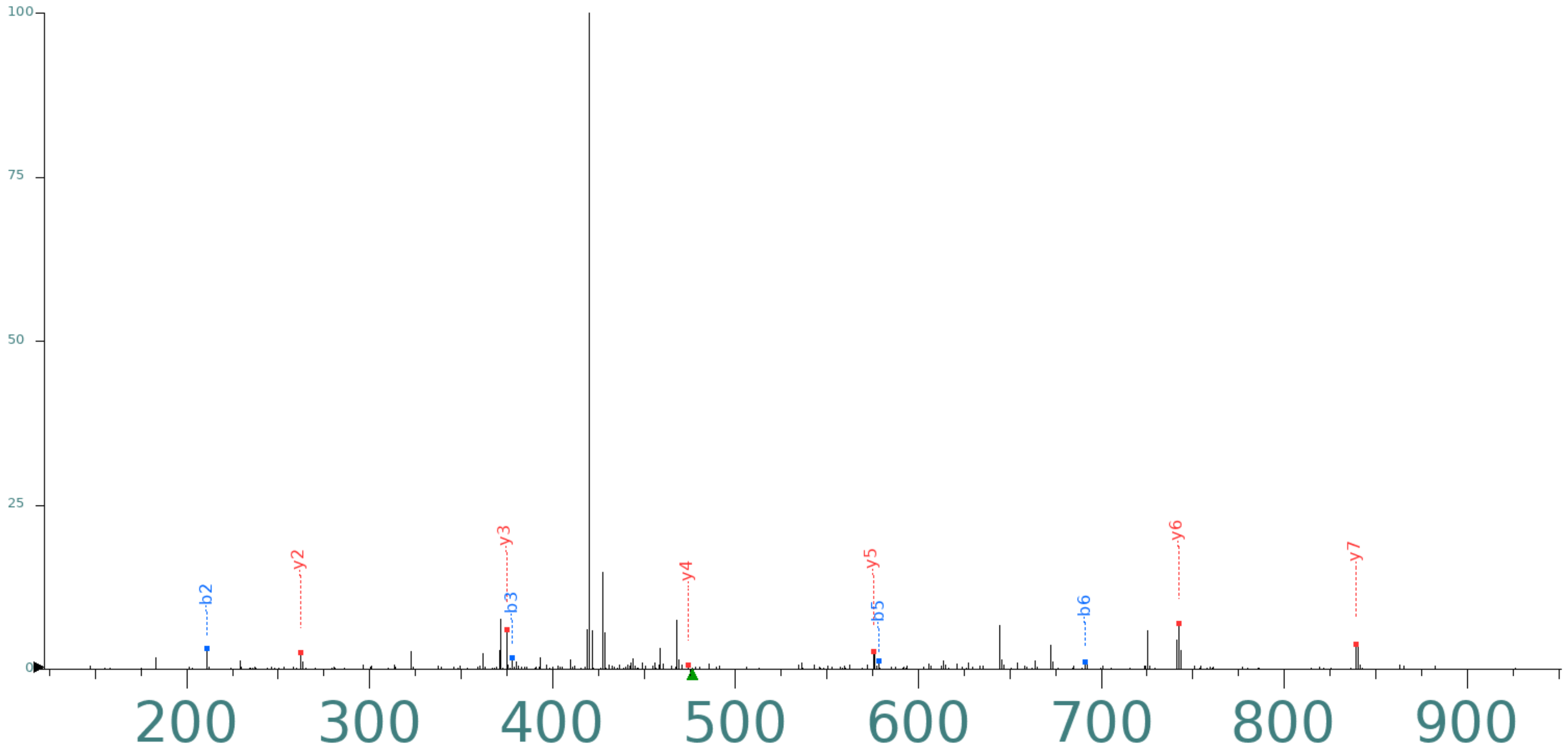

Predicted Fragmentation Pattern

+1

Seq	#	b: Δ Error	b	y	y: Δ Error	+1
R	1	---	157.108	---	---	12
P	2	52.818	**254.161**	1372.639	---	11
S#	3	---	421.160	1275.586	---	10
R	4	---	577.261	1108.587	---	9
I	5	-149.998	**690.345**	952.486	---	8
P	6	---	787.397	**839.402**	35.728	7
S	7	---	874.429	**742.349**	72.253	6
T	8	---	975.477	655.317	---	5
V	9	---	1074.546	554.270	---	4
L	10	---	1187.630	455.201	---	3
S#	11	---	1354.628	342.117	---	2
R	12	---	---	175.119	---	1

+2

Seq	#	b: Δ Error	b	y	y: Δ Error	+1
R	1	---	79.058	---	---	12
P	2	---	127.584	686.823	---	11
S#	3	---	211.083	**638.297**	455.581	10
R	4	-574.641	**289.134**	**554.797**	10.575	9
I	5	57.158	**345.676**	**476.747**	-30.672	8
P	6	---	394.202	**420.205**	508.422	7
S	7	---	437.718	**371.678**	111.789	6
T	8	-174.102	**488.242**	**328.162**	-234.831	5
V	9	---	537.776	277.639	---	4
L	10	---	594.318	228.104	---	3
S#	11	---	677.818	171.562	---	2
R	12	---	---	88.063	---	1

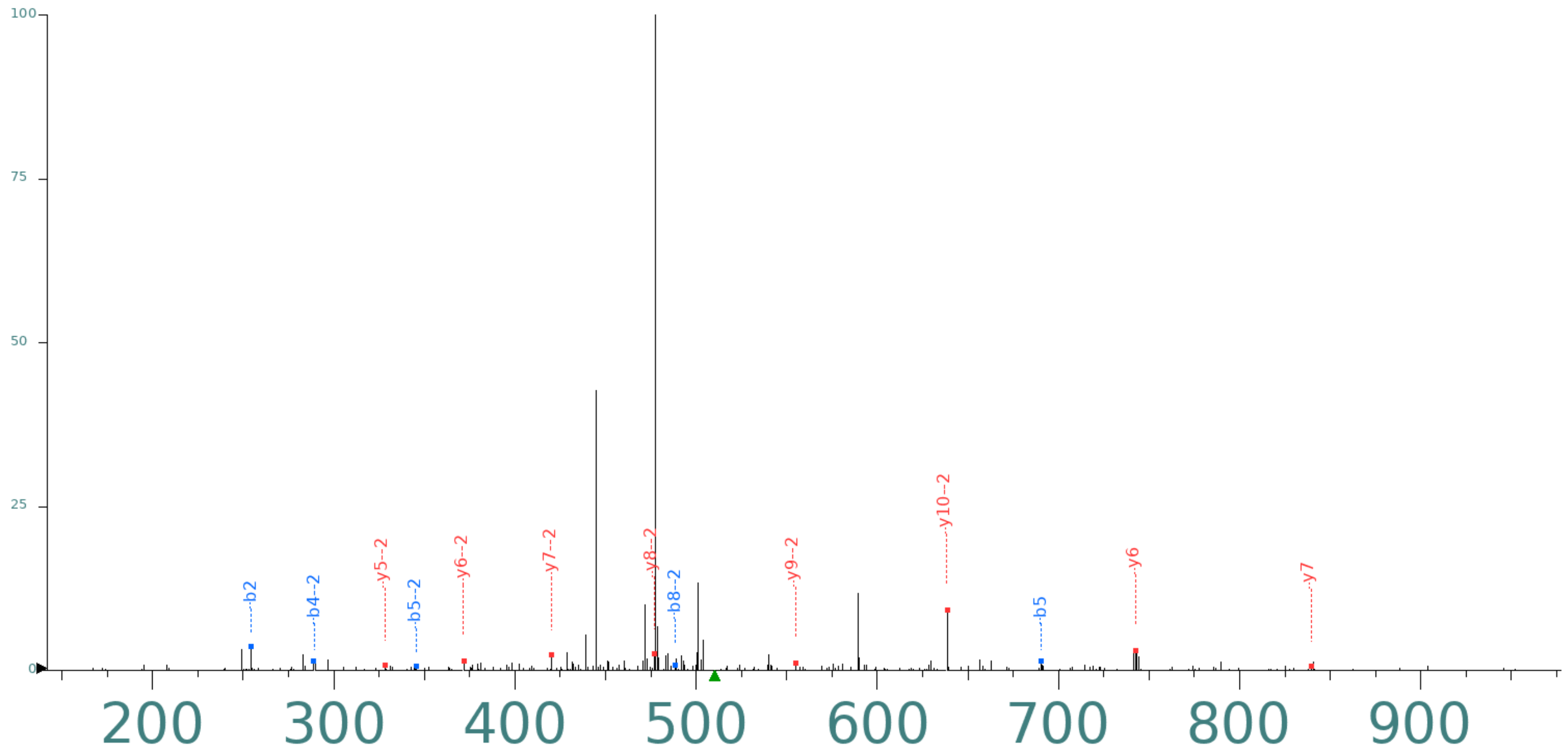

Predicted Fragmentation Pattern

Seq	#	b: Δ Error	b	y	y: Δ Error	+1
I	1	---	114.091	---	---	8
P	2	226.724	211.144	839.402	-7.899	7
S#	3	228.484	378.142	742.349	-8.234	6
T	4	---	479.190	575.351	132.583	5
V	5	81.144	578.259	474.303	---	4
L	6	---	691.343	375.235	87.100	3
S	7	---	778.375	262.151	424.684	2
R	8	---	---	175.119	-59.923	1

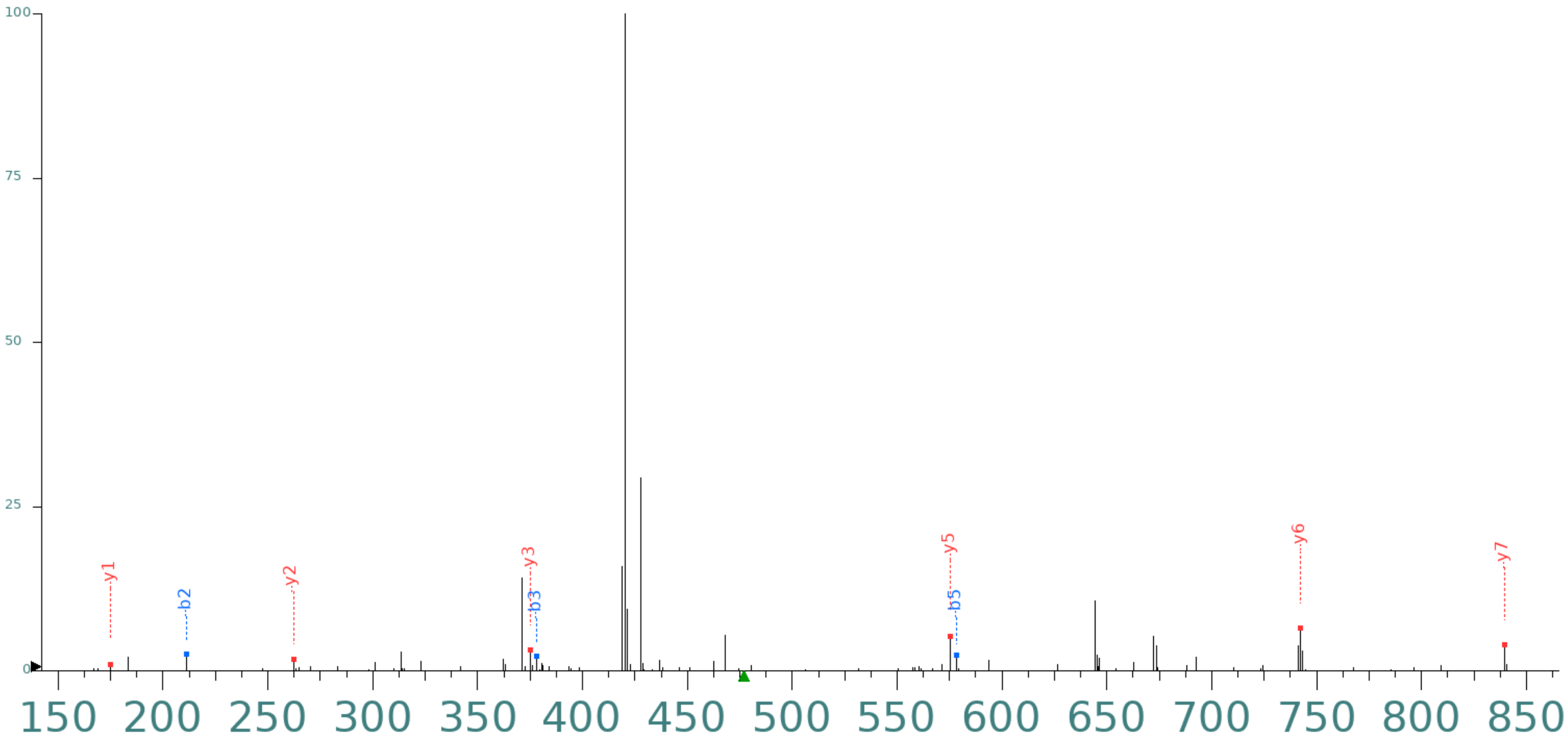

Predicted Fragmentation Pattern

+1

Seq	#	b: Δ Error	b	y	y: Δ Error	+1
R	1	---	157.108	---	---	12
P	2	-18.142	254.161	1372.639	---	11
S#	3	-879.492	421.160	1275.586	---	10
R	4	83.098	577.261	1108.587	---	9
I	5	-198.465	690.345	952.486	125.457	8
P	6	---	787.397	839.402	-128.400	7
S#	7	---	954.396	742.349	---	6
T	8	---	1055.443	575.351	296.942	5
V	9	---	1154.512	474.303	---	4
L	10	---	1267.596	375.235	-419.261	3
S	11	---	1354.628	262.151	---	2
R	12	---	---	175.119	---	1

+2

Seq	#	b: Δ Error	b	y	y: Δ Error	+1
R	1	---	79.058	---	---	12
P	2	---	127.584	686.823	---	11
S#	3	---	211.083	638.297	175.108	10
R	4	-610.042	289.134	554.797	---	9
I	5	1340.861	345.676	476.747	---	8
P	6	-241.829	394.202	420.205	-226.486	7
S#	7	110.500	477.702	371.678	---	6
T	8	---	528.225	288.179	---	5
V	9	-781.201	577.760	237.655	---	4
L	10	---	634.302	188.121	---	3
S	11	-684.923	677.818	131.579	---	2
R	12	---	---	88.063	---	1

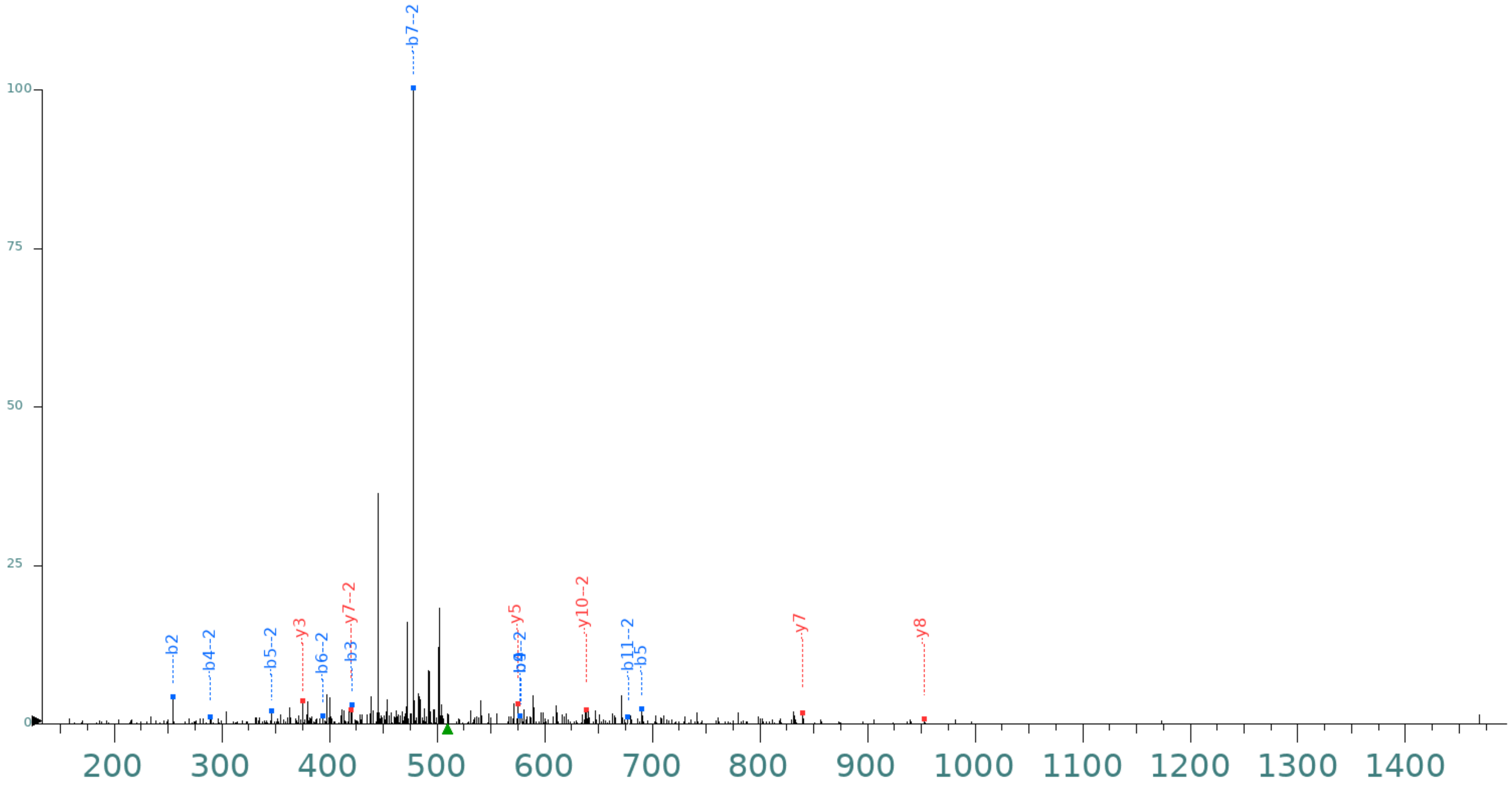

Predicted Fragmentation Pattern

+1

Seq	#	b: Δ Error	b	y	y: Δ Error	+1
R	1	---	157.108	---	---	12
P	2	-250.904	254.161	1372.639	---	11
S#	3	919.203	421.160	1275.586	---	10
R	4	267.219	577.261	1108.587	---	9
I	5	-46.181	690.345	952.486	107.519	8
P	6	72.100	787.397	839.402	-44.766	7
S	7	44.562	874.429	742.349	---	6
T#	8	---	1055.443	655.317	121.215	5
V	9	---	1154.512	474.303	-156.230	4
L	10	---	1267.596	375.235	-136.078	3
S	11	---	1354.628	262.151	---	2
R	12	---	---	175.119	-1362.420	1

+2

Seq	#	b: Δ Error	b	y	y: Δ Error	+1
R	1	---	79.058	---	---	12
P	2	---	127.584	686.823	-357.339	11
S#	3	---	211.083	638.297	24.246	10
R	4	-115.823	289.134	554.797	624.061	9
I	5	1011.192	345.676	476.747	---	8
P	6	-1059.166	394.202	420.205	-571.296	7
S	7	456.751	437.718	371.678	-1149.046	6
T#	8	---	528.225	328.162	---	5
V	9	-596.921	577.760	237.655	---	4
L	10	546.953	634.302	188.121	---	3
S	11	661.034	677.818	131.579	---	2
R	12	---	---	88.063	---	1

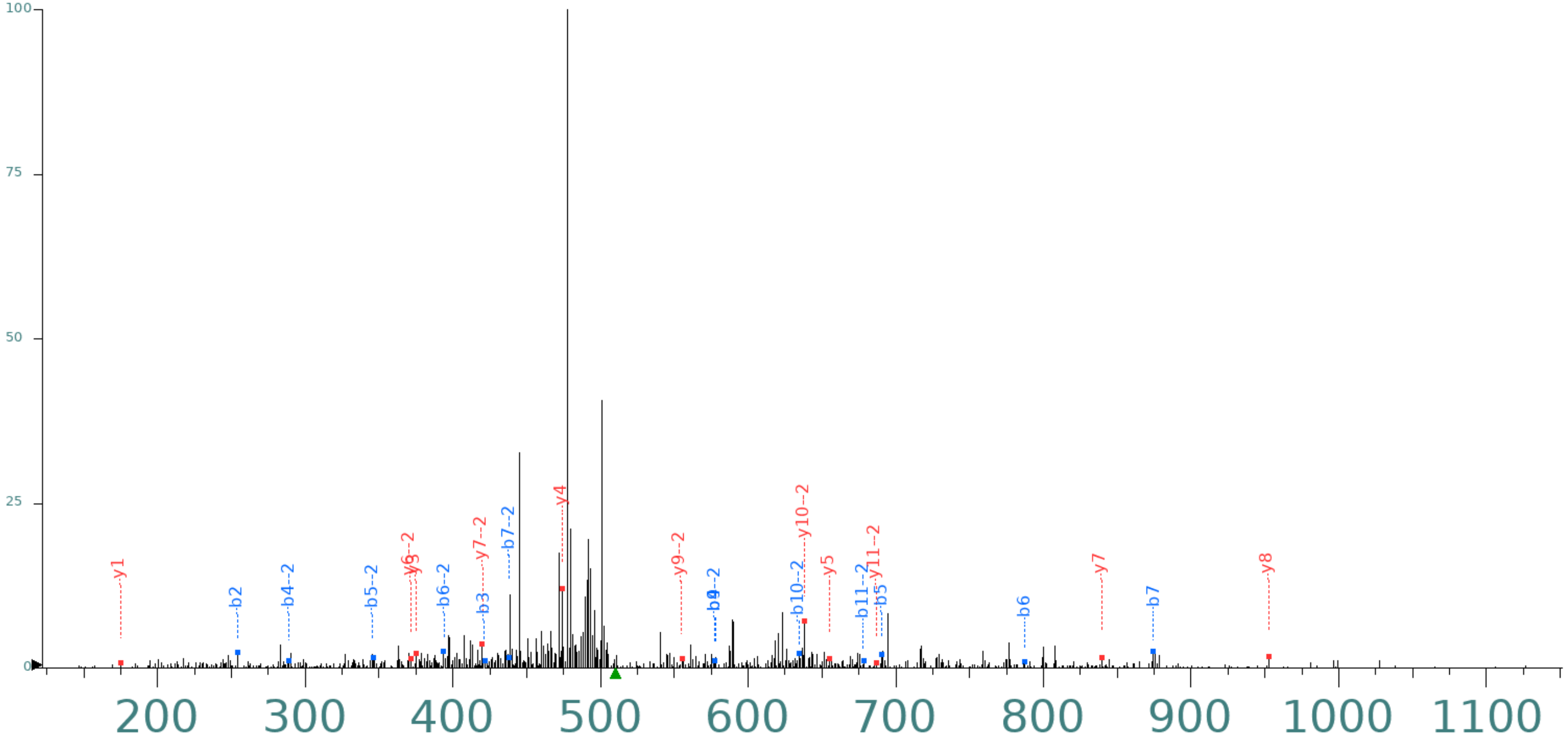

Predicted Fragmentation Pattern

+1

Seq	#	b: Δ Error	b	y	y: Δ Error	+1
R	1	---	157.108	---	---	12
P	2	-22.945	**254.161**	1372.639	---	11
S#	3	---	421.160	1275.586	---	10
R	4	---	577.261	1108.587	---	9
I	5	-91.543	**690.345**	952.486	---	8
P	6	---	787.397	839.402	---	7
S#	7	---	954.396	**742.349**	308.132	6
T	8	---	1055.443	**575.351**	193.773	5
V	9	---	1154.512	474.303	---	4
L	10	---	1267.596	**375.235**	928.734	3
S	11	---	1354.628	262.151	---	2
R	12	---	---	175.119	---	1

+2

Seq	#	b: Δ Error	b	y	y: Δ Error	+1
R	1	---	79.058	---	---	12
P	2	---	127.584	686.823	---	11
S#	3	---	211.083	**638.297**	-34.275	10
R	4	---	289.134	554.797	---	9
I	5	-12.053	**345.676**	476.747	---	8
P	6	---	394.202	**420.205**	145.096	7
S#	7	-8.696	**477.702**	371.678	---	6
T	8	---	528.225	288.179	---	5
V	9	---	577.760	237.655	---	4
L	10	---	634.302	188.121	---	3
S	11	194.722	**677.818**	131.579	---	2
R	12	---	---	88.063	---	1

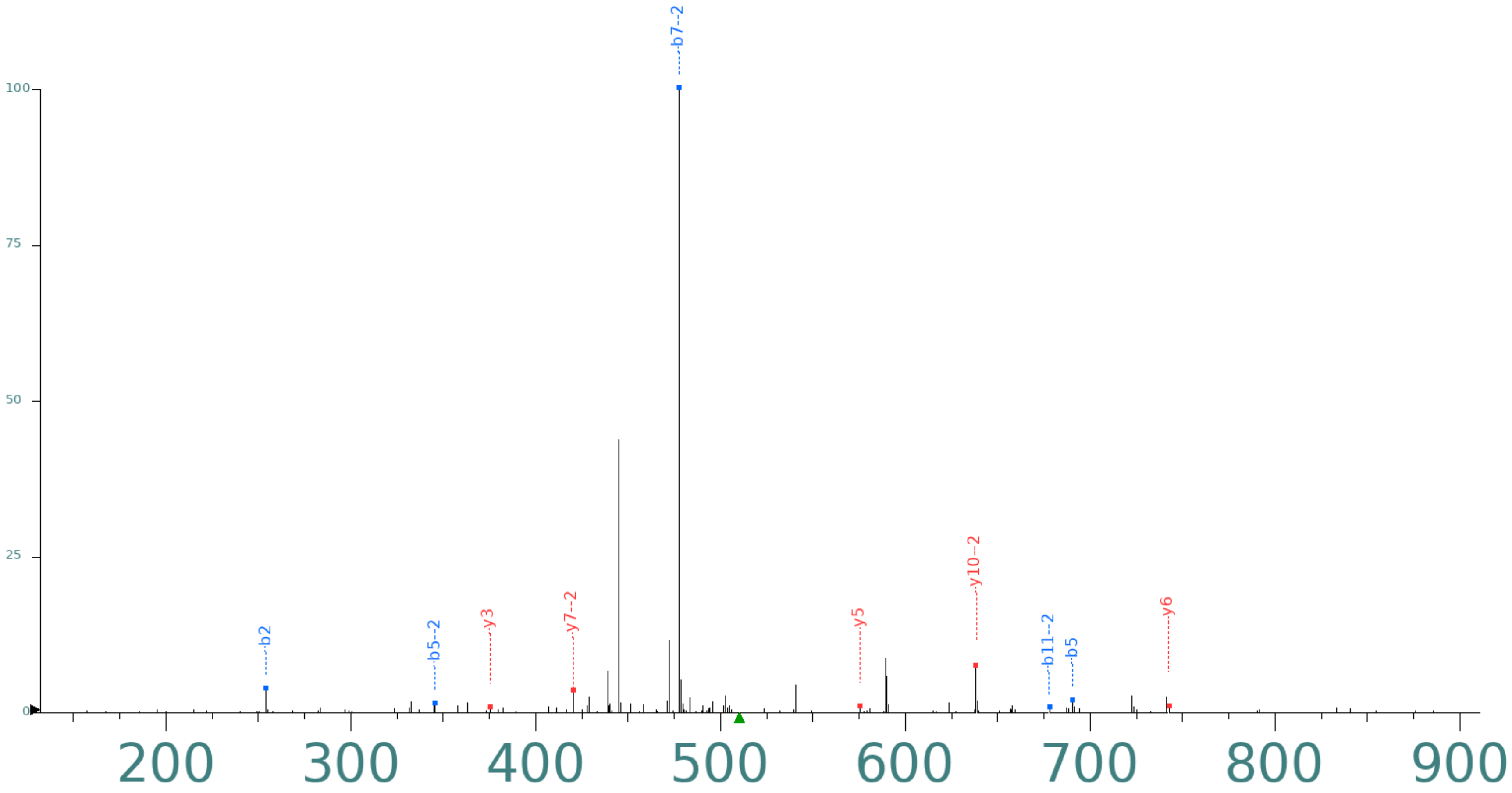

Predicted Fragmentation Pattern

+1

Seq	#	b: Δ Error	b	y	y: Δ Error	+1
R	1	---	157.108	---	---	12
P	2	-116.374	**254.161**	1372.639	---	11
S#	3	-1001.310	**421.160**	1275.586	---	10
R	4	---	577.261	1108.587	---	9
I	5	-67.756	**690.345**	952.486	---	8
P	6	291.930	**787.397**	**839.402**	73.825	7
S	7	---	874.429	**742.349**	-60.446	6
T#	8	---	1055.443	**655.317**	81.919	5
V	9	---	1154.512	474.303	---	4
L	10	---	1267.596	**375.235**	-367.251	3
S	11	---	1354.628	**262.151**	-280.090	2
R	12	---	---	**175.119**	-395.541	1

+2

Seq	#	b: Δ Error	b	y	y: Δ Error	+1
R	1	---	79.058	---	---	12
P	2	---	127.584	686.823	---	11
S#	3	-1673.460	**211.083**	**638.297**	101.882	10
R	4	---	289.134	554.797	---	9
I	5	1073.214	**345.676**	**476.747**	235.820	8
P	6	-581.036	**394.202**	**420.205**	-556.027	7
S	7	---	437.718	**371.678**	-1146.907	6
T#	8	-293.189	**528.225**	**328.162**	-733.024	5
V	9	---	577.760	**237.655**	-1900.632	4
L	10	-523.516	**634.302**	188.121	---	3
S	11	-647.324	**677.818**	131.579	---	2
R	12	---	---	88.063	---	1

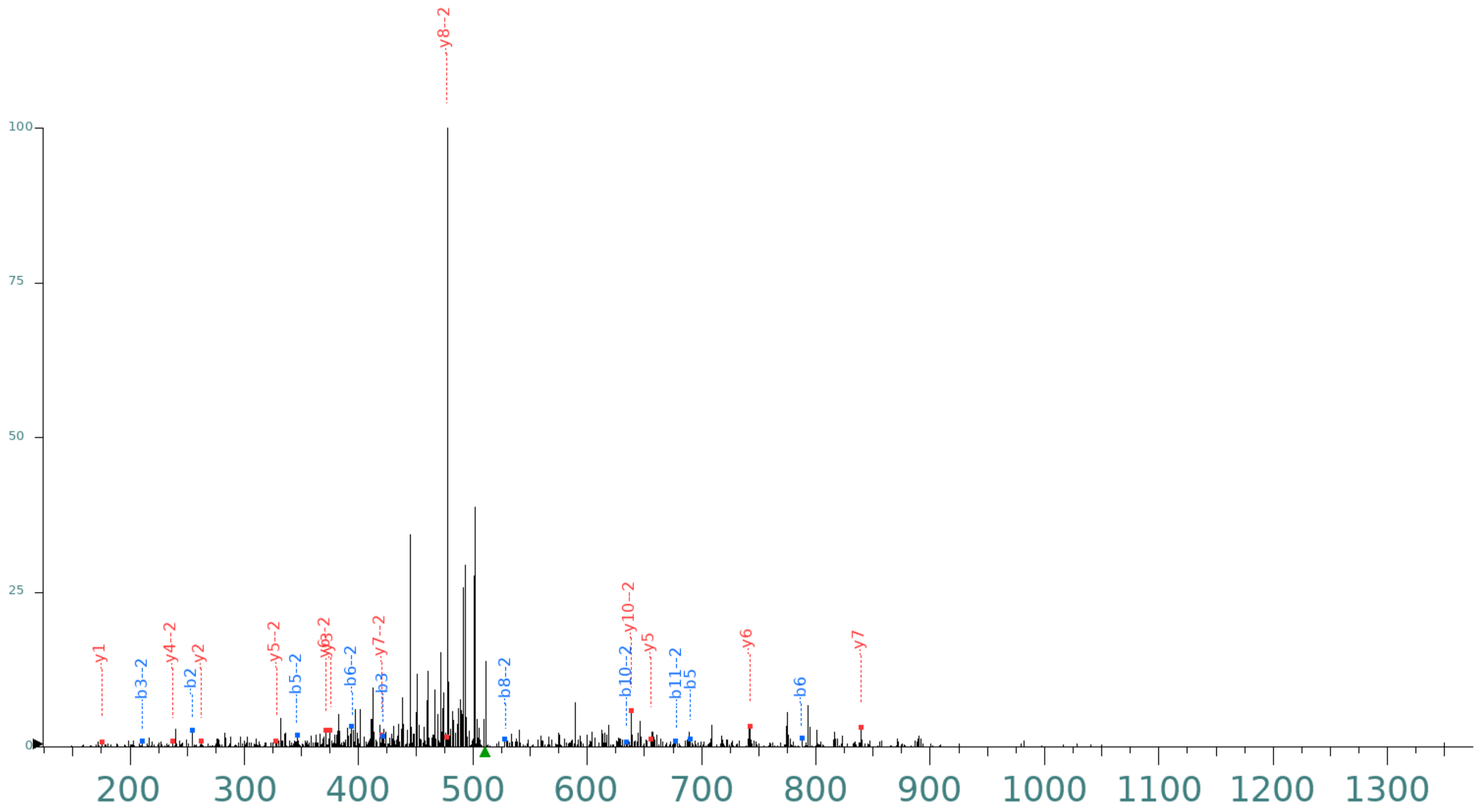

Predicted Fragmentation Pattern

+1

Seq	#	b: Δ Error	b	y	y: Δ Error	+1
R	1	---	157.108	---	---	12
P	2	69.146	**254.161**	1372.639	---	11
S#	3	-463.153	**421.160**	1275.586	---	10
R	4	-93.900	**577.261**	1108.587	---	9
I	5	68.576	**690.345**	952.486	---	8
P	6	436.622	**787.397**	**839.402**	114.464	7
S	7	-162.489	**874.429**	**742.349**	12.074	6
T#	8	---	1055.443	**655.317**	-698.782	5
V	9	---	1154.512	**474.303**	-75.526	4
L	10	---	1267.596	**375.235**	-385.483	3
S	11	---	1354.628	**262.151**	373.736	2
R	12	---	---	**175.119**	-510.837	1

+2

Seq	#	b: Δ Error	b	y	y: Δ Error	+1
R	1	---	79.058	---	---	12
P	2	---	127.584	**686.823**	-52.493	11
S#	3	---	211.083	**638.297**	-364.493	10
R	4	---	289.134	**554.797**	265.736	9
I	5	684.390	**345.676**	476.747	---	8
P	6	157.052	**394.202**	**420.205**	-222.272	7
S	7	880.219	**437.718**	**371.678**	-1270.201	6
T#	8	34.820	**528.225**	**328.162**	-417.221	5
V	9	451.719	**577.760**	237.655	---	4
L	10	82.771	**634.302**	188.121	---	3
S	11	---	677.818	131.579	---	2
R	12	---	---	88.063	---	1

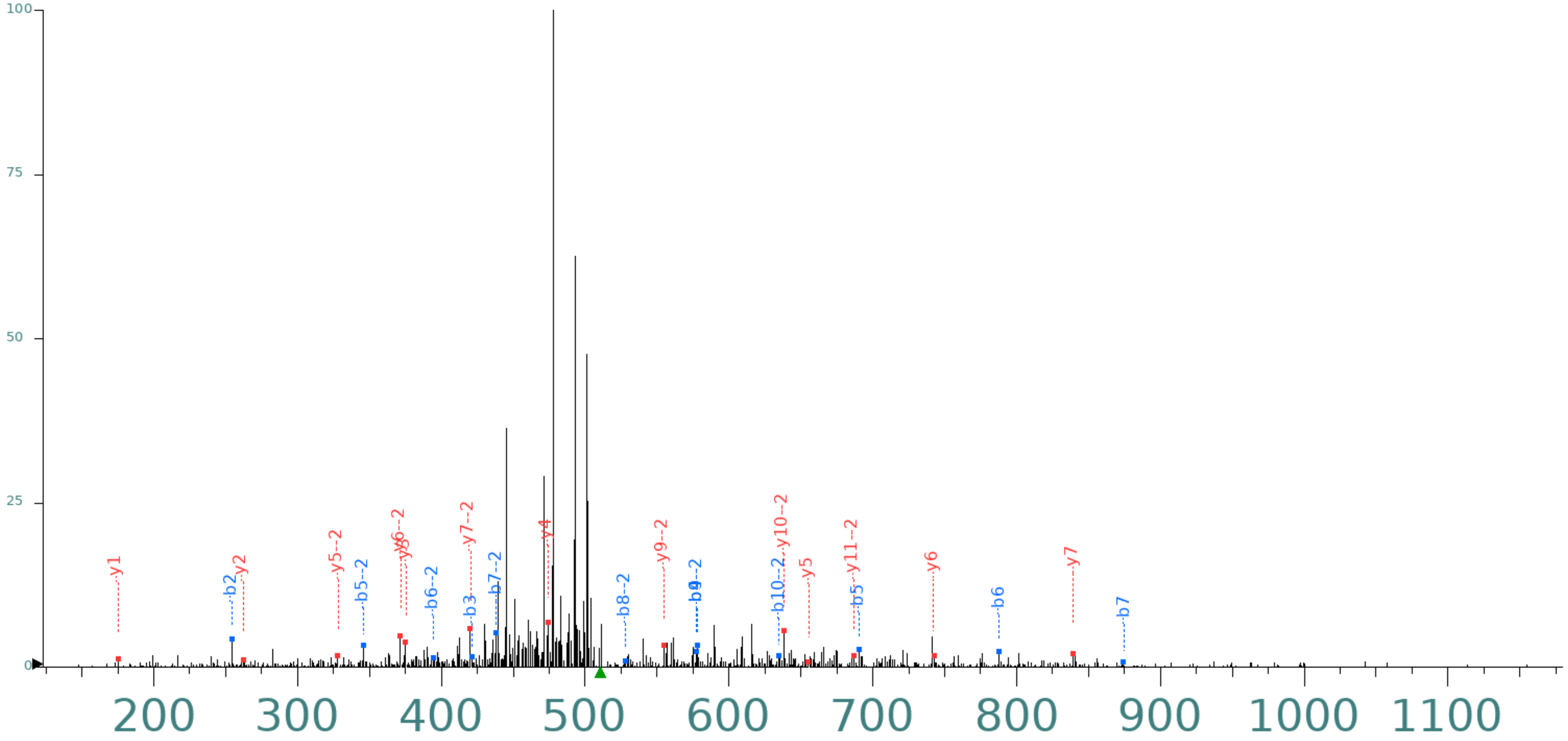

Predicted Fragmentation Pattern

Seq	#	b: Δ Error	b	y	y: Δ Error	+1
I	1	---	114.091	---	---	8
P	2	-92.365	211.144	839.402	-392.484	7
S	3	-450.071	298.176	742.349	128.973	6
T#	4	---	479.190	655.317	385.221	5
V	5	-57.862	578.259	474.303	---	4
L	6	---	691.343	375.235	-379.867	3
S	7	-122.122	778.375	262.151	-16.687	2
R	8	---	---	175.119	11.791	1

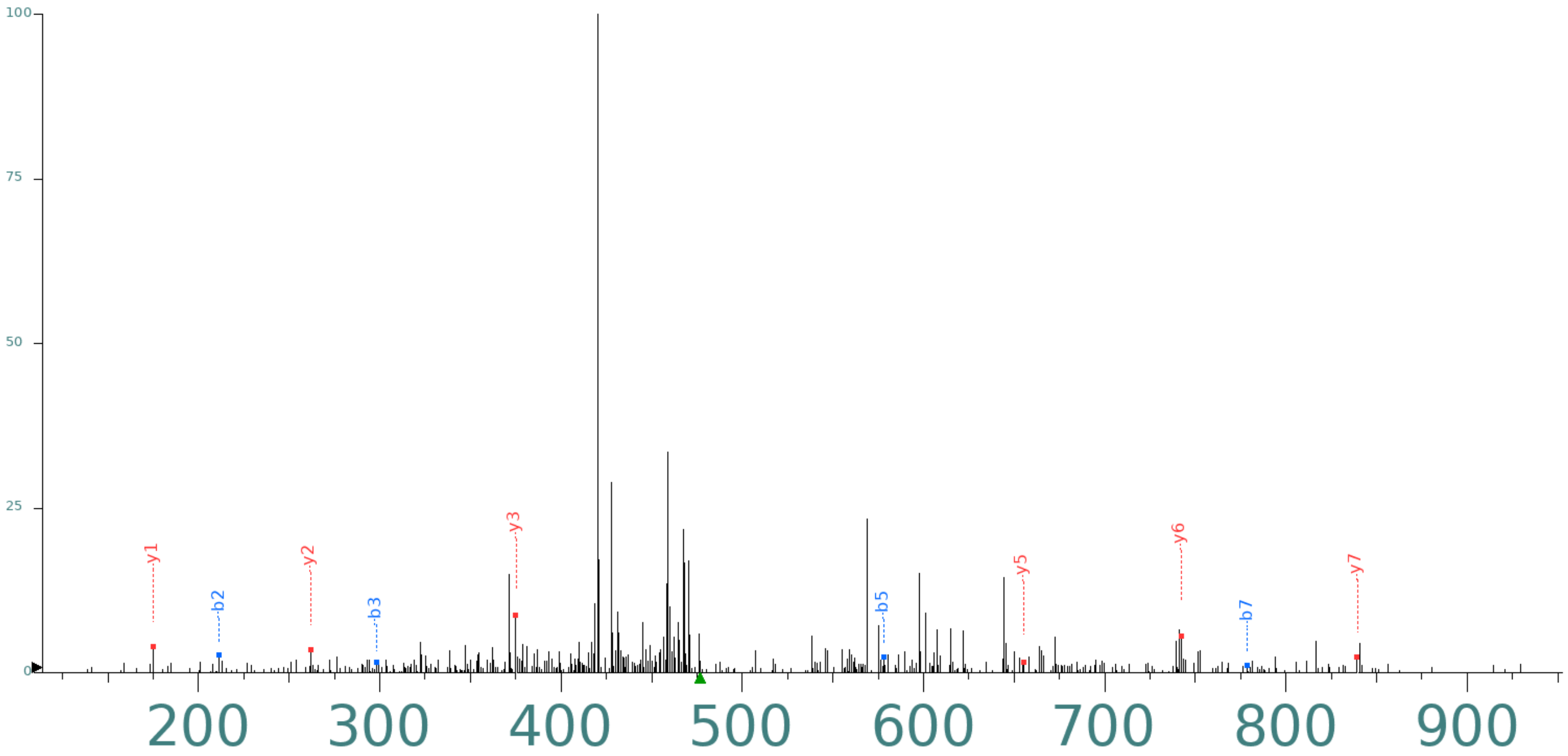

Predicted Fragmentation Pattern

Seq	#	b: Δ Error	b	y	y: Δ Error	+1
T	1	---	102.055	---	---	14
N	2	---	216.098	1434.674	---	13
V	3	---	315.166	1320.631	---	12
L	4	---	428.250	1221.562	---	11
S#	5	---	595.249	**1108.478**	87.204	10
P	6	---	692.301	**941.480**	-20.357	9
H	7	---	829.360	844.427	---	8
T	8	---	930.408	**707.368**	-65.349	7
S	9	---	1017.440	**606.321**	162.835	6
G	10	---	1074.462	**519.289**	432.652	5
S	11	-15.294	**1161.494**	**462.267**	461.283	4
I	12	---	1274.578	375.235	---	3
S	13	---	1361.610	**262.151**	40.470	2
R	14	---	---	175.119	---	1

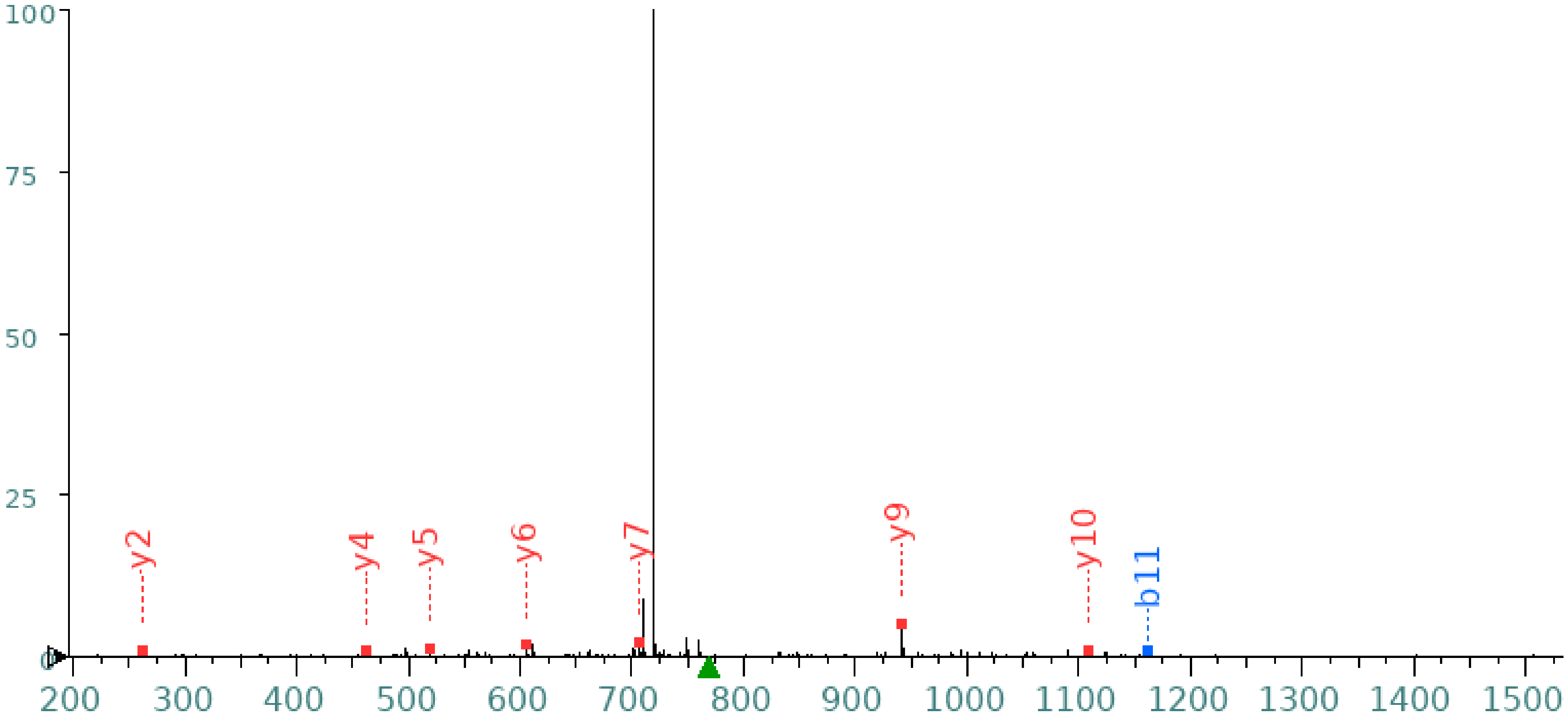

Predicted Fragmentation Pattern

+1

Seq	#	b: Δ Error	b	y	y: Δ Error	+1
S	1	---	88.039	---	---	20
D	2	---	203.066	2151.890	---	19
T	3	---	304.114	2036.863	---	18
G	4	---	361.135	1935.815	---	17
S	5	---	448.167	1878.794	---	16
Q	6	---	576.226	1791.762	---	15
Y#	7	-263.552	**819.256**	1663.703	---	14
I	8	-213.129	**932.340**	1420.674	---	13
E	9	-58.302	**1061.382**	1307.590	---	12
D	10	-110.171	**1176.409**	**1178.547**	-132.713	11
S	11	44.056	**1263.441**	1063.520	---	10
I	12	-152.876	**1376.525**	**976.488**	-364.290	9
S	13	-190.872	**1463.557**	**863.404**	-118.971	8
Q	14	---	1591.616	**776.372**	145.024	7
G	15	---	1648.637	**648.313**	-144.088	6
A	16	---	1719.675	**591.292**	-23.083	5
V	17	---	1818.743	**520.255**	-274.898	4
C	18	---	1978.774	**421.186**	-430.235	3
N	19	---	2092.817	**261.156**	-499.694	2
K	20	---	---	147.113	---	1

+2

Seq	#	b: Δ Error	b	y	y: Δ Error	+1
S	1	---	44.523	---	---	20
D	2	---	102.037	1076.449	---	19
T	3	---	152.561	**1018.935**	-122.389	18
G	4	---	181.071	968.411	---	17
S	5	---	224.587	939.901	---	16
Q	6	---	288.617	**896.385**	309.569	15
Y#	7	---	410.131	832.355	---	14
I	8	---	466.673	**710.840**	162.658	13
E	9	-281.991	**531.195**	**654.298**	-354.944	12
D	10	---	588.708	**589.777**	753.993	11
S	11	172.334	**632.224**	**532.264**	300.575	10
I	12	-248.295	**688.766**	488.748	---	9
S	13	-86.409	**732.282**	**432.206**	284.404	8
Q	14	-275.124	**796.312**	**388.690**	160.282	7
G	15	-131.165	**824.822**	324.660	---	6
A	16	-252.220	**860.341**	296.150	---	5
V	17	225.957	**909.875**	**260.631**	1510.444	4
C	18	252.004	**989.890**	211.097	---	3
N	19	-30.115	**1046.912**	131.082	---	2
K	20	---	---	74.060	---	1

LPA3-LPA

Jan 24 2020

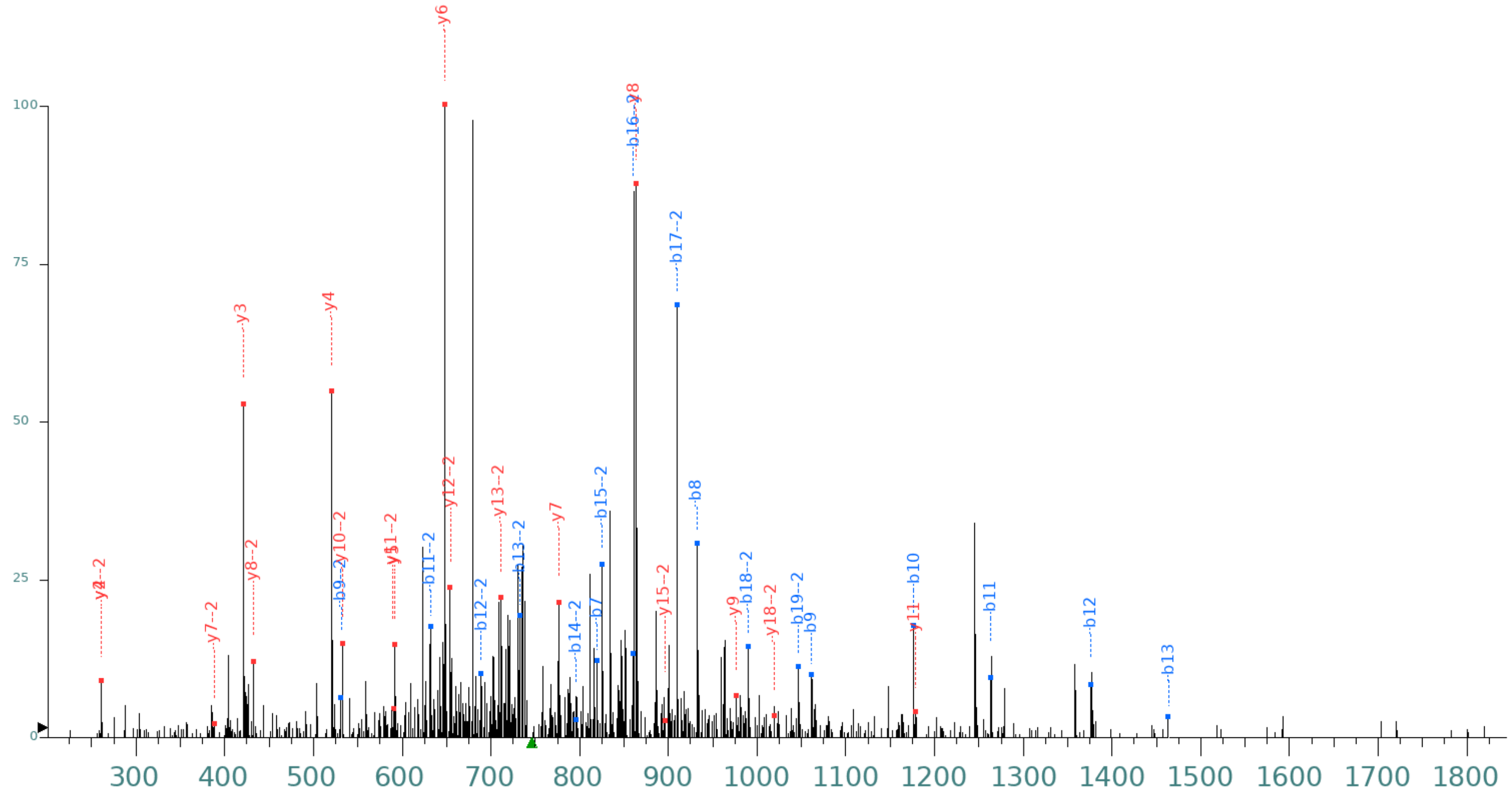

Predicted Fragmentation Pattern

Seq	#	b: Δ Error	b	y	y: Δ Error	+1
S	1	---	88.039	---	---	20
D	2	---	203.066	2151.890	---	19
T	3	---	304.114	2036.863	---	18
G	4	-584.559	361.135	1935.815	---	17
S#	5	---	528.134	1878.794	---	16
Q	6	-167.181	656.192	1711.796	-8.654	15
Y	7	140.292	819.256	1583.737	-119.214	14
I	8	-45.169	932.340	1420.674	-105.243	13
E	9	-172.649	1061.382	1307.590	-116.619	12
D	10	-176.599	1176.409	1178.547	-126.808	11
S	11	4.832	1263.441	1063.520	-153.187	10
I	12	6.505	1376.525	976.488	-82.518	9
S	13	106.914	1463.557	863.404	-114.446	8
Q	14	-141.822	1591.616	776.372	34.903	7
G	15	-41.889	1648.637	648.313	-167.255	6
A	16	-33.417	1719.675	591.292	-273.887	5
V	17	-12.699	1818.743	520.255	-193.441	4
C	18	47.050	1978.774	421.186	-473.675	3
N	19	---	2092.817	261.156	---	2
K	20	---	---	147.113	---	1

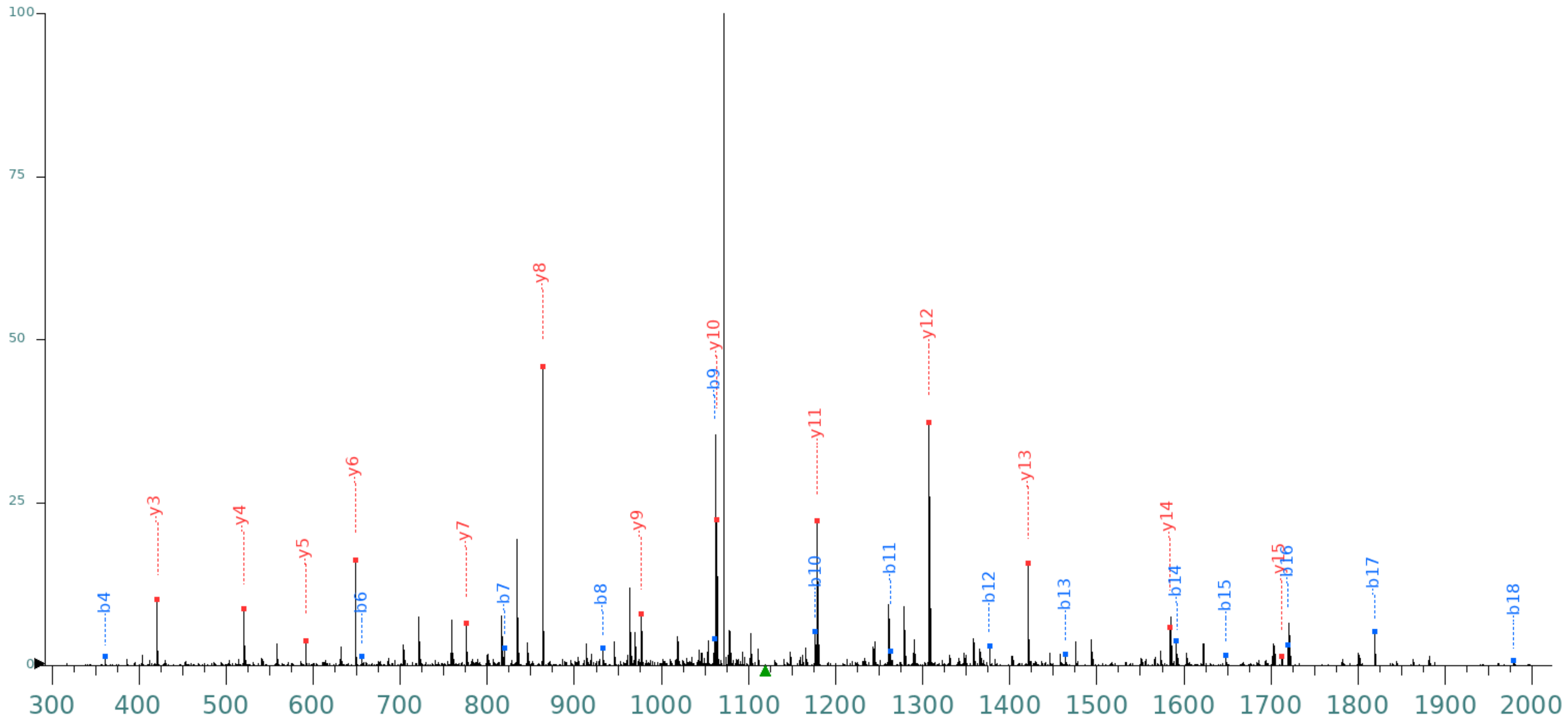

Predicted Fragmentation Pattern

Seq	#	b: Δ Error	b	y	y: Δ Error	+1
T	1	---	102.055	---	---	14
N	2	30.023	216.098	1434.674	---	13
V	3	-82.814	315.166	1320.631	---	12
L	4	-263.578	428.250	1221.562	-123.999	11
S	5	-169.370	515.282	1108.478	-92.189	10
P	6	-515.704	612.335	1021.446	-53.683	9
H	7	-37.495	749.394	924.393	-30.101	8
T	8	-38.075	850.442	787.335	172.171	7
S	9	39.631	937.474	686.287	87.282	6
G	10	---	994.495	599.255	---	5
S#	11	---	1161.494	542.233	---	4
I	12	45.378	1274.578	375.235	217.594	3
S	13	---	1361.610	262.151	---	2
R	14	---	---	175.119	---	1

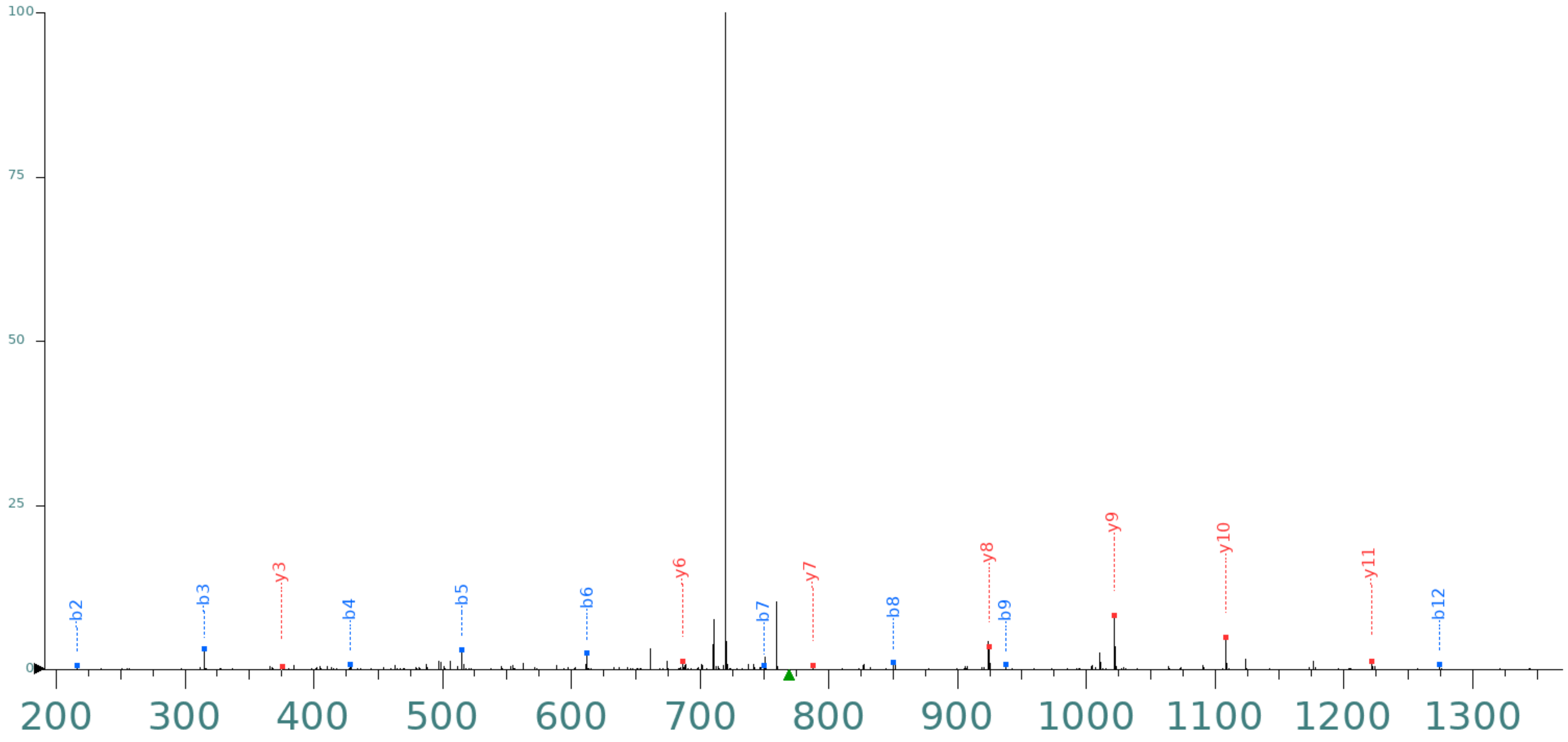

Predicted Fragmentation Pattern

Seq	#	b: Δ Error	b	y	y: Δ Error	+1
S	1	---	88.039	---	---	20
D	2	---	203.066	2151.890	---	19
T	3	---	304.114	2036.863	---	18
G	4	---	361.135	1935.815	---	17
S#	5	---	528.134	1878.794	---	16
Q	6	---	656.192	1711.796	---	15
Y	7	196.149	819.256	1583.737	184.682	14
I	8	218.214	932.340	1420.674	96.853	13
E	9	-9.304	1061.382	1307.590	99.502	12
D	10	251.504	1176.409	1178.547	172.309	11
S	11	141.622	1263.441	1063.520	49.076	10
I	12	149.613	1376.525	976.488	205.844	9
S	13	190.546	1463.557	863.404	53.668	8
Q	14	176.455	1591.616	776.372	138.344	7
G	15	---	1648.637	648.313	27.087	6
A	16	-2.964	1719.675	591.292	818.859	5
V	17	138.163	1818.743	520.255	511.766	4
C	18	---	1978.774	421.186	306.374	3
N	19	---	2092.817	261.156	---	2
K	20	---	---	147.113	---	1

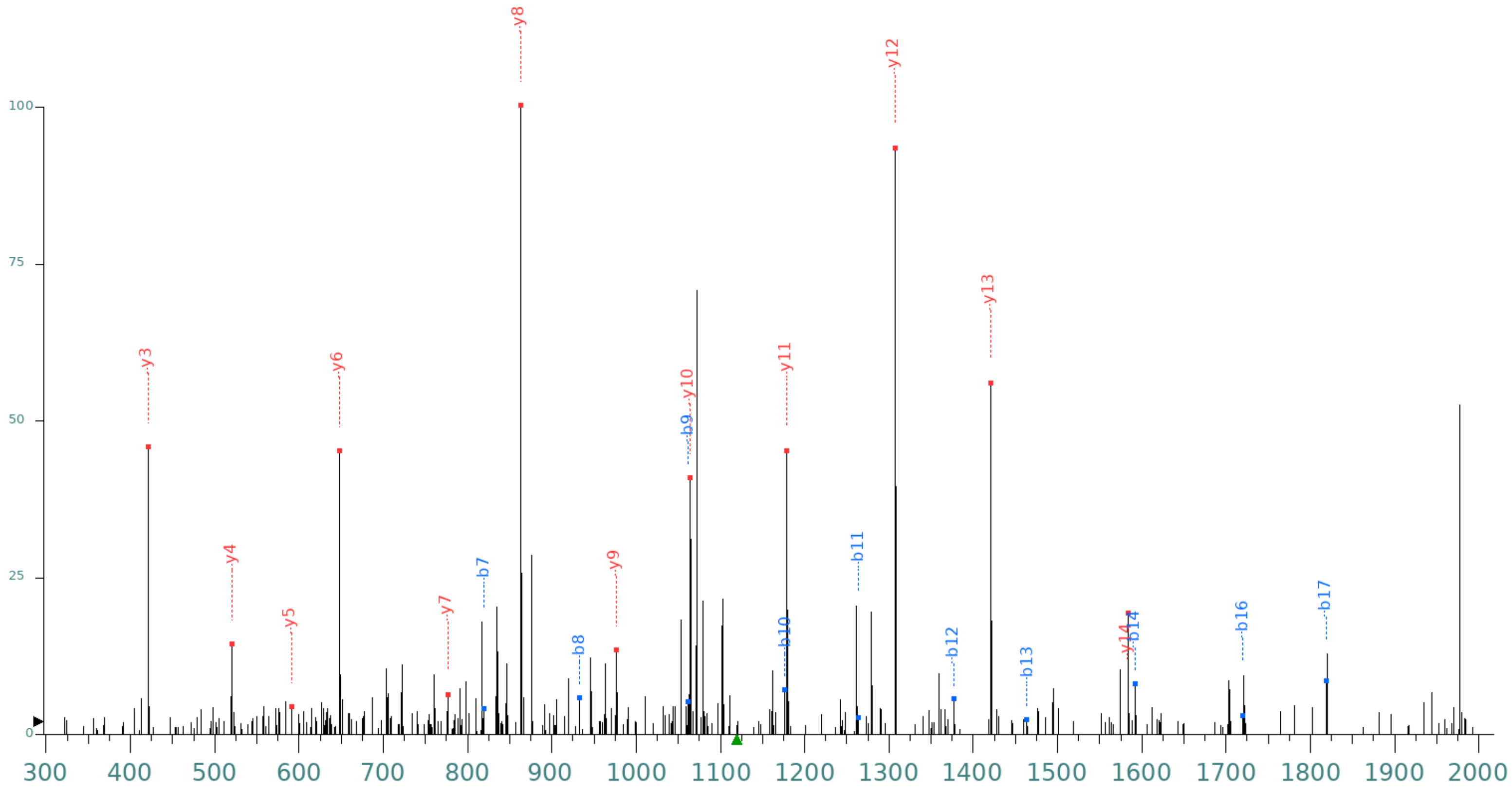

Predicted Fragmentation Pattern

Seq	#	b: Δ Error	b	y	y: Δ Error	+1
S	1	---	88.039	---	---	20
D	2	---	203.066	2151.890	---	19
T#	3	---	384.080	2036.863	---	18
G	4	---	441.102	1855.849	---	17
S	5	-69.500	**528.134**	1798.828	---	16
Q	6	---	656.192	1711.796	---	15
Y	7	156.528	**819.256**	1583.737	---	14
I	8	---	932.340	**1420.674**	96.079	13
E	9	-415.234	**1061.382**	**1307.590**	43.590	12
D	10	107.114	**1176.409**	**1178.547**	169.617	11
S	11	---	1263.441	**1063.520**	84.538	10
I	12	204.220	**1376.525**	**976.488**	152.109	9
S	13	---	1463.557	**863.404**	75.156	8
Q	14	260.247	**1591.616**	**776.372**	138.972	7
G	15	---	1648.637	**648.313**	-84.575	6
A	16	---	1719.675	591.292	---	5
V	17	---	1818.743	**520.255**	254.451	4
C	18	194.145	**1978.774**	**421.186**	-146.985	3
N	19	---	2092.817	261.156	---	2
K	20	---	---	147.113	---	1

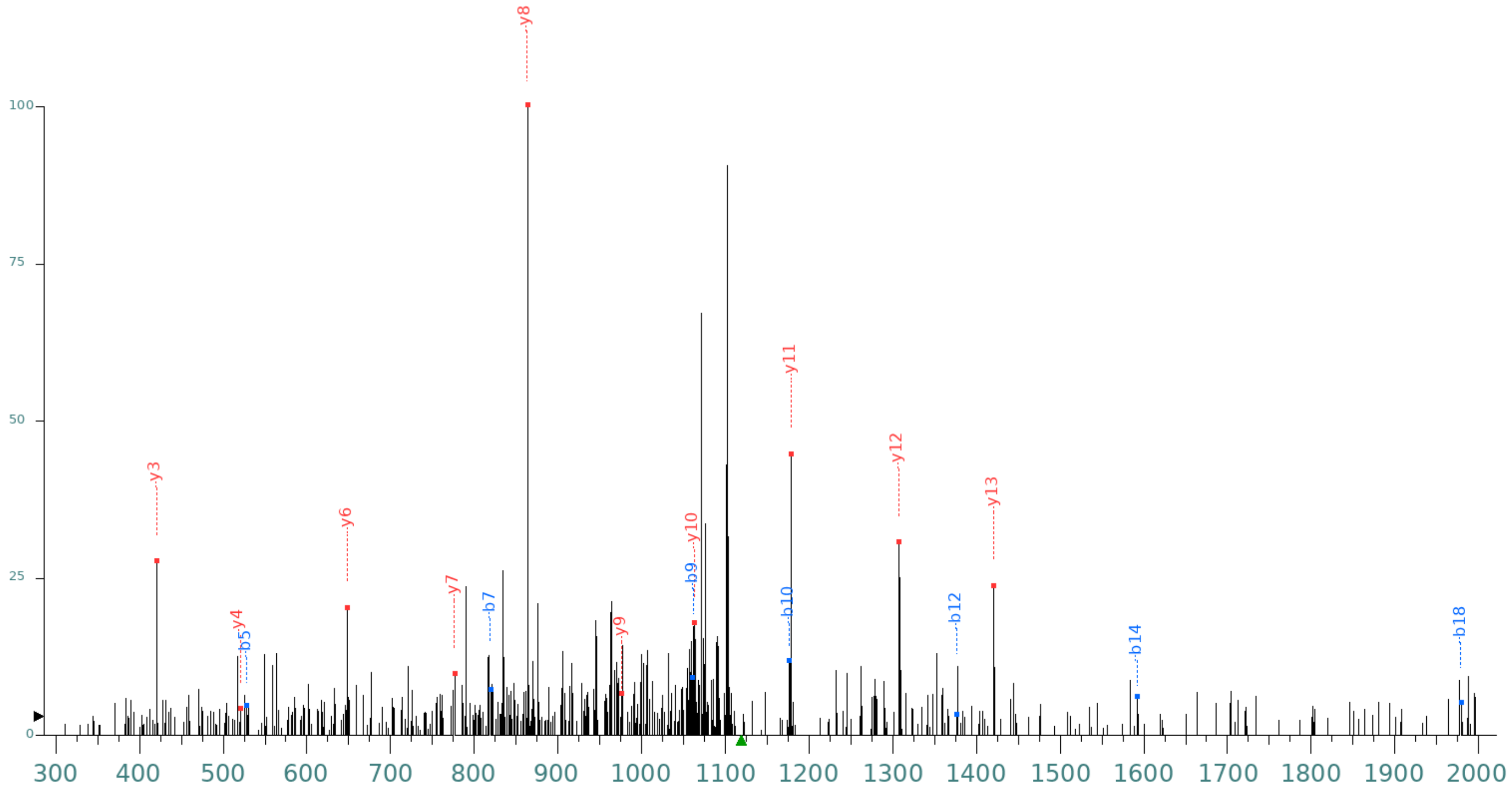

Predicted Fragmentation Pattern

+1

Seq	#	b: Δ Error	b	y	y: Δ Error	+1
K	1	---	129.102	---	---	15
T	2	---	230.150	1535.721	---	14
N	3	---	344.193	1434.674	---	13
V	4	151.929	**443.261**	1320.631	---	12
L	5	---	556.345	1221.562	---	11
S	6	213.829	**643.377**	**1108.478**	74.212	10
P	7	-35.118	**740.430**	**1021.446**	-61.094	9
H	8	100.275	**877.489**	**924.393**	197.193	8
T	9	161.748	**978.537**	**787.335**	27.390	7
S	10	---	1065.569	**686.287**	110.845	6
G	11	---	1122.590	**599.255**	-41.328	5
S#	12	---	1289.589	**542.233**	286.042	4
I	13	---	1402.673	**375.235**	-445.309	3
S	14	---	1489.705	262.151	---	2
R	15	---	---	**175.119**	216.248	1

+2

Seq	#	b: Δ Error	b	y	y: Δ Error	+1
K	1	---	65.055	---	---	15
T	2	---	115.579	**768.364**	-61.090	14
N	3	---	172.600	**717.840**	-55.051	13
V	4	---	222.134	**660.819**	222.936	12
L	5	---	278.676	**611.285**	233.357	11
S	6	---	322.192	554.743	---	10
P	7	---	370.719	**511.227**	-370.996	9
H	8	-170.485	**439.248**	**462.700**	-714.153	8
T	9	-120.434	**489.772**	394.171	---	7
S	10	184.155	**533.288**	343.647	---	6
G	11	---	561.799	300.131	---	5
S#	12	---	645.298	271.620	---	4
I	13	-349.955	**701.840**	188.121	---	3
S	14	-132.600	**745.356**	131.579	---	2
R	15	---	---	88.063	---	1

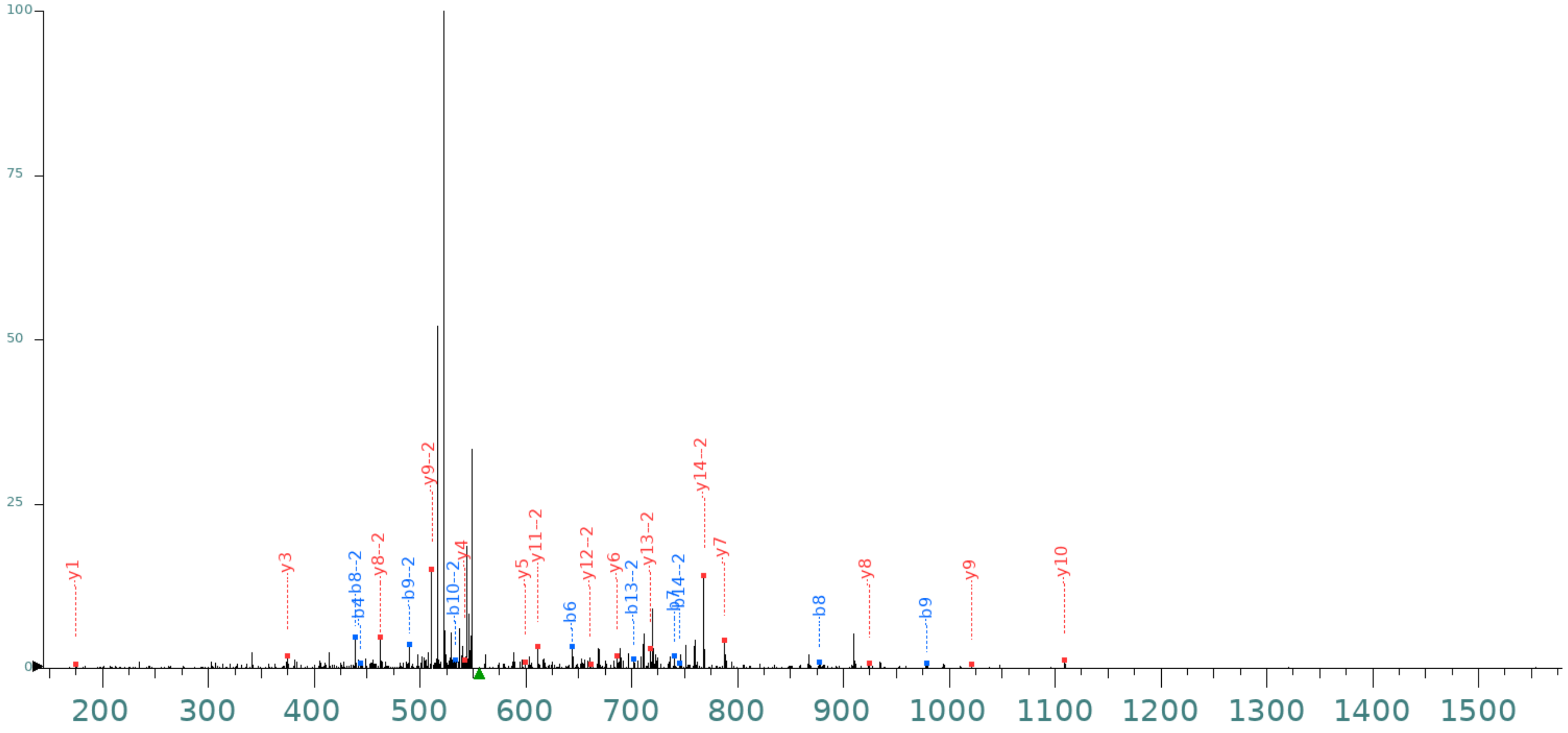

Predicted Fragmentation Pattern

+1

Seq	#	b: Δ Error	b	y	y: Δ Error	+1
S	1	---	88.039	---	---	20
D	2	---	203.066	2151.890	---	19
T	3	---	304.114	2036.863	---	18
G	4	---	361.135	1935.815	---	17
S	5	33.096	448.167	1878.794	---	16
Q	6	-228.390	576.226	1791.762	---	15
Y	7	120.416	739.289	1663.703	---	14
I	8	-111.259	852.373	1500.640	---	13
E	9	10.670	981.416	1387.556	---	12
D	10	---	1096.443	1258.513	---	11
S	11	-57.023	1183.475	1143.486	---	10
I	12	---	1296.559	1056.454	---	9
S#	13	---	1463.557	943.370	71.803	8
Q	14	---	1591.616	776.372	-21.778	7
G	15	---	1648.637	648.313	-84.387	6
A	16	---	1719.675	591.292	100.156	5
V	17	---	1818.743	520.255	-297.671	4
C	18	---	1978.774	421.186	-26.977	3
N	19	---	2092.817	261.156	---	2
K	20	---	---	147.113	---	1

+2

Seq	#	b: Δ Error	b	y	y: Δ Error	+1
S	1	---	44.523	---	---	20
D	2	---	102.037	1076.449	28.766	19
T	3	---	152.561	1018.935	404.950	18
G	4	---	181.071	968.411	-48.491	17
S	5	---	224.587	939.901	246.020	16
Q	6	---	288.617	896.385	-277.351	15
Y	7	---	370.148	832.355	426.055	14
I	8	---	426.690	750.824	---	13
E	9	44.192	491.212	694.282	-198.001	12
D	10	---	548.725	629.760	60.869	11
S	11	---	592.241	572.247	-505.092	10
I	12	---	648.783	528.731	180.416	9
S#	13	560.490	732.282	472.189	---	8
Q	14	550.830	796.312	388.690	---	7
G	15	-56.487	824.822	324.660	-1323.697	6
A	16	-224.256	860.341	296.150	---	5
V	17	-142.218	909.875	260.631	---	4
C	18	449.296	989.890	211.097	---	3
N	19	458.348	1046.912	131.082	---	2
K	20	---	---	74.060	---	1

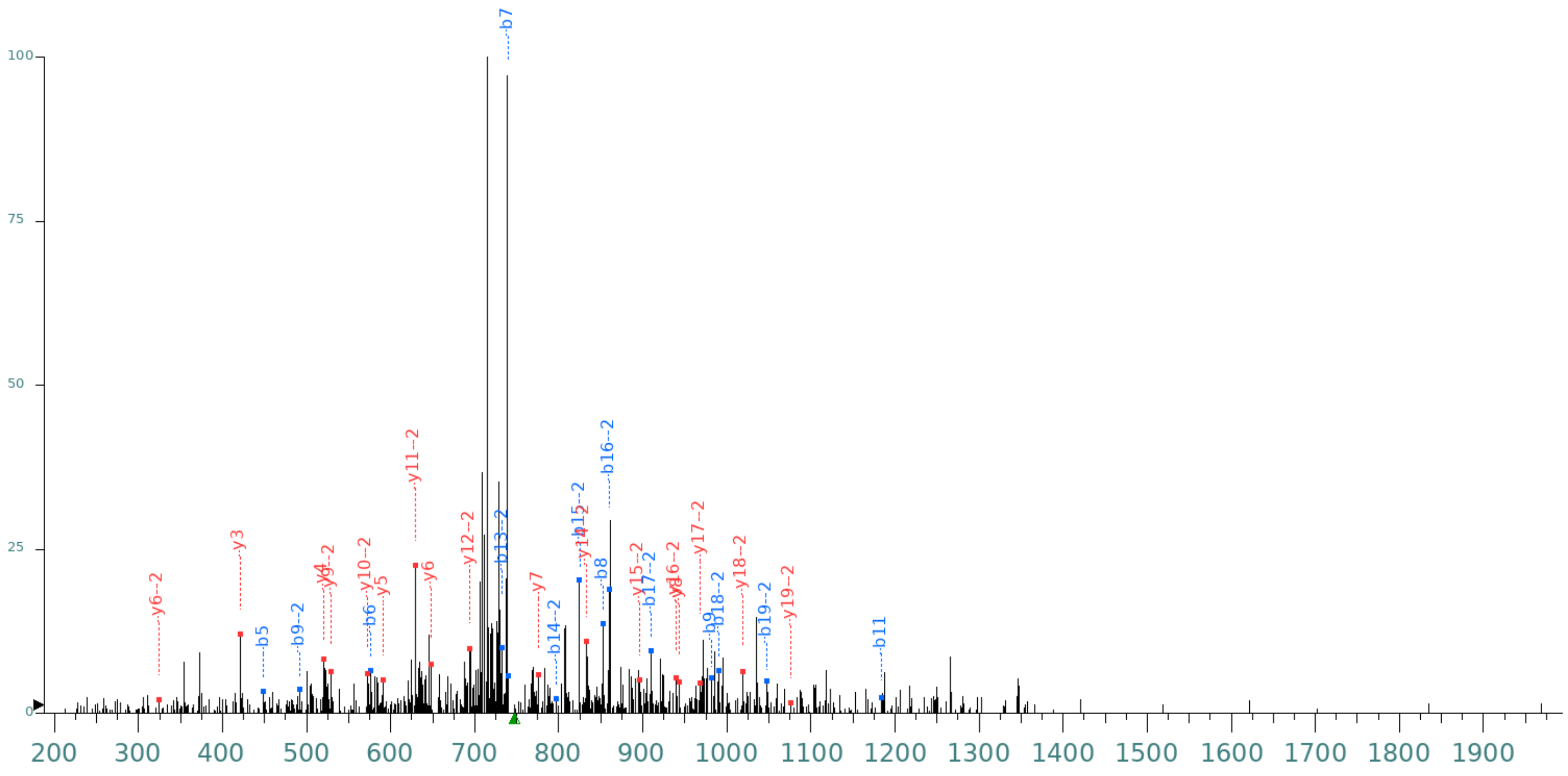

Predicted Fragmentation Pattern

+1

Seq	#	b: Δ Error	b	y	y: Δ Error	+1
R	1	---	157.108	---	---	12
P	2	336.377	**254.161**	1372.639	---	11
S#	3	---	421.160	1275.586	---	10
R	4	-517.618	**577.261**	1108.587	---	9
I	5	138.584	**690.345**	952.486	---	8
P	6	---	787.397	**839.402**	54.122	7
S	7	166.197	**874.429**	**742.349**	174.097	6
T	8	---	975.477	655.317	---	5
V	9	---	1074.546	**554.270**	823.860	4
L	10	---	1187.630	**455.201**	-234.612	3
S#	11	---	1354.628	**342.117**	340.007	2
R	12	---	---	175.119	---	1

+2

Seq	#	b: Δ Error	b	y	y: Δ Error	+1
R	1	---	79.058	---	---	12
P	2	---	127.584	**686.823**	-663.172	11
S#	3	360.426	**211.083**	**638.297**	44.039	10
R	4	-407.711	**289.134**	**554.797**	-127.178	9
I	5	274.706	**345.676**	476.747	---	8
P	6	318.620	**394.202**	**420.205**	-25.408	7
S	7	---	437.718	371.678	---	6
T	8	136.185	**488.242**	328.162	---	5
V	9	---	537.776	277.639	---	4
L	10	-0.674	**594.318**	**228.104**	-85.838	3
S#	11	---	677.818	171.562	---	2
R	12	---	---	88.063	---	1

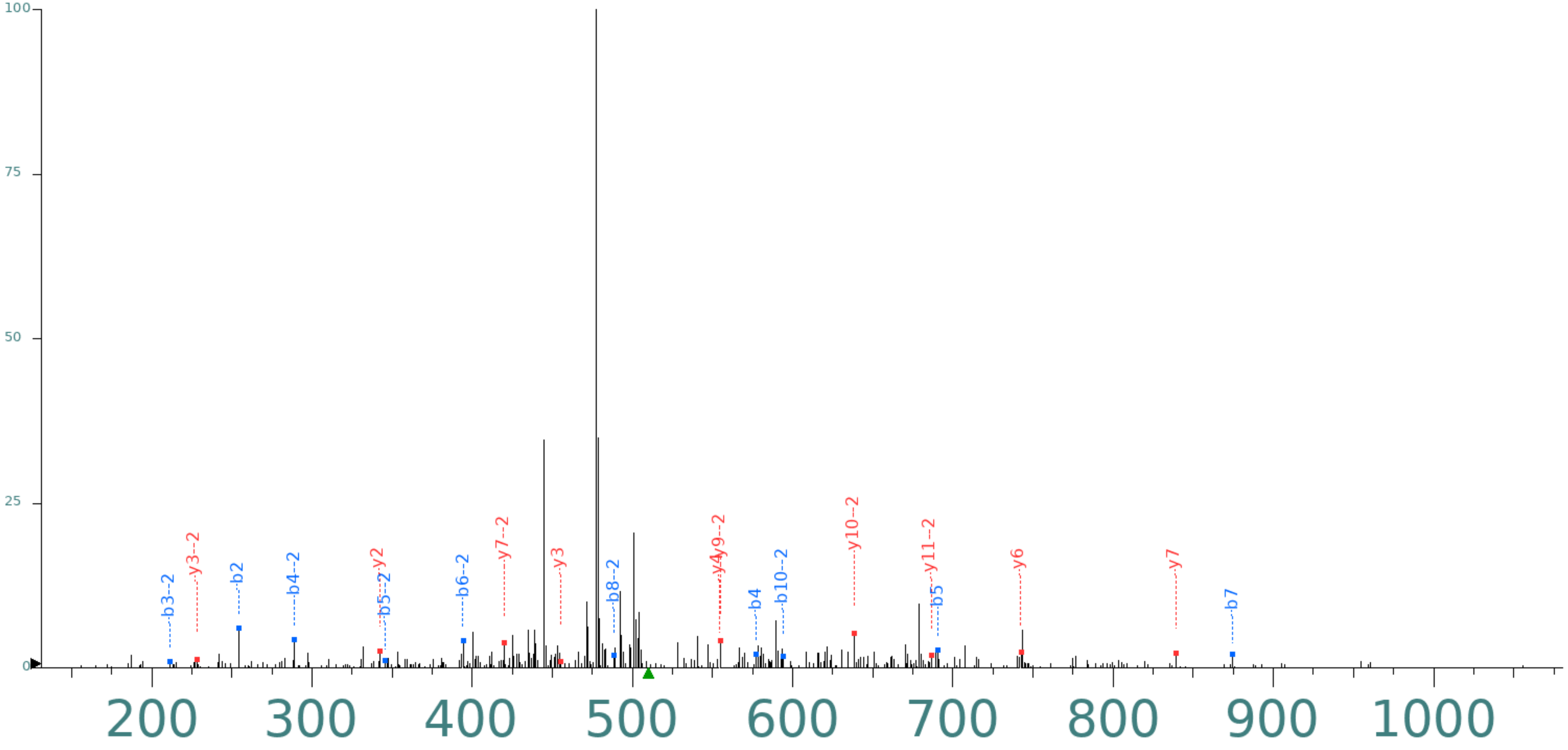

Predicted Fragmentation Pattern

Seq	#	b: Δ Error	b	y	y: Δ Error	+1
I	1	---	114.091	---	---	8
P	2	-593.376	211.144	839.402	-46.002	7
S#	3	318.178	378.142	742.349	-79.195	6
T	4	---	479.190	575.351	-58.247	5
V	5	308.726	578.259	474.303	444.486	4
L	6	-46.128	691.343	375.235	-210.520	3
S	7	---	778.375	262.151	219.116	2
R	8	---	---	175.119	-581.231	1

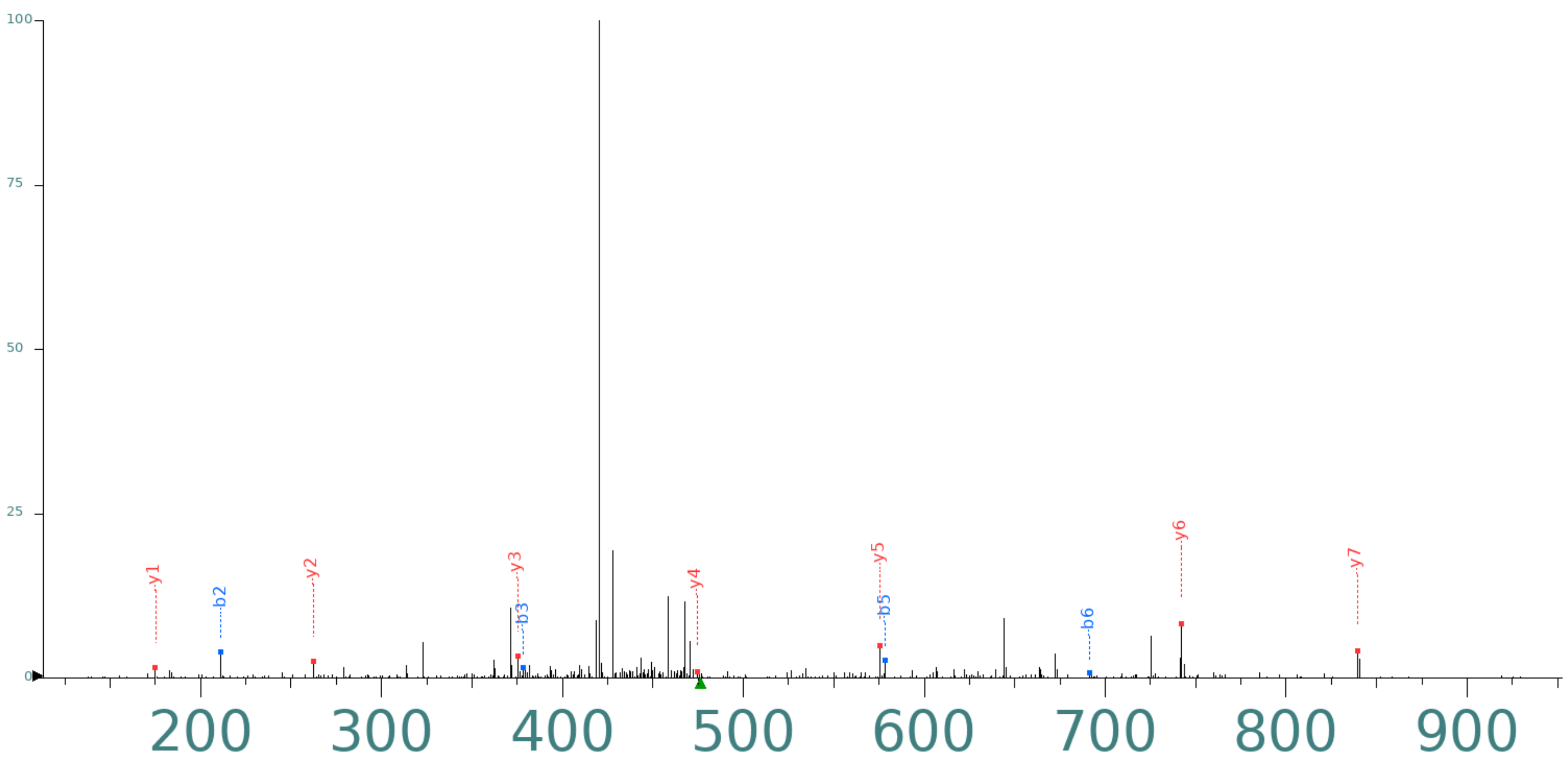

Predicted Fragmentation Pattern

+1

Seq	#	b: Δ Error	b	y	y: Δ Error	+1
R	1	---	157.108	---	---	12
P	2	-489.423	254.161	1372.639	---	11
S#	3	1168.527	421.160	1275.586	---	10
R	4	---	577.261	1108.587	---	9
I	5	132.397	690.345	952.486	---	8
P	6	31.253	787.397	839.402	81.459	7
S	7	---	874.429	742.349	333.603	6
T#	8	---	1055.443	655.317	367.725	5
V	9	---	1154.512	474.303	-195.106	4
L	10	---	1267.596	375.235	563.948	3
S	11	---	1354.628	262.151	293.116	2
R	12	---	---	175.119	---	1

+2

Seq	#	b: Δ Error	b	y	y: Δ Error	+1
R	1	---	79.058	---	---	12
P	2	---	127.584	686.823	---	11
S#	3	---	211.083	638.297	48.150	10
R	4	---	289.134	554.797	---	9
I	5	526.201	345.676	476.747	474.799	8
P	6	83.215	394.202	420.205	369.104	7
S	7	621.112	437.718	371.678	---	6
T#	8	688.346	528.225	328.162	---	5
V	9	165.714	577.760	237.655	---	4
L	10	254.185	634.302	188.121	---	3
S	11	-529.585	677.818	131.579	---	2
R	12	---	---	88.063	---	1

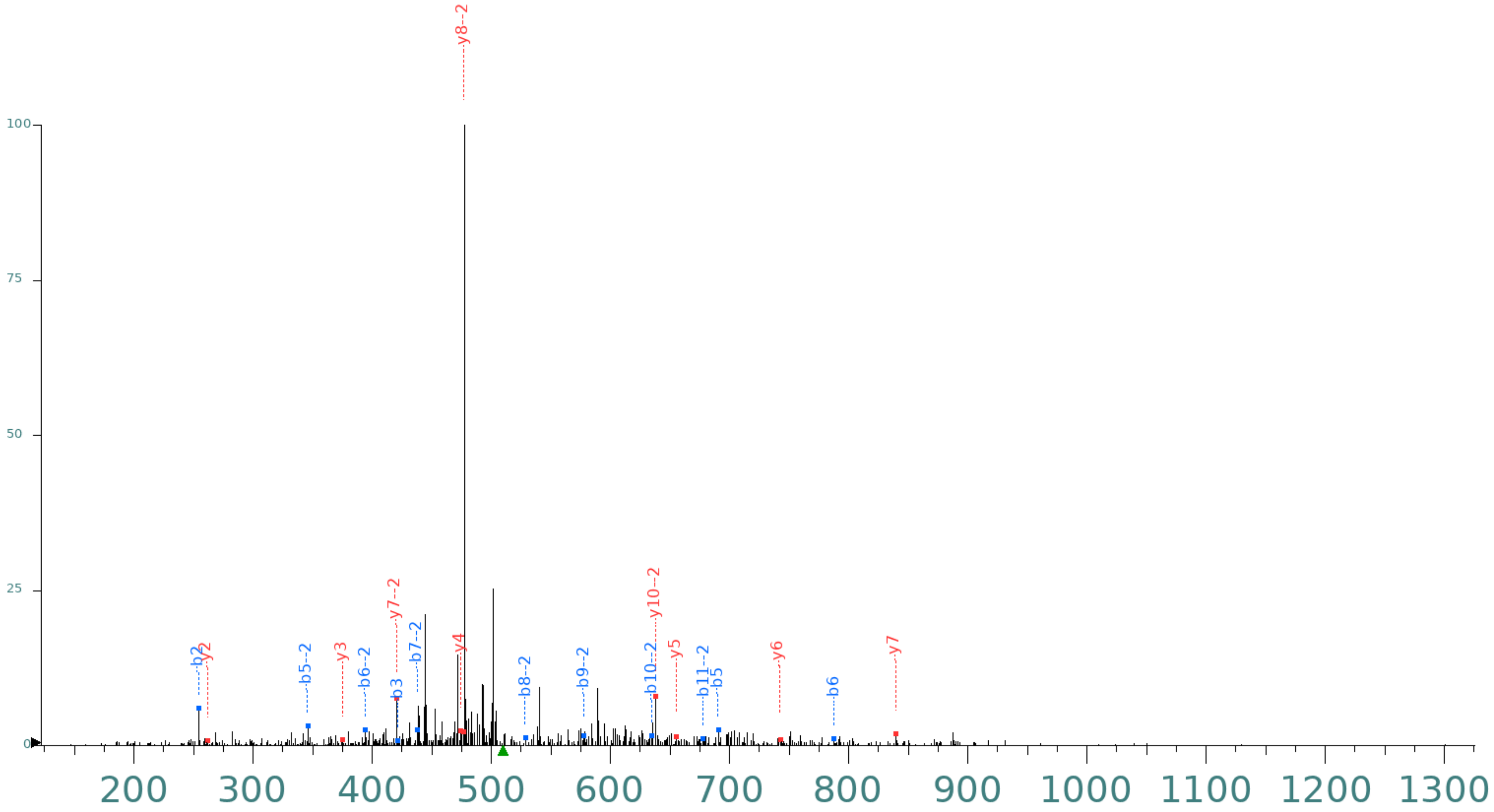

Predicted Fragmentation Pattern

Seq	#	b: Δ Error	b	y	y: Δ Error	+1
I	1	---	114.091	---	---	8
P	2	220.656	211.144	839.402	-71.818	7
S#	3	128.285	378.142	742.349	111.218	6
T	4	---	479.190	575.351	270.330	5
V	5	348.176	578.259	474.303	84.362	4
L	6	---	691.343	375.235	43.188	3
S	7	---	778.375	262.151	345.003	2
R	8	---	---	175.119	---	1

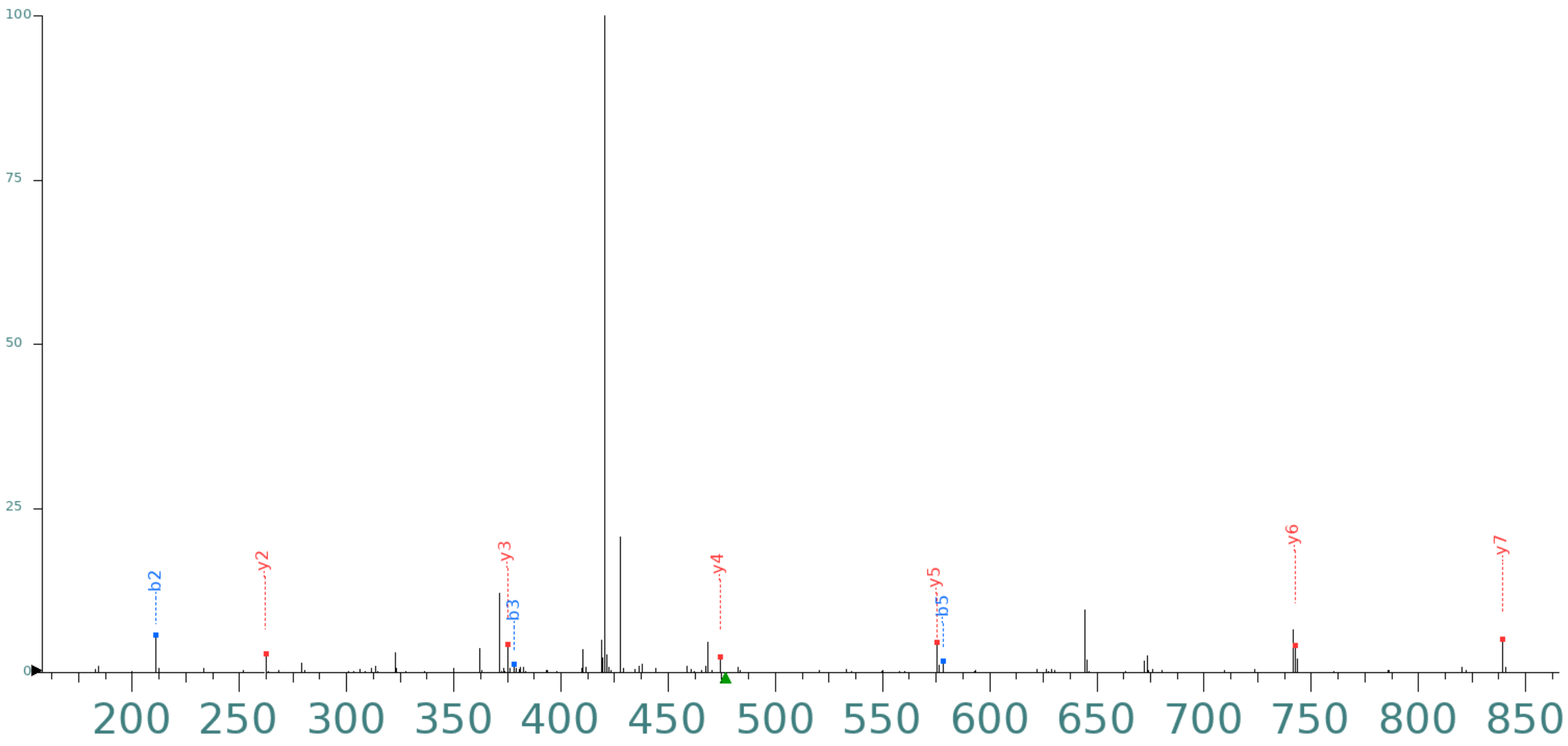

Predicted Fragmentation Pattern

+1

Seq	#	b: Δ Error	b	y	y: Δ Error	+1
R	1	---	157.108	---	---	12
P	2	208.210	**254.161**	1292.672	---	11
S#	3	---	421.160	1195.619	---	10
R	4	---	577.261	1028.621	---	9
I	5	-244.460	**690.345**	872.520	---	8
P	6	---	787.397	**759.436**	119.634	7
S	7	---	874.429	662.383	---	6
T	8	---	975.477	575.351	---	5
V	9	---	1074.546	**474.303**	-184.357	4
L	10	---	1187.630	**375.235**	665.241	3
S	11	---	1274.662	262.151	---	2
R	12	---	---	175.119	---	1

+2

Seq	#	b: Δ Error	b	y	y: Δ Error	+1
R	1	---	79.058	---	---	12
P	2	---	127.584	**646.840**	127.982	11
S#	3	---	211.083	**598.313**	721.698	10
R	4	---	289.134	**514.814**	-547.407	9
I	5	-519.248	**345.676**	436.764	---	8
P	6	---	394.202	**380.222**	-316.695	7
S	7	---	437.718	**331.695**	-169.870	6
T	8	---	488.242	288.179	---	5
V	9	-43.006	**537.776**	237.655	---	4
L	10	---	594.318	188.121	---	3
S	11	---	637.834	131.579	---	2
R	12	---	---	88.063	---	1

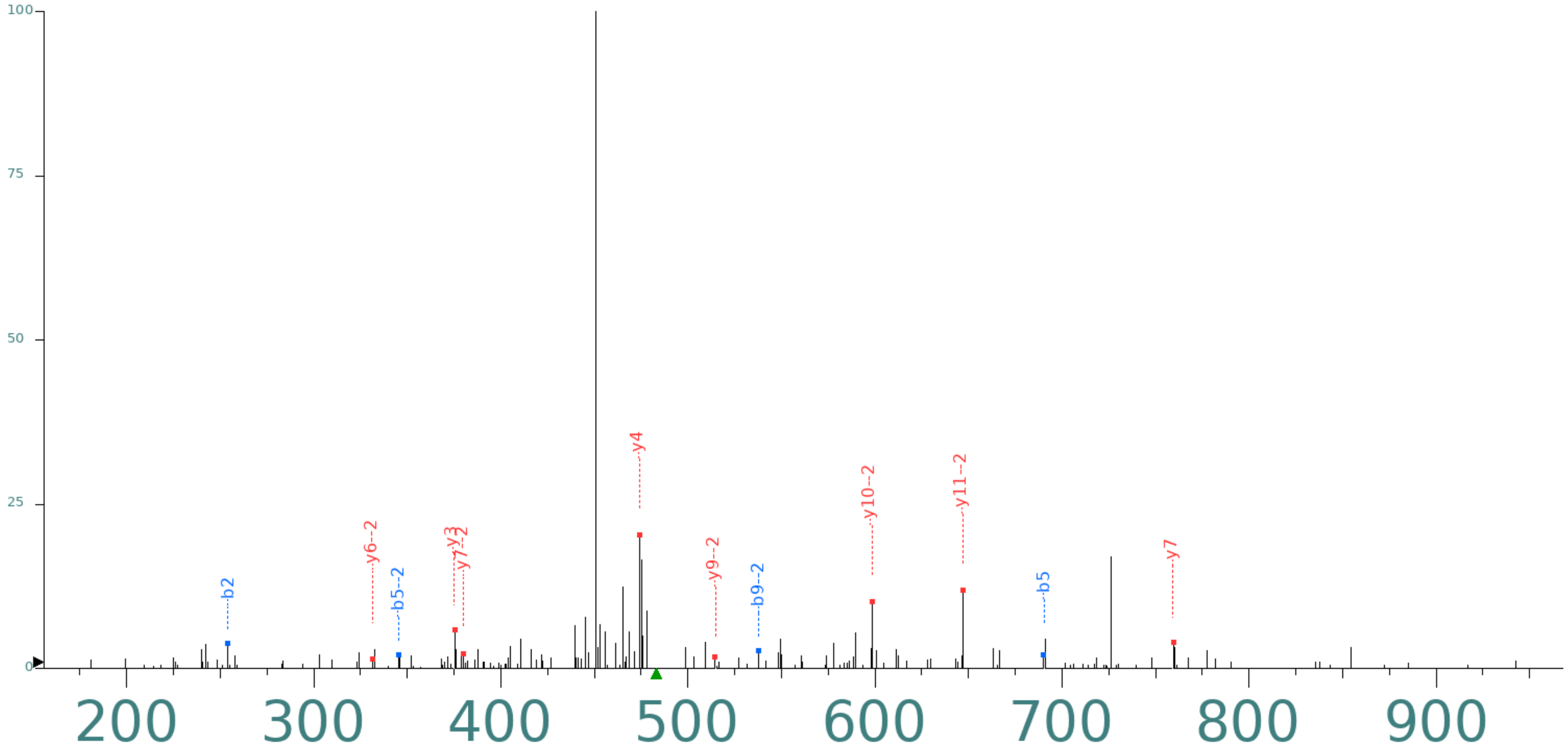

Predicted Fragmentation Pattern

+1

Seq	#	b: Δ Error	b	y	y: Δ Error	+1
R	1	---	157.108	---	---	12
P	2	-826.788	**254.161**	1372.639	---	11
$S^{\#}$	3	-105.718	**421.160**	1275.586	---	10
R	4	643.601	**577.261**	1108.587	---	9
I	5	-153.182	**690.345**	952.486	---	8
P	6	---	787.397	**839.402**	28.748	7
S	7	286.966	**874.429**	742.349	---	6
T	8	---	975.477	655.317	---	5
V	9	---	1074.546	554.270	---	4
L	10	---	1187.630	455.201	---	3
$S^{\#}$	11	---	1354.628	342.117	---	2
R	12	---	---	175.119	---	1

+2

Seq	#	b: Δ Error	b	y	y: Δ Error	+1
R	1	---	79.058	---	---	12
P	2	---	127.584	**686.823**	-602.400	11
$S^{\#}$	3	-2064.985	**211.083**	**638.297**	-436.459	10
R	4	559.386	**289.134**	554.797	---	9
I	5	193.876	**345.676**	476.747	---	8
P	6	183.519	**394.202**	**420.205**	-232.298	7
S	7	-1075.379	**437.718**	371.678	---	6
T	8	-421.394	**488.242**	328.162	---	5
V	9	---	537.776	277.639	---	4
L	10	-841.654	**594.318**	228.104	---	3
$S^{\#}$	11	---	677.818	171.562	---	2
R	12	---	---	88.063	---	1

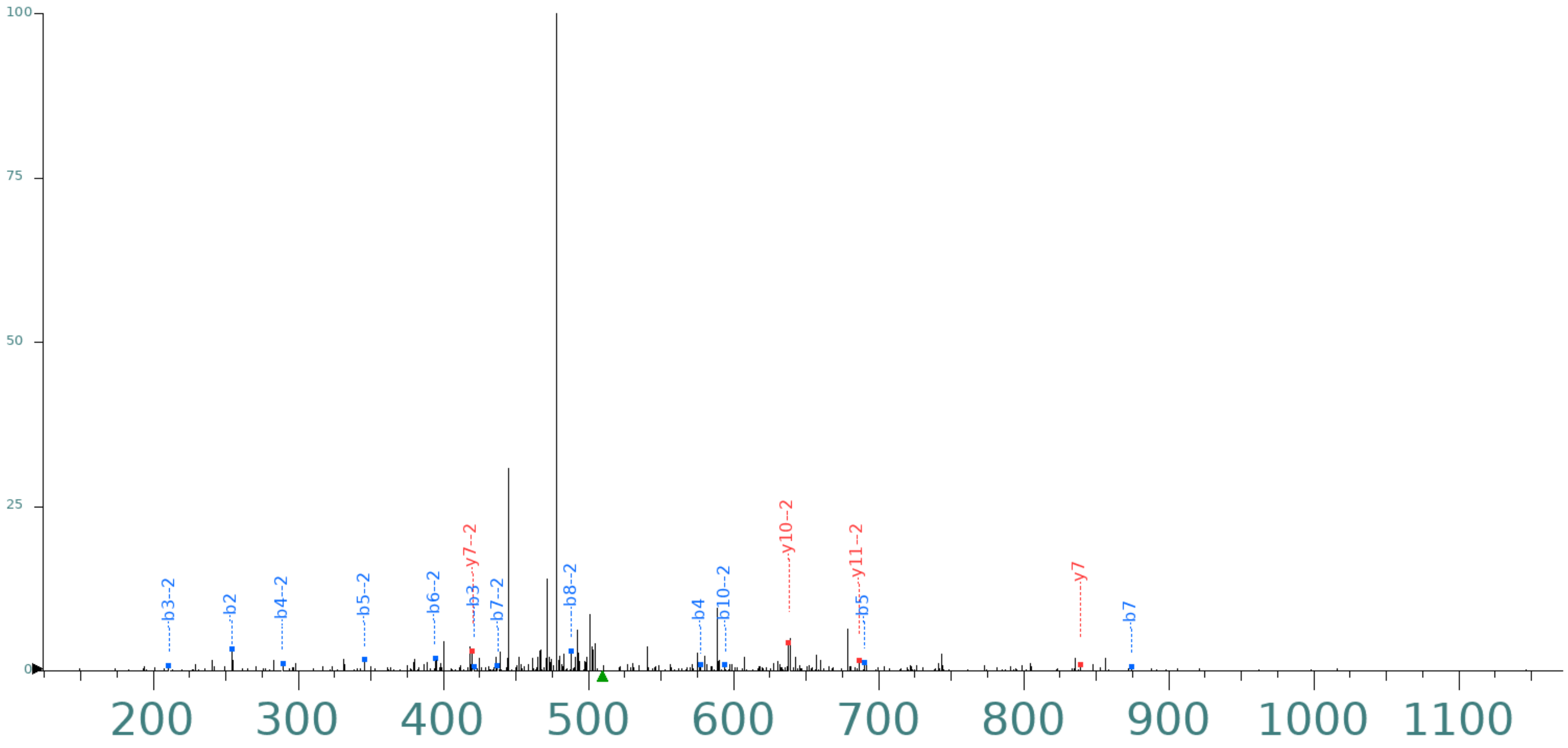

Predicted Fragmentation Pattern

+1

Seq	#	b: Δ Error	b	y	y: Δ Error	+1
R	1	---	157.108	---	---	12
P	2	-851.384	**254.161**	1372.639	---	11
S#	3	---	421.160	1275.586	---	10
R	4	385.351	**577.261**	1108.587	---	9
I	5	-32.123	**690.345**	952.486	---	8
P	6	---	787.397	**839.402**	-134.655	7
S	7	---	874.429	742.349	---	6
T	8	---	975.477	655.317	---	5
V	9	---	1074.546	**554.270**	858.599	4
L	10	---	1187.630	455.201	---	3
S#	11	---	1354.628	342.117	---	2
R	12	---	---	**175.119**	-807.919	1

+2

Seq	#	b: Δ Error	b	y	y: Δ Error	+1
R	1	---	79.058	---	---	12
P	2	---	127.584	686.823	---	11
S#	3	---	211.083	**638.297**	-202.035	10
R	4	---	289.134	**554.797**	-92.406	9
I	5	231.557	**345.676**	**476.747**	196.150	8
P	6	1131.083	**394.202**	**420.205**	477.659	7
S	7	-782.711	**437.718**	371.678	---	6
T	8	---	488.242	328.162	---	5
V	9	---	537.776	277.639	---	4
L	10	7.028	**594.318**	228.104	---	3
S#	11	---	677.818	171.562	---	2
R	12	---	---	88.063	---	1

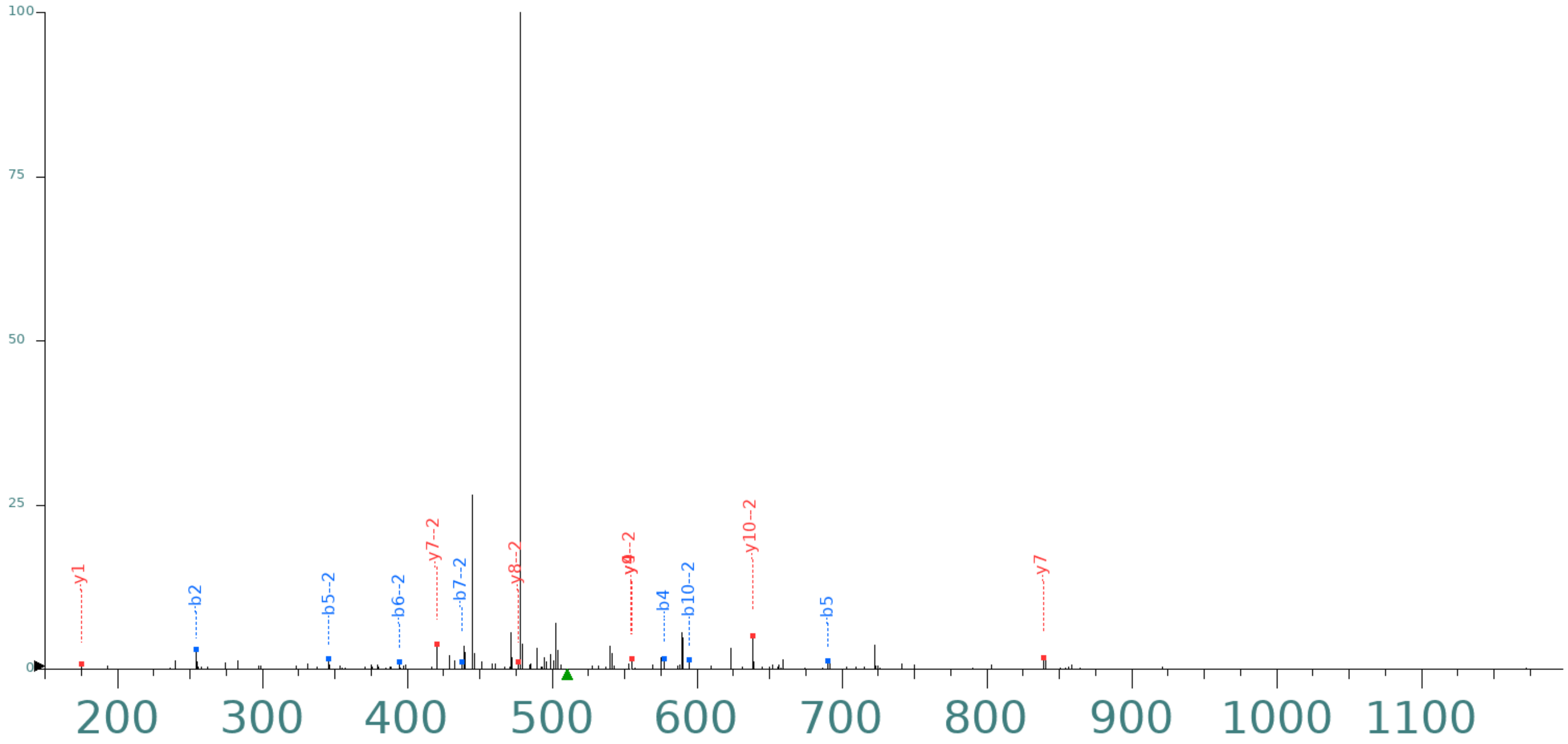

Predicted Fragmentation Pattern

+1

Seq	#	b: Δ Error	b	y	y: Δ Error	+1
R	1	---	157.108	---	---	12
P	2	-474.940	254.161	1372.639	---	11
S#	3	-144.277	421.160	1275.586	---	10
R	4	---	577.261	1108.587	---	9
I	5	77.416	690.345	952.486	---	8
P	6	---	787.397	839.402	133.728	7
S	7	270.294	874.429	742.349	---	6
T#	8	---	1055.443	655.317	726.845	5
V	9	---	1154.512	474.303	-105.772	4
L	10	---	1267.596	375.235	343.746	3
S	11	---	1354.628	262.151	---	2
R	12	---	---	175.119	-243.657	1

+2

Seq	#	b: Δ Error	b	y	y: Δ Error	+1
R	1	---	79.058	---	---	12
P	2	---	127.584	686.823	---	11
S#	3	---	211.083	638.297	-115.661	10
R	4	---	289.134	554.797	85.157	9
I	5	-482.573	345.676	476.747	-852.160	8
P	6	---	394.202	420.205	-238.983	7
S	7	215.683	437.718	371.678	---	6
T#	8	727.692	528.225	328.162	---	5
V	9	---	577.760	237.655	---	4
L	10	-149.915	634.302	188.121	---	3
S	11	---	677.818	131.579	---	2
R	12	---	---	88.063	---	1

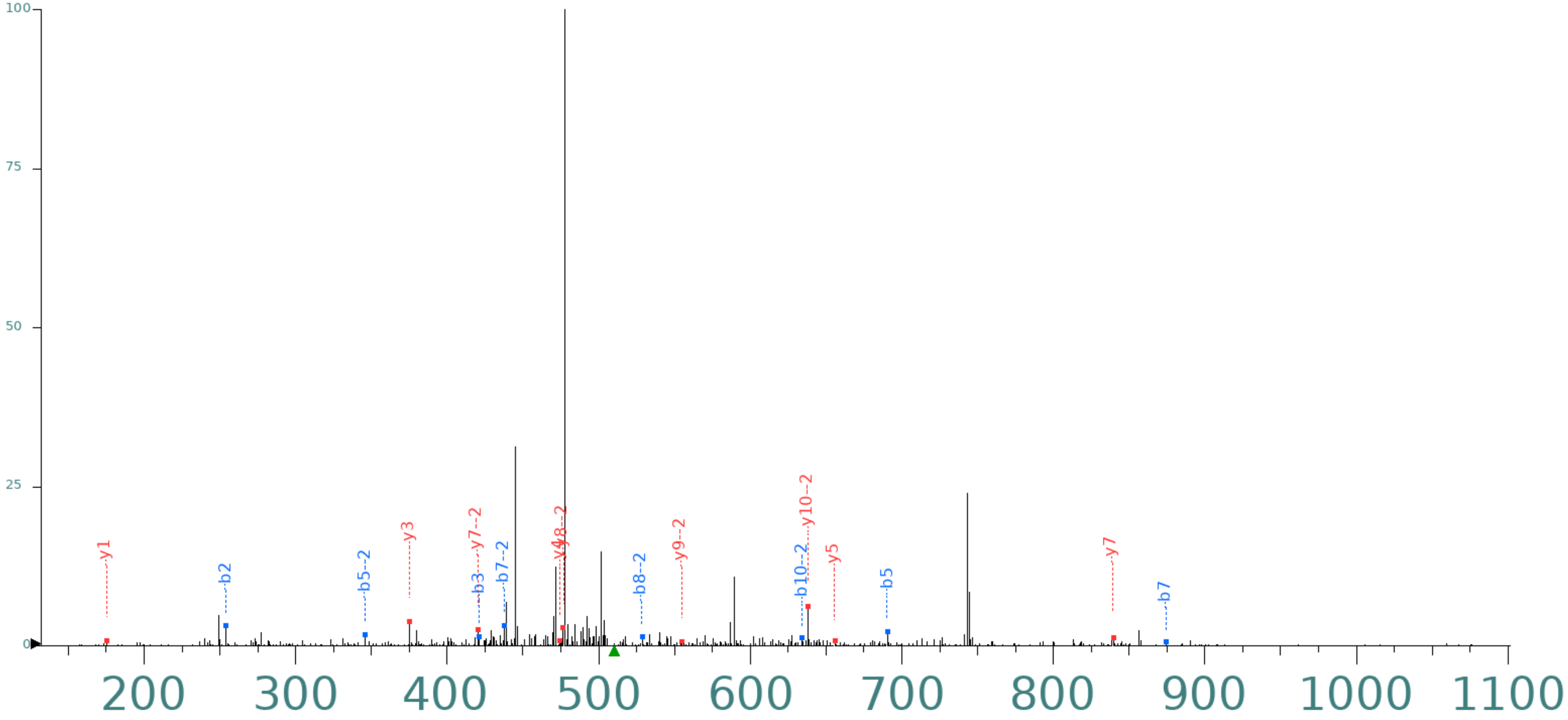

Predicted Fragmentation Pattern

Seq	#	b: Δ Error	b	y	y: Δ Error	+1
T	1	---	102.055	---	---	14
N	2	---	216.098	1514.640	---	13
V	3	---	315.166	1400.597	---	12
L	4	---	428.250	1301.529	-12.745	11
S#	5	-13.126	595.249	1188.445	186.265	10
P	6	---	692.301	1021.446	-70.177	9
H	7	---	829.360	924.393	451.167	8
T	8	---	930.408	787.335	-96.497	7
S	9	118.547	1017.440	686.287	211.577	6
G	10	-45.121	1074.462	599.255	123.251	5
S#	11	---	1241.460	542.233	---	4
I	12	337.531	1354.544	375.235	---	3
S	13	---	1441.576	262.151	---	2
R	14	---	---	175.119	---	1

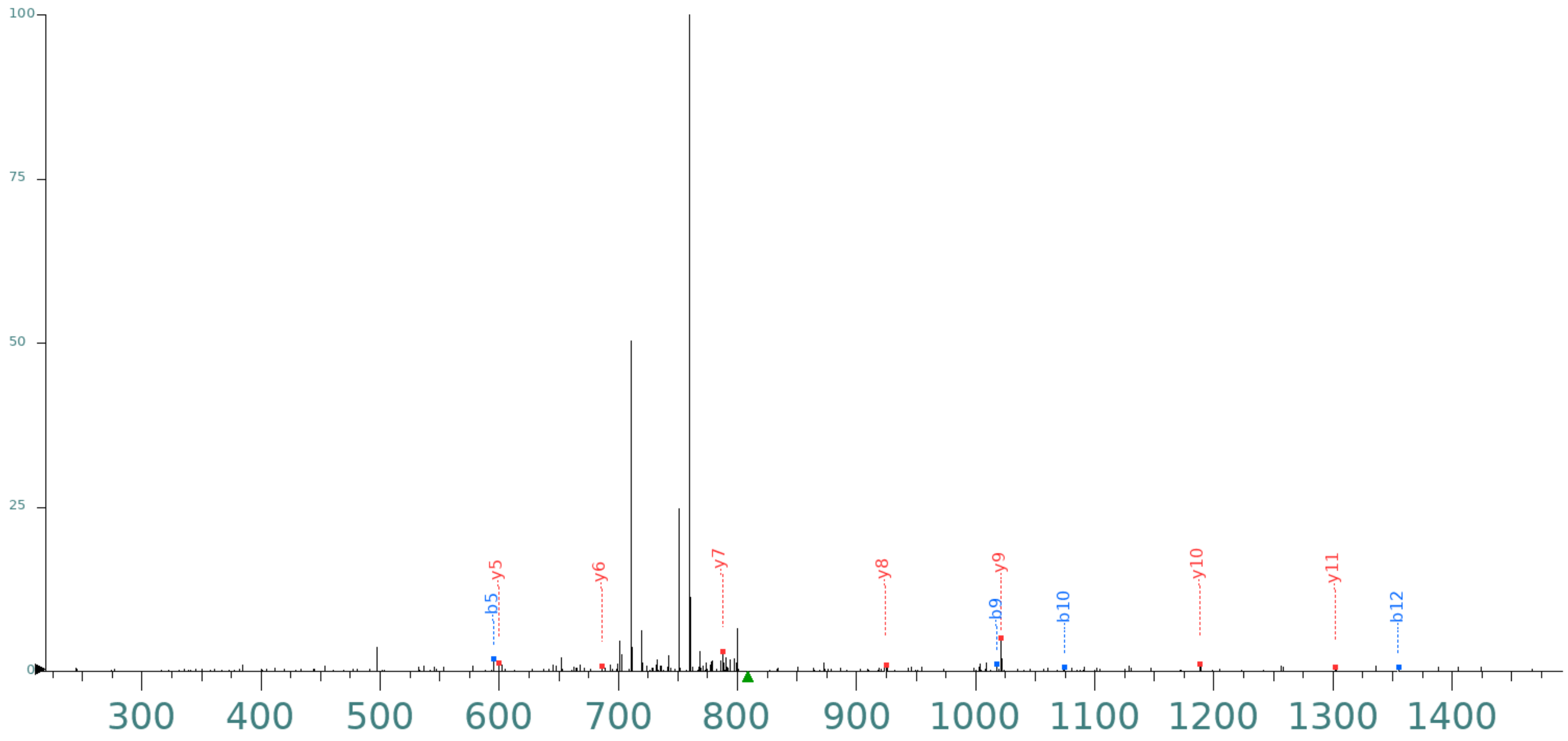

Predicted Fragmentation Pattern

Seq	#	b: Δ Error	b	y	y: Δ Error	+1
T	1	---	102.055	---	---	14
N	2	---	216.098	1434.674	---	13
V	3	-288.170	**315.166**	1320.631	---	12
L	4	---	428.250	**1221.562**	74.171	11
S#	5	---	595.249	**1108.478**	24.441	10
P	6	---	692.301	**941.480**	-115.020	9
H	7	---	829.360	**844.427**	-147.944	8
T	8	44.418	**930.408**	**707.368**	-138.706	7
S	9	-63.929	**1017.440**	**606.321**	61.186	6
G	10	---	1074.462	**519.289**	519.194	5
S	11	---	1161.494	**462.267**	78.722	4
I	12	---	1274.578	375.235	---	3
S	13	---	1361.610	262.151	---	2
R	14	---	---	175.119	---	1

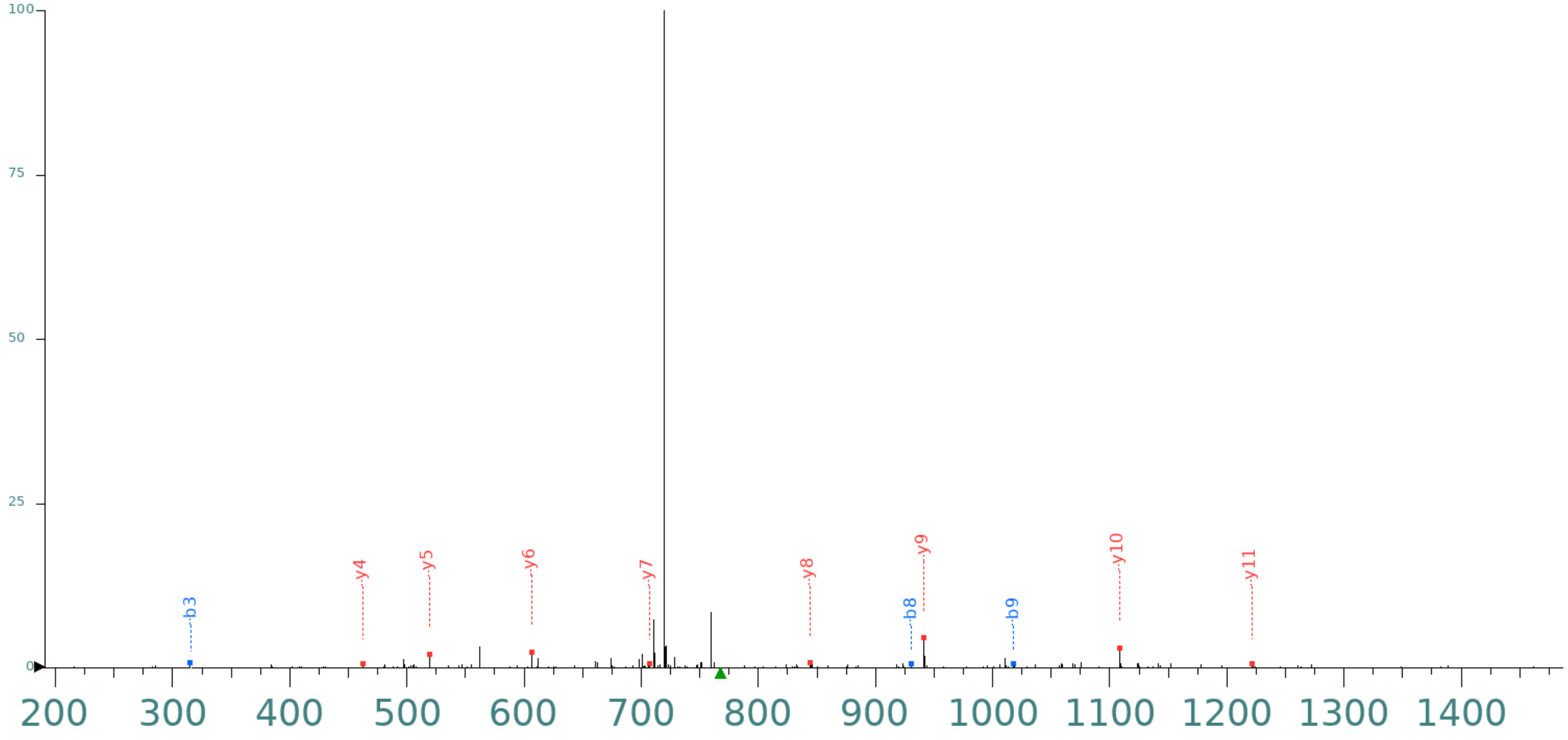

Predicted Fragmentation Pattern

+1

Seq	#	b: Δ Error	b	y	y: Δ Error	+1
R	1	---	157.108	---	---	12
P	2	-624.474	254.161	1372.639	---	11
S#	3	---	421.160	1275.586	---	10
R	4	---	577.261	1108.587	---	9
I	5	88.731	690.345	952.486	---	8
P	6	---	787.397	839.402	-1.573	7
S#	7	---	954.396	742.349	-53.293	6
T	8	---	1055.443	575.351	-150.877	5
V	9	---	1154.512	474.303	374.732	4
L	10	---	1267.596	375.235	96.939	3
S	11	---	1354.628	262.151	---	2
R	12	---	---	175.119	---	1

+2

Seq	#	b: Δ Error	b	y	y: Δ Error	+1
R	1	---	79.058	---	---	12
P	2	---	127.584	686.823	---	11
S#	3	-291.639	211.083	638.297	226.723	10
R	4	-379.613	289.134	554.797	---	9
I	5	624.789	345.676	476.747	---	8
P	6	---	394.202	420.205	32.402	7
S#	7	17.880	477.702	371.678	-775.643	6
T	8	---	528.225	288.179	---	5
V	9	570.549	577.760	237.655	---	4
L	10	-447.810	634.302	188.121	---	3
S	11	---	677.818	131.579	---	2
R	12	---	---	88.063	---	1

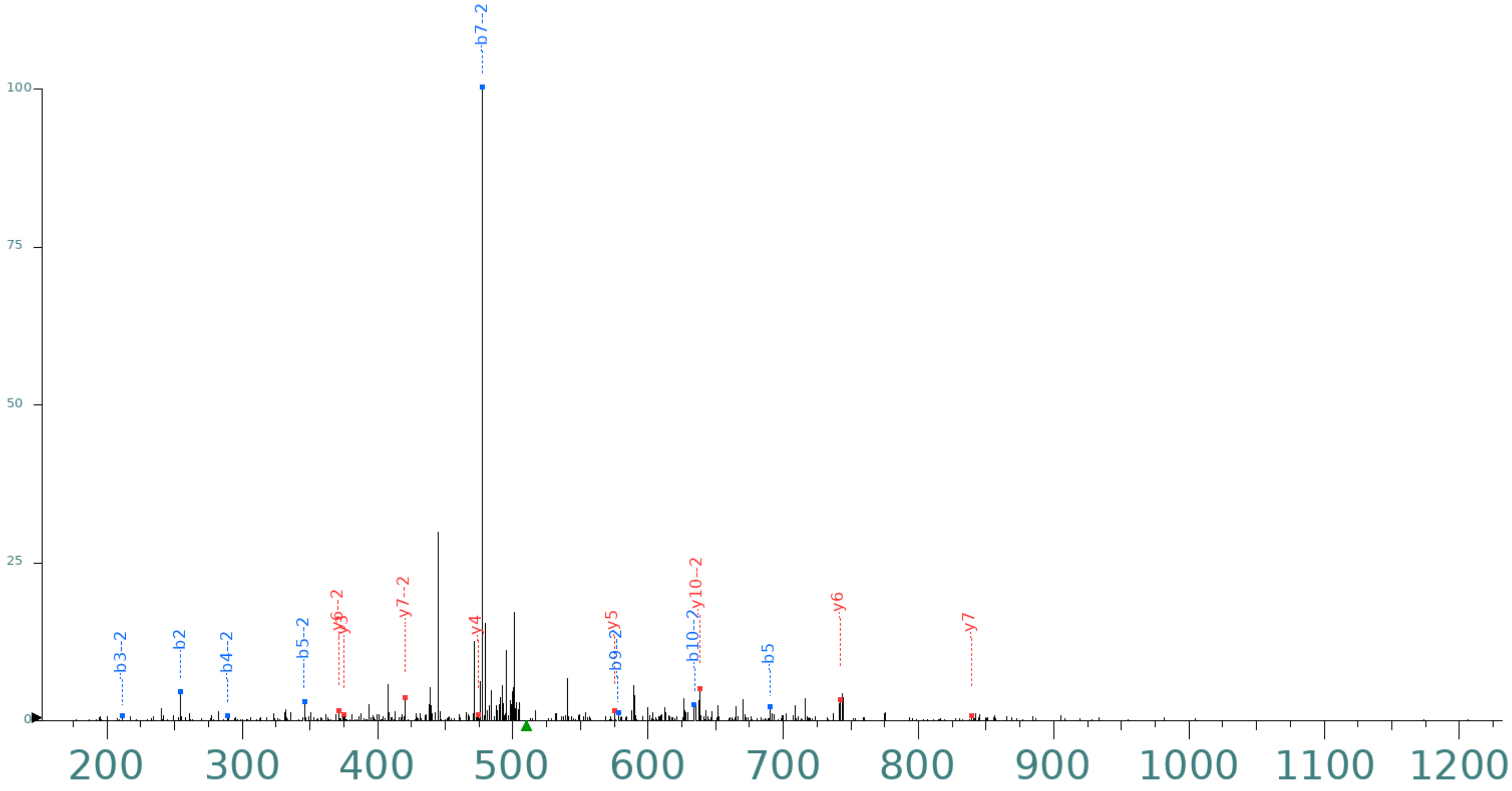

Predicted Fragmentation Pattern

+1

Seq	#	b: Δ Error	b	y	y: Δ Error	+1
R	1	-1459.088	157.108	---	---	12
P	2	-702.804	254.161	1372.639	---	11
S#	3	-335.375	421.160	1275.586	---	10
R	4	-345.019	577.261	1108.587	---	9
I	5	-15.235	690.345	952.486	---	8
P	6	264.505	787.397	839.402	118.898	7
S	7	158.103	874.429	742.349	37.561	6
T#	8	---	1055.443	655.317	-367.789	5
V	9	---	1154.512	474.303	-464.490	4
L	10	---	1267.596	375.235	-307.434	3
S	11	---	1354.628	262.151	---	2
R	12	---	---	175.119	-1225.703	1

+2

Seq	#	b: Δ Error	b	y	y: Δ Error	+1
R	1	---	79.058	---	---	12
P	2	---	127.584	686.823	-329.950	11
S#	3	-484.074	211.083	638.297	-568.454	10
R	4	706.650	289.134	554.797	---	9
I	5	201.994	345.676	476.747	297.623	8
P	6	473.948	394.202	420.205	-380.981	7
S	7	677.511	437.718	371.678	-1169.045	6
T#	8	86.233	528.225	328.162	-720.731	5
V	9	864.443	577.760	237.655	---	4
L	10	323.426	634.302	188.121	---	3
S	11	655.009	677.818	131.579	---	2
R	12	---	---	88.063	---	1

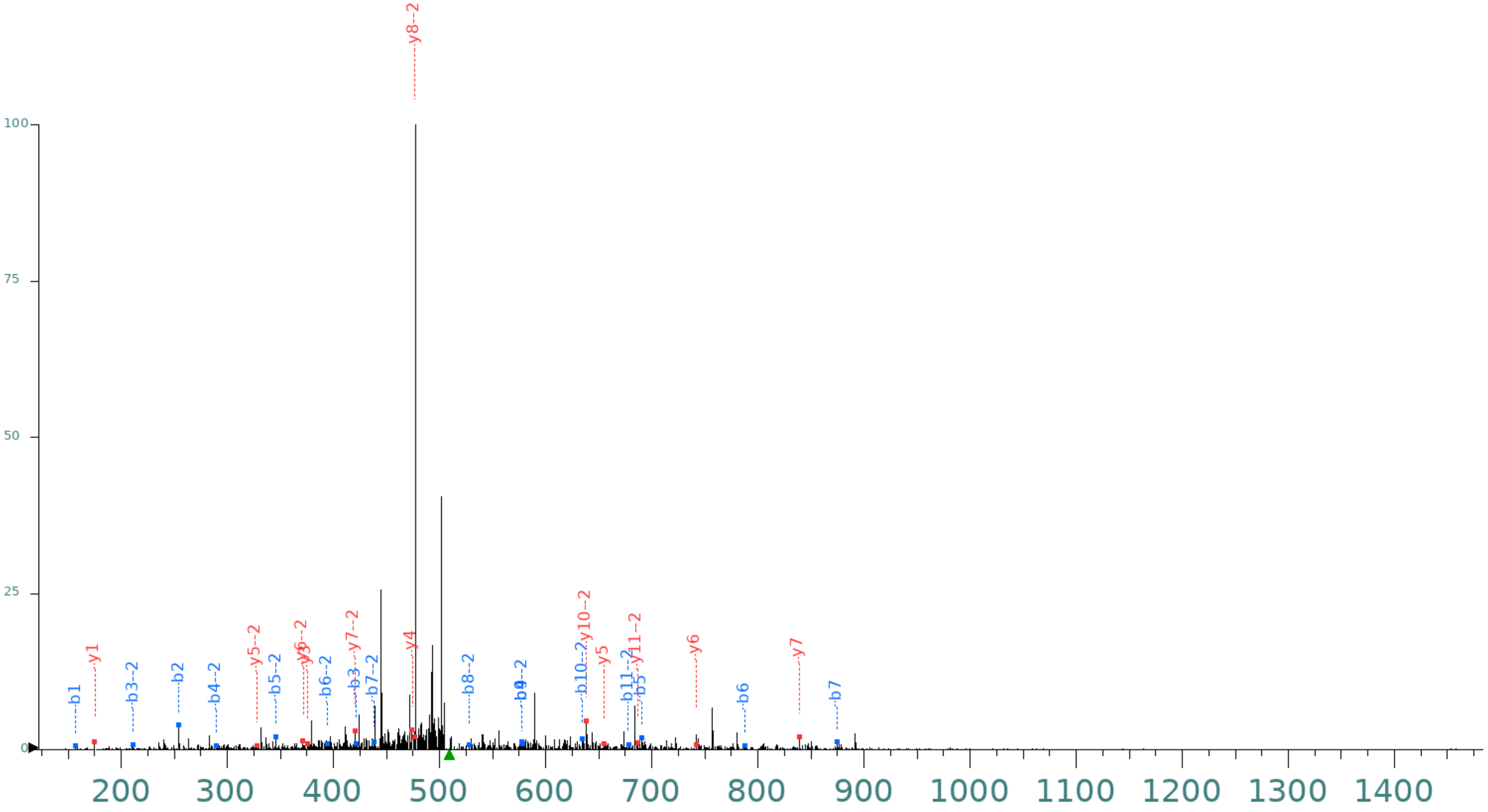

Predicted Fragmentation Pattern

+1

Seq	#	b: Δ Error	b	y	y: Δ Error	+1
R	1	---	157.108	---	---	12
P	2	-246.699	254.161	1372.639	---	11
S#	3	440.164	421.160	1275.586	---	10
R	4	---	577.261	1108.587	---	9
I	5	-227.300	690.345	952.486	---	8
P	6	---	787.397	839.402	-30.586	7
S	7	-382.898	874.429	742.349	308.543	6
T#	8	---	1055.443	655.317	---	5
V	9	---	1154.512	474.303	-323.470	4
L	10	---	1267.596	375.235	1298.850	3
S	11	---	1354.628	262.151	7.760	2
R	12	---	---	175.119	---	1

+2

Seq	#	b: Δ Error	b	y	y: Δ Error	+1
R	1	---	79.058	---	---	12
P	2	---	127.584	686.823	---	11
S#	3	-211.866	211.083	638.297	184.762	10
R	4	---	289.134	554.797	---	9
I	5	-107.234	345.676	476.747	---	8
P	6	---	394.202	420.205	-241.599	7
S	7	---	437.718	371.678	-1255.055	6
T#	8	-275.615	528.225	328.162	-12.703	5
V	9	635.546	577.760	237.655	---	4
L	10	25.043	634.302	188.121	---	3
S	11	---	677.818	131.579	---	2
R	12	---	---	88.063	---	1

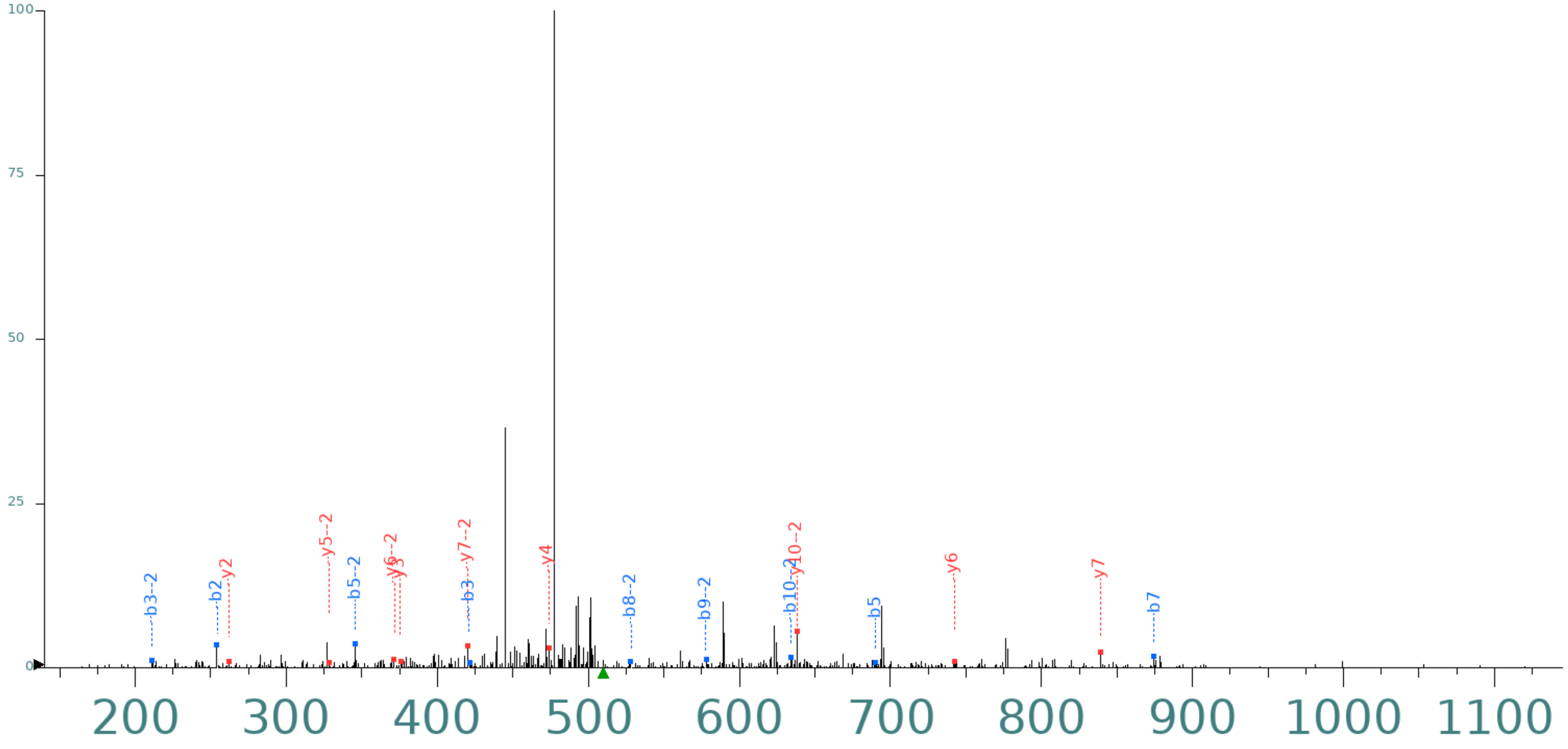

Predicted Fragmentation Pattern

+1

Seq	#	b: Δ Error	b	y	y: Δ Error	+1
R	1	---	157.108	---	---	12
P	2	634.705	**254.161**	1372.639	---	11
S#	3	---	421.160	1275.586	---	10
R	4	---	577.261	1108.587	---	9
I	5	-274.359	**690.345**	952.486	---	8
P	6	---	787.397	**839.402**	11.661	7
S#	7	---	954.396	**742.349**	-38.410	6
T	8	---	1055.443	**575.351**	-6.475	5
V	9	---	1154.512	**474.303**	697.511	4
L	10	---	1267.596	**375.235**	-413.482	3
S	11	---	1354.628	262.151	---	2
R	12	---	---	175.119	---	1

+2

Seq	#	b: Δ Error	b	y	y: Δ Error	+1
R	1	---	79.058	---	---	12
P	2	---	127.584	686.823	---	11
S#	3	---	211.083	**638.297**	302.224	10
R	4	66.784	**289.134**	**554.797**	137.512	9
I	5	142.600	**345.676**	476.747	---	8
P	6	-177.702	**394.202**	**420.205**	150.905	7
S#	7	36.916	**477.702**	**371.678**	1048.778	6
T	8	---	528.225	288.179	---	5
V	9	---	577.760	237.655	---	4
L	10	135.779	**634.302**	188.121	---	3
S	11	-281.936	**677.818**	131.579	---	2
R	12	---	---	88.063	---	1

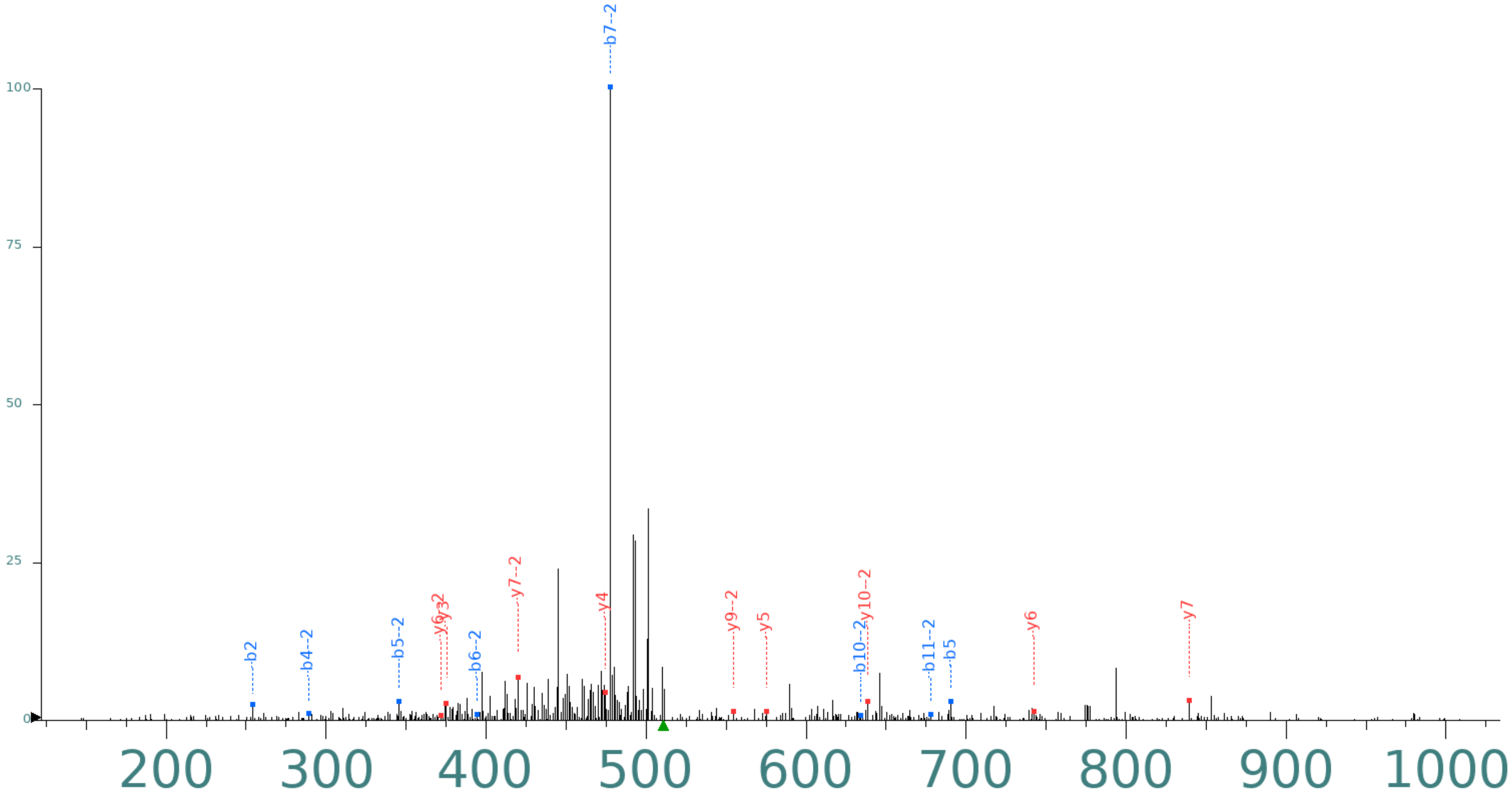

Predicted Fragmentation Pattern

+1

Seq	#	b: Δ Error	b	y	y: Δ Error	+1
R	1	---	157.108	---	---	12
P	2	-543.811	**254.161**	1372.639	---	11
S	3	-184.297	**341.193**	1275.586	---	10
R	4	---	497.294	1188.554	---	9
I	5	---	610.378	1032.453	---	8
P	6	---	707.431	919.369	---	7
S	7	-341.622	**794.463**	822.316	---	6
T#	8	---	975.477	735.284	---	5
V	9	---	1074.546	554.270	---	4
L	10	---	1187.630	**455.201**	-259.698	3
S#	11	---	1354.628	342.117	---	2
R	12	---	---	**175.119**	-904.367	1

+2

Seq	#	b: Δ Error	b	y	y: Δ Error	+1
R	1	---	79.058	---	---	12
P	2	---	127.584	686.823	---	11
S	3	---	171.100	**638.297**	255.109	10
R	4	-227.615	**249.151**	594.781	---	9
I	5	---	305.693	516.730	---	8
P	6	---	354.219	**460.188**	502.803	7
S	7	788.192	**397.735**	**411.662**	-1168.785	6
T#	8	---	488.242	368.146	---	5
V	9	433.035	**537.776**	277.639	---	4
L	10	---	594.318	228.104	---	3
S#	11	---	677.818	**171.562**	2476.664	2
R	12	---	---	88.063	---	1

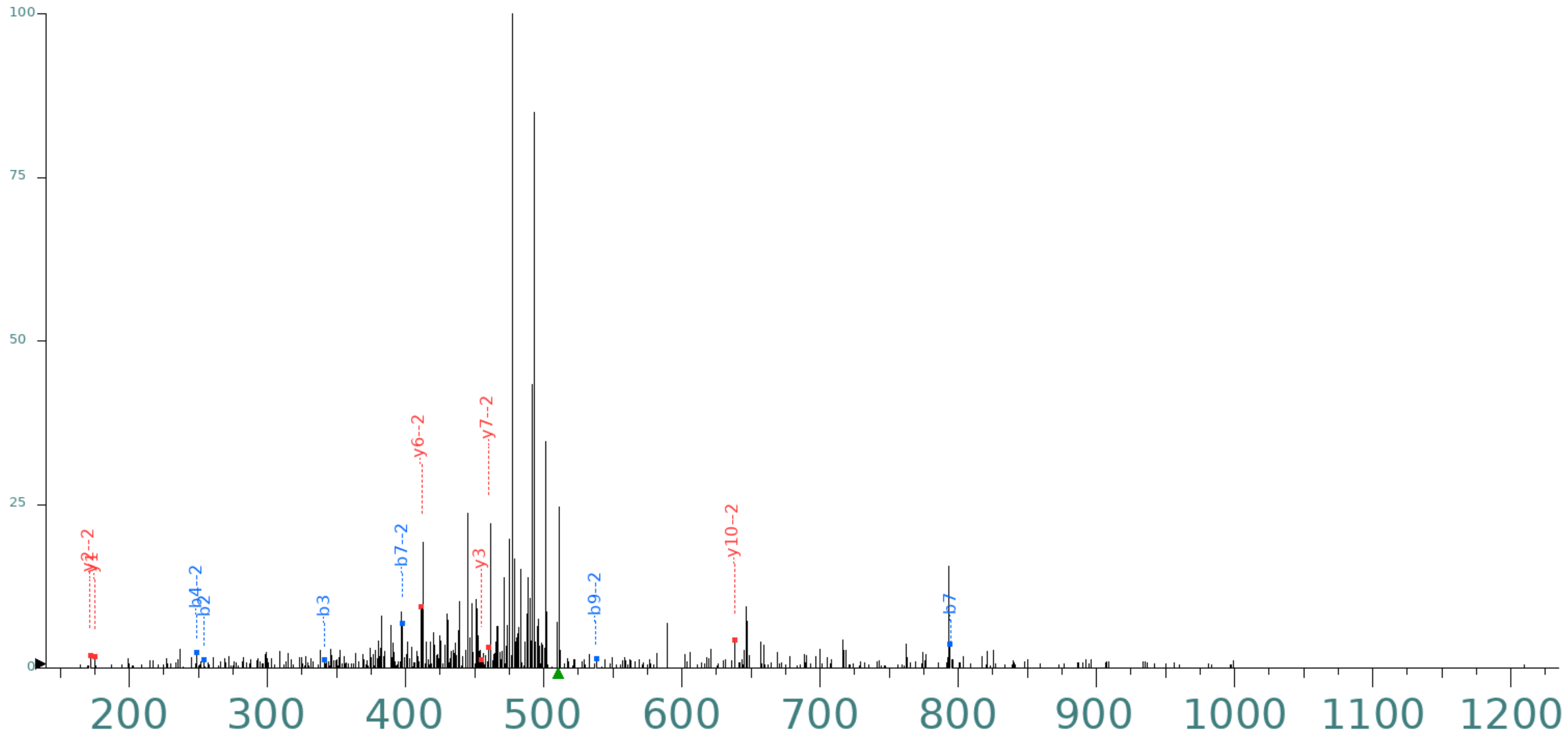

Predicted Fragmentation Pattern

+1

Seq	#	b: Δ Error	b	y	y: Δ Error	+1
R	1	---	157.108	---	---	12
P	2	46.455	**254.161**	1372.639	---	11
S#	3	341.115	**421.160**	1275.586	---	10
R	4	50.219	**577.261**	1108.587	---	9
I	5	262.312	**690.345**	952.486	---	8
P	6	254.743	**787.397**	**839.402**	-7.099	7
S	7	157.615	**874.429**	**742.349**	39.041	6
T	8	---	975.477	**655.317**	-650.098	5
V	9	---	1074.546	554.270	---	4
L	10	---	1187.630	455.201	---	3
S#	11	---	1354.628	**342.117**	1360.080	2
R	12	---	---	**175.119**	527.955	1

+2

Seq	#	b: Δ Error	b	y	y: Δ Error	+1
R	1	---	79.058	---	---	12
P	2	---	127.584	686.823	---	11
S#	3	---	211.083	**638.297**	-247.859	10
R	4	-26.305	**289.134**	**554.797**	422.727	9
I	5	---	345.676	**476.747**	237.611	8
P	6	-430.851	**394.202**	**420.205**	-123.612	7
S	7	678.208	**437.718**	**371.678**	-1270.448	6
T	8	-208.618	**488.242**	328.162	---	5
V	9	---	537.776	277.639	---	4
L	10	---	594.318	**228.104**	-833.730	3
S#	11	---	677.818	171.562	---	2
R	12	---	---	88.063	---	1

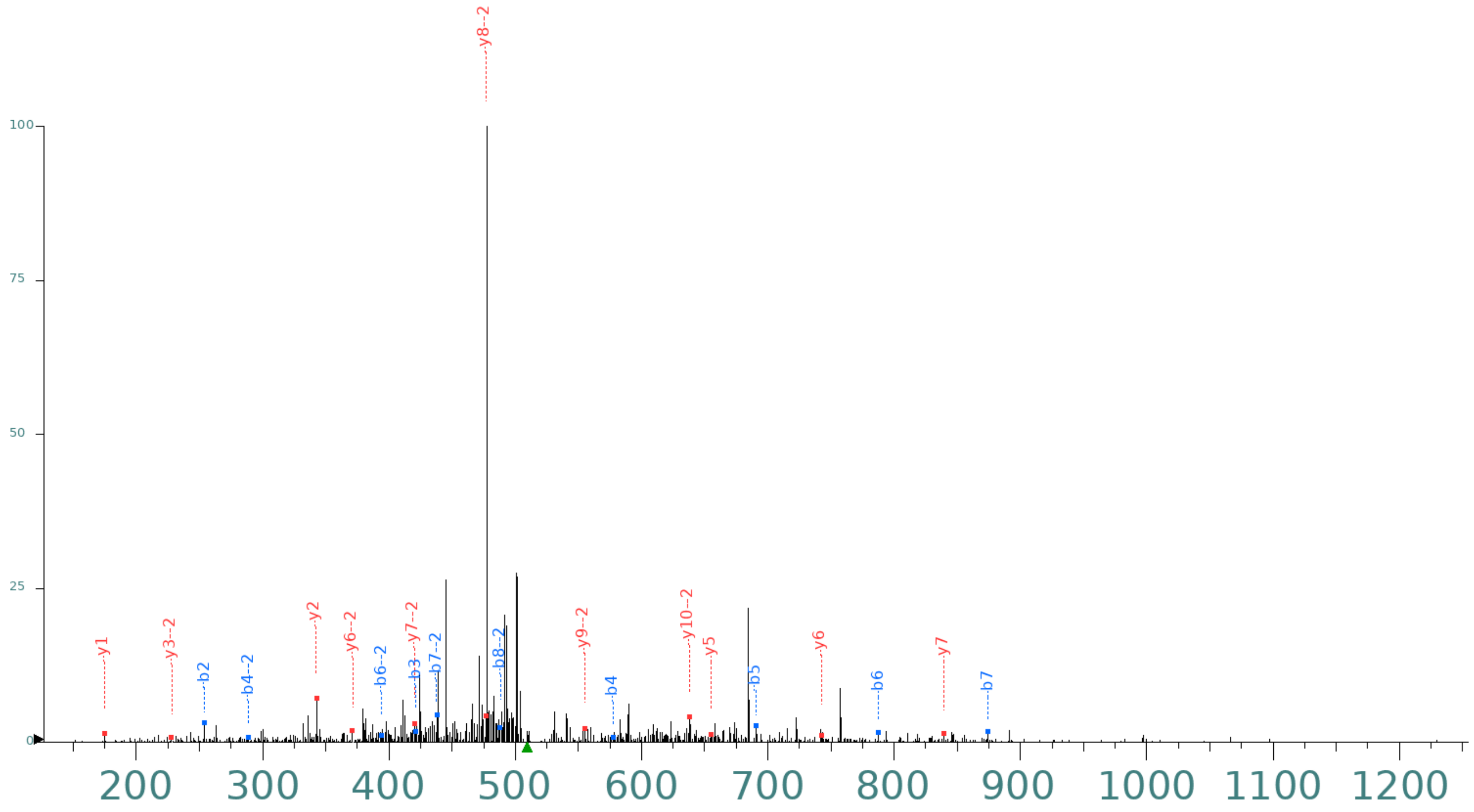

LPA3-PMA Jan 24 2020

Predicted Fragmentation Pattern

Seq	#	b: Δ Error	b	y	y: Δ Error	+1
S	1	---	88.039	---	---	20
D	2	---	203.066	2151.890	---	19
T#	3	---	384.080	2036.863	---	18
G	4	---	441.102	**1855.849**	-33.716	17
S	5	---	528.134	1798.828	---	16
Q	6	---	656.192	**1711.796**	47.252	15
Y	7	454.349	**819.256**	**1583.737**	-21.003	14
I	8	80.780	**932.340**	**1420.674**	15.491	13
E	9	---	1061.382	**1307.590**	-13.635	12
D	10	-10.959	**1176.409**	**1178.547**	39.655	11
S	11	56.809	**1263.441**	**1063.520**	-143.198	10
I	12	---	1376.525	**976.488**	80.495	9
S	13	100.076	**1463.557**	**863.404**	121.661	8
Q	14	76.392	**1591.616**	776.372	---	7
G	15	---	1648.637	**648.313**	150.017	6
A	16	---	1719.675	591.292	---	5
V	17	-78.212	**1818.743**	**520.255**	99.881	4
C	18	-20.005	**1978.774**	**421.186**	67.865	3
N	19	---	2092.817	261.156	---	2
K	20	---	---	147.113	---	1

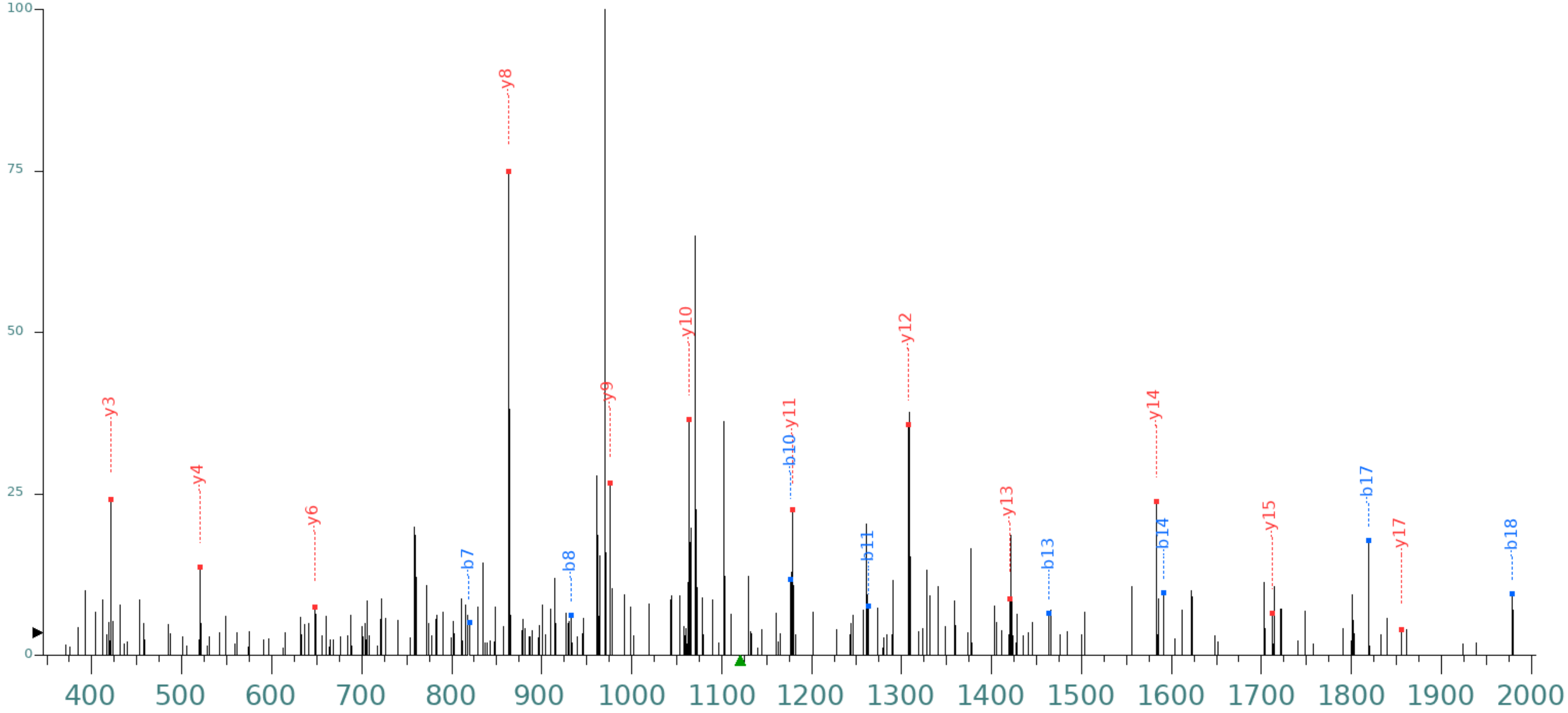

Predicted Fragmentation Pattern

+1

Seq	#	b: Δ Error	b	y	y: Δ Error	+1
S	1	---	88.039	---	---	20
D	2	---	203.066	2151.890	---	19
T	3	---	304.114	2036.863	---	18
G	4	---	361.135	1935.815	---	17
S	5	---	448.167	1878.794	---	16
Q	6	272.706	**576.226**	1791.762	---	15
Y	7	-244.706	**739.289**	1663.703	---	14
I	8	187.244	**852.373**	1500.640	---	13
E	9	320.278	**981.416**	1387.556	---	12
D	10	---	1096.443	1258.513	---	11
S	11	---	1183.475	1143.486	---	10
I	12	66.045	**1296.559**	1056.454	---	9
S#	13	---	1463.557	**943.370**	171.738	8
Q	14	---	1591.616	**776.372**	-46.387	7
G	15	---	1648.637	**648.313**	-118.098	6
A	16	---	1719.675	**591.292**	-36.710	5
V	17	---	1818.743	**520.255**	274.384	4
C	18	---	1978.774	**421.186**	151.968	3
N	19	---	2092.817	261.156	---	2
K	20	---	---	147.113	---	1

+2

Seq	#	b: Δ Error	b	y	y: Δ Error	+1
S	1	---	44.523	---	---	20
D	2	---	102.037	1076.449	---	19
T	3	---	152.561	1018.935	---	18
G	4	---	181.071	968.411	---	17
S	5	---	224.587	939.901	---	16
Q	6	---	288.617	**896.385**	-266.586	15
Y	7	---	370.148	**832.355**	180.408	14
I	8	---	426.690	750.824	---	13
E	9	879.031	**491.212**	**694.282**	174.224	12
D	10	-873.707	**548.725**	**629.760**	0.590	11
S	11	85.488	**592.241**	**572.247**	218.155	10
I	12	---	648.783	**528.731**	819.299	9
S#	13	-286.939	**732.282**	472.189	---	8
Q	14	---	796.312	**388.690**	-597.080	7
G	15	319.249	**824.822**	324.660	---	6
A	16	107.584	**860.341**	296.150	---	5
V	17	-56.875	**909.875**	260.631	---	4
C	18	-363.416	**989.890**	211.097	---	3
N	19	334.733	**1046.912**	131.082	---	2
K	20	---	---	74.060	---	1

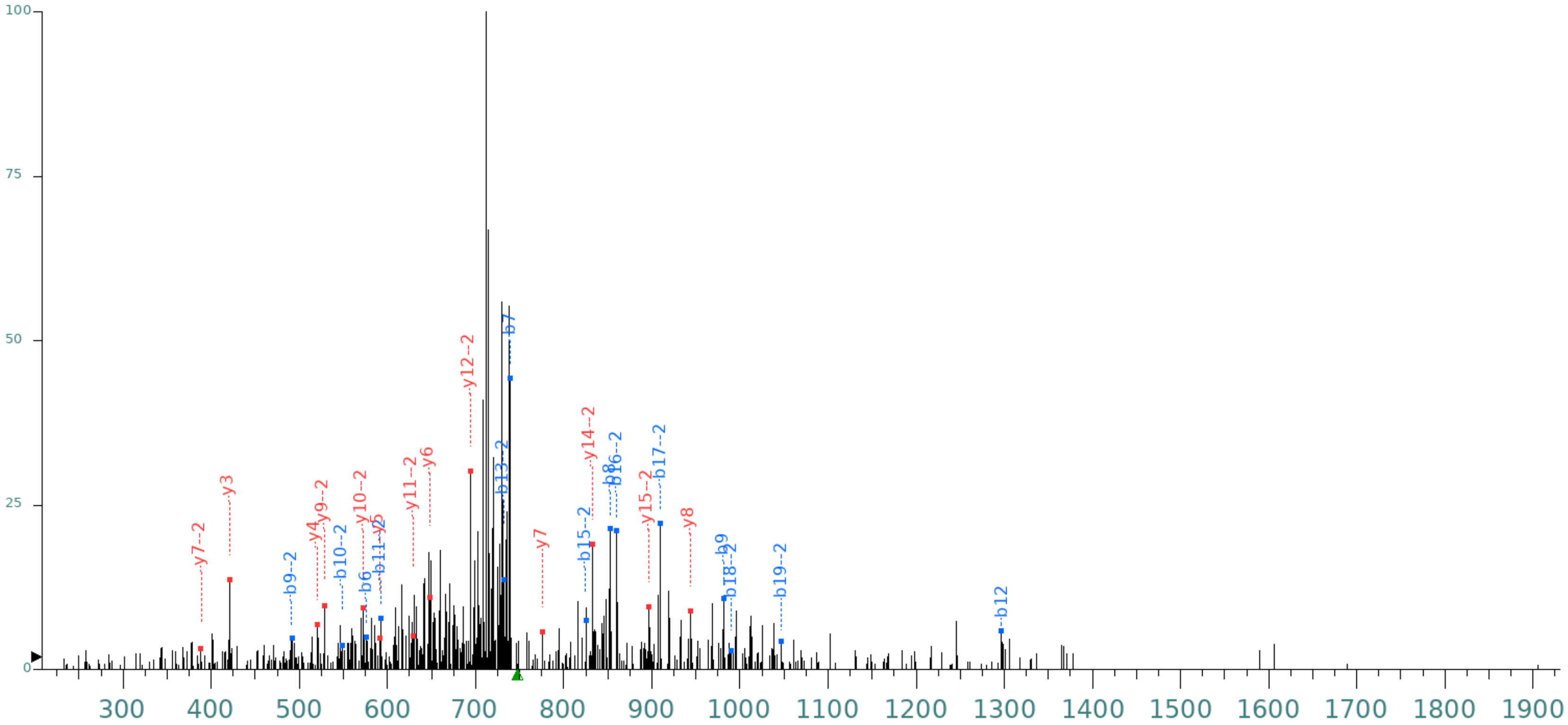

Predicted Fragmentation Pattern

+1

Seq	#	b: Δ Error	b	y	y: Δ Error	+1
S	1	---	88.039	---	---	20
D	2	---	203.066	2151.890	---	19
T	3	---	304.114	2036.863	---	18
G	4	---	361.135	1935.815	---	17
S	5	---	448.167	1878.794	---	16
Q	6	860.120	576.226	1791.762	---	15
Y	7	---	739.289	1663.703	---	14
I	8	387.483	852.373	1500.640	---	13
E	9	73.228	981.416	1387.556	---	12
D	10	---	1096.443	1258.513	---	11
S	11	61.181	1183.475	1143.486	---	10
I	12	---	1296.559	1056.454	---	9
S#	13	---	1463.557	943.370	111.650	8
Q	14	---	1591.616	776.372	---	7
G	15	---	1648.637	648.313	138.818	6
A	16	---	1719.675	591.292	125.234	5
V	17	---	1818.743	520.255	218.686	4
C	18	---	1978.774	421.186	17.295	3
N	19	---	2092.817	261.156	-399.927	2
K	20	---	---	147.113	---	1

+2

Seq	#	b: Δ Error	b	y	y: Δ Error	+1
S	1	---	44.523	---	---	20
D	2	---	102.037	1076.449	---	19
T	3	---	152.561	1018.935	170.034	18
G	4	---	181.071	968.411	---	17
S	5	1204.212	224.587	939.901	319.553	16
Q	6	---	288.617	896.385	---	15
Y	7	---	370.148	832.355	-570.619	14
I	8	---	426.690	750.824	---	13
E	9	---	491.212	694.282	57.329	12
D	10	---	548.725	629.760	-295.388	11
S	11	---	592.241	572.247	---	10
I	12	-585.692	648.783	528.731	440.796	9
S#	13	-317.880	732.282	472.189	---	8
Q	14	---	796.312	388.690	---	7
G	15	178.945	824.822	324.660	---	6
A	16	149.926	860.341	296.150	---	5
V	17	-98.941	909.875	260.631	1610.011	4
C	18	494.572	989.890	211.097	---	3
N	19	---	1046.912	131.082	---	2
K	20	---	---	74.060	---	1

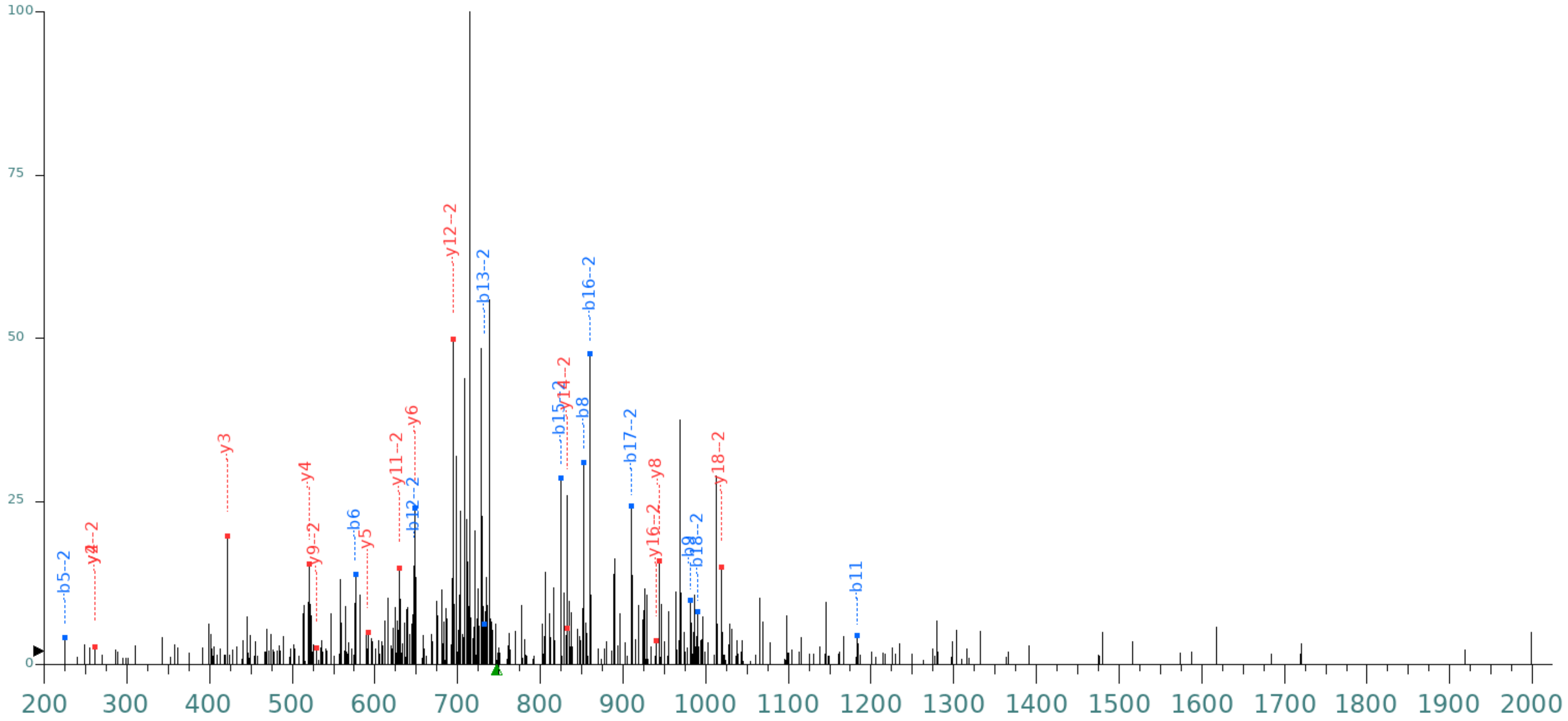

Predicted Fragmentation Pattern

+1

Seq	#	b: Δ Error	b	y	y: Δ Error	+1
R	1	---	157.108	---	---	12
P	2	31.747	**254.161**	1372.639	---	11
S#	3	-586.901	**421.160**	1275.586	---	10
R	4	256.440	**577.261**	1108.587	---	9
I	5	715.602	**690.345**	952.486	---	8
P	6	---	787.397	**839.402**	246.753	7
S	7	---	874.429	742.349	---	6
T#	8	---	1055.443	655.317	---	5
V	9	---	1154.512	**474.303**	-1003.178	4
L	10	---	1267.596	**375.235**	-530.870	3
S	11	---	1354.628	**262.151**	-489.793	2
R	12	---	---	**175.119**	-1187.096	1

+2

Seq	#	b: Δ Error	b	y	y: Δ Error	+1
R	1	---	79.058	---	---	12
P	2	---	127.584	686.823	---	11
S#	3	---	211.083	**638.297**	185.622	10
R	4	---	289.134	**554.797**	419.429	9
I	5	-740.589	**345.676**	**476.747**	-234.028	8
P	6	-18.039	**394.202**	**420.205**	-364.410	7
S	7	---	437.718	**371.678**	1151.351	6
T#	8	400.484	**528.225**	**328.162**	113.386	5
V	9	-607.709	**577.760**	237.655	---	4
L	10	-160.695	**634.302**	188.121	---	3
S	11	248.906	**677.818**	131.579	---	2
R	12	---	---	88.063	---	1

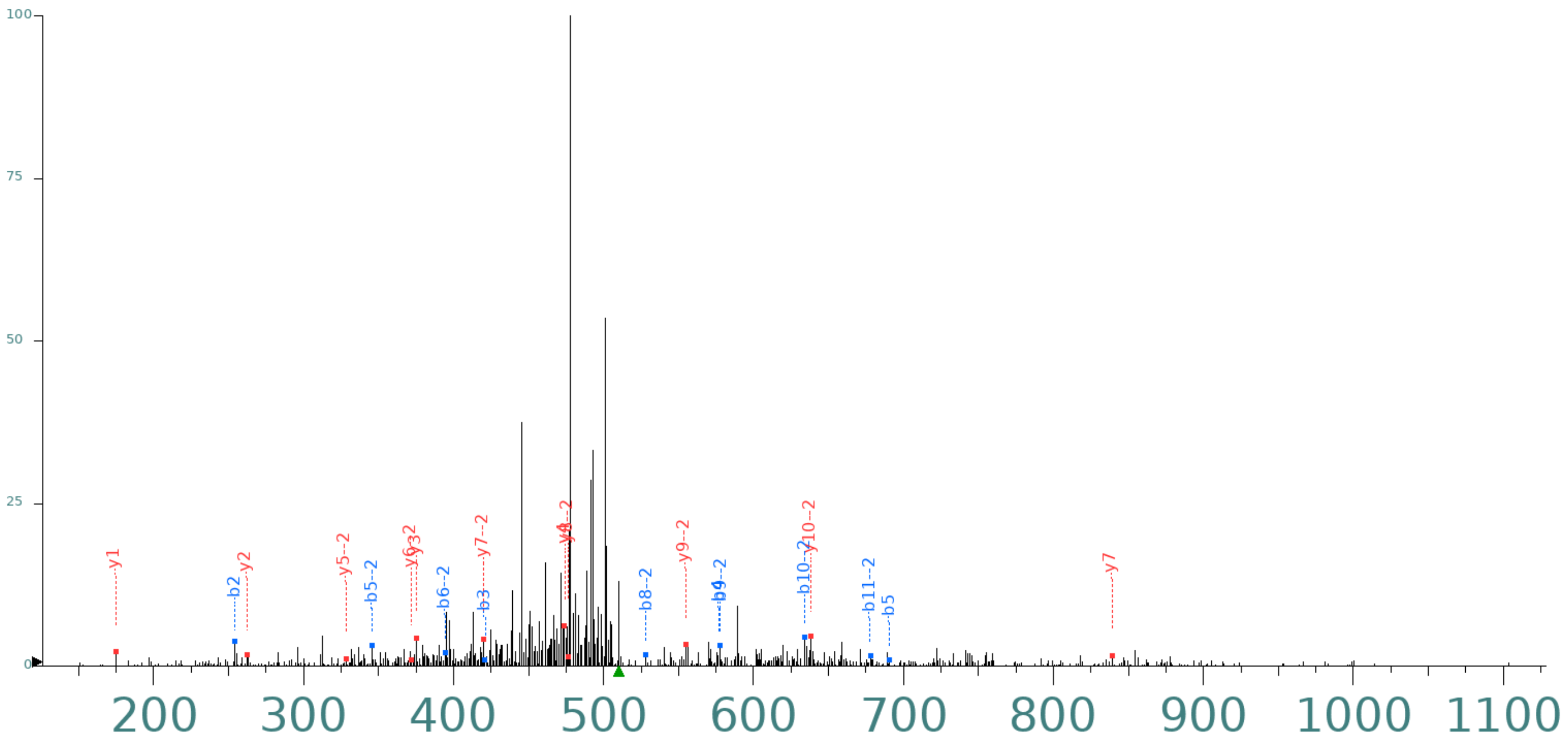

Predicted Fragmentation Pattern

+1

Seq	#	b: Δ Error	b	y	y: Δ Error	+1
R	1	---	157.108	---	---	12
P	2	-486.598	254.161	1372.639	---	11
S#	3	---	421.160	1275.586	---	10
R	4	---	577.261	1108.587	---	9
I	5	-151.590	690.345	952.486	---	8
P	6	---	787.397	839.402	127.549	7
S	7	89.646	874.429	742.349	121.575	6
T#	8	---	1055.443	655.317	---	5
V	9	---	1154.512	474.303	-108.089	4
L	10	---	1267.596	375.235	-16.588	3
S	11	---	1354.628	262.151	-465.556	2
R	12	---	---	175.119	---	1

+2

Seq	#	b: Δ Error	b	y	y: Δ Error	+1
R	1	---	79.058	---	---	12
P	2	---	127.584	686.823	---	11
S#	3	---	211.083	638.297	-2.623	10
R	4	-193.319	289.134	554.797	---	9
I	5	-1218.875	345.676	476.747	383.724	8
P	6	-463.860	394.202	420.205	530.332	7
S	7	-811.621	437.718	371.678	-105.548	6
T#	8	-246.598	528.225	328.162	---	5
V	9	---	577.760	237.655	---	4
L	10	326.599	634.302	188.121	---	3
S	11	91.198	677.818	131.579	---	2
R	12	---	---	88.063	---	1

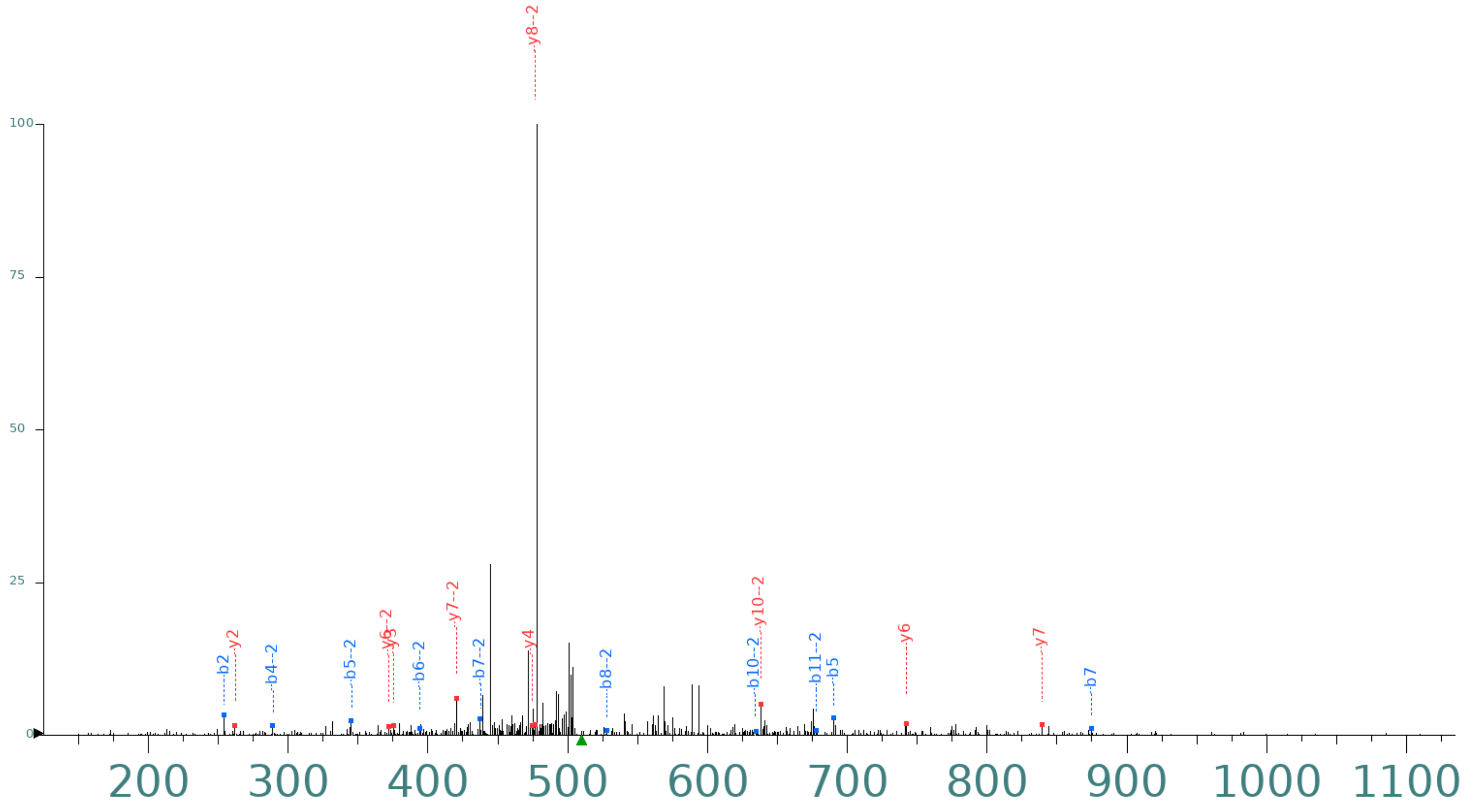

Predicted Fragmentation Pattern

Seq	#	b: Δ Error	b	y	y: Δ Error	+[illegible]
I	1	---	114.091	---	---	[illegible]
P	2	-762.276	211.144	839.402	-229.070	[illegible]
S#	3	-909.328	378.142	742.349	-121.628	[illegible]
T	4	---	479.190	575.351	-133.686	[illegible]
V	5	-291.526	578.259	474.303	757.326	[illegible]
L	6	47.542	691.343	375.235	131.008	[illegible]
S	7	---	778.375	262.151	90.171	[illegible]
R	8	---	---	175.119	-350.024	[illegible]

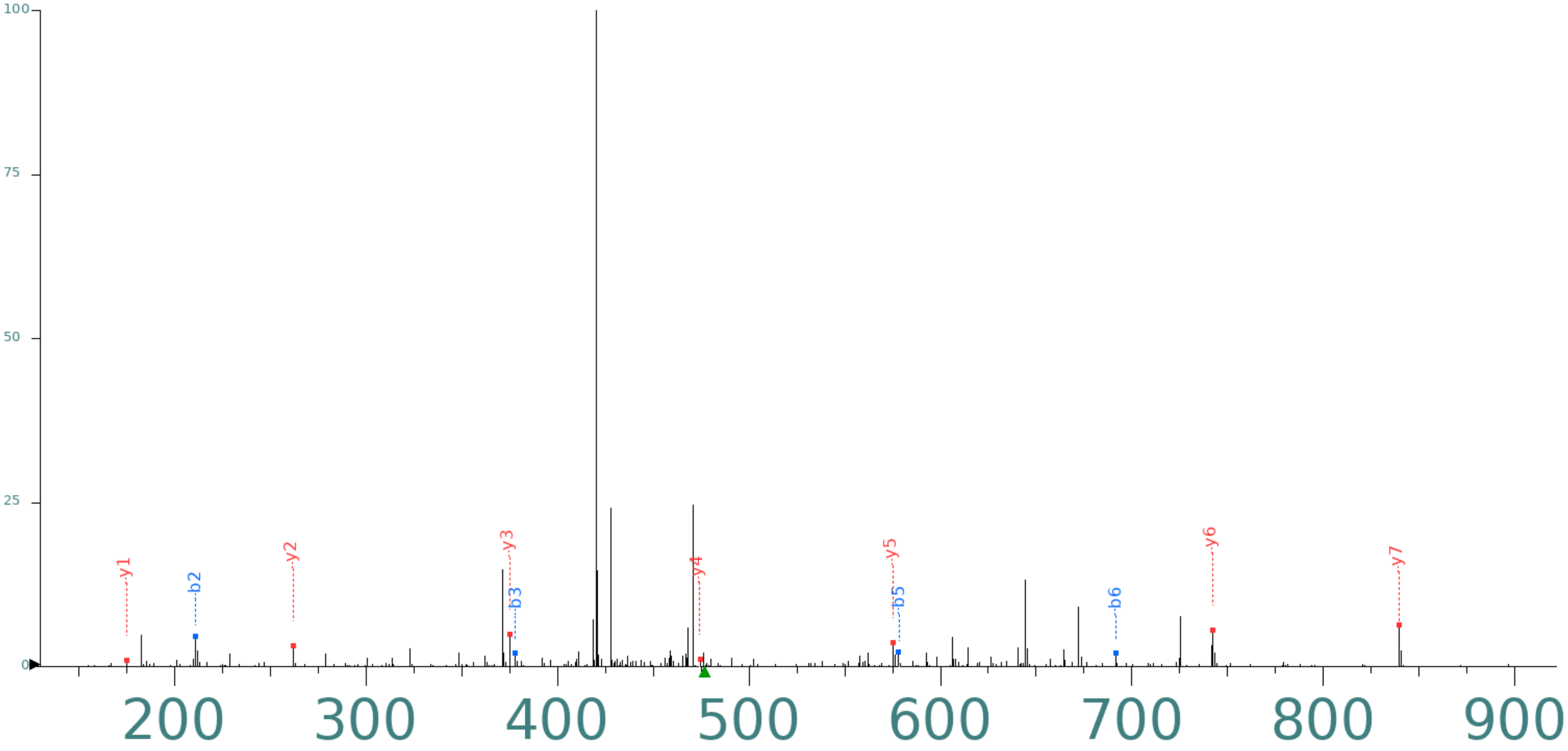

Predicted Fragmentation Pattern

+1

Seq	#	b: Δ Error	b	y	y: Δ Error	+1
T	1	---	102.055	---	---	14
N	2	-981.375	**216.098**	1434.674	---	13
V	3	-432.260	**315.166**	1320.631	---	12
L	4	-48.160	**428.250**	1221.562	---	11
S	5	---	515.282	1108.478	---	10
P	6	-41.281	**612.335**	**1021.446**	-96.951	9
H	7	---	749.394	924.393	---	8
T	8	---	850.442	**787.335**	-202.811	7
S	9	---	937.474	**686.287**	27.613	6
G	10	---	994.495	599.255	---	5
S#	11	---	1161.494	542.233	---	4
I	12	---	1274.578	**375.235**	1314.668	3
S	13	---	1361.610	**262.151**	-463.342	2
R	14	---	---	175.119	---	1

+2

Seq	#	b: Δ Error	b	y	y: Δ Error	+1
T	1	---	51.531	---	---	14
N	2	---	108.553	**717.840**	55.313	13
V	3	---	158.087	**660.819**	174.650	12
L	4	---	214.629	**611.285**	201.220	11
S	5	---	258.145	**554.743**	119.949	10
P	6	---	306.671	**511.227**	419.802	9
H	7	---	375.201	**462.700**	-308.508	8
T	8	---	425.725	**394.171**	-178.326	7
S	9	502.901	**469.241**	343.647	---	6
G	10	---	497.751	**300.131**	407.070	5
S#	11	---	581.250	**271.620**	-1358.999	4
I	12	---	637.792	188.121	---	3
S	13	---	681.308	131.579	---	2
R	14	---	---	88.063	---	1

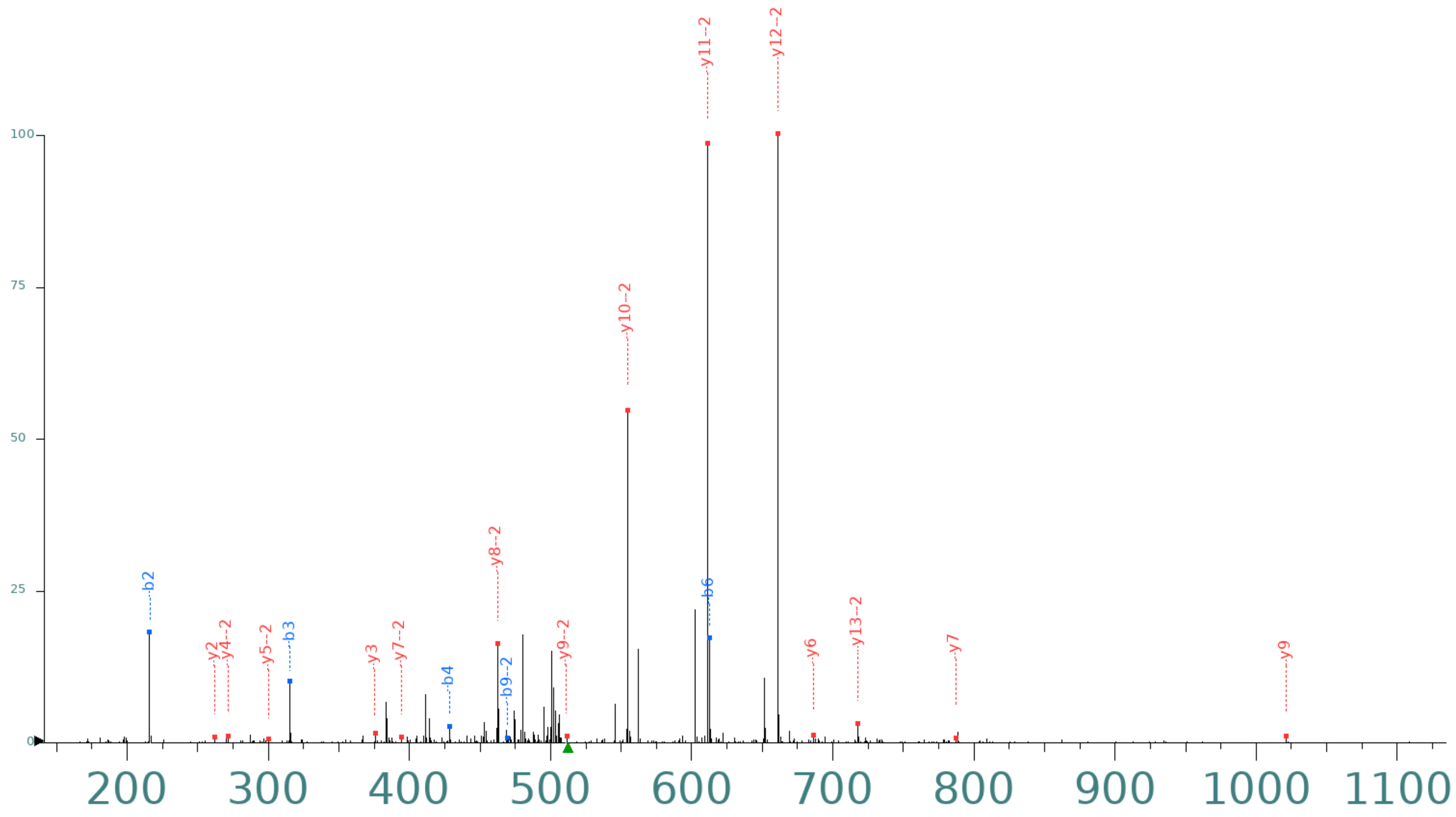

Predicted Fragmentation Pattern

Seq	#	b: Δ Error	b	y	y: Δ Error	+1
I	1	---	114.091	---	---	8
P	2	-385.476	211.144	839.402	130.020	7
S#	3	96.092	378.142	742.349	6.730	6
T	4	---	479.190	575.351	180.836	5
V	5	260.412	578.259	474.303	-406.467	4
L	6	82.586	691.343	375.235	198.327	3
S	7	---	778.375	262.151	366.989	2
R	8	---	---	175.119	---	1

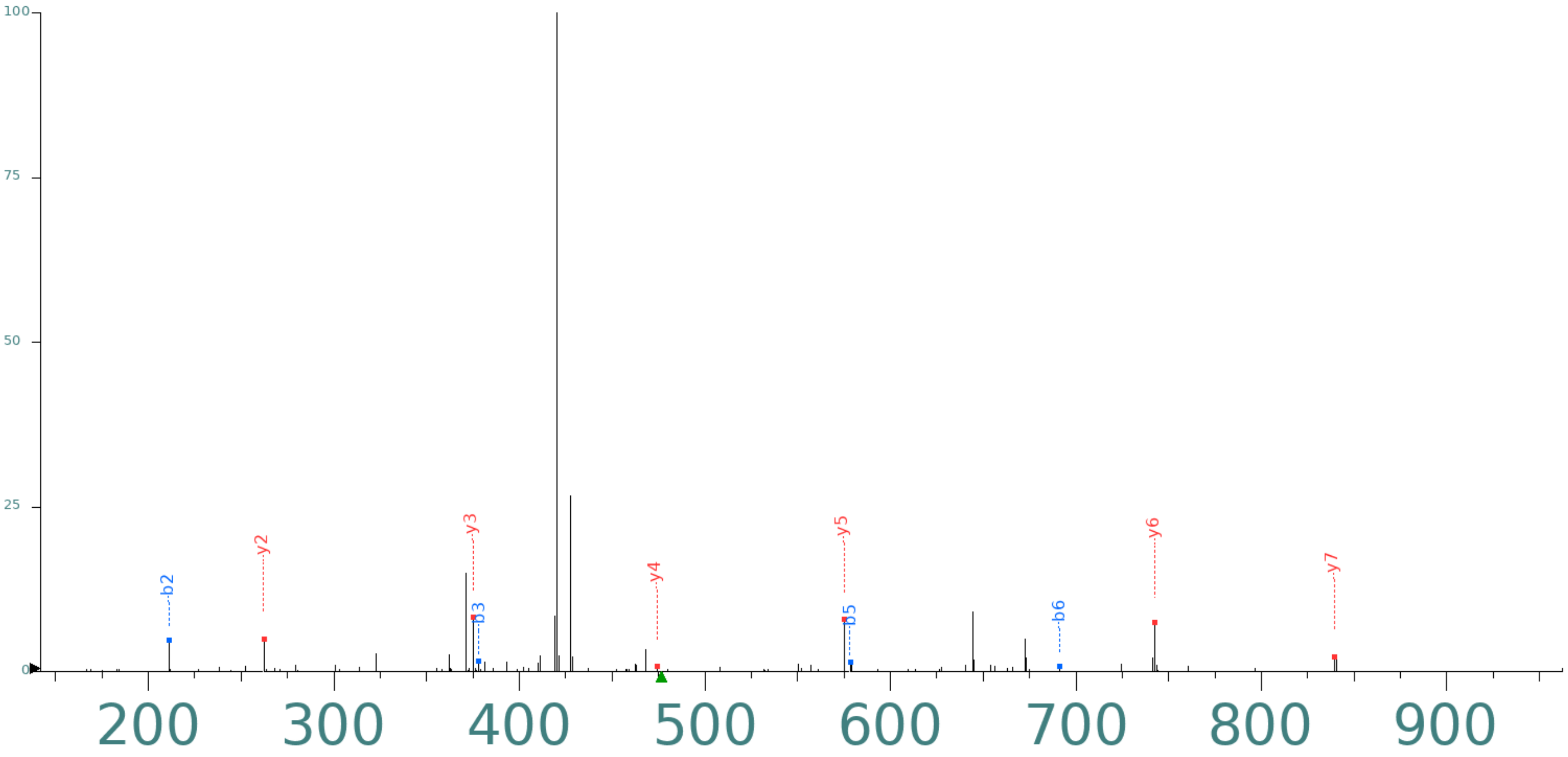

Predicted Fragmentation Pattern

Seq	#	b: Δ Error	b	y	y: Δ Error	+1
I	1	---	114.091	---	---	8
P	2	-295.659	211.144	839.402	-96.981	7
S#	3	199.039	378.142	742.349	134.069	6
T	4	---	479.190	575.351	-0.322	5
V	5	-179.801	578.259	474.303	514.744	4
L	6	156.905	691.343	375.235	-325.582	3
S	7	---	778.375	262.151	249.486	2
R	8	---	---	175.119	---	1

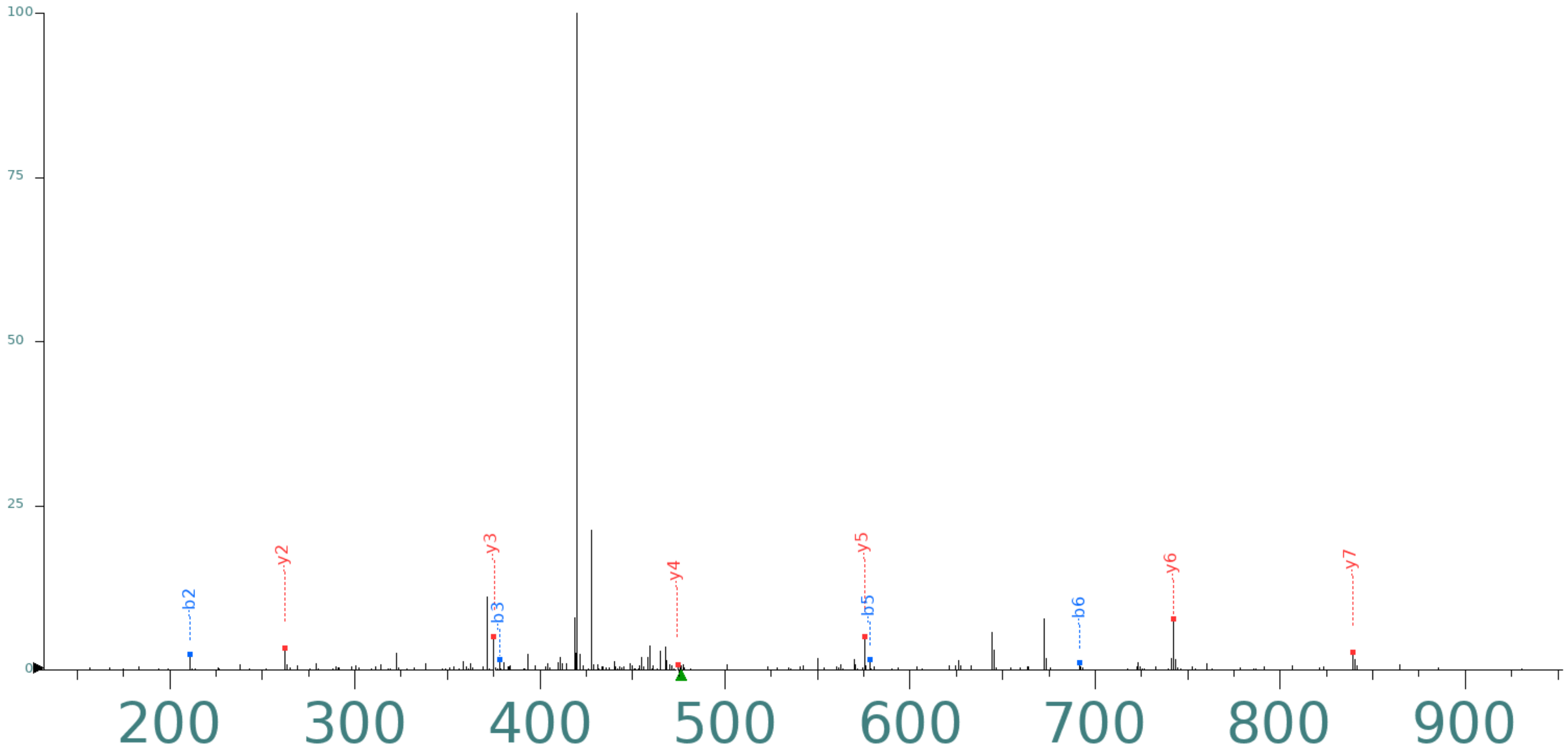

Predicted Fragmentation Pattern

+1

Seq	#	b: Δ Error	b	y	y: Δ Error	+1
R	1	---	157.108	---	---	12
P	2	-682.544	**254.161**	1292.672	---	11
S#	3	---	421.160	1195.619	---	10
R	4	---	577.261	1028.621	---	9
I	5	139.203	**690.345**	872.520	---	8
P	6	---	787.397	**759.436**	-32.411	7
S	7	---	874.429	**662.383**	158.576	6
T	8	---	975.477	**575.351**	252.411	5
V	9	---	1074.546	**474.303**	299.953	4
L	10	---	1187.630	**375.235**	36.682	3
S	11	---	1274.662	262.151	---	2
R	12	---	---	175.119	---	1

+2

Seq	#	b: Δ Error	b	y	y: Δ Error	+1
R	1	---	79.058	---	---	12
P	2	---	127.584	**646.840**	-719.485	11
S#	3	---	211.083	**598.313**	49.550	10
R	4	---	289.134	**514.814**	176.300	9
I	5	-733.427	**345.676**	**436.764**	-654.276	8
P	6	181.894	**394.202**	**380.222**	193.357	7
S	7	-889.770	**437.718**	**331.695**	209.727	6
T	8	---	488.242	**288.179**	213.631	5
V	9	---	537.776	237.655	---	4
L	10	---	594.318	188.121	---	3
S	11	---	637.834	131.579	---	2
R	12	---	---	88.063	---	1

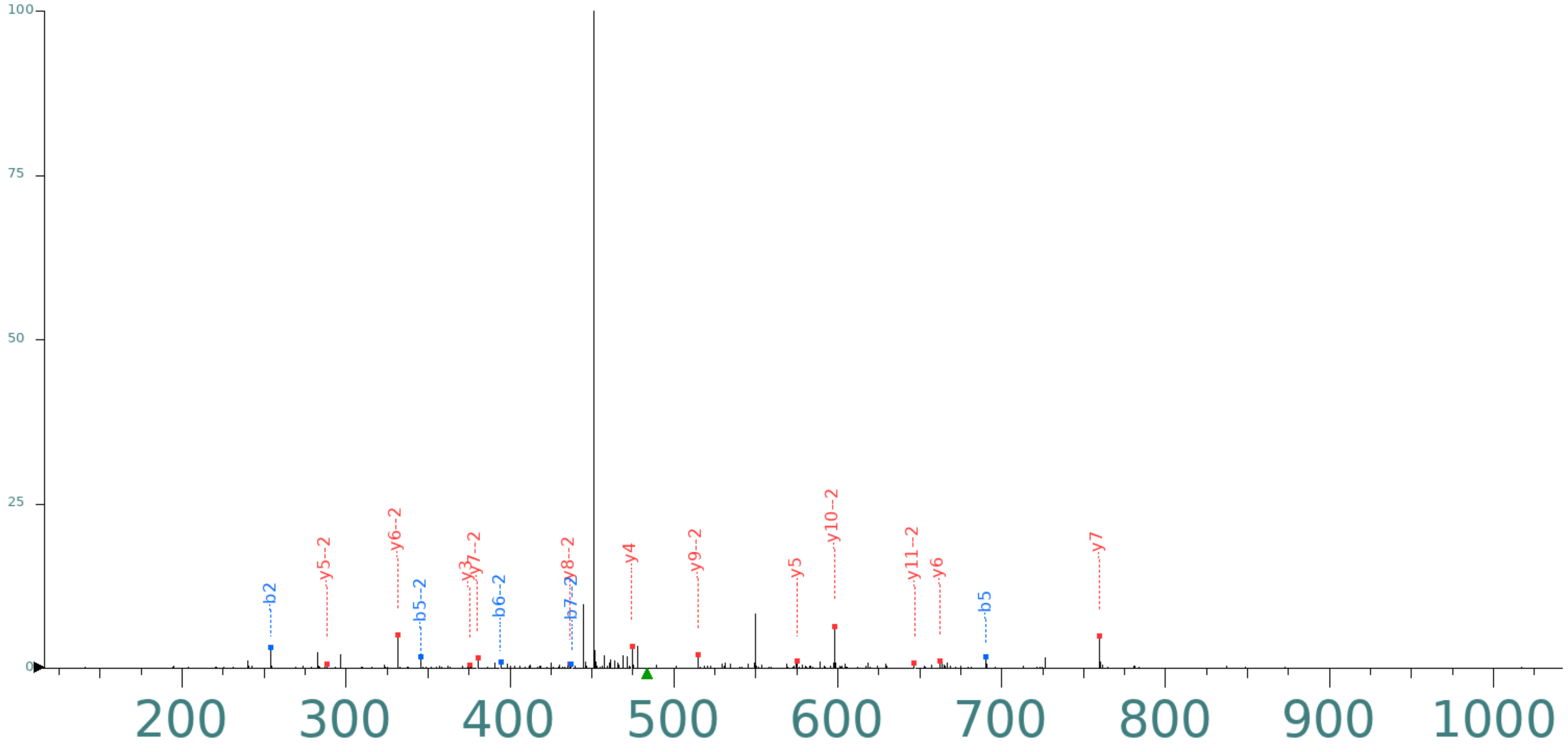

Predicted Fragmentation Pattern

+1

Seq	#	b: Δ Error	b	y	y: Δ Error	+1
R	1	---	157.108	---	---	12
P	2	155.758	**254.161**	1372.639	---	11
S#	3	---	421.160	1275.586	---	10
R	4	33.092	**577.261**	1108.587	---	9
I	5	671.545	**690.345**	952.486	---	8
P	6	---	787.397	**839.402**	225.604	7
S#	7	---	954.396	**742.349**	199.822	6
T	8	---	1055.443	**575.351**	-101.430	5
V	9	---	1154.512	**474.303**	170.622	4
L	10	---	1267.596	**375.235**	-122.818	3
S	11	---	1354.628	262.151	---	2
R	12	---	---	175.119	---	1

+2

Seq	#	b: Δ Error	b	y	y: Δ Error	+1
R	1	---	79.058	---	---	12
P	2	---	127.584	686.823	---	11
S#	3	---	211.083	**638.297**	-125.226	10
R	4	-183.605	**289.134**	**554.797**	254.410	9
I	5	---	345.676	476.747	---	8
P	6	---	394.202	**420.205**	558.190	7
S#	7	79.458	**477.702**	371.678	---	6
T	8	---	528.225	288.179	---	5
V	9	-831.250	**577.760**	237.655	---	4
L	10	197.630	**634.302**	188.121	---	3
S	11	---	677.818	131.579	---	2
R	12	---	---	88.063	---	1

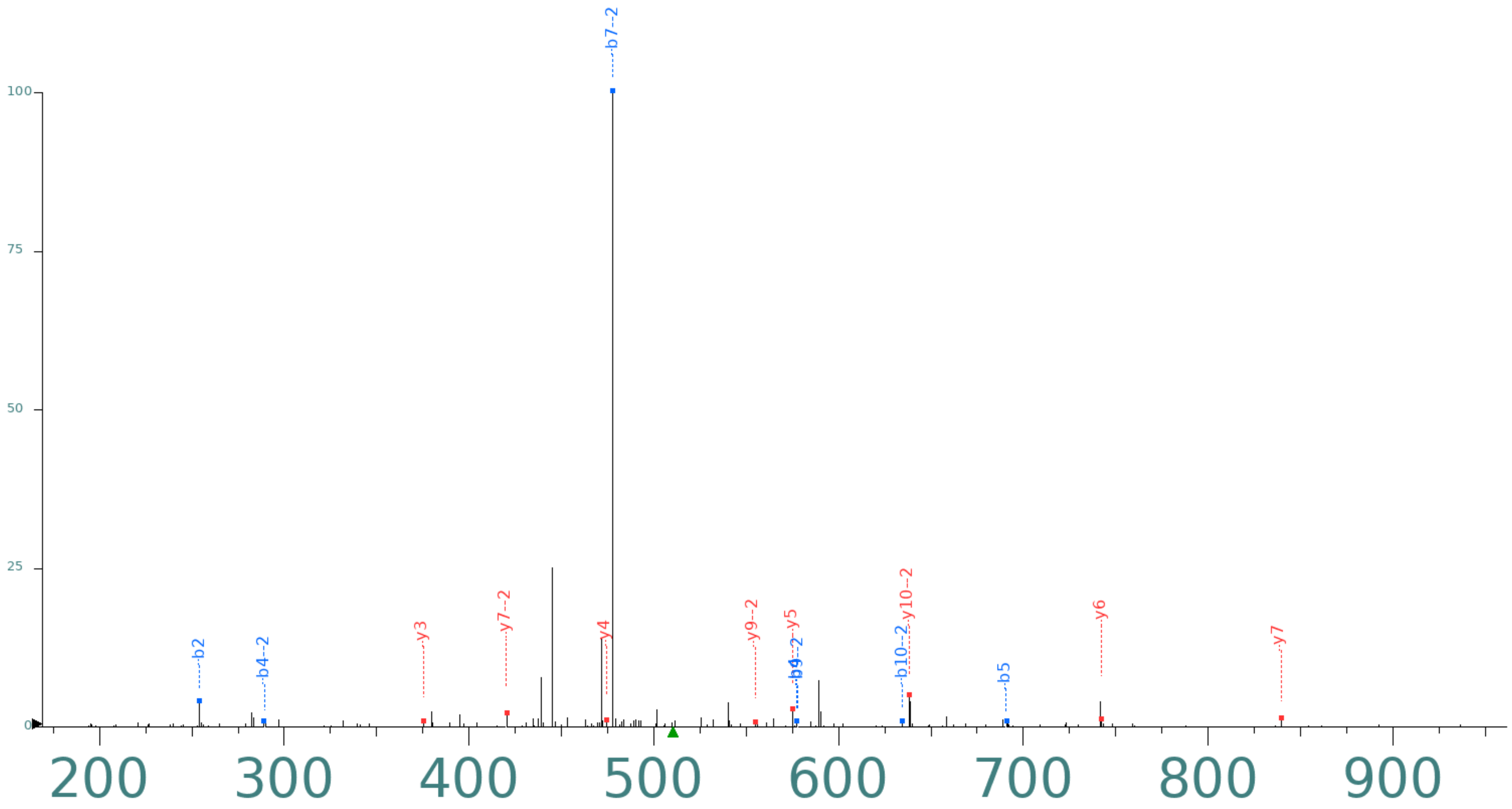

Predicted Fragmentation Pattern

Seq	#	b: Δ Error	b	y	y: Δ Error	+1
I	1	---	114.091	---	---	8
P	2	-340.927	211.144	839.402	-87.599	7
S#	3	-56.594	378.142	742.349	-89.227	6
T	4	126.111	479.190	575.351	89.417	5
V	5	186.982	578.259	474.303	470.199	4
L	6	116.922	691.343	375.235	-256.981	3
S	7	---	778.375	262.151	-254.349	2
R	8	---	---	175.119	-498.626	1

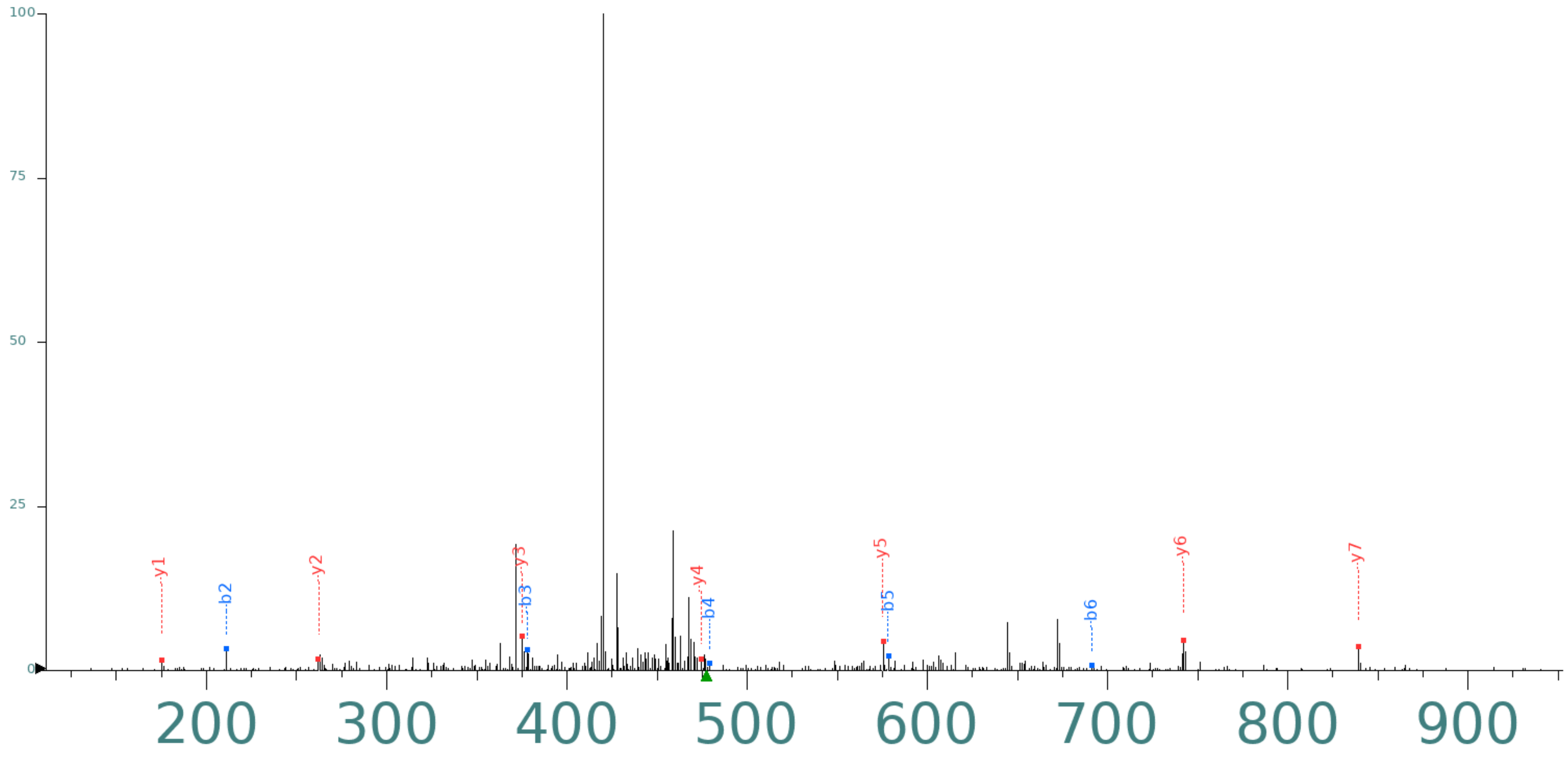

Predicted Fragmentation Pattern

Seq	#	b: Δ Error	b	y	y: Δ Error	+1
T	1	---	102.055	---	---	14
N	2	---	216.098	1514.640	---	13
V	3	491.734	**315.166**	1400.597	---	12
L	4	---	428.250	**1301.529**	75.599	11
S#	5	228.913	**595.249**	**1188.445**	46.298	10
P	6	339.532	**692.301**	**1021.446**	93.544	9
H	7	-54.661	**829.360**	**924.393**	96.531	8
T#	8	353.613	**1010.374**	**787.335**	-137.903	7
S	9	---	1097.406	**606.321**	627.136	6
G	10	---	1154.428	519.289	---	5
S	11	---	1241.460	462.267	---	4
I	12	---	1354.544	**375.235**	-151.129	3
S	13	---	1441.576	**262.151**	-102.260	2
R	14	---	---	175.119	---	1

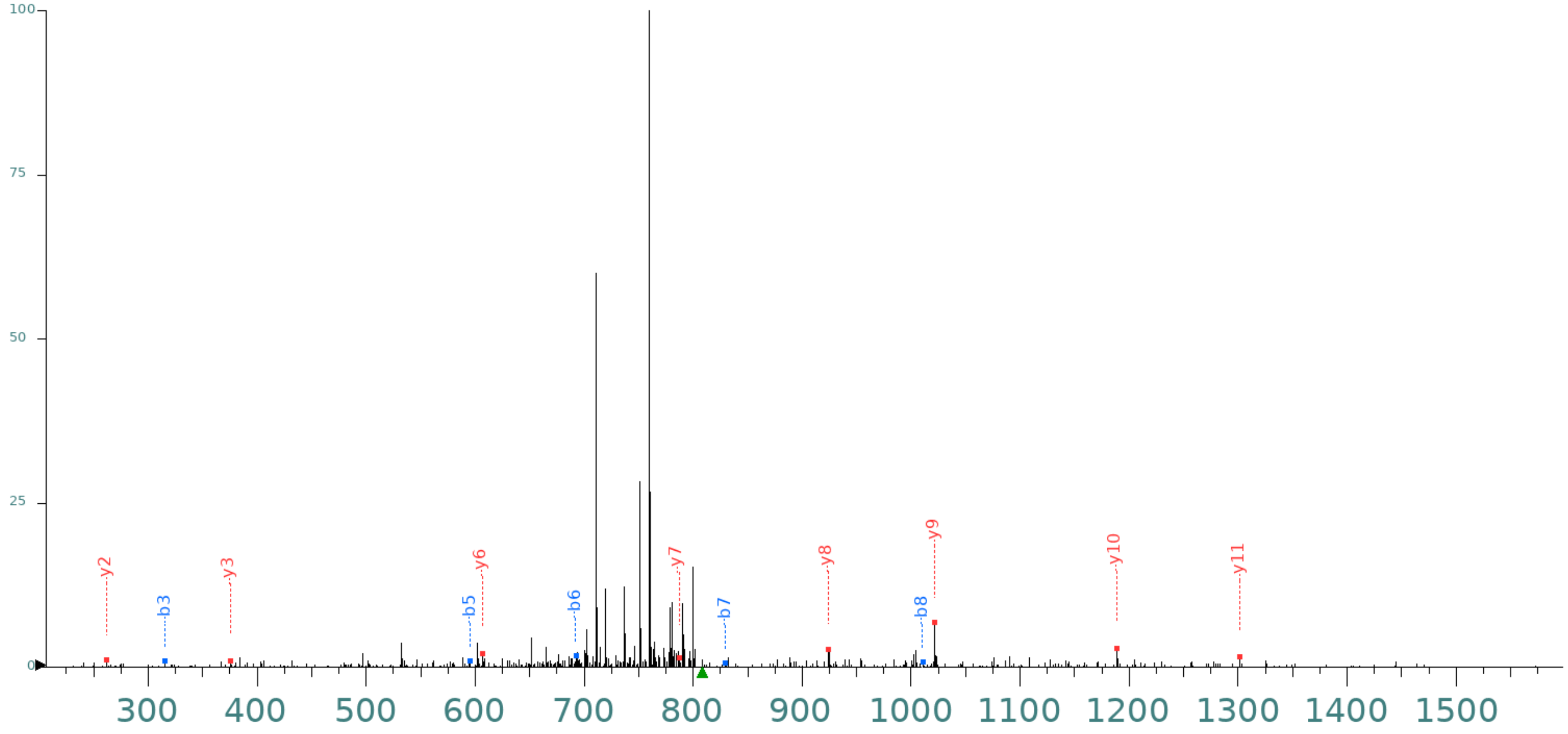

Predicted Fragmentation Pattern

+1

Seq	#	b: Δ Error	b	y	y: Δ Error	+1
R	1	---	157.108	---	---	12
P	2	-685.791	**254.161**	1372.639	---	11
S#	3	---	421.160	1275.586	---	10
R	4	698.824	**577.261**	1108.587	---	9
I	5	172.524	**690.345**	952.486	---	8
P	6	---	787.397	**839.402**	116.790	7
S#	7	---	954.396	**742.349**	-70.150	6
T	8	---	1055.443	575.351	---	5
V	9	---	1154.512	**474.303**	971.535	4
L	10	---	1267.596	**375.235**	-116.392	3
S	11	---	1354.628	**262.151**	-633.141	2
R	12	---	---	175.119	---	1

+2

Seq	#	b: Δ Error	b	y	y: Δ Error	+1
R	1	---	79.058	---	---	12
P	2	---	127.584	686.823	---	11
S#	3	---	211.083	**638.297**	120.715	10
R	4	1123.541	**289.134**	**554.797**	-482.077	9
I	5	-1287.998	**345.676**	476.747	---	8
P	6	1192.099	**394.202**	420.205	---	7
S#	7	-74.374	**477.702**	371.678	---	6
T	8	---	528.225	288.179	---	5
V	9	-164.942	**577.760**	237.655	---	4
L	10	---	634.302	188.121	---	3
S	11	---	677.818	131.579	---	2
R	12	---	---	88.063	---	1

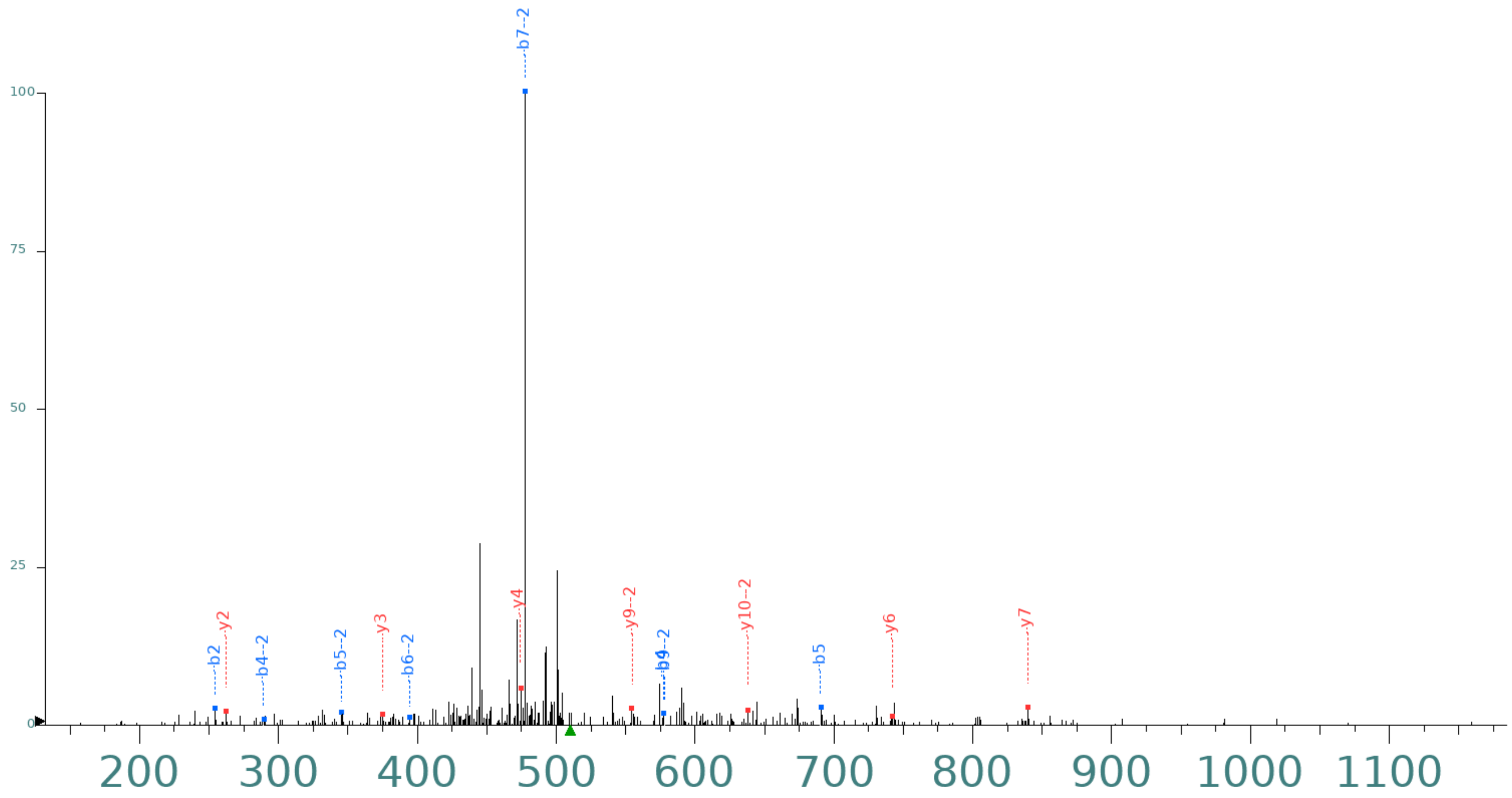

Predicted Fragmentation Pattern

+1

Seq	#	b: Δ Error	b	y	y: Δ Error	+1
R	1	---	157.108	---	---	12
P	2	-535.638	**254.161**	1372.639	---	11
S#	3	-1045.457	**421.160**	1275.586	---	10
R	4	232.028	**577.261**	1108.587	---	9
I	5	274.860	**690.345**	952.486	---	8
P	6	---	787.397	**839.402**	16.605	7
S#	7	---	954.396	742.349	---	6
T	8	---	1055.443	**575.351**	366.592	5
V	9	---	1154.512	**474.303**	-728.349	4
L	10	---	1267.596	**375.235**	-250.227	3
S	11	---	1354.628	262.151	---	2
R	12	---	---	175.119	---	1

+2

Seq	#	b: Δ Error	b	y	y: Δ Error	+1
R	1	---	79.058	---	---	12
P	2	---	127.584	**686.823**	-672.249	11
S#	3	---	211.083	**638.297**	639.643	10
R	4	---	289.134	**554.797**	882.642	9
I	5	-1170.380	**345.676**	**476.747**	743.067	8
P	6	-702.735	**394.202**	**420.205**	-275.896	7
S#	7	232.988	**477.702**	371.678	---	6
T	8	876.856	**528.225**	288.179	---	5
V	9	-632.142	**577.760**	237.655	---	4
L	10	---	634.302	188.121	---	3
S	11	---	677.818	131.579	---	2
R	12	---	---	88.063	---	1

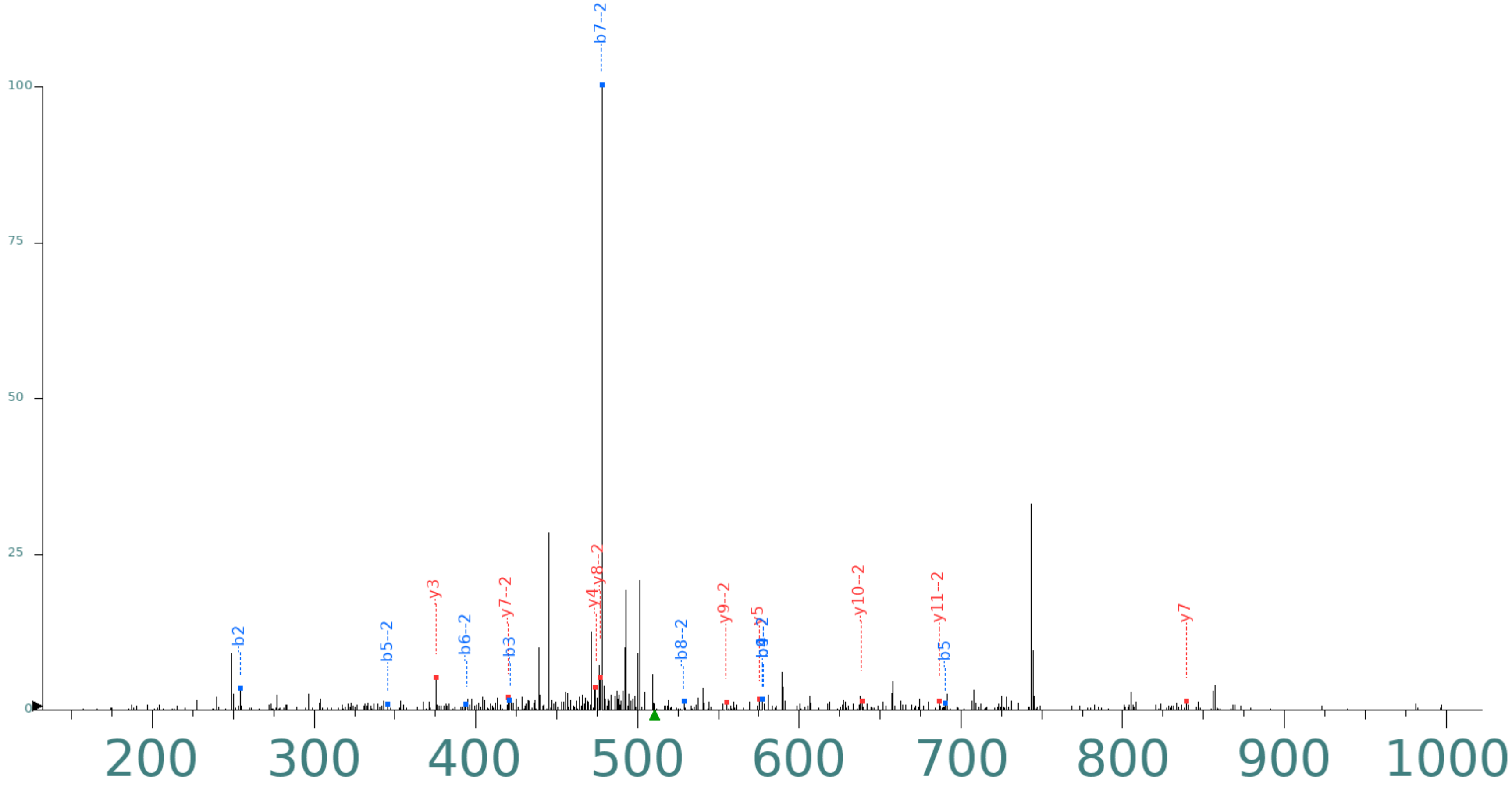

Predicted Fragmentation Pattern

+1

Seq	#	b: Δ Error	b	y	y: Δ Error	+1
R	1	---	157.108	---	---	12
P	2	-667.034	**254.161**	1372.639	---	11
S#	3	-721.420	**421.160**	1275.586	---	10
R	4	-680.323	**577.261**	1108.587	---	9
I	5	522.659	**690.345**	952.486	---	8
P	6	---	787.397	**839.402**	-125.127	7
S	7	---	874.429	**742.349**	326.537	6
T#	8	---	1055.443	**655.317**	660.530	5
V	9	---	1154.512	474.303	---	4
L	10	---	1267.596	**375.235**	-297.017	3
S	11	---	1354.628	262.151	---	2
R	12	---	---	175.119	---	1

+2

Seq	#	b: Δ Error	b	y	y: Δ Error	+1
R	1	---	79.058	---	---	12
P	2	---	127.584	686.823	---	11
S#	3	---	211.083	**638.297**	-294.739	10
R	4	---	289.134	**554.797**	2.654	9
I	5	-219.303	**345.676**	476.747	---	8
P	6	---	394.202	**420.205**	-411.508	7
S	7	---	437.718	371.678	---	6
T#	8	-782.018	**528.225**	**328.162**	-189.244	5
V	9	---	577.760	237.655	---	4
L	10	764.422	**634.302**	188.121	---	3
S	11	704.647	**677.818**	131.579	---	2
R	12	---	---	88.063	---	1

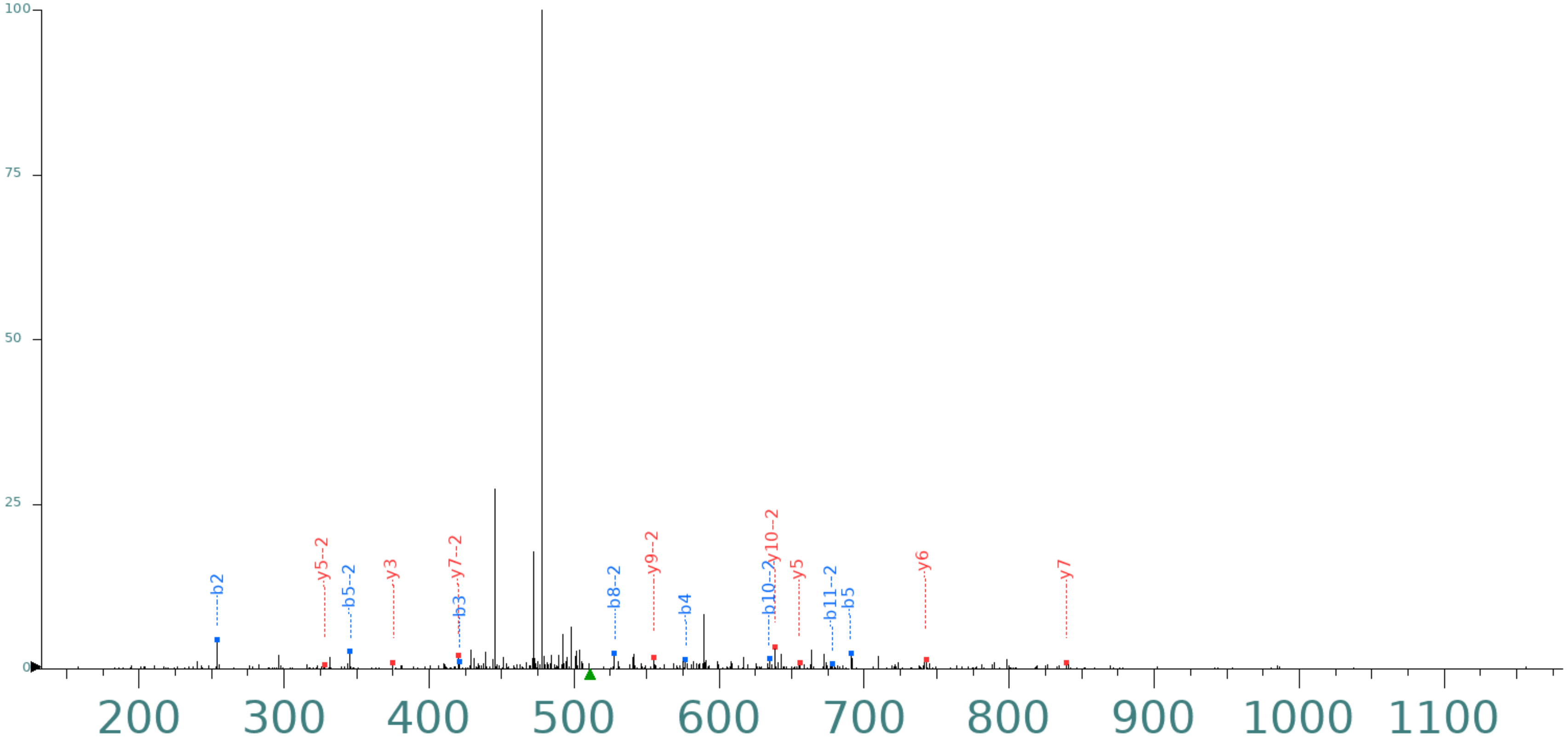

Predicted Fragmentation Pattern

Seq	#	b: Δ Error	b	y	y: Δ Error	+1
T	1	---	102.055	---	---	14
N	2	---	216.098	1434.674	---	13
V	3	-84.073	315.166	1320.631	21.430	12
L	4	---	428.250	1221.562	-130.196	11
S	5	8.925	515.282	1108.478	43.161	10
P	6	-806.943	612.335	1021.446	-32.349	9
H	7	165.199	749.394	924.393	119.635	8
T#	8	5.454	930.408	787.335	---	7
S	9	173.421	1017.440	606.321	156.797	6
G	10	138.116	1074.462	519.289	190.091	5
S	11	399.473	1161.494	462.267	-41.953	4
I	12	-15.819	1274.578	375.235	---	3
S	13	---	1361.610	262.151	-80.371	2
R	14	---	---	175.119	---	1

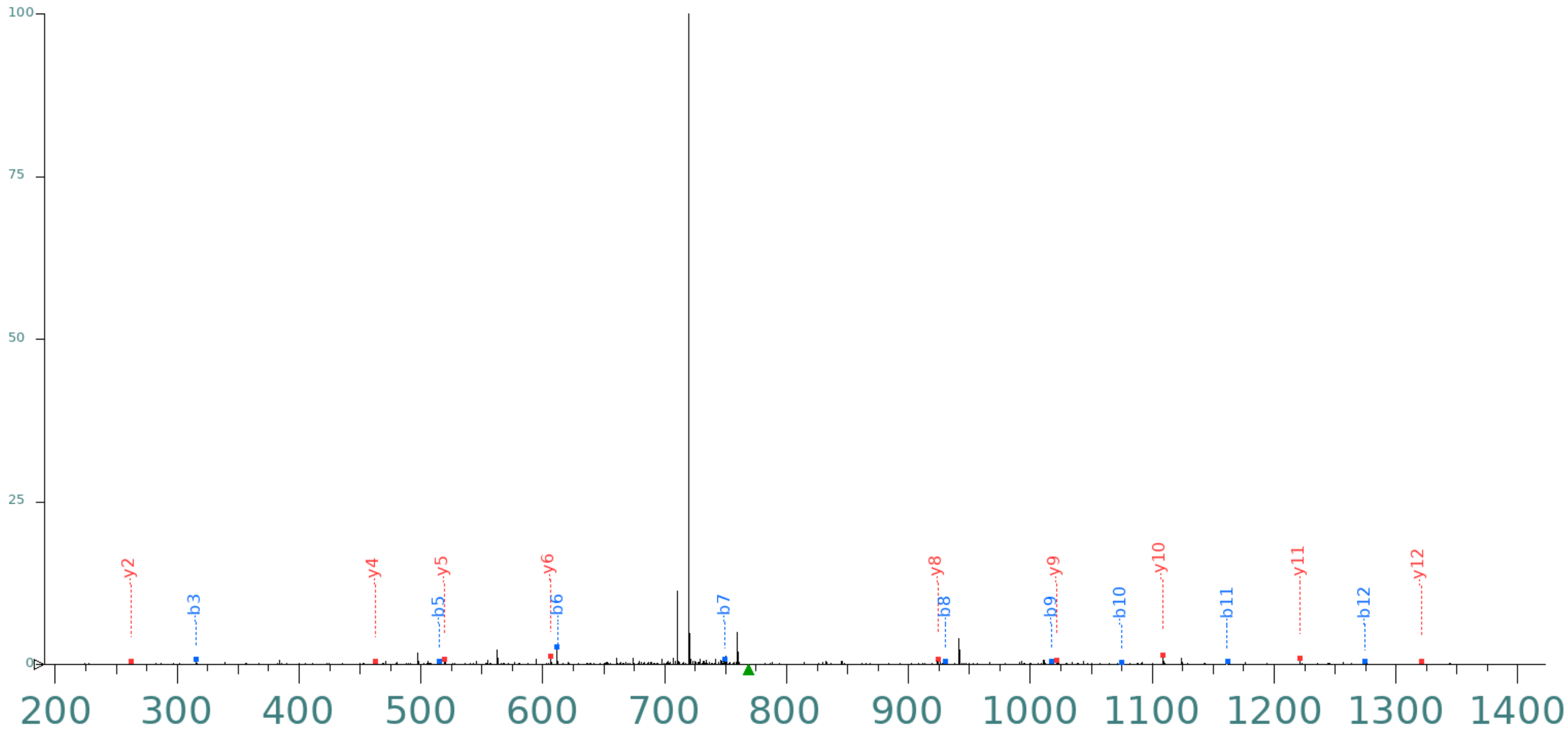

Predicted Fragmentation Pattern

Seq	#	b: Δ Error	b	y	y: Δ Error	+1
I	1	---	114.091	---	---	8
P	2	0.723	211.144	839.402	-76.254	7
S#	3	-659.157	378.142	742.349	-65.874	6
T	4	---	479.190	575.351	-97.716	5
V	5	136.757	578.259	474.303	185.866	4
L	6	36.066	691.343	375.235	-367.658	3
S	7	---	778.375	262.151	-456.118	2
R	8	---	---	175.119	-498.713	1

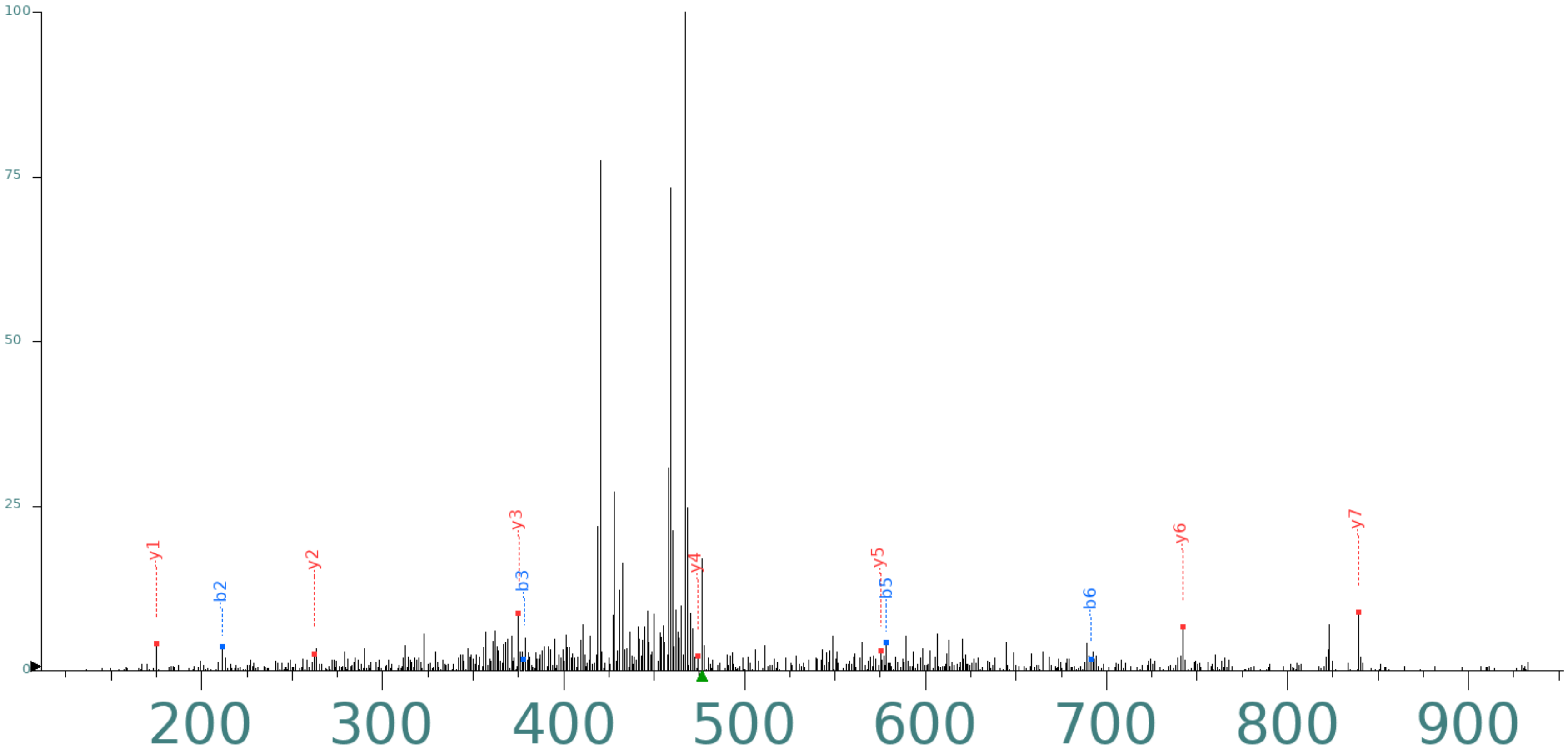

Predicted Fragmentation Pattern

+1

Seq	#	b: Δ Error	b	y	y: Δ Error	+1
R	1	---	157.108	---	---	12
P	2	-892.760	254.161	1372.639	---	11
S#	3	---	421.160	1275.586	---	10
R	4	230.442	577.261	1108.587	---	9
I	5	121.613	690.345	952.486	---	8
P	6	---	787.397	839.402	-183.388	7
S	7	-94.347	874.429	742.349	55.401	6
T	8	---	975.477	655.317	338.407	5
V	9	---	1074.546	554.270	---	4
L	10	---	1187.630	455.201	-942.066	3
S#	11	---	1354.628	342.117	---	2
R	12	---	---	175.119	-284.109	1

+2

Seq	#	b: Δ Error	b	y	y: Δ Error	+1
R	1	---	79.058	---	---	12
P	2	---	127.584	686.823	---	11
S#	3	-451.153	211.083	638.297	-206.436	10
R	4	-818.269	289.134	554.797	---	9
I	5	-1049.959	345.676	476.747	339.076	8
P	6	-385.139	394.202	420.205	496.233	7
S	7	-625.062	437.718	371.678	1133.001	6
T	8	-553.158	488.242	328.162	---	5
V	9	874.521	537.776	277.639	-718.221	4
L	10	---	594.318	228.104	-257.279	3
S#	11	---	677.818	171.562	---	2
R	12	---	---	88.063	---	1

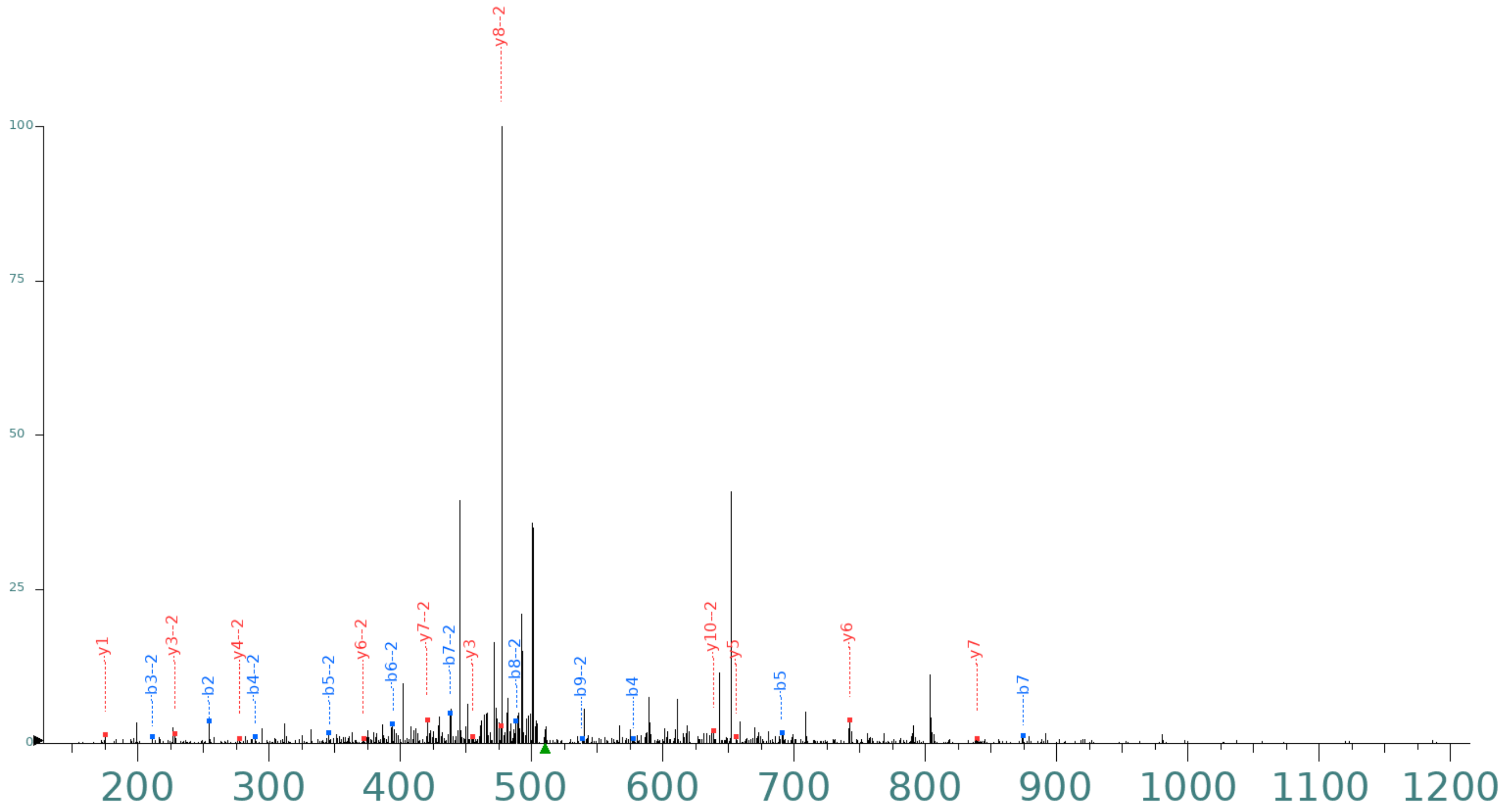

Predicted Fragmentation Pattern

Seq	#	b: Δ Error	b	y	y: Δ Error	+1
I	1	---	114.091	---	---	8
P	2	-177.519	211.144	839.402	56.594	7
S#	3	-302.507	378.142	742.349	102.751	6
T	4	---	479.190	575.351	-276.958	5
V	5	---	578.259	474.303	---	4
L	6	---	691.343	375.235	10.333	3
S	7	---	778.375	262.151	-263.317	2
R	8	---	---	175.119	155.103	1

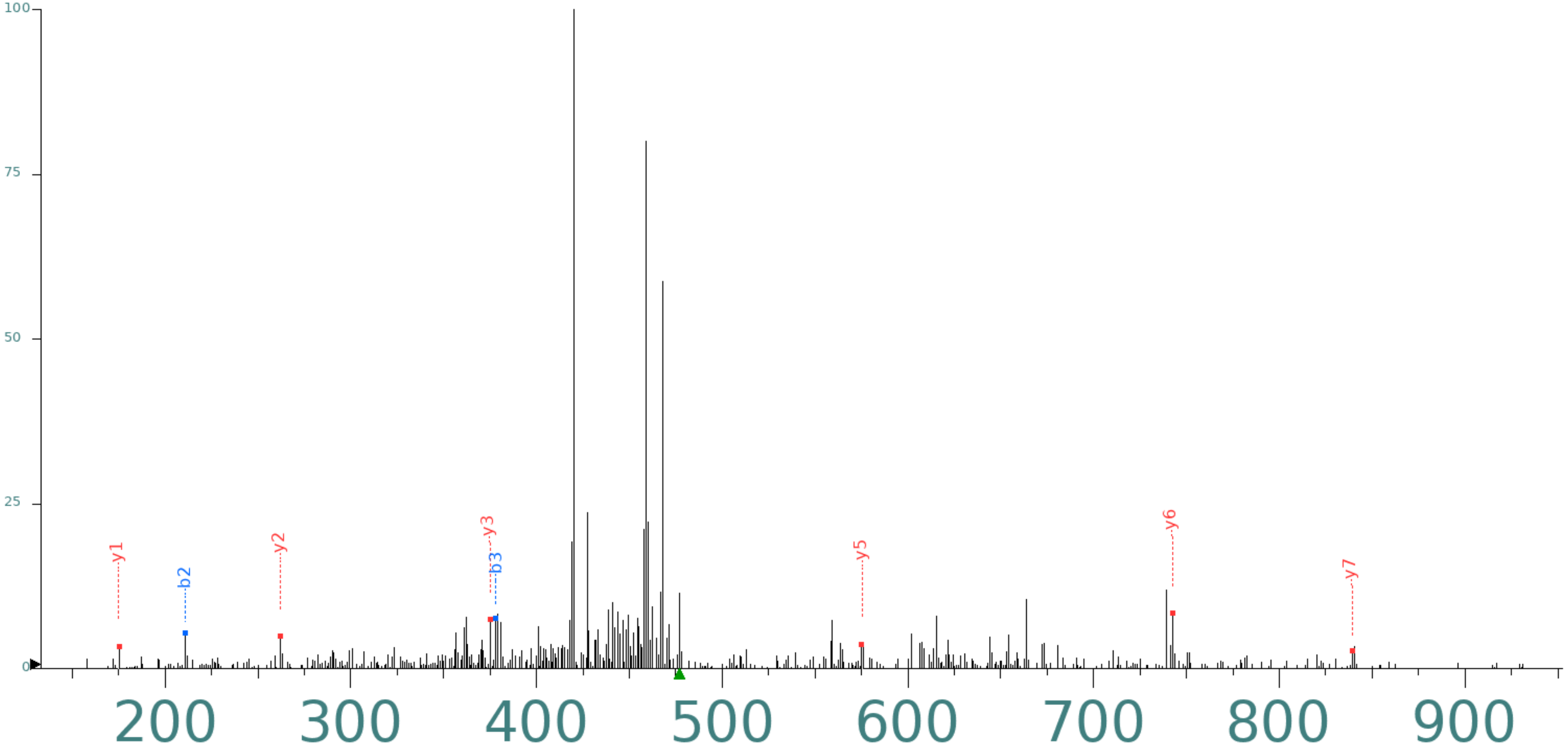

Predicted Fragmentation Pattern

Seq	#	b: Δ Error	b	y	y: Δ Error	+1
I	1	---	114.091	---	---	8
P	2	-247.069	211.144	839.402	---	7
S#	3	-589.827	378.142	742.349	-77.386	6
T	4	411.906	479.190	575.351	-265.813	5
V	5	77.239	578.259	474.303	---	4
L	6	190.971	691.343	375.235	-296.204	3
S	7	-244.571	778.375	262.151	-715.322	2
R	8	---	---	175.119	---	1

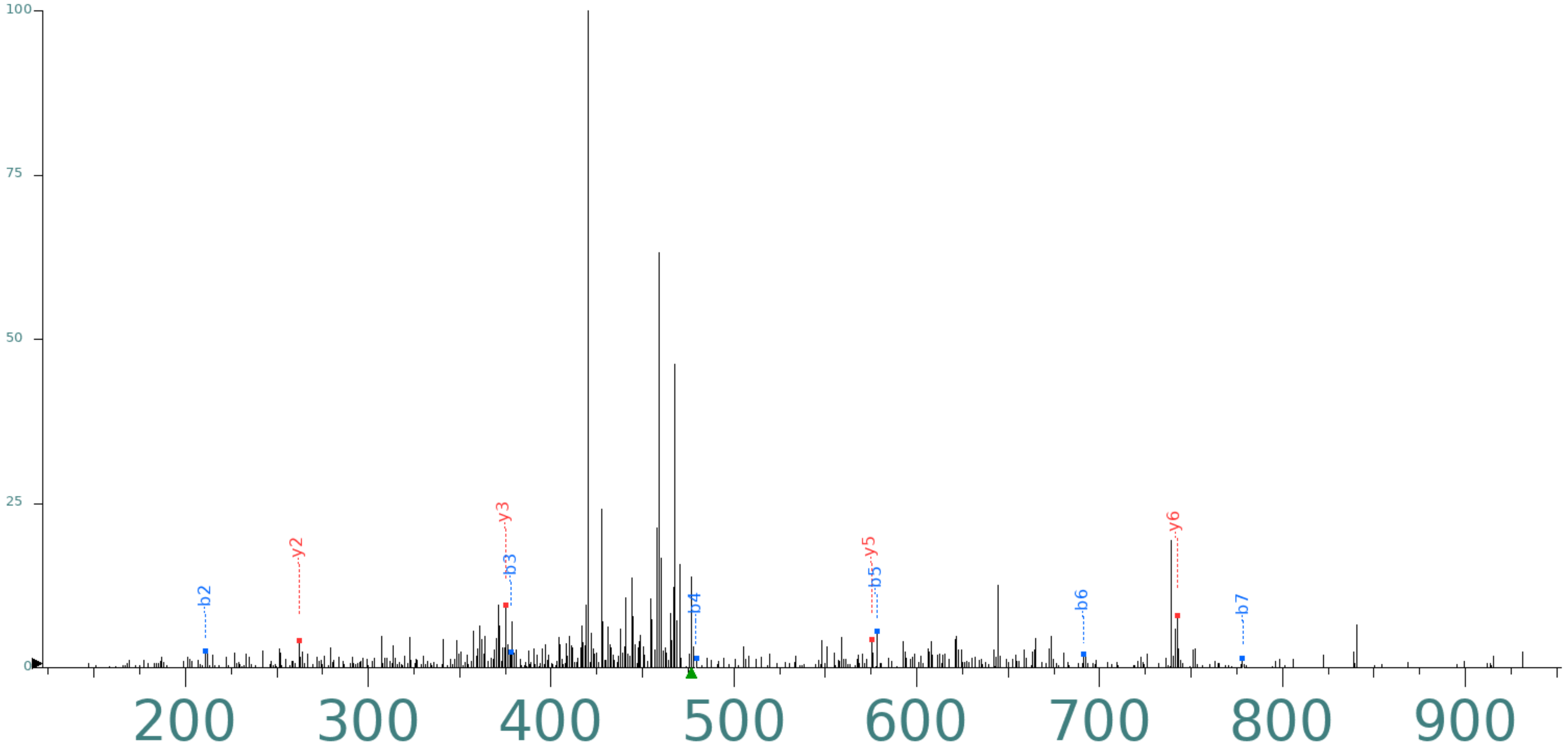

Predicted Fragmentation Pattern

+1

Seq	#	b: Δ Error	b	y	y: Δ Error	+1
R	1	-984.652	**157.108**	---	---	12
P	2	-810.491	**254.161**	1372.639	---	11
S#	3	-544.536	**421.160**	1275.586	---	10
R	4	-129.646	**577.261**	1108.587	---	9
I	5	-159.019	**690.345**	952.486	---	8
P	6	---	787.397	**839.402**	35.728	7
S	7	-159.627	**874.429**	**742.349**	153.221	6
T#	8	---	1055.443	655.317	---	5
V	9	---	1154.512	**474.303**	-235.272	4
L	10	---	1267.596	**375.235**	-419.343	3
S	11	---	1354.628	**262.151**	-265.414	2
R	12	---	---	**175.119**	-347.931	1

+2

Seq	#	b: Δ Error	b	y	y: Δ Error	+1
R	1	---	79.058	---	---	12
P	2	---	127.584	686.823	---	11
S#	3	---	211.083	**638.297**	256.160	10
R	4	-717.527	**289.134**	**554.797**	-166.354	9
I	5	---	345.676	476.747	---	8
P	6	-452.702	**394.202**	**420.205**	-57.801	7
S	7	-1074.541	**437.718**	**371.678**	1246.285	6
T#	8	---	528.225	328.162	---	5
V	9	626.367	**577.760**	237.655	---	4
L	10	---	634.302	188.121	---	3
S	11	-615.678	**677.818**	131.579	---	2
R	12	---	---	88.063	---	1

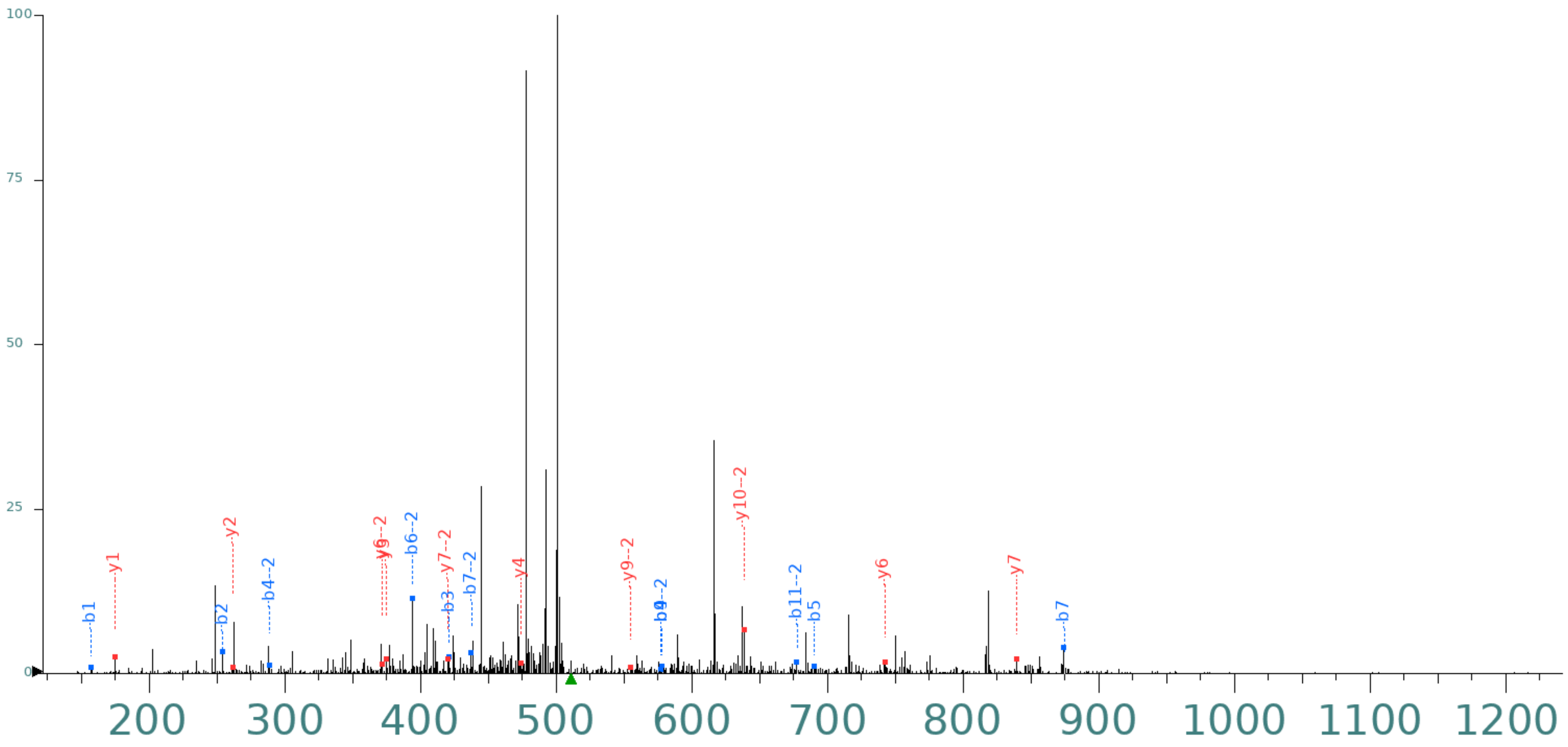

Predicted Fragmentation Pattern

+1

Seq	#	b: Δ Error	b	y	y: Δ Error	+1
R	1	---	157.108	---	---	12
P	2	193.807	**254.161**	1372.639	---	11
S#	3	-1171.382	**421.160**	1275.586	---	10
R	4	---	577.261	1108.587	---	9
I	5	-328.412	**690.345**	952.486	---	8
P	6	---	787.397	**839.402**	574.999	7
S#	7	---	954.396	**742.349**	198.507	6
T	8	---	1055.443	**575.351**	90.902	5
V	9	---	1154.512	**474.303**	-285.485	4
L	10	---	1267.596	375.235	---	3
S	11	---	1354.628	262.151	---	2
R	12	---	---	175.119	---	1

+2

Seq	#	b: Δ Error	b	y	y: Δ Error	+1
R	1	---	79.058	---	---	12
P	2	---	127.584	686.823	---	11
S#	3	---	211.083	**638.297**	96.337	10
R	4	---	289.134	554.797	---	9
I	5	28.292	**345.676**	**476.747**	526.214	8
P	6	---	394.202	**420.205**	1098.209	7
S#	7	128.255	**477.702**	**371.678**	360.703	6
T	8	---	528.225	288.179	---	5
V	9	---	577.760	237.655	---	4
L	10	---	634.302	188.121	---	3
S	11	---	677.818	131.579	---	2
R	12	---	---	88.063	---	1

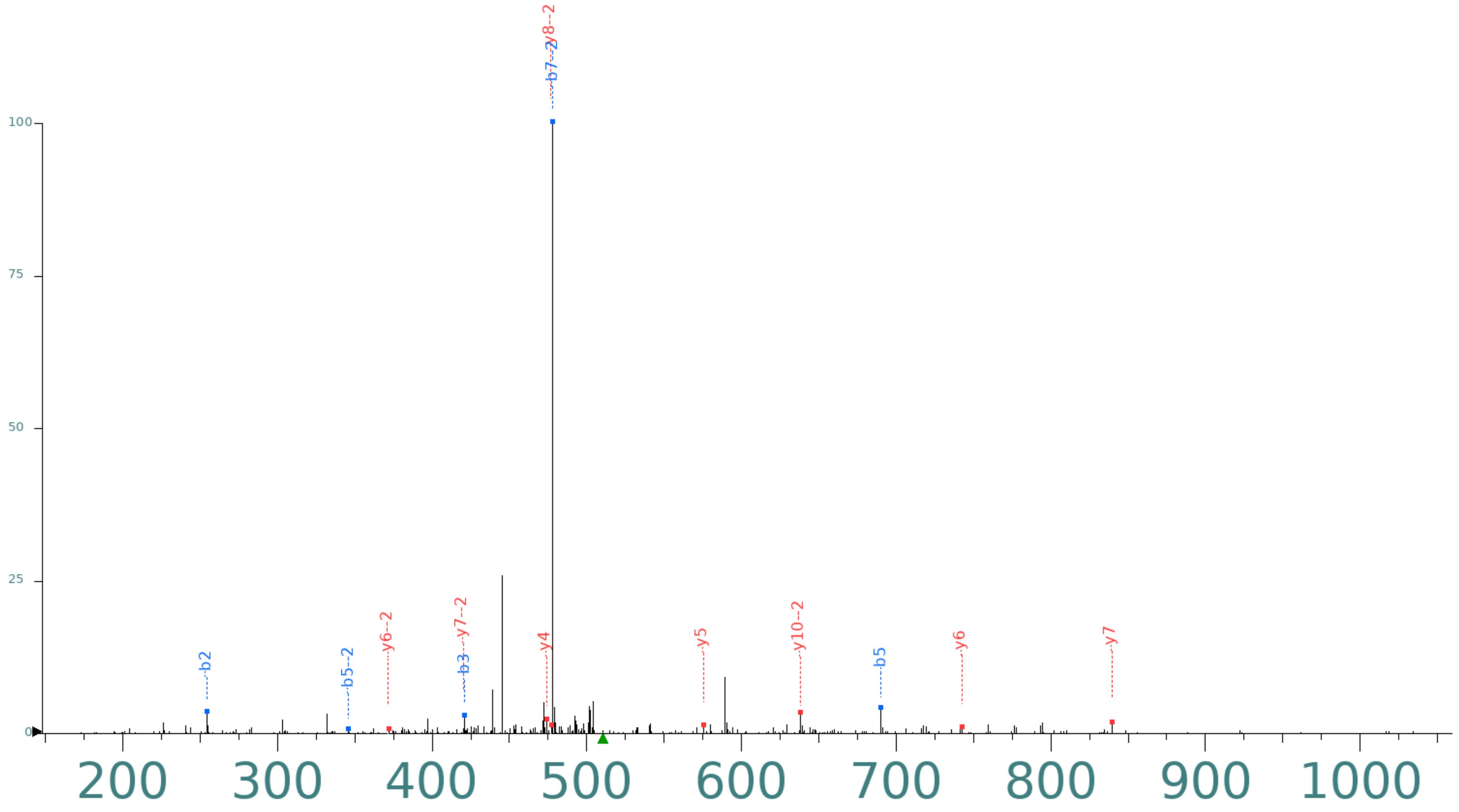

Predicted Fragmentation Pattern

+1

Seq	#	b: Δ Error	b	y	y: Δ Error	+1
R	1	---	157.108	---	---	12
P	2	-821.195	**254.161**	1372.639	---	11
S#	3	-811.119	**421.160**	1275.586	---	10
R	4	-489.782	**577.261**	1108.587	---	9
I	5	-306.383	**690.345**	952.486	---	8
P	6	---	787.397	**839.402**	-98.654	7
S#	7	---	954.396	742.349	---	6
T	8	---	1055.443	**575.351**	7.210	5
V	9	---	1154.512	**474.303**	-924.787	4
L	10	---	1267.596	**375.235**	-362.693	3
S	11	---	1354.628	**262.151**	-469.052	2
R	12	---	---	175.119	---	1

+2

Seq	#	b: Δ Error	b	y	y: Δ Error	+1
R	1	---	79.058	---	---	12
P	2	---	127.584	**686.823**	-167.955	11
S#	3	---	211.083	**638.297**	215.445	10
R	4	---	289.134	554.797	---	9
I	5	-1220.379	**345.676**	**476.747**	-608.815	8
P	6	152.718	**394.202**	**420.205**	-520.548	7
S#	7	-38.084	**477.702**	371.678	---	6
T	8	---	528.225	288.179	---	5
V	9	---	577.760	237.655	---	4
L	10	242.066	**634.302**	188.121	---	3
S	11	---	677.818	131.579	---	2
R	12	---	---	88.063	---	1

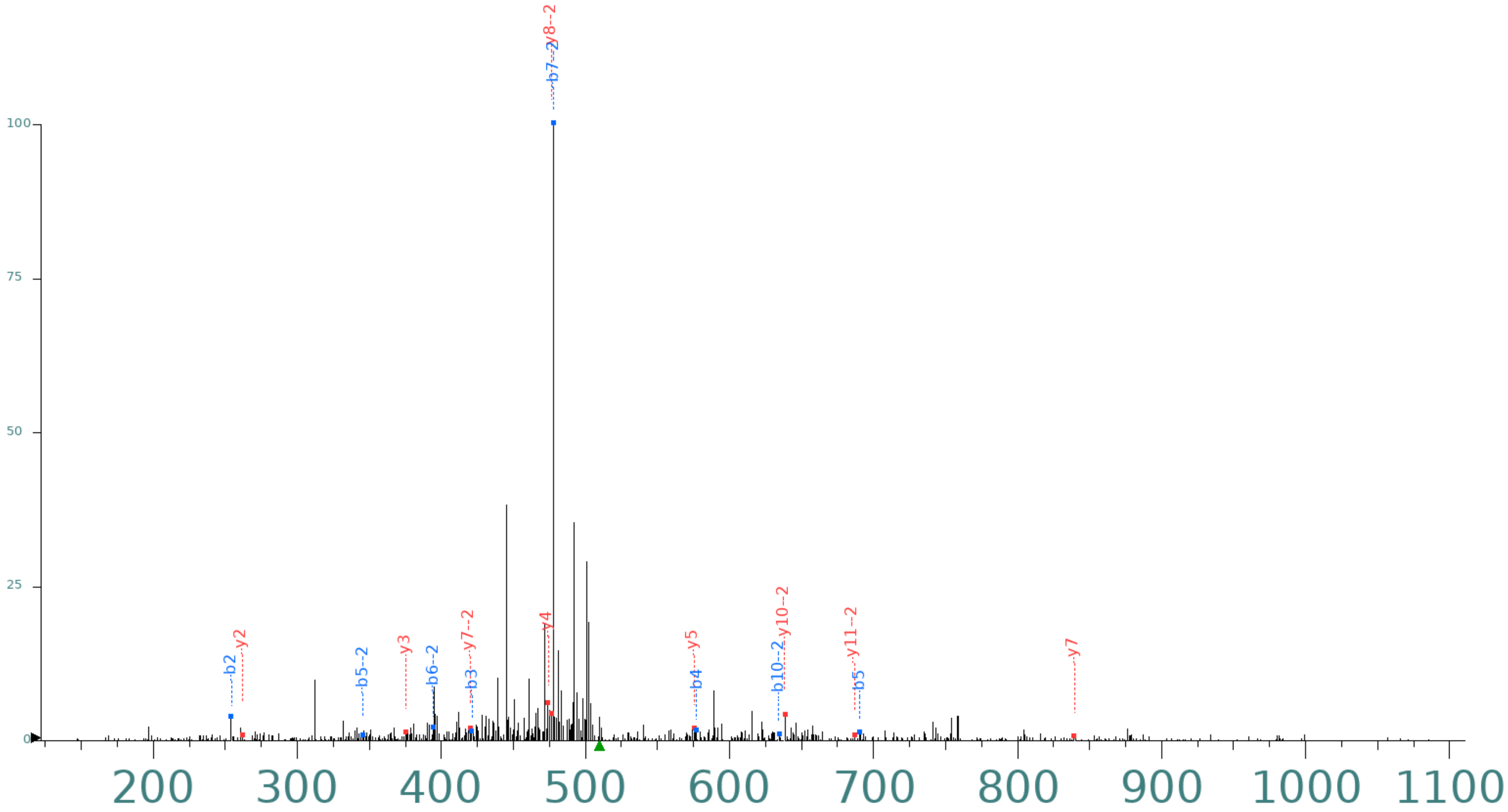

Predicted Fragmentation Pattern

Seq	#	b: Δ Error	b	y	y: Δ Error	+1
I	1	---	114.091	---	---	8
P	2	---	211.144	839.402	80.150	7
S	3	---	298.176	742.349	2.455	6
T	4	---	399.224	655.317	---	5
V	5	444.215	498.292	554.270	---	4
L	6	---	611.376	455.201	-366.228	3
S#	7	-173.028	778.375	342.117	140.290	2
R	8	---	---	175.119	216.771	1

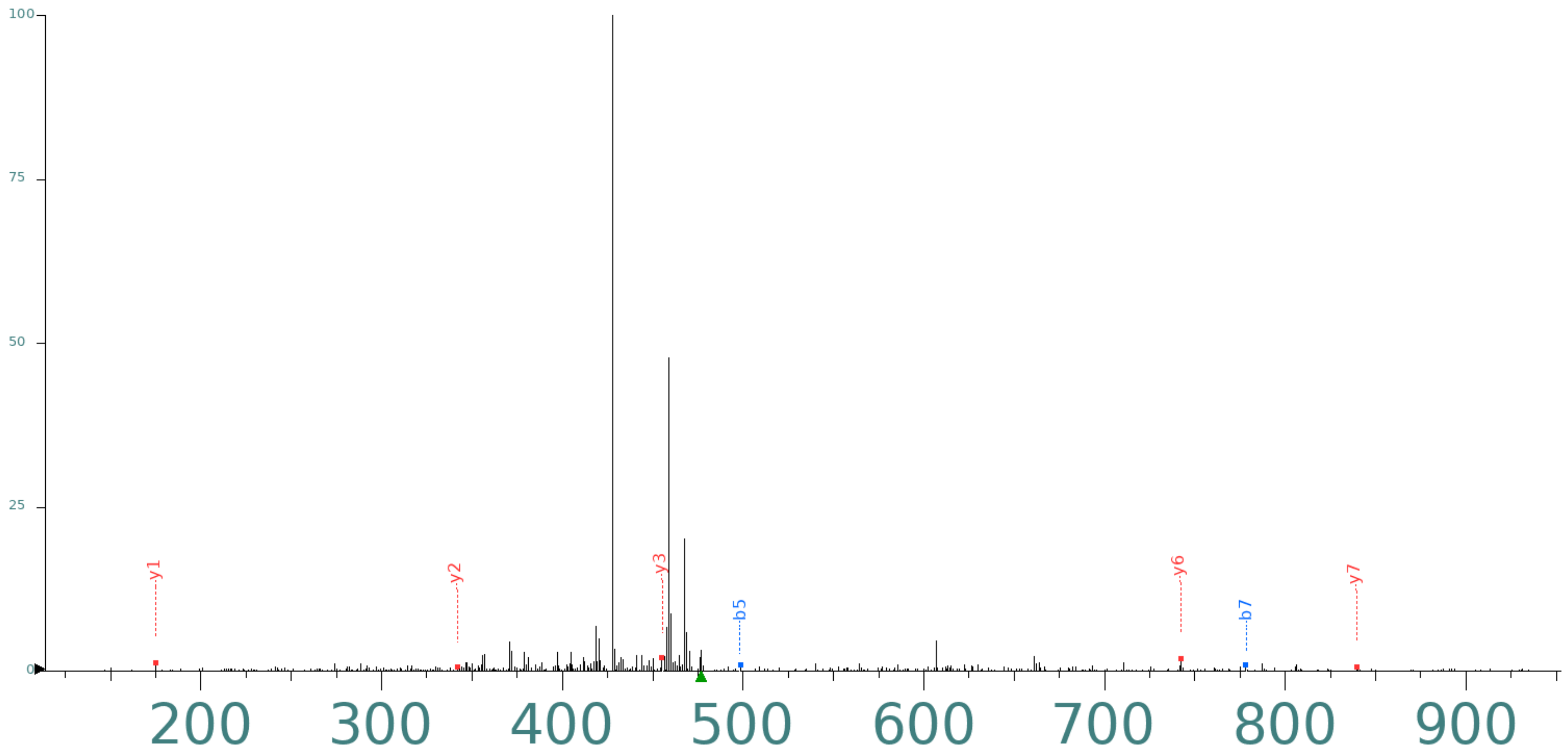

052205

LPA3-PMA

Predicted Fragmentation Pattern

Seq	#	b: Δ Error	b	y	y: Δ Error	+1
S#	1	---	168.006	---	---	20
D	2	---	283.033	2071.924	---	19
T	3	306.461	**384.080**	1956.897	---	18
G	4	---	441.102	1855.849	---	17
S	5	---	528.134	**1798.828**	209.325	16
Q	6	---	656.192	**1711.796**	44.328	15
Y	7	594.561	**819.256**	**1583.737**	80.887	14
I	8	78.816	**932.340**	**1420.674**	129.067	13
E	9	147.664	**1061.382**	**1307.590**	93.528	12
D	10	74.123	**1176.409**	**1178.547**	57.572	11
S	11	247.763	**1263.441**	**1063.520**	77.652	10
I	12	301.010	**1376.525**	**976.488**	70.121	9
S	13	---	1463.557	**863.404**	86.747	8
Q	14	66.039	**1591.616**	**776.372**	230.605	7
G	15	248.226	**1648.637**	**648.313**	25.204	6
A	16	150.624	**1719.675**	**591.292**	555.588	5
V	17	109.310	**1818.743**	**520.255**	-42.887	4
C	18	96.950	**1978.774**	**421.186**	-161.408	3
N	19	---	2092.817	261.156	---	2
K	20	---	---	147.113	---	1

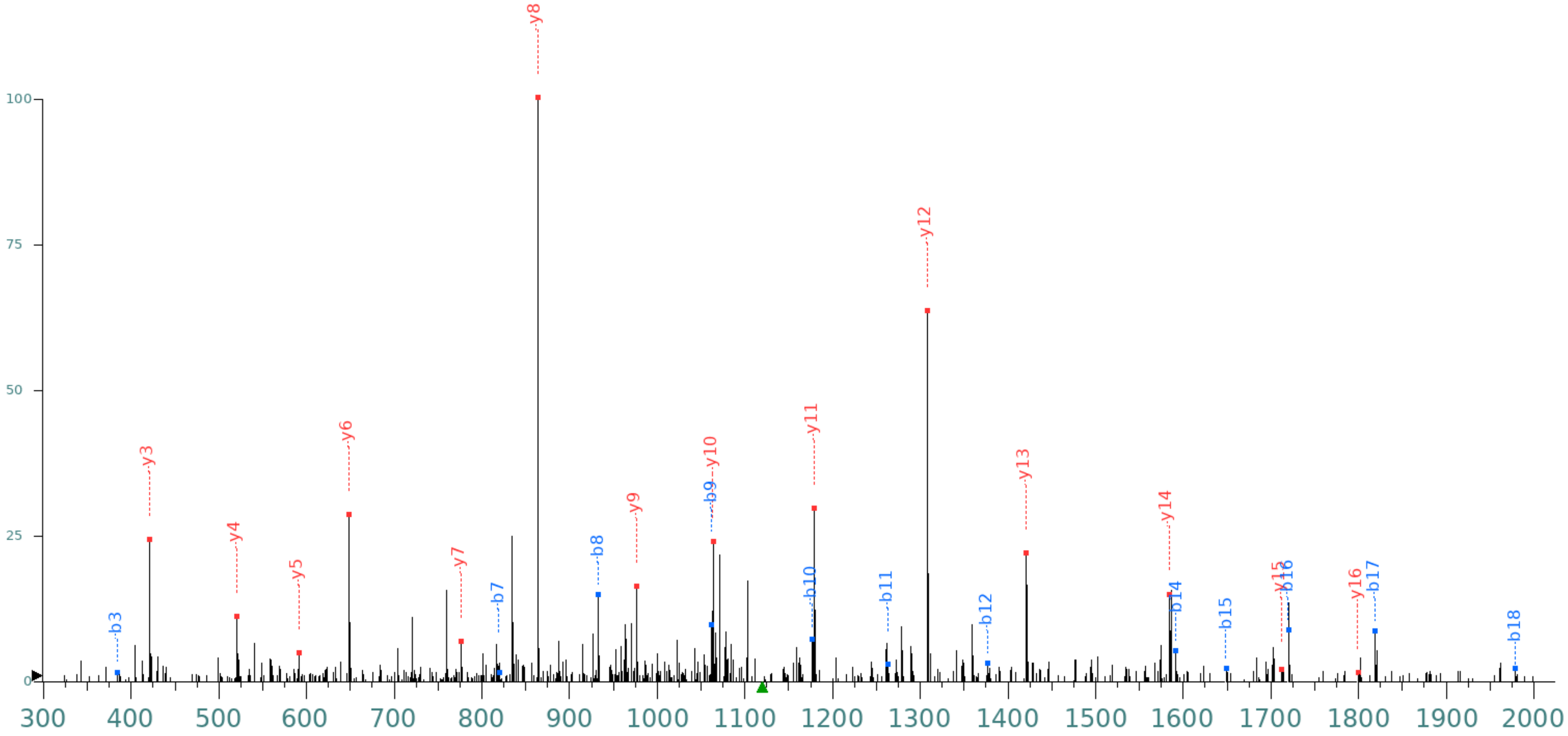

Predicted Fragmentation Pattern

Seq	#	b: Δ Error	b	y	y: Δ Error	+1
T	1	---	102.055	---	---	14
N	2	---	216.098	1434.674	---	13
V	3	216.157	315.166	1320.631	264.645	12
L	4	617.538	428.250	1221.562	261.576	11
S	5	330.053	515.282	1108.478	138.731	10
P	6	-135.092	612.335	1021.446	67.496	9
H	7	187.833	749.394	924.393	225.638	8
T	8	78.831	850.442	787.335	282.046	7
S	9	300.097	937.474	686.287	518.002	6
G	10	121.202	994.495	599.255	199.310	5
S#	11	---	1161.494	542.233	---	4
I	12	220.022	1274.578	375.235	209.952	3
S	13	240.249	1361.610	262.151	796.400	2
R	14	---	---	175.119	---	1

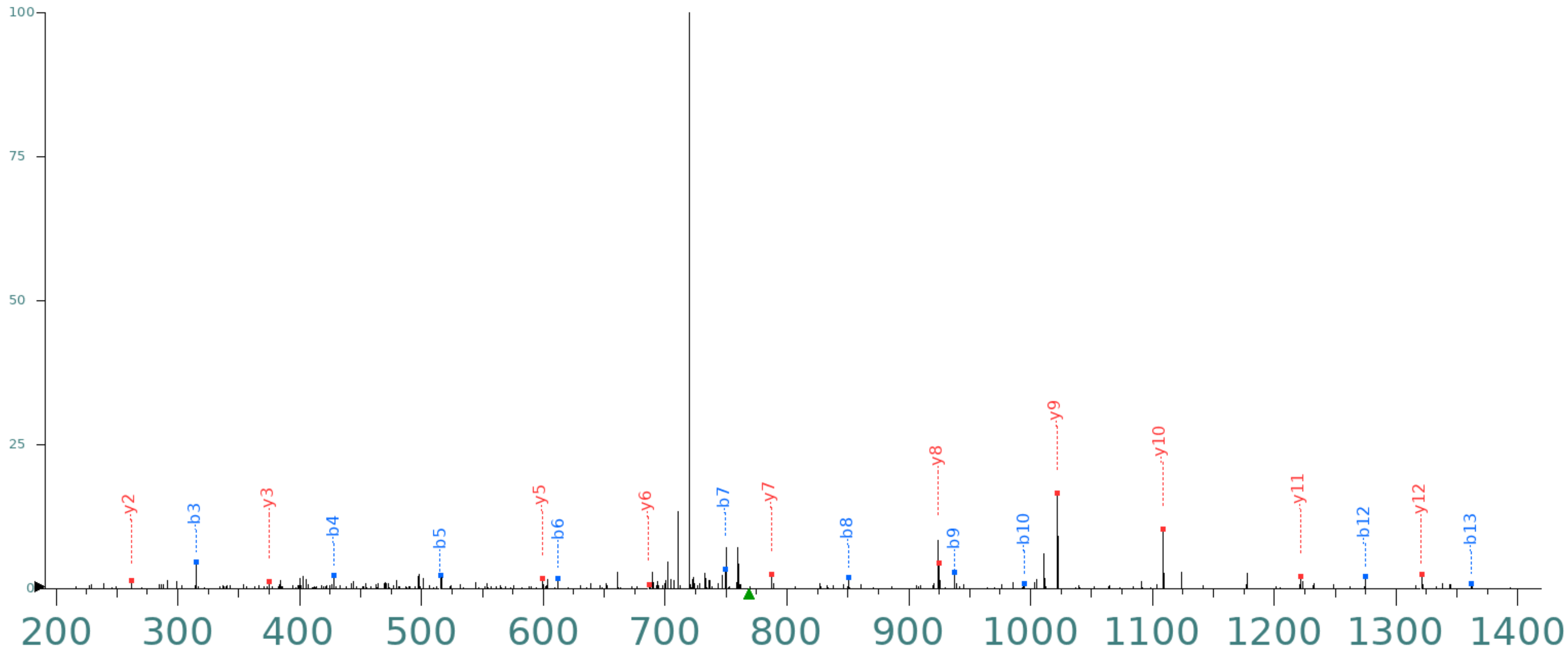

Predicted Fragmentation Pattern

+1

Seq	#	b: Δ Error	b	y	y: Δ Error	+1
S#	1	---	168.006	---	---	20
D	2	---	283.033	2071.924	---	19
T	3	219.423	**384.080**	1956.897	---	18
G	4	---	441.102	1855.849	---	17
S	5	---	528.134	1798.828	---	16
Q	6	328.783	**656.192**	1711.796	---	15
Y	7	209.330	**819.256**	1583.737	---	14
I	8	323.555	**932.340**	1420.674	---	13
E	9	198.136	**1061.382**	1307.590	---	12
D	10	---	1176.409	**1178.547**	8.894	11
S	11	308.019	**1263.441**	**1063.520**	56.995	10
I	12	180.463	**1376.525**	976.488	---	9
S	13	---	1463.557	**863.404**	157.916	8
Q	14	---	1591.616	**776.372**	540.112	7
G	15	---	1648.637	**648.313**	-11.700	6
A	16	-0.053	**1719.675**	**591.292**	-121.263	5
V	17	---	1818.743	**520.255**	23.164	4
C	18	---	1978.774	**421.186**	-162.785	3
N	19	---	2092.817	**261.156**	-372.561	2
K	20	---	---	147.113	---	1

+2

Seq	#	b: Δ Error	b	y	y: Δ Error	+1
S#	1	---	84.506	---	---	20
D	2	---	142.020	1036.465	---	19
T	3	---	192.544	978.952	---	18
G	4	---	221.054	**928.428**	-308.165	17
S	5	---	264.571	**899.917**	270.247	16
Q	6	-1020.238	**328.600**	856.401	---	15
Y	7	393.630	**410.131**	**792.372**	173.738	14
I	8	---	466.673	**710.840**	579.987	13
E	9	396.770	**531.195**	**654.298**	-74.974	12
D	10	---	588.708	**589.777**	753.167	11
S	11	-312.463	**632.224**	**532.264**	715.834	10
I	12	-370.127	**688.766**	**488.748**	-890.648	9
S	13	412.356	**732.282**	**432.206**	70.606	8
Q	14	303.087	**796.312**	388.690	---	7
G	15	19.437	**824.822**	324.660	---	6
A	16	368.033	**860.341**	296.150	---	5
V	17	-134.972	**909.875**	**260.631**	1637.321	4
C	18	---	989.890	211.097	---	3
N	19	---	1046.912	131.082	---	2
K	20	---	---	74.060	---	1

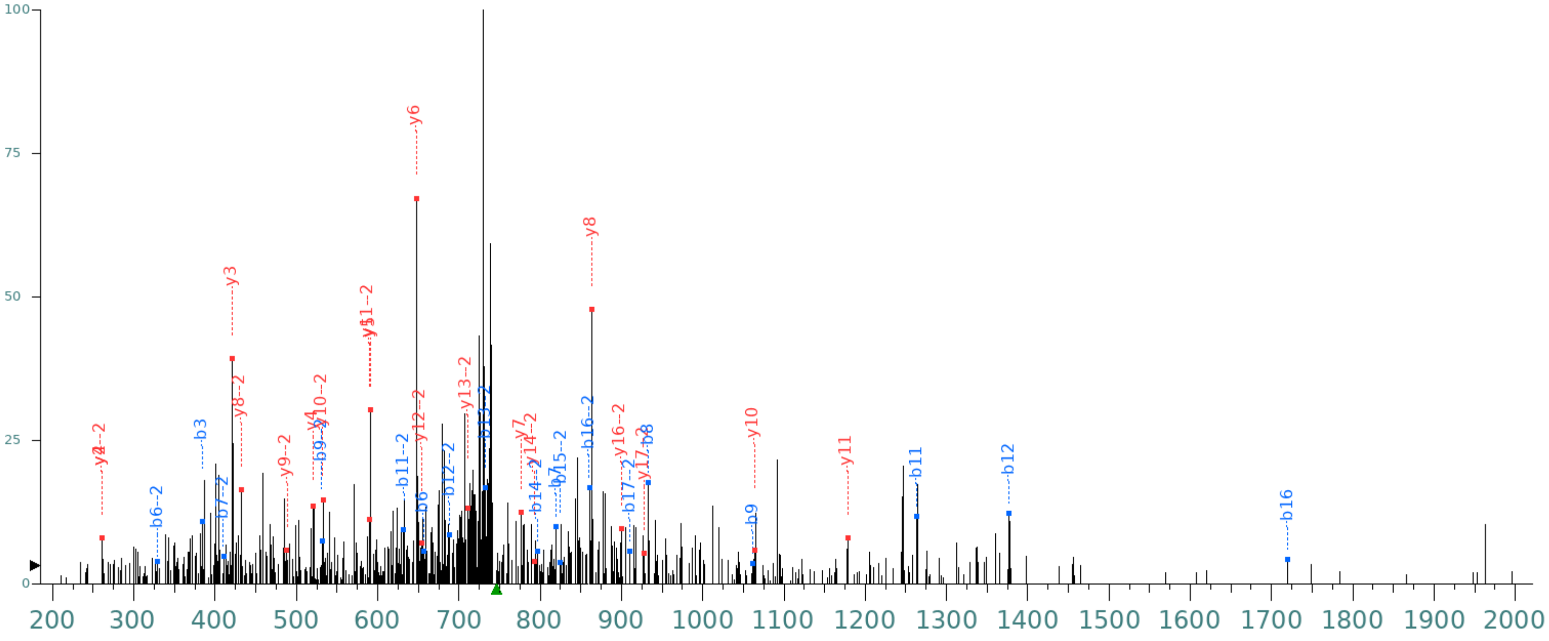

Predicted Fragmentation Pattern

Seq	#	b: Δ Error	b	y	y: Δ Error	+1
T	1	---	102.055	---	---	14
N	2	416.585	**216.098**	1434.674	---	13
V	3	-94.436	**315.166**	1320.631	---	12
L	4	365.664	**428.250**	**1221.562**	6.824	11
S	5	152.937	**515.282**	**1108.478**	92.820	10
P	6	118.687	**612.335**	**1021.446**	101.012	9
H	7	33.445	**749.394**	**924.393**	372.725	8
T	8	325.186	**850.442**	**787.335**	152.022	7
S	9	---	937.474	**686.287**	251.935	6
G	10	342.655	**994.495**	599.255	---	5
S#	11	---	1161.494	**542.233**	510.875	4
I	12	---	1274.578	**375.235**	2.769	3
S	13	---	1361.610	**262.151**	569.241	2
R	14	---	---	175.119	---	1

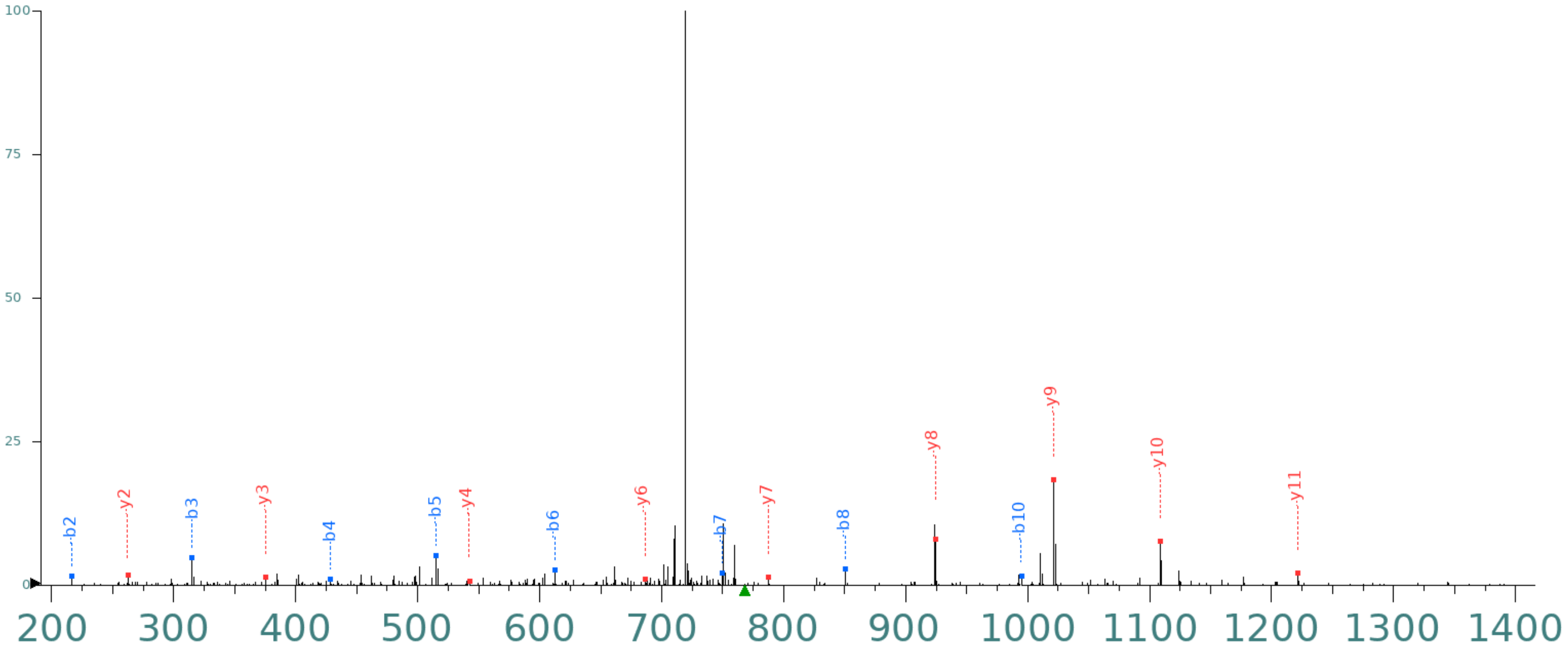

Predicted Fragmentation Pattern

Seq	#	b: Δ Error	b	y	y: Δ Error	+1
T	1	---	102.055	---	---	[illegible]
N	2	---	216.098	1434.674	---	[illegible]
V	3	223.900	**315.166**	1320.631	---	[illegible]
L	4	-238.125	**428.250**	**1221.562**	317.205	[illegible]
S	5	480.125	**515.282**	**1108.478**	55.713	[illegible]
P	6	-590.743	**612.335**	**1021.446**	139.305	[illegible]
H	7	39.879	**749.394**	**924.393**	352.733	[illegible]
T	8	96.411	**850.442**	**787.335**	438.449	[illegible]
S#	9	---	1017.440	686.287	---	[illegible]
G	10	---	1074.462	**519.289**	470.817	[illegible]
S	11	---	1161.494	**462.267**	12.711	[illegible]
I	12	---	1274.578	**375.235**	226.292	[illegible]
S	13	---	1361.610	262.151	---	[illegible]
R	14	---	---	175.119	---	[illegible]

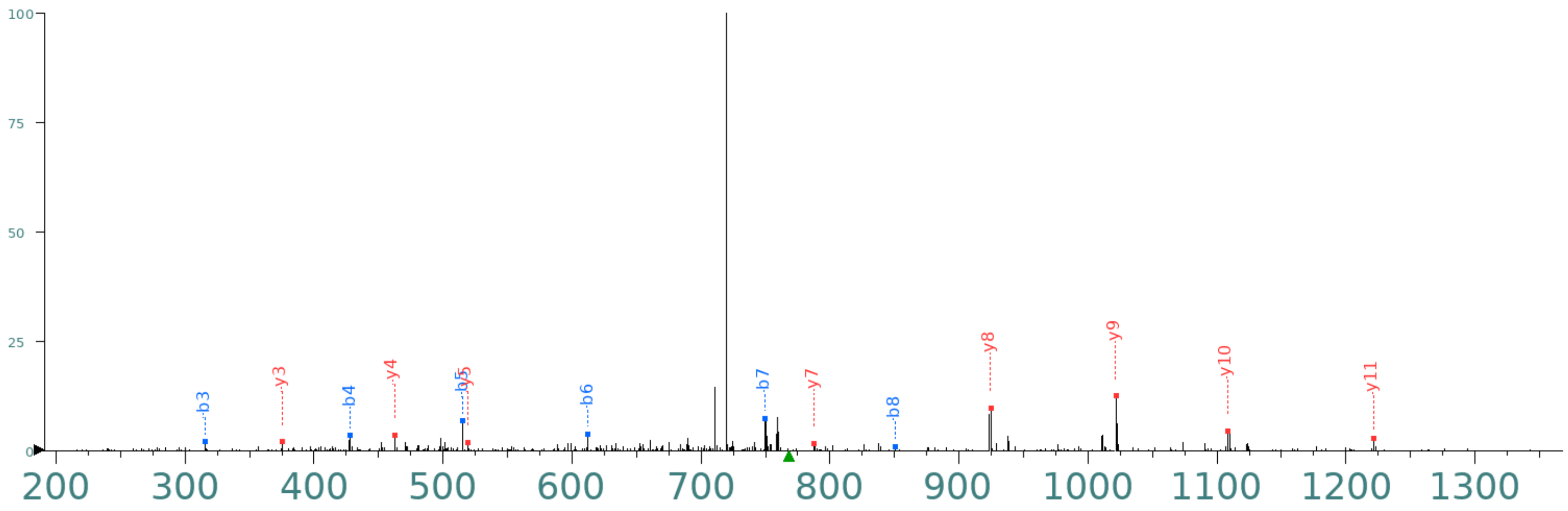

Predicted Fragmentation Pattern

Seq	#	b: Δ Error	b	y	y: Δ Error	+1
I	1	---	114.091	---	---	8
P	2	-255.312	211.144	839.402	271.244	7
S#	3	348.260	378.142	742.349	33.615	6
T	4	---	479.190	575.351	121.341	5
V	5	235.092	578.259	474.303	---	4
L	6	320.860	691.343	375.235	413.555	3
S	7	---	778.375	262.151	890.070	2
R	8	---	---	175.119	948.886	1

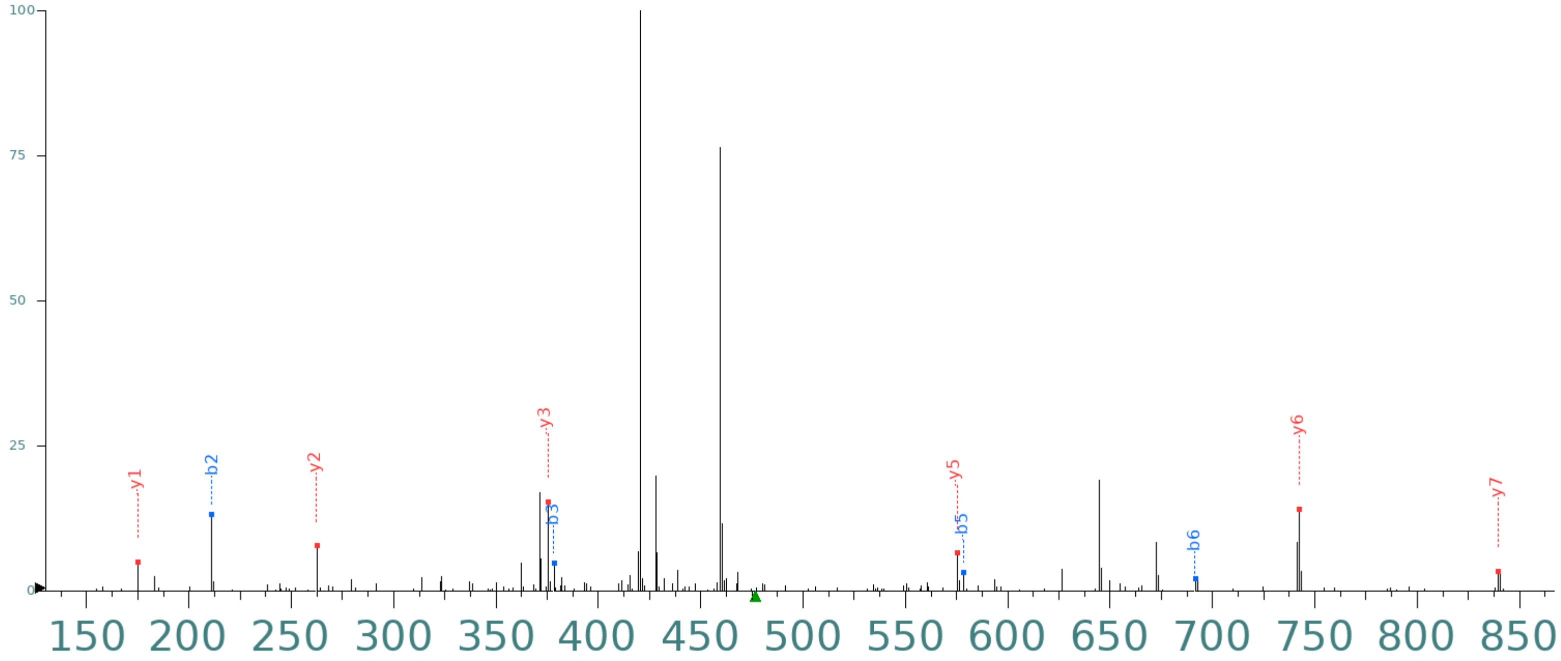

Predicted Fragmentation Pattern

Seq	#	b: Δ Error	b	y	y: Δ Error	+1
I	1	---	114.091	---	---	8
P	2	299.386	211.144	839.402	327.636	7
S#	3	374.470	378.142	742.349	309.118	6
T	4	---	479.190	575.351	371.680	5
V	5	-127.116	578.259	474.303	---	4
L	6	295.802	691.343	375.235	712.184	3
S	7	-24.875	778.375	262.151	81.209	2
R	8	---	---	175.119	303.337	1

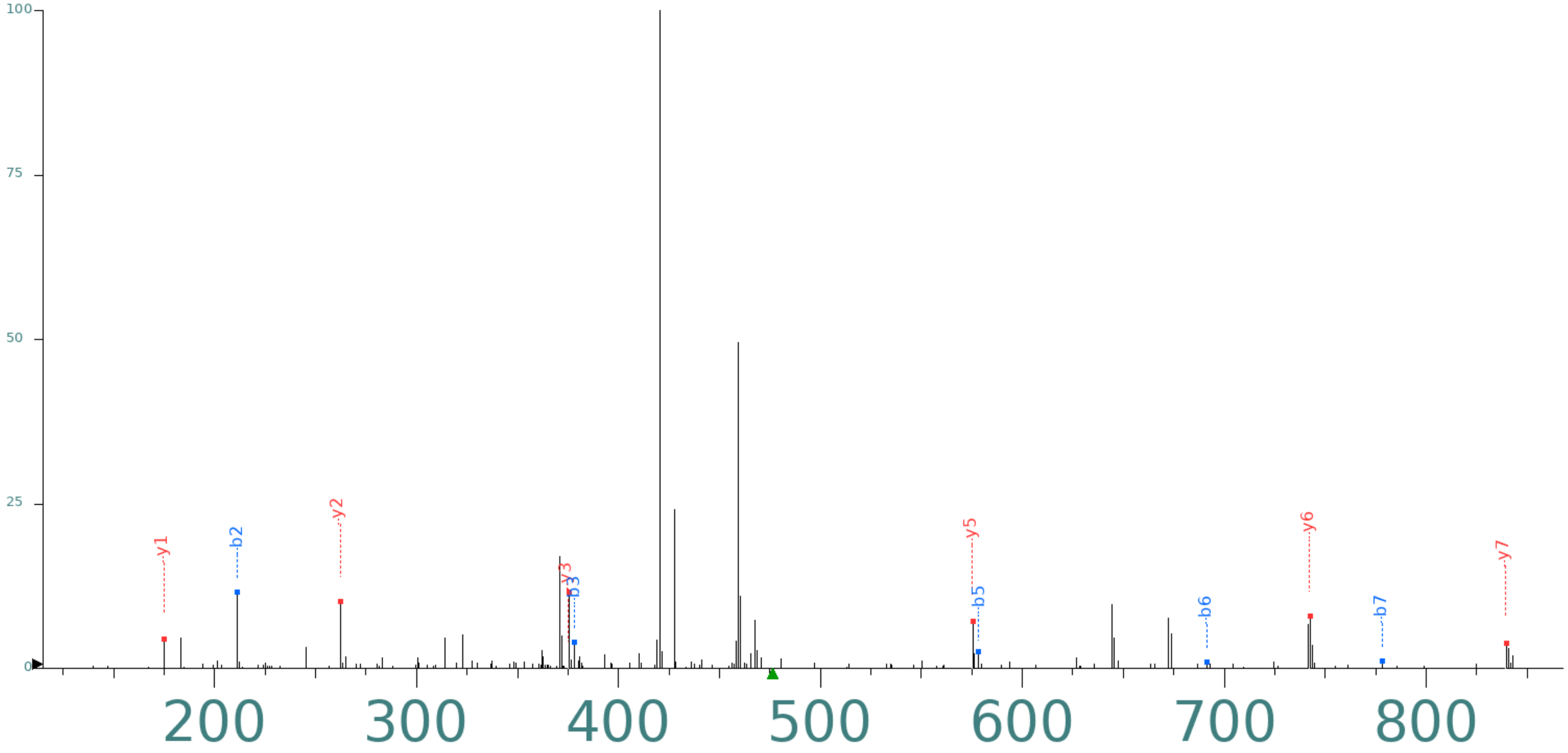

Predicted Fragmentation Pattern

Seq	#	b: Δ Error	b	y	y: Δ Error	+1
T	1	---	102.055	---	---	14
N	2	143.334	**216.098**	1434.674	---	13
V	3	193.121	**315.166**	1320.631	---	12
L	4	242.386	**428.250**	**1221.562**	-25.753	11
S#	5	---	595.249	**1108.478**	-49.895	10
P	6	---	692.301	**941.480**	131.196	9
H	7	320.196	**829.360**	**844.427**	126.725	8
T	8	---	930.408	**707.368**	26.980	7
S	9	---	1017.440	**606.321**	-76.928	6
G	10	---	1074.462	**519.289**	292.533	5
S	11	---	1161.494	462.267	---	4
I	12	325.889	**1274.578**	**375.235**	287.583	3
S	13	---	1361.610	**262.151**	618.076	2
R	14	---	---	175.119	---	1

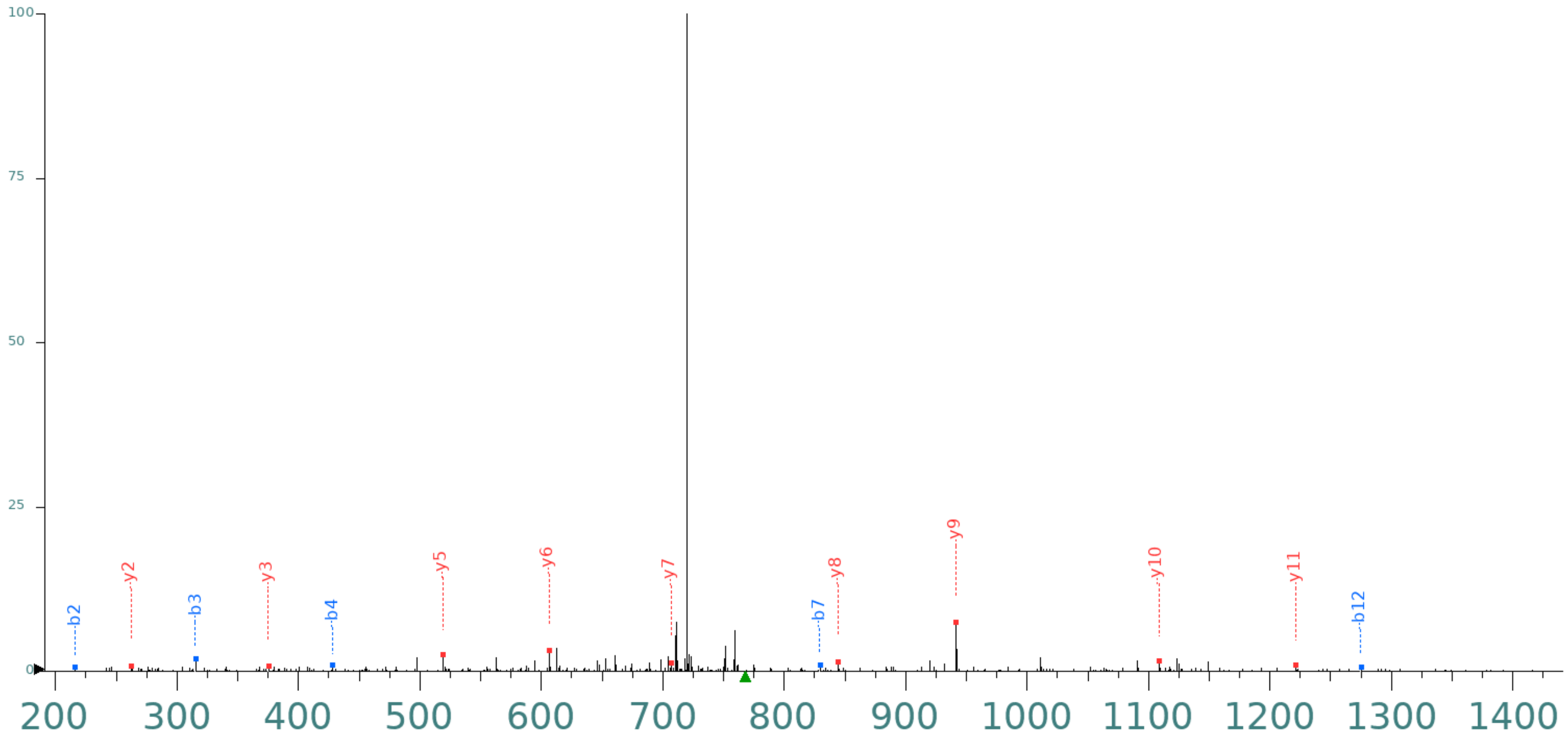

Predicted Fragmentation Pattern

Seq	#	b: Δ Error	b	y	y: Δ Error	+1
R	1	---	157.108	---	---	12
P	2	919.673	254.161	1292.672	---	11
S	3	102.125	341.193	1195.619	0.853	10
R	4	-316.835	497.294	1108.587	403.923	9
I	5	502.530	610.378	952.486	---	8
P	6	33.264	707.431	839.402	437.566	7
S#	7	---	874.429	742.349	---	6
T	8	---	975.477	575.351	-189.929	5
V	9	76.186	1074.546	474.303	-130.486	4
L	10	246.114	1187.630	375.235	309.854	3
S	11	---	1274.662	262.151	285.205	2
R	12	---	---	175.119	---	1

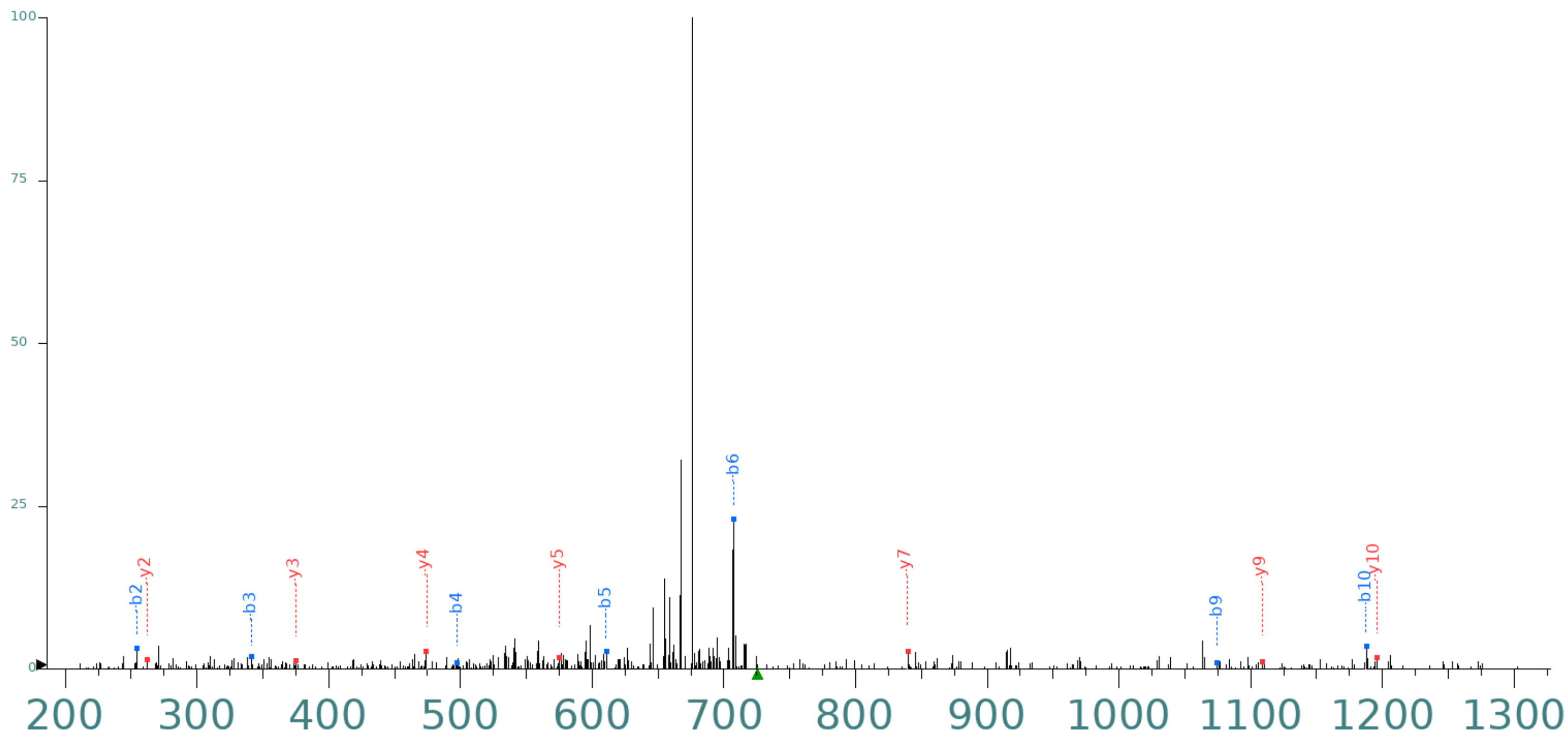

Predicted Fragmentation Pattern

Seq	#	b: Δ Error	b	y	y: Δ Error	+1
I	1	---	114.091	---	---	8
P	2	-136.603	211.144	839.402	36.382	7
S#	3	-72.898	378.142	742.349	80.145	6
T	4	---	479.190	575.351	377.828	5
V	5	107.421	578.259	474.303	---	4
L	6	634.237	691.343	375.235	275.715	3
S	7	483.404	778.375	262.151	-483.384	2
R	8	---	---	175.119	20.069	1

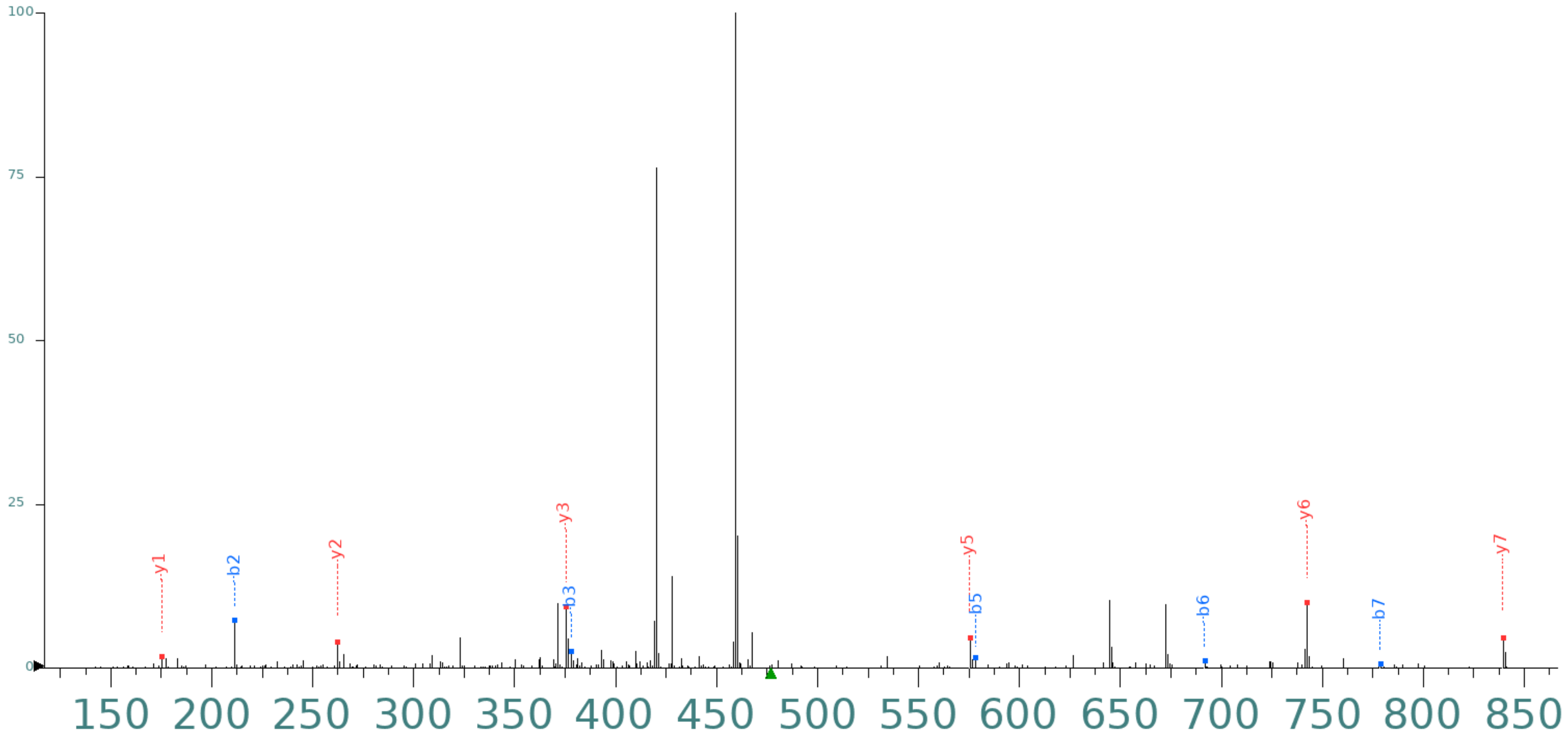

Predicted Fragmentation Pattern

Seq	#	b: Δ Error	b	y	y: Δ Error	+1
T	1	---	102.055	---	---	14
N	2	228.105	216.098	1434.674	---	13
V	3	-39.235	315.166	1320.631	---	12
L	4	---	428.250	1221.562	62.381	11
S	5	---	515.282	1108.478	129.483	10
P	6	-17.158	612.335	1021.446	188.406	9
H	7	-49.550	749.394	924.393	---	8
T#	8	-8.650	930.408	787.335	---	7
S	9	---	1017.440	606.321	-221.325	6
G	10	---	1074.462	519.289	-239.172	5
S	11	---	1161.494	462.267	-204.792	4
I	12	---	1274.578	375.235	-211.904	3
S	13	---	1361.610	262.151	-136.843	2
R	14	---	---	175.119	---	1

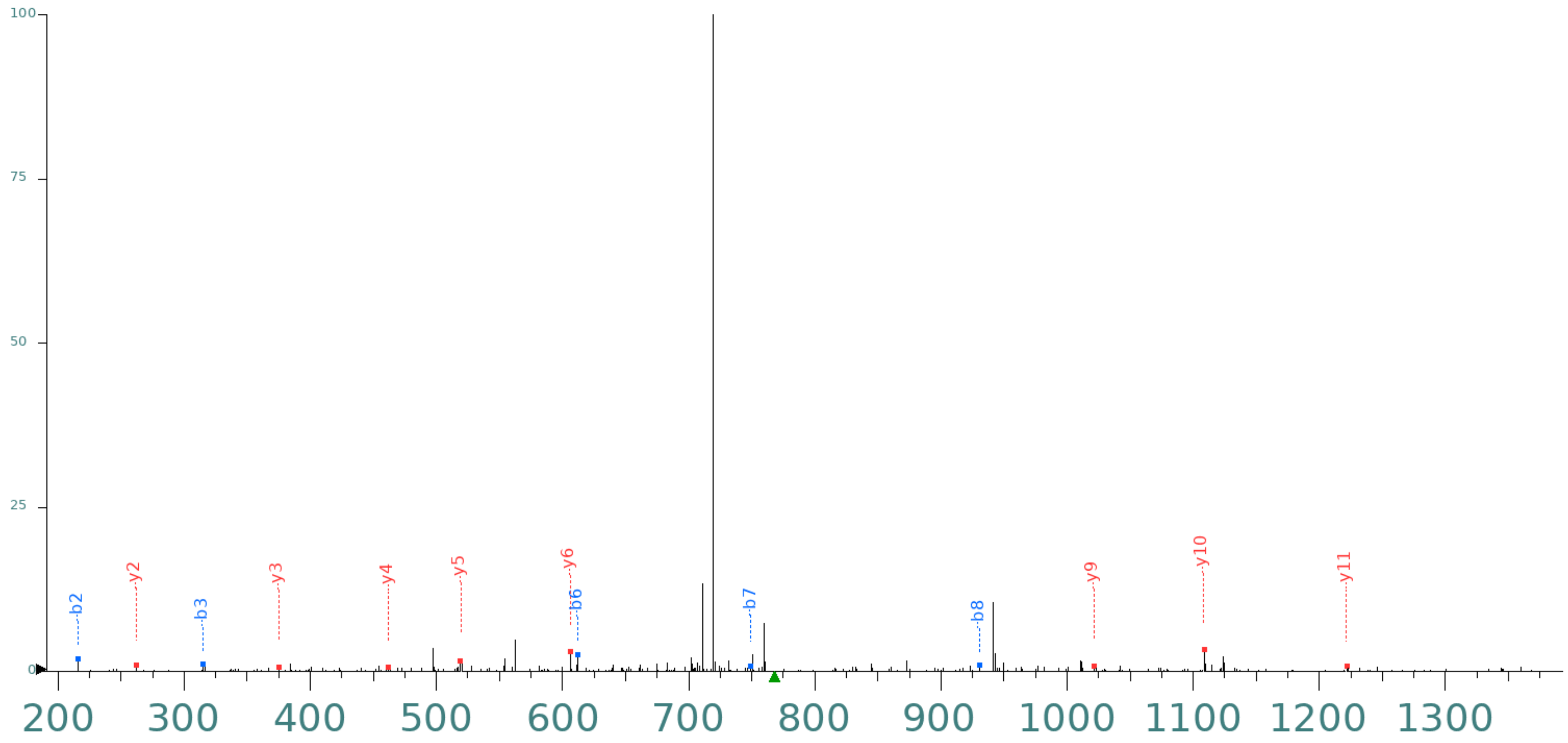

Predicted Fragmentation Pattern

Seq	#	b: Δ Error	b	y	y: Δ Error	+1
I	1	---	114.091	---	---	8
P	2	-440.589	211.144	839.402	63.428	7
S#	3	-93.723	378.142	742.349	62.635	6
T	4	---	479.190	575.351	37.230	5
V	5	81.460	578.259	474.303	304.390	4
L	6	301.625	691.343	375.235	-255.191	3
S	7	---	778.375	262.151	-161.297	2
R	8	---	---	175.119	567.209	1

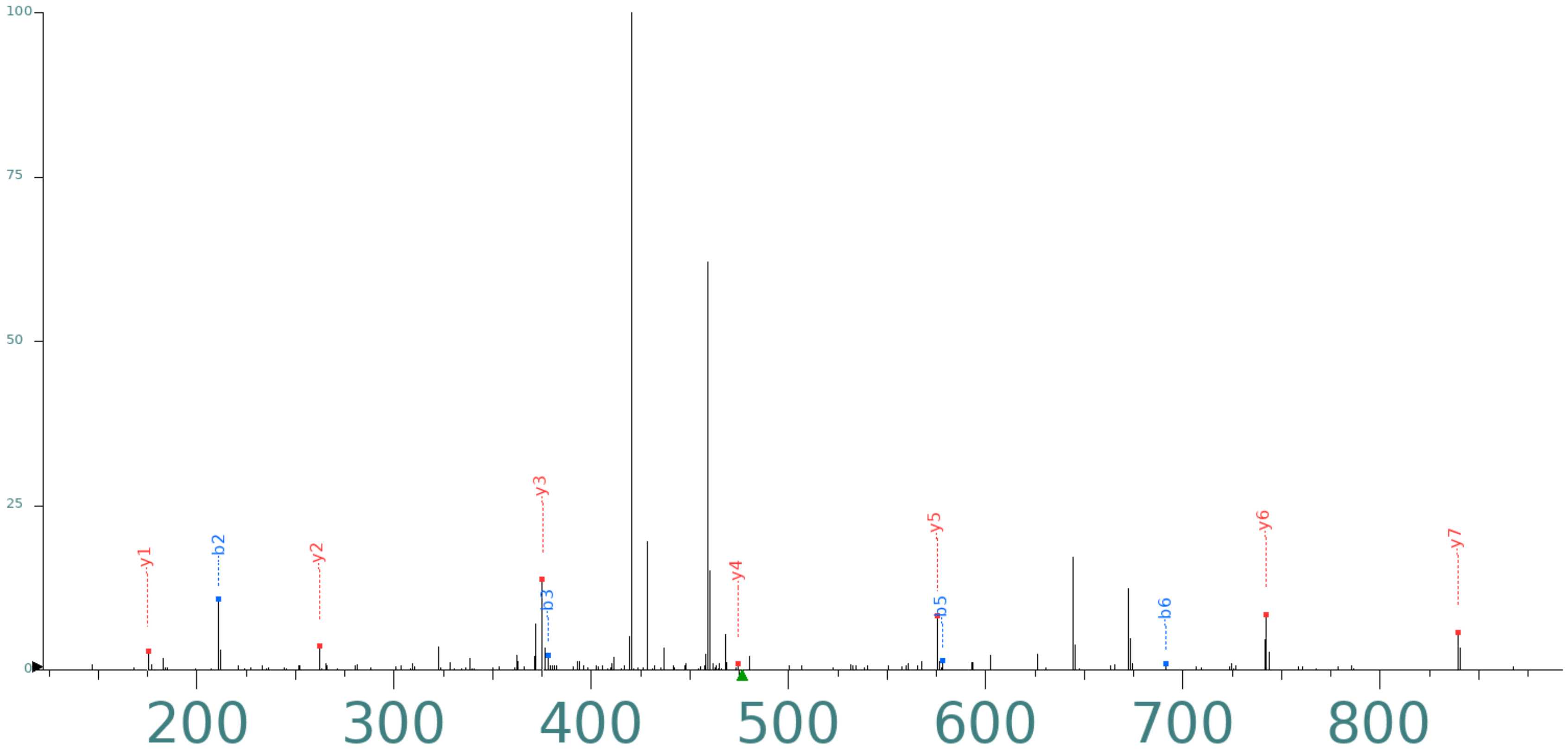

Predicted Fragmentation Pattern

Seq	#	b: Δ Error	b	y	y: Δ Error	+1
I	1	---	114.091	---	---	8
P	2	-273.244	211.144	839.402	254.747	7
S#	3	201.460	378.142	742.349	373.205	6
T	4	---	479.190	575.351	411.006	5
V	5	---	578.259	474.303	---	4
L	6	-585.537	691.343	375.235	-130.058	3
S	7	---	778.375	262.151	135.562	2
R	8	---	---	175.119	904.269	1

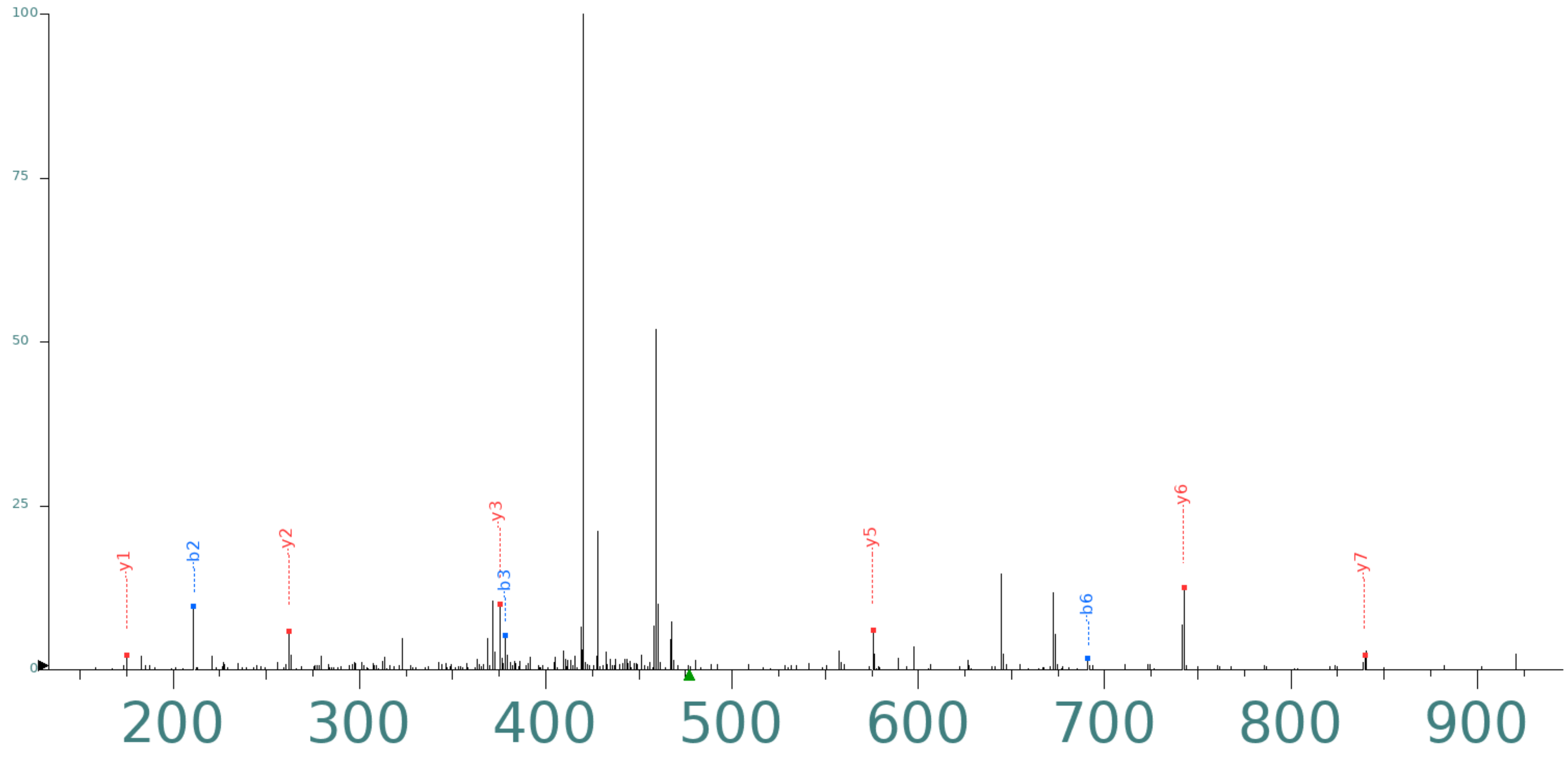

Predicted Fragmentation Pattern

Seq	#	b: Δ Error	b	y	y: Δ Error	+1
R	1	---	157.108	---	---	12
P	2	-413.048	254.161	1292.672	-84.230	11
S	3	---	341.193	1195.619	---	10
R	4	---	497.294	1108.587	---	9
I	5	648.954	610.378	952.486	---	8
P	6	47.240	707.431	839.402	547.762	7
S#	7	---	874.429	742.349	---	6
T	8	---	975.477	575.351	354.719	5
V	9	---	1074.546	474.303	804.158	4
L	10	---	1187.630	375.235	---	3
S	11	160.065	1274.662	262.151	---	2
R	12	---	---	175.119	---	1

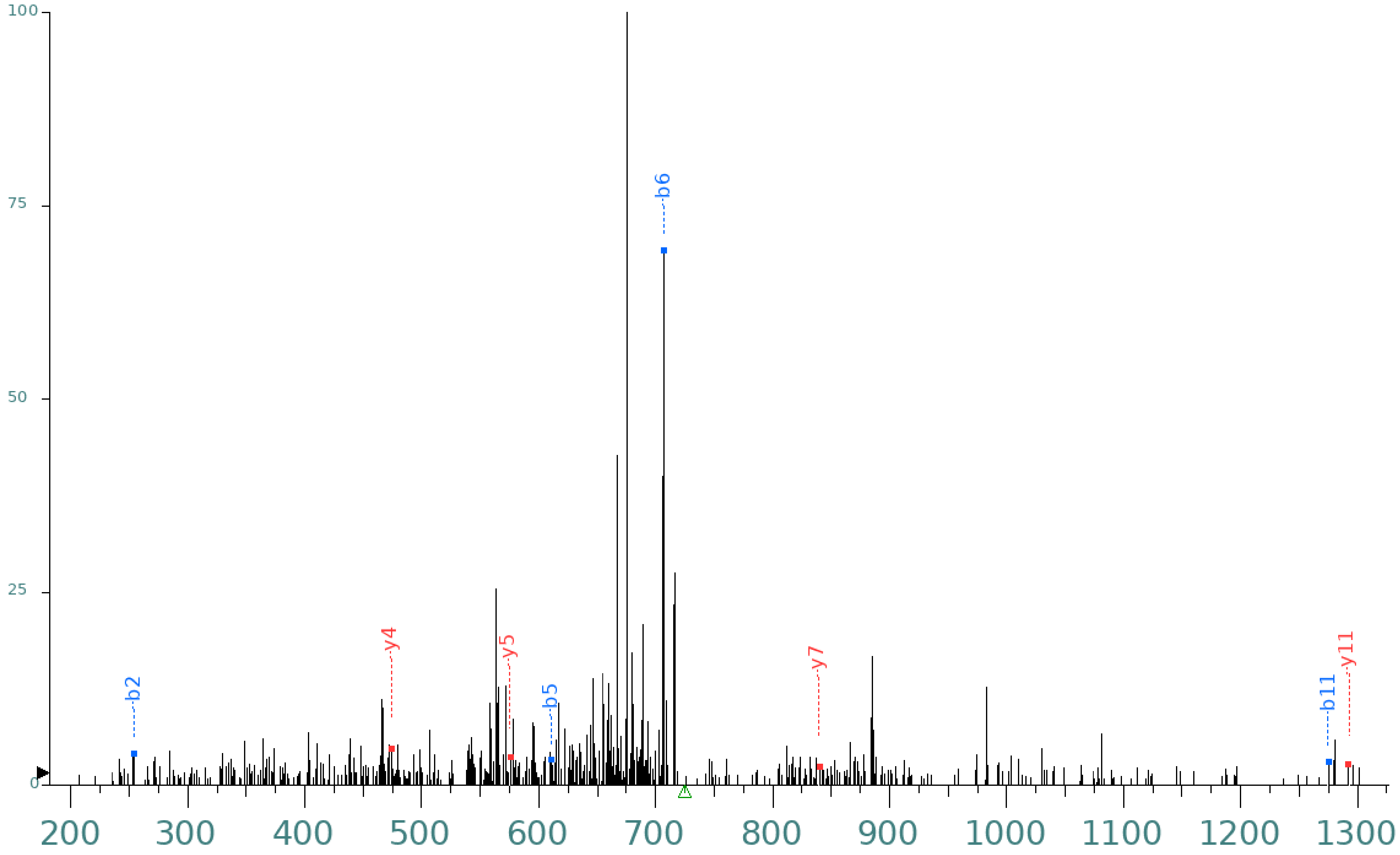

Predicted Fragmentation Pattern

Seq	#	b: Δ Error	b	y	y: Δ Error	+1
R	1	---	157.108	---	---	12
P	2	314.898	**254.161**	1292.672	---	11
S	3	---	341.193	1195.619	---	10
R	4	---	497.294	1108.587	---	9
I	5	115.986	**610.378**	952.486	---	8
P	6	-93.571	**707.431**	**839.402**	388.525	7
S	7	458.194	**794.463**	742.349	---	6
T#	8	-154.866	**975.477**	**655.317**	241.413	5
V	9	---	1074.546	474.303	---	4
L	10	---	1187.630	**375.235**	673.931	3
S	11	---	1274.662	262.151	---	2
R	12	---	---	175.119	---	1

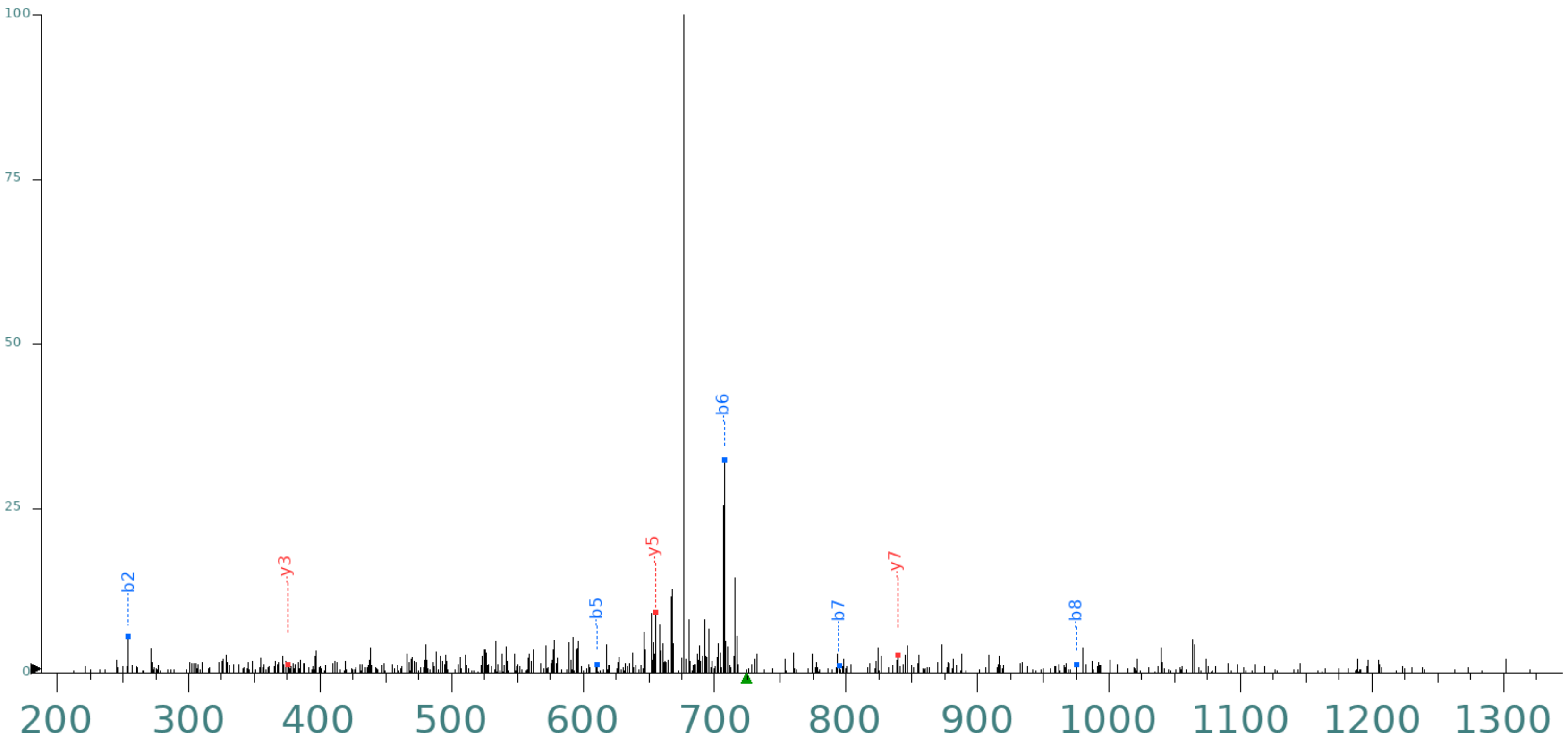

LPA3-LPA

Predicted Fragmentation Pattern

Seq	#	b: Δ Error	b	y	y: Δ Error	+1
S	1	---	88.039	---	---	20
D	2	---	203.066	2151.890	---	19
T#	3	---	384.080	2036.863	---	18
G	4	---	441.102	1855.849	---	17
S	5	---	528.134	1798.828	---	16
Q	6	---	656.192	1711.796	---	15
Y	7	---	819.256	1583.737	125.891	14
I	8	260.223	932.340	1420.674	145.818	13
E	9	-367.122	1061.382	1307.590	97.635	12
D	10	211.884	1176.409	1178.547	24.637	11
S	11	---	1263.441	1063.520	4.888	10
I	12	---	1376.525	976.488	64.121	9
S	13	---	1463.557	863.404	216.497	8
Q	14	---	1591.616	776.372	354.588	7
G	15	---	1648.637	648.313	47.232	6
A	16	264.223	1719.675	591.292	230.690	5
V	17	---	1818.743	520.255	265.238	4
C	18	97.937	1978.774	421.186	-146.043	3
N	19	---	2092.817	261.156	---	2
K	20	---	---	147.113	---	1

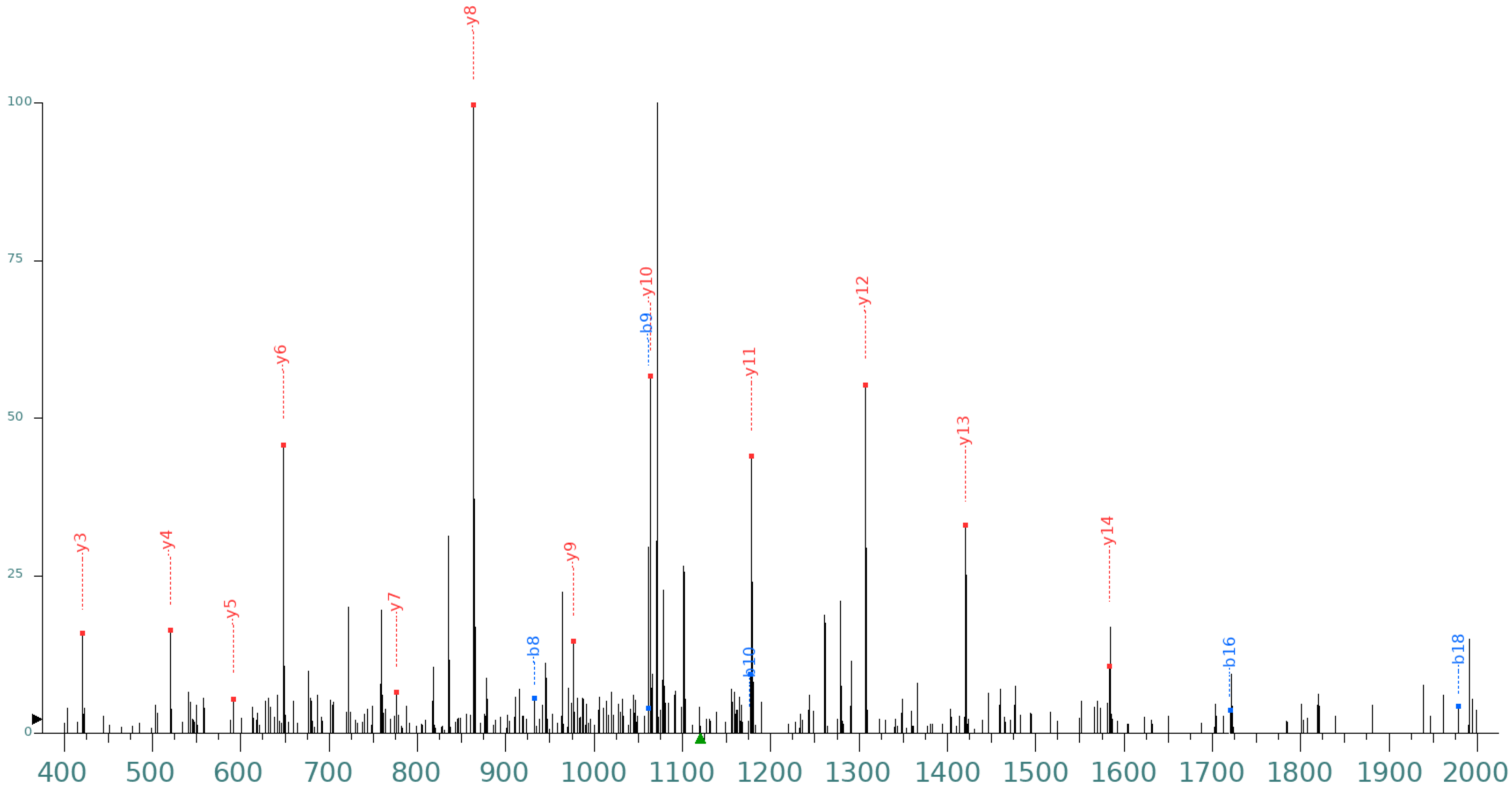

Predicted Fragmentation Pattern

Seq	#	b: Δ Error	b	y	y: Δ Error	+1
S	1	---	88.039	---	---	20
D	2	---	203.066	2151.890	---	19
T	3	---	304.114	2036.863	---	18
G	4	---	361.135	1935.815	---	17
S	5	---	448.167	1878.794	---	16
Q	6	---	576.226	1791.762	---	15
Y	7	177.777	739.289	1663.703	46.467	14
I	8	191.324	852.373	1500.640	50.441	13
E	9	232.202	981.416	1387.556	246.425	12
D	10	249.832	1096.443	1258.513	114.412	11
S	11	106.661	1183.475	1143.486	241.780	10
I	12	91.649	1296.559	1056.454	66.179	9
S#	13	---	1463.557	943.370	107.057	8
Q	14	---	1591.616	776.372	262.193	7
G	15	---	1648.637	648.313	57.022	6
A	16	---	1719.675	591.292	301.773	5
V	17	266.307	1818.743	520.255	137.765	4
C	18	106.202	1978.774	421.186	-127.779	3
N	19	---	2092.817	261.156	---	2
K	20	---	---	147.113	---	1

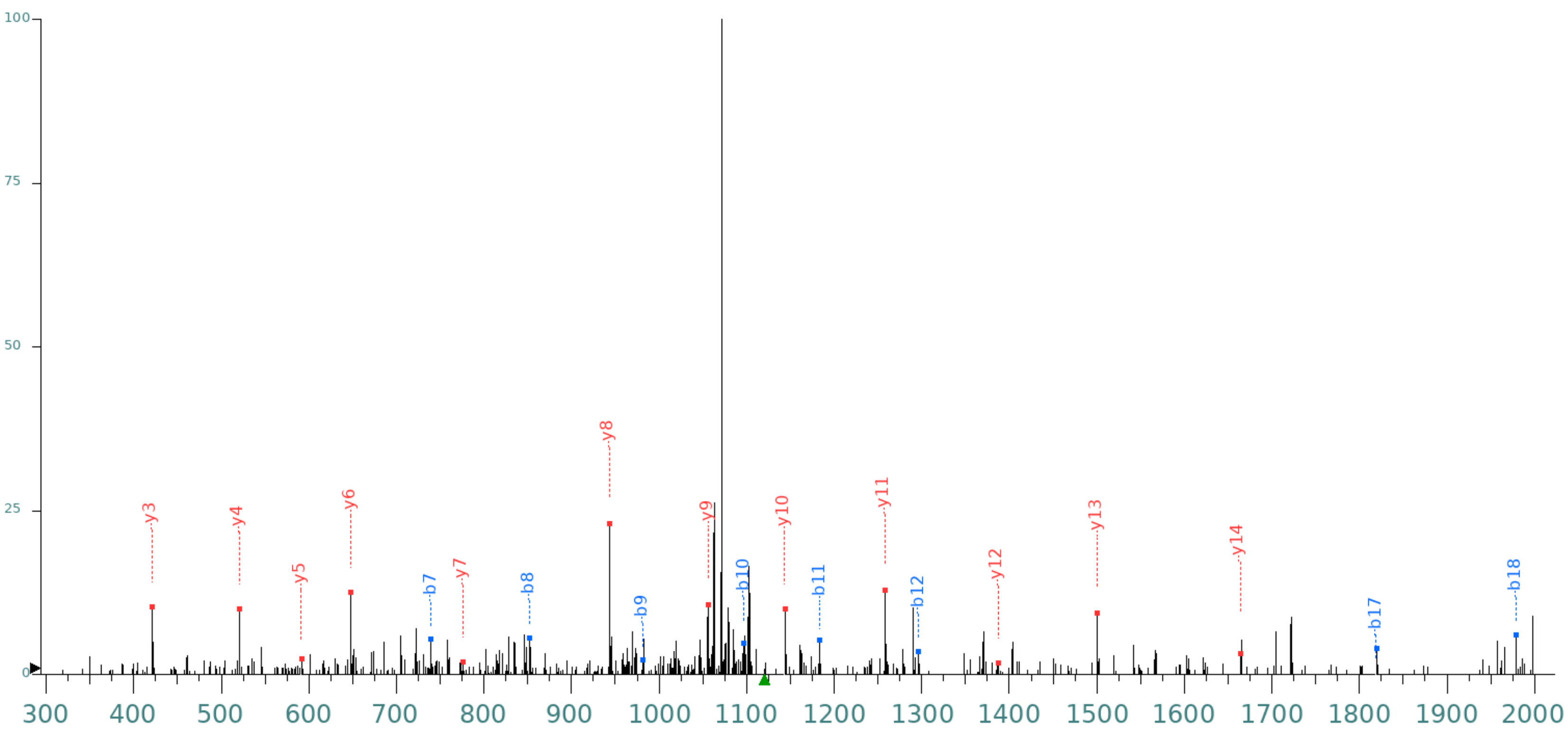

Predicted Fragmentation Pattern

Seq	#	b: Δ Error	b	y	y: Δ Error	+1
T	1	---	102.055	---	---	14
N	2	23.175	216.098	1434.674	---	13
V	3	374.284	315.166	1320.631	---	12
L	4	281.987	428.250	1221.562	322.698	11
S	5	221.139	515.282	1108.478	132.676	10
P	6	228.093	612.335	1021.446	49.213	9
H	7	51.525	749.394	924.393	170.263	8
T	8	71.871	850.442	787.335	173.566	7
S	9	298.275	937.474	686.287	389.963	6
G	10	---	994.495	599.255	297.446	5
S#	11	---	1161.494	542.233	-77.121	4
I	12	---	1274.578	375.235	8.218	3
S	13	45.582	1361.610	262.151	531.332	2
R	14	---	---	175.119	---	1

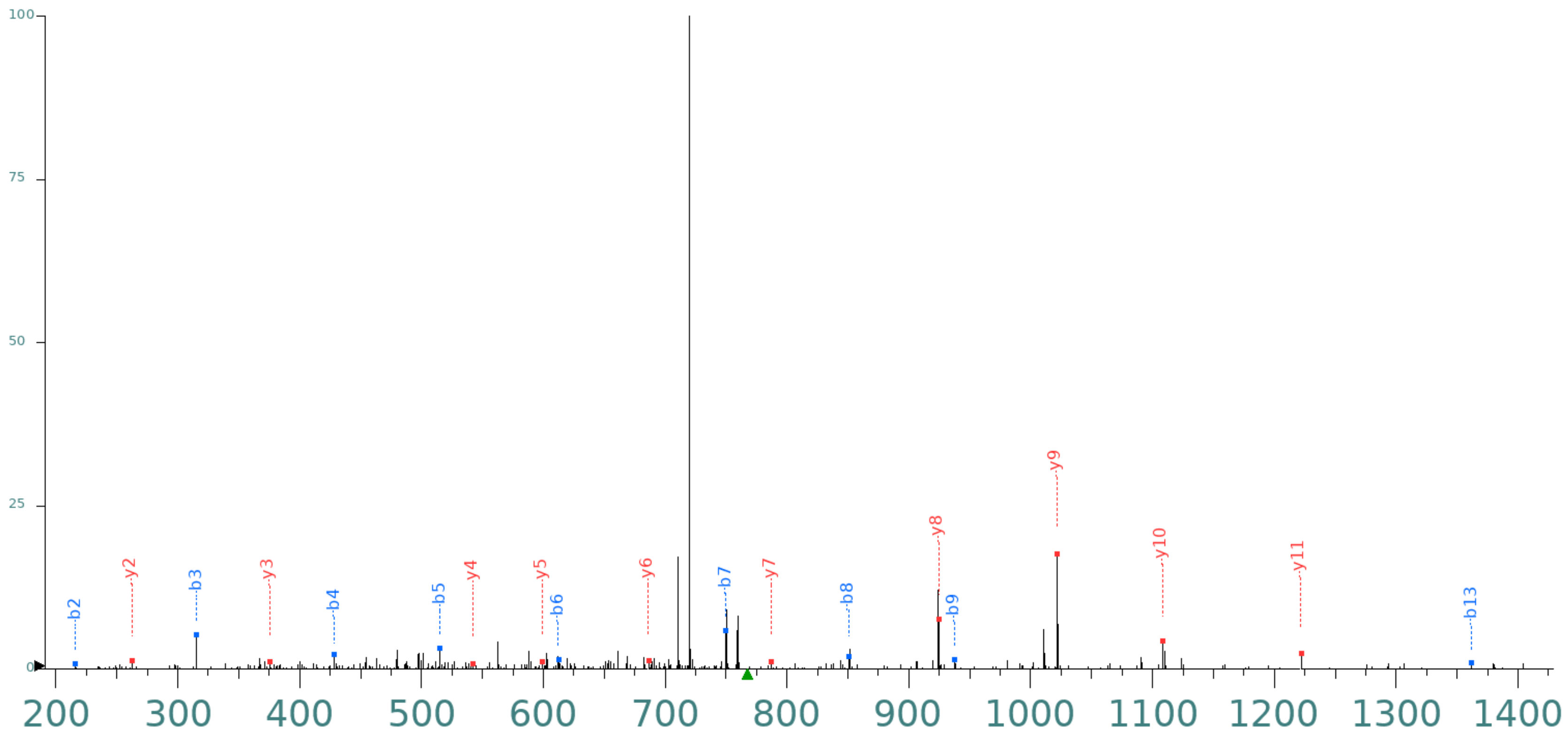

Predicted Fragmentation Pattern

Seq	#	b: Δ Error	b	y	y: Δ Error	+1
T	1	---	102.055	---	---	14
N	2	-346.097	**216.098**	1434.674	---	13
V	3	-220.742	**315.166**	1320.631	---	12
L	4	753.531	**428.250**	**1221.562**	54.388	11
S	5	313.717	**515.282**	**1108.478**	103.169	10
P	6	-10.081	**612.335**	**1021.446**	90.915	9
H	7	184.088	**749.394**	**924.393**	288.068	8
T	8	348.351	**850.442**	787.335	---	7
S	9	---	937.474	**686.287**	175.661	6
G	10	307.328	**994.495**	**599.255**	150.743	5
S#	11	---	1161.494	**542.233**	378.057	4
I	12	---	1274.578	**375.235**	338.057	3
S	13	---	1361.610	**262.151**	778.733	2
R	14	---	---	175.119	---	1

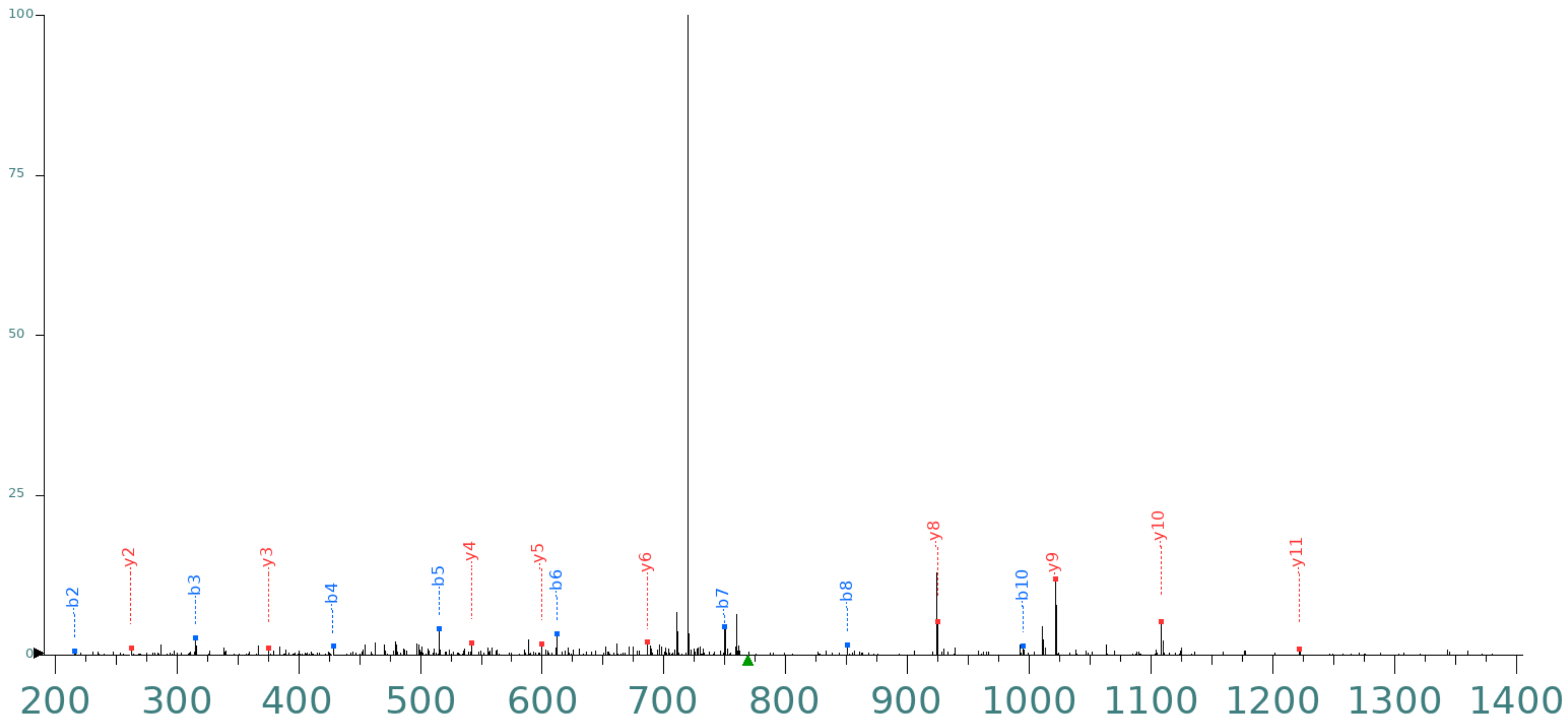

Predicted Fragmentation Pattern

Seq	#	b: Δ Error	b	y	y: Δ Error	+1
T	1	---	102.055	---	---	14
N	2	---	216.098	1434.674	---	13
V	3	34.162	**315.166**	1320.631	---	12
L	4	953.291	**428.250**	**1221.562**	282.950	11
S	5	---	515.282	**1108.478**	199.059	10
P	6	87.993	**612.335**	**1021.446**	286.654	9
H	7	367.728	**749.394**	**924.393**	98.775	8
T#	8	319.184	**930.408**	787.335	---	7
S	9	-1.237	**1017.440**	**606.321**	241.321	6
G	10	---	1074.462	**519.289**	397.186	5
S	11	---	1161.494	**462.267**	247.011	4
I	12	---	1274.578	**375.235**	218.732	3
S	13	---	1361.610	262.151	---	2
R	14	---	---	175.119	---	1

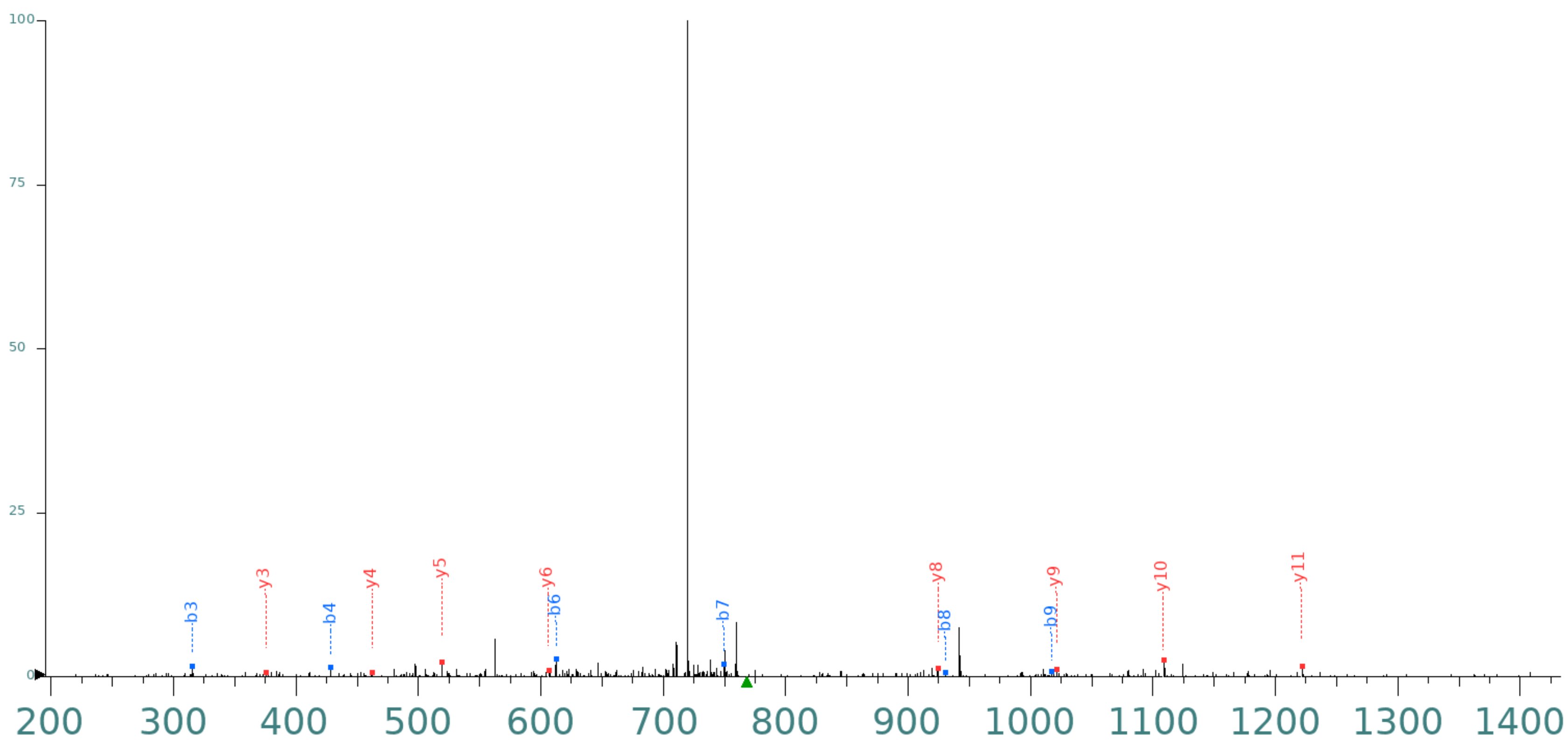

Predicted Fragmentation Pattern

Seq	#	b: Δ Error	b	y	y: Δ Error	+1
I	1	---	114.091	---	---	8
P	2	-888.518	211.144	839.402	-82.072	7
S#	3	-164.196	378.142	742.349	187.741	6
T	4	---	479.190	575.351	42.322	5
V	5	122.828	578.259	474.303	-149.086	4
L	6	44.629	691.343	375.235	-405.261	3
S	7	---	778.375	262.151	268.684	2
R	8	---	---	175.119	-318.198	1

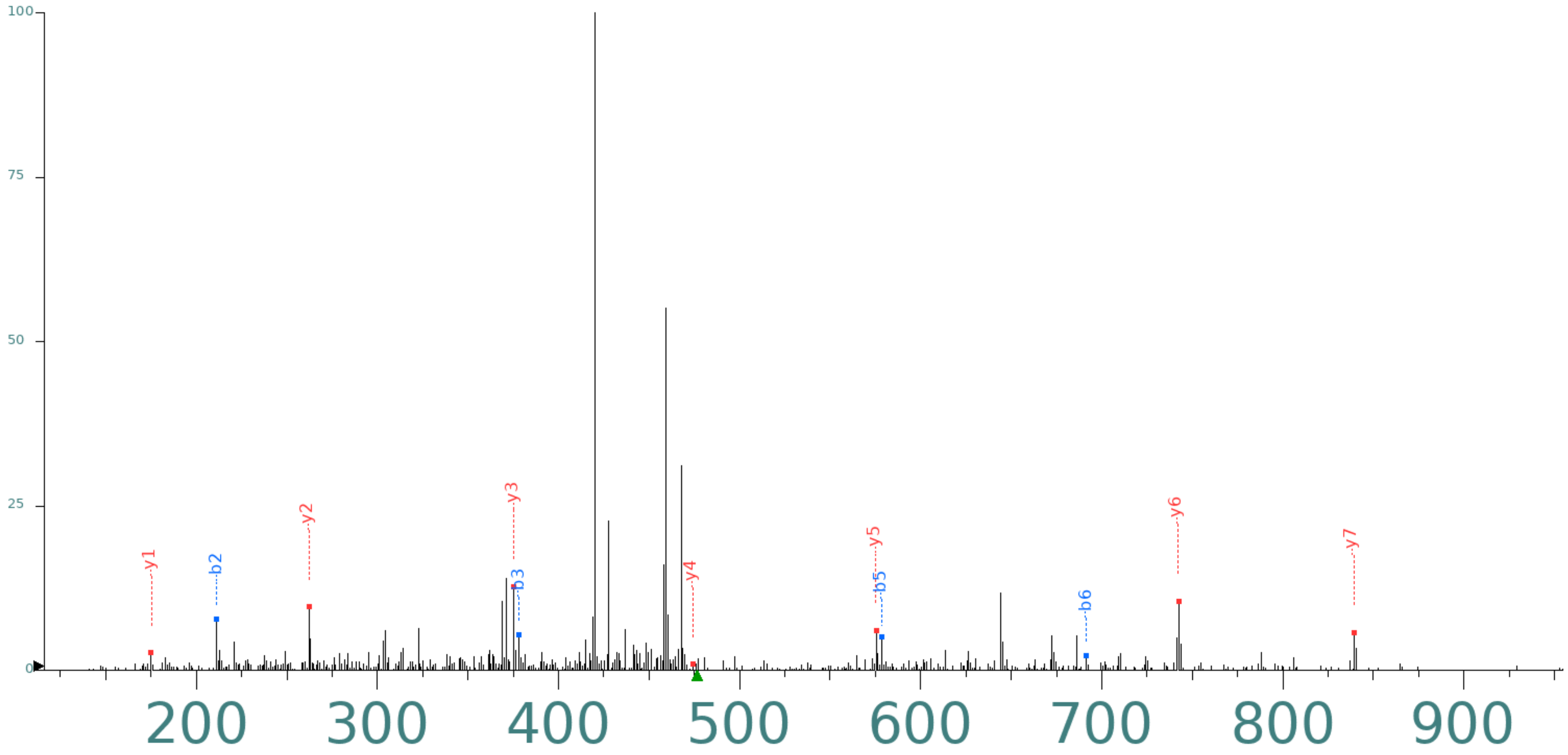

Predicted Fragmentation Pattern

Seq	#	b: Δ Error	b	y	y: Δ Error	+1
I	1	---	114.091	---	---	8
P	2	-663.418	211.144	839.402	291.011	7
S#	3	-617.057	378.142	742.349	125.767	6
T	4	---	479.190	575.351	252.411	5
V	5	272.016	578.259	474.303	---	4
L	6	84.881	691.343	375.235	-25.778	3
S	7	---	778.375	262.151	-192.856	2
R	8	---	---	175.119	601.152	1

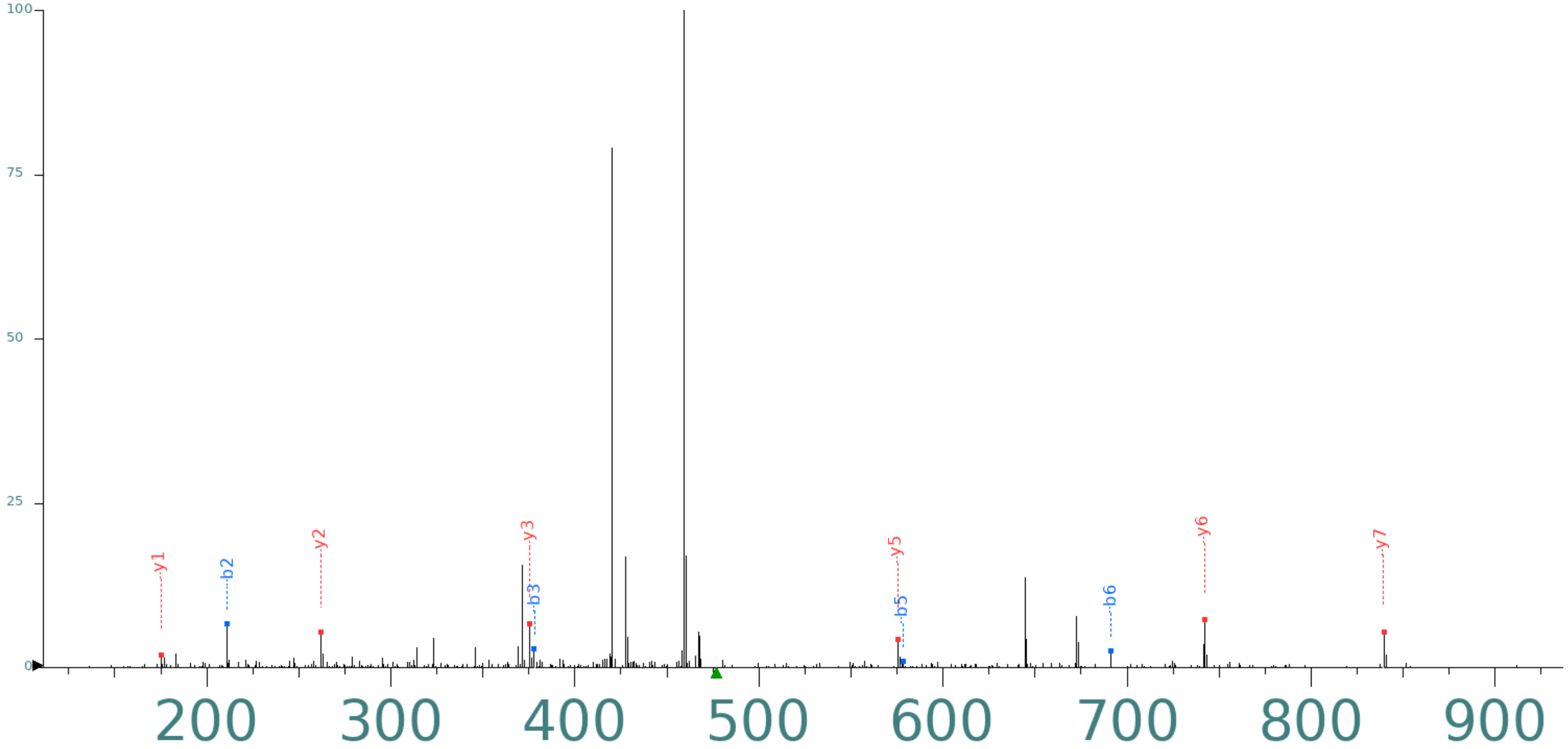

Predicted Fragmentation Pattern

Seq	#	b: Δ Error	b	y	y: Δ Error	+1
I	1	---	114.091	---	---	8
P	2	-56.226	211.144	839.402	229.964	7
S#	3	243.649	378.142	742.349	125.521	6
T	4	---	479.190	575.351	130.250	5
V	5	515.645	578.259	474.303	---	4
L	6	225.034	691.343	375.235	-142.993	3
S	7	---	778.375	262.151	-205.200	2
R	8	---	---	175.119	-214.890	1

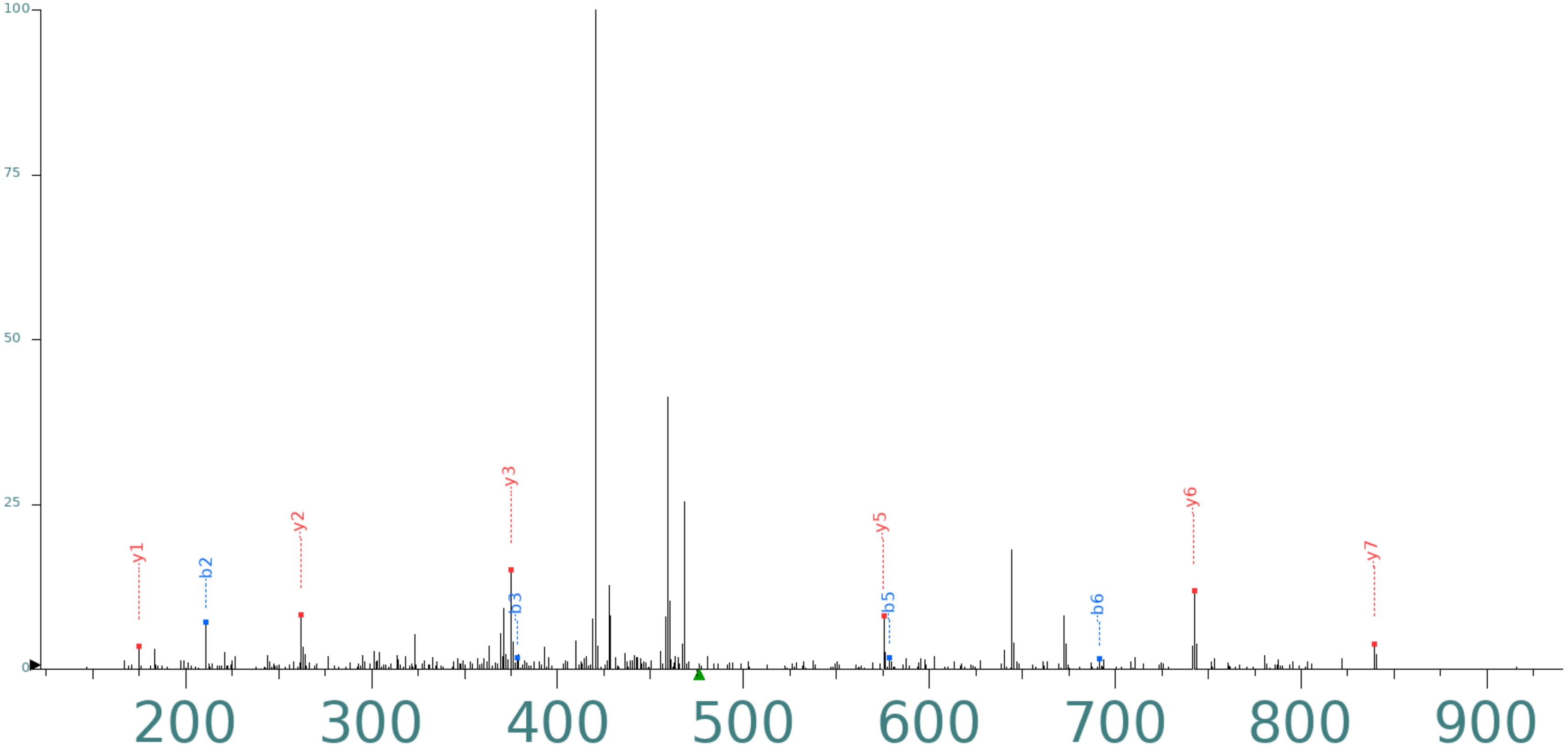

Predicted Fragmentation Pattern

Seq	#	b: Δ Error	b	y	y: Δ Error	+1
I	1	---	114.091	---	---	8
P	2	-502.219	211.144	839.402	96.289	7
S#	3	544.437	378.142	742.349	83.433	6
T	4	---	479.190	575.351	400.089	5
V	5	382.138	578.259	474.303	---	4
L	6	-196.956	691.343	375.235	93.930	3
S	7	---	778.375	262.151	-304.784	2
R	8	---	---	175.119	489.220	1

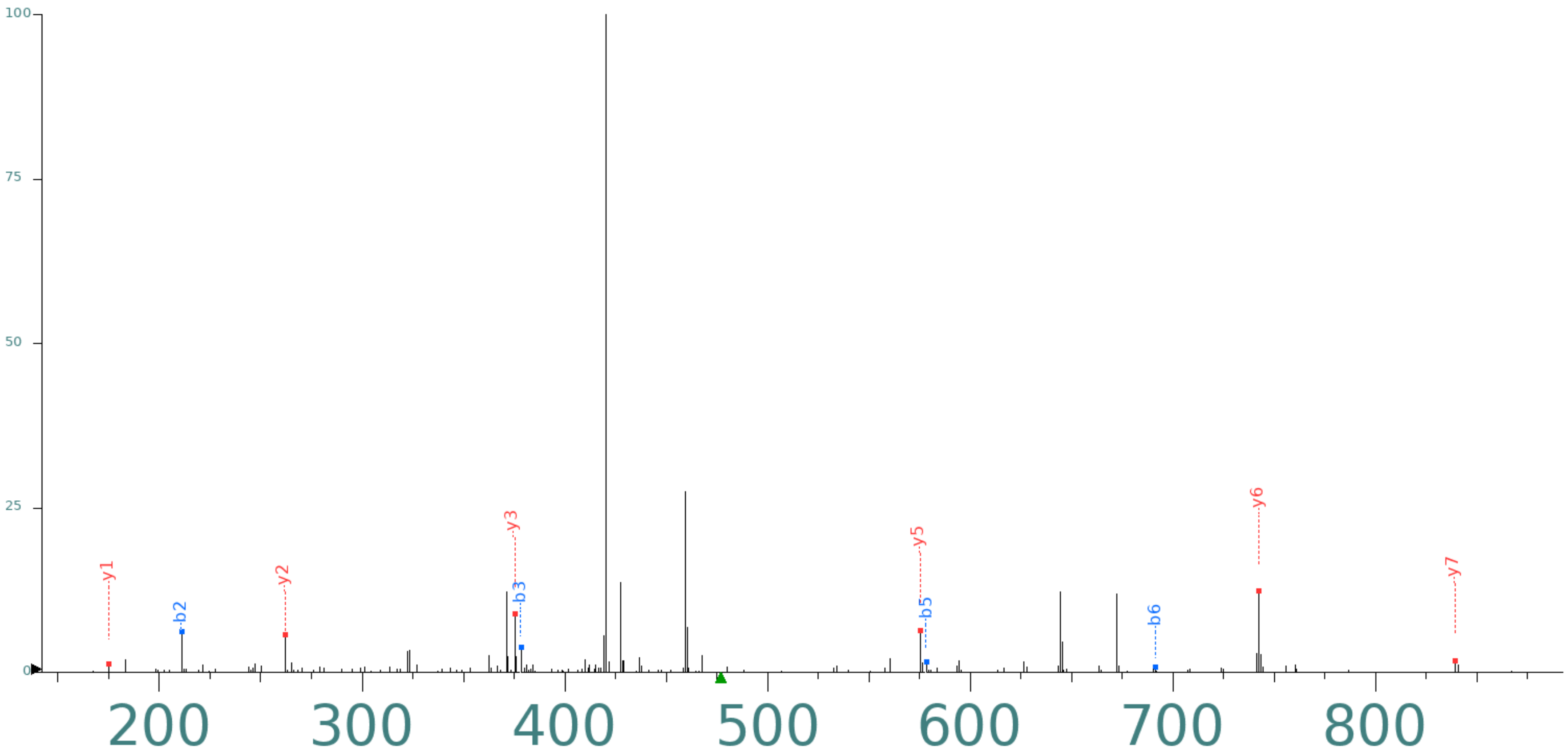

LPA3- Basal

Predicted Fragmentation Pattern

Seq	#	b: Δ Error	b	y	y: Δ Error	+1
I	1	---	114.091	---	---	8
P	2	-218.728	211.144	839.402	71.862	7
S#	3	208.397	378.142	742.349	277.400	6
T	4	---	479.190	575.351	134.704	5
V	5	211.143	578.259	474.303	---	4
L	6	-212.589	691.343	375.235	84.579	3
S	7	---	778.375	262.151	300.679	2
R	8	---	---	175.119	137.158	1

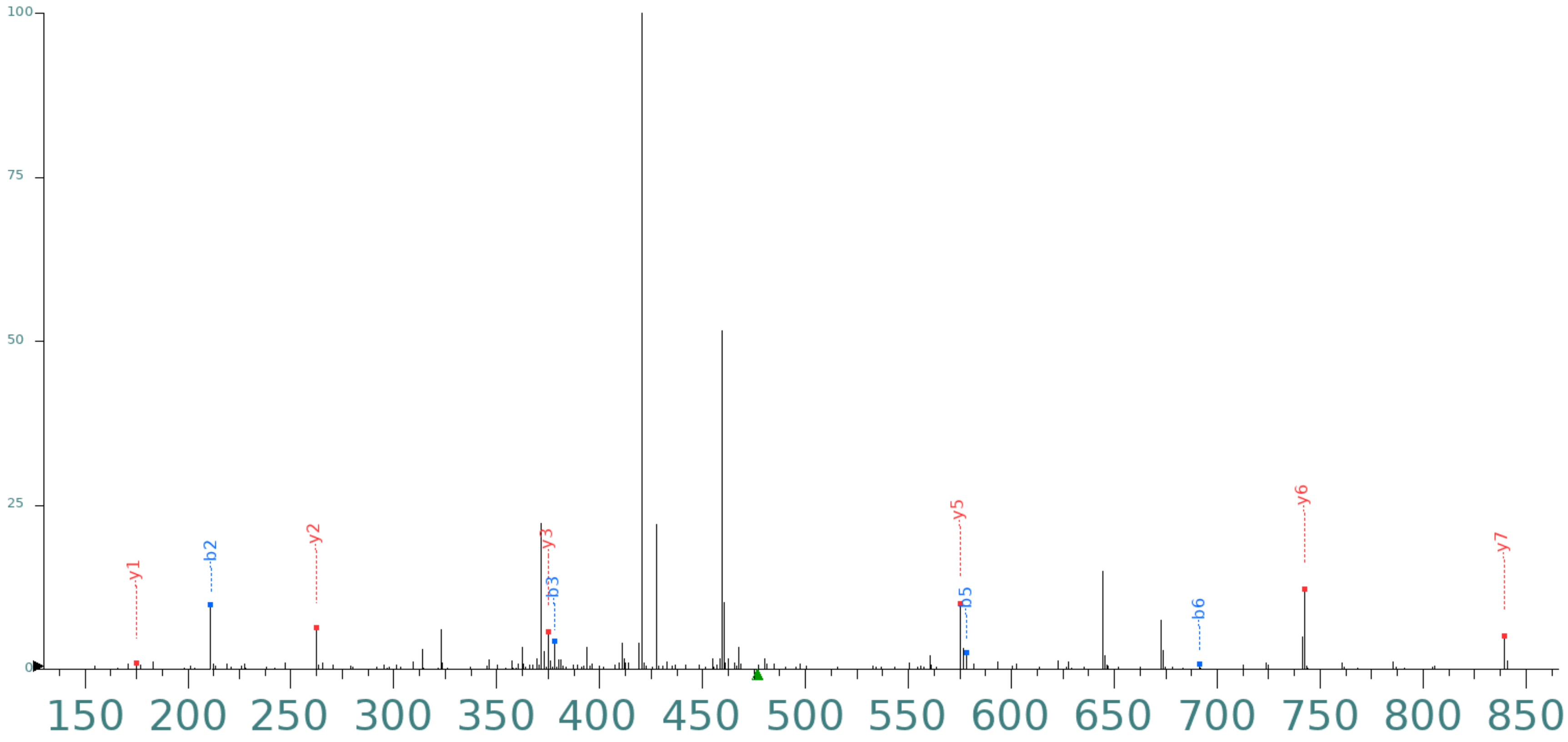

Predicted Fragmentation Pattern

Seq	#	b: Δ Error	b	y	y: Δ Error	+1
I	1	---	114.091	---	---	8
P	2	194.073	211.144	839.402	175.088	7
S#	3	32.344	378.142	742.349	-4.041	6
T	4	---	479.190	575.351	77.643	5
V	5	49.800	578.259	474.303	978.663	4
L	6	---	691.343	375.235	-182.939	3
S	7	334.775	778.375	262.151	-241.421	2
R	8	---	---	175.119	-97.658	1

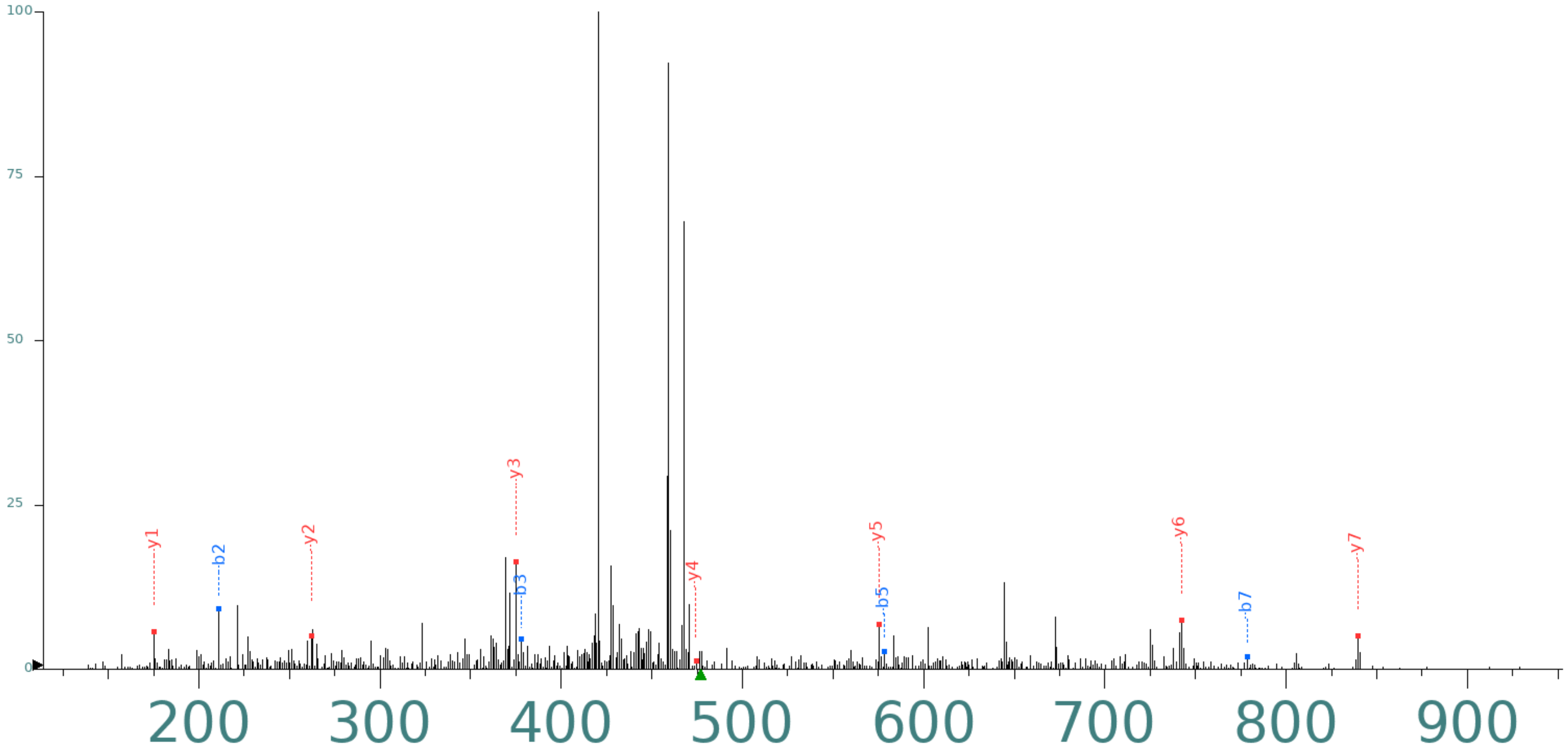

Predicted Fragmentation Pattern

Seq	#	b: Δ Error	b	y	y: Δ Error	+1
S	1	---	88.039	---	---	20
D	2	---	203.066	2151.890	---	19
T#	3	---	384.080	2036.863	---	18
G	4	35.618	**441.102**	**1855.849**	-18.850	17
S	5	---	528.134	**1798.828**	43.244	16
Q	6	280.910	**656.192**	1711.796	---	15
Y	7	263.615	**819.256**	**1583.737**	107.396	14
I	8	115.469	**932.340**	**1420.674**	3.290	13
E	9	9.328	**1061.382**	**1307.590**	41.723	12
D	10	-19.157	**1176.409**	**1178.547**	7.030	11
S	11	44.540	**1263.441**	**1063.520**	75.587	10
I	12	76.734	**1376.525**	**976.488**	24.622	9
S	13	137.517	**1463.557**	**863.404**	-28.897	8
Q	14	27.082	**1591.616**	**776.372**	109.735	7
G	15	---	1648.637	**648.313**	-2.851	6
A	16	-6.371	**1719.675**	**591.292**	142.158	5
V	17	91.393	**1818.743**	**520.255**	-36.199	4
C	18	---	1978.774	**421.186**	-355.836	3
N	19	---	2092.817	261.156	---	2
K	20	---	---	147.113	---	1

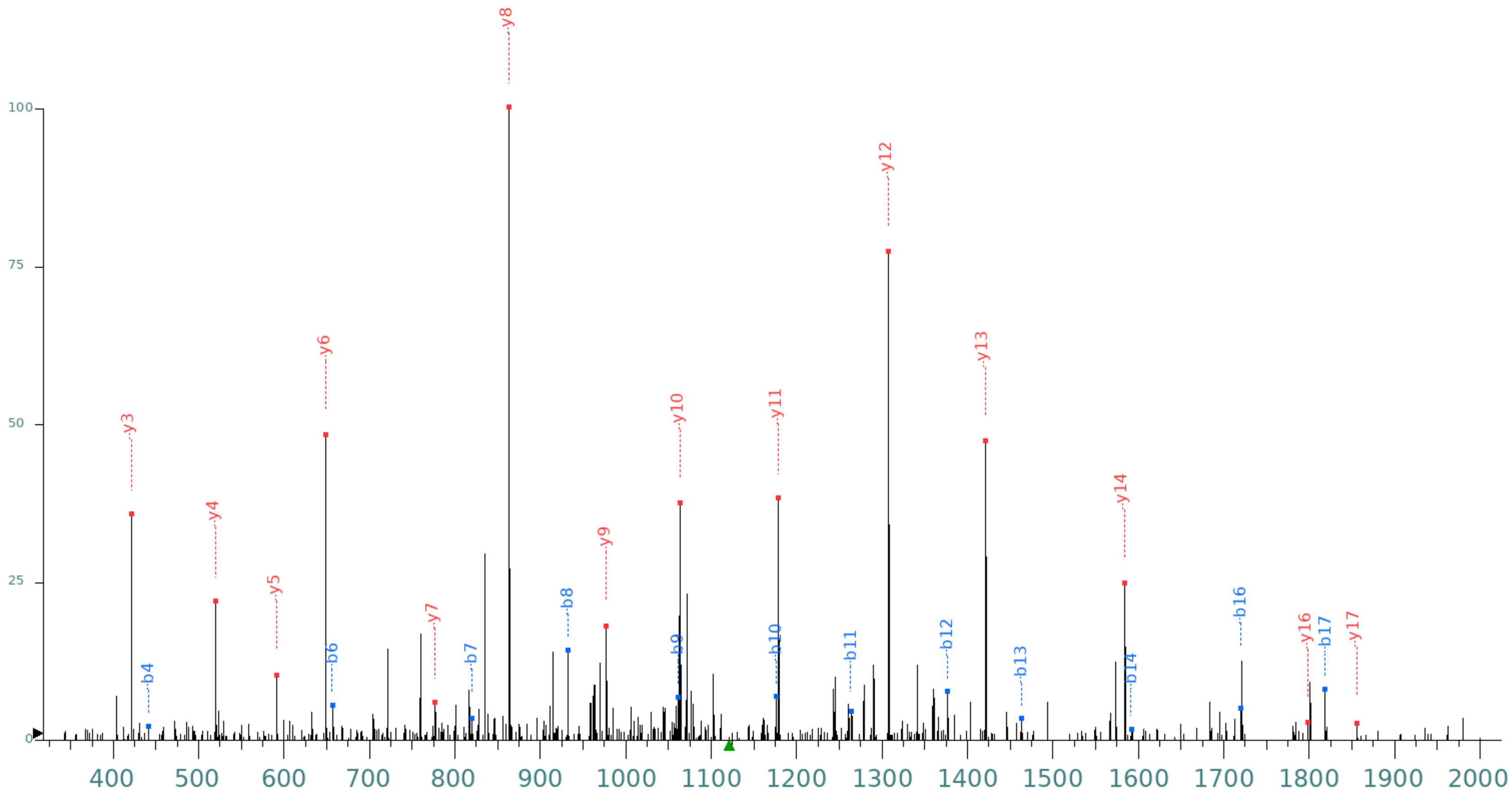

Predicted Fragmentation Pattern

Seq	#	b: Δ Error	b	y	y: Δ Error	+1
S	1	---	88.039	---	---	20
D	2	---	203.066	2151.890	---	19
T	3	---	304.114	2036.863	---	18
G	4	---	361.135	1935.815	---	17
S	5	41.198	**448.167**	1878.794	---	16
Q	6	238.616	**576.226**	1791.762	---	15
Y	7	-40.810	**739.289**	**1663.703**	149.902	14
I	8	-54.681	**852.373**	**1500.640**	157.714	13
E	9	124.028	**981.416**	**1387.556**	175.987	12
D	10	125.074	**1096.443**	**1258.513**	84.155	11
S	11	140.793	**1183.475**	**1143.486**	280.084	10
I	12	-8.988	**1296.559**	1056.454	---	9
S#	13	---	1463.557	**943.370**	126.074	8
Q	14	---	1591.616	**776.372**	132.213	7
G	15	---	1648.637	**648.313**	-85.140	6
A	16	97.896	**1719.675**	**591.292**	157.843	5
V	17	147.490	**1818.743**	**520.255**	9.321	4
C	18	145.057	**1978.774**	**421.186**	228.670	3
N	19	---	2092.817	261.156	---	2
K	20	---	---	147.113	---	1

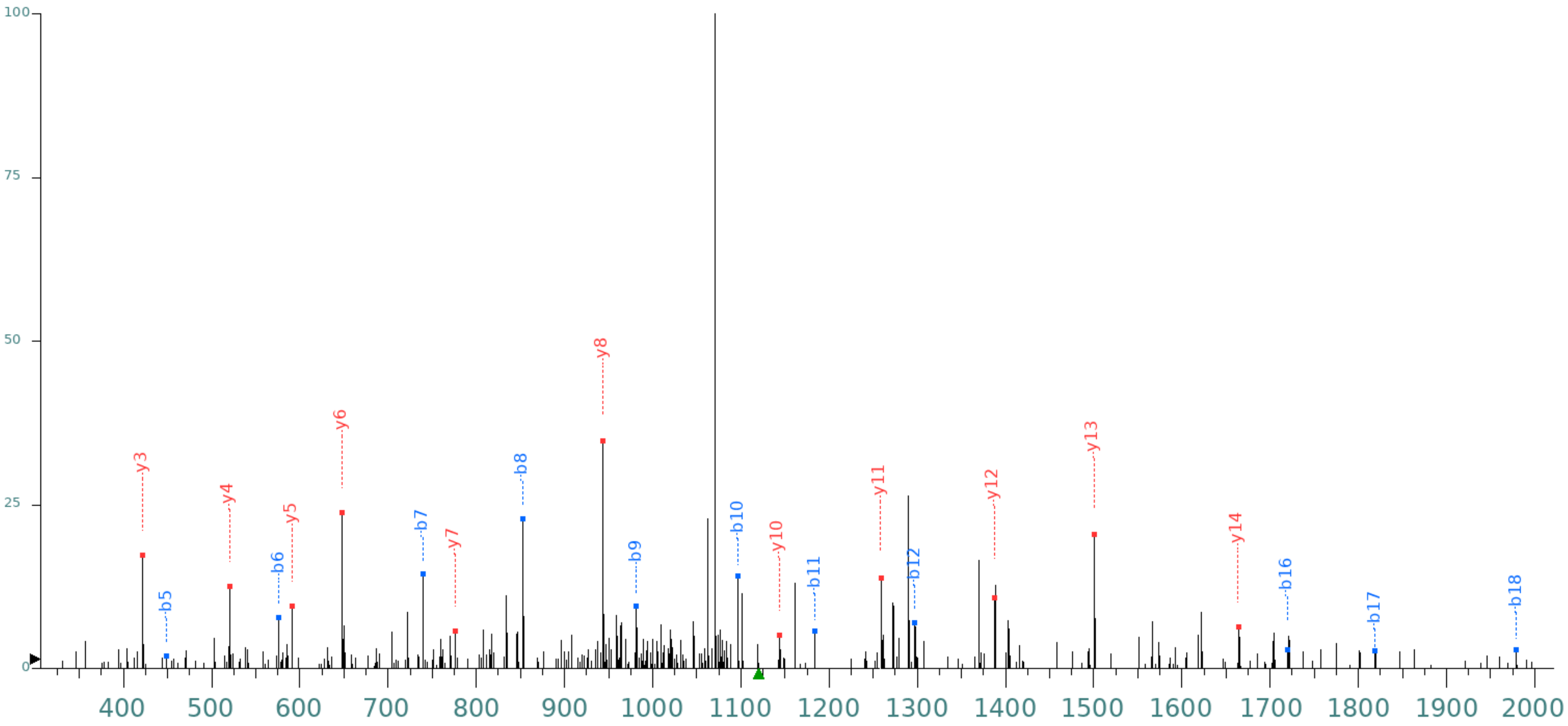

Predicted Fragmentation Pattern

+1

Seq	#	b: Δ Error	b	y	y: Δ Error	+1
S#	1	---	168.006	---	---	20
D	2	216.192	**283.033**	2071.924	---	19
T	3	308.526	**384.080**	1956.897	---	18
G	4	---	441.102	1855.849	---	17
S	5	623.984	**528.134**	1798.828	---	16
Q	6	-403.851	**656.192**	1711.796	---	15
Y	7	233.904	**819.256**	1583.737	---	14
I	8	316.228	**932.340**	1420.674	---	13
E	9	247.913	**1061.382**	1307.590	---	12
D	10	220.596	**1176.409**	**1178.547**	137.518	11
S	11	242.645	**1263.441**	**1063.520**	-30.235	10
I	12	229.837	**1376.525**	**976.488**	323.228	9
S	13	---	1463.557	**863.404**	72.116	8
Q	14	---	1591.616	**776.372**	112.564	7
G	15	---	1648.637	**648.313**	-8.217	6
A	16	---	1719.675	**591.292**	298.575	5
V	17	---	1818.743	**520.255**	194.880	4
C	18	---	1978.774	**421.186**	302.464	3
N	19	---	2092.817	**261.156**	326.386	2
K	20	---	---	147.113	---	1

+2

Seq	#	b: Δ Error	b	y	y: Δ Error	+1
S#	1	---	84.506	---	---	20
D	2	---	142.020	1036.465	---	19
T	3	---	192.544	**978.952**	-422.879	18
G	4	---	221.054	928.428	---	17
S	5	---	264.571	899.917	---	16
Q	6	---	328.600	**856.401**	259.997	15
Y	7	---	410.131	792.372	---	14
I	8	---	466.673	710.840	---	13
E	9	---	531.195	**654.298**	-33.365	12
D	10	-555.726	**588.708**	589.777	---	11
S	11	364.250	**632.224**	**532.264**	707.246	10
I	12	453.394	**688.766**	**488.748**	-847.364	9
S	13	-280.017	**732.282**	432.206	---	8
Q	14	---	796.312	**388.690**	620.564	7
G	15	-159.293	**824.822**	324.660	---	6
A	16	177.017	**860.341**	**296.150**	754.788	5
V	17	192.029	**909.875**	260.631	---	4
C	18	---	989.890	211.097	---	3
N	19	269.709	**1046.912**	131.082	---	2
K	20	---	---	74.060	---	1

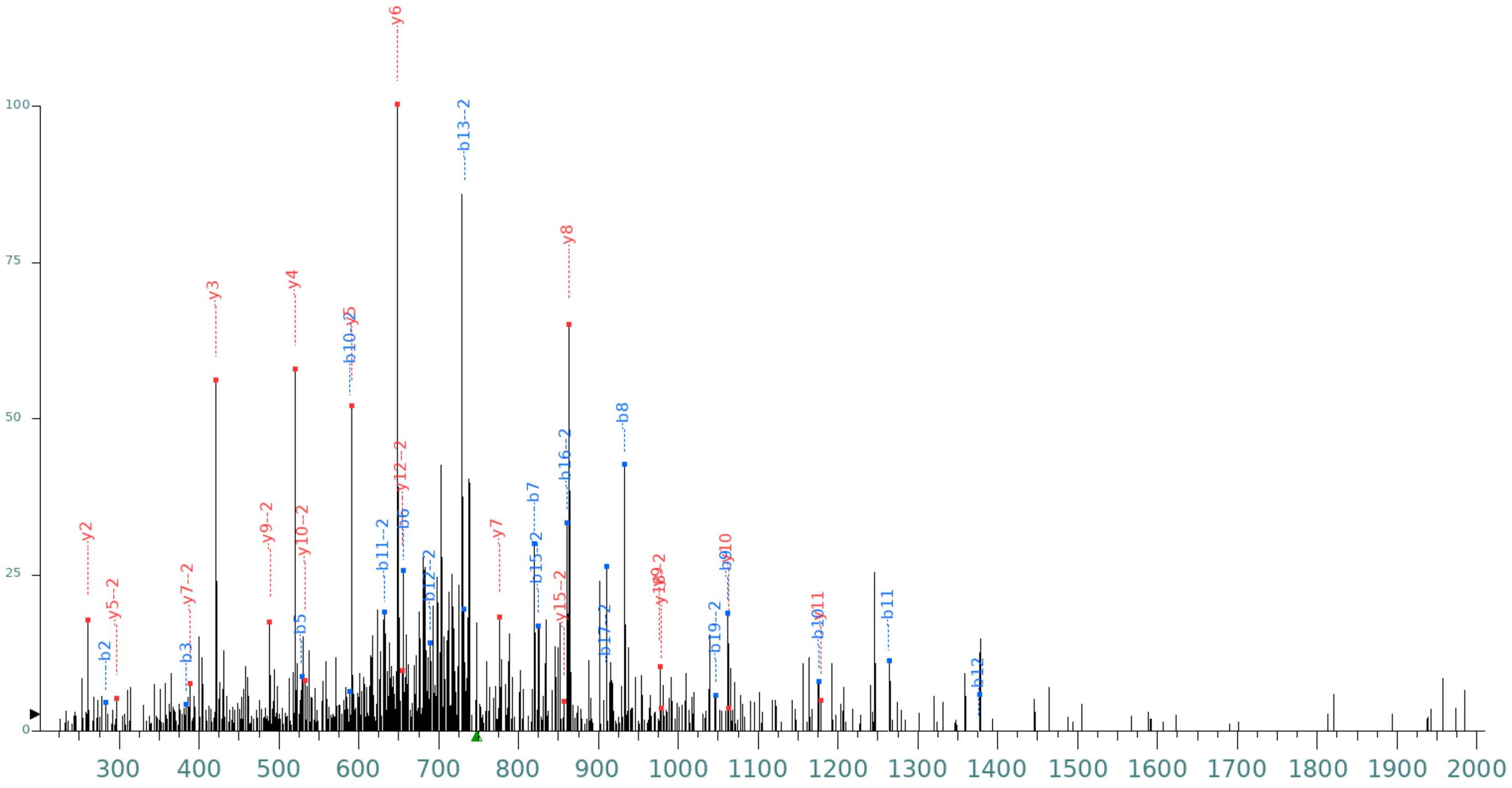

Predicted Fragmentation Pattern

Seq	#	b: Δ Error	b	y	y: Δ Error	+1
S	1	---	88.039	---	---	20
D	2	---	203.066	2151.890	---	19
T	3	---	304.114	2036.863	---	18
G	4	473.977	**361.135**	1935.815	---	17
S#	5	---	528.134	1878.794	---	16
Q	6	---	656.192	**1711.796**	49.248	15
Y	7	---	819.256	**1583.737**	184.837	14
I	8	234.442	**932.340**	**1420.674**	170.126	13
E	9	---	1061.382	**1307.590**	132.915	12
D	10	-44.373	**1176.409**	**1178.547**	69.689	11
S	11	---	1263.441	**1063.520**	205.939	10
I	12	194.646	**1376.525**	**976.488**	95.244	9
S	13	---	1463.557	**863.404**	3.339	8
Q	14	-36.500	**1591.616**	**776.372**	452.464	7
G	15	---	1648.637	**648.313**	230.763	6
A	16	95.554	**1719.675**	**591.292**	310.955	5
V	17	109.914	**1818.743**	**520.255**	211.415	4
C	18	230.157	**1978.774**	**421.186**	372.626	3
N	19	---	2092.817	261.156	---	2
K	20	---	---	147.113	---	1

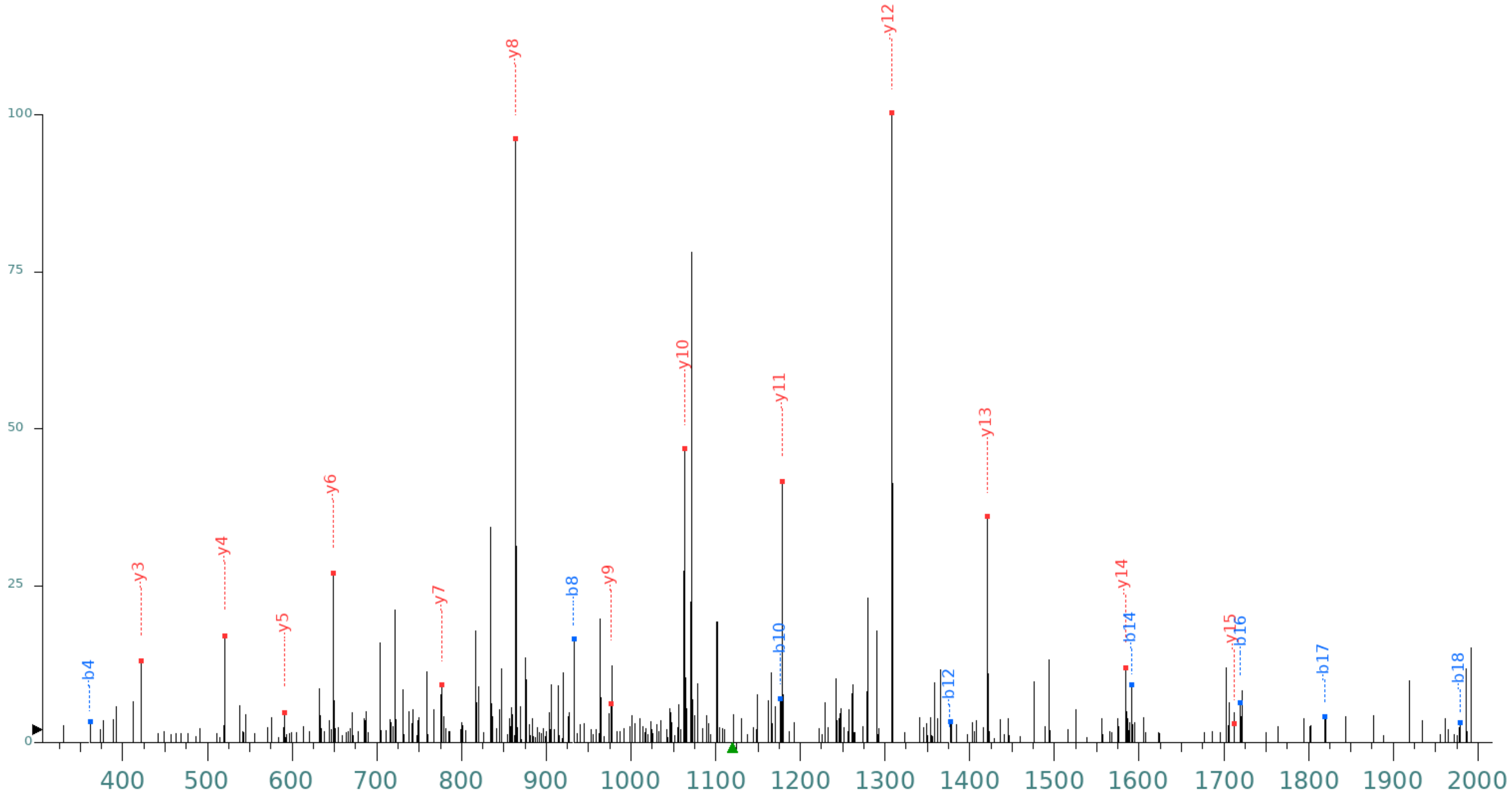

Predicted Fragmentation Pattern

Seq	#	b: Δ Error	b	y	y: Δ Error	+1
S	1	---	88.039	---	---	20
D	2	---	203.066	2151.890	---	19
T	3	---	304.114	2036.863	---	18
G	4	---	361.135	1935.815	---	17
S#	5	---	528.134	1878.794	---	16
Q	6	---	656.192	1711.796	---	15
Y	7	196.149	**819.256**	**1583.737**	184.682	14
I	8	218.214	**932.340**	**1420.674**	96.853	13
E	9	-9.304	**1061.382**	**1307.590**	99.502	12
D	10	251.504	**1176.409**	**1178.547**	172.309	11
S	11	141.622	**1263.441**	**1063.520**	49.076	10
I	12	149.613	**1376.525**	**976.488**	205.844	9
S	13	190.546	**1463.557**	**863.404**	53.668	8
Q	14	176.455	**1591.616**	**776.372**	138.344	7
G	15	---	1648.637	**648.313**	27.087	6
A	16	-2.964	**1719.675**	**591.292**	818.859	5
V	17	138.163	**1818.743**	**520.255**	511.766	4
C	18	---	1978.774	**421.186**	306.374	3
N	19	---	2092.817	261.156	---	2
K	20	---	---	147.113	---	1

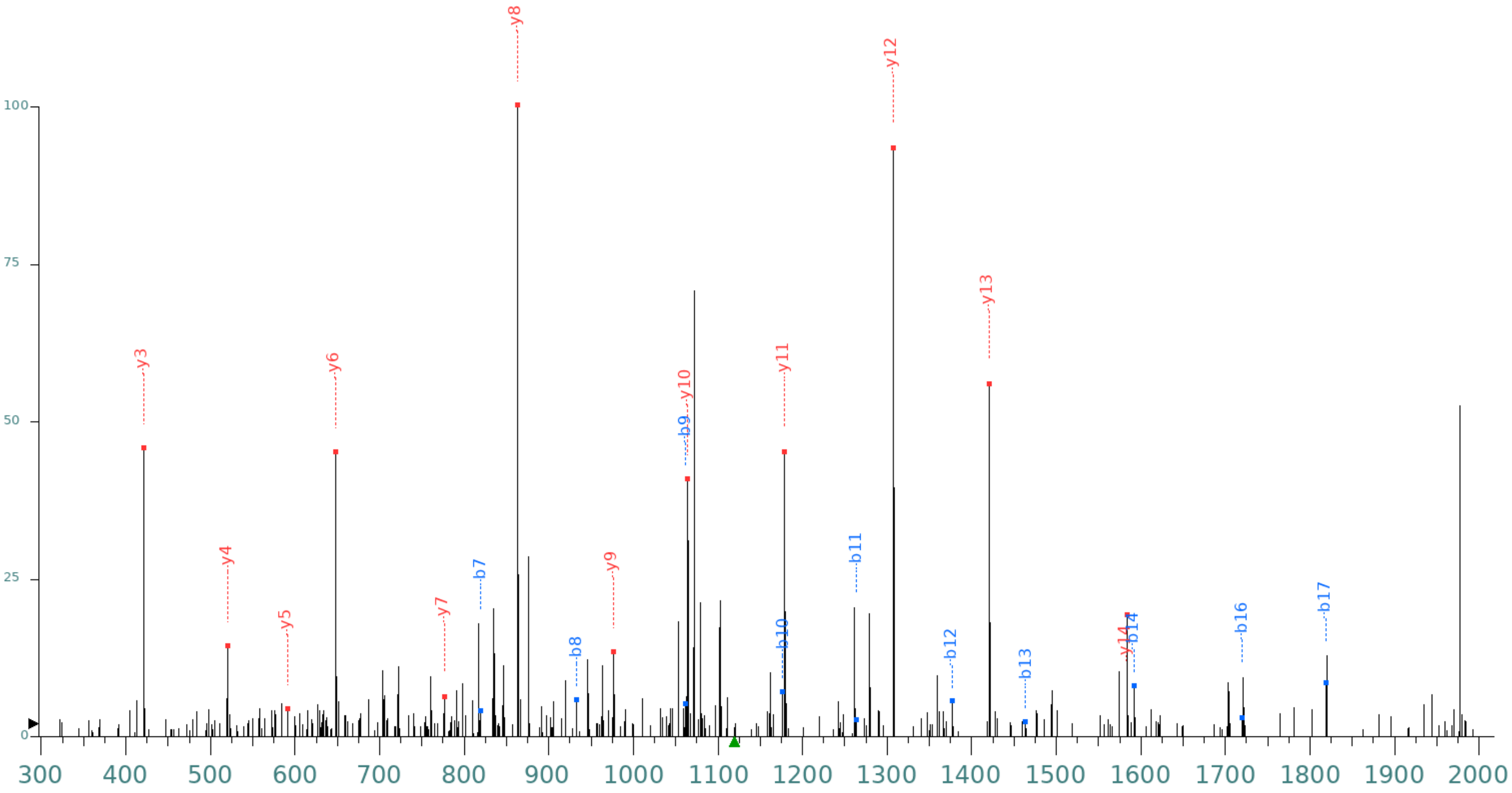

Predicted Fragmentation Pattern

+1

Seq	#	b: Δ Error	b	y	y: Δ Error	+1
T	1	---	102.055	---	---	14
N	2	-628.602	**216.098**	1434.674	---	13
V	3	-265.112	**315.166**	1320.631	---	12
L	4	-37.755	**428.250**	1221.562	---	11
S	5	---	515.282	**1108.478**	354.578	10
P	6	71.848	**612.335**	1021.446	---	9
H	7	---	749.394	**924.393**	124.784	8
T	8	---	850.442	**787.335**	454.869	7
S	9	---	937.474	**686.287**	248.023	6
G	10	---	994.495	**599.255**	126.408	5
S#	11	---	1161.494	542.233	---	4
I	12	---	1274.578	**375.235**	757.012	3
S	13	---	1361.610	**262.151**	-468.120	2
R	14	---	---	**175.119**	-64.455	1

+2

Seq	#	b: Δ Error	b	y	y: Δ Error	+1
T	1	---	51.531	---	---	14
N	2	---	108.553	**717.840**	86.768	13
V	3	---	158.087	**660.819**	19.233	12
L	4	1753.138	**214.629**	**611.285**	477.310	11
S	5	591.470	**258.145**	**554.743**	65.607	10
P	6	1228.527	**306.671**	511.227	---	9
H	7	848.552	**375.201**	**462.700**	648.311	8
T	8	-558.494	**425.725**	394.171	---	7
S	9	-468.575	**469.241**	**343.647**	-750.584	6
G	10	655.037	**497.751**	**300.131**	511.705	5
S#	11	---	581.250	**271.620**	-1061.667	4
I	12	---	637.792	**188.121**	-29.312	3
S	13	---	681.308	131.579	---	2
R	14	---	---	88.063	---	1

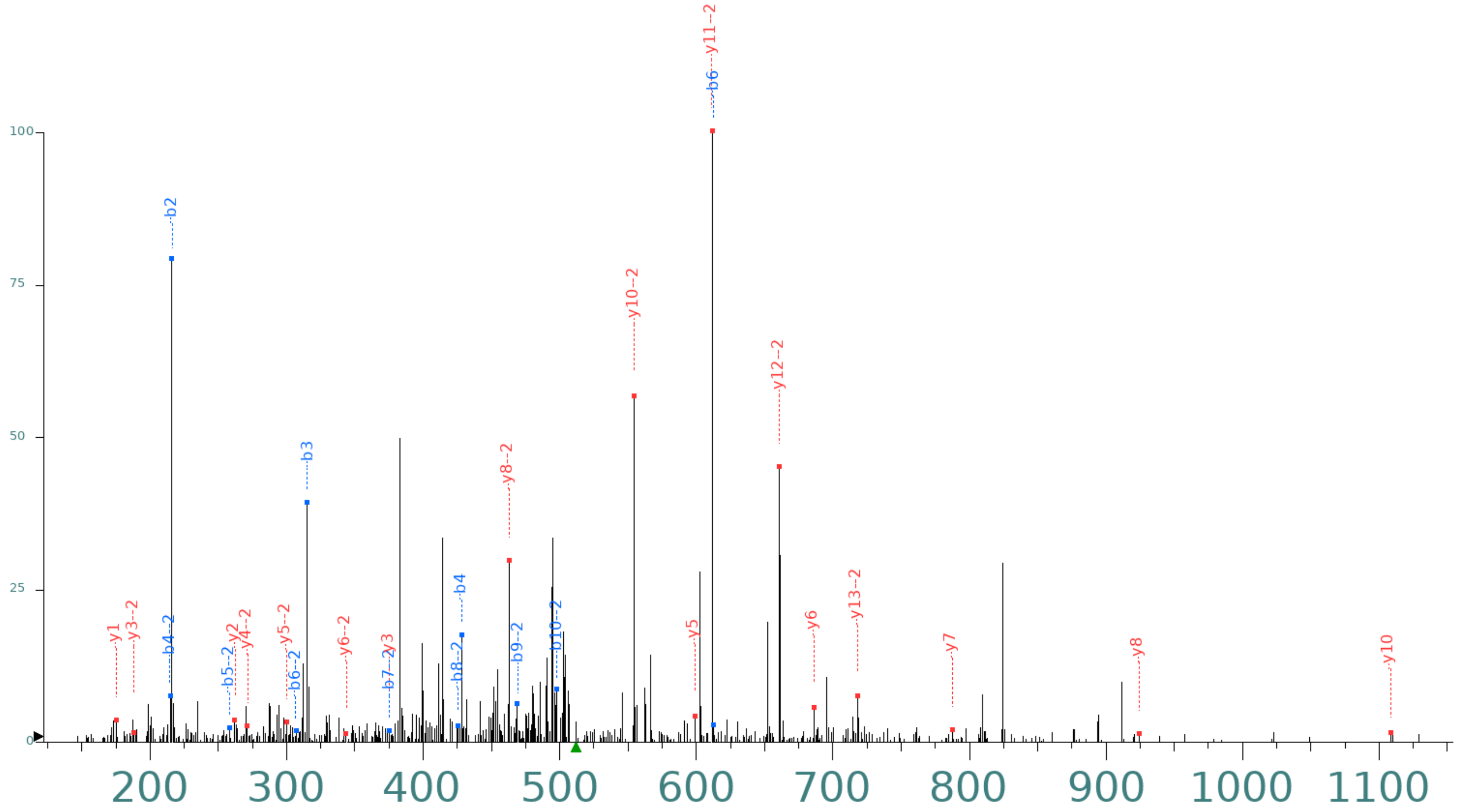

Predicted Fragmentation Pattern

Seq	#	b: Δ Error	b	y	y: Δ Error	+1
T	1	---	102.055	---	---	14
N	2	248.643	216.098	1434.674	---	13
V	3	394.506	315.166	1320.631	---	12
L	4	341.310	428.250	1221.562	175.275	11
S#	5	---	595.249	1108.478	-8.376	10
P	6	605.532	692.301	941.480	158.611	9
H	7	298.500	829.360	844.427	351.047	8
T	8	165.885	930.408	707.368	84.094	7
S	9	319.902	1017.440	606.321	495.112	6
G	10	---	1074.462	519.289	159.073	5
S	11	---	1161.494	462.267	125.981	4
I	12	---	1274.578	375.235	178.734	3
S	13	270.984	1361.610	262.151	-197.514	2
R	14	---	---	175.119	---	1

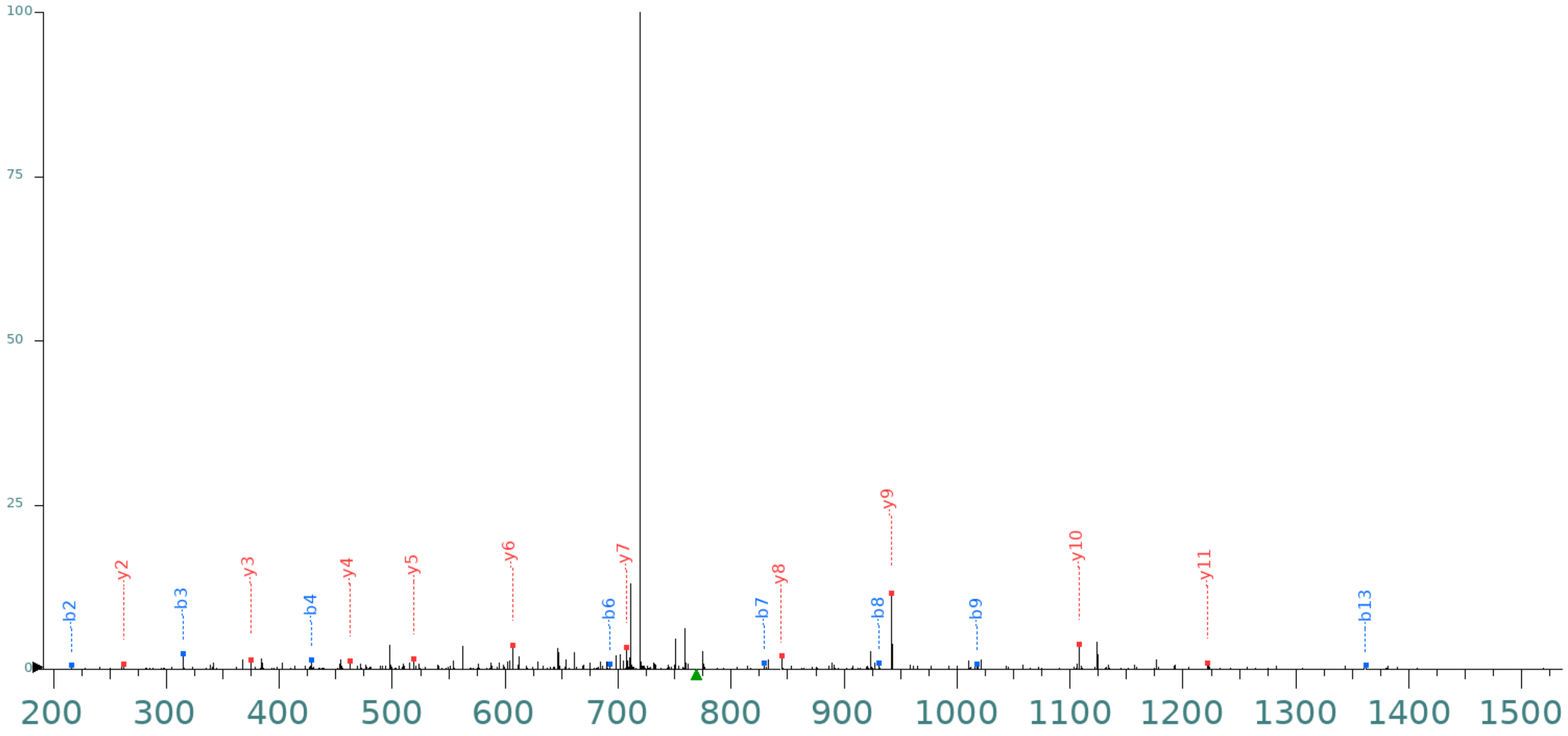

Predicted Fragmentation Pattern

Seq	#	b: Δ Error	b	y	y: Δ Error	+1
I	1	---	114.091	---	---	8
P	2	-745.485	211.144	839.402	52.014	7
S#	3	192.263	378.142	742.349	83.927	6
T	4	---	479.190	575.351	196.742	5
V	5	94.546	578.259	474.303	---	4
L	6	190.794	691.343	375.235	-142.749	3
S	7	---	778.375	262.151	-383.416	2
R	8	---	---	175.119	29.217	1

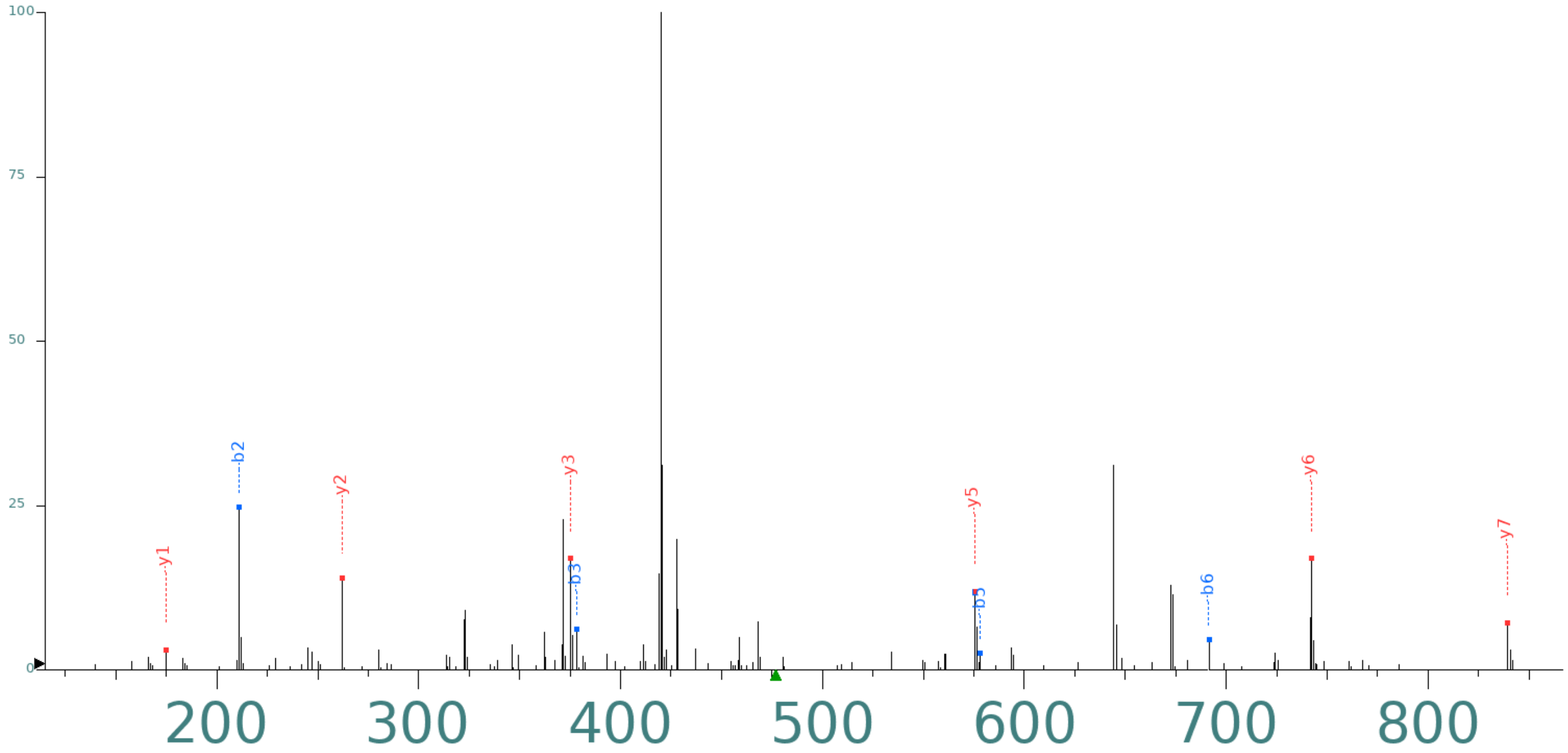

Predicted Fragmentation Pattern

Seq	#	b: Δ Error	b	y	y: Δ Error	+1
I	1	---	114.091	---	---	8
P	2	-696.562	211.144	839.402	95.635	7
S#	3	-186.478	378.142	742.349	136.699	6
T	4	---	479.190	575.351	85.492	5
V	5	269.484	578.259	474.303	---	4
L	6	440.748	691.343	375.235	259.621	3
S	7	---	778.375	262.151	-430.485	2
R	8	---	---	175.119	-691.857	1

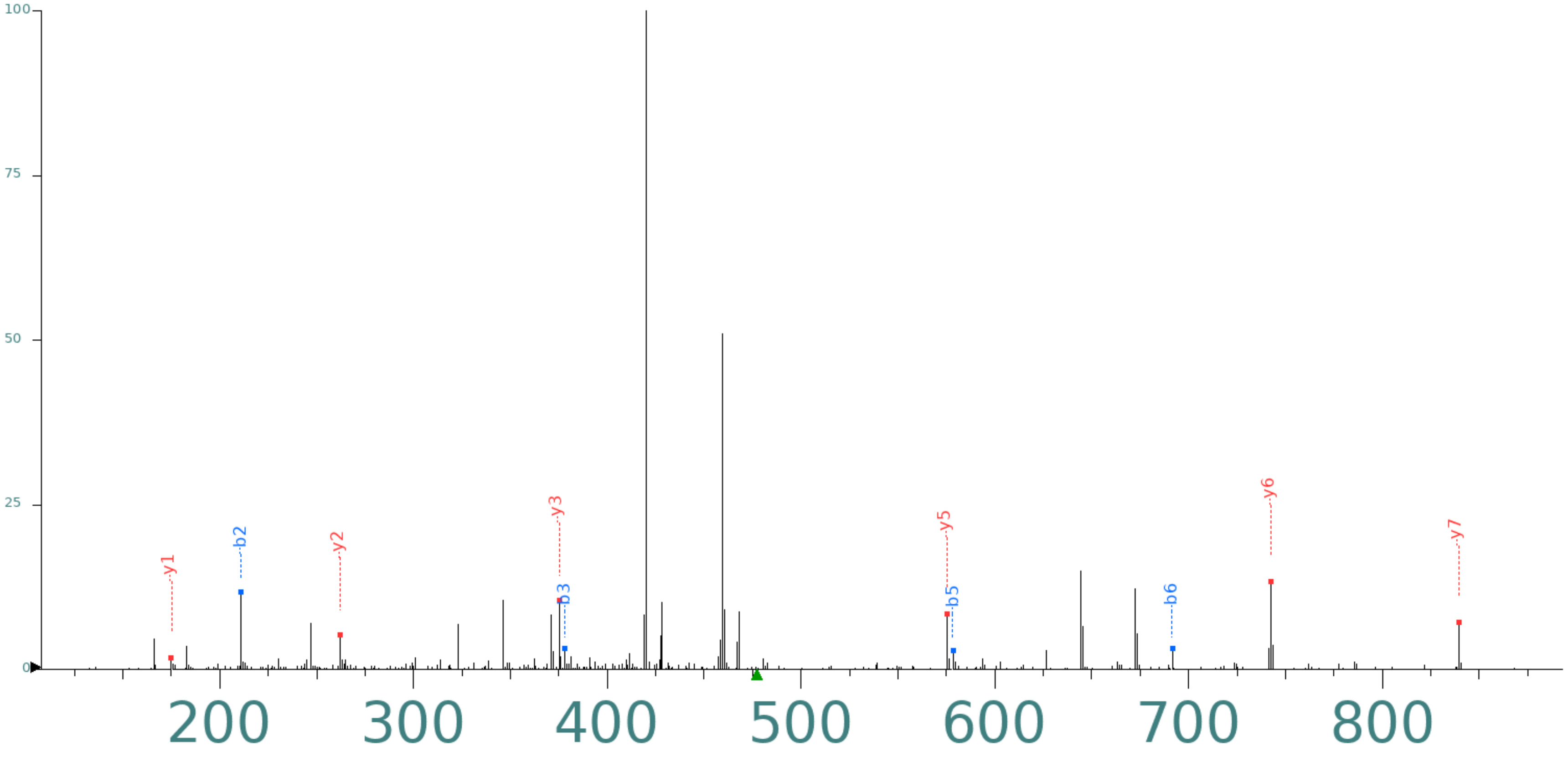

Predicted Fragmentation Pattern

+1

Seq	#	b: Δ Error	b	y	y: Δ Error	+1
R	1	---	157.108	---	---	12
P	2	-536.479	254.161	1292.672	---	11
S#	3	---	421.160	1195.619	---	10
R	4	104.874	577.261	1028.621	---	9
I	5	584.038	690.345	872.520	---	8
P	6	---	787.397	759.436	17.016	7
S	7	---	874.429	662.383	348.114	6
T	8	---	975.477	575.351	-97.928	5
V	9	---	1074.546	474.303	574.645	4
L	10	---	1187.630	375.235	177.352	3
S	11	---	1274.662	262.151	---	2
R	12	---	---	175.119	535.005	1

+2

Seq	#	b: Δ Error	b	y	y: Δ Error	+1
R	1	---	79.058	---	---	12
P	2	---	127.584	646.840	-303.314	11
S#	3	---	211.083	598.313	764.276	10
R	4	-695.753	289.134	514.814	-16.918	9
I	5	-508.643	345.676	436.764	-1059.248	8
P	6	---	394.202	380.222	-98.049	7
S	7	-931.608	437.718	331.695	657.770	6
T	8	---	488.242	288.179	---	5
V	9	---	537.776	237.655	90.511	4
L	10	672.160	594.318	188.121	---	3
S	11	---	637.834	131.579	---	2
R	12	---	---	88.063	---	1

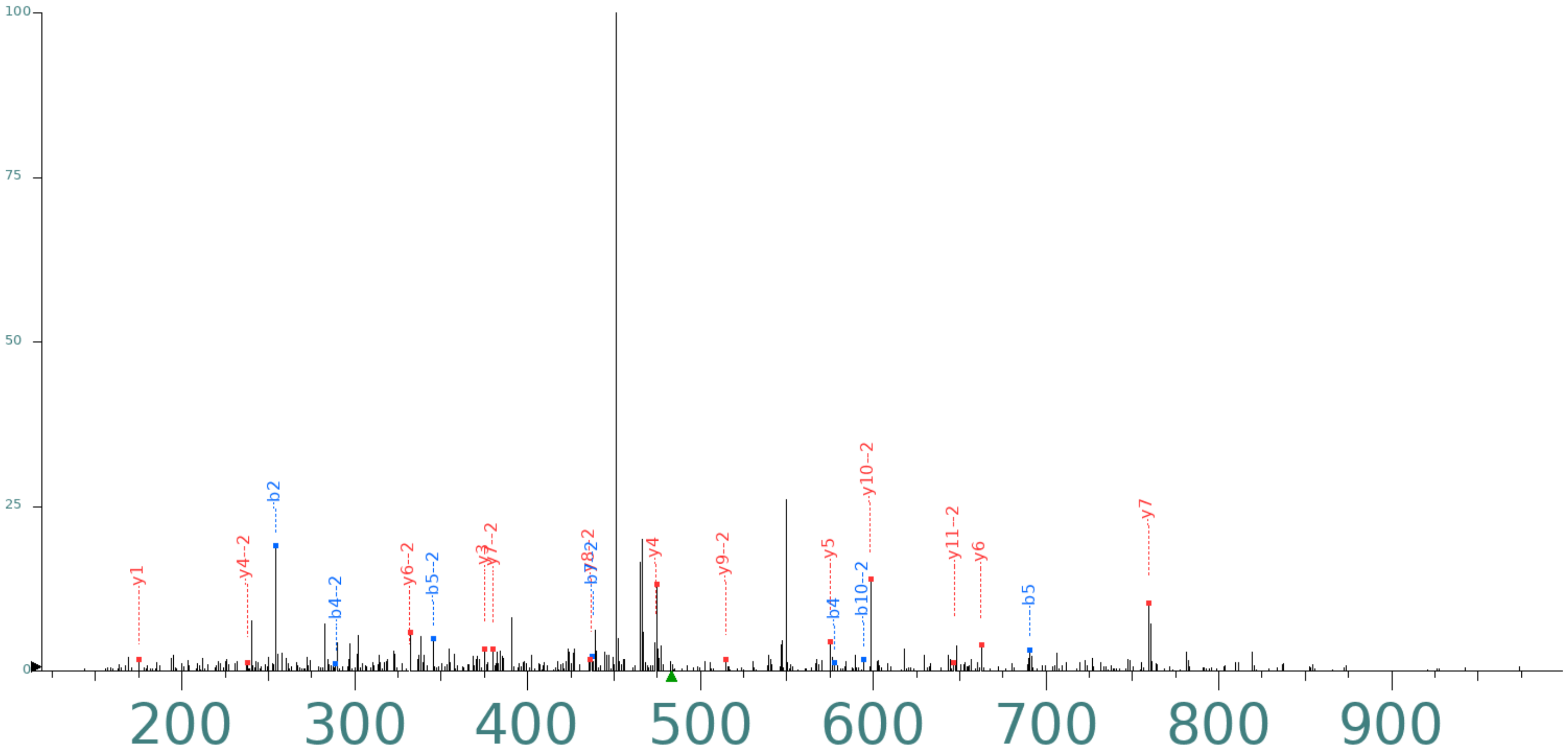

Predicted Fragmentation Pattern

Seq	#	b: Δ Error	b	y	y: Δ Error	+1
I	1	---	114.091	---	---	8
P	2	-536.365	211.144	839.402	413.373	7
S#	3	157.330	378.142	742.349	67.321	6
T	4	---	479.190	575.351	402.951	5
V	5	171.577	578.259	474.303	---	4
L	6	591.294	691.343	375.235	-176.756	3
S	7	---	778.375	262.151	-381.086	2
R	8	---	---	175.119	8.219	1

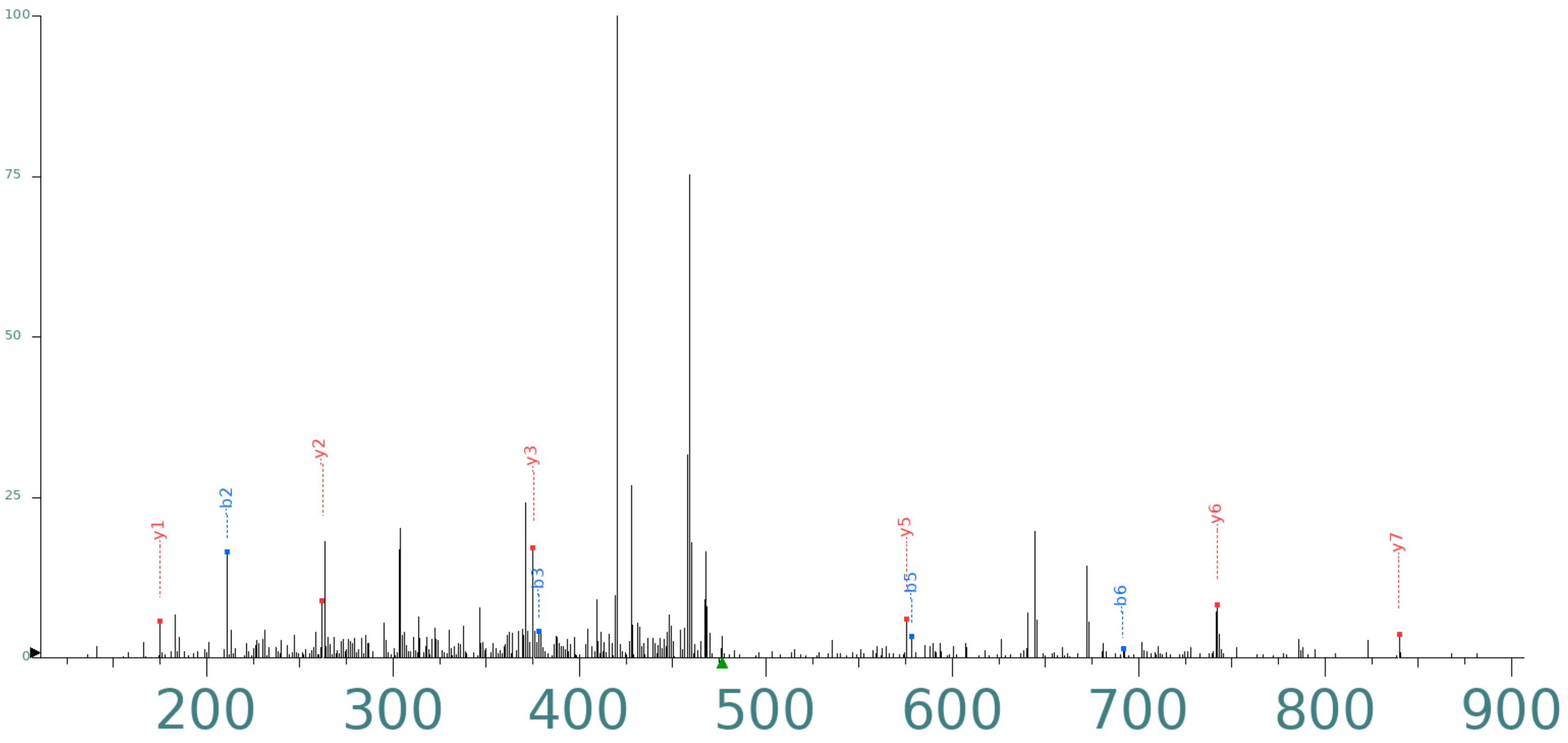

Predicted Fragmentation Pattern

Seq	#	b: Δ Error	b	y	y: Δ Error	+1
I	1	---	114.091	---	---	8
P	2	-47.698	211.144	839.402	253.003	7
S#	3	417.612	378.142	742.349	---	6
T	4	---	479.190	575.351	12.408	5
V	5	46.528	578.259	474.303	338.148	4
L	6	---	691.343	375.235	181.254	3
S	7	---	778.375	262.151	189.210	2
R	8	---	---	175.119	838.337	1

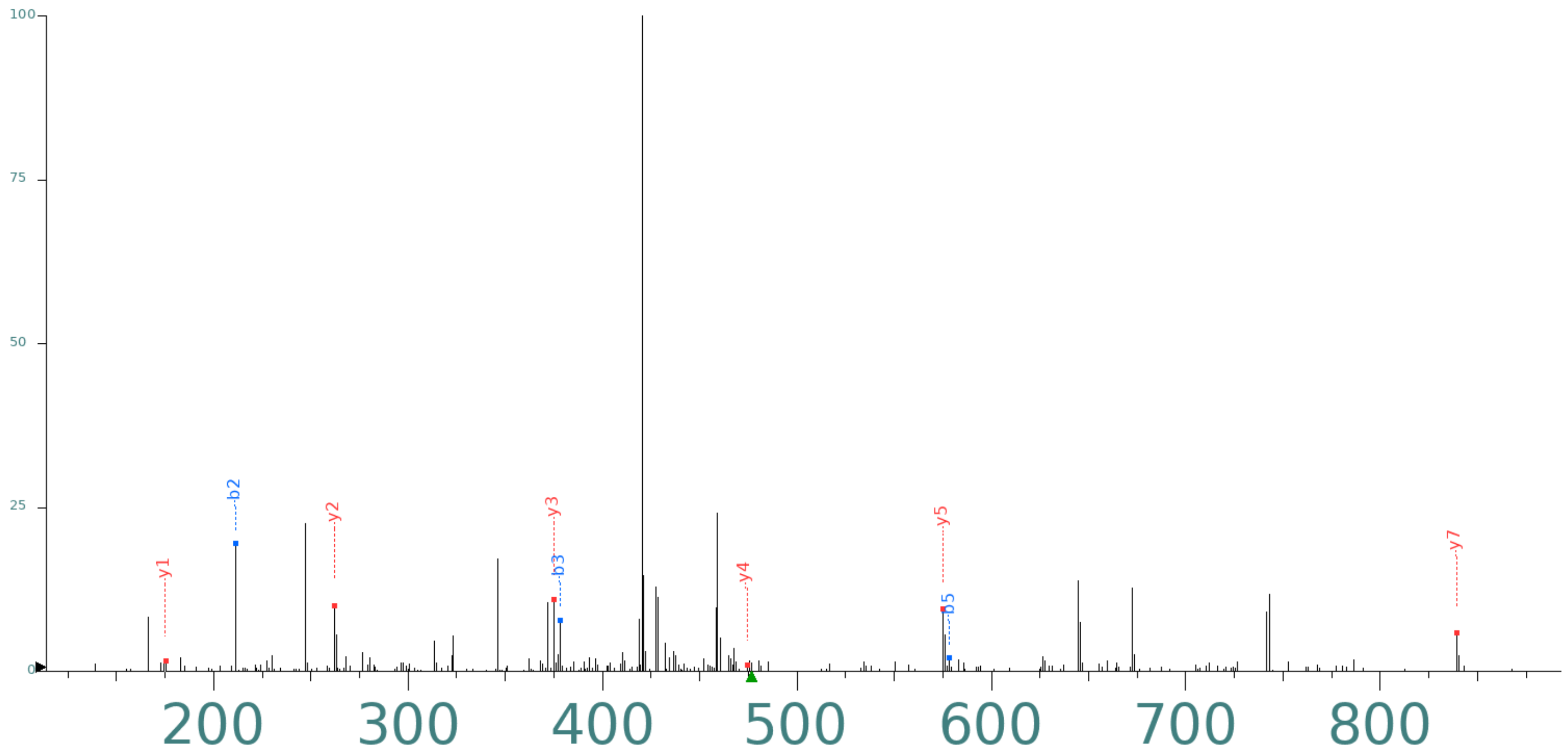

Predicted Fragmentation Pattern

Seq	#	b: Δ Error	b	y	y: Δ Error	+1
I	1	---	114.091	---	---	8
P	2	476.737	211.144	839.402	340.061	7
S#	3	---	378.142	742.349	666.667	6
T	4	---	479.190	575.351	214.981	5
V	5	---	578.259	474.303	---	4
L	6	234.830	691.343	375.235	455.403	3
S	7	---	778.375	262.151	48.036	2
R	8	---	---	175.119	730.373	1

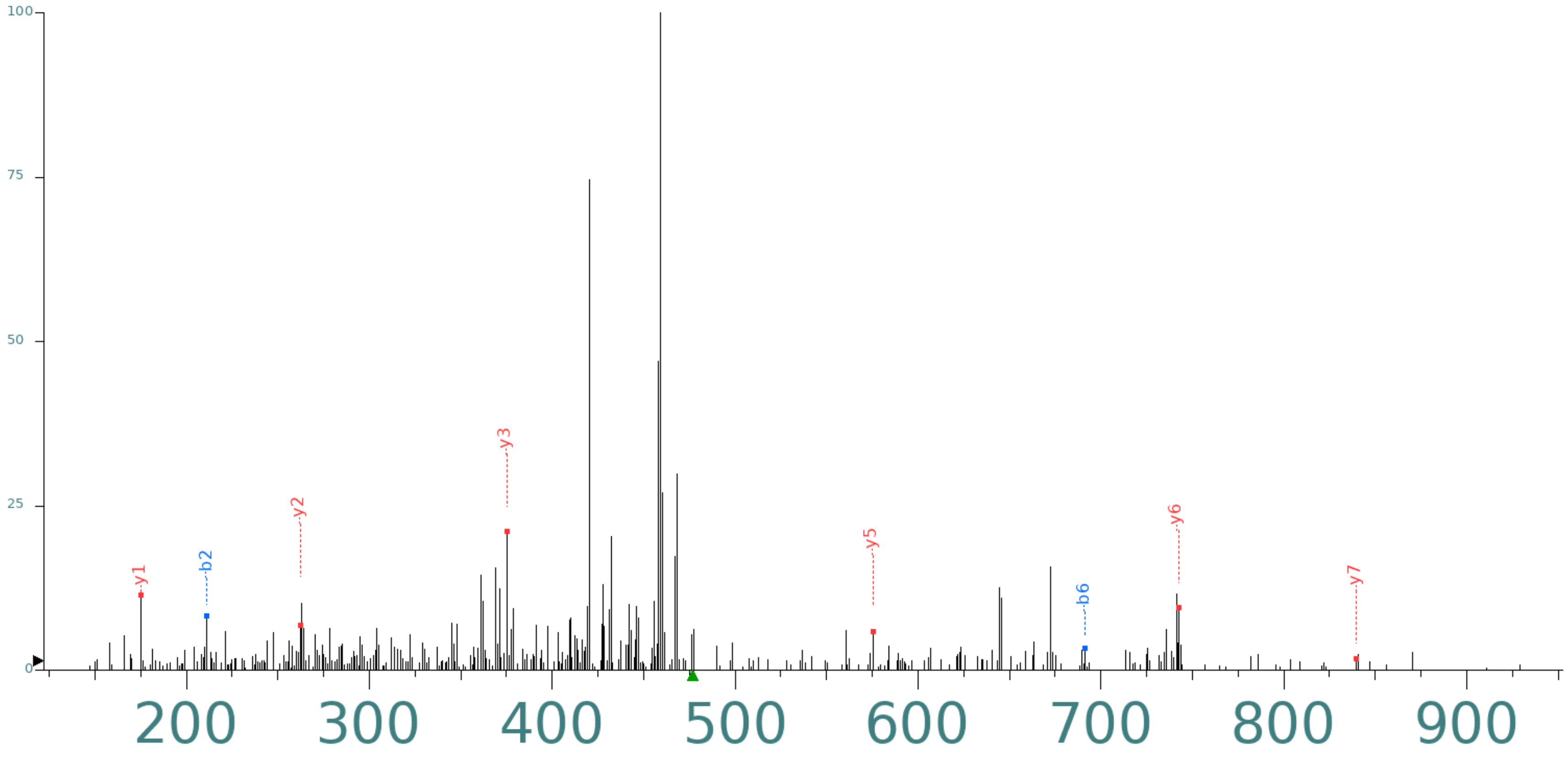

LPA3-PMA 18 March

Predicted Fragmentation Pattern

Seq	#	b: Δ Error	b	y	y: Δ Error	+1
S	1	---	88.039	---	---	20
D	2	---	203.066	2151.890	---	19
T	3	---	304.114	2036.863	---	18
G	4	---	361.135	1935.815	---	17
S	5	---	448.167	1878.794	---	16
Q	6	---	576.226	1791.762	---	15
Y#	7	34.072	819.256	1663.703	---	14
I	8	-32.730	932.340	1420.674	32.847	13
E	9	73.958	1061.382	1307.590	56.565	12
D	10	143.734	1176.409	1178.547	85.844	11
S	11	210.969	1263.441	1063.520	-23.807	10
I	12	146.598	1376.525	976.488	35.247	9
S	13	297.671	1463.557	863.404	35.573	8
Q	14	115.423	1591.616	776.372	291.893	7
G	15	102.264	1648.637	648.313	-10.571	6
A	16	71.778	1719.675	591.292	120.899	5
V	17	175.134	1818.743	520.255	211.650	4
C	18	196.119	1978.774	421.186	-235.850	3
N	19	---	2092.817	261.156	---	2
K	20	---	---	147.113	---	1

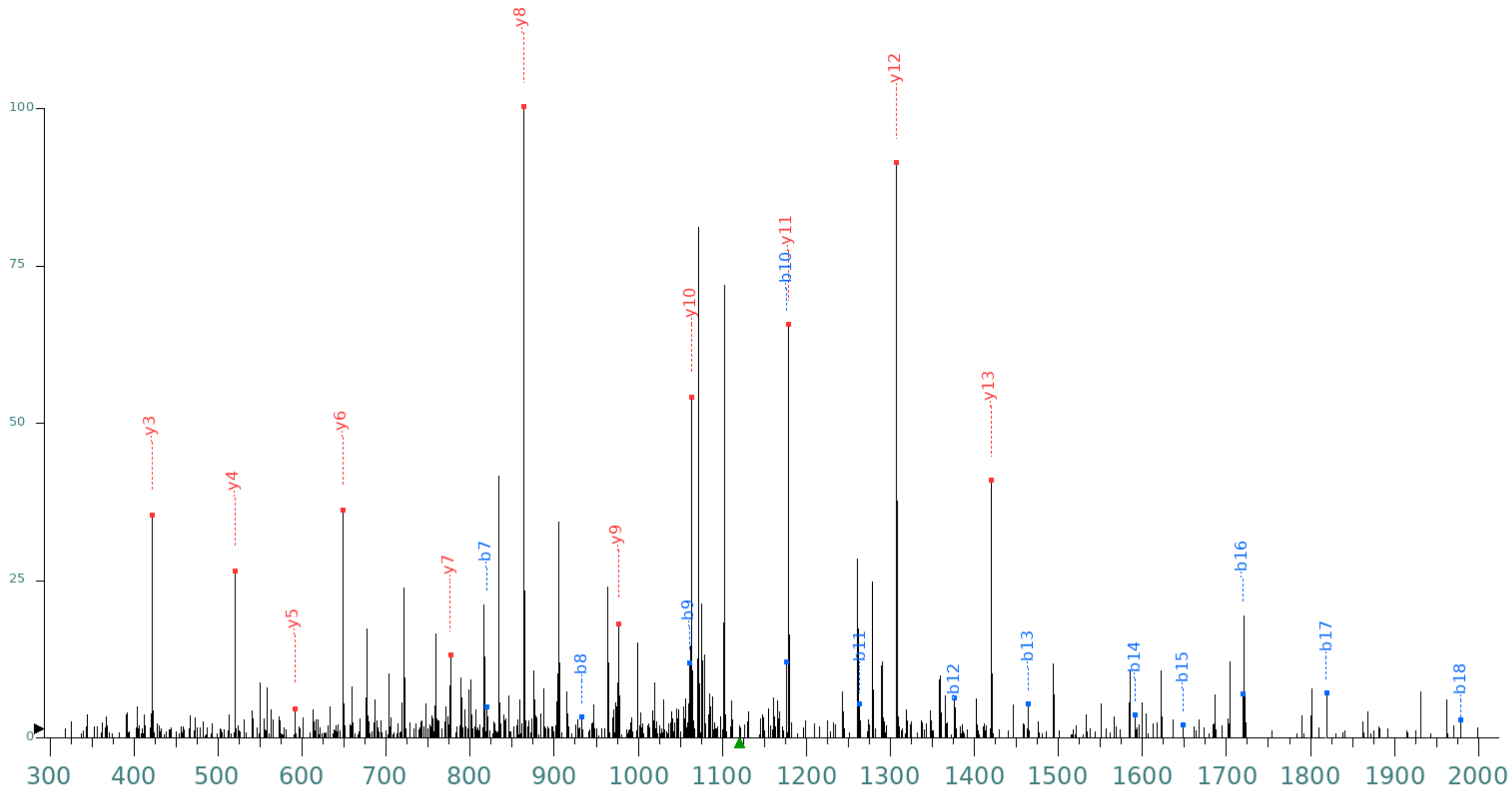

Predicted Fragmentation Pattern

Seq	#	b: Δ Error	b	y	y: Δ Error	+1
S	1	---	88.039	---	---	20
D	2	---	203.066	2151.890	---	19
T#	3	---	384.080	2036.863	---	18
G	4	---	441.102	1855.849	---	17
S	5	-69.500	**528.134**	1798.828	---	16
Q	6	---	656.192	1711.796	---	15
Y	7	156.528	**819.256**	1583.737	---	14
I	8	---	932.340	**1420.674**	96.079	13
E	9	-415.234	**1061.382**	**1307.590**	43.590	12
D	10	107.114	**1176.409**	**1178.547**	169.617	11
S	11	---	1263.441	**1063.520**	84.538	10
I	12	204.220	**1376.525**	**976.488**	152.109	9
S	13	---	1463.557	**863.404**	75.156	8
Q	14	260.247	**1591.616**	**776.372**	138.972	7
G	15	---	1648.637	**648.313**	-84.575	6
A	16	---	1719.675	591.292	---	5
V	17	---	1818.743	**520.255**	254.451	4
C	18	194.145	**1978.774**	**421.186**	-146.985	3
N	19	---	2092.817	261.156	---	2
K	20	---	---	147.113	---	1

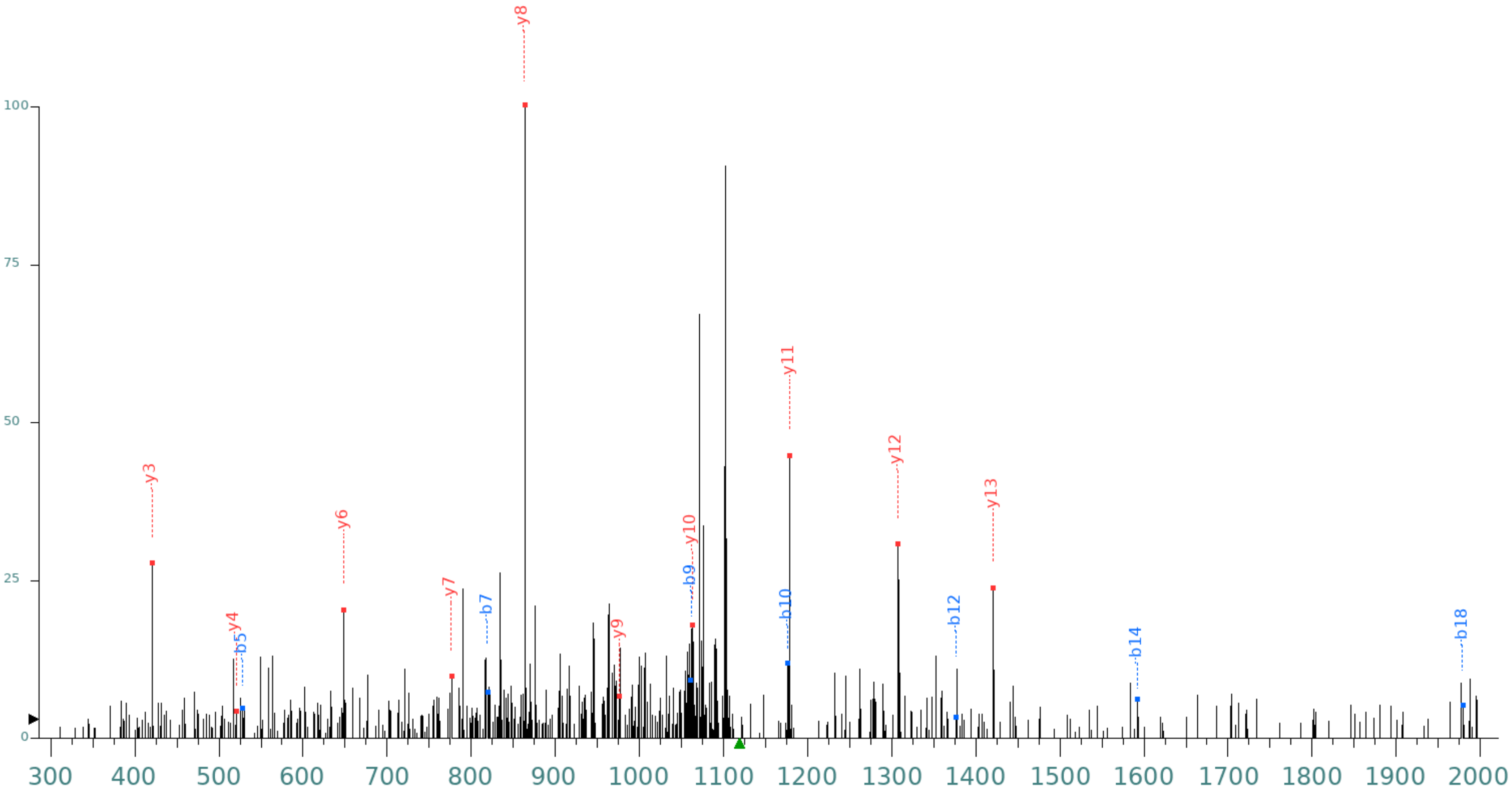

Predicted Fragmentation Pattern

Seq	#	b: Δ Error	b	y	y: Δ Error	+1
I	1	---	114.091	---	---	8
P	2	-82.101	211.144	839.402	317.753	7
S#	3	640.361	378.142	742.349	240.503	6
T	4	---	479.190	575.351	272.662	5
V	5	114.491	578.259	474.303	---	4
L	6	415.872	691.343	375.235	-115.334	3
S	7	---	778.375	262.151	139.752	2
R	8	---	---	175.119	228.790	1

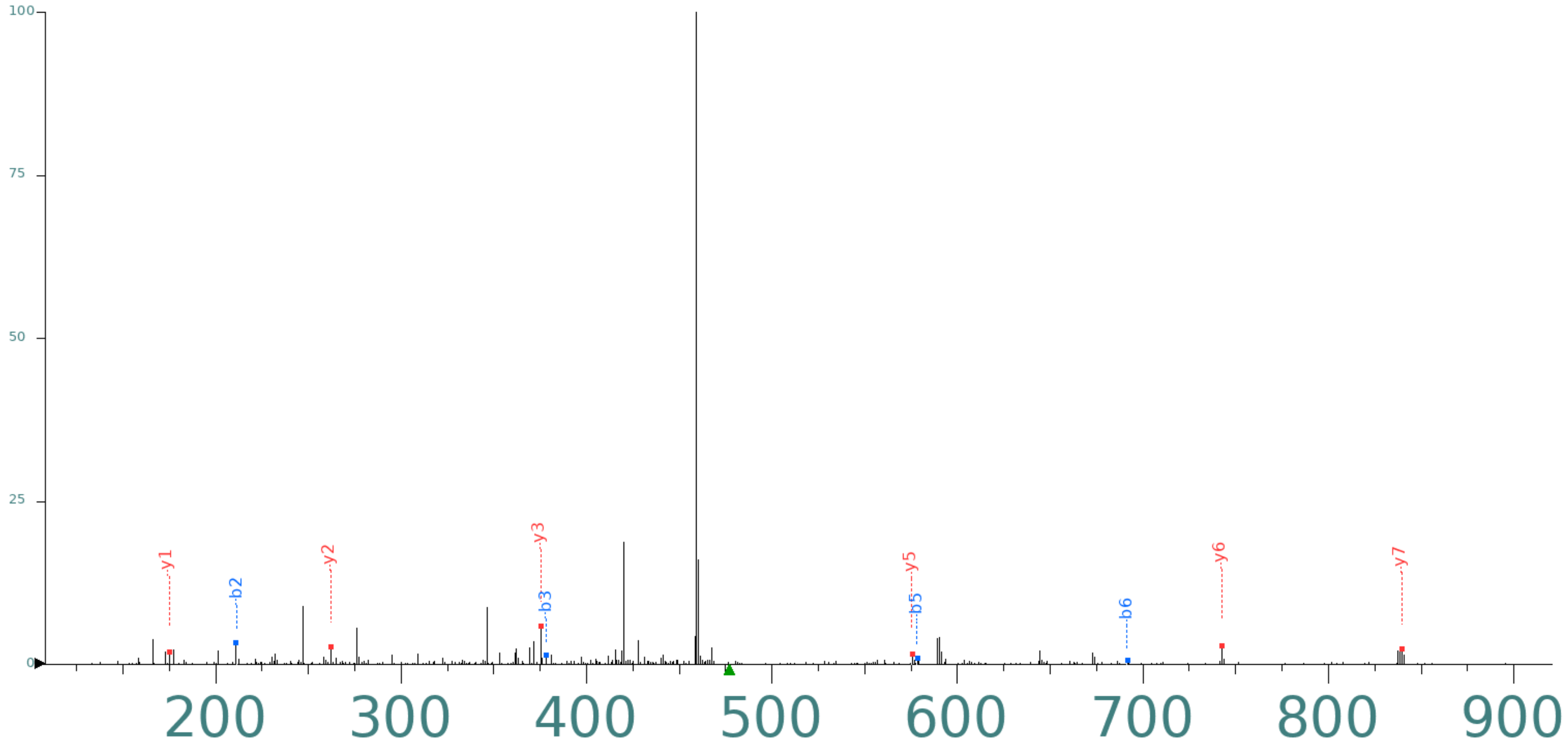

LPA3-LPA

Predicted Fragmentation Pattern

Seq	#	b: Δ Error	b	y	y: Δ Error	+1
I	1	---	114.091	---	---	8
P	2	-336.010	211.144	839.402	2.863	7
S#	3	434.223	378.142	742.349	55.647	6
T	4	---	479.190	575.351	258.667	5
V	5	430.652	578.259	474.303	571.239	4
L	6	9.317	691.343	375.235	26.842	3
S	7	-41.186	778.375	262.151	-195.068	2
R	8	---	---	175.119	491.222	1

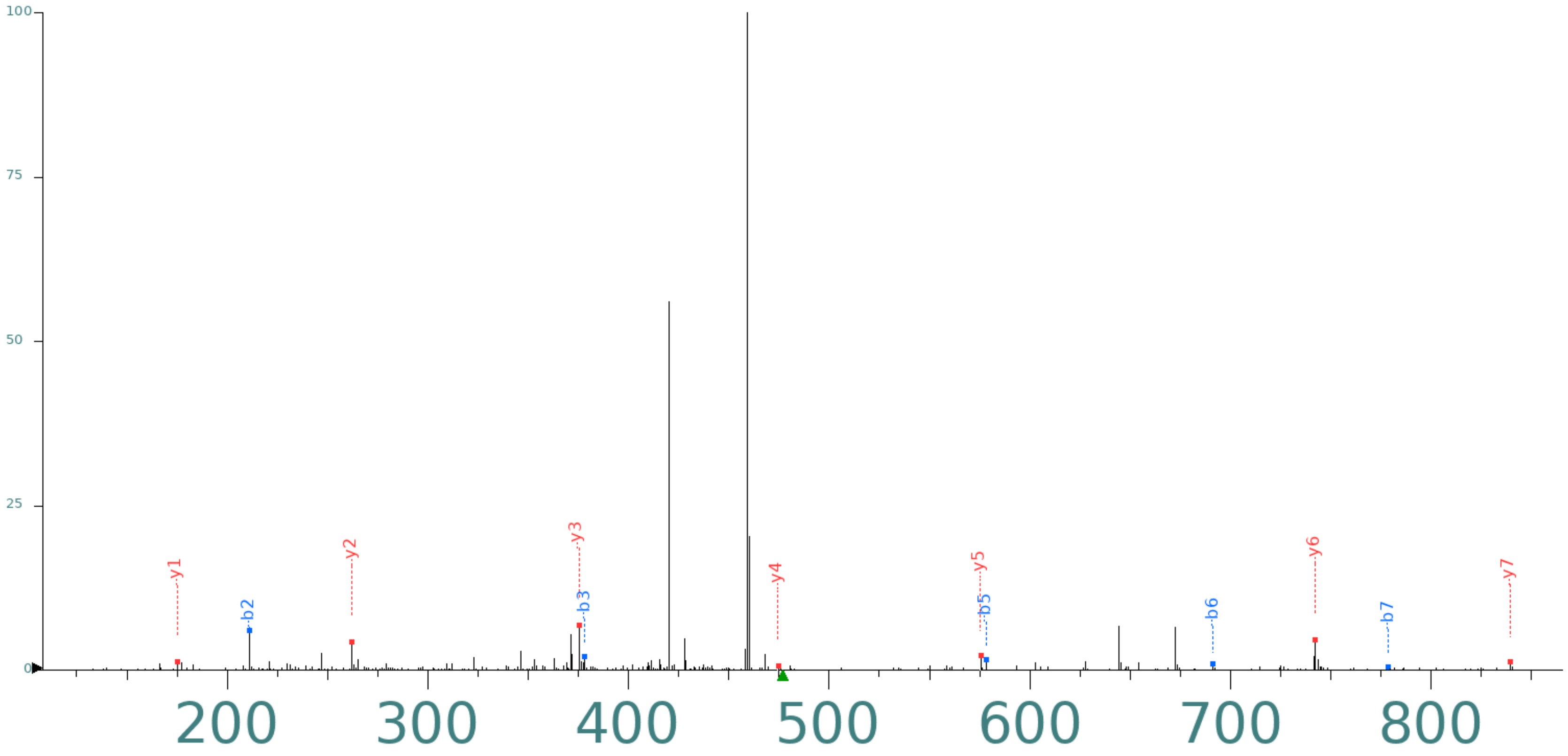

Predicted Fragmentation Pattern

Seq	#	b: Δ Error	b	y	y: Δ Error	+1
I	1	---	114.091	---	---	8
P	2	-925.296	211.144	839.402	130.166	7
S#	3	23.548	378.142	742.349	233.517	6
T	4	---	479.190	575.351	144.355	5
V	5	199.960	578.259	474.303	209.148	4
L	6	-44.009	691.343	375.235	-251.448	3
S	7	---	778.375	262.151	-421.280	2
R	8	---	---	175.119	-174.705	1

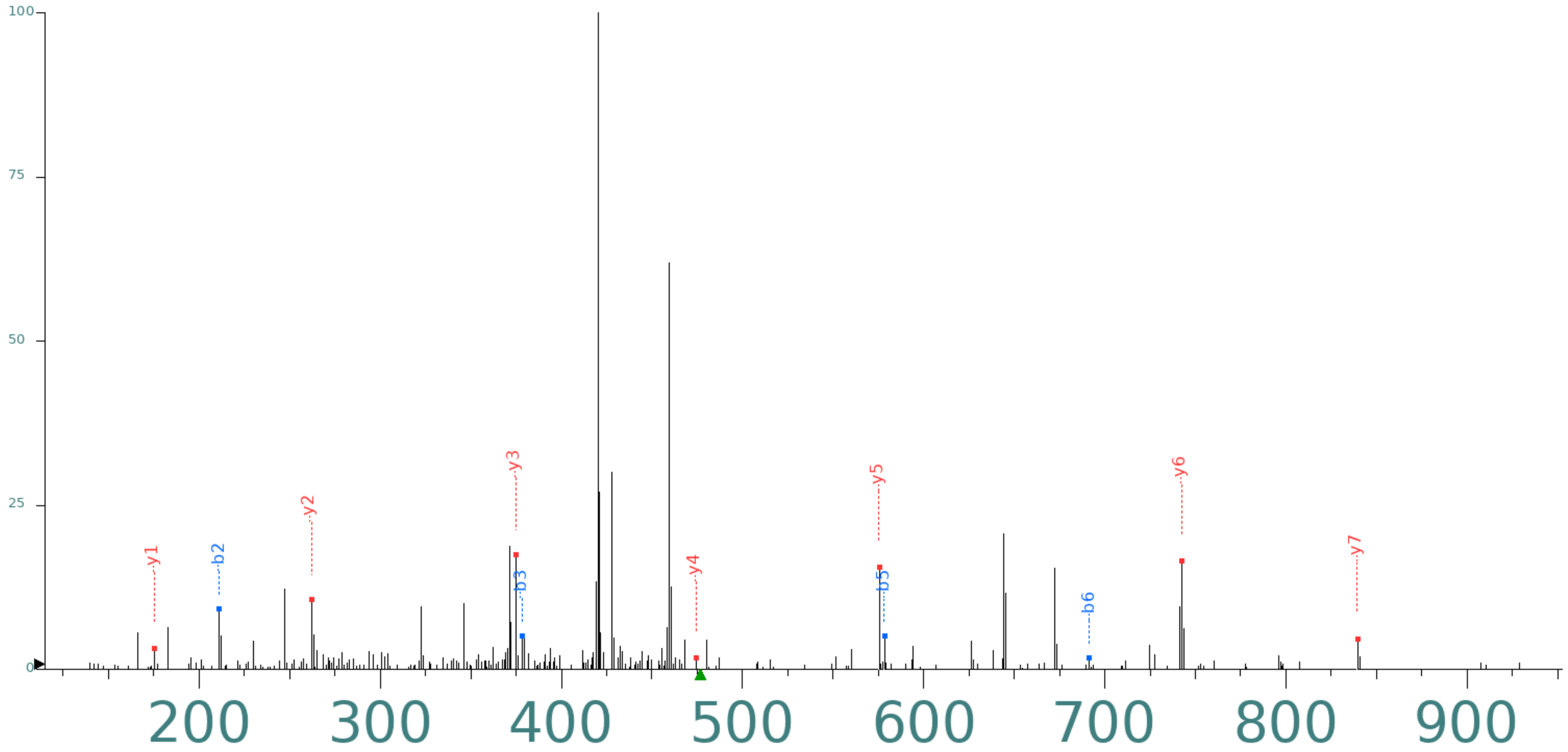

Predicted Fragmentation Pattern

Seq	#	b: Δ Error	b	y	y: Δ Error	+1
I	1	---	114.091	---	---	8
P	2	-567.619	211.144	839.402	472.581	7
S#	3	362.938	378.142	742.349	102.669	6
T	4	---	479.190	575.351	343.906	5
V	5	679.473	578.259	474.303	---	4
L	6	241.713	691.343	375.235	6.673	3
S	7	---	778.375	262.151	-181.560	2
R	8	---	---	175.119	---	1

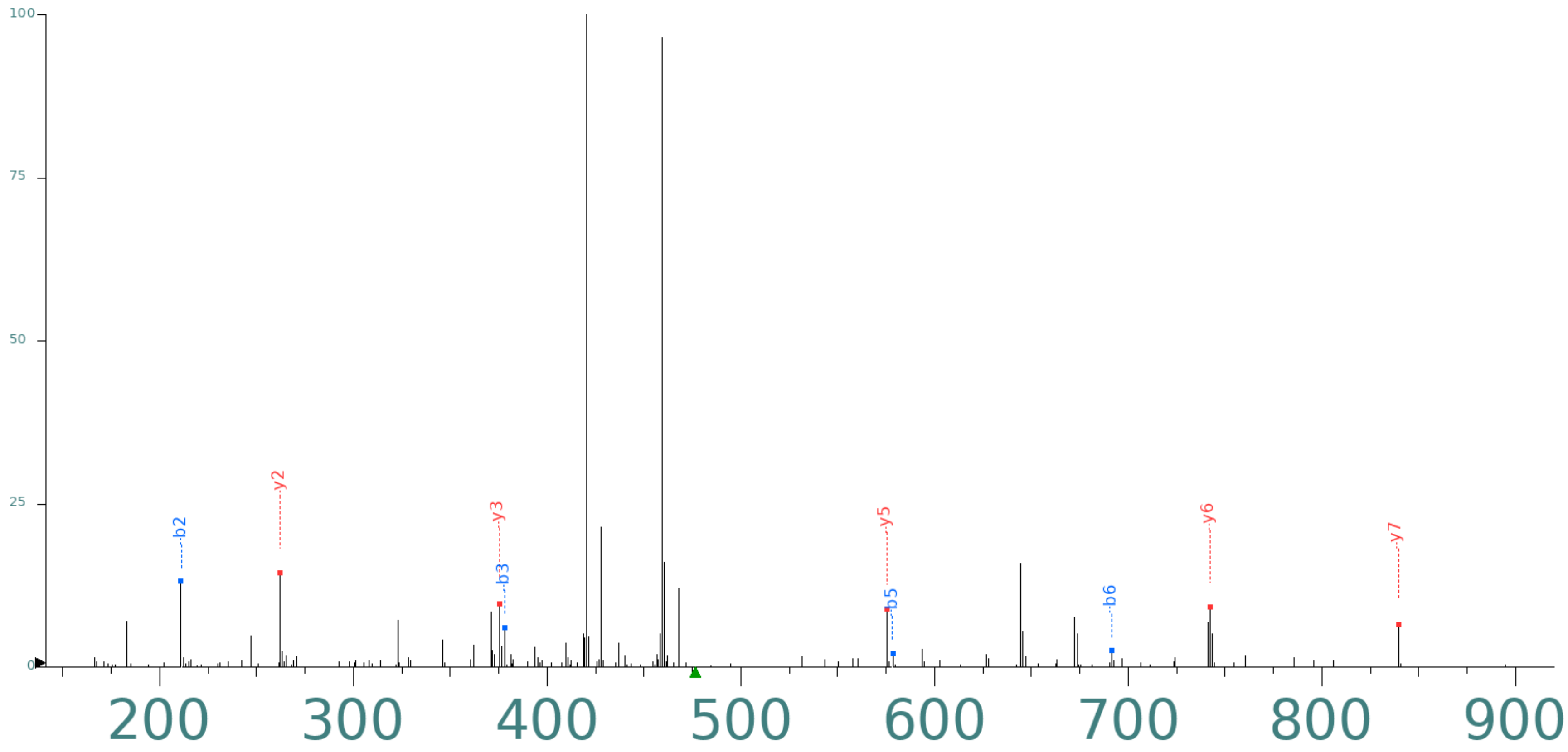

Predicted Fragmentation Pattern

Seq	#	b: Δ Error	b	y	y: Δ Error	+1
I	1	---	114.091	---	---	8
P	2	85.413	211.144	839.402	-190.371	7
S#	3	44.287	378.142	742.349	326.373	6
T	4	---	479.190	575.351	343.376	5
V	5	258.619	578.259	474.303	---	4
L	6	220.004	691.343	375.235	-227.200	3
S	7	---	778.375	262.151	-451.574	2
R	8	---	---	175.119	-18.618	1

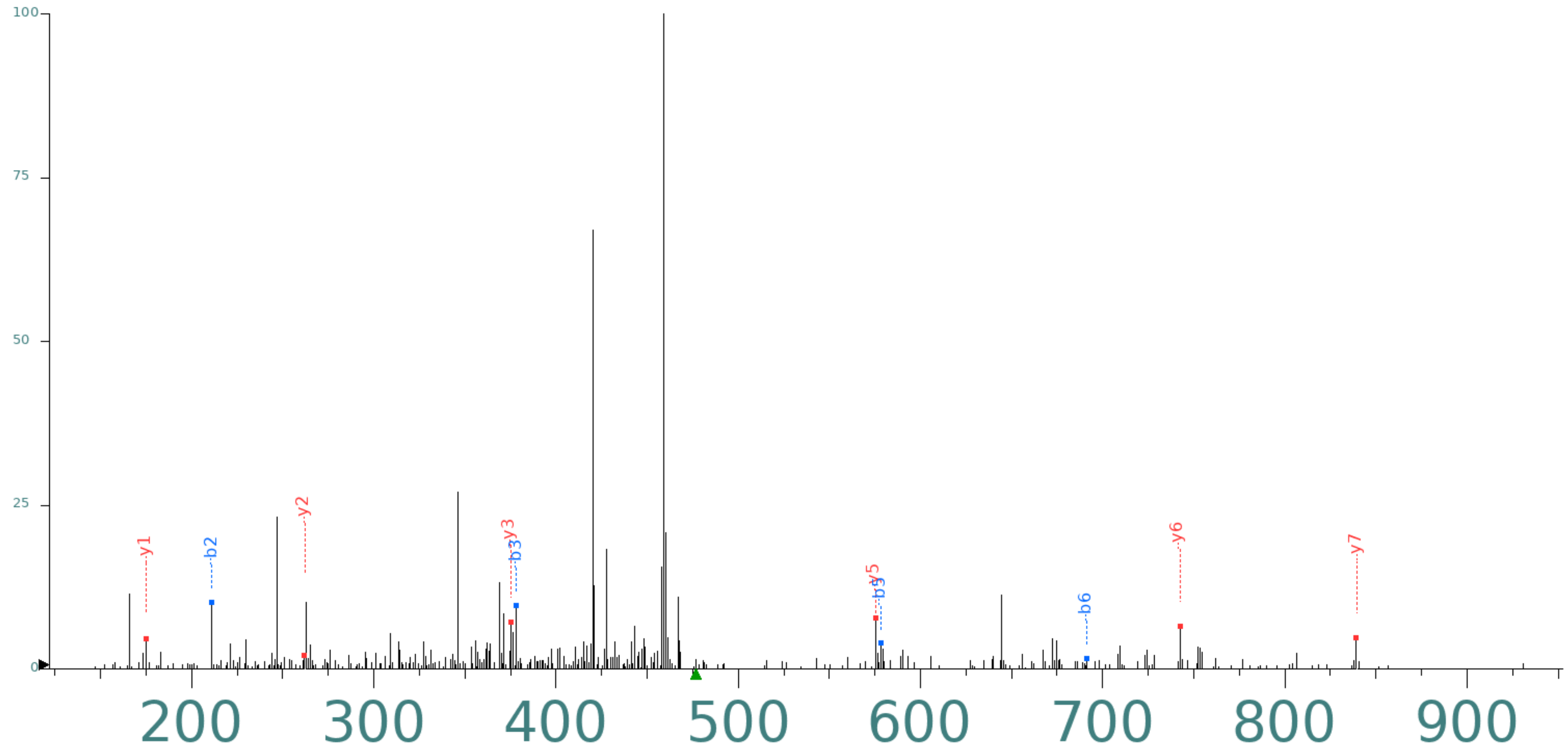

LPA3-LPA

Predicted Fragmentation Pattern

Seq	#	b: Δ Error	b	y	y: Δ Error	+1
I	1	---	114.091	---	---	8
P	2	-442.904	211.144	839.402	163.749	7
S#	3	57.198	378.142	742.349	53.674	6
T	4	---	479.190	575.351	-2.868	5
V	5	---	578.259	474.303	---	4
L	6	-22.731	691.343	375.235	-389.959	3
S	7	---	778.375	262.151	-297.678	2
R	8	---	---	175.119	271.638	1

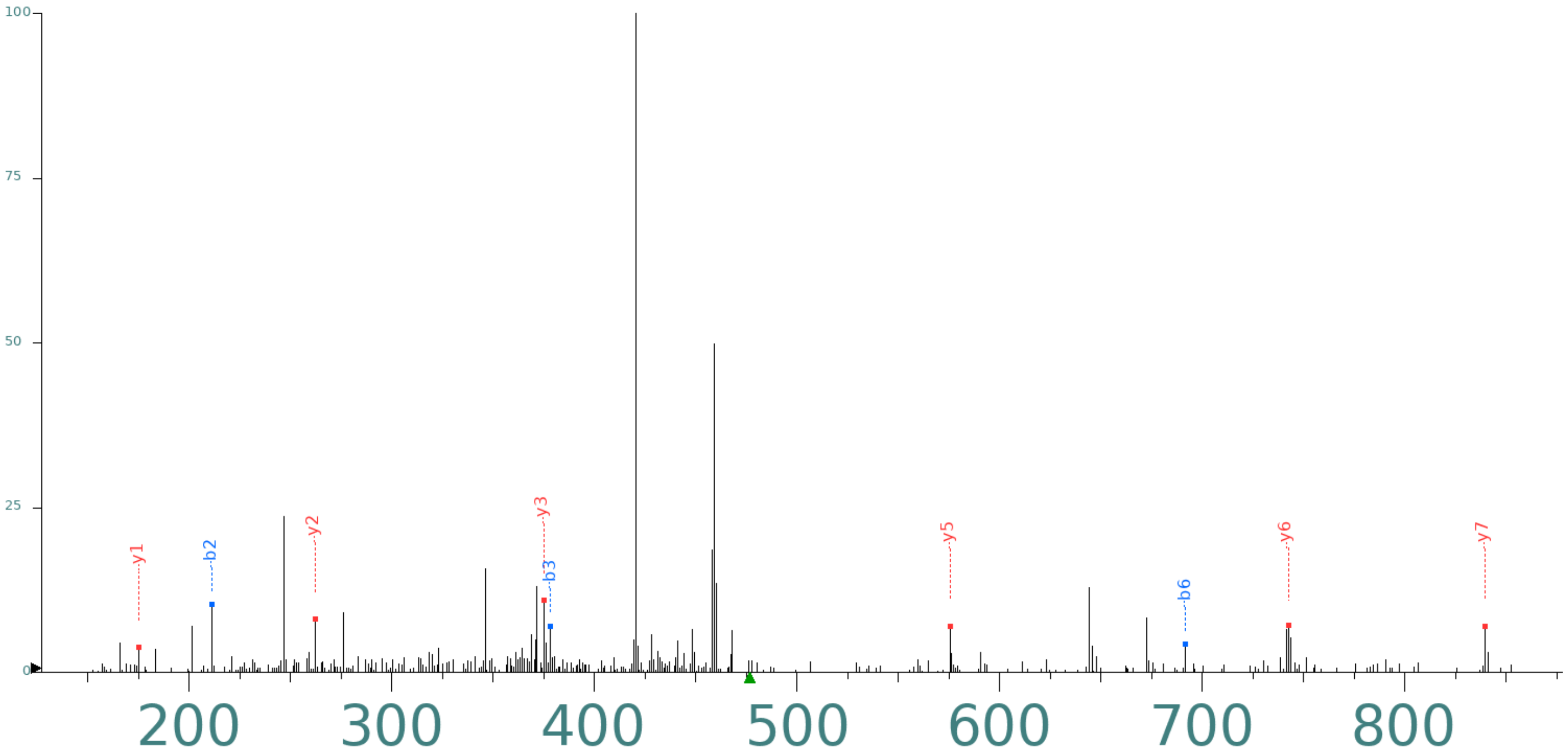

Predicted Fragmentation Pattern

Seq	#	b: Δ Error	b	y	y: Δ Error	+1
I	1	---	114.091	---	---	8
P	2	393.557	211.144	839.402	247.044	7
S#	3	339.308	378.142	742.349	369.754	6
T	4	---	479.190	575.351	53.141	5
V	5	267.902	578.259	474.303	---	4
L	6	525.684	691.343	375.235	-3.493	3
S	7	---	778.375	262.151	-343.458	2
R	8	---	---	175.119	579.829	1

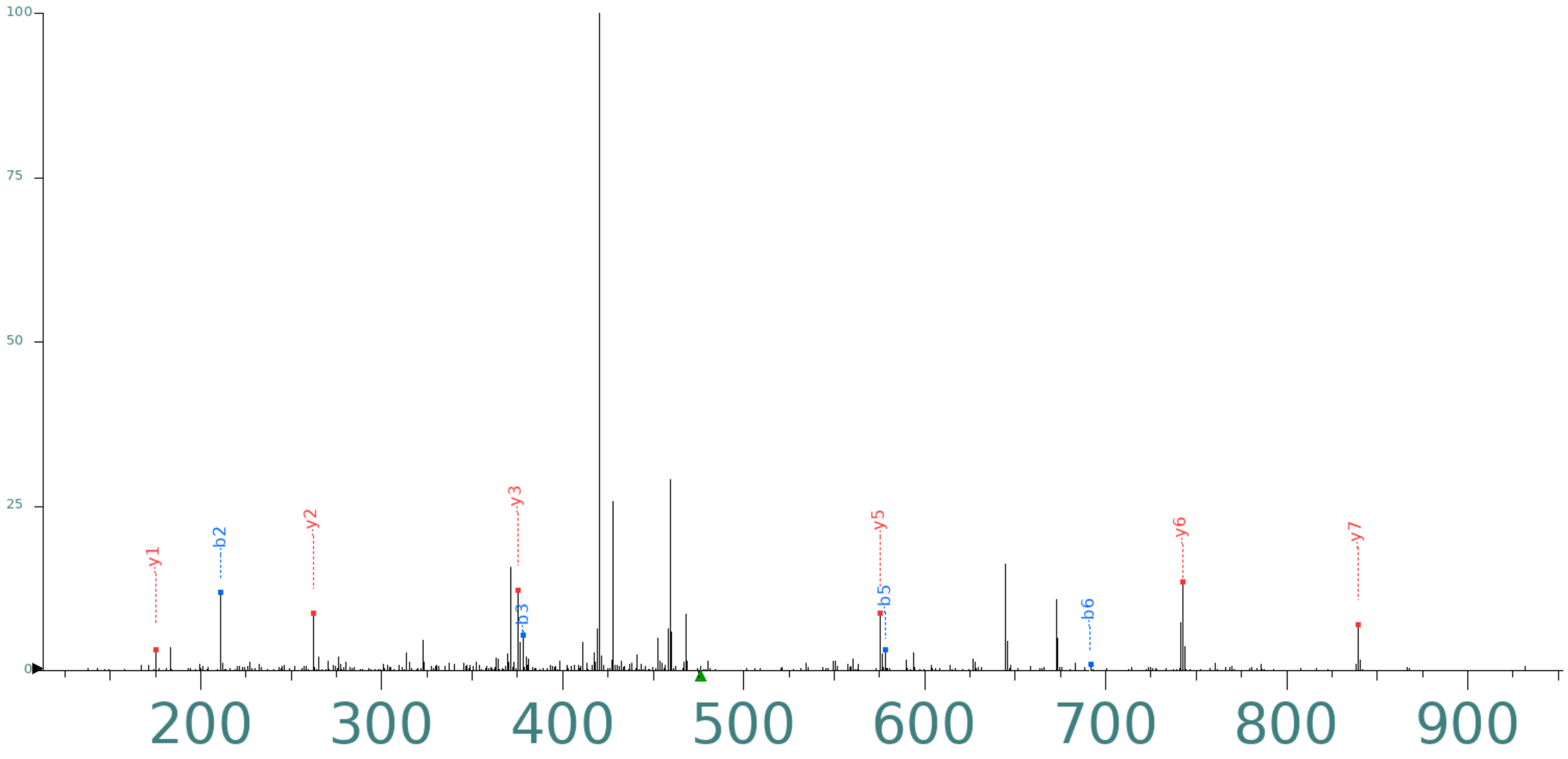

Predicted Fragmentation Pattern

Seq	#	b: Δ Error	b	y	y: Δ Error	+1
I	1	---	114.091	---	---	8
P	2	176.157	211.144	839.402	272.407	7
S#	3	---	378.142	742.349	307.803	6
T	4	---	479.190	575.351	451.707	5
V	5	111.853	578.259	474.303	---	4
L	6	231.212	691.343	375.235	43.513	3
S	7	630.422	778.375	262.151	318.362	2
R	8	---	---	175.119	9.613	1

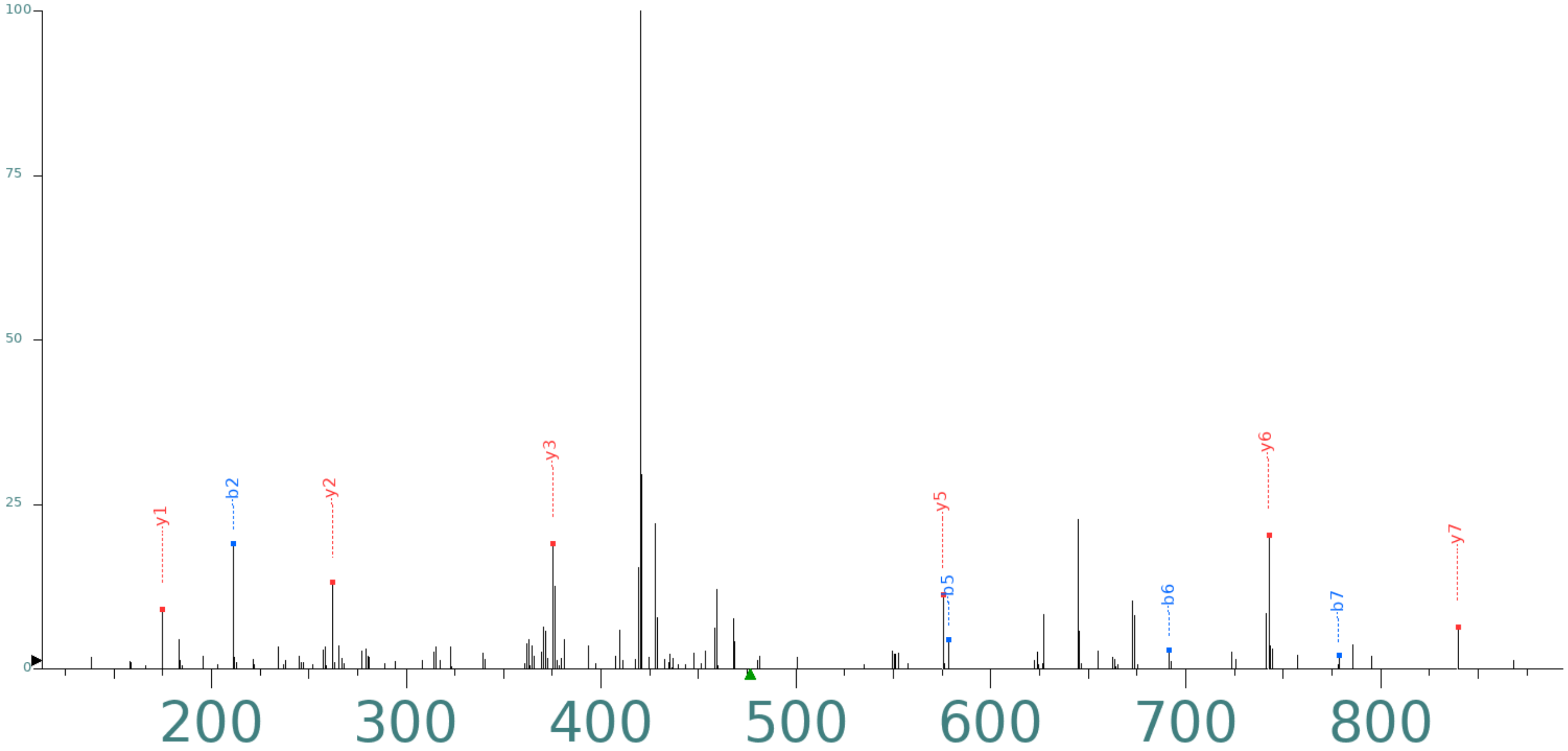

LPA3-LPA

Predicted Fragmentation Pattern

Seq	#	b: Δ Error	b	y	y: Δ Error	+1
S	1	---	88.039	---	---	20
D	2	---	203.066	2151.890	---	19
T#	3	---	384.080	2036.863	---	18
G	4	---	441.102	1855.849	---	17
S	5	---	528.134	1798.828	---	16
Q	6	---	656.192	**1711.796**	165.389	15
Y	7	165.316	**819.256**	**1583.737**	-18.614	14
I	8	---	932.340	**1420.674**	221.145	13
E	9	---	1061.382	**1307.590**	2.516	12
D	10	158.464	**1176.409**	**1178.547**	158.227	11
S	11	218.019	**1263.441**	**1063.520**	1.215	10
I	12	---	1376.525	**976.488**	-12.256	9
S	13	---	1463.557	**863.404**	213.812	8
Q	14	150.847	**1591.616**	**776.372**	220.546	7
G	15	---	1648.637	**648.313**	-71.393	6
A	16	---	1719.675	**591.292**	277.324	5
V	17	210.290	**1818.743**	**520.255**	-269.733	4
C	18	---	1978.774	**421.186**	161.746	3
N	19	---	2092.817	261.156	---	2
K	20	---	---	147.113	---	1

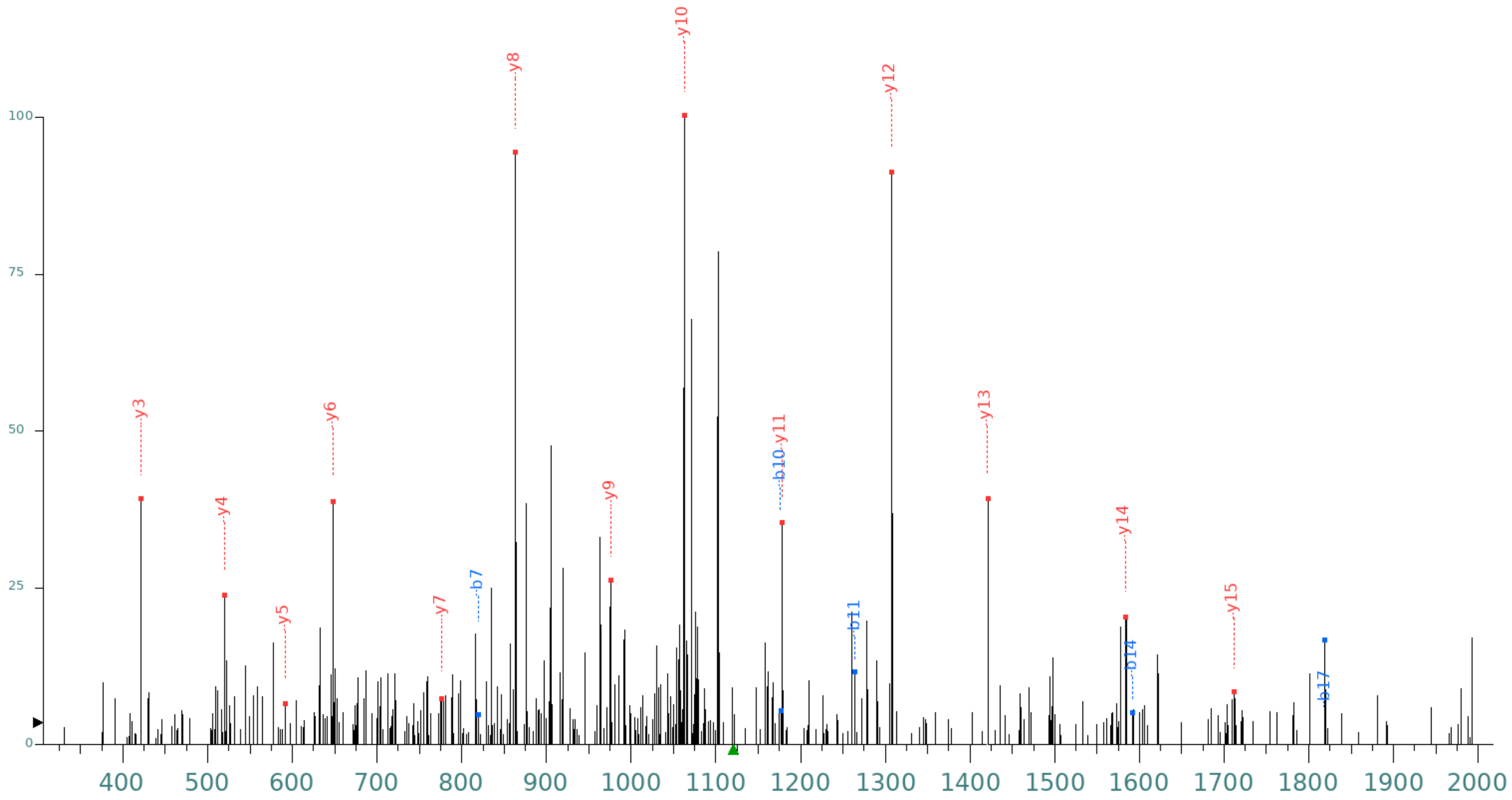

Predicted Fragmentation Pattern

Seq	#	b: Δ Error	b	y	y: Δ Error	+1
S	1	---	88.039	---	---	20
D	2	---	203.066	2151.890	---	19
T#	3	---	384.080	2036.863	---	18
G	4	---	441.102	1855.849	---	17
S	5	---	528.134	1798.828	---	16
Q	6	---	656.192	1711.796	---	15
Y	7	-551.658	**819.256**	**1583.737**	82.197	14
I	8	266.111	**932.340**	**1420.674**	162.396	13
E	9	68.899	**1061.382**	**1307.590**	122.369	12
D	10	227.442	**1176.409**	**1178.547**	68.239	11
S	11	343.454	**1263.441**	**1063.520**	-60.195	10
I	12	138.176	**1376.525**	**976.488**	141.236	9
S	13	167.201	**1463.557**	**863.404**	105.689	8
Q	14	215.401	**1591.616**	**776.372**	336.519	7
G	15	137.796	**1648.637**	**648.313**	54.386	6
A	16	275.504	**1719.675**	**591.292**	50.926	5
V	17	---	1818.743	**520.255**	235.455	4
C	18	---	1978.774	**421.186**	-81.107	3
N	19	---	2092.817	261.156	---	2
K	20	---	---	147.113	---	1

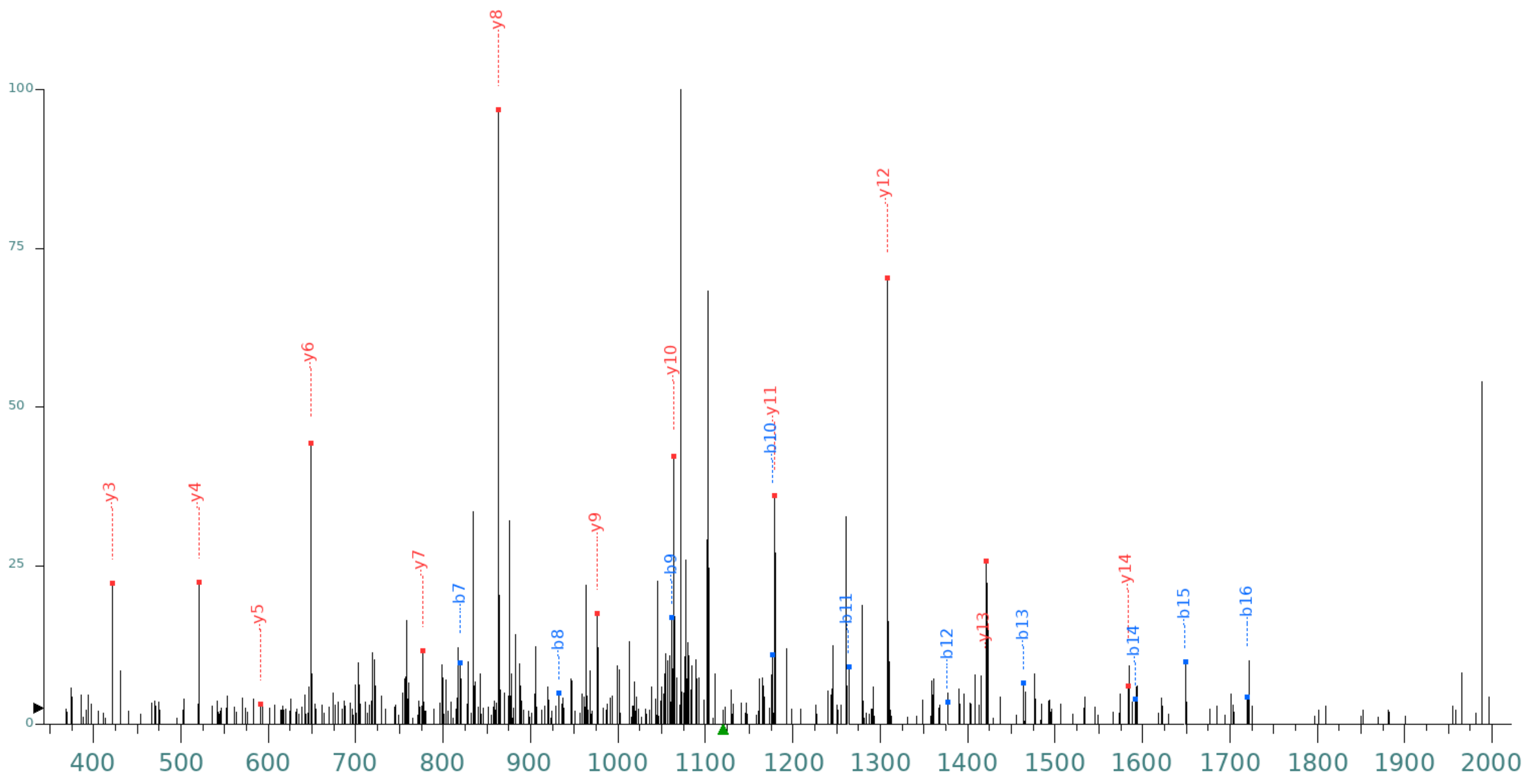

Predicted Fragmentation Pattern

Seq	#	b: Δ Error	b	y	y: Δ Error	+1
I	1	---	114.091	---	---	8
P	2	205.197	211.144	839.402	185.846	7
S#	3	37.428	378.142	742.349	232.531	6
T	4	---	479.190	575.351	22.486	5
V	5	299.971	578.259	474.303	---	4
L	6	187.529	691.343	375.235	-99.146	3
S	7	---	778.375	262.151	286.950	2
R	8	---	---	175.119	275.296	1

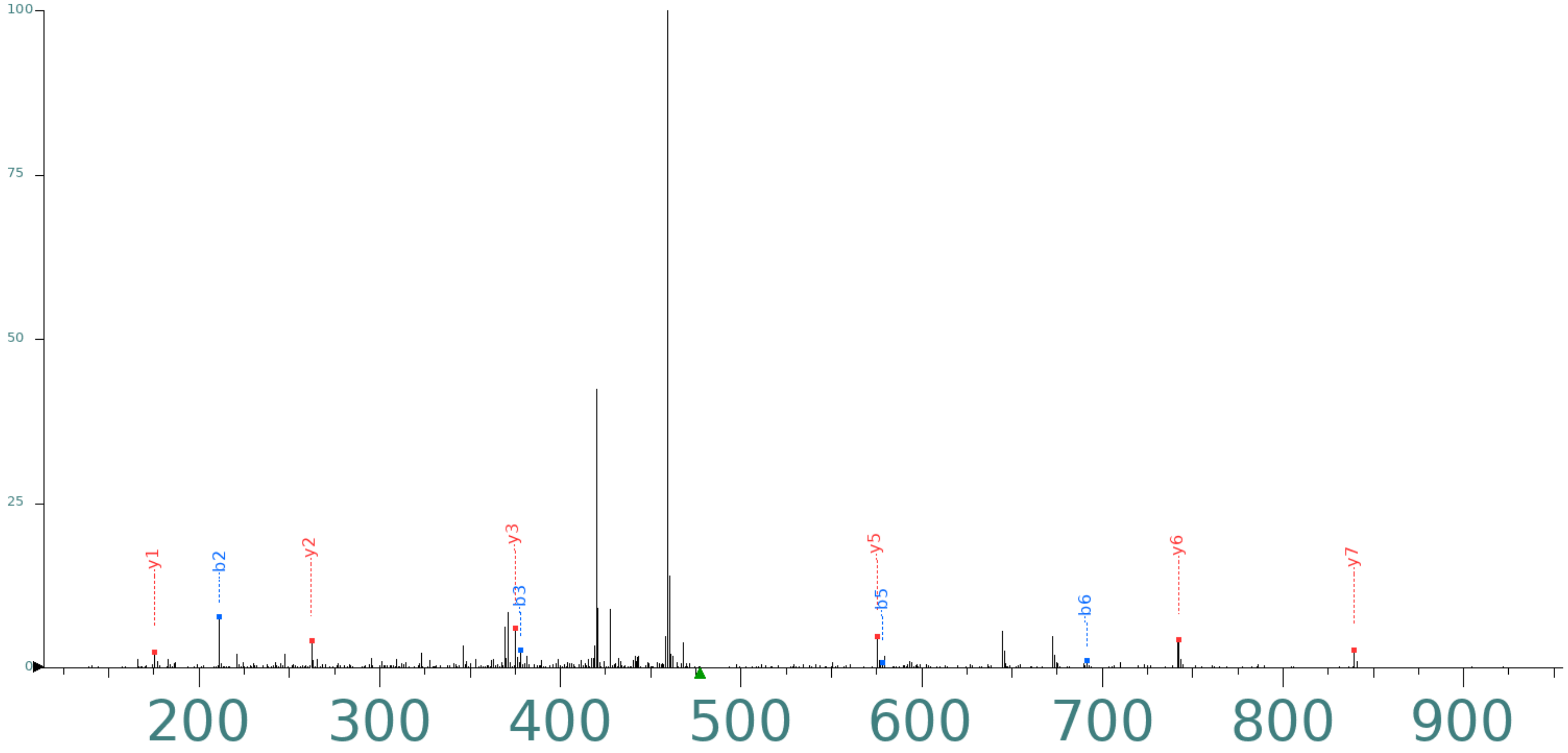

Predicted Fragmentation Pattern

Seq	#	b: Δ Error	b	y	y: Δ Error	+1
I	1	---	114.091	---	---	8
P	2	140.613	211.144	839.402	169.419	7
S#	3	304.063	378.142	742.349	-625.019	6
T	4	---	479.190	575.351	436.021	5
V	5	511.216	578.259	474.303	512.944	4
L	6	220.446	691.343	375.235	371.297	3
S	7	---	778.375	262.151	-60.112	2
R	8	---	---	175.119	312.480	1

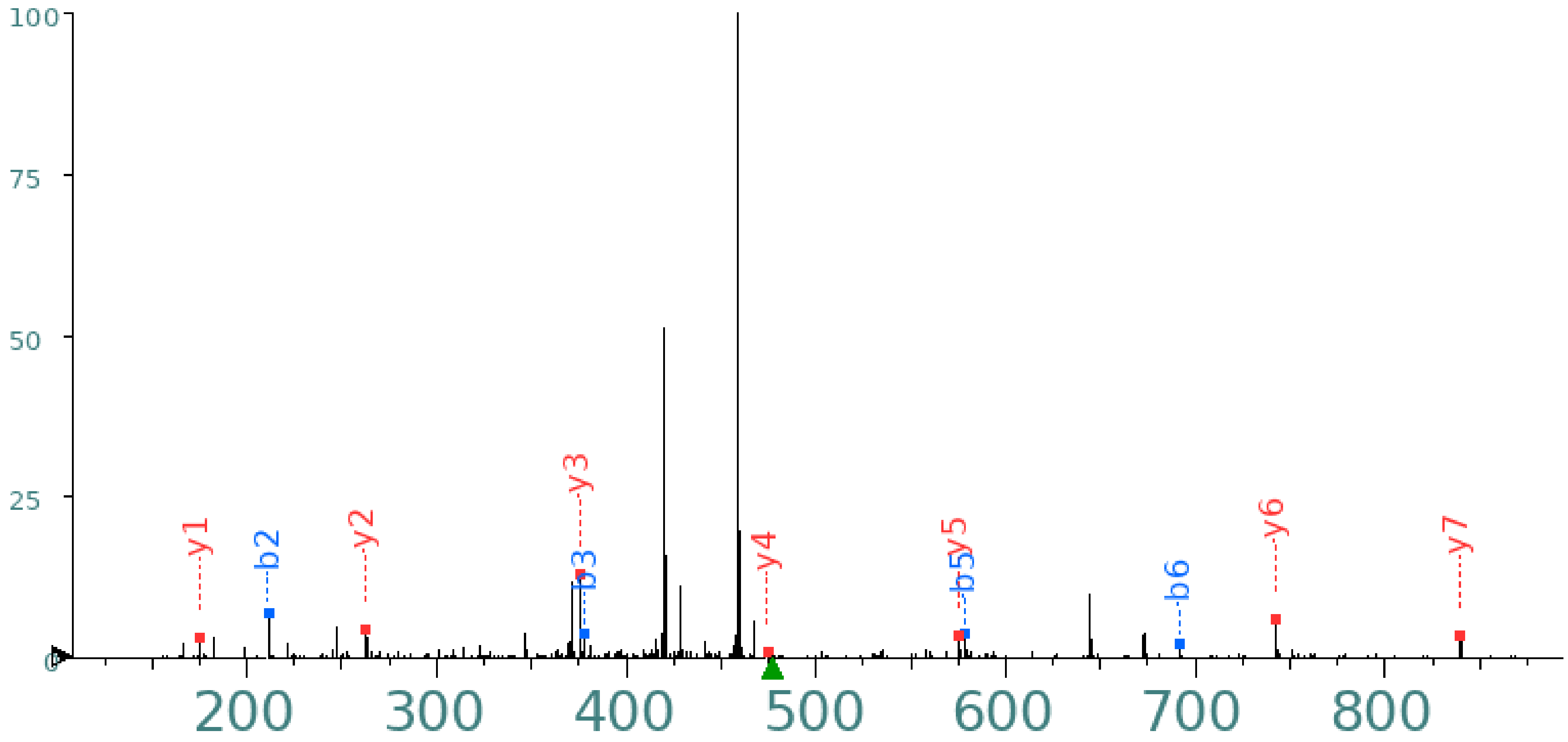

Predicted Fragmentation Pattern

Seq	#	b: Δ Error	b	y	y: Δ Error	+1
I	1	---	114.091	---	---	8
P	2	-711.180	211.144	839.402	35.219	7
S#	3	515.334	378.142	742.349	106.450	6
T	4	---	479.190	575.351	77.749	5
V	5	8.849	578.259	474.303	---	4
L	6	614.926	691.343	375.235	-127.862	3
S	7	145.419	778.375	262.151	-495.153	2
R	8	---	---	175.119	415.833	1

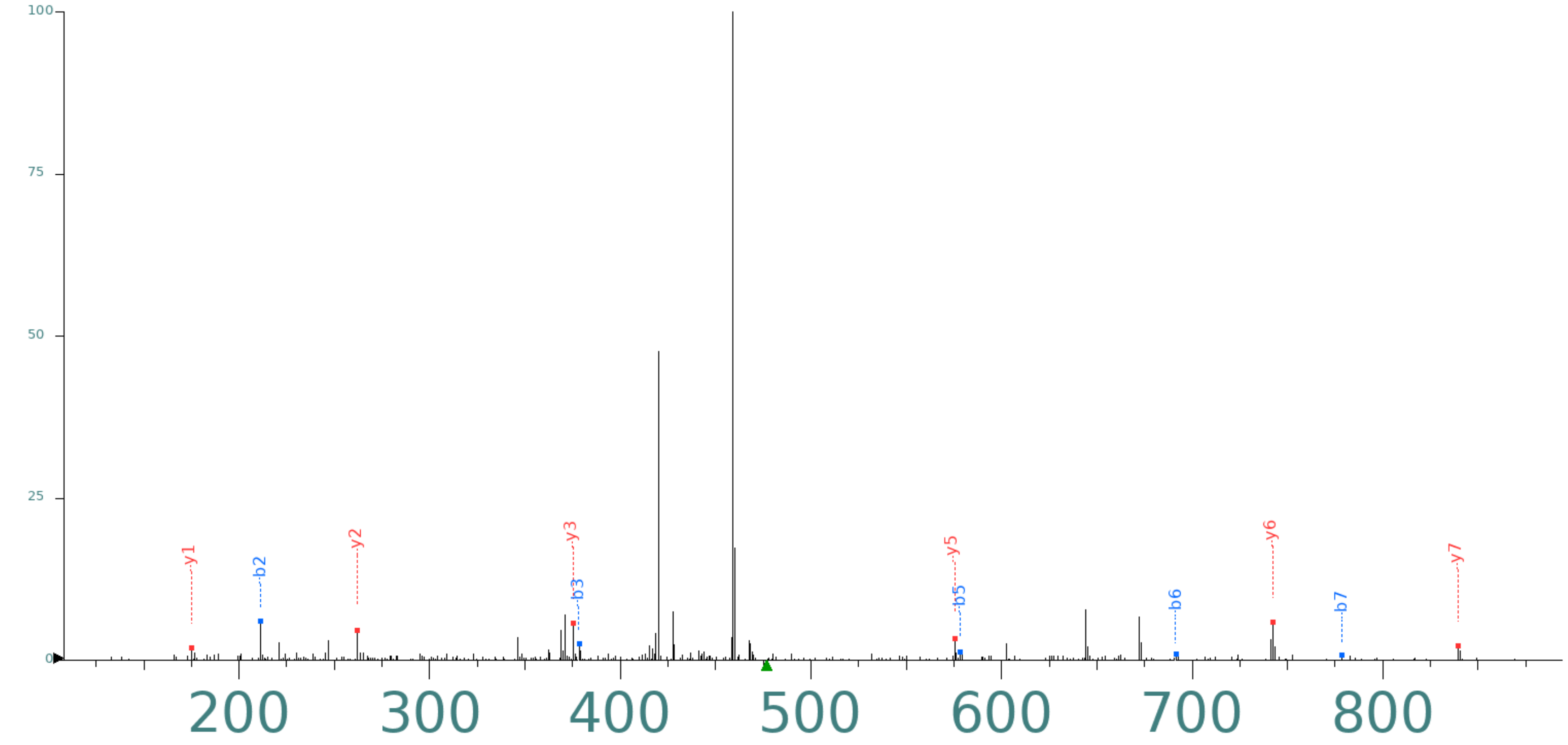

Predicted Fragmentation Pattern

Seq	#	b: Δ Error	b	y	y: Δ Error	+1
I	1	---	114.091	---	---	8
P	2	-366.673	211.144	839.402	167.384	7
S#	3	83.343	378.142	742.349	202.452	6
T	4	---	479.190	575.351	-15.598	5
V	5	124.516	578.259	474.303	---	4
L	6	200.149	691.343	375.235	-460.369	3
S	7	---	778.375	262.151	-520.907	2
R	8	---	---	175.119	100.222	1

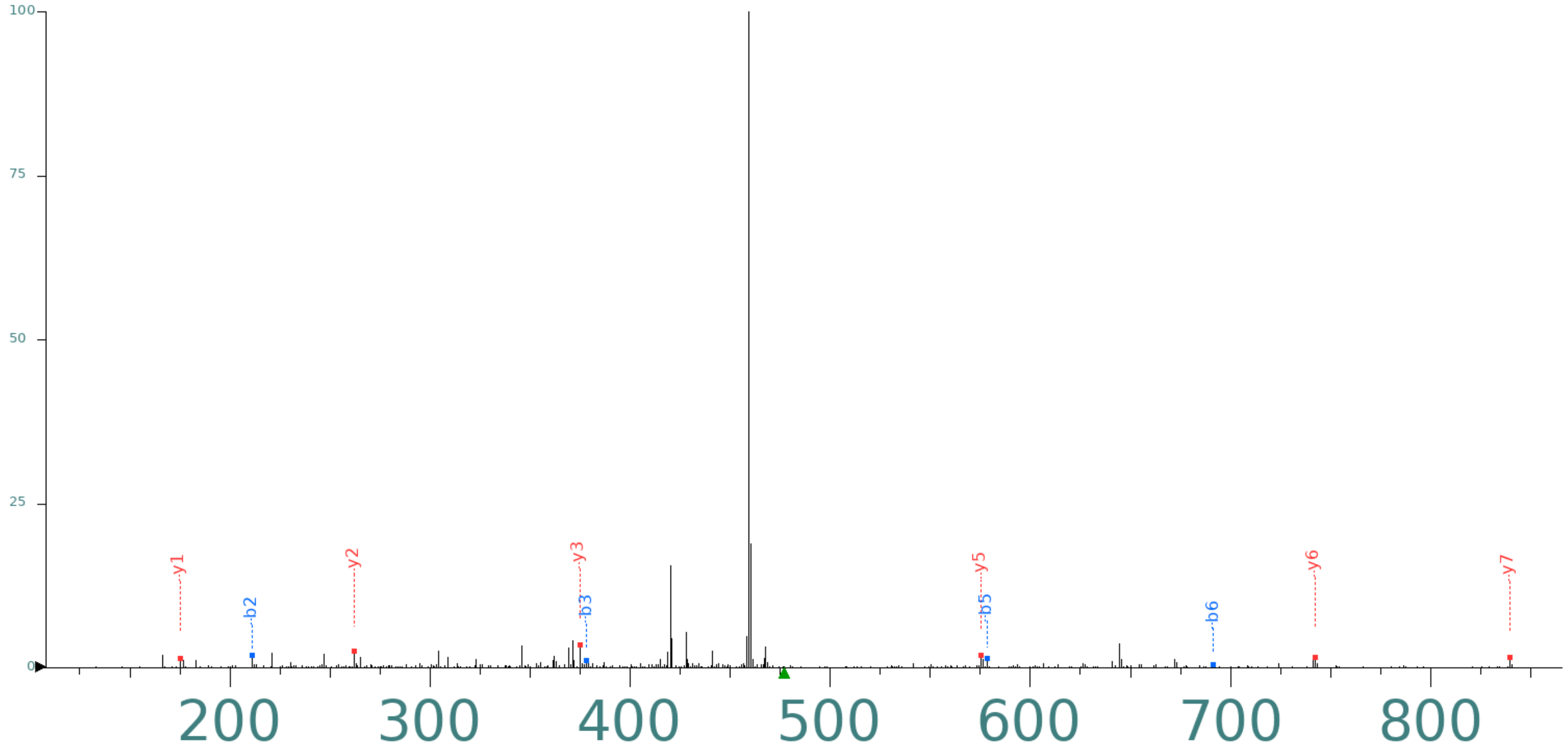

Predicted Fragmentation Pattern

Seq	#	b: Δ Error	b	y	y: Δ Error	+1
I	1	---	114.091	---	---	8
P	2	-803.533	211.144	839.402	24.603	7
S#	3	202.912	378.142	742.349	-66.120	6
T	4	---	479.190	575.351	-5.626	5
V	5	45.367	578.259	474.303	-155.908	4
L	6	-53.368	691.343	375.235	10.577	3
S	7	361.182	778.375	262.151	-346.486	2
R	8	---	---	175.119	-38.138	1

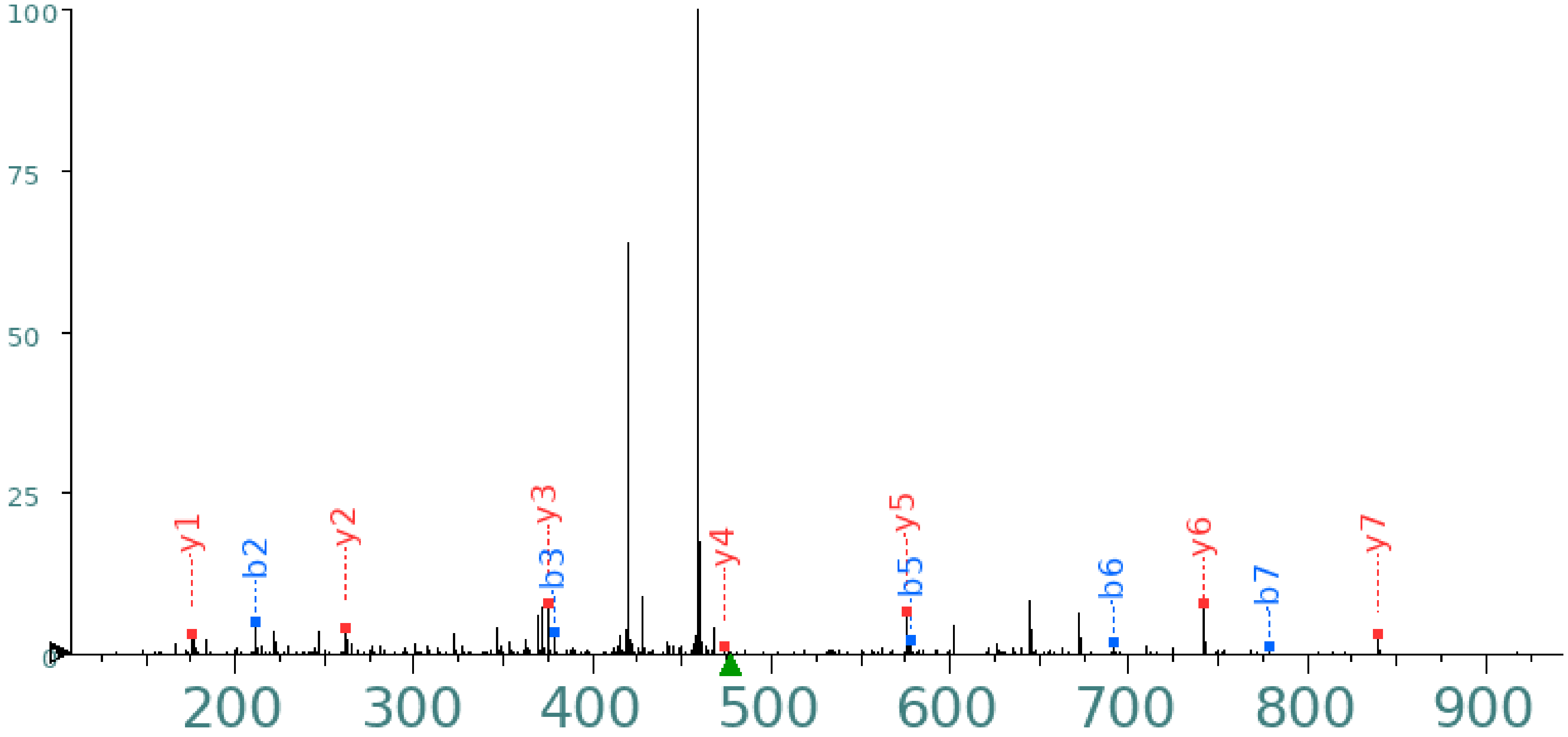

Predicted Fragmentation Pattern

Seq	#	b: Δ Error	b	y	y: Δ Error	+1
I	1	---	114.091	---	---	8
P	2	-830.315	211.144	839.402	157.207	7
S#	3	183.066	378.142	742.349	222.587	6
T	4	---	479.190	575.351	301.076	5
V	5	484.644	578.259	474.303	-151.338	4
L	6	93.179	691.343	375.235	387.875	3
S	7	---	778.375	262.151	-483.151	2
R	8	---	---	175.119	-140.538	1

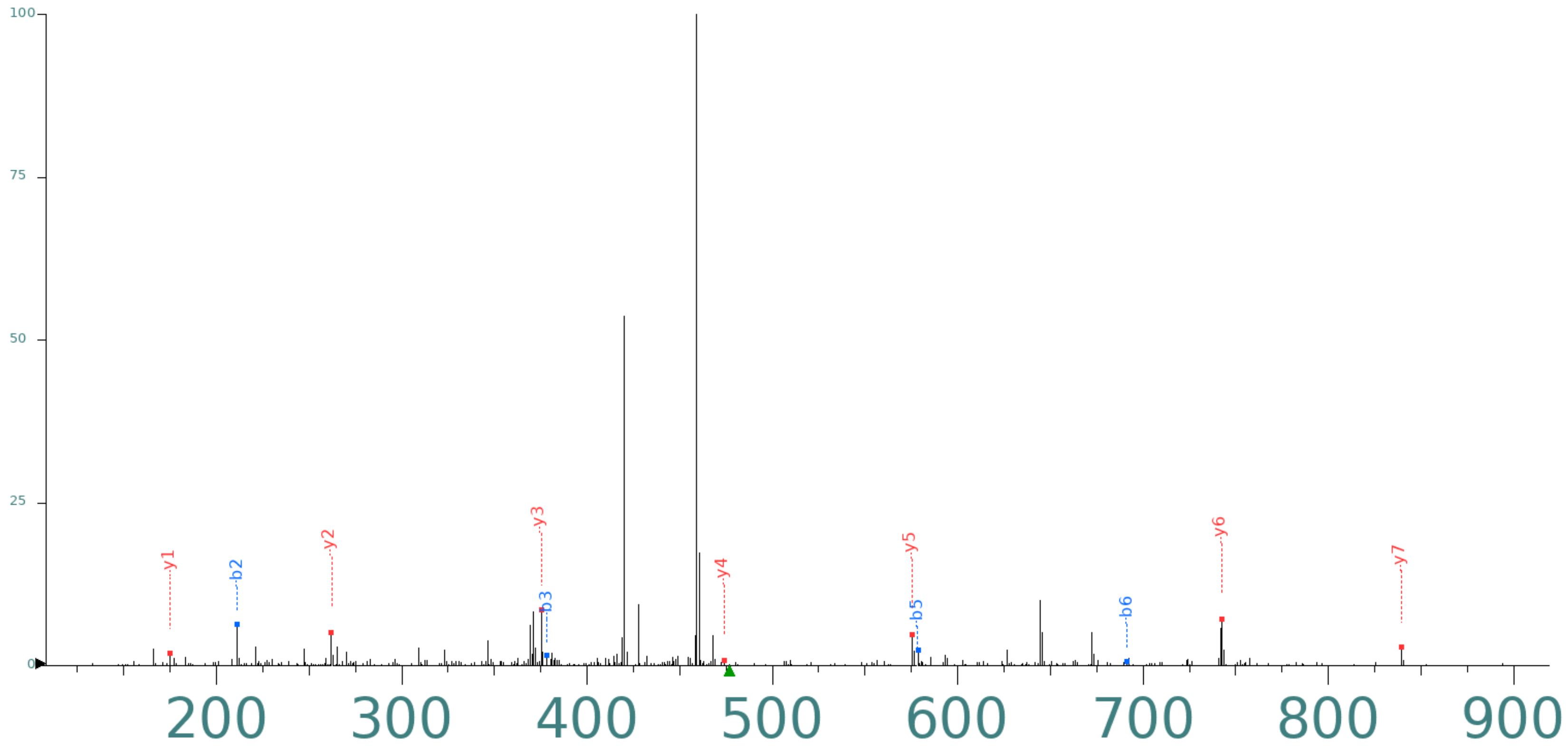

LPA3-PMA

Predicted Fragmentation Pattern

Seq	#	b: Δ Error	b	y	y: Δ Error	+1
I	1	---	114.091	---	---	8
P	2	379.260	211.144	839.402	279.456	7
S#	3	324.872	378.142	742.349	202.206	6
T	4	---	479.190	575.351	-158.942	5
V	5	290.266	578.259	474.303	109.129	4
L	6	43.040	691.343	375.235	-80.437	3
S	7	570.038	778.375	262.151	-203.803	2
R	8	---	---	175.119	885.221	1

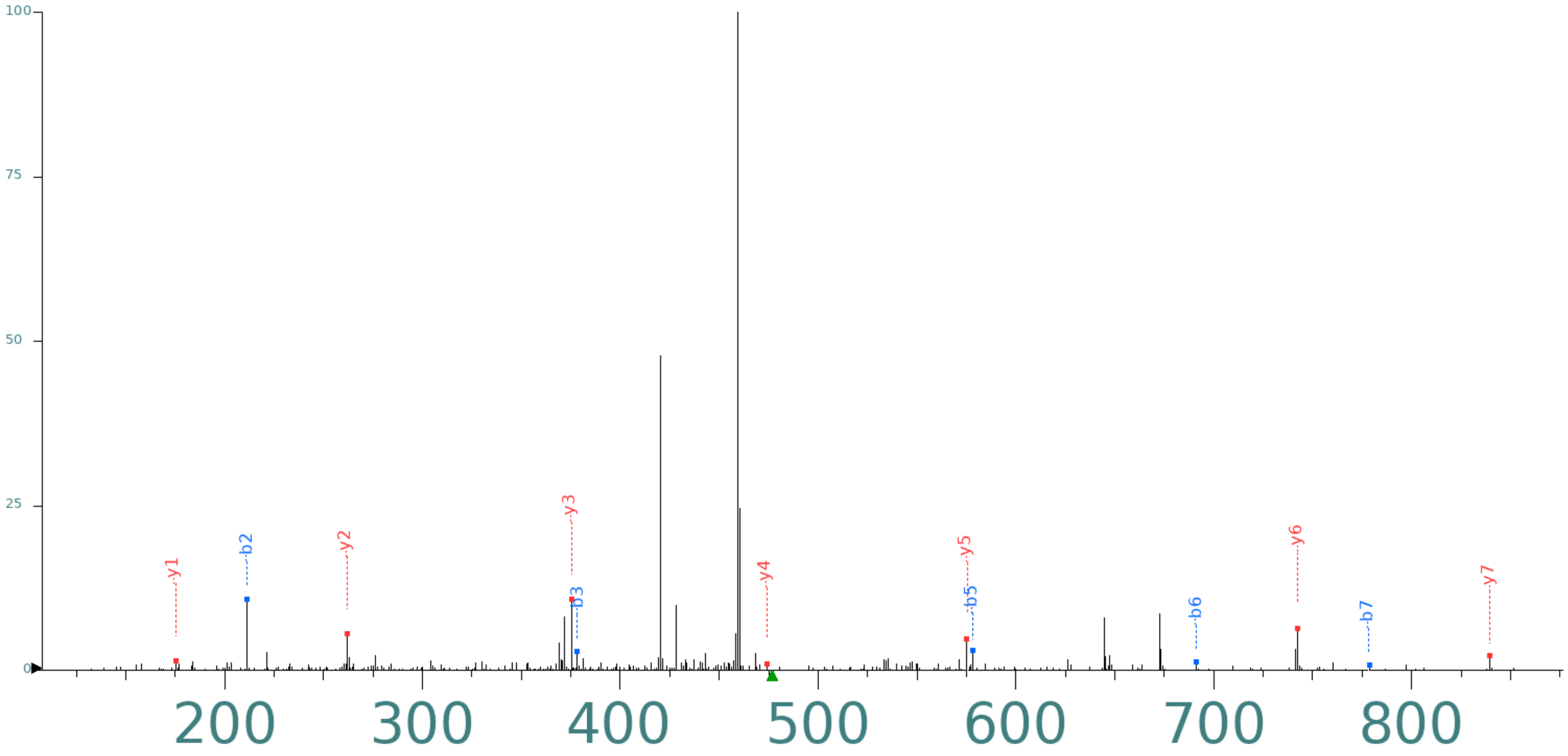

Predicted Fragmentation Pattern

Seq	#	b: Δ Error	b	y	y: Δ Error	+1
I	1	---	114.091	---	---	8
P	2	570.008	211.144	839.402	240.939	7
S#	3	326.485	378.142	742.349	270.990	6
T	4	---	479.190	575.351	354.401	5
V	5	---	578.259	474.303	---	4
L	6	---	691.343	375.235	-61.810	3
S	7	---	778.375	262.151	1129.860	2
R	8	---	---	175.119	243.421	1

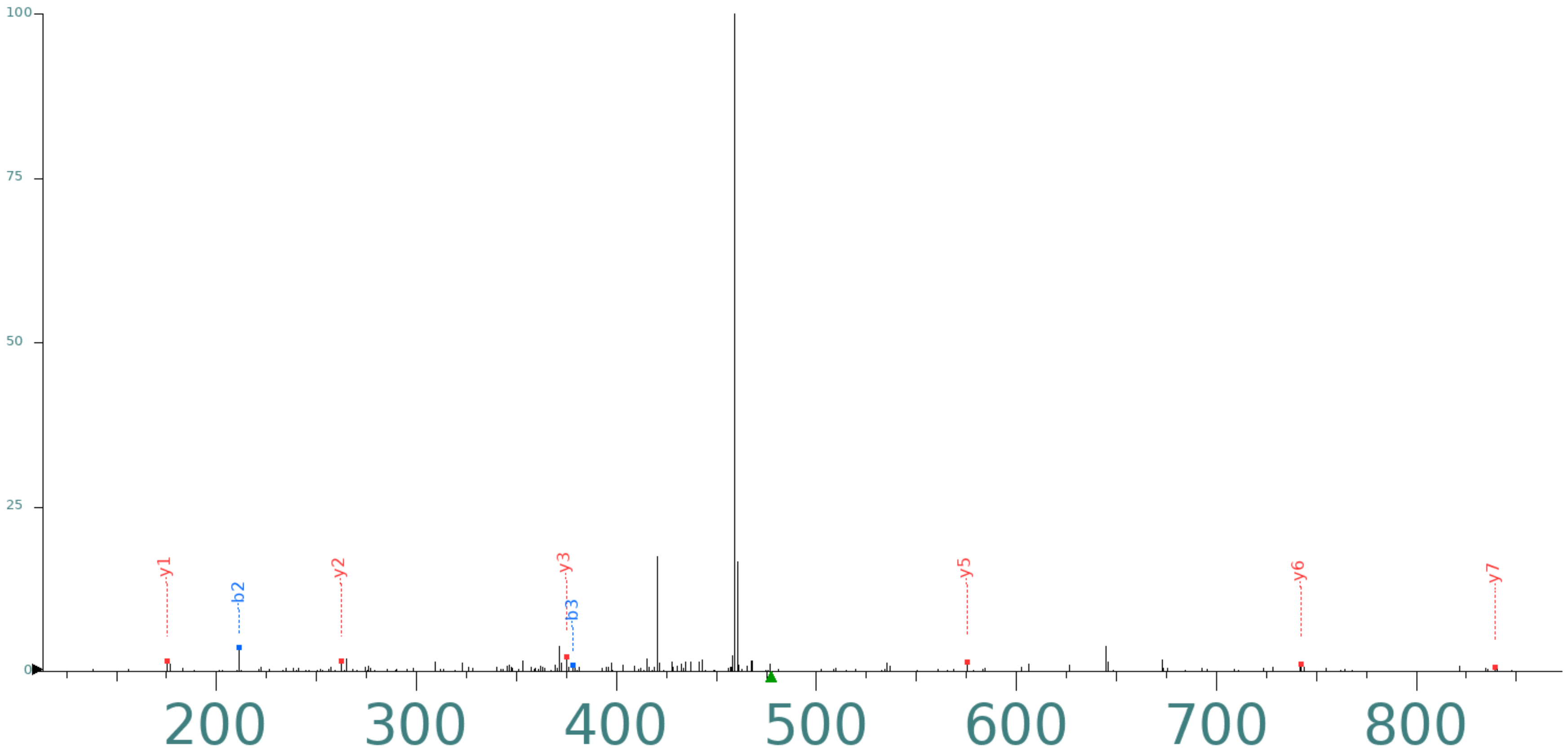

Basal

Predicted Fragmentation Pattern

Seq	#	b: Δ Error	b	y	y: Δ Error	+1
I	1	---	114.091	---	---	8
P	2	-1103.145	211.144	839.402	221.751	7
S#	3	-132.066	378.142	742.349	129.137	6
T	4	---	479.190	575.351	176.488	5
V	5	213.464	578.259	474.303	---	4
L	6	---	691.343	375.235	-148.932	3
S	7	---	778.375	262.151	101.345	2
R	8	---	---	175.119	51.783	1

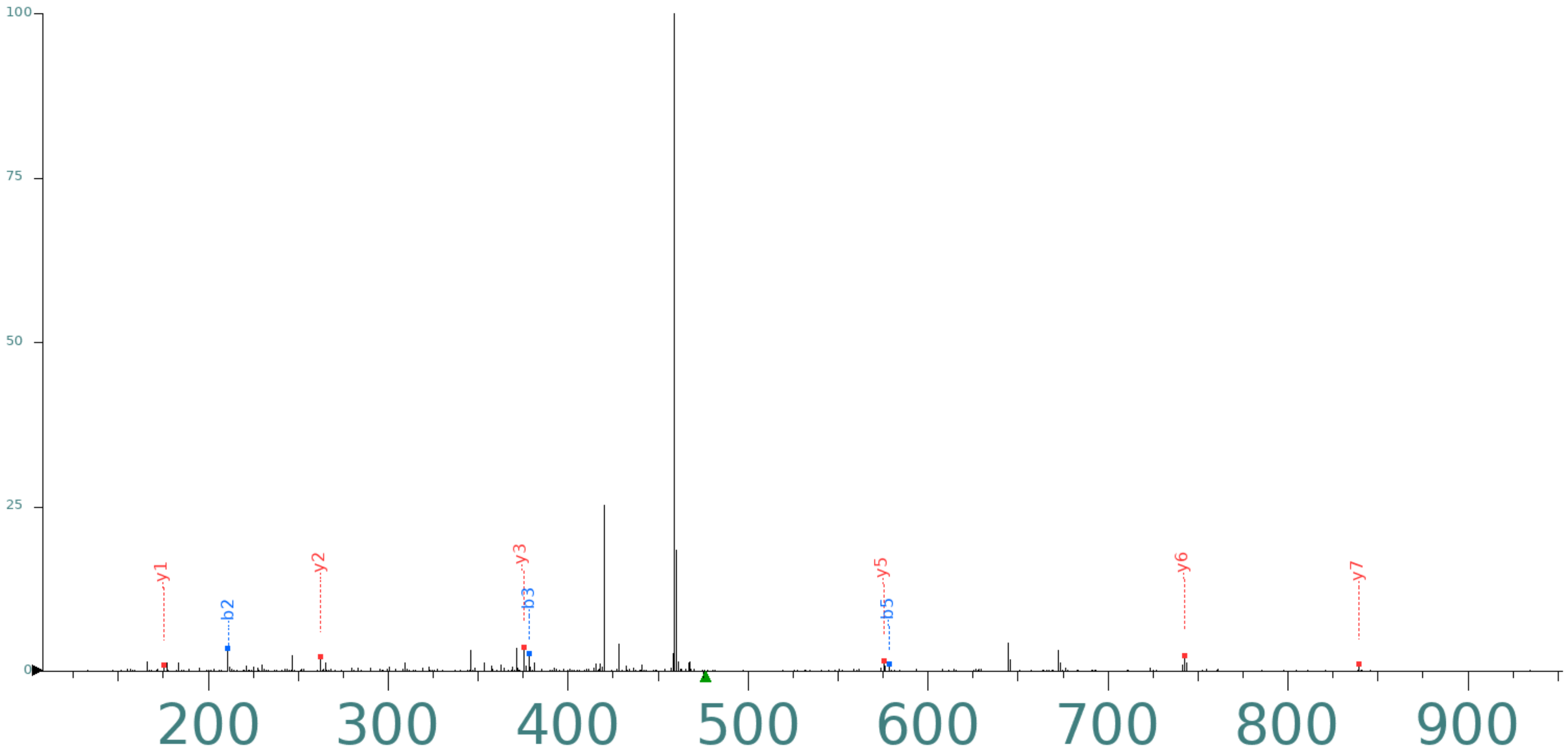

Predicted Fragmentation Pattern

Seq	#	b: Δ Error	b	y	y: Δ Error	+1
I	1	---	114.091	---	---	8
P	2	-766.184	211.144	839.402	171.309	7
S#	3	432.046	378.142	742.349	440.405	6
T	4	---	479.190	575.351	418.744	5
V	5	496.454	578.259	474.303	---	4
L	6	277.096	691.343	375.235	60.184	3
S	7	---	778.375	262.151	-555.285	2
R	8	---	---	175.119	195.780	1

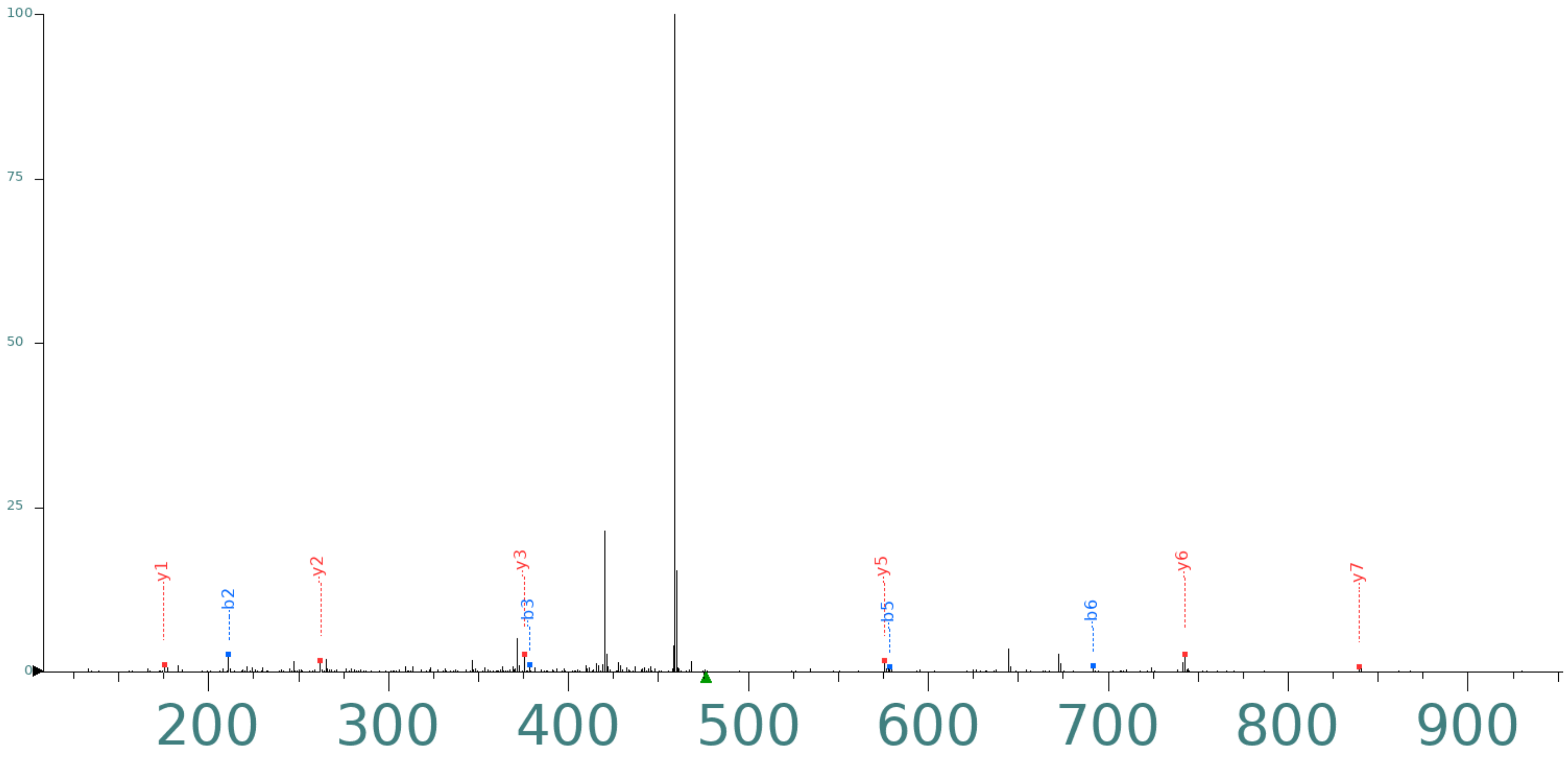

Predicted Fragmentation Pattern

Seq	#	b: Δ Error	b	y	y: Δ Error	+1
I	1	---	114.091	---	---	8
P	2	221.667	211.144	839.402	-28.841	7
S#	3	164.430	378.142	742.349	266.306	6
T	4	---	479.190	575.351	344.224	5
V	5	149.841	578.259	474.303	454.578	4
L	6	287.596	691.343	375.235	435.495	3
S	7	139.696	778.375	262.151	-172.942	2
R	8	---	---	175.119	344.350	1

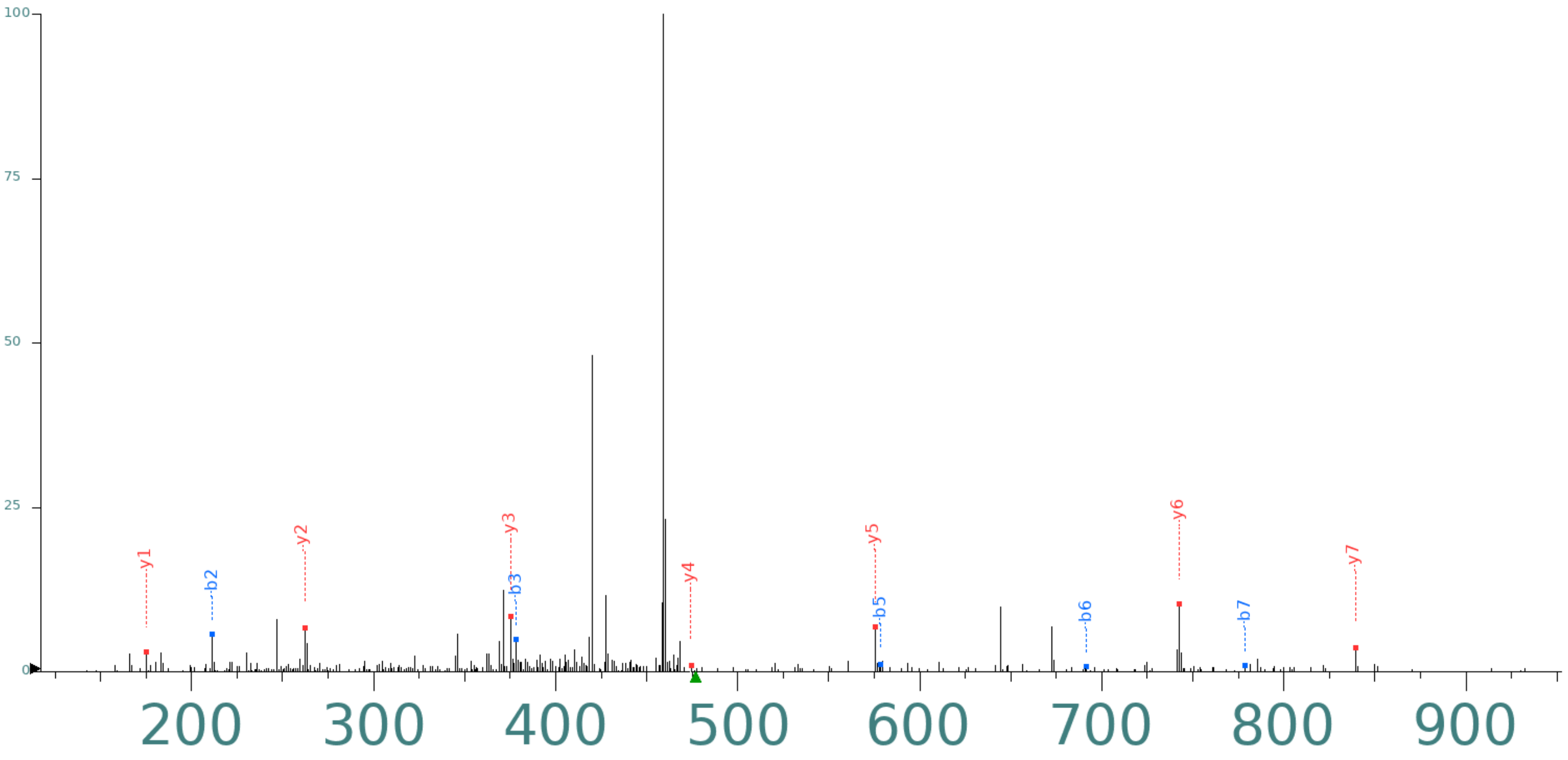

Predicted Fragmentation Pattern

Seq	#	b: Δ Error	b	y	y: Δ Error	+1
I	1	---	114.091	---	---	8
P	2	15.755	211.144	839.402	252.349	7
S#	3	-241.623	378.142	742.349	-93.175	6
T	4	---	479.190	575.351	128.129	5
V	5	172.210	578.259	474.303	45.633	4
L	6	366.560	691.343	375.235	507.485	3
S	7	---	778.375	262.151	380.716	2
R	8	---	---	175.119	328.067	1

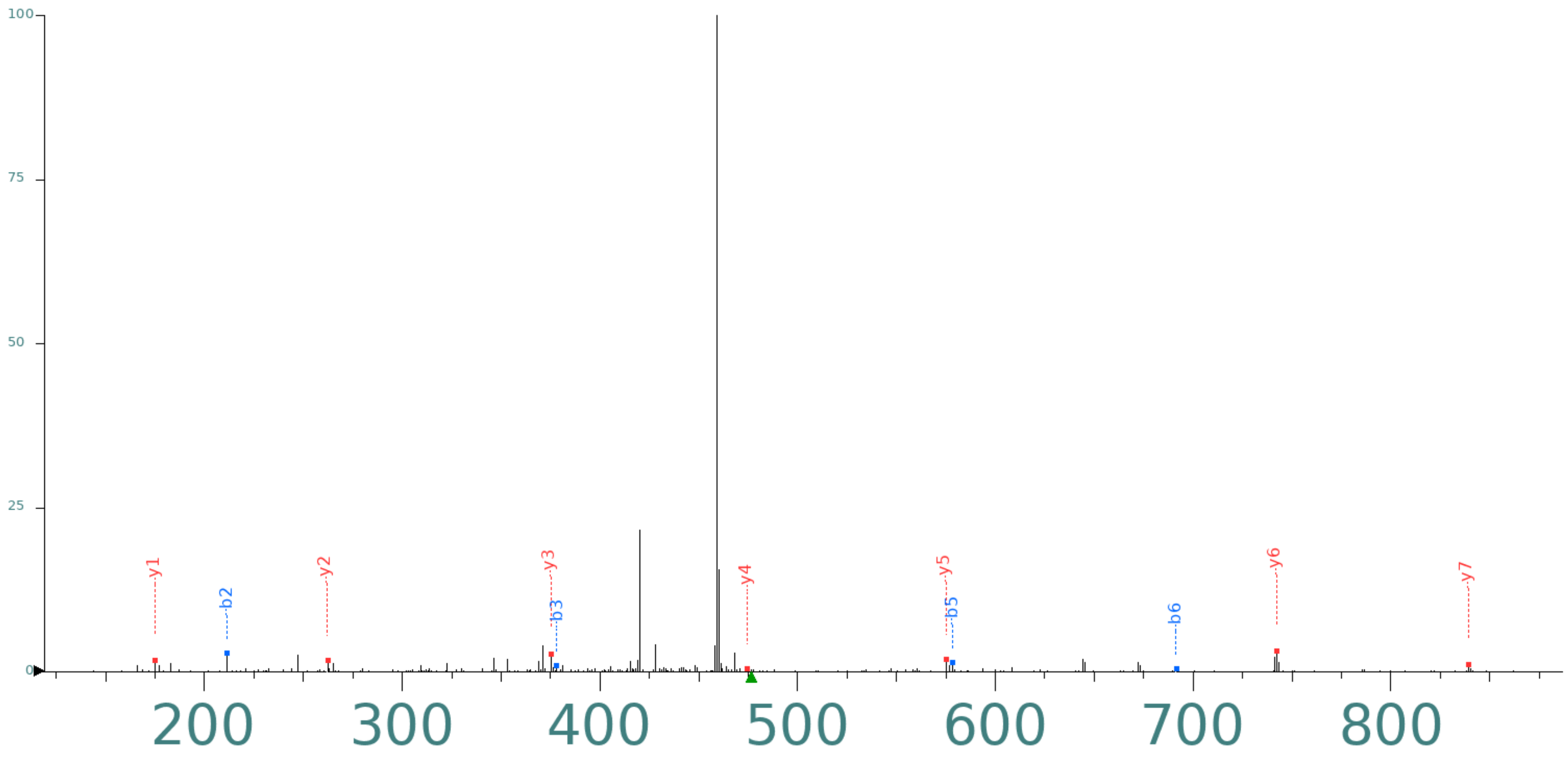

PMA

Predicted Fragmentation Pattern

Seq	#	b: Δ Error	b	y	y: Δ Error	+1
S#	1	---	168.006	---	---	20
D	2	---	283.033	2071.924	---	19
T	3	306.461	**384.080**	1956.897	---	18
G	4	---	441.102	1855.849	---	17
S	5	---	528.134	**1798.828**	209.325	16
Q	6	---	656.192	**1711.796**	44.328	15
Y	7	594.561	**819.256**	**1583.737**	80.887	14
I	8	78.816	**932.340**	**1420.674**	129.067	13
E	9	147.664	**1061.382**	**1307.590**	93.528	12
D	10	74.123	**1176.409**	**1178.547**	57.572	11
S	11	247.763	**1263.441**	**1063.520**	77.652	10
I	12	301.010	**1376.525**	**976.488**	70.121	9
S	13	---	1463.557	**863.404**	86.747	8
Q	14	66.039	**1591.616**	**776.372**	230.605	7
G	15	248.226	**1648.637**	**648.313**	25.204	6
A	16	150.624	**1719.675**	**591.292**	555.588	5
V	17	109.310	**1818.743**	**520.255**	-42.887	4
C	18	96.950	**1978.774**	**421.186**	-161.408	3
N	19	---	2092.817	261.156	---	2
K	20	---	---	147.113	---	1

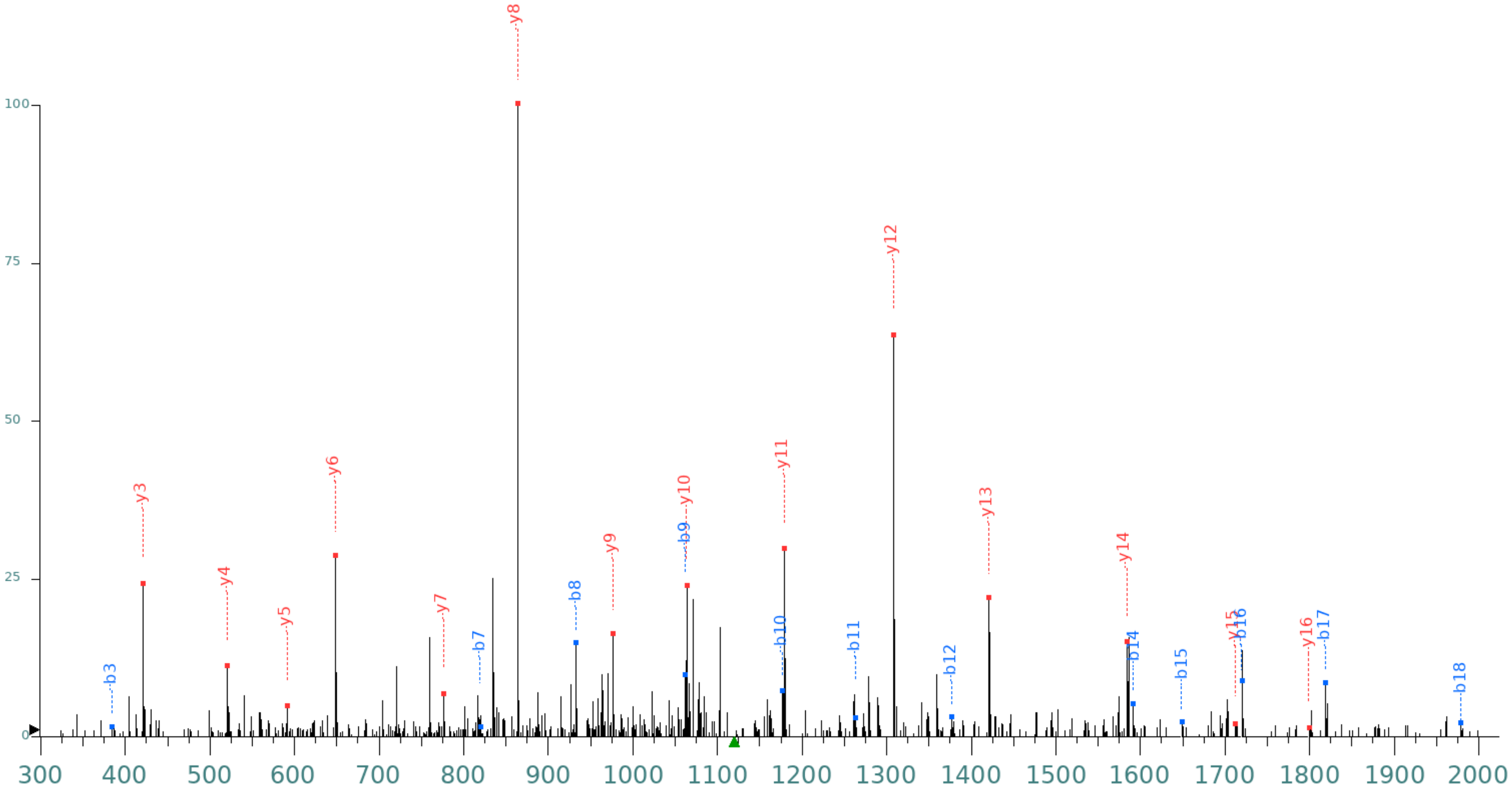

Predicted Fragmentation Pattern

Seq	#	b: Δ Error	b	y	y: Δ Error	+1
S	1	---	88.039	---	---	20
D	2	---	203.066	2151.890	---	19
T	3	---	304.114	2036.863	---	18
G	4	---	361.135	**1935.815**	212.290	17
S	5	---	448.167	**1878.794**	-50.676	16
Q	6	173.925	**576.226**	1791.762	---	15
Y	7	34.815	**739.289**	**1663.703**	5.454	14
I	8	69.626	**852.373**	**1500.640**	205.365	13
E	9	239.040	**981.416**	**1387.556**	169.215	12
D	10	164.030	**1096.443**	**1258.513**	175.501	11
S	11	---	1183.475	**1143.486**	173.167	10
I	12	260.494	**1296.559**	1056.454	---	9
S#	13	---	1463.557	**943.370**	73.937	8
Q	14	---	1591.616	**776.372**	275.550	7
G	15	---	1648.637	**648.313**	299.640	6
A	16	---	1719.675	**591.292**	132.457	5
V	17	95.084	**1818.743**	**520.255**	167.203	4
C	18	-92.128	**1978.774**	**421.186**	-175.687	3
N	19	---	2092.817	261.156	---	2
K	20	---	---	147.113	---	1

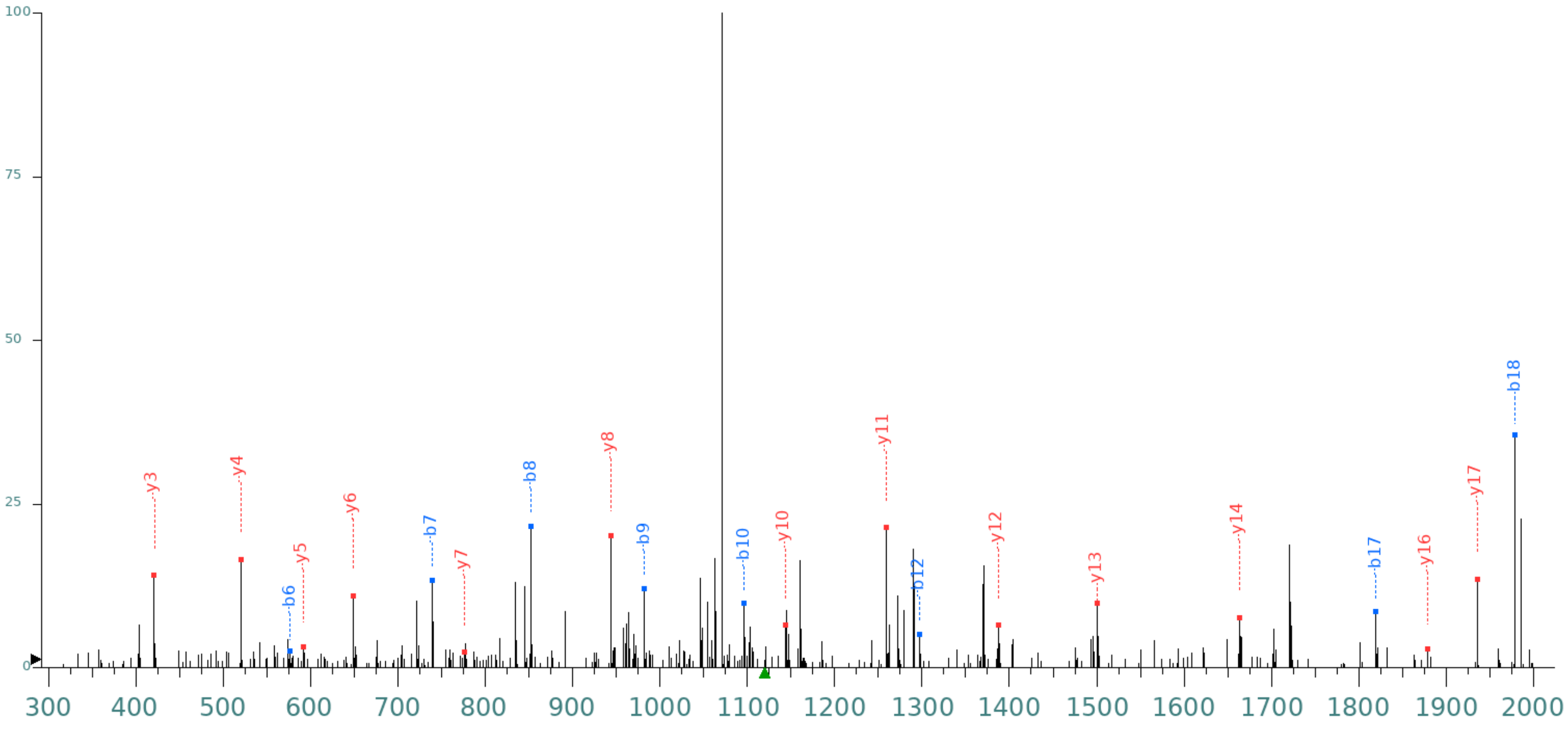

Predicted Fragmentation Pattern

Seq	#	b: Δ Error	b	y	y: Δ Error	+1
S	1	---	88.039	---	---	20
D	2	---	203.066	2151.890	---	19
T#	3	---	384.080	2036.863	---	18
G	4	---	441.102	1855.849	---	17
S	5	---	528.134	1798.828	---	16
Q	6	---	656.192	1711.796	---	15
Y	7	216.107	819.256	1583.737	-33.799	14
I	8	75.806	932.340	1420.674	-30.479	13
E	9	144.904	1061.382	1307.590	46.204	12
D	10	-4.837	1176.409	1178.547	248.199	11
S	11	-79.811	1263.441	1063.520	96.932	10
I	12	249.160	1376.525	976.488	191.598	9
S	13	139.268	1463.557	863.404	46.176	8
Q	14	42.266	1591.616	776.372	-22.014	7
G	15	---	1648.637	648.313	40.266	6
A	16	---	1719.675	591.292	336.744	5
V	17	27.974	1818.743	520.255	345.435	4
C	18	---	1978.774	421.186	372.409	3
N	19	---	2092.817	261.156	---	2
K	20	---	---	147.113	---	1

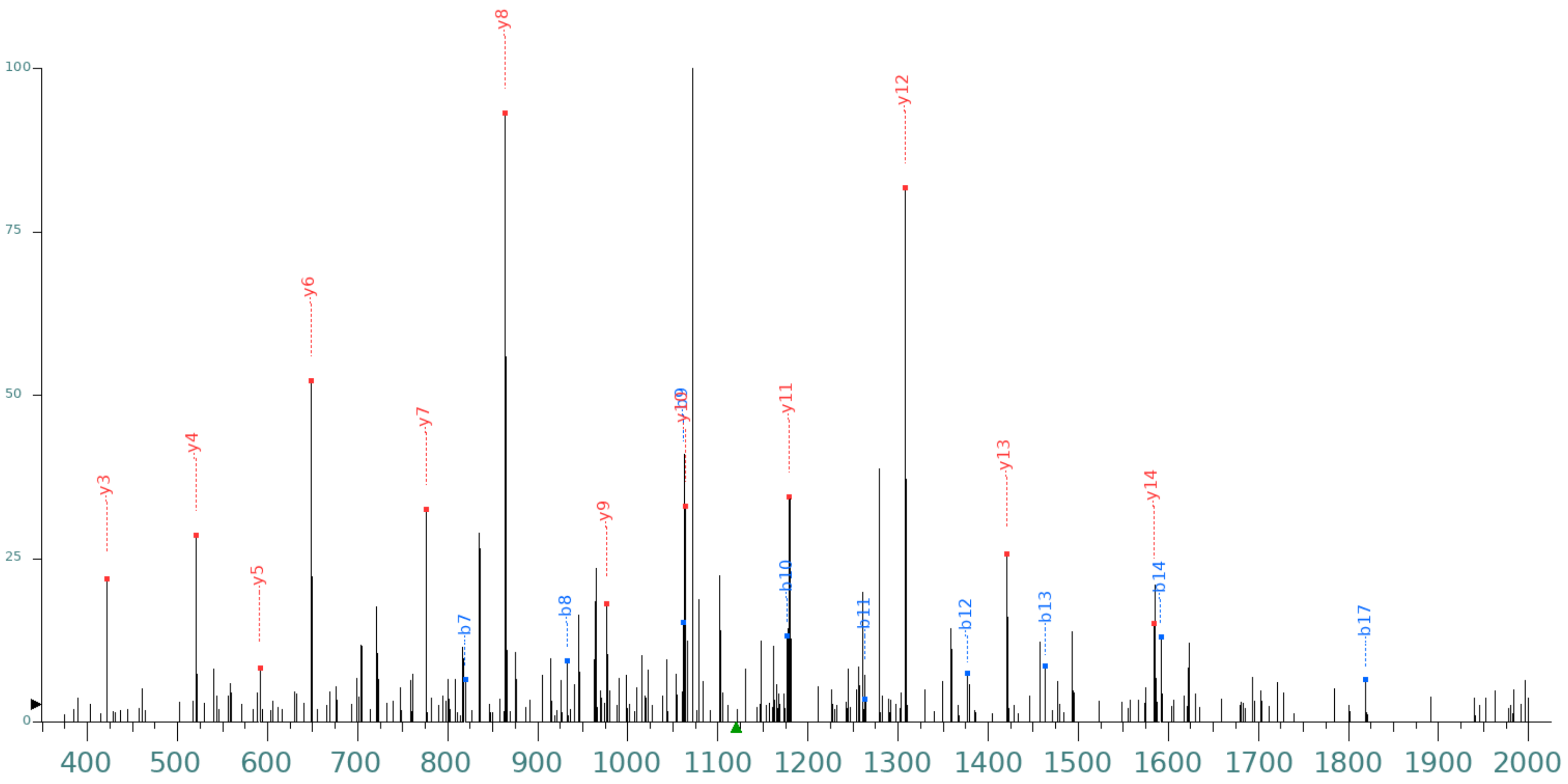

Predicted Fragmentation Pattern

Seq	#	b: Δ Error	b	y	y: Δ Error	+1
S	1	---	88.039	---	---	20
D	2	---	203.066	2151.890	---	19
T	3	---	304.114	2036.863	---	18
G	4	2.497	**361.135**	1935.815	---	17
S	5	---	448.167	1878.794	---	16
Q	6	---	576.226	1791.762	---	15
Y	7	18.634	**739.289**	**1663.703**	112.638	14
I	8	14.350	**852.373**	**1500.640**	146.573	13
E	9	-94.877	**981.416**	**1387.556**	234.554	12
D	10	231.582	**1096.443**	**1258.513**	195.281	11
S	11	---	1183.475	**1143.486**	-72.554	10
I	12	52.301	**1296.559**	1056.454	---	9
S#	13	---	1463.557	**943.370**	207.827	8
Q	14	130.988	**1591.616**	**776.372**	1.100	7
G	15	---	1648.637	**648.313**	573.361	6
A	16	0.940	**1719.675**	**591.292**	510.837	5
V	17	124.139	**1818.743**	**520.255**	433.941	4
C	18	---	1978.774	**421.186**	-223.527	3
N	19	---	2092.817	261.156	---	2
K	20	---	---	147.113	---	1

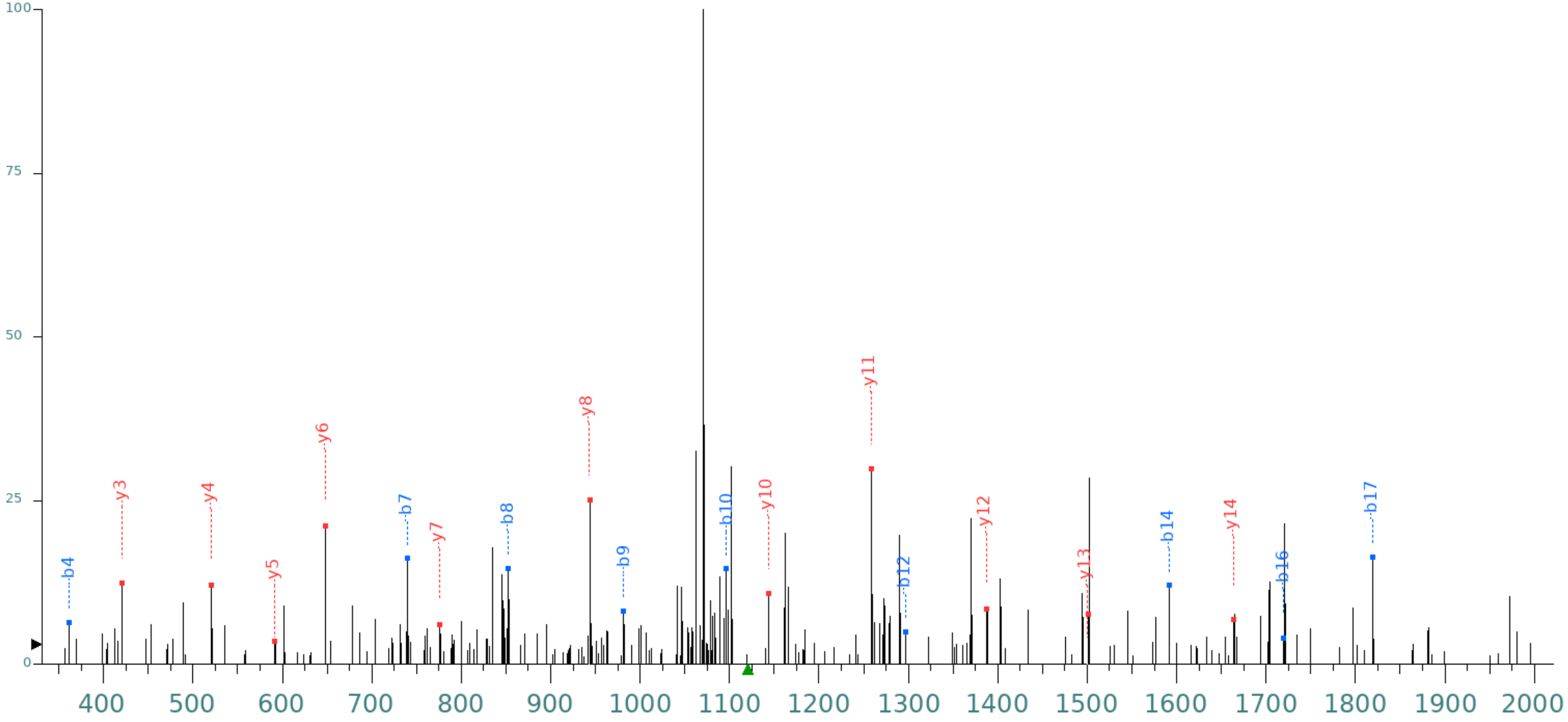

Predicted Fragmentation Pattern

Seq	#	b: Δ Error	b	y	y: Δ Error	+1
T	1	---	102.055	---	---	14
N	2	---	216.098	1434.674	---	13
V	3	216.157	315.166	1320.631	264.645	12
L	4	617.538	428.250	1221.562	261.576	11
S	5	330.053	515.282	1108.478	138.731	10
P	6	-135.092	612.335	1021.446	67.496	9
H	7	187.833	749.394	924.393	225.638	8
T	8	78.831	850.442	787.335	282.046	7
S	9	300.097	937.474	686.287	518.002	6
G	10	121.202	994.495	599.255	199.310	5
S#	11	---	1161.494	542.233	---	4
I	12	220.022	1274.578	375.235	209.952	3
S	13	240.249	1361.610	262.151	796.400	2
R	14	---	---	175.119	---	1

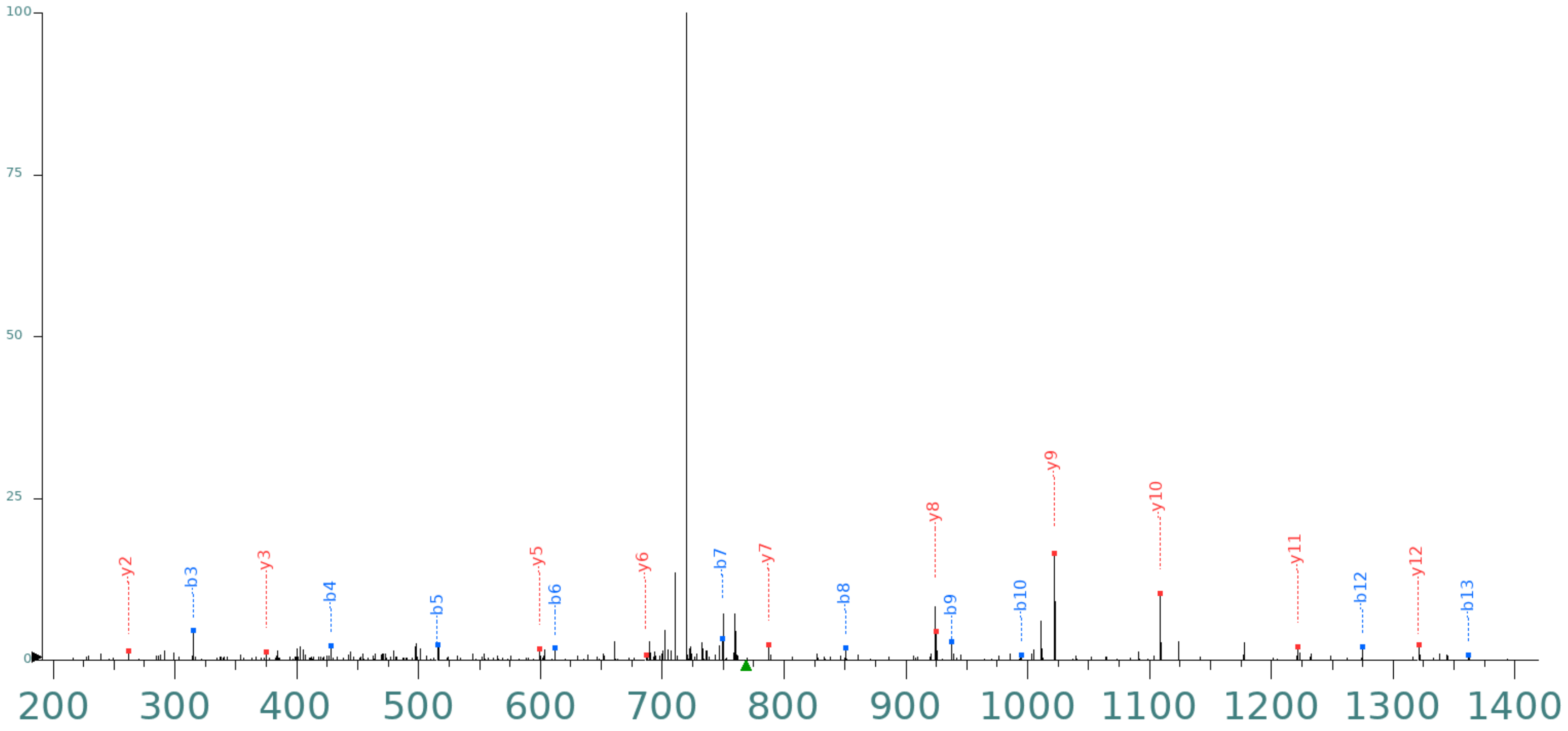

Predicted Fragmentation Pattern

+1

Seq	#	b: Δ Error	b	y	y: Δ Error	+1
S#	1	---	168.006	---	---	20
D	2	---	283.033	2071.924	---	19
T	3	219.423	384.080	1956.897	---	18
G	4	---	441.102	1855.849	---	17
S	5	---	528.134	1798.828	---	16
Q	6	328.783	656.192	1711.796	---	15
Y	7	209.330	819.256	1583.737	---	14
I	8	323.555	932.340	1420.674	---	13
E	9	198.136	1061.382	1307.590	---	12
D	10	---	1176.409	1178.547	8.894	11
S	11	308.019	1263.441	1063.520	56.995	10
I	12	180.463	1376.525	976.488	---	9
S	13	---	1463.557	863.404	157.916	8
Q	14	---	1591.616	776.372	540.112	7
G	15	---	1648.637	648.313	-11.700	6
A	16	-0.053	1719.675	591.292	-121.263	5
V	17	---	1818.743	520.255	23.164	4
C	18	---	1978.774	421.186	-162.785	3
N	19	---	2092.817	261.156	-372.561	2
K	20	---	---	147.113	---	1

+2

Seq	#	b: Δ Error	b	y	y: Δ Error	+1
S#	1	---	84.506	---	---	20
D	2	---	142.020	1036.465	---	19
T	3	---	192.544	978.952	---	18
G	4	---	221.054	928.428	-308.165	17
S	5	---	264.571	899.917	270.247	16
Q	6	-1020.238	328.600	856.401	---	15
Y	7	393.630	410.131	792.372	173.738	14
I	8	---	466.673	710.840	579.987	13
E	9	396.770	531.195	654.298	-74.974	12
D	10	---	588.708	589.777	753.167	11
S	11	-312.463	632.224	532.264	715.834	10
I	12	-370.127	688.766	488.748	-890.648	9
S	13	412.356	732.282	432.206	70.606	8
Q	14	303.087	796.312	388.690	---	7
G	15	19.437	824.822	324.660	---	6
A	16	368.033	860.341	296.150	---	5
V	17	-134.972	909.875	260.631	1637.321	4
C	18	---	989.890	211.097	---	3
N	19	---	1046.912	131.082	---	2
K	20	---	---	74.060	---	1

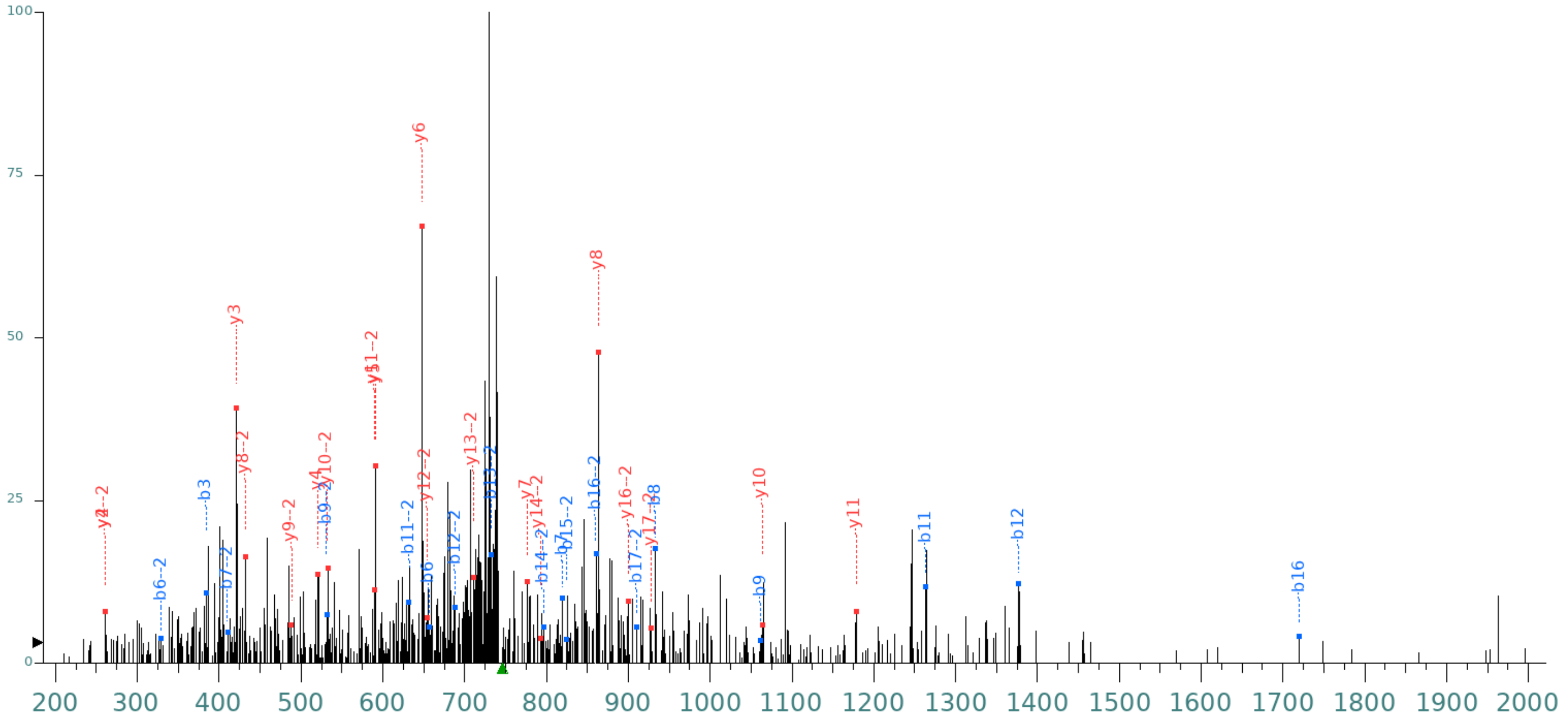

Predicted Fragmentation Pattern

Seq	#	b: Δ Error	b	y	y: Δ Error	+1
T	1	---	102.055	---	---	14
N	2	416.585	**216.098**	1434.674	---	13
V	3	-94.436	**315.166**	1320.631	---	12
L	4	365.664	**428.250**	**1221.562**	6.824	11
S	5	152.937	**515.282**	**1108.478**	92.820	10
P	6	118.687	**612.335**	**1021.446**	101.012	9
H	7	33.445	**749.394**	**924.393**	372.725	8
T	8	325.186	**850.442**	**787.335**	152.022	7
S	9	---	937.474	**686.287**	251.935	6
G	10	342.655	**994.495**	599.255	---	5
S#	11	---	1161.494	**542.233**	510.875	4
I	12	---	1274.578	**375.235**	2.769	3
S	13	---	1361.610	**262.151**	569.241	2
R	14	---	---	175.119	---	1

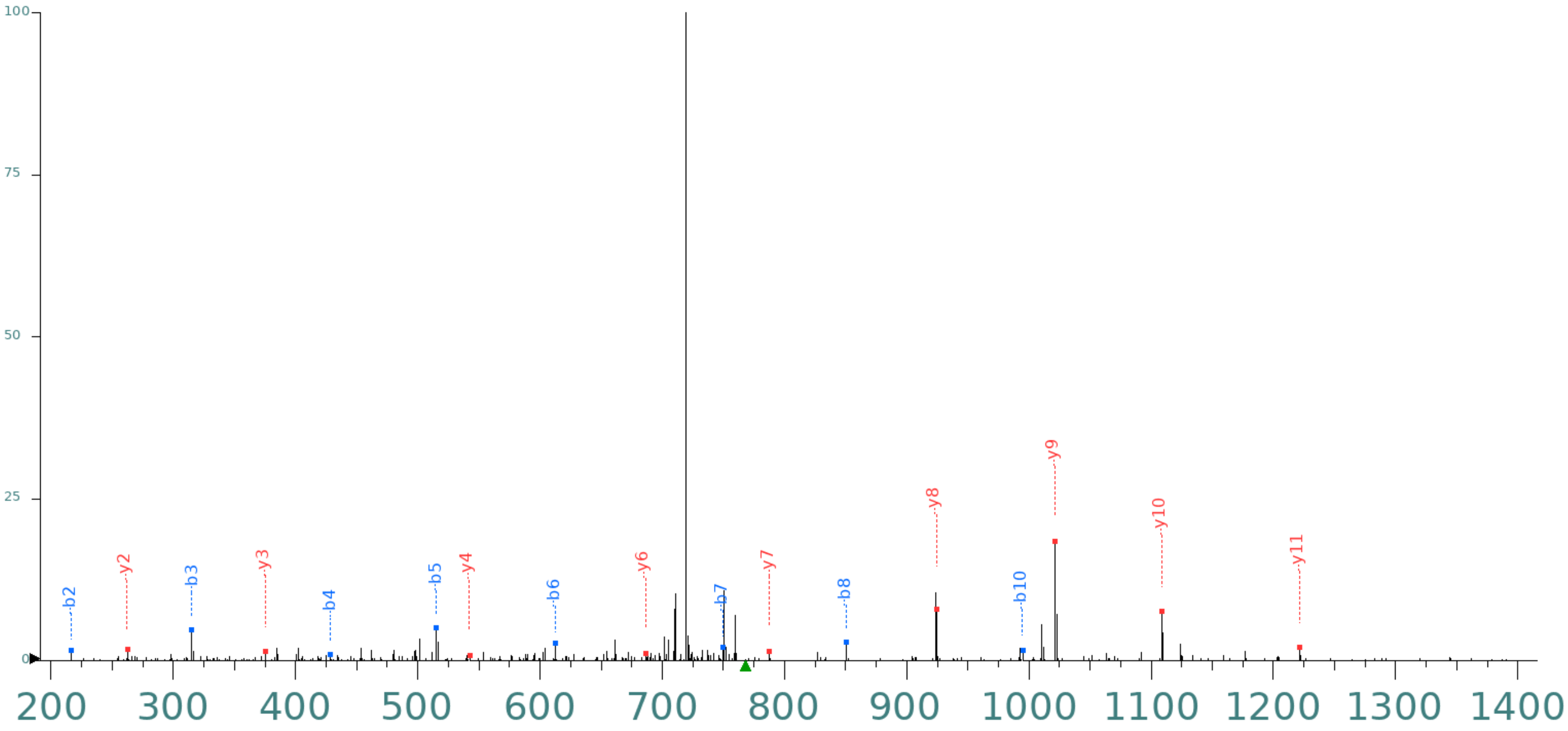

Predicted Fragmentation Pattern

Seq	#	b: Δ Error	b	y	y: Δ Error	+1
T	1	---	102.055	---	---	14
N	2	---	216.098	1434.674	---	13
V	3	223.900	315.166	1320.631	---	12
L	4	-238.125	428.250	1221.562	317.205	11
S	5	480.125	515.282	1108.478	55.713	10
P	6	-590.743	612.335	1021.446	139.305	9
H	7	39.879	749.394	924.393	352.733	8
T	8	96.411	850.442	787.335	438.449	7
S#	9	---	1017.440	686.287	---	6
G	10	---	1074.462	519.289	470.817	5
S	11	---	1161.494	462.267	12.711	4
I	12	---	1274.578	375.235	226.292	3
S	13	---	1361.610	262.151	---	2
R	14	---	---	175.119	---	1

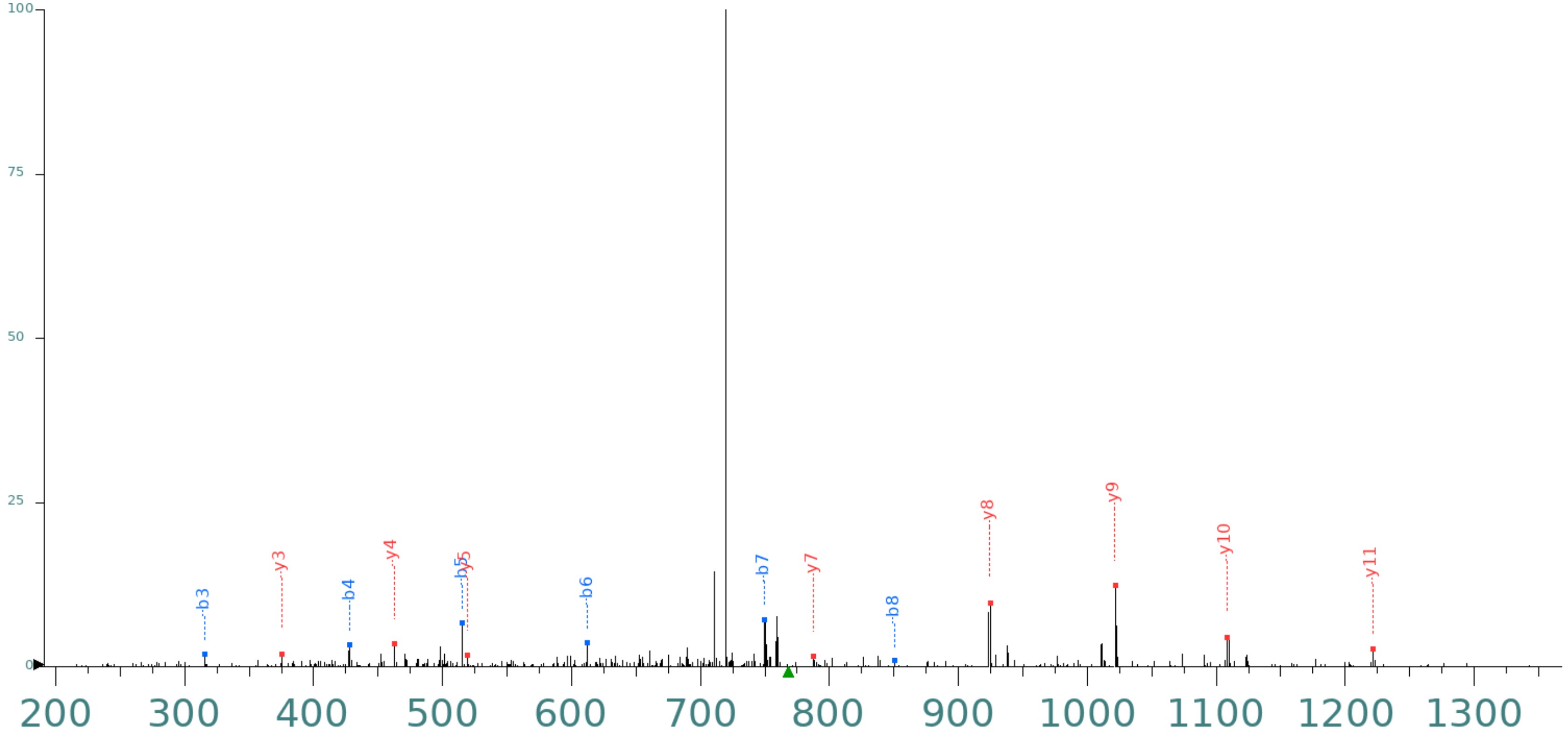

Predicted Fragmentation Pattern

Seq	#	b: Δ Error	b	y	y: Δ Error	+1
T	1	---	102.055	---	---	14
N	2	---	216.098	1434.674	---	13
V	3	5.308	**315.166**	1320.631	---	12
L	4	430.460	**428.250**	**1221.562**	342.570	11
S	5	248.843	**515.282**	**1108.478**	110.436	10
P	6	27.397	**612.335**	**1021.446**	211.820	9
H	7	339.242	**749.394**	**924.393**	222.998	8
T	8	127.337	**850.442**	**787.335**	333.566	7
S#	9	---	1017.440	**686.287**	532.217	6
G	10	---	1074.462	519.289	---	5
S	11	---	1161.494	**462.267**	386.087	4
I	12	---	1274.578	**375.235**	460.116	3
S	13	---	1361.610	**262.151**	628.540	2
R	14	---	---	175.119	---	1

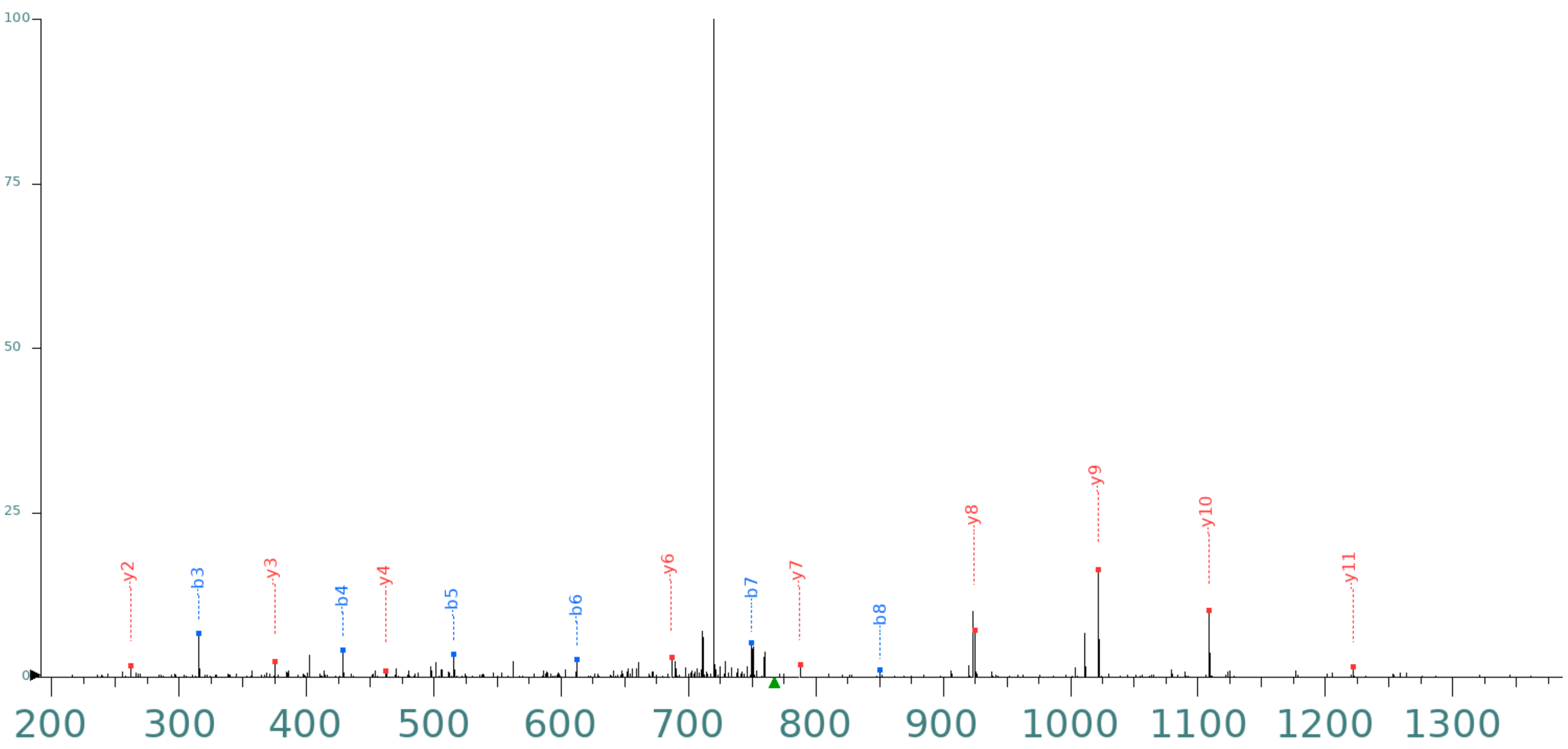

Predicted Fragmentation Pattern

Seq	#	b: Δ Error	b	y	y: Δ Error	+1
I	1	---	114.091	---	---	8
P	2	-255.312	211.144	839.402	271.244	7
S#	3	348.260	378.142	742.349	33.615	6
T	4	---	479.190	575.351	121.341	5
V	5	235.092	578.259	474.303	---	4
L	6	320.860	691.343	375.235	413.555	3
S	7	---	778.375	262.151	890.070	2
R	8	---	---	175.119	948.886	1

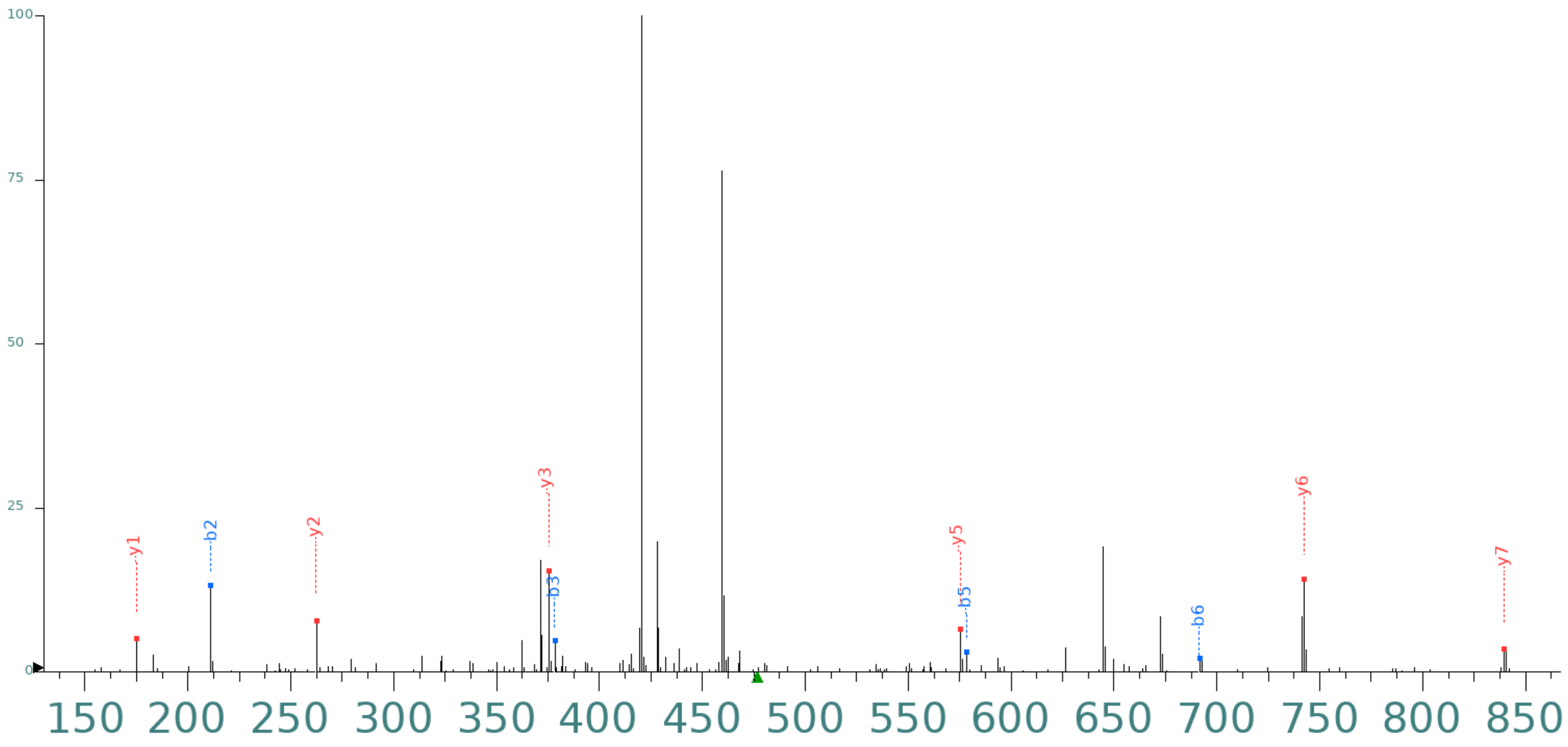

Predicted Fragmentation Pattern

Seq	#	b: Δ Error	b	y	y: Δ Error	+1
I	1	---	114.091	---	---	8
P	2	299.386	211.144	839.402	327.636	7
S#	3	374.470	378.142	742.349	309.118	6
T	4	---	479.190	575.351	371.680	5
V	5	-127.116	578.259	474.303	---	4
L	6	295.802	691.343	375.235	712.184	3
S	7	-24.875	778.375	262.151	81.209	2
R	8	---	---	175.119	303.337	1

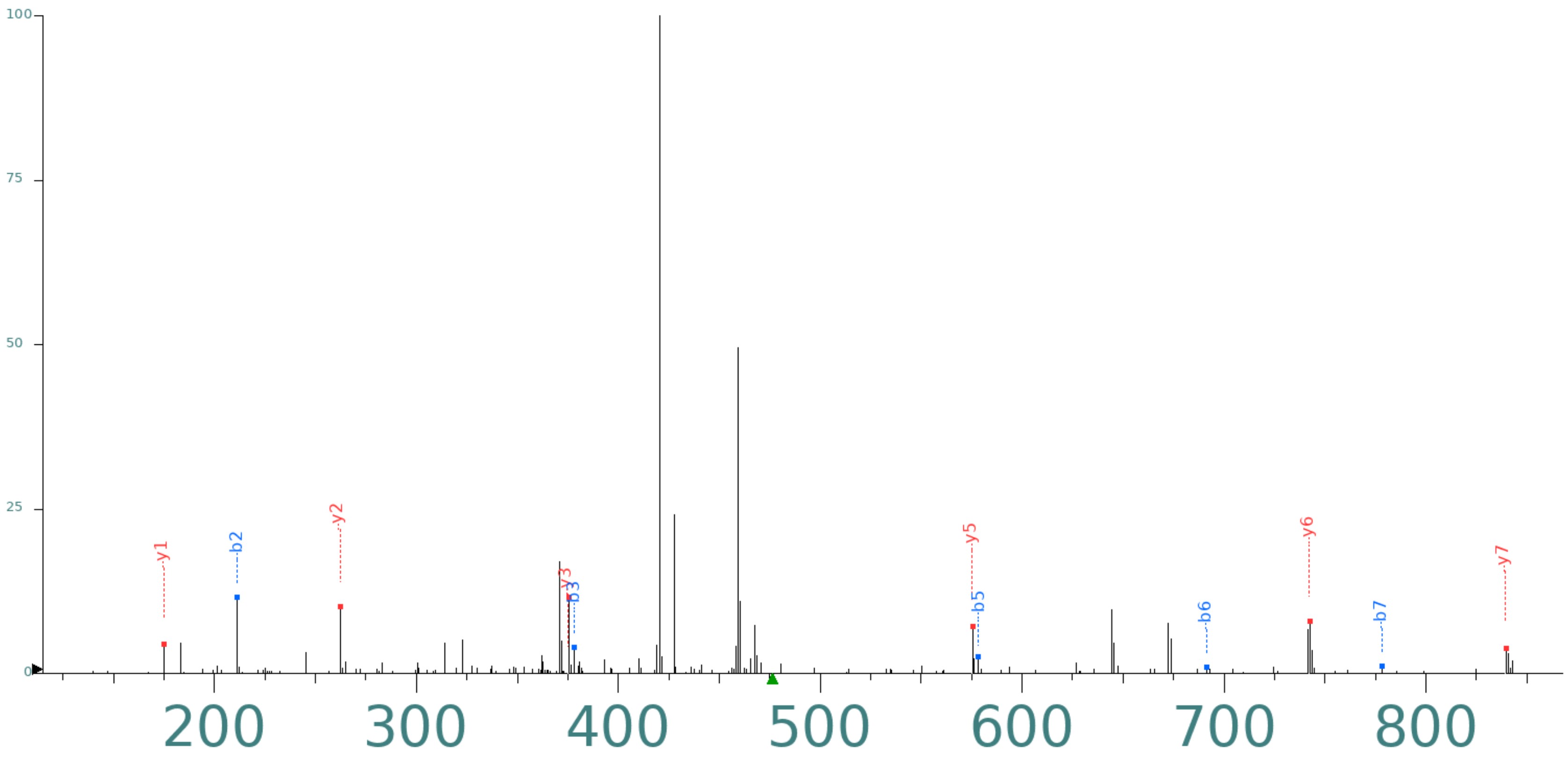

Predicted Fragmentation Pattern

Seq	#	b: Δ Error	b	y	y: Δ Error	+1
T	1	---	102.055	---	---	14
N	2	143.334	216.098	1434.674	---	13
V	3	193.121	315.166	1320.631	---	12
L	4	242.386	428.250	1221.562	-25.753	11
S#	5	---	595.249	1108.478	-49.895	10
P	6	---	692.301	941.480	131.196	9
H	7	320.196	829.360	844.427	126.725	8
T	8	---	930.408	707.368	26.980	7
S	9	---	1017.440	606.321	-76.928	6
G	10	---	1074.462	519.289	292.533	5
S	11	---	1161.494	462.267	---	4
I	12	325.889	1274.578	375.235	287.583	3
S	13	---	1361.610	262.151	618.076	2
R	14	---	---	175.119	---	1

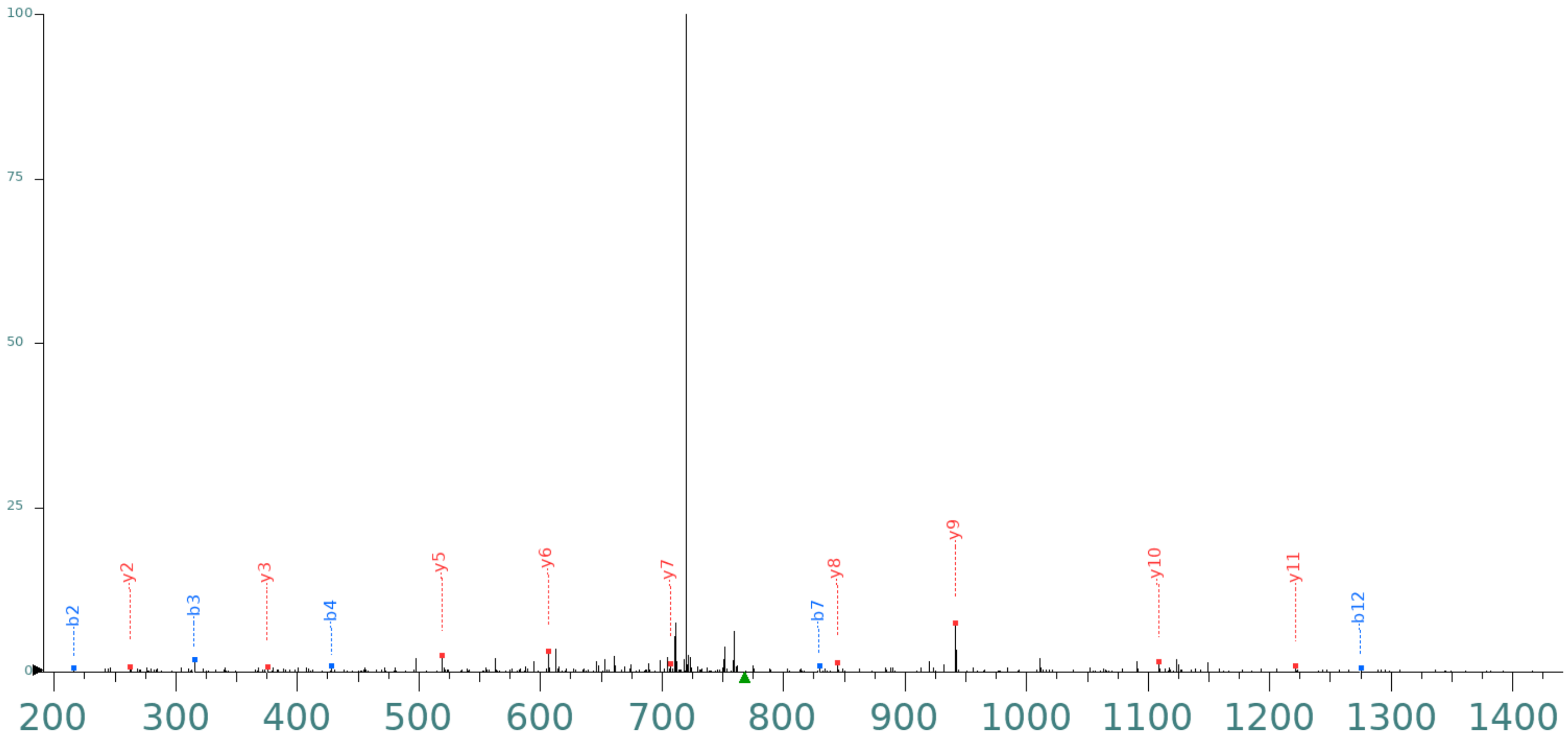

Predicted Fragmentation Pattern

Seq	#	b: Δ Error	b	y	y: Δ Error	+1
R	1	---	157.108	---	---	12
P	2	919.673	254.161	1292.672	---	11
S	3	102.125	341.193	1195.619	0.853	10
R	4	-316.835	497.294	1108.587	403.923	9
I	5	502.530	610.378	952.486	---	8
P	6	33.264	707.431	839.402	437.566	7
S#	7	---	874.429	742.349	---	6
T	8	---	975.477	575.351	-189.929	5
V	9	76.186	1074.546	474.303	-130.486	4
L	10	246.114	1187.630	375.235	309.854	3
S	11	---	1274.662	262.151	285.205	2
R	12	---	---	175.119	---	1

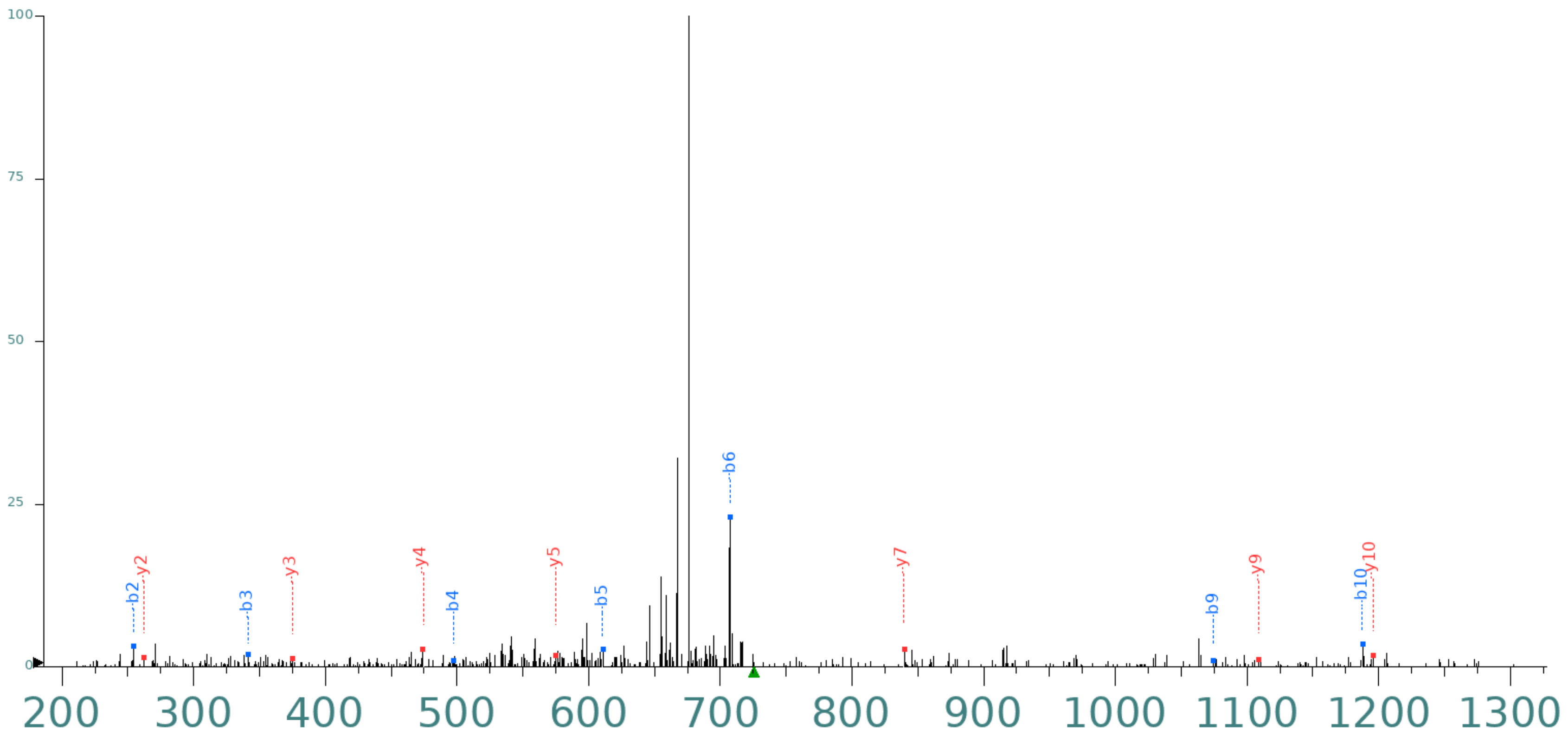

Predicted Fragmentation Pattern

Seq	#	b: Δ Error	b	y	y: Δ Error	+1
I	1	---	114.091	---	---	8
P	2	-136.603	211.144	839.402	36.382	7
S$^{\#}$	3	-72.898	378.142	742.349	80.145	6
T	4	---	479.190	575.351	377.828	5
V	5	107.421	578.259	474.303	---	4
L	6	634.237	691.343	375.235	275.715	3
S	7	483.404	778.375	262.151	-483.384	2
R	8	---	---	175.119	20.069	1

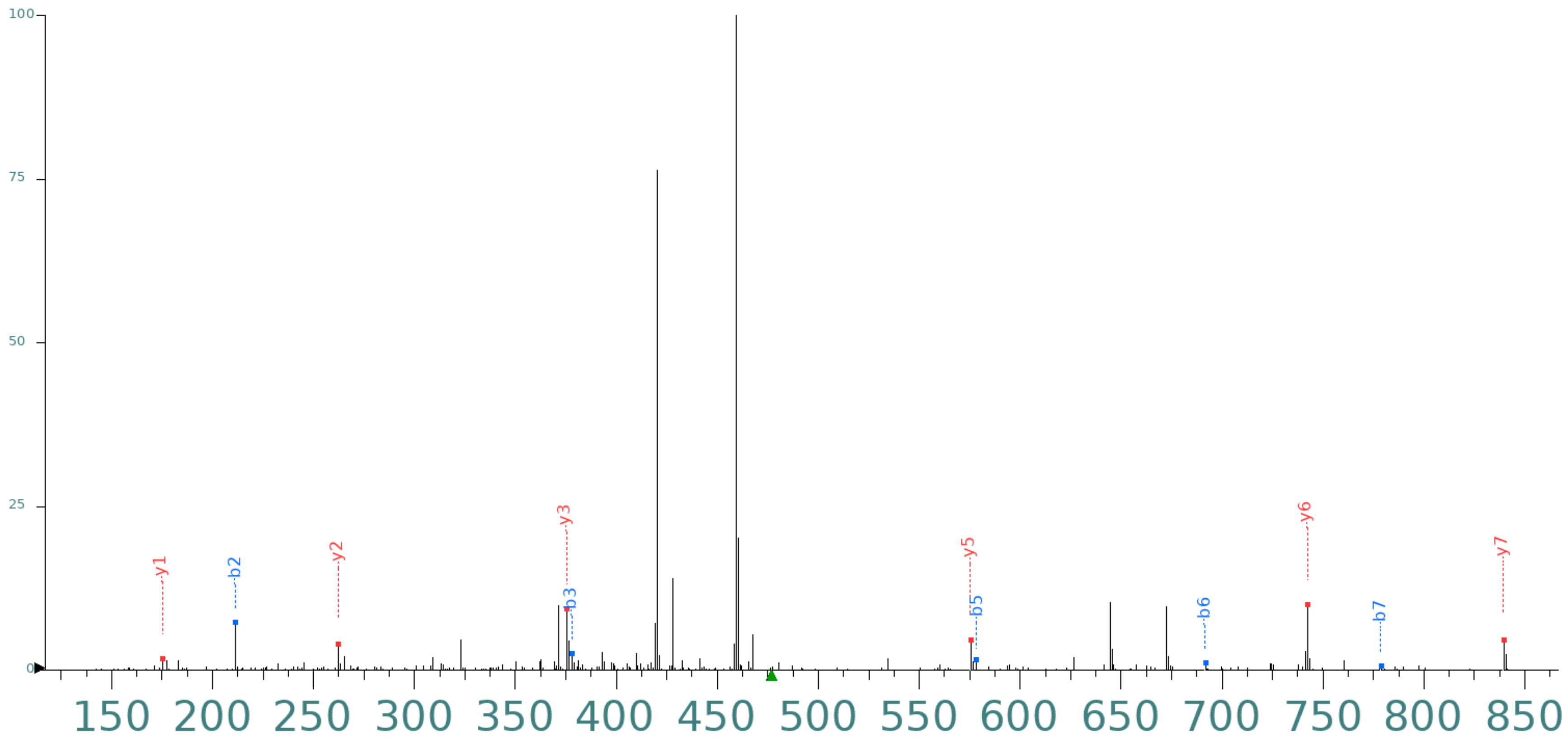

Predicted Fragmentation Pattern

Seq	#	b: Δ Error	b	y	y: Δ Error	+1
T	1	---	102.055	---	---	14
N	2	228.105	**216.098**	1434.674	---	13
V	3	-39.235	**315.166**	1320.631	---	12
L	4	---	428.250	**1221.562**	62.381	11
S	5	---	515.282	**1108.478**	129.483	10
P	6	-17.158	**612.335**	**1021.446**	188.406	9
H	7	-49.550	**749.394**	924.393	---	8
T#	8	-8.650	**930.408**	787.335	---	7
S	9	---	1017.440	**606.321**	-221.325	6
G	10	---	1074.462	**519.289**	-239.172	5
S	11	---	1161.494	**462.267**	-204.792	4
I	12	---	1274.578	**375.235**	-211.904	3
S	13	---	1361.610	**262.151**	-136.843	2
R	14	---	---	175.119	---	1

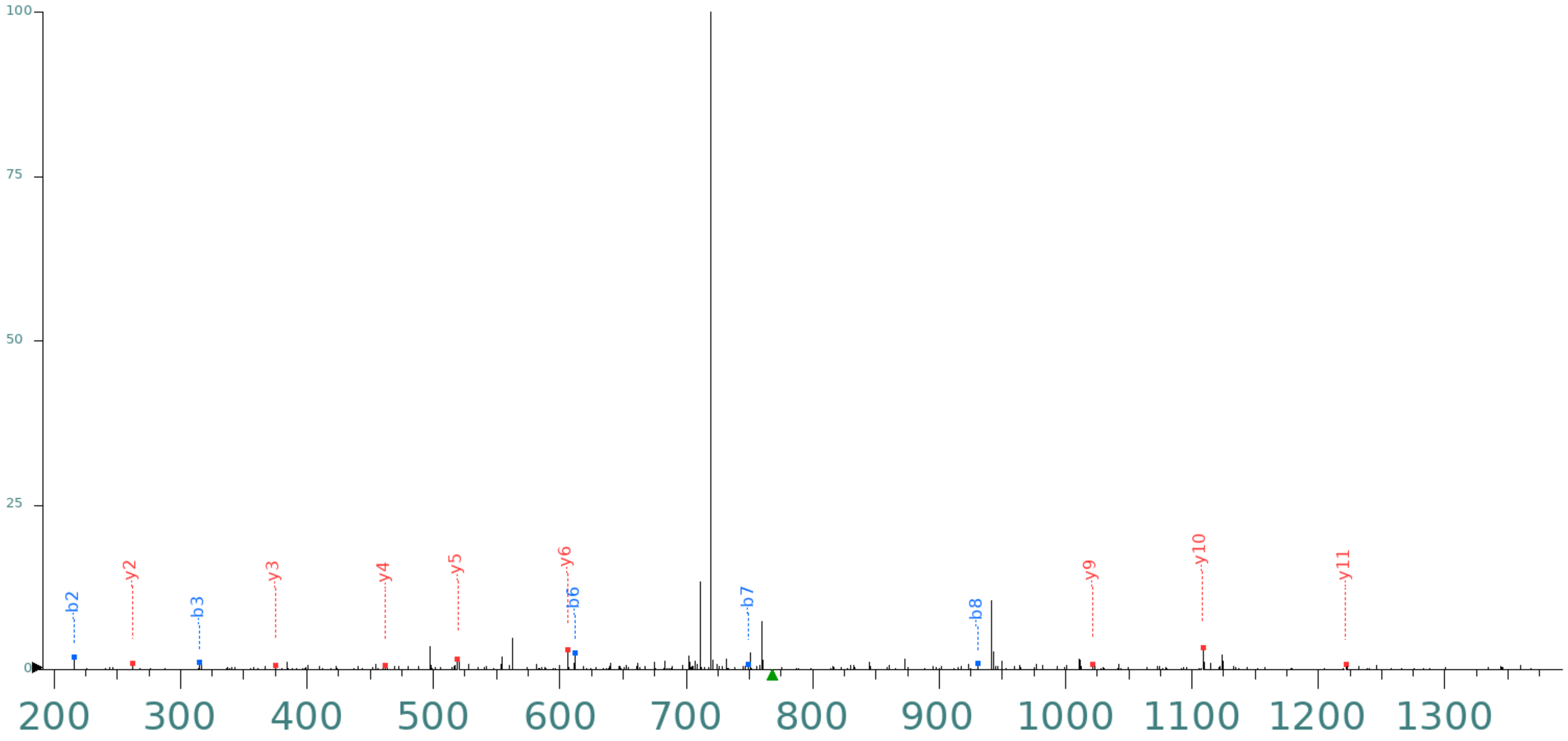

Predicted Fragmentation Pattern

Seq	#	b: Δ Error	b	y	y: Δ Error	+1
I	1	---	114.091	---	---	8
P	2	-440.589	211.144	839.402	63.428	7
S#	3	-93.723	378.142	742.349	62.635	6
T	4	---	479.190	575.351	37.230	5
V	5	81.460	578.259	474.303	304.390	4
L	6	301.625	691.343	375.235	-255.191	3
S	7	---	778.375	262.151	-161.297	2
R	8	---	---	175.119	567.209	1

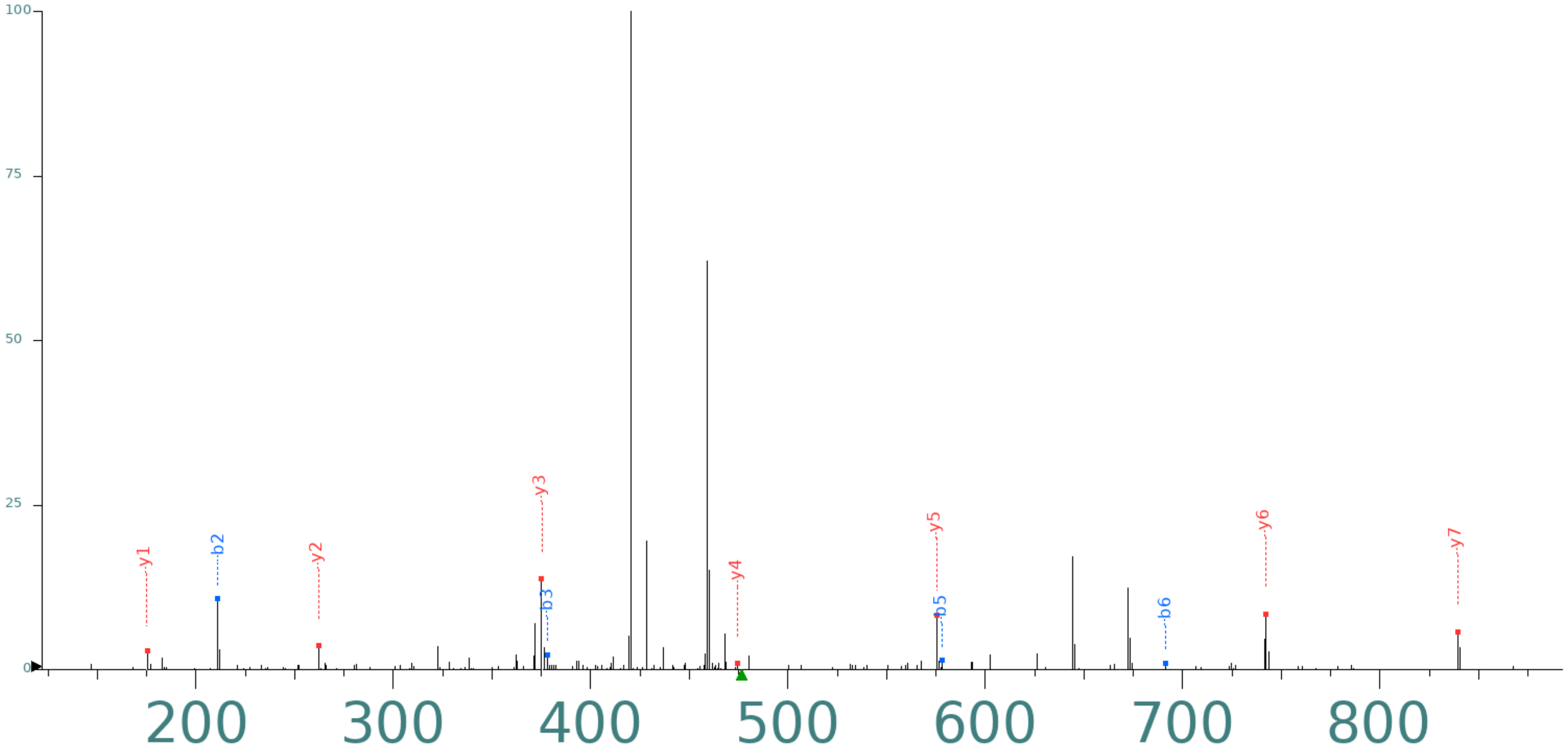

Predicted Fragmentation Pattern

Seq	#	b: Δ Error	b	y	y: Δ Error	+1
I	1	---	114.091	---	---	8
P	2	-273.244	211.144	839.402	254.747	7
S#	3	201.460	378.142	742.349	373.205	6
T	4	---	479.190	575.351	411.006	5
V	5	---	578.259	474.303	---	4
L	6	-585.537	691.343	375.235	-130.058	3
S	7	---	778.375	262.151	135.562	2
R	8	---	---	175.119	904.269	1

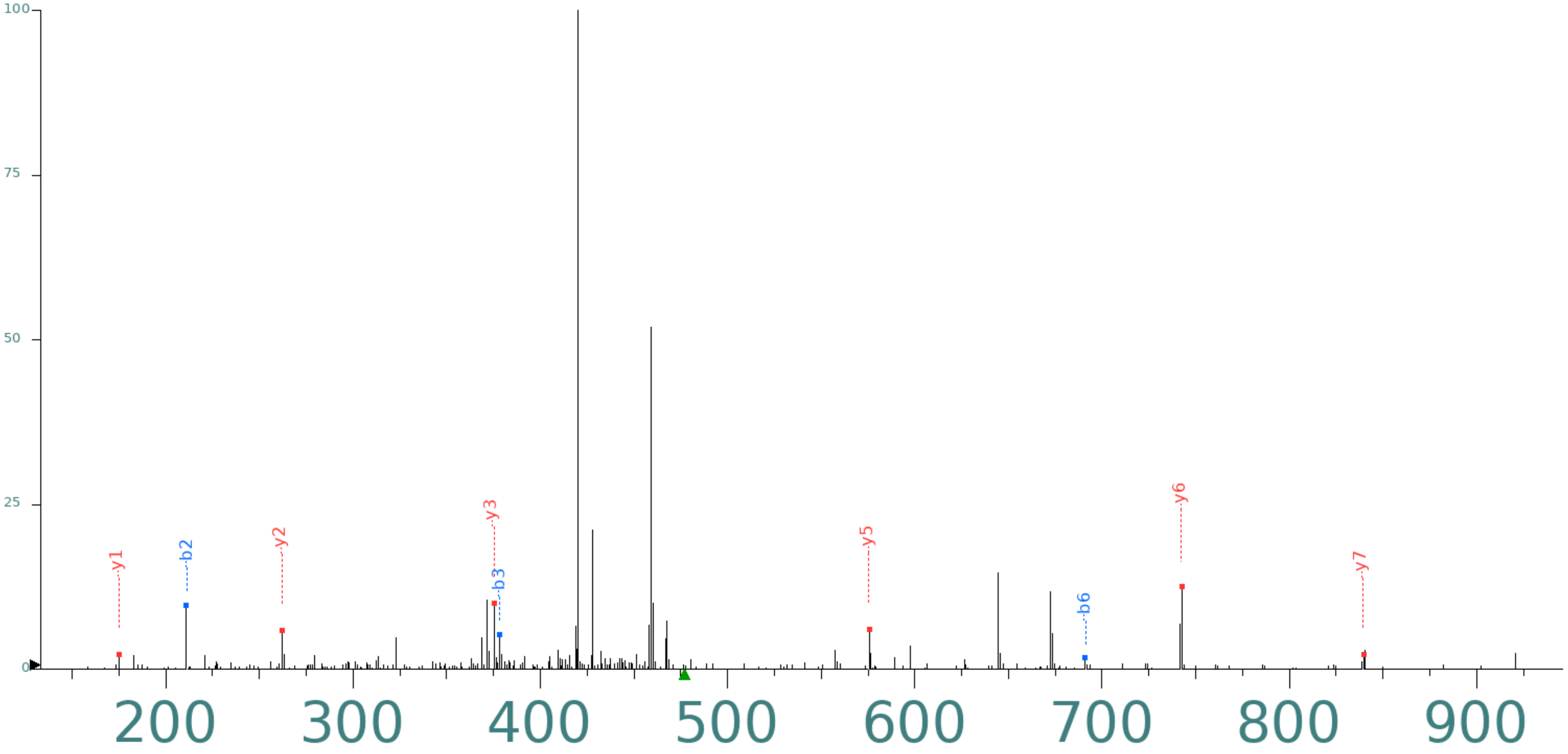

Predicted Fragmentation Pattern

Seq	#	b: Δ Error	b	y	y: Δ Error	+1
R	1	---	157.108	---	---	12
P	2	-413.048	254.161	1292.672	-84.230	11
S	3	---	341.193	1195.619	---	10
R	4	---	497.294	1108.587	---	9
I	5	648.954	610.378	952.486	---	8
P	6	47.240	707.431	839.402	547.762	7
S#	7	---	874.429	742.349	---	6
T	8	---	975.477	575.351	354.719	5
V	9	---	1074.546	474.303	804.158	4
L	10	---	1187.630	375.235	---	3
S	11	160.065	1274.662	262.151	---	2
R	12	---	---	175.119	---	1

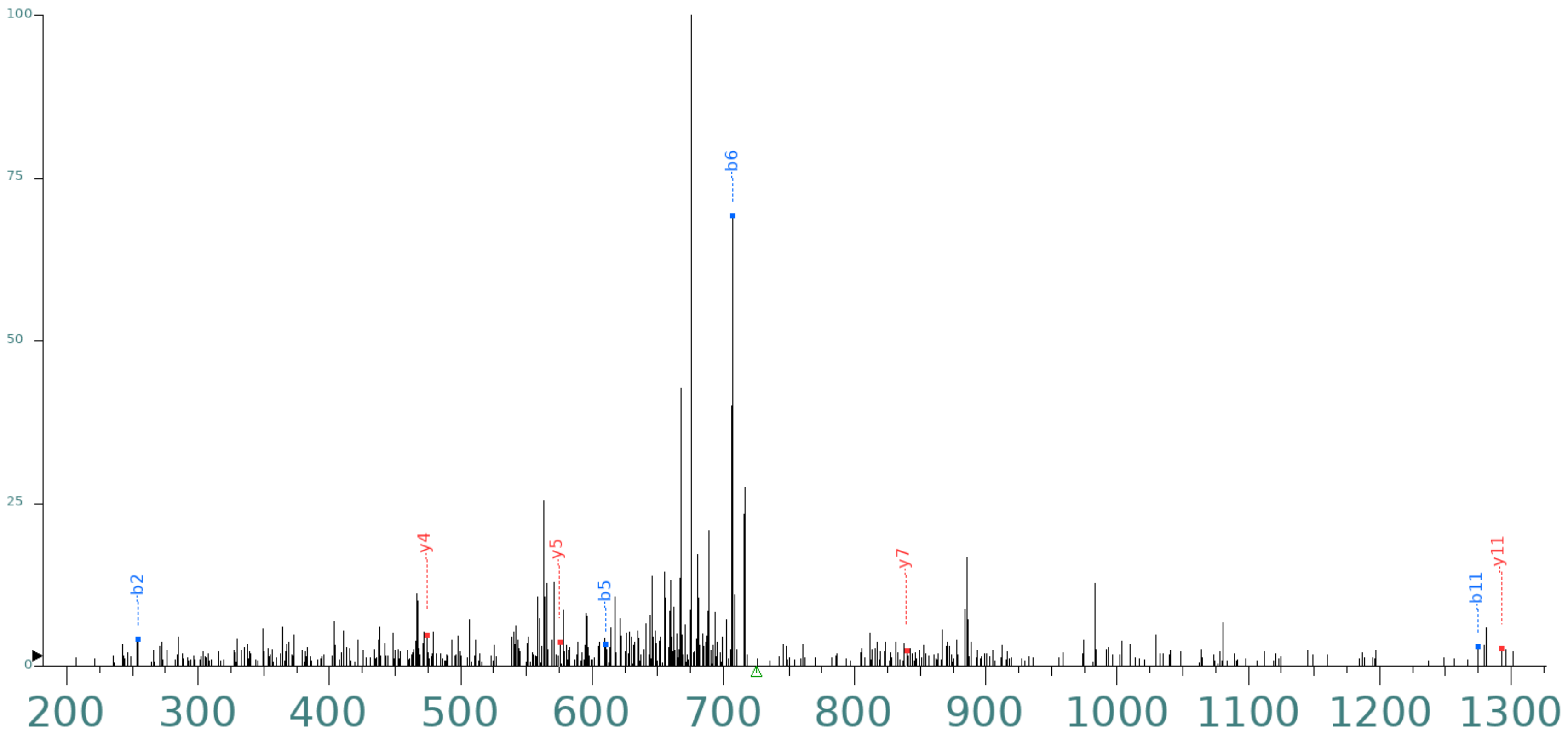

Predicted Fragmentation Pattern

Seq	#	b: Δ Error	b	y	y: Δ Error	+1
R	1	---	157.108	---	---	12
P	2	314.898	254.161	1292.672	---	11
S	3	---	341.193	1195.619	---	10
R	4	---	497.294	1108.587	---	9
I	5	115.986	610.378	952.486	---	8
P	6	-93.571	707.431	839.402	388.525	7
S	7	458.194	794.463	742.349	---	6
T#	8	-154.866	975.477	655.317	241.413	5
V	9	---	1074.546	474.303	---	4
L	10	---	1187.630	375.235	673.931	3
S	11	---	1274.662	262.151	---	2
R	12	---	---	175.119	---	1

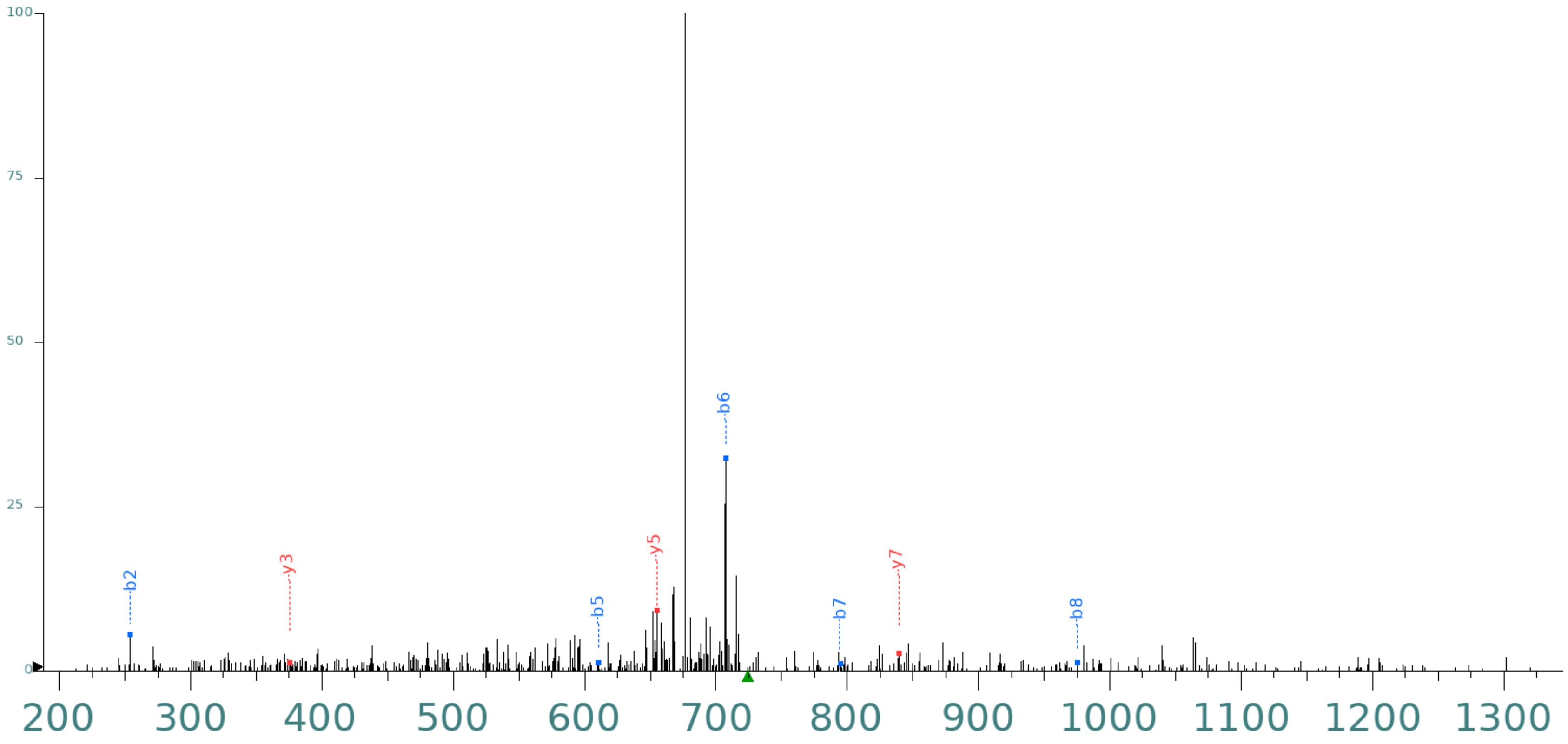

Basal

Predicted Fragmentation Pattern

Seq	#	b: Δ Error	b	y	y: Δ Error	+1
S	1	---	88.039	---	---	20
D	2	---	203.066	2151.890	---	19
T#	3	---	384.080	2036.863	---	18
G	4	35.618	441.102	1855.849	-18.850	17
S	5	---	528.134	1798.828	43.244	16
Q	6	280.910	656.192	1711.796	---	15
Y	7	263.615	819.256	1583.737	107.396	14
I	8	115.469	932.340	1420.674	3.290	13
E	9	9.328	1061.382	1307.590	41.723	12
D	10	-19.157	1176.409	1178.547	7.030	11
S	11	44.540	1263.441	1063.520	75.587	10
I	12	76.734	1376.525	976.488	24.622	9
S	13	137.517	1463.557	863.404	-28.897	8
Q	14	27.082	1591.616	776.372	109.735	7
G	15	---	1648.637	648.313	-2.851	6
A	16	-6.371	1719.675	591.292	142.158	5
V	17	91.393	1818.743	520.255	-36.199	4
C	18	---	1978.774	421.186	-355.836	3
N	19	---	2092.817	261.156	---	2
K	20	---	---	147.113	---	1

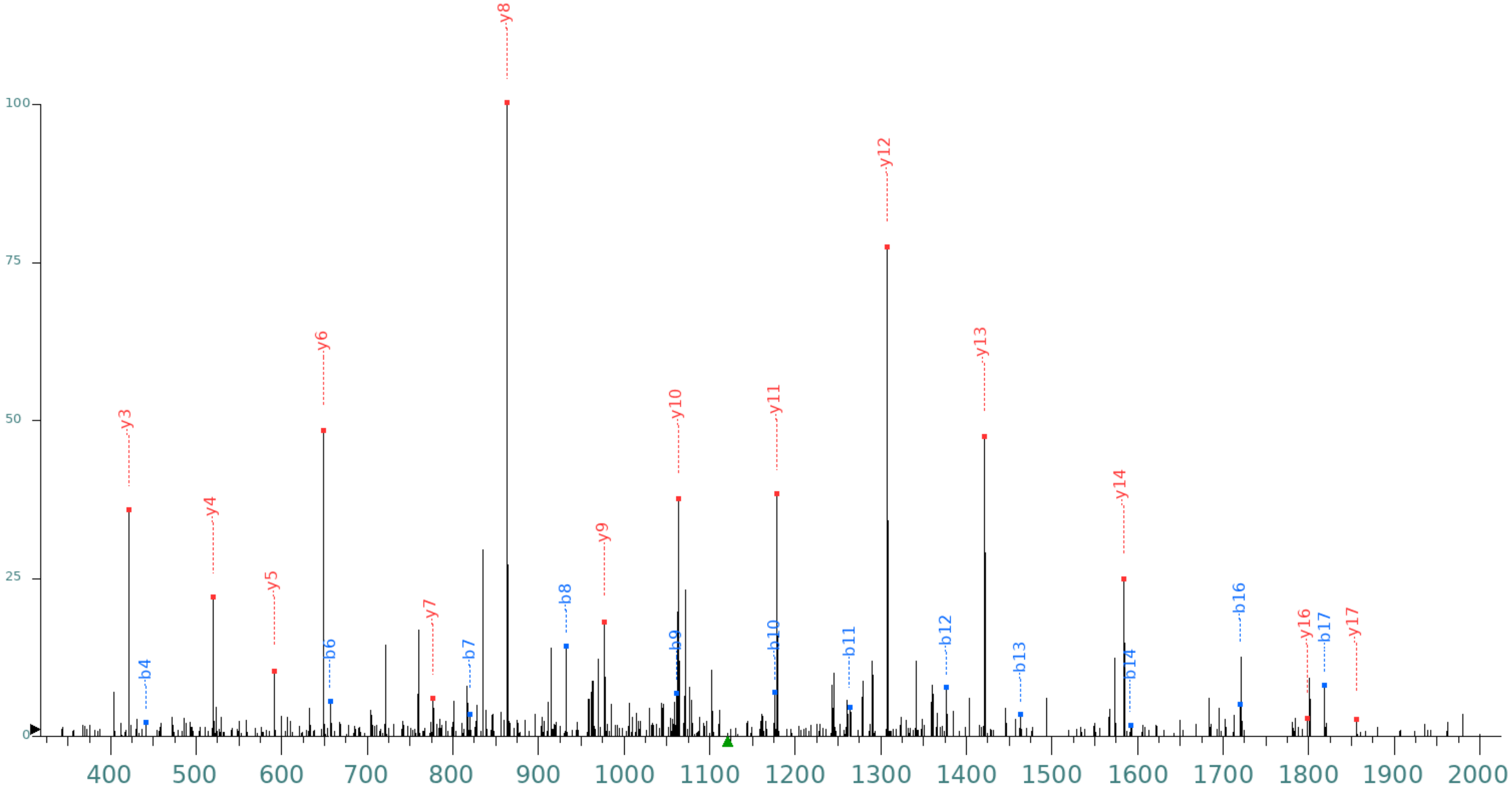

Predicted Fragmentation Pattern

Seq	#	b: Δ Error	b	y	y: Δ Error	+1
S	1	---	88.039	---	---	20
D	2	---	203.066	2151.890	---	19
T	3	---	304.114	2036.863	---	18
G	4	---	361.135	1935.815	---	17
S	5	41.198	**448.167**	1878.794	---	16
Q	6	238.616	**576.226**	1791.762	---	15
Y	7	-40.810	**739.289**	**1663.703**	149.902	14
I	8	-54.681	**852.373**	**1500.640**	157.714	13
E	9	124.028	**981.416**	**1387.556**	175.987	12
D	10	125.074	**1096.443**	**1258.513**	84.155	11
S	11	140.793	**1183.475**	**1143.486**	280.084	10
I	12	-8.988	**1296.559**	1056.454	---	9
S#	13	---	1463.557	**943.370**	126.074	8
Q	14	---	1591.616	**776.372**	132.213	7
G	15	---	1648.637	**648.313**	-85.140	6
A	16	97.896	**1719.675**	**591.292**	157.843	5
V	17	147.490	**1818.743**	**520.255**	9.321	4
C	18	145.057	**1978.774**	**421.186**	228.670	3
N	19	---	2092.817	261.156	---	2
K	20	---	---	147.113	---	1

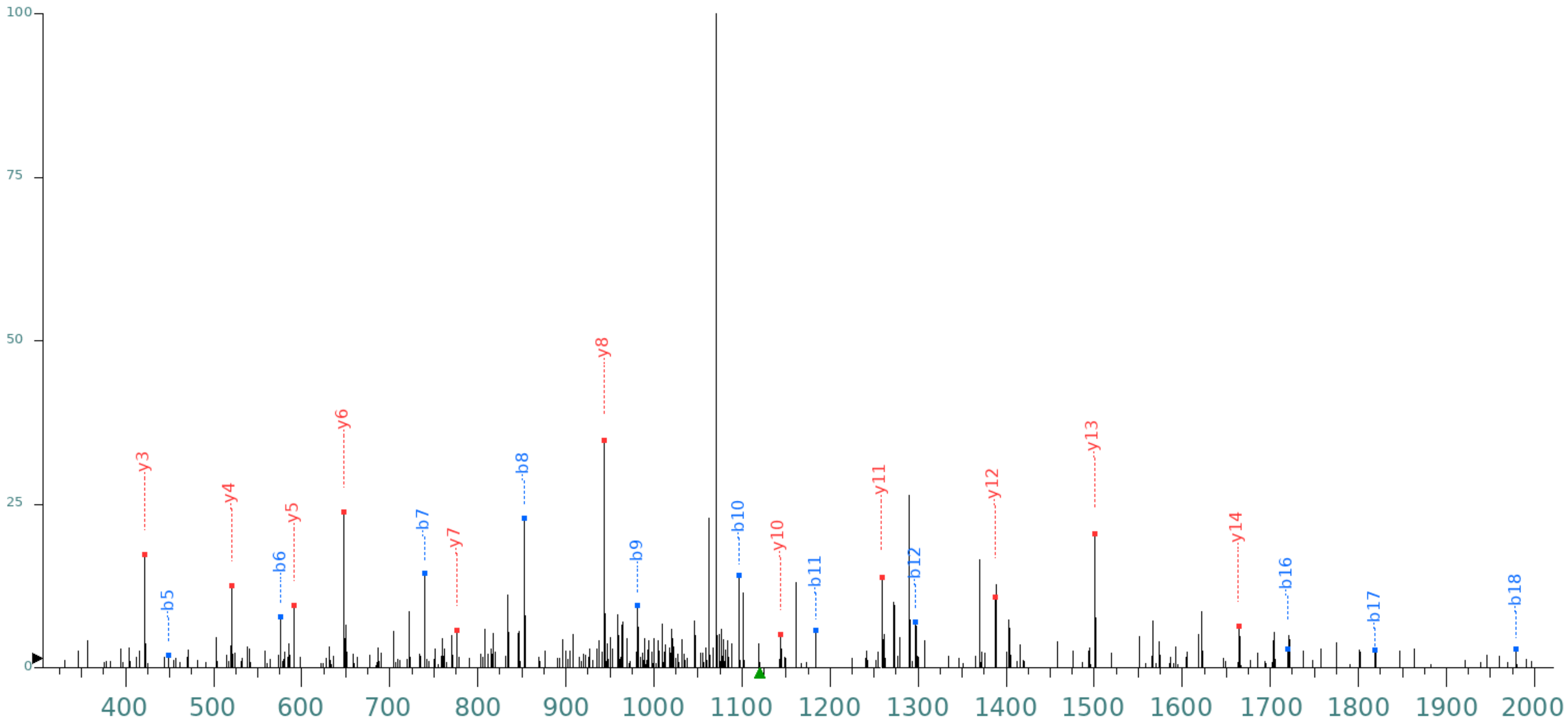

Predicted Fragmentation Pattern

+1

Seq	#	b: Δ Error	b	y	y: Δ Error	+1
S#	1	---	168.006	---	---	20
D	2	216.192	**283.033**	2071.924	---	19
T	3	308.526	**384.080**	1956.897	---	18
G	4	---	441.102	1855.849	---	17
S	5	623.984	**528.134**	1798.828	---	16
Q	6	-403.851	**656.192**	1711.796	---	15
Y	7	233.904	**819.256**	1583.737	---	14
I	8	316.228	**932.340**	1420.674	---	13
E	9	247.913	**1061.382**	1307.590	---	12
D	10	220.596	**1176.409**	**1178.547**	137.518	11
S	11	242.645	**1263.441**	**1063.520**	-30.235	10
I	12	229.837	**1376.525**	**976.488**	323.228	9
S	13	---	1463.557	**863.404**	72.116	8
Q	14	---	1591.616	**776.372**	112.564	7
G	15	---	1648.637	**648.313**	-8.217	6
A	16	---	1719.675	**591.292**	298.575	5
V	17	---	1818.743	**520.255**	194.880	4
C	18	---	1978.774	**421.186**	302.464	3
N	19	---	2092.817	**261.156**	326.386	2
K	20	---	---	147.113	---	1

+2

Seq	#	b: Δ Error	b	y	y: Δ Error	+1
S#	1	---	84.506	---	---	20
D	2	---	142.020	1036.465	---	19
T	3	---	192.544	**978.952**	-422.879	18
G	4	---	221.054	928.428	---	17
S	5	---	264.571	899.917	---	16
Q	6	---	328.600	**856.401**	259.997	15
Y	7	---	410.131	792.372	---	14
I	8	---	466.673	710.840	---	13
E	9	---	531.195	**654.298**	-33.365	12
D	10	-555.726	**588.708**	589.777	---	11
S	11	364.250	**632.224**	**532.264**	707.246	10
I	12	453.394	**688.766**	**488.748**	-847.364	9
S	13	-280.017	**732.282**	432.206	---	8
Q	14	---	796.312	**388.690**	620.564	7
G	15	-159.293	**824.822**	324.660	---	6
A	16	177.017	**860.341**	**296.150**	754.788	5
V	17	192.029	**909.875**	260.631	---	4
C	18	---	989.890	211.097	---	3
N	19	269.709	**1046.912**	131.082	---	2
K	20	---	---	74.060	---	1

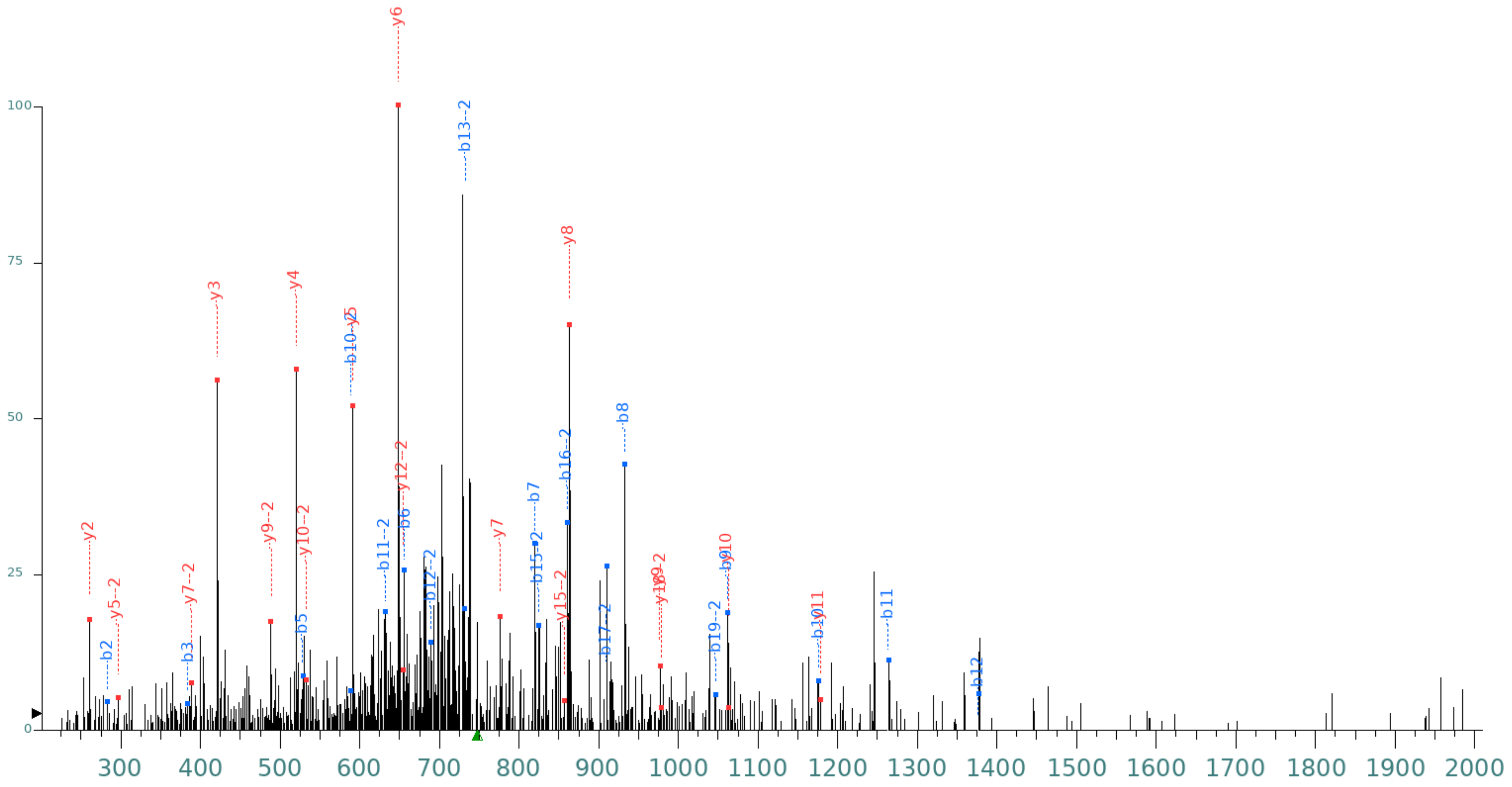

Predicted Fragmentation Pattern

Seq	#	b: Δ Error	b	y	y: Δ Error	+1
S	1	---	88.039	---	---	20
D	2	---	203.066	2151.890	---	19
T	3	---	304.114	2036.863	---	18
G	4	---	361.135	1935.815	---	17
S#	5	---	528.134	1878.794	---	16
Q	6	---	656.192	1711.796	---	15
Y	7	196.149	**819.256**	**1583.737**	184.682	14
I	8	218.214	**932.340**	**1420.674**	96.853	13
E	9	-9.304	**1061.382**	**1307.590**	99.502	12
D	10	251.504	**1176.409**	**1178.547**	172.309	11
S	11	141.622	**1263.441**	**1063.520**	49.076	10
I	12	149.613	**1376.525**	**976.488**	205.844	9
S	13	190.546	**1463.557**	**863.404**	53.668	8
Q	14	176.455	**1591.616**	**776.372**	138.344	7
G	15	---	1648.637	**648.313**	27.087	6
A	16	-2.964	**1719.675**	**591.292**	818.859	5
V	17	138.163	**1818.743**	**520.255**	511.766	4
C	18	---	1978.774	**421.186**	306.374	3
N	19	---	2092.817	261.156	---	2
K	20	---	---	147.113	---	1

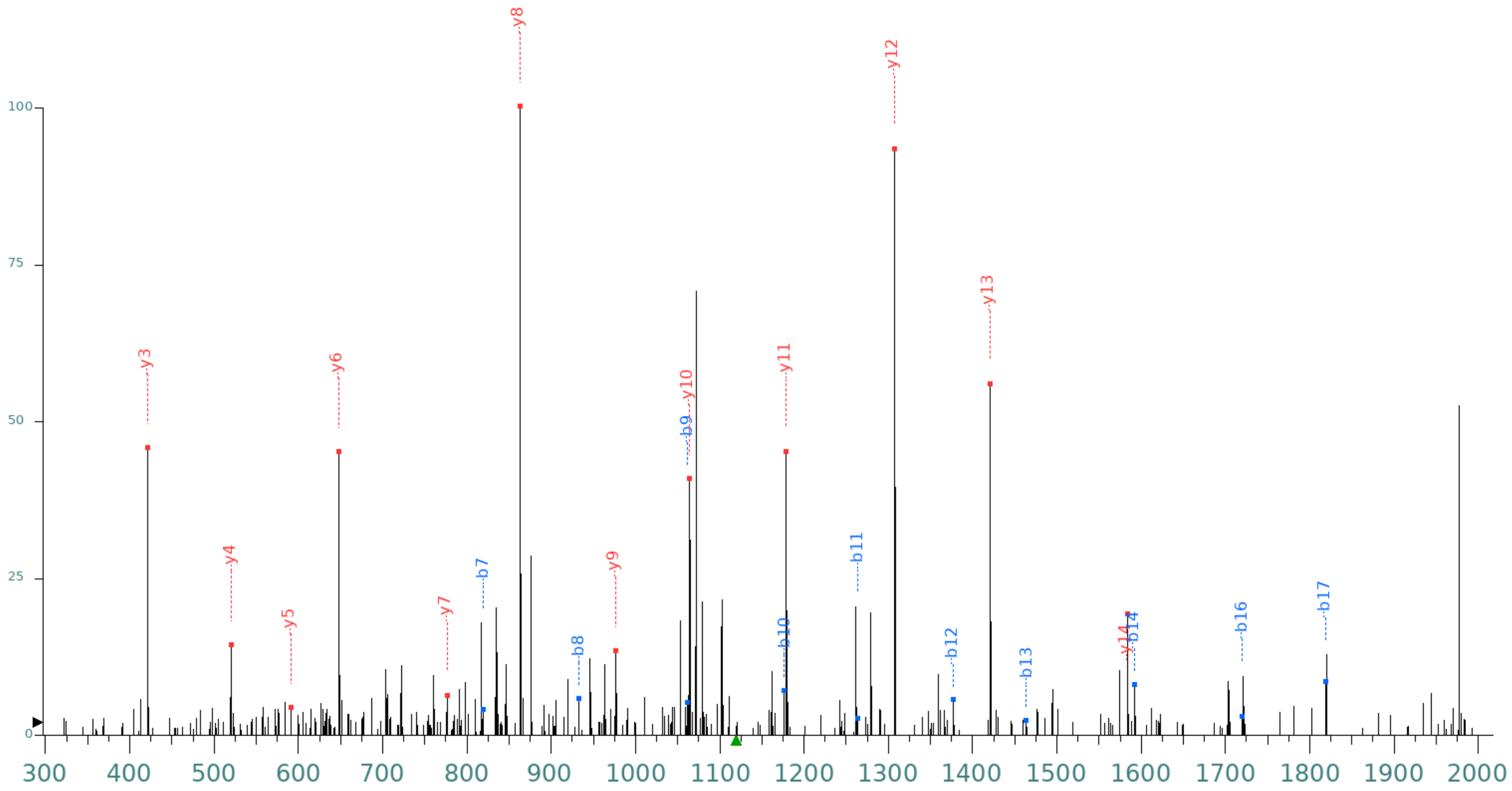

Predicted Fragmentation Pattern

Seq	#	b: Δ Error	b	y	y: Δ Error	+1
S	1	---	88.039	---	---	20
D	2	---	203.066	2151.890	---	19
T	3	---	304.114	2036.863	---	18
G	4	473.977	**361.135**	1935.815	---	17
S#	5	---	528.134	1878.794	---	16
Q	6	---	656.192	**1711.796**	49.248	15
Y	7	---	819.256	**1583.737**	184.837	14
I	8	234.442	**932.340**	**1420.674**	170.126	13
E	9	---	1061.382	**1307.590**	132.915	12
D	10	-44.373	**1176.409**	**1178.547**	69.689	11
S	11	---	1263.441	**1063.520**	205.939	10
I	12	194.646	**1376.525**	**976.488**	95.244	9
S	13	---	1463.557	**863.404**	3.339	8
Q	14	-36.500	**1591.616**	**776.372**	452.464	7
G	15	---	1648.637	**648.313**	230.763	6
A	16	95.554	**1719.675**	**591.292**	310.955	5
V	17	109.914	**1818.743**	**520.255**	211.415	4
C	18	230.157	**1978.774**	**421.186**	372.626	3
N	19	---	2092.817	261.156	---	2
K	20	---	---	147.113	---	1

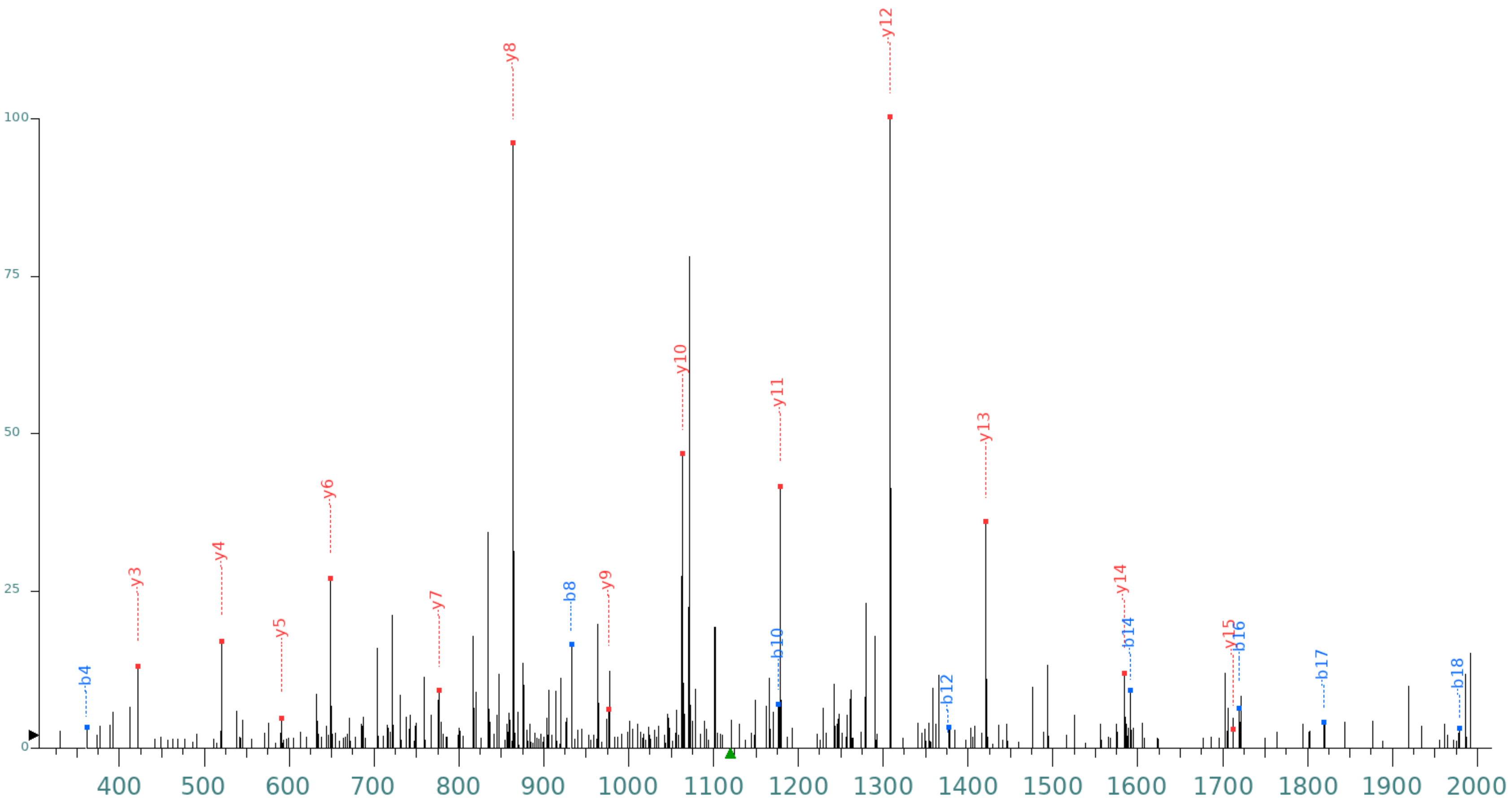

Predicted Fragmentation Pattern

+1

Seq	#	b: Δ Error	b	y	y: Δ Error	+1
T	1	---	102.055	---	---	14
N	2	-628.602	216.098	1434.674	---	13
V	3	-265.112	315.166	1320.631	---	12
L	4	-37.755	428.250	1221.562	---	11
S	5	---	515.282	1108.478	354.578	10
P	6	71.848	612.335	1021.446	---	9
H	7	---	749.394	924.393	124.784	8
T	8	---	850.442	787.335	454.869	7
S	9	---	937.474	686.287	248.023	6
G	10	---	994.495	599.255	126.408	5
S#	11	---	1161.494	542.233	---	4
I	12	---	1274.578	375.235	757.012	3
S	13	---	1361.610	262.151	-468.120	2
R	14	---	---	175.119	-64.455	1

+2

Seq	#	b: Δ Error	b	y	y: Δ Error	+1
T	1	---	51.531	---	---	14
N	2	---	108.553	717.840	86.768	13
V	3	---	158.087	660.819	19.233	12
L	4	1753.138	214.629	611.285	477.310	11
S	5	591.470	258.145	554.743	65.607	10
P	6	1228.527	306.671	511.227	---	9
H	7	848.552	375.201	462.700	648.311	8
T	8	-558.494	425.725	394.171	---	7
S	9	-468.575	469.241	343.647	-750.584	6
G	10	655.037	497.751	300.131	511.705	5
S#	11	---	581.250	271.620	-1061.667	4
I	12	---	637.792	188.121	-29.312	3
S	13	---	681.308	131.579	---	2
R	14	---	---	88.063	---	1

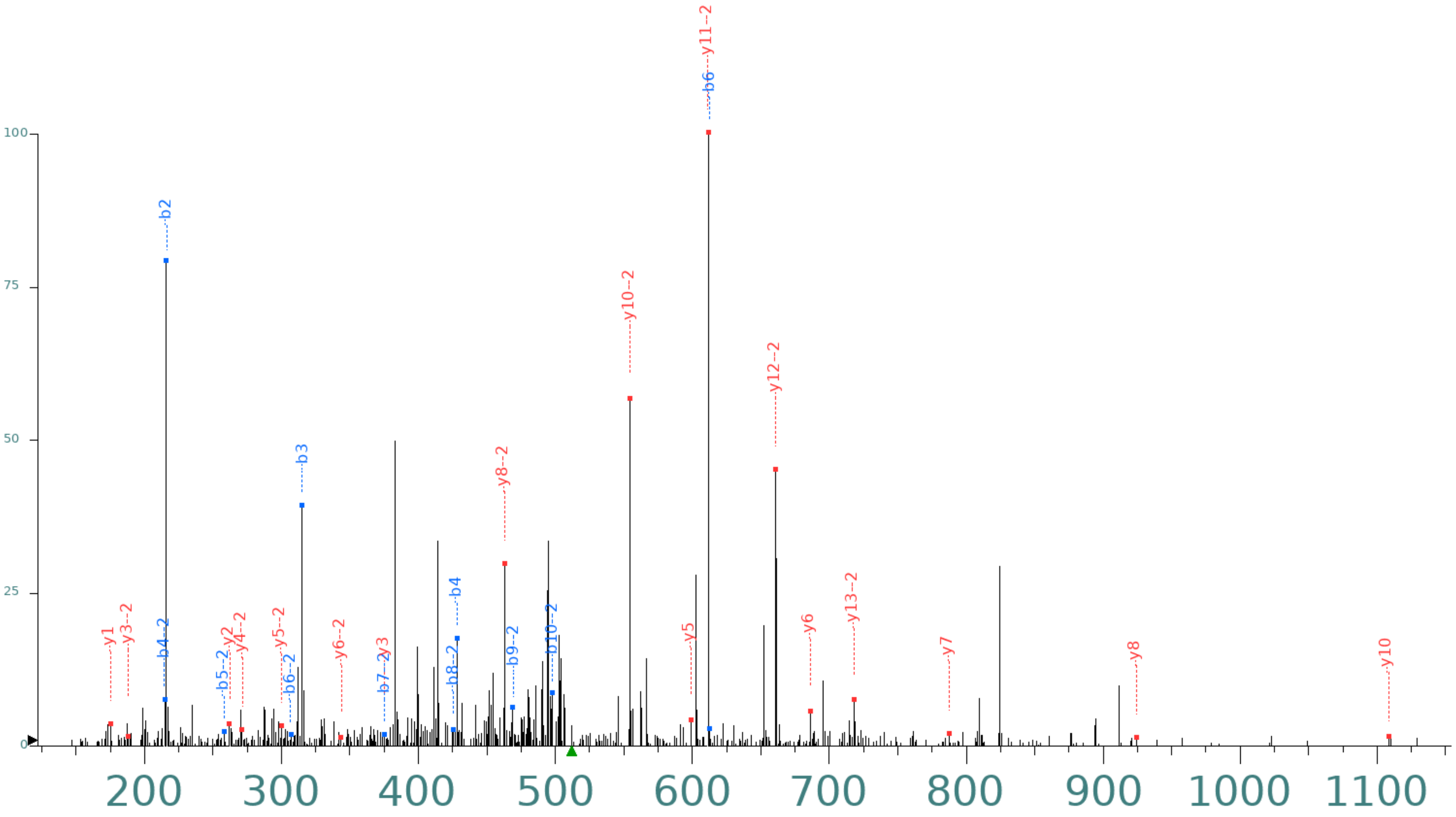

Predicted Fragmentation Pattern

Seq	#	b: Δ Error	b	y	y: Δ Error	+1
T	1	---	102.055	---	---	14
N	2	248.643	216.098	1434.674	---	13
V	3	394.506	315.166	1320.631	---	12
L	4	341.310	428.250	1221.562	175.275	11
S#	5	---	595.249	1108.478	-8.376	10
P	6	605.532	692.301	941.480	158.611	9
H	7	298.500	829.360	844.427	351.047	8
T	8	165.885	930.408	707.368	84.094	7
S	9	319.902	1017.440	606.321	495.112	6
G	10	---	1074.462	519.289	159.073	5
S	11	---	1161.494	462.267	125.981	4
I	12	---	1274.578	375.235	178.734	3
S	13	270.984	1361.610	262.151	-197.514	2
R	14	---	---	175.119	---	1

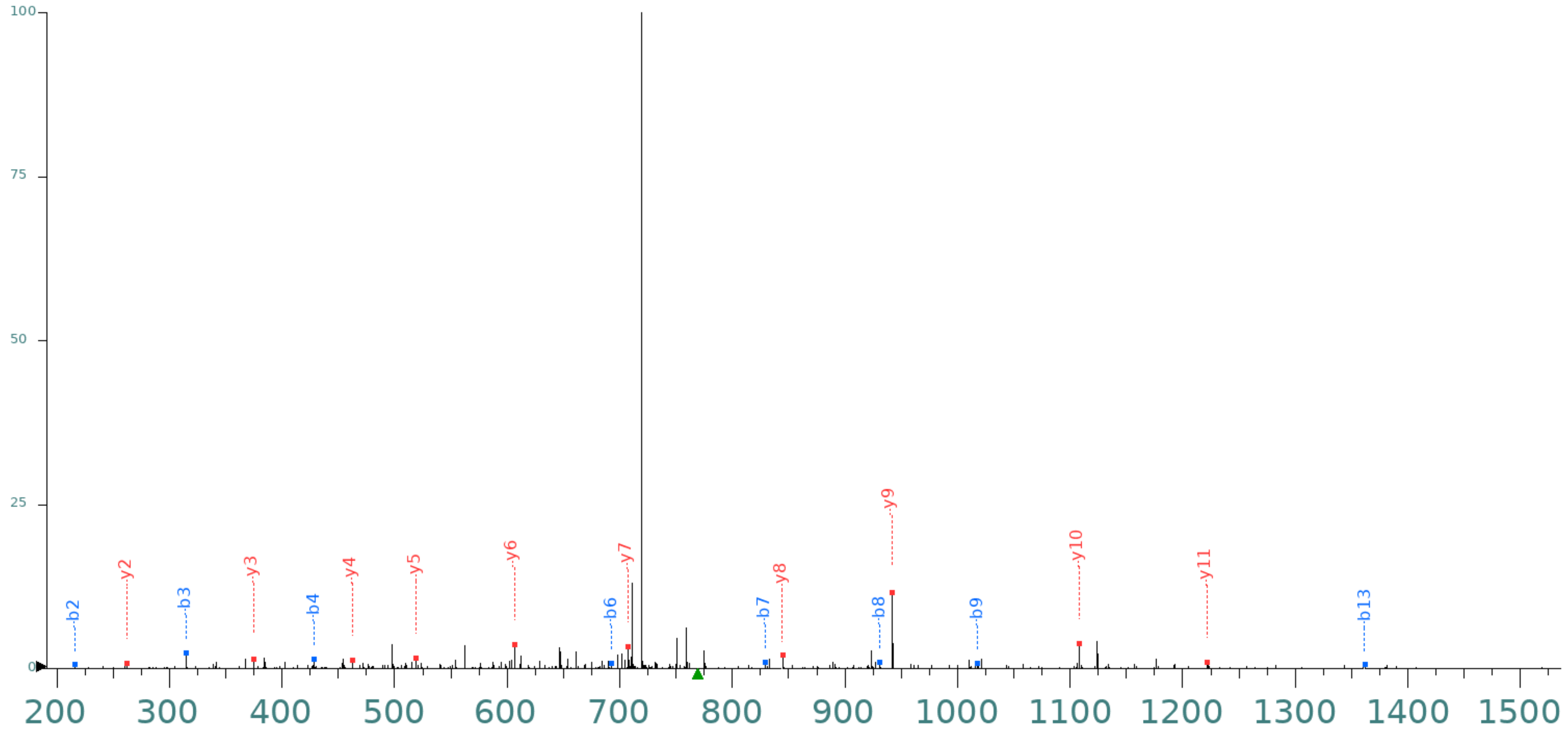

Predicted Fragmentation Pattern

Seq	#	b: Δ Error	b	y	y: Δ Error	+1
I	1	---	114.091	---	---	8
P	2	-745.485	211.144	839.402	52.014	7
S#	3	192.263	378.142	742.349	83.927	6
T	4	---	479.190	575.351	196.742	5
V	5	94.546	578.259	474.303	---	4
L	6	190.794	691.343	375.235	-142.749	3
S	7	---	778.375	262.151	-383.416	2
R	8	---	---	175.119	29.217	1

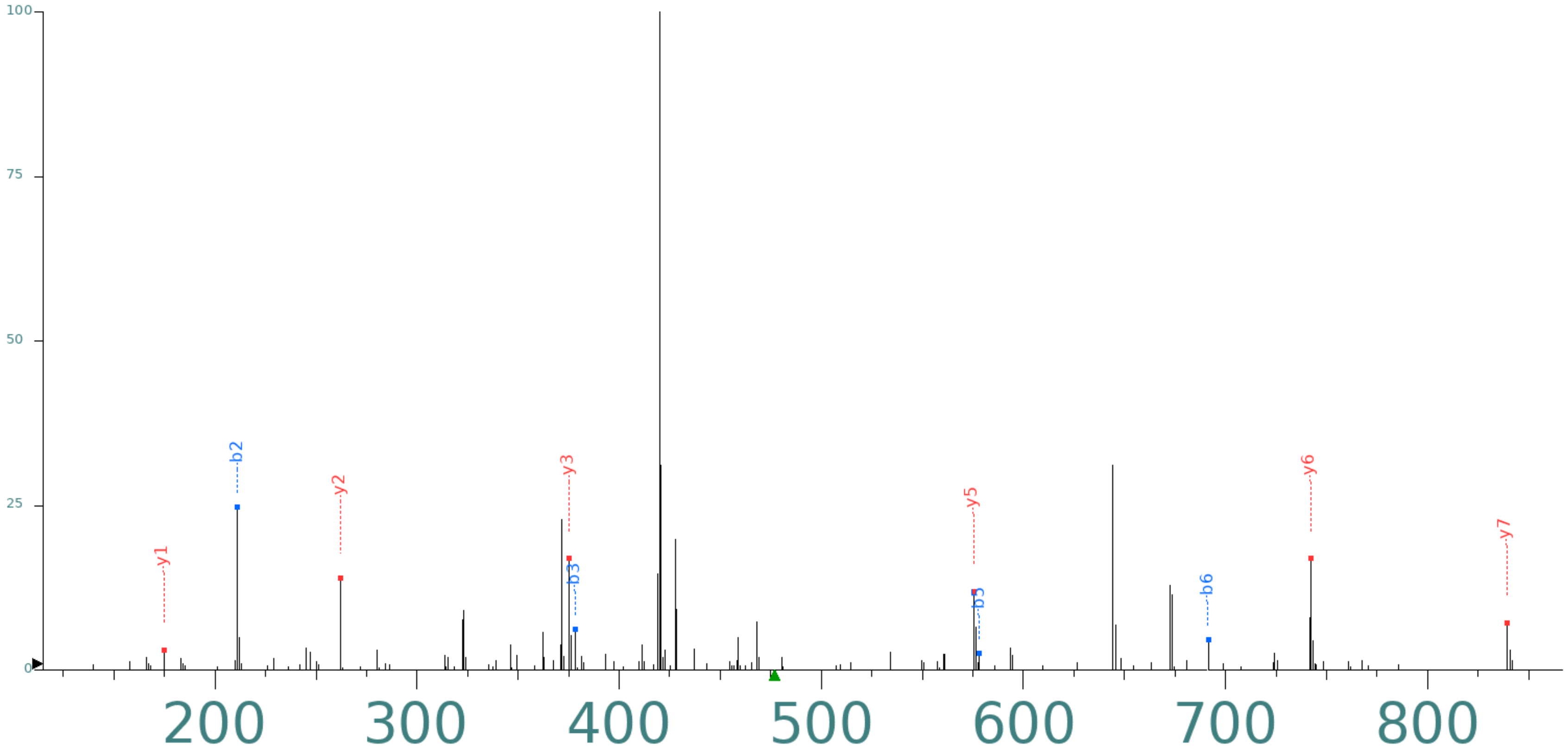

Predicted Fragmentation Pattern

Seq	#	b: Δ Error	b	y	y: Δ Error	+1
I	1	---	114.091	---	---	8
P	2	-696.562	211.144	839.402	95.635	7
S#	3	-186.478	378.142	742.349	136.699	6
T	4	---	479.190	575.351	85.492	5
V	5	269.484	578.259	474.303	---	4
L	6	440.748	691.343	375.235	259.621	3
S	7	---	778.375	262.151	-430.485	2
R	8	---	---	175.119	-691.857	1

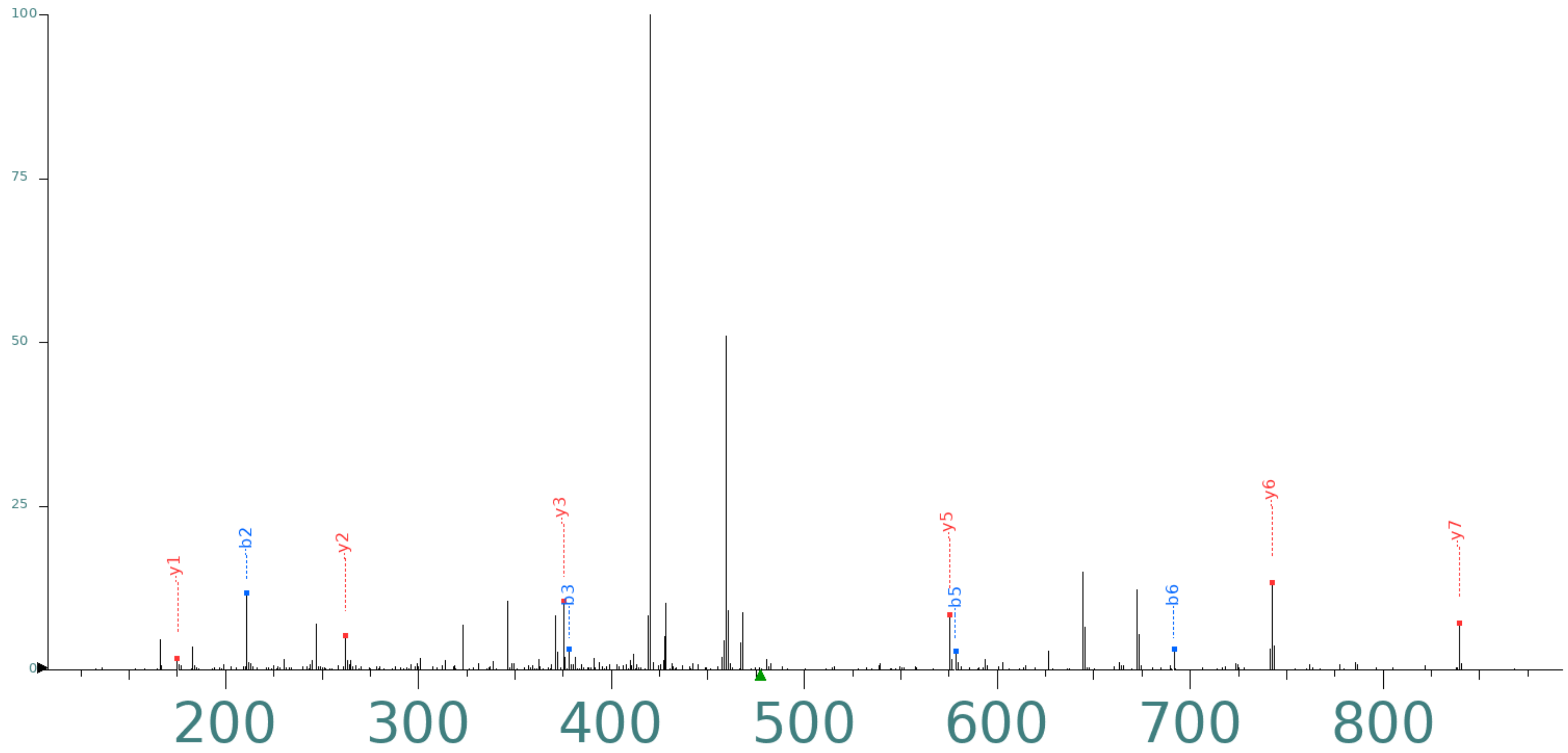

Predicted Fragmentation Pattern

+1

Seq	#	b: Δ Error	b	y	y: Δ Error	+1
R	1	---	157.108	---	---	12
P	2	-536.479	**254.161**	1292.672	---	11
S#	3	---	421.160	1195.619	---	10
R	4	104.874	**577.261**	1028.621	---	9
I	5	584.038	**690.345**	872.520	---	8
P	6	---	787.397	**759.436**	17.016	7
S	7	---	874.429	**662.383**	348.114	6
T	8	---	975.477	**575.351**	-97.928	5
V	9	---	1074.546	**474.303**	574.645	4
L	10	---	1187.630	**375.235**	177.352	3
S	11	---	1274.662	262.151	---	2
R	12	---	---	**175.119**	535.005	1

+2

Seq	#	b: Δ Error	b	y	y: Δ Error	+1
R	1	---	79.058	---	---	12
P	2	---	127.584	**646.840**	-303.314	11
S#	3	---	211.083	**598.313**	764.276	10
R	4	-695.753	**289.134**	**514.814**	-16.918	9
I	5	-508.643	**345.676**	**436.764**	-1059.248	8
P	6	---	394.202	**380.222**	-98.049	7
S	7	-931.608	**437.718**	**331.695**	657.770	6
T	8	---	488.242	288.179	---	5
V	9	---	537.776	**237.655**	90.511	4
L	10	672.160	**594.318**	188.121	---	3
S	11	---	637.834	131.579	---	2
R	12	---	---	88.063	---	1

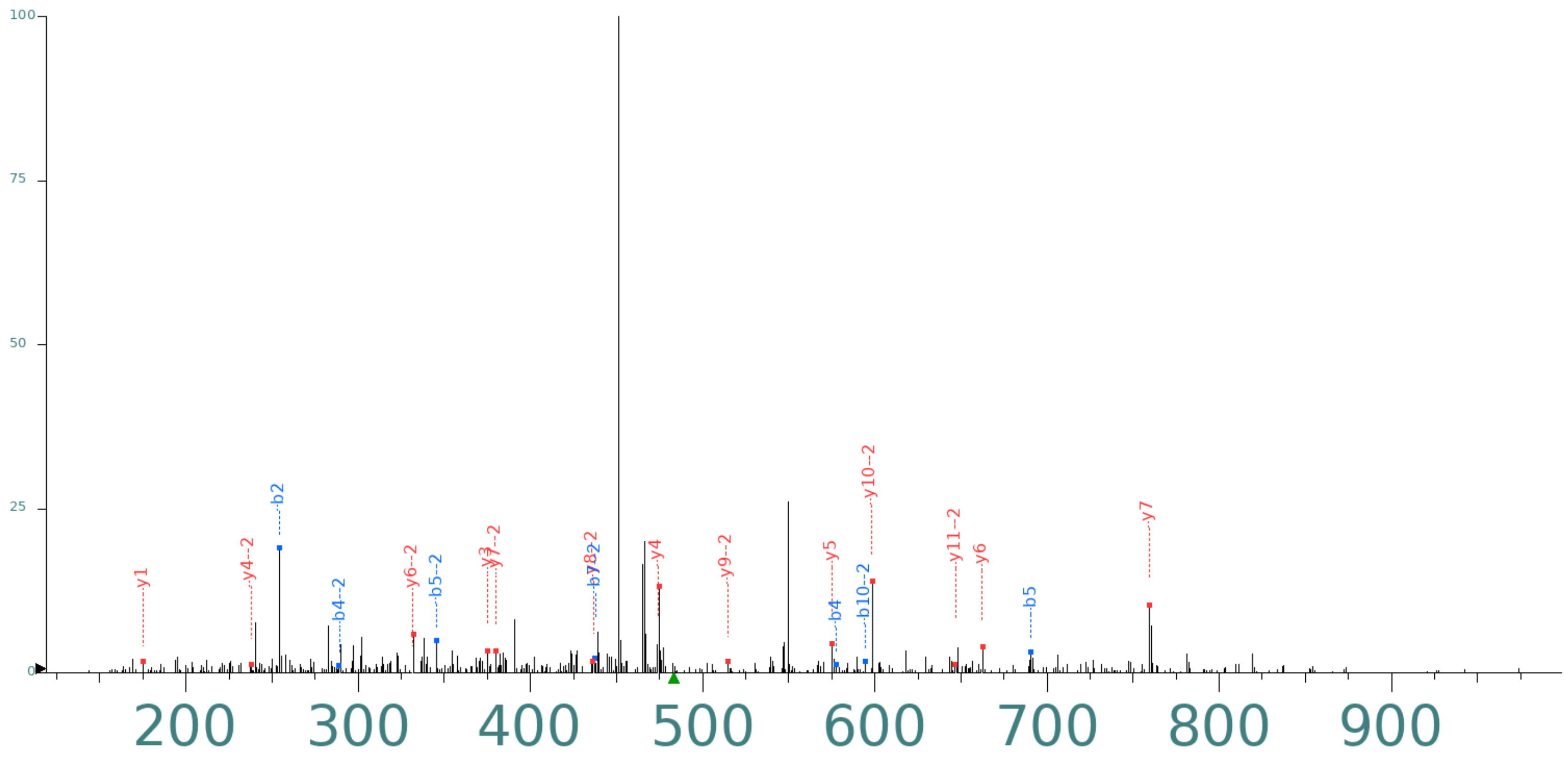

Predicted Fragmentation Pattern

Seq	#	b: Δ Error	b	y	y: Δ Error	+1
I	1	---	114.091	---	---	8
P	2	-536.365	211.144	839.402	413.373	7
S#	3	157.330	378.142	742.349	67.321	6
T	4	---	479.190	575.351	402.951	5
V	5	171.577	578.259	474.303	---	4
L	6	591.294	691.343	375.235	-176.756	3
S	7	---	778.375	262.151	-381.086	2
R	8	---	---	175.119	8.219	1

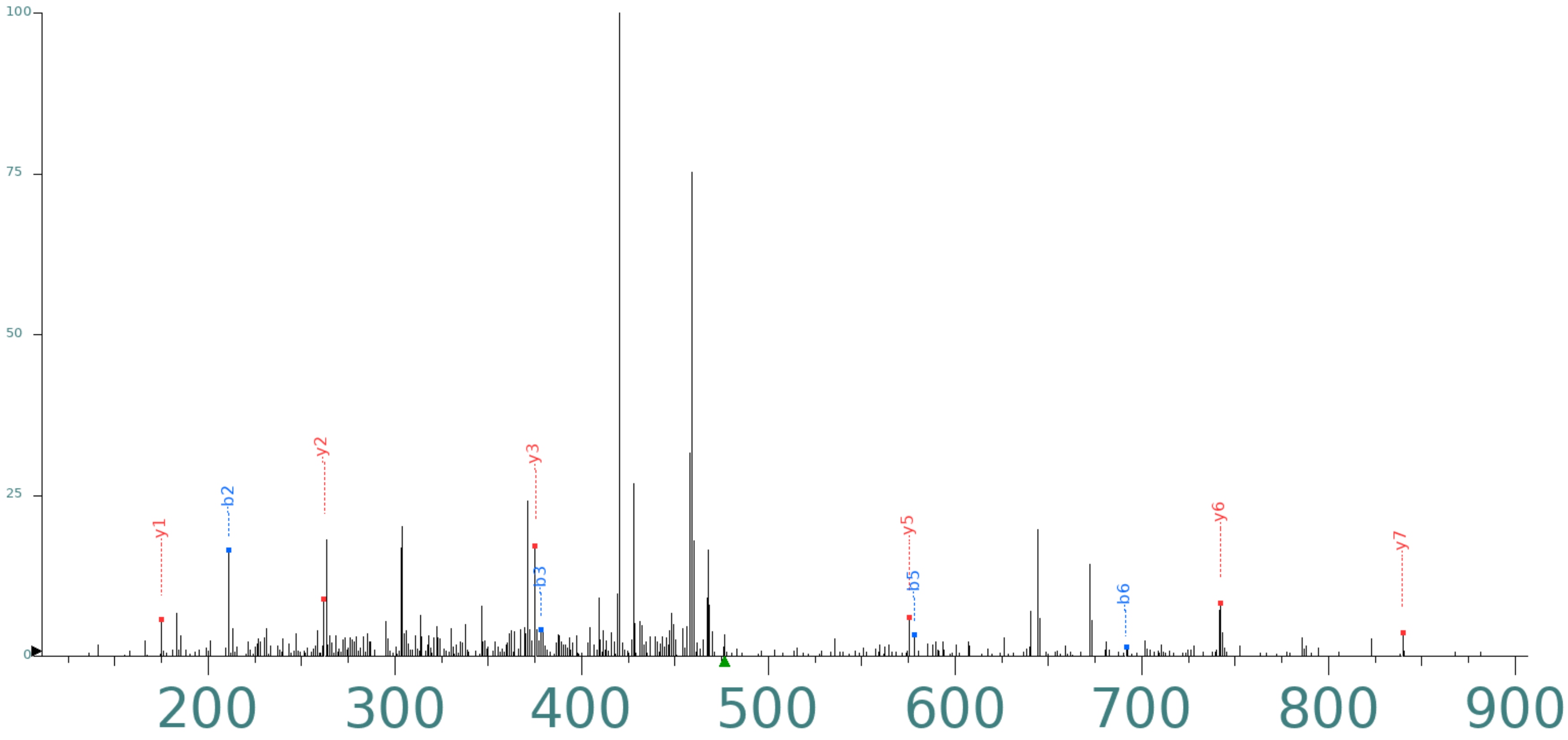

Predicted Fragmentation Pattern

Seq	#	b: Δ Error	b	y	y: Δ Error	+1
I	1	---	114.091	---	---	8
P	2	-951.506	211.144	839.402	398.552	7
S#	3	190.166	378.142	742.349	295.642	6
T	4	---	479.190	575.351	-181.757	5
V	5	473.677	578.259	474.303	---	4
L	6	456.007	691.343	375.235	187.758	3
S	7	---	778.375	262.151	609.007	2
R	8	---	---	175.119	-507.348	1

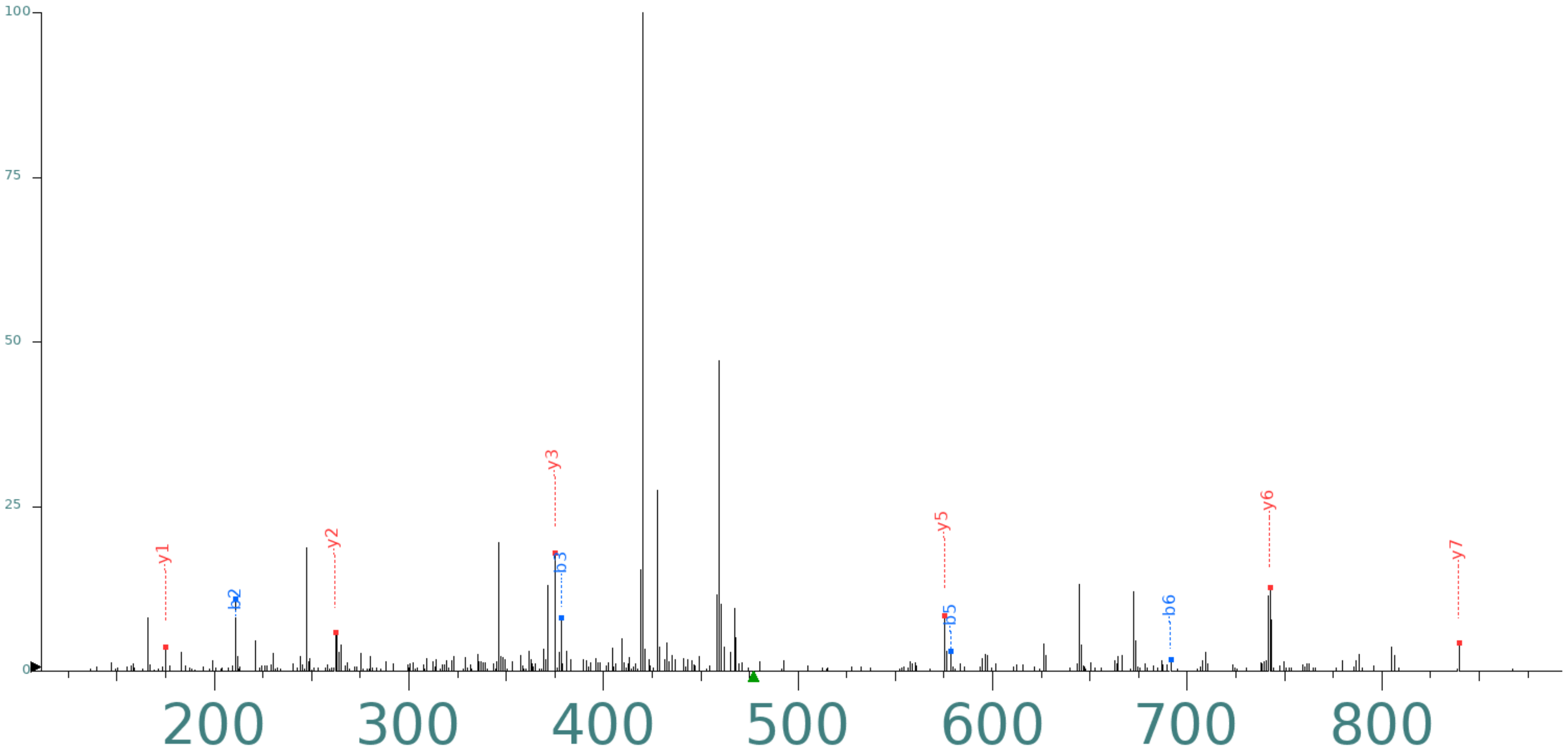

Predicted Fragmentation Pattern

Seq	#	b: Δ Error	b	y	y: Δ Error	+1
I	1	---	114.091	---	---	8
P	2	-47.698	211.144	839.402	253.003	7
S#	3	417.612	378.142	742.349	---	6
T	4	---	479.190	575.351	12.408	5
V	5	46.528	578.259	474.303	338.148	4
L	6	---	691.343	375.235	181.254	3
S	7	---	778.375	262.151	189.210	2
R	8	---	---	175.119	838.337	1

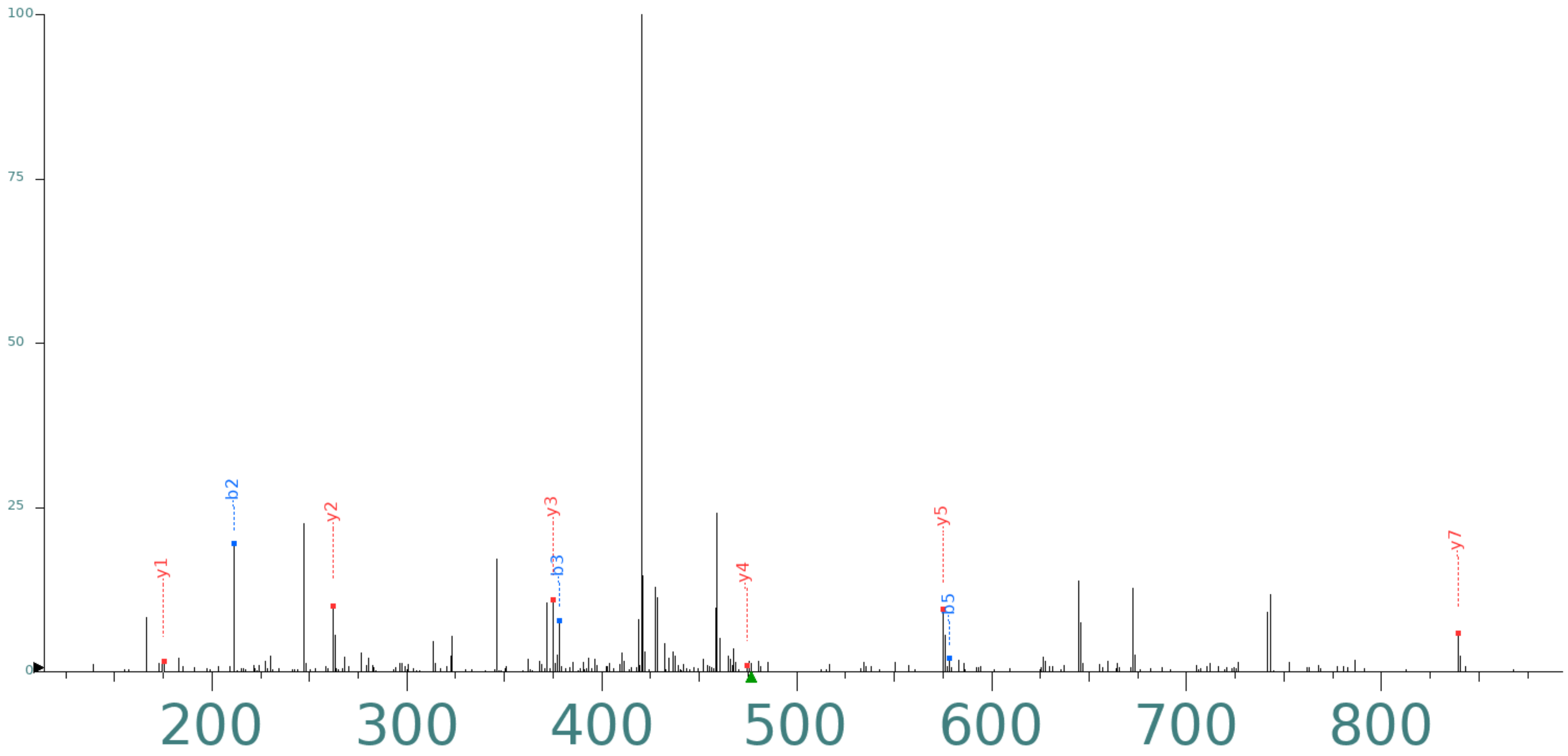

Predicted Fragmentation Pattern

Seq	#	b: Δ Error	b	y	y: Δ Error	+1
I	1	---	114.091	---	---	8
P	2	476.737	211.144	839.402	340.061	7
S#	3	---	378.142	742.349	666.667	6
T	4	---	479.190	575.351	214.981	5
V	5	---	578.259	474.303	---	4
L	6	234.830	691.343	375.235	455.403	3
S	7	---	778.375	262.151	48.036	2
R	8	---	---	175.119	730.373	1

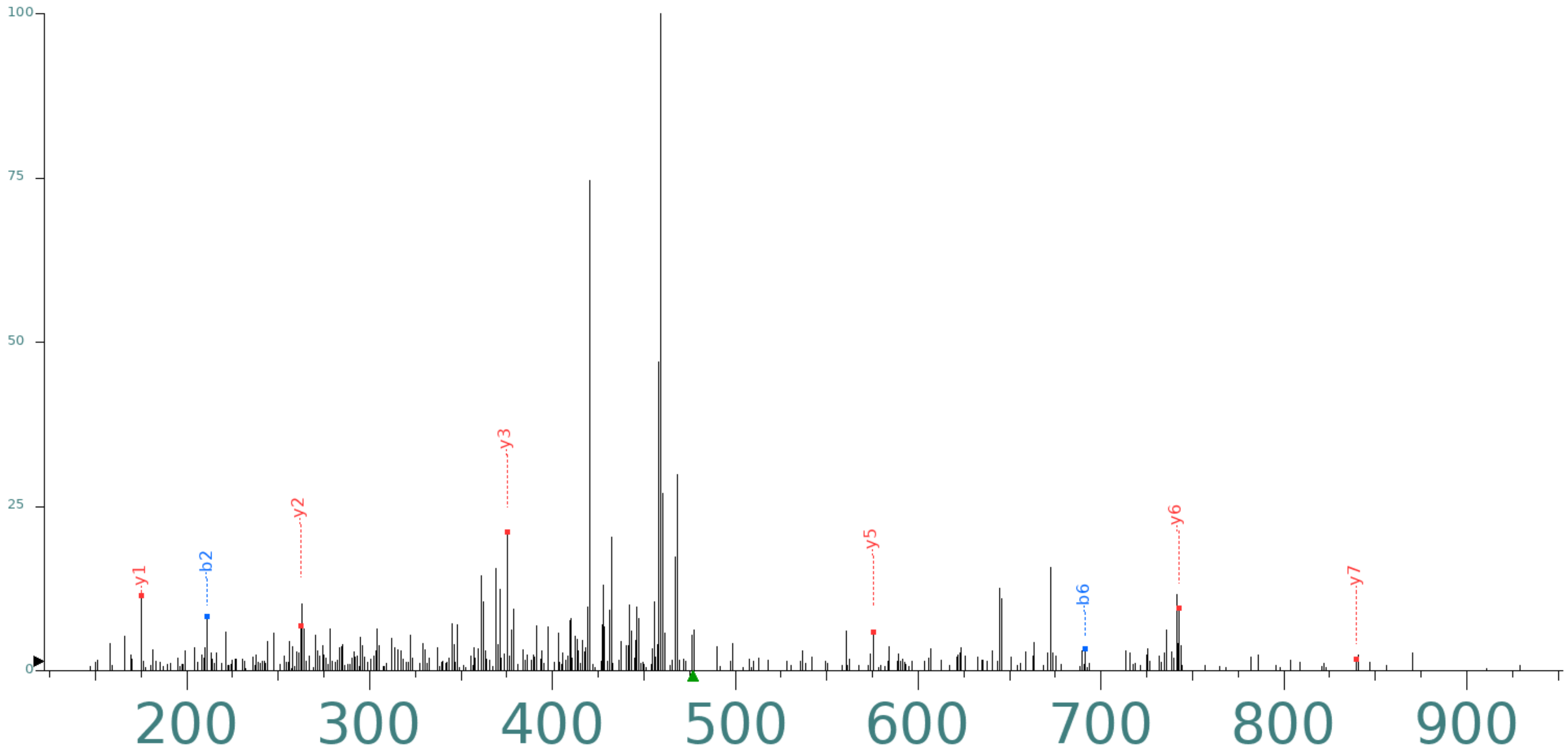

Supplement: Supplementary file 1 [file ijms-25-06491-s001.zip › Supplementary-Mass Spectrometry Spectra.pdf]
